# Author Correction: Multistable and dynamic CRISPRi-based synthetic circuits

**DOI:** 10.1038/s41467-021-23622-x

**Published:** 2021-05-19

**Authors:** Javier Santos-Moreno, Eve Tasiudi, Joerg Stelling, Yolanda Schaerli

**Affiliations:** 1 Department of Fundamental Microbiology, University of Lausanne, Biophore Building, 1015 Lausanne, Switzerland; 2 Department of Biosystems Science and Engineering, ETH Zurich and SIB Swiss Institute of Bioinformatics, Basel, Switzerland

**Keywords:** CRISPR-Cas systems, Dynamic networks, Genetic circuit engineering, Multistability, Synthetic biology

Correction to: *Nature Communications* 10.1038/s41467-020-16574-1, published online 02 June 2020.

The original version of the Supplementary information associated with this Article did not include Supplementary code for the analysis. This has now been added to the Source data file and the Code availability statement has been corrected from “The code for the analysis is available as Supplementary folder,” to read “The code for the analysis is available as a Supplementary folder within the Source data file”. The HTML has been updated to include a corrected version of the Source data and the PDF and HTML versions of the Article have been corrected.

## Supplementary information

Supplementary Information

