# Supplementary material for: Multistable and dynamic CRISPRi-based synthetic circuits
Source: Nat Commun. 2020 Jun 2;11:2746. doi: 10.1038/s41467-020-16574-1 (PMC7265303; doi:10.1038/s41467-020-16574-1)
Supplement: Supplementary file 1 — Supplementary Information [file 41467_2020_16574_MOESM1_ESM.pdf]

Supplementary Information

Multistable and dynamic CRISPRi-based synthetic circuits

Javier Santos-Moreno[1], Eve Tasiudi[2], Joerg Stelling[2] and Yolanda Schaerli[1]

[1]Department of Fundamental Microbiology, University of Lausanne, Biophore Building, 1015 Lausanne, Switzerland

[2]Department of Biosystems Science and Engineering, ETH Zurich and SIB Swiss Institute of Bioinformatics, Basel, Switzerland

yolanda.schaerli@unil.ch

Name	Sense	Annealing site*	Purpose	Sequence(5'-->3')**	Reference
PR 79	For	upstream MCS (in P(BAD) region)	colony-PCR of node 1 (N1) and whole circuit, sequencing of N1	ACGGCGTCACACTTTGC	†
PR 387	For	ColA ori	colony-PCR of node 1 (N1) and whole circuit, sequencing of N1 (when P(BAD) absent)	CTTCGATCAAACCGCCTCCC	This work
PR 75	For	upstream MCS (in CloDF13 vector)	colony-PCR of node 1 (N1) and whole circuit, sequencing of N1 (in CloDF13 vector)	GCCCACCGGAAGGAGCTG	This work
PR 72	Rev	5' end of mKO2	colony-PCR and sequencing of N1 regulatory region (when using mKO2)	TCATCTCTGGTTTGATAACCGAAACCAT	†
PR 69	Rev	5' end of mCherry	colony-PCR and sequencing of N1 regulatory region (when using mCherry)	CCTCGCCCTTGCTCACCAT	This work
PR 80	Rev	spacer 1	colony-PCR and sequencing of N1 (also covers reporter)	AACTGGGTGGGACTCGGC	†
PR 81	For	spacer 1	colony-PCR and sequencing of N2	GCGAGAACTGCTGCCTGG	†
PR 68	Rev	5' end of sfGFP	colony-PCR and sequencing of N2 regulatory region (when using sfGFP)	GTAAACAGTTCTTCGCCTTTACGCAT	†
PR 70	Rev	5' end of mCitrine	colony-PCR and sequencing of N2 regulatory region (when using mCitrine)	CCAGTGAATAATTCTTCACCTTTAGACAT	This work
PR 87	Rev	spacer 2.5	colony-PCR and sequencing of N2 (also covers reporter)	CACTGAAAAGTCTACGGAACTGCTGAG	†
PR 86	For	spacer 2.5	colony-PCR and sequencing of N3	CTCAGCAGTTCCGTAGACTTTTCAGTG	†
PR 71	Rev	5' end of mKate2	colony-PCR and sequencing of N3 regulatory region (when using mKate2)	GCCATGTTATTTTCTTCTCCTTTACTAACCAT	†
PR 440	Rev	5' end of Cerulean	colony-PCR and sequencing of N3 regulatory region (when using Cerulean)	CACCATCTAATTCAACAAGAATTGGGACAACTCC	This work
PR 89	Rev	downstream MCS	colony-PCR of N3 and whole circuit, sequencing of N3 (in ColA backbone)	GGGCCGTTGCTTCACAACG	†
PR 362	Rev	downstream MCS	colony-PCR of N3 and whole circuit, sequencing of N3 (in pSC101 backbone)	GCCGGATCTGCGATTCTGATAACAAAC	This work
PR 14	For	Prefix	Adds Linker_14 overlap to insert N1 during Step 1	AGGATAGATTCTGGAAACTTTACCGTCCGAGCTC**CAGCCTGCGGTCCGG**	†
PR 145	Rev	Suffix	Adds ECK120029600 Terminator overlap to insert N1 during Step 1	GCGGTCTTAAGTTTTTTGGCTGAACCATGG**CGGGCGTCCCAGCGA**	†
PR 15	Rev	Suffix	Adds Linker_0 overlap to insert N1 during Step 1 (when mKO2 reporter is present)	ACGGAGAAGCCCTATCAACTGTTTATTGCTCGAG**CGGGCGTCCCAGCGA**	†
PR 16	For	Prefix	Adds Spacer 1 overlap to insert N2 during Step 1	CGAAATCCCTGAAACTGAGACTGTAGAAAATAAGCTT**CAGCCTGCGGTCCGG**	†
PR 153	Rev	Suffix	Adds L3S2P21 Terminator overlap to insert N2 during Step 1	CTCTTTTCTGGAATTTGGTACCGAGCCTGCAGG**CGGGCGTCCCAGCGA**	†
PR 19	Rev	Suffix	Adds Linker_10 overlap to insert N2 during Step 1 (when sfGFP reporter is present)	ACAGTGCTCAATCGTGTAGAAATCTCTTGGATCC**CGGGCGTCCCAGCGA**	†
PR 20	For	Prefix	Adds Spacer 2.5 overlap to insert N3 during Step 1	AGAAGTATTGGTAATCGTTGAAAACTCAGTCGAC**CAGCCTGCGGTCCGG**	†
PR 147	Rev	Suffix	Adds ECK120033737 Terminator overlap to insert N3 during Step 1	CGGGCTTTTTTCTGTGTTTCCGCGGCCGC**CGGGCGTCCCAGCGA**	†
PR 23	Rev	Suffix	Adds Linker_11 overlap to insert N3 during Step 1 (when mKate2 reporter is present)	CTTCAGTTTCCAACGACGAGTGTAATAGGACGTC**CGGGCGTCCCAGCGA**	†
PR 4	For	Prefix	Adds Linker_1 (For)	GGGACTACACTTACGAAACTATTGATTGCT**CAGCCTGCGGTCCGG**	This work
PR 3	Rev	Suffix	Adds Linker_1 (Rev)	AGCAATCAATAGTTTCGTAAGTGTAGTCCC**CGGGCGTCCCAGCGA**	This work
PR 6	For	Prefix	Adds Linker_17 (For)	GTAGTGCTTATCAGACCCAATACTGTTGAA**CAGCCTGCGGTCCGG**	This work
PR 5	Rev	Suffix	Adds Linker_17 (Rev)	TTCAACAGTATTGGGTCTGATAAGCACTAC**CGGGCGTCCCAGCGA**	This work
PR 8	For	Prefix	Adds Linker_19 (For)	TGAACAGTTGCTCTGATTGAAACCACGATT**CAGCCTGCGGTCCGG**	This work
PR 7	Rev	Suffix	Adds Linker_19 (Rev)	AATCGTGGTTTCAATCAGAGCAACTGTTCA**CGGGCGTCCCAGCGA**	This work
PR 162	For	Prefix	Adds Linker_21 (For)	AGAGCCGAATCGCACTTATTTACAGTAGTT**CAGCCTGCGGTCCGG**	This work
PR 163	Rev	Suffix	Adds Linker_21 (Rev)	AACTACTGTAAATAAGTGCGATTCGGCTCT**CGGGCGTCCCAGCGA**	This work
PR 18	For	Prefix	Adds Linker_24 (For)	AGATAGCCGTTACACAGGTGACACTTATTT**CAGCCTGCGGTCCGG**	This work
PR 17	Rev	Suffix	Adds Linker_24 (Rev)	AAATAAGTGTCACCTGTGTAACGGCTATCT**CGGGCGTCCCAGCGA**	This work
PR 22	For	Prefix	Adds Linker_25 (For)	AGTGACGACTGCGAAGTAACCTCTATTTAT**CAGCCTGCGGTCCGG**	This work
PR 21	Rev	Suffix	Adds Linker_25 (Rev)	ATAAATAGAGGTTACTTCGCAGTCGTCACT**CGGGCGTCCCAGCGA**	This work

*MCS, multiple cloning site.

** **Prefix** and **Suffix** sequences (Casini et al. 2014, Nucleic Acids Res. 42, e7) are indicated in green and red, respectively.

Supplementary Table 1. Primers used in this study. †Primers from Santos-Moreno & Schaerli 2019, ACS Synth Biol. 8, 1691-1697.

Name	Addgene ID	Resistance	Ori	Relevant features	Related figure(s)
pC-0	124421†	Kanamycin	ColA	Empty multiple cloning site	Used for non-fluorescent control bacteria
pC-0_v2	124422†	Ampicillin	ColA	Empty multiple cloning site	Used for non-fluorescent control bacteria
pJ1996_v2	140664†	Spectinomycin	CloDF13	dCas9 & Csy4	Fig. 1, Fig. 4, Fig. 5, Suppl. Fig. 1, Suppl. Fig. 2, Suppl. Fig. 3, Suppl. Fig. 4, Suppl. Fig. 6
pJ2018	140665	Spectinomycin	CloDF13	dCas9, LuxR & Csy4	Fig. 2
pJ2077.2	140666	Ampicillin	pSC101	Controller plasmid for TS circuit and cL and cR controls	Fig. 2
pJ2076.2_TS	140667	Kanamycin	ColA	Toggle switch (TS)	Fig. 2
pJ2076.2_cL	140668	Kanamycin	ColA	cL control for toggle switch experiment	Fig. 2
pJ2076.2_cR	140669	Kanamycin	ColA	cR control for toggle switch experiment	Fig. 2
pJ2042.2	140670	Kanamycin	ColA	3-color stripe	Fig. 4a, b, c & d, Suppl. Fig. 1
pJ2042.2_Bs	140671	Kanamycin	ColA	1-color stripe (BLUE)	Fig. 4e, f, g & h, Suppl. Fig. 3
pJ2048.2_Gs	140672	Ampicillin	ColA	1-color stripe (GREEN)	Fig. 4e & f, Suppl. Fig. 3
pJ2048_2xNOT	140673	Ampicillin	ColA	Double-inverter	Fig. 4g & h
pJ2072.2_CRISPRlator	140674	Kanamycin	ColA	CRISPlator	Fig. 5
pJ2072.2_c1	140675	Kanamycin	ColA	CRISPlator control (open ring)	Suppl. Fig. 6
pJ2044	140676	Kanamycin	ColA	NOT gate with sg-1	Fig. 1
pJ2043	140677	Kanamycin	ColA	NOT gate with sg-2	Fig. 1
pJ2039	140678	Kanamycin	ColA	NOT gate with sg-3	Fig. 1
pJ2040	140679	Kanamycin	ColA	NOT gate with sg-4	Fig. 1
pJ2044_N2only	140680	Kanamycin	ColA	Control ("C") lacking sg-1	Fig. 1
pJ2043_N2only	140681	Kanamycin	ColA	Control ("C") lacking sg-2	Fig. 1
pJ2039_N2only	140682	Kanamycin	ColA	Control ("C") lacking sg-3	Fig. 1
pJ2040_N2only	140683	Kanamycin	ColA	Control ("C") lacking sg-4	Fig. 1
pJ2044_t4	140684	Kanamycin	ColA	NOT gate with sg-1t4	Fig. 1
pJ2040_t4	140685	Kanamycin	ColA	NOT gate with sg-4t4	Fig. 1
pJ2042.2_GFPonly	140686	Kanamycin	ColA	1-color stripe	Suppl. Fig. 2a
pJ2048.2	140687	Kanamycin	ColA	3-color stripe (different sgRNAs)	Suppl. Fig. 2b
pJ2042.2_invRep	140688	Kanamycin	ColA	3-color stripe (inverted reporters)	Suppl. Fig. 2c
pJ2042.2_Brocc	140689	Kanamycin	ColA	Broccoli stripe	Suppl. Fig. 4

Supplementary Table 2. Plasmids used in this study. †Plasmids from Santos-Moreno & Schaerli 2019, ACS Synth Biol. 8, 1691-1697. The rest of the plasmids were constructed in this study.

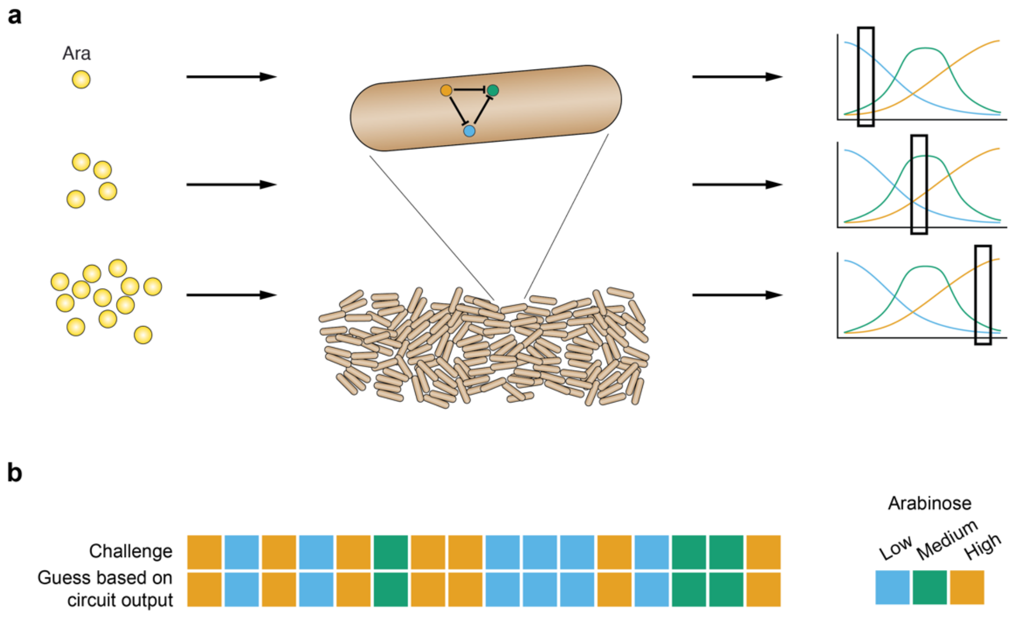

Supplementary Fig. 1. Bacteria carrying the CRISPRi stripe network (Fig. 4) work as a biosensor for Ara concentration determination. (**a**) Engineered bacteria bearing the IFFL circuit exhibit distinct levels of the three fluorescent reporters depending on the Ara concentration they are subjected to. (**b**) 16 blinded solutions with low, medium or high Ara were used to challenge the engineered bacteria, and the identity of the challenge was identified with 100% accuracy based on the fluorescence readout. Data from three biological replicates. Source data are provided as a Source Data file.

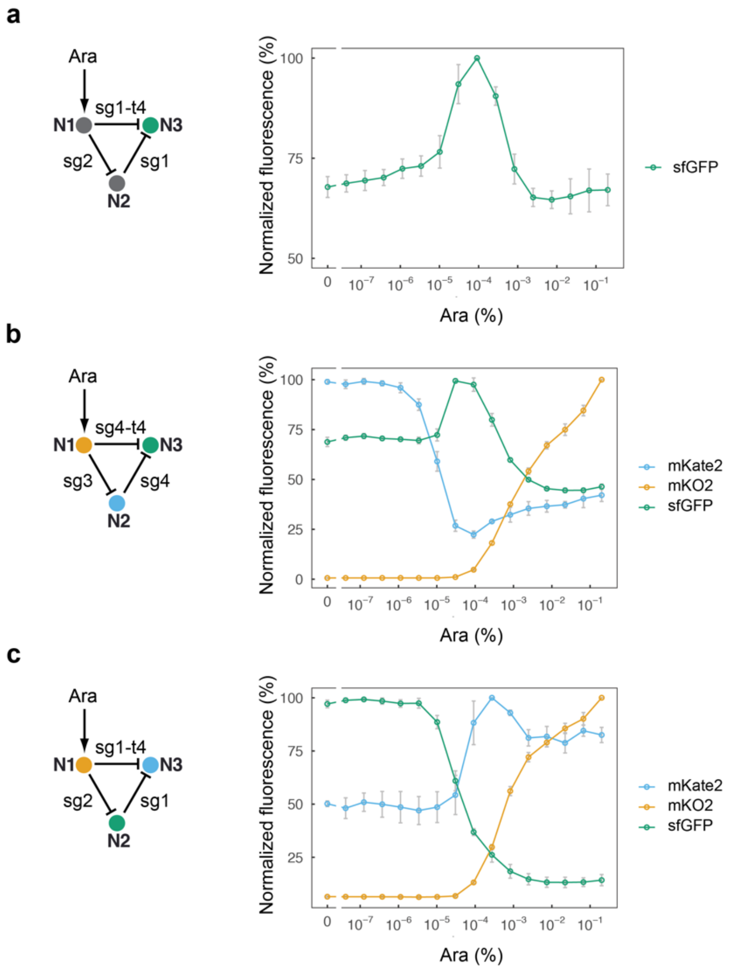

Supplementary Fig. 2. Robustness of the CRISPRi IFFL to variations in its design. (**a**) Design and behavior of a circuit with only one reporter (sfGFP-MarAn20 in N3) instead of three. (**b**) Design and behavior of a network relying on a different set of regulators. (**c**) Design and behavior of a circuit in which reporters for N2 and N3 are inverted compared to the original design. All networks still display a stripe and thus showcase the robustness of the design. Mean and s.d. of three biological replicates. Source data are provided as a Source Data file.

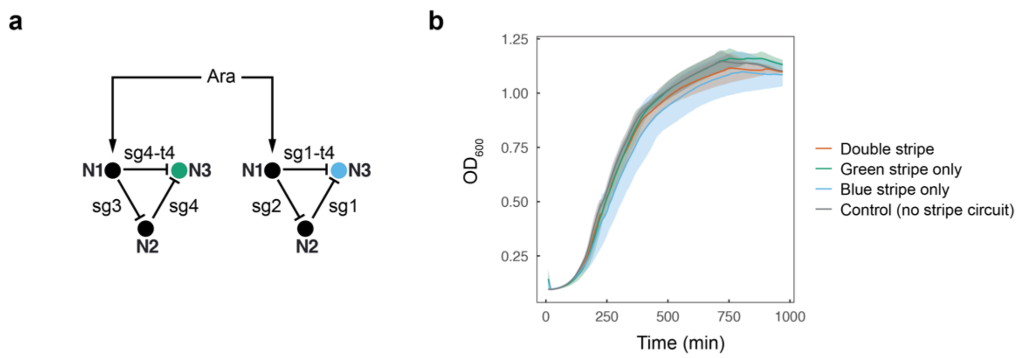

Supplementary Fig. 3. Increase of CRISPRi circuit complexity does not impact cell growth. (**a**) Design of the double stripe-forming two-IFFL circuit. (**b**) Growth curves of bacteria carrying the double stripe circuit depicted in (a) compared to cells carrying either the blue stripe or the green stripe circuits only, or cells lacking both stripe networks. Lines and shades correspond to the mean and s.d. of 3 biological replicates, respectively. Source data are provided as a Source Data file.

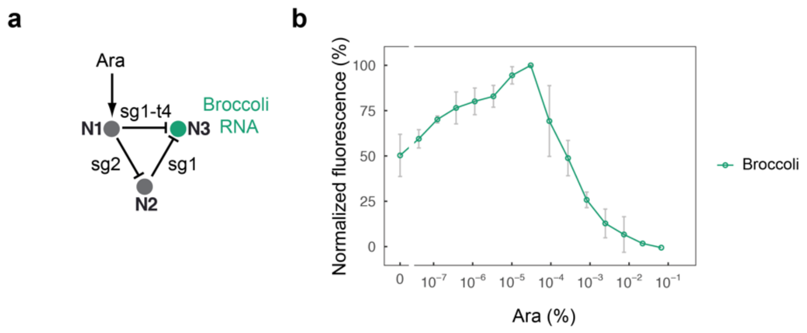

Supplementary Fig. 4. A RNA-reported and RNA-controlled IFFL. (**a**) Design of the synthetic circuit. (**b**) Stripe behavior of the CRISPRi IFFL as reported by a Broccoli RNA aptamer in the presence of DFHBI-1T. Mean and s.d. of three biological replicates. Source data are provided as a Source Data file.

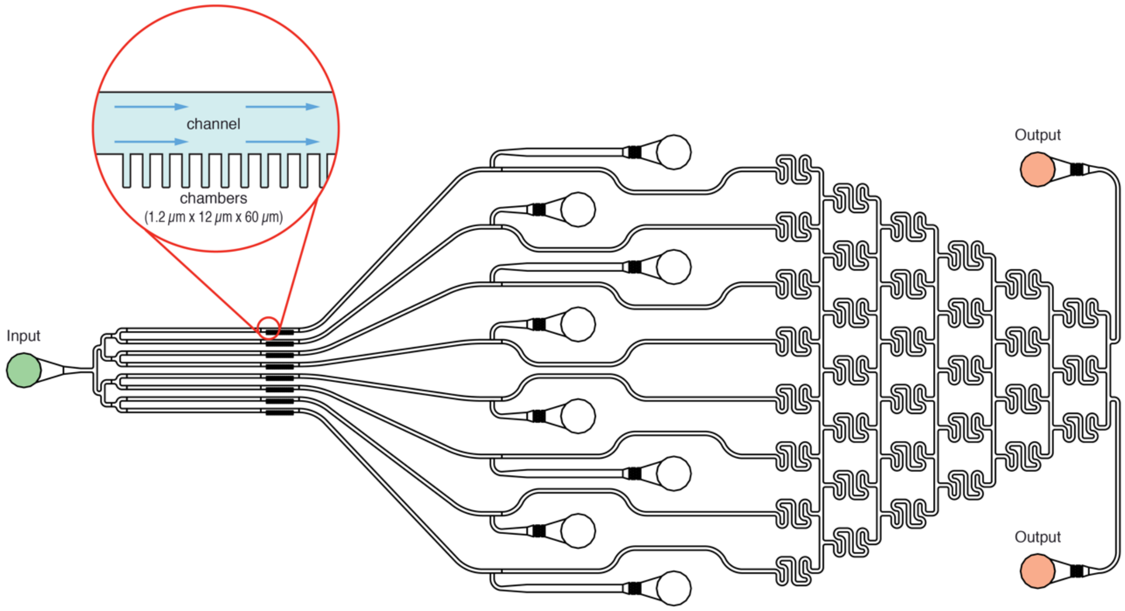

Supplementary Fig. 5. Design of the microfluidic device. Bacteria were grown in chambers (1.2 µm x 12 µm x 60 µm, h x w x l) harboring approximately 110 cells each. The downstream, tree-like design can be used to generate a concentration gradient. This feature was not used in this study.

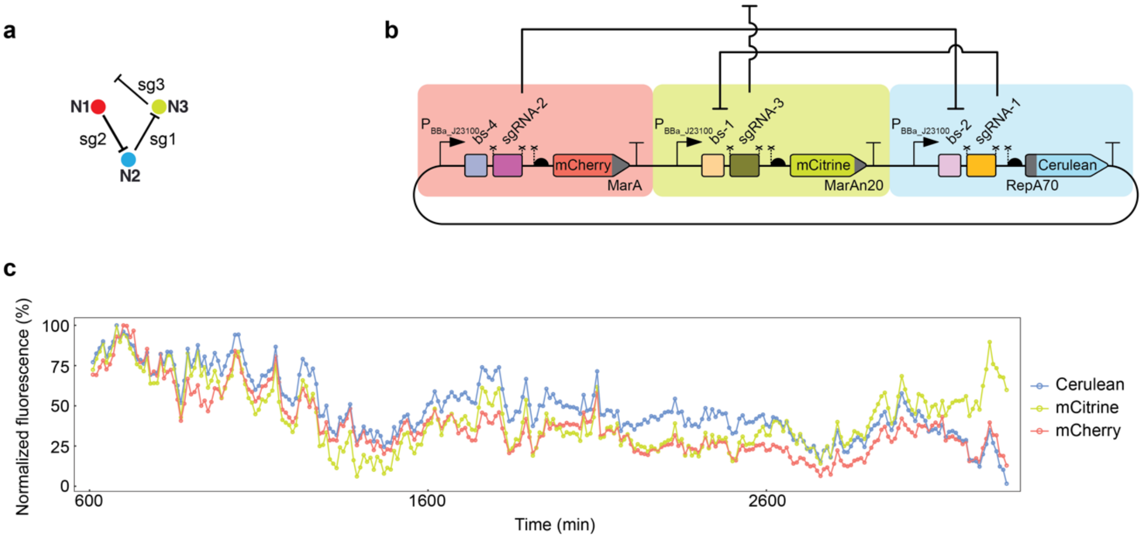

Supplementary Fig. 6. An open-ring control for the CRISPRlator lacking any oscillatory behavior. (**a**) Open-ring topology of the control circuit. (**b**) Molecular implementation. The sgRNA-4 present in the CRISPRlator was replaced with sgRNA-3, which has no cognate binding site in the circuit and is thus unable to close the repression ring. Symbols as in Fig. 1. (**c**) Quantification of the population-level fluorescence over time. Source data are provided as a Source Data file.

Abbreviation	Explanation
G1	Gene 1
G2	Gene 2
G3	Gene 3
G4	Gene 4
sg1	single guide RNA 1
sg2	single guide RNA 2
Cas	dCas9 complex
Cassg1	Cas with sg1
Cassg2	Cas with sg2
G1P2	Cassg2 specifically bound to G1
G2P1	Cassg1 specifically bound to G2
G3P1	Cassg1 specifically bound to G3
G4P2	Cassg2 specifically bound to G4
G1U1	Cassg1 unspecifically bound to G1
G1U2	Cassg2 unspecifically bound to G1
G2U1	Cassg1 unspecifically bound to G2
G2U2	Cassg2 unspecifically bound to G2
G1P2U1	Cassg2 specifically and Cassg1 unspecifically bound to G1
G1P2U2	Cassg2 specifically and Cassg2 unspecifically bound to G1
G2P1U1	Cassg1 specifically and Cassg1 unspecifically bound to G2
G2P1U2	Cassg1 specifically and Cassg2 unspecifically bound to G2

Supplementary Table 3. Abbreviations and functionality of the species used in models of the CRISPRi toggle switch (TS). Note that sgRNA-1 (abbreviated sg1) and sgRNA-2 (sg2) in the model represent two generic sgRNAs, and not necessarily the specific sequences labelled as sgRNA-1 and sgRNA-2 in the experimental parts of this work.

a

b

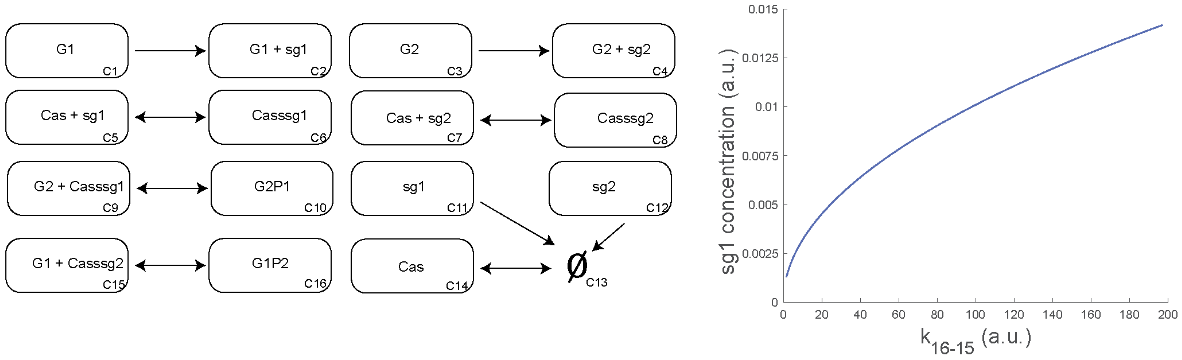

Supplementary Fig. 7. Simplest model for the TS (case 1). (**a**) Model structure assuming that Cassg1/2 complexes bind to a single, specific site on their target genes. Boxes denote complexes in chemical reaction network theory, that is, subsets of reactants (Supplementary Table 3) that are jointly educts or products of a reaction (complex identifiers in lower right corner). Single-headed arrows: irreversible reactions; double-headed arrows: reversible reactions; Ø: source or sink for components. (**b**) Bifurcation diagram of the steady state sg1 concentration upon varying the constant $k_{16\text{-}15}$, which corresponds to the reaction of complexes C16 and C15 (unbinding of the specifically bound Cassg2 to G1). The analysis indicates that the system cannot show bistability.

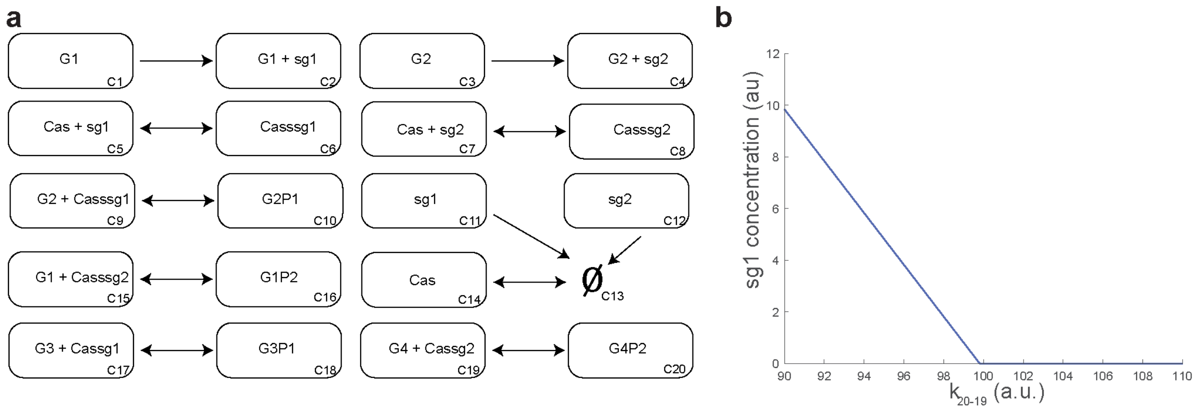

Supplementary Fig. 8. Extended model for the TS (case 2). (**a**) Model structure, assuming that the Cassg1/2 complexes can bind to other sequences in the genome specifically and not only to G1 and G2. Notation as in Supplementary Fig. 7. (**b**) Bifurcation diagram of the steady state sg1 concentration with varying the constant $k_{20\text{-}19}$, which corresponds to the reaction of complexes C20 and C19 (unbinding of the specifically bound Cassg2 to G4).

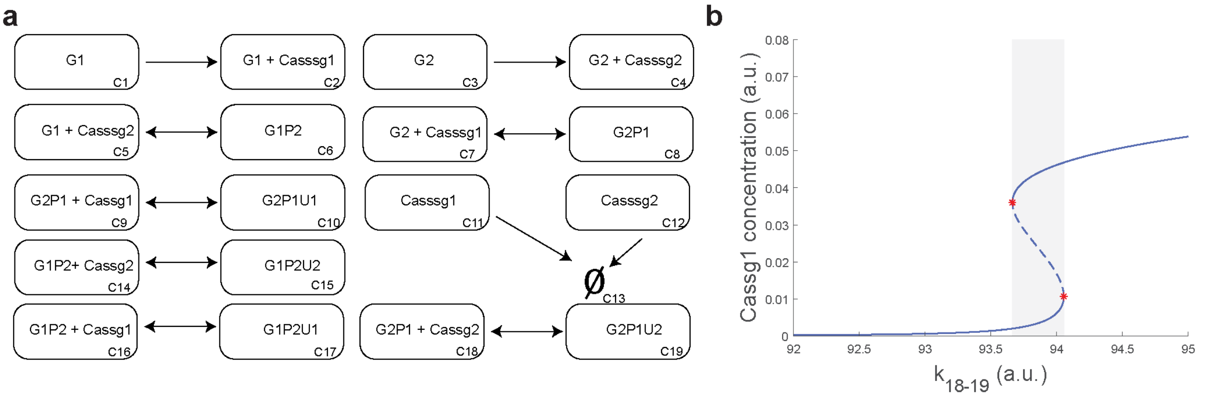

Supplementary Fig. 9. Model for the TS with unspecific binding (case 3). (**a**) Model structure as in Supplementary Fig. 7. The model assumes that we can neglect the formation of the Cassg1/2 complexes and that the Cassg1/2 complexes can bind unspecifically via PAM sequences to G1 and G2. (**b**) Bifurcation diagram of the steady state Cassg1 concentration with varying the constant $k_{18\text{-}19}$, which corresponds to the reaction of complexes C18 and C19 (unspecific binding of Cassg2 to G2P1). Stable and unstable steady states are represented by solid and dashed lines, respectively. Red stars indicate limit points and bistability regions are enclosed in the grey area.

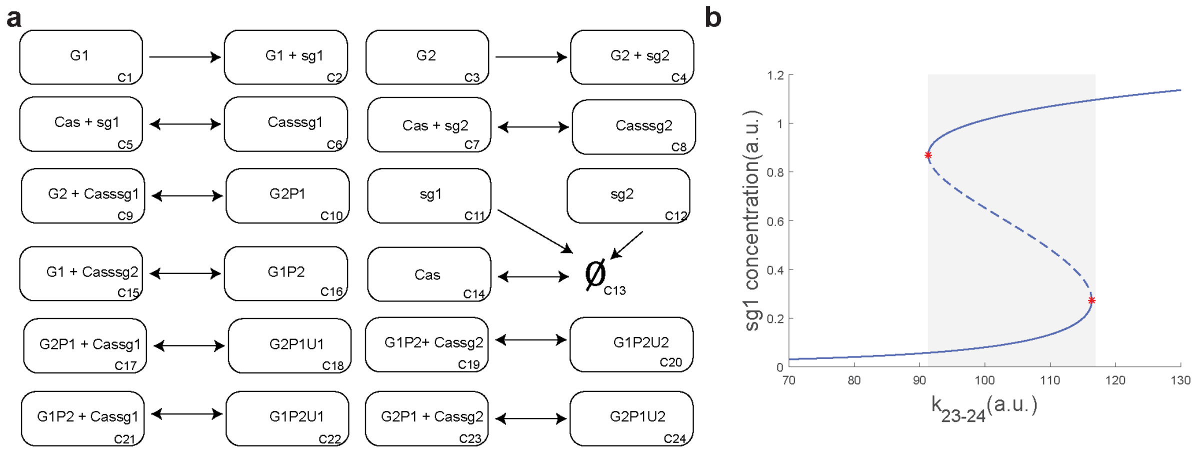

Supplementary Fig. 10. Model for the TS with unspecific binding (case 4). (**a**) Model structure, extension of case 3 (Supplementary Fig. 9) by Cassg1/2 complex formation. (**b**) Bifurcation diagram of the steady state sg1 concentration with varying the constant $k_{23\text{-}24}$, which corresponds to the reaction of complexes C23 and C24 (unspecific binding of Cassg2 to G2P1). Symbols are as in Supplementary Fig. 9.

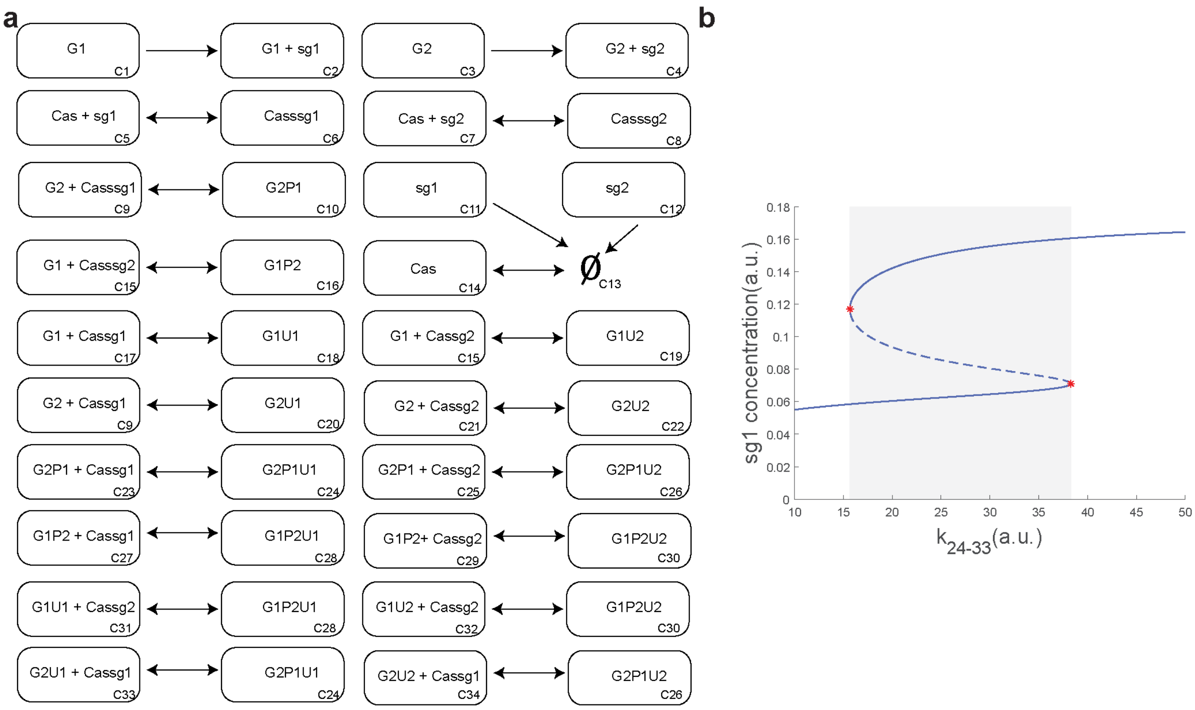

Supplementary Fig. 11. Model for the TS with unspecific binding (case 5). (**a**) Model structure, which extends case 4 (Supplementary Fig. 10) to include the unspecific binding of Cassg1 and Cassg2 to G2 and G1, respectively. (**b**) Bifurcation diagram of the steady state concentration of sg1 with varying constant $k_{24\text{-}33}$, which corresponds to the reaction of complexes C24 and C33 (unbinding of the unspecific complex Cassg1 from G2P1U1). Symbols are as in Supplementary Fig. 9.

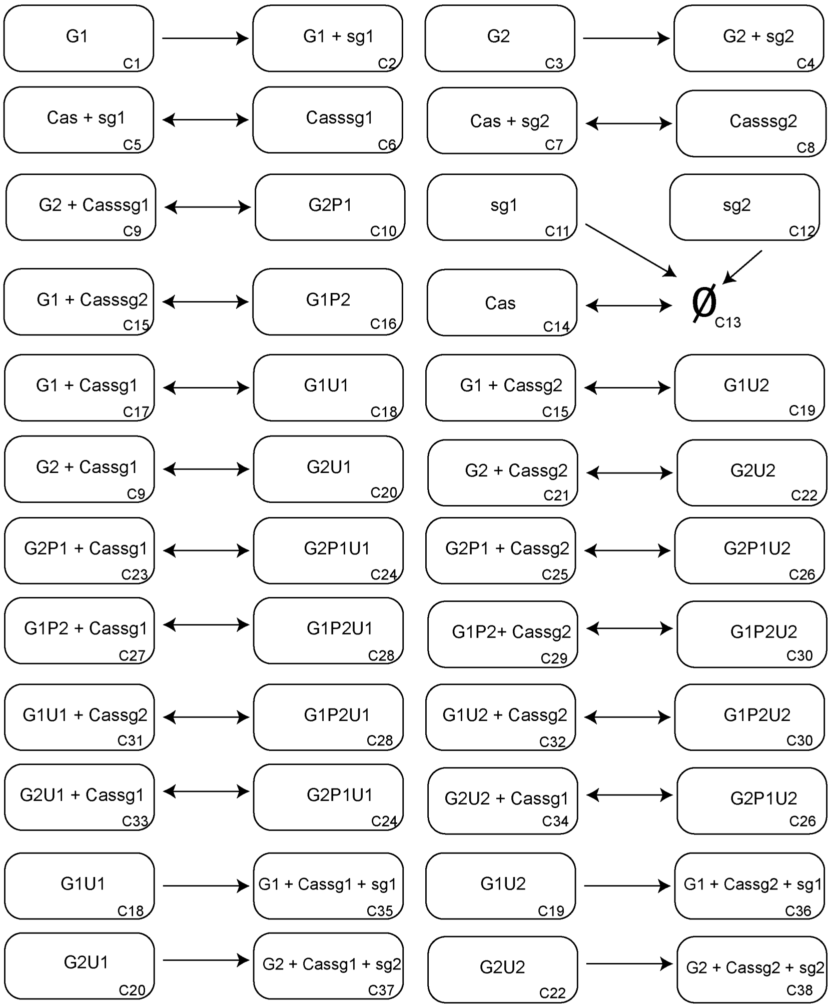

Supplementary Fig. 12. Complete model for the TS. Compared to the other cases, this model assumes that unspecific binding of Cassg1/2 complexes does not affect gene expression. The bifurcation diagram of this model with experimentally constrained parameters (Supplementary Table 4) is shown as Fig. 3b in the main text.

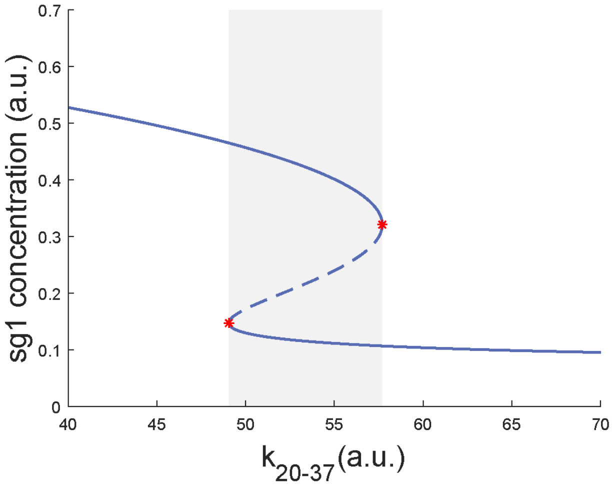

Supplementary Fig. 13. Bifurcation diagram of the complete model without experimentally-derived constraints on parameters. The varying parameter corresponds to the reaction of complexes C20 and C37 (Supplementary Fig. 12) and is similar to the one represented in Fig. 3b (main text). Symbols are as in Supplementary Fig. 9.

Parameters	Process	Lower Bound	Upper Bound	pBest
k_1_2	Production	1	100	50.72434
k_3_4	Production	1	100	42.33746
k_13_14	Production	1	100	1.031285
k_18_35	Production	0.1	100	65.26698
k_19_36	Production	0.1	100	75.94251
k_20_37	Production	0.1	100	68.53327
k_22_38	Production	0.1	100	63.44098
k_5_6	Association	1	100	1.188162
k_7_8	Association	1	100	1.071073
k_6_5	Dissociation	0.01	10	9.601046
k_8_7	Dissociation	0.01	10	9.432942
k_11_13	Degradation	0.001	1	0.986721
k_12_13	Degradation	0.001	1	0.964785
k_14_13	Degradation	0.001	1	0.988306
k_9_10*	Specific binding	1	100	4.612951
k_15_16*	Specific binding	1	100	33.02472
k_31_28*	Specific binding	1	100	42.83338
k_32_30*	Specific binding	1	100	76.30581
k_33_24*	Specific binding	1	100	51.87789
k_34_26*	Specific binding	1	100	65.45974
k_10_9*	Specific unbinding	0.001	0.1	0.093696
k_16_15*	Specific unbinding	0.001	0.1	0.096394
k_28_31*	Specific unbinding	0.001	0.1	0.089324
k_30_32*	Specific unbinding	0.001	0.1	0.094013
k_24_33*	Specific unbinding	0.001	0.1	0.085184
k_26_34*	Specific unbinding	0.001	0.1	0.095339
k_17_18*	Unspecific binding	1	100	76.13001
k_15_19*	Unspecific binding	1	100	94.13042
k_9_20*	Unspecific binding	1	100	85.43037
k_21_22*	Unspecific binding	1	100	94.57936
k_23_24*	Unspecific binding	1	100	53.13512
k_25_26*	Unspecific binding	1	100	86.54855
k_27_28*	Unspecific binding	1	100	89.51009
k_29_30*	Unspecific binding	1	100	65.04785
k_18_17*	Unspecific unbinding	1	100	11.96749
k_19_15*	Unspecific unbinding	1	100	3.707992
k_20_9*	Unspecific unbinding	1	100	12.95706
k_22_21*	Unspecific unbinding	1	100	5.169212
k_24_23*	Unspecific unbinding	1	100	1.177815
k_26_25*	Unspecific unbinding	1	100	1.521193
k_28_27*	Unspecific unbinding	1	100	1.472313
k_30_29*	Unspecific unbinding	1	100	1.446816

Supplementary Table 4. Parameters for the complete model with experimental constraints. Parameter names represent the reaction between the complexes in which they participate; 'k_i_j'

is the rate constant for the irreversible reaction from complex C_i to C_j. During parameter space exploration for identifying a limit point, parameters denoted with * were constrained in a range defined by experimental data from Martens *et al.* 2019, Nat Commun. 10, 3552. Specifically, we allowed for +/- two orders of magnitude for unspecific binding and +/- one order of magnitude for specific binding. pBest is the optimal decision vector.
