# Supplementary material for: Multistable and dynamic CRISPRi-based synthetic circuits
Source: Nat Commun. 2020 Jun 2;11:2746. doi: 10.1038/s41467-020-16574-1 (PMC7265303; doi:10.1038/s41467-020-16574-1)
Supplement: Supplementary file 4 — Supplementary Data 1 [file 41467_2020_16574_MOESM4_ESM.pdf]

(from 1-1070 bp)

pC-0 (4150 bp)

ACTTTTCATACTCCCGCCATTCAGAGAAGAAACCAATTGTCCATATTGCATCAGACATTGCCGTCACTGCGTCTTTTACTGGCTCTTCTCGCTAACCAAACCGGTAA
TGAAAAGTATGAGGGCGGTAAGTCTCTTCTTTGGTTAACAGGTATAACGTAGTCTGTAACGGCAGTGACGCAGAAAATGACCGAGAAGAGCGATTGGTTTGGCCATT

CCCCGCTTATTAAAAGCATTCTGTAACAAAGCGGGACCAAAGCCATGACAAAAACGCGTAACAAAAGTGTCTATAATCACGGCAGAAAAGTCCACATTGATTATTTG
GGGGCGAATAATTTTCGTAAGACATTGTTTCGCCCTGGTTTCGGTACTGTTTTTGCGCATTGTTTTCACAGATATTAGTGCCGTCTTTTCAGGTGTAACTAATAAAC

EcoRI
CACGGCGTCACACTTTGCTATGCCATAGCATTTTTATCCATAAGATTAGCGGtTCCTACCTGACGCTTTTTATCGCAACTCTCTACTGTTTCTCCATACCGAATTCA
GTGCCGCAGTGTGAAACGATACGGTATCGTAAAAATAGGTATTCTAATCGCCaAGGATGGACTGCGAAAAATAGCGTTGAGAGATGACAAAGAGGTATGGCTTAAGT
P(BAD) promoter

SacI XhoI NcoI
TAGGATAGATTCTGGAAACTTTACCGTCCGAGCTCAGGCTTACCTTACTCGAGCAATAAACAGTTGATAGGGCTTCTCCGTTACCATGGTTCAGCCAAAAAACTTAA
ATCCTATCTAAGACCTTTGAAATGGCAGGCTCGAGTCCGAATGGAATGAGCTCGTTATTTGTCAACTATCCCGAAGAGGCAATGGTACCAAGTCGGTTTTTTGAATT
Linker_14 Spa... 8 Linker_0 ECK120...nator

GACCGCCGGTCTTGTCCACTACCTTGCAGTAATGCGGTGGACAGGATCGGCGGTTTTCTTTTCTCTTCTCAATTCTTCTGACCTGTAACGAATAATAGATAGTAAAG
CTGGCGGCCAGAACAGGTGATGGAACGTCATTACGCCACCTGTCCTAGCCGCCAAAAGAAAAGAGAAGAGTTAAGAAGACTGGACATTGCTTATTATCTATCATTTC
ECK120029600 Terminator Spacer 1

TAGTCTCCGATTGAGTTTTCTCTGCCGAGTCCCACCCAGTTCTGTGATTTCAGTAAGTTGGTAATTGATACACTGTTGCGAGAACTGCTGCCTGGTAGTAGATAGGT
ATCAGAGGCTAACTCAAAAGAGACGGCTCAGGGTGGGTCAAGACACTAAAGTCATTCAACCATTAACTATGTGACAACGCTCTTGACGACGGACCATCATCTATCCA
Spacer 1

HindIII BamHI
TGTTATTGAGTAAGAAGGTAAAGTGAACGAAATCCCTGAAACTGAGACTGTAGAAAATAAGCTTGTCCAGACTATTGGATCCAAGAGATTTCTACACGATTGAGCAC
ACAATAACTCATTCTTCCATTTCACTTGCTTTAGGGACTTTGACTCTGACATCTTTTATTCGAACAGGTCTGATAACCTAGGTTCTCTAAAGATGTGCTAACTCGTG
Spacer 1 Spa... 7 Linker_10

SbfI KpnI
TGTCTCCTGCAGGCTCGGTACCAAATTCCAGAAAAGAGGCCTCCCGAAAGGGGGGCCTTTTTTCGTTTTGGTCCTAATAGATAAAGGATAGGTCTGGTAGTGTTGTT
ACAGAGGACGTCCGAGCCATGGTTTAAGGTCTTTTCTCCGGAGGGCTTTCCCCCCGGAAAAAAGCAAAACCAGGATTATCTATTTCCTATCCAGACCATCACAACAA
L3S2P21 Terminator Spacer 2.5

CGTTCTCGCAGGTAAATCAATAATACTCAGCAGTTCCGTAGACTTTTCAGTGGGACAGGGTAGCGATAACAGATAGATTGTAATAAGACACAGTAGGTGCTCGTAGT
GCAAGAGCGTCCATTTAGTTATTATGAGTCGTCAAGGCATCTGAAAAGTCACCCTGTCCCATCGCTATTGTCTATCTAACATTATTCTGTGTCATCCACGAGCATCA
Spacer 2.5

SalI AatII
TGCGTGAAGAGAACCGCTCAGGAAATCCAGTCAGAAGTATTGGTAATCGTTGAAAACTCAGTCGACGCACTTACTGAAGACGTCCTATTACACTCGTCGTTGGAAAC
ACGCACTTCTCTTGGCGAGTCCTTTAGGTCAGTCTTCATAACCATTAGCAACTTTTGAGTCAGCTGCGTGAATGACTTCTGCAGGATAATGTGAGCAGCAACCTTTG
Spacer 2.5 Spa... 5 Linker_11

pC-0 (4150 bp) (from 1071-2354 bp)

NotI

TGAAGATGCGGCCGCggaaacacagAAAAAAGCCCGCACCTGACAGTGCGGGCTTTTTTTTTcgaccaaaggTAGCGAACGACGAGTCACTGTTGAGGATAAATACT
ACTTCTACGCCGGCGcctttgtgtcTTTTTTCGGGCGTGGACTGTCACGCCCGAAAAAAAAAgctggtttccATCGCTTGCTGCTCAGTGACAACTCCTATTTATGA

ECK120033737 Terminator
Linker 8

KasI BbvCI AscI

TTCTCTACTAGGCGCCTGTTACACAGGTCCTCAGCGGCGCGCCTTTGTCGGTGAACGCTCTCCTGAGTAGGACAAATCCGCCGGGAGCGGATTTGAACGTTGTGAAG
AAGAGATGATCCGCGGACAATGTGTCCAGGAGTCGCCGCGCGGAAACAGCCACTTGCGAGAGGACTCATCCTGTTTAGGCGGCCCTCGCCTAAACTTGCAACACTTC

Li...8
Spa...15

CAACGGCCCGGAGGGTGGCGGGCAGGACGCCCGCCATAAACTGCCAGGCATCAAACTAAGCAGAAGGCCATCCTGACGGATGGCCTTTTTGCGTTTCAGATCTACCG
GTTGCCGGGCCTCCCACCGCCCGTCCTGCGGGCGGTATTTGACGGTCCGTAGTTTGATTCGTCTTCCGGTAGGACTGCCTACCGGAAAAACGCAAAGTCTAGATGGC

GTaaaccagcaatagacataagcggctatttaacgaccctgccctgaaccgacgacaagctgacgaccgggtctccgcaagtggcacttttcggggaaatgtgcgcg
CAtttggtcgttatctgtattcgccgataaattgctgggacgggacttggctgctgttcgactgctggcccagaggcgttcaccgtgaaaagcccctttacacgcgc

gaacccctatttgtttatttttctaaatacattcaaatatgtatccgctcatgaattaattcttagaaaaactcatcgagcatcaaatgaaactgcaatttattcat
cttggggataaacaaataaaaagatttatgtaagtttatacataggcgagtacttaattaagaatctttttgagtagctcgtagtttactttgacgttaaataagta

KanR

atcaggattatcaataccatatttttgaaaaagccgtttctgtaatgaaggagaaaactcaccgaggcagttccataggatggcaagatcctggtatcggtctgcga
tagtcctaatagttatggtataaaaactttttcggcaaagacattacttcctcttttgagtggctccgtcaaggtatcctaccgttctaggaccatagccagacgct

KanR

ttccgactcgtccaacatcaatacaacctattaatttcccctcgtcaaaaataaggttatcaagtgagaaatcaccatgagtgacgactgaatccggtgagaatggc
aaggctgagcaggttgtagttatgttggataattaaaggggagcagtttttattccaatagttcactctttagtggtactcactgctgacttaggccactcttaccg

KanR

aaaagtttatgcatttctttccagacttgttcaacaggccagccattacgctcgtcatcaaaatcactcgcatcaaccaaaccgttattcattcgtgattgcgcctg
ttttcaaatacgtaaagaaaggtctgaacaagttgtccggtcggtaatgcgagcagtagttttagtgagcgtagttggtttggcaataagtaagcactaacgcggac

KanR

agcgagacgaaatacgcggtcgctgttaaaaggacaattacaaacaggaatcgaatgcaaccggcgcaggaacactgccagcgcatcaacaatattttcacctgaat
tcgctctgctttatgcgccagcgacaattttcctgttaatgtttgtccttagcttacgttggccgcgtccttgtgacggtcgcgtagttgttataaaagtggactta

KanR

caggatattcttctaatacctggaatgctgttttcccggggatcgcagtggtgagtaaccatgcatcatcaggagtacggataaaatgcttgatggtcggaagaggc
gtcctataagaagattatggaccttacgacaaaagggcccctagcgtcaccactcattggtacgtagtagtcctcatgcctattttacgaactaccagccttctccg

KanR

ataaattccgtcagccagtttagtctgaccatctcatctgtaacatcattggcaacgctacctttgccatgtttcagaaacaactctggcgcatcgggcttcccata
tatttaaggcagtcggtcaaatcagactggtagagtagacattgtagtaaccgttgcgatggaaacggtacaaagtctttgttgagaccgcgtagcccgaagggtat

KanR

caatcgatagattgtcgcacctgattgcccgacattatcgcgagcccatttatacccatataaatcagcatccatgttggaatttaatcgcggcctagagcaagacg
gttagctatctaacagcgtggactaacgggctgtaatagcgctcgggtaaatatgggtatatttagtcgtaggtacaaccttaaattagcgccggatctcgttctgc

KanR

pC-0 (4150 bp) (from 2355-3638 bp)

tttcccgttgaatatggctcatactcttcctttttcaatattattgaagcatttatcagggttattgtctcatgagcggatacatatttgaatgtatttagaaaaat
aaagggcaacttataccgagtatgagaaggaaaaagttataataacttcgtaaatagtcccaataacagagtactcgcctatgtataaacttacataaatcttttta
KanR

aaacaaataggcatgctagcgcagaaacgtcctagaagatgccaggaggatacttagcagagagacaataaggccggagcgaagccgtttttccataggctccgccc
tttgtttatccgtacgatcgcgtctttgcaggatcttctacggtcctcctatgaatcgtctctctgttattccggcctcgcttcggcaaaaaggtatccgaggcggg
ColA ori

ccctgacgaacatcacgaaatctgacgctcaaatcagtggtggcgaaacccgacaggactataaagataccaggcgtttccccctgatggctccctcttgcgctctc
gggactgcttgtagtgctttagactgcgagtttagtcaccaccgctttgggctgtcctgatatttctatggtccgcaaagggggactaccgagggagaacgcgagag
ColA ori

ctgttcccgtcctgcggcgtccgtgttgtggtggaggctttacccaaatcaccacgtcccgttccgtgtagacagttcgctccaagctgggctgtgtgcaagaaccc
gacaagggcaggacgccgcaggcacaacaccacctccgaaatgggtttagtggtgcagggcaaggcacatctgtcaagcgaggttcgacccgacacacgttcttggg
ColA ori

cccgttcagcccgactgctgcgccttatccggtaactatcatcttgagtccaacccggaaagacacgacaaaacgccactggcagcagccattggtaactgagaatt
gggcaagtcgggctgacgacgcggaataggccattgatagtagaactcaggttgggcctttctgtgctgttttgcggtgaccgtcgtcggtaaccattgactcttaa
ColA ori

agtggatttagatatcgagagtcttgaagtggtggcctaacagaggctacactgaaaggacagtatttggtatctgcgctccactaaagccagttaccaggttaagc
tcacctaaatctatagctctcagaacttcaccaccggattgtctccgatgtgactttcctgtcataaaccatagacgcgaggtgatttcggtcaatggtccaattcg
ColA ori

agttccccaactgacttaaccttcgatcaaaccgcctccccaggcggtttttttcgtttacagagcaggagattacgacgatcgtaaaaggatctcaagaagatcctt
tcaaggggttgactgaattggaagctagtttggcggaggggtccgccaaaaaaagcaaatgtctcgtcctctaatgctgctagcattttcctagagttcttctaggaa
ColA ori

tacggattcccgacaccatcactctagatttcagtgcaatttatctcttcaaatgtagcacctgaagtcagccccatacgatataagttgtaattctcatgttagtc
atgcctaagggctgtggtagtgagatctaaagtcacgttaaatagagaagtttacatcgtggacttcagtcggggtatgctatattcaacattaagagtacaatcag
ColA ori

atgccccgcgcccaccggaaggagctgactgggttgCTCCTAgGGTCTGATTCGTTACCAATTATGACAACTTGACGGCTACATCATTCACTTTTTCTTCACAACCG
tacggggcgcgggtggccttcctcgactgacccaacGAGGATcCCAGACTAAGCAATGGTTAATACTGTTGAACTGCCGATGTAGTAAGTGAAAAAGAAGTGTTGGC
araC

GCACGGAACTCGCTCGGGCTGGCCCCGGTGCATTTTTTAAATACCCGCGAGAAATAGAGTTGATCGTCAAAACCAACATTGCGACCGACGGTGGCGATAGGCATCCG
CGTGCCTTGAGCGAGCCCGACCGGGGCCACGTAAAAAATTTATGGGCGCTCTTTATCTCAACTAGCAGTTTTGGTTGTAACGCTGGCTGCCACCGCTATCCGTAGGC
araC

GGTGGTGCTCAAAAGCAGCTTCGCCTGGCTGATACGTTGGTCCTCGCGCCAGCTTAAGACGCTAATCCCTAACTGCTGGCGGAAAAGATGTGACAGACGCGACGGCG
CCACCACGAGTTTTCGTCGAAGCGGACCGACTATGCAACCAGGAGCGCGGTCGAATTCTGCGATTAGGGATTGACGACCGCCTTTTCTACACTGTCTGCGCTGCCGC
araC

ACAAGCAAACATGCTGTGCGACGCTGGCGATATCAAAATTGCTGTCTGCCAGGTGATCGCTGATGTACTGACAAGCCTCGCGTACCCGATTATCCATCGGTGGATGG
TGTTCGTTTGTACGACACGCTGCGACCGCTATAGTTTTAACGACAGACGGTCCACTAGCGACTACATGACTGTTCGGAGCGCATGGGCTAATAGGTAGCCACCTACC
araC

pC-0 (4150 bp) (from 3639-4150 bp)

AGCGACTCGTTAATCGCTTCCATGCGCCGCAGTAACAATTGCTCAAGCAGATTTATCGCCAGCAGCTCCGAATAGCGCCCTTCCCCTTGCCCGGCGTTAATGATTTG
TCGCTGAGCAATTAGCGAAGGTACGCGGCGTCATTGTTAACGAGTTCGTCTAAATAGCGGTCGTCGAGGCTTATCGCGGGAAGGGGAACGGGCCGCAATTACTAAAC

araC

CCCAAACAGGTCGCTGAAATGCGGCTGGTGCGCTTCATCCGGGCGAAAGAACCCCGTATTGGCAAATATTGACGGCCAGTTAAGCCATTCATGCCAGTAGGCGCGCG
GGGTTTGTCCAGCGACTTTACGCCGACCACGCGAAGTAGGCCCGCTTTCTTGGGGCATAACCGTTTATAACTGCCGGTCAATTCGGTAAGTACGGTCATCCGCGCGC

araC

GACGAAAGTAAACCCACTGGTGATACCATTCGCGAGCCTCCGGATGACGACCGTAGTGATGAATCTCTCCTGGCGGGAACAGCAAAATATCACCCGGTCGGCAAACA
CTGCTTTCATTTGGGTGACCACTATGGTAAGCGCTCGGAGGCCTACTGCTGGCATCACTACTTAGAGAGGACCGCCCTTGTCGTTTTATAGTGGGCCAGCCGTTTGT

araC

AATTCTCGTCCCTGATTTTTCACCACCCCCTGACCGCGAATGGTGAGATTGAGAATATAACCTTTCATTCCCAGCGGTCGGTCGATAAAAAAATCGAGATAACCGTT
TTAAGAGCAGGGACTAAAAAGTGGTGGGGGACTGGCGCTTACCACTCTAACTCTTATATTGGAAAGTAAGGGTCGCCAGCCAGCTATTTTTTTAGCTCTATTGGCAA

araC

GGCCTCAATCGGCGTTAAACCCGCCACCAGATGGGCATTAAACGAGTATCCCGGCAGCAGGGGATCATTTTGCGCTTCAGCCAT
CCGGAGTTAGCCGCAATTTGGGCGGTGGTCTACCCGTAATTTGCTCATAGGGCCGTCGTCCCCTAGTAAAACGCGAAGTCGGTA

araC

(from 1-1070 bp)

pC-0_v2 (4145 bp)

ACTTTTCATACTCCCGCCATTCAGAGAAGAAACCAATTGTCCATATTGCATCAGACATTGCCGTCACTGCGTCTTTTACTGGCTCTTCTCGCTAACCAAACCGGTAA
TGAAAAGTATGAGGGCGGTAAGTCTCTTCTTTGGTTAACAGGTATAACGTAGTCTGTAACGGCAGTGACGCAGAAAATGACCGAGAAGAGCGATTGGTTTGGCCATT

CCCCGCTTATTAAAAGCATTCTGTAACAAAGCGGGACCAAAGCCATGACAAAAACGCGTAACAAAAGTGTCTATAATCACGGCAGAAAAGTCCACATTGATTATTTG
GGGGCGAATAATTTTCGTAAGACATTGTTTCGCCCTGGTTTCGGTACTGTTTTTGCGCATTGTTTTCACAGATATTAGTGCCGTCTTTTCAGGTGTAACTAATAAAC

EcoRI

CACGGCGTCACACTTTGCTATGCCATAGCATTTTTATCCATAAGATTAGCGGtTCCTACCTGACGCTTTTTATCGCAACTCTCTACTGTTTCTCCATACCGAATTCA
GTGCCGCAGTGTGAAACGATACGGTATCGTAAAAATAGGTATTCTAATCGCCaAGGATGGACTGCGAAAAATAGCGTTGAGAGATGACAAAGAGGTATGGCTTAAGT

P(BAD) promoter

SacI XhoI NcoI

TAGGATAGATTCTGGAAACTTTACCGTCCGAGCTCAGGCTTACCTTACTCGAGCAATAAACAGTTGATAGGGCTTCTCCGTTACCATGGTTCAGCCAAAAAACTTAA
ATCCTATCTAAGACCTTTGAAATGGCAGGCTCGAGTCCGAATGGAATGAGCTCGTTATTTGTCAACTATCCCGAAGAGGCAATGGTACCAAGTCGGTTTTTTGAATT

Linker_14 Spa... 8 Linker_0 ECK120...nator

GACCGCCGGTCTTGTCCACTACCTTGCAGTAATGCGGTGGACAGGATCGGCGGTTTTCTTTTCTCTTCTCAATTCTTCTGACCTGTAACGAATAATAGATAGTAAAG
CTGGCGGCCAGAACAGGTGATGGAACGTCATTACGCCACCTGTCCTAGCCGCCAAAAGAAAAGAGAAGAGTTAAGAAGACTGGACATTGCTTATTATCTATCATTTC

ECK120029600 Terminator Spacer 1

TAGTCTCCGATTGAGTTTTCTCTGCCGAGTCCCACCCAGTTCTGTGATTTCAGTAAGTTGGTAATTGATACACTGTTGCGAGAACTGCTGCCTGGTAGTAGATAGGT
ATCAGAGGCTAACTCAAAAGAGACGGCTCAGGGTGGGTCAAGACACTAAAGTCATTCAACCATTAACTATGTGACAACGCTCTTGACGACGGACCATCATCTATCCA

Spacer 1

HindIII BamHI

TGTTATTGAGTAAGAAGGTAAAGTGAACGAAATCCCTGAAACTGAGACTGTAGAAAATAAGCTTGTCCAGACTATTGGATCCAAGAGATTTCTACACGATTGAGCAC
ACAATAACTCATTCTTCCATTTCACTTGCTTTAGGGACTTTGACTCTGACATCTTTTATTCGAACAGGTCTGATAACCTAGGTTCTCTAAAGATGTGCTAACTCGTG

Spacer 1 Spa... 7 Linker_10

SbfI KpnI

TGTCTCCTGCAGGCTCGGTACCAAATTCCAGAAAAGAGGCCTCCCGAAAGGGGGGCCTTTTTTCGTTTTGGTCCTAATAGATAAAGGATAGGTCTGGTAGTGTTGTT
ACAGAGGACGTCCGAGCCATGGTTTAAGGTCTTTTCTCCGGAGGGCTTTCCCCCCGGAAAAAAGCAAAACCAGGATTATCTATTTCCTATCCAGACCATCACAACAA

L3S2P21 Terminator Spacer 2.5

CGTTCTCGCAGGTAAATCAATAATACTCAGCAGTTCCGTAGACTTTTCAGTGGGACAGGGTAGCGATAACAGATAGATTGTAATAAGACACAGTAGGTGCTCGTAGT
GCAAGAGCGTCCATTTAGTTATTATGAGTCGTCAAGGCATCTGAAAAGTCACCCTGTCCCATCGCTATTGTCTATCTAACATTATTCTGTGTCATCCACGAGCATCA

Spacer 2.5

SalI AatII

TGCGTGAAGAGAACCGCTCAGGAAATCCAGTCAGAAGTATTGGTAATCGTTGAAAACTCAGTCGACGCACTTACTGAAGACGTCCTATTACACTCGTCGTTGGAAAC
ACGCACTTCTCTTGGCGAGTCCTTTAGGTCAGTCTTCATAACCATTAGCAACTTTTGAGTCAGCTGCGTGAATGACTTCTGCAGGATAATGTGAGCAGCAACCTTTG

Spacer 2.5 Spa... 5 Linker_11

pC-0_v2 (4145 bp) (from 1071-2354 bp)

NotI

TGAAGATGCGGCCGCggaaacacagAAAAAAGCCCGCACCTGACAGTGCGGGCTTTTTTTTTcgaccaaaggTAGCGAACGACGAGTCACTGTTGAGGATAAATACT
ACTTCTACGCCGGCGcctttgtgtcTTTTTTCGGGCGTGGACTGTCACGCCCGAAAAAAAAAgctggtttccATCGCTTGCTGCTCAGTGACAACTCCTATTTATGA

ECK120033737 Terminator — Linker 8

KasI — BbvCI — AscI

TTCTCTACTAGGCGCCTGTTACACAGGTCCTCAGCGGCGCGCCTTTGTCGGTGAACGCTCTCCTGAGTAGGACAAATCCGCCGGGAGCGGATTTGAACGTTGTGAAG
AAGAGATGATCCGCGGACAATGTGTCCAGGAGTCGCCGCGCGGAAACAGCCACTTGCGAGAGGACTCATCCTGTTTAGGCGGCCCTCGCCTAAACTTGCAACACTTC

Li...8 — Spa...15

CAACGGCCCGGAGGGTGGCGGGCAGGACGCCCGCCATAAACTGCCAGGCATCAAACTAAGCAGAAGGCCATCCTGACGGATGGCCTTTTTGCGTTTCAGATCTggga
GTTGCCGGGCCTCCCACCGCCCGTCCTGCGGGCGGTATTTGACGGTCCGTAGTTTGATTCGTCTTCCGGTAGGACTGCCTACCGGAAAAACGCAAAGTCTAGAccct

ttttggtcatgagattatcaaaaaggatcttcacctagatccttttaaattaaaaatgaagttttaaatcaatctaaagtatatatgagtaaacttggtctgacagt
aaaaccagtactctaatagtttttcctagaagtggatctaggaaaatttaatttttacttcaaaatttagttagatttcatatatactcatttgaaccagactgtca

taccaatgcttaatcagtgaggcacctatctcagcgatctgtctatttcgttcatccatagttgcctgactccccgtcgtgtagataactacgatacgggagggctt
atggttacgaattagtcactccgtggatagagtcgctagacagataaagcaagtaggtatcaacggactgaggggcagcacatctattgatgctatgccctcccgaa

AmpR

accatctggccccagtgctgcaatgataccgcgagacccacgctcaccggctccagatttatcagcaataaaccagccagccggaagggccgagcgcagaagtggtc
tggtagaccggggtcacgacgttactatggcgctctgggtgcgagtggccgaggtctaaatagtcgttatttggtcggtcggccttcccggctcgcgtcttcaccag

AmpR

ctgcaactttatccgcctccatccagtctattaattgttgccgggaagctagagtaagtagttcgccagttaatagtttgcgcaacgttgttgccattgctgcaggc
gacgttgaaataggcggaggtaggtcagataattaacaacggcccttcgatctcattcatcaagcggtcaattatcaaacgcgttgcaacaacggtaacgacgtccg

AmpR

atcgtggtgtcacgctcgtcgtttggtatggcttcattcagctccggttcccaacgatcaaggcgagttacatgatcccccatgttgtgcaaaaaagcggttagctc
tagcaccacagtgcgagcagcaaaccataccgaagtaagtcgaggccaagggttgctagttccgctcaatgtactagggggtacaacacgttttttcgccaatcgag

AmpR

cttcggtcctccgatcgttgtcagaagtaagttggccgcagtgttatcactcatggttatggcagcactgcataattctcttactgtcatgccatccgtaagatgct
gaagccaggaggctagcaacagtcttcattcaaccggcgtcacaatagtgagtaccaataccgtcgtgacgtattaagagaatgacagtacggtaggcattctacga

AmpR

tttctgtgactggtgagtactcaaccaagtcattctgagaatagtgtatgcggcgaccgagttgctcttgcccggcgtcaacacgggataataccgcgccacatagc
aaagacactgaccactcatgagttggttcagtaagactcttatcacatacgccgctggctcaacgagaacgggccgcagttgtgccctattatggcgcggtgtatcg

AmpR

agaactttaaaagtgctcatcattggaaaacgttcttcggggcgaaaactctcaaggatcttaccgctgttgagatccagttcgatgtaacccactcgtgcacccaa
tcttgaaattttcacgagtagtaaccttttgcaagaagccccgcttttgagagttcctagaatggcgacaactctaggtcaagctacattgggtgagcacgtgggtt

AmpR

ctgatcttcagcatcttttactttcaccagcgtttctgggtgagcaaaaacaggaaggcaaaatgccgcaaaaaagggaataagggcgacacggaaatgttgaatac
gactagaagtcgtagaaaatgaaagtggtcgcaaagacccactcgtttttgtccttccgttttacggcgttttttcccttattcccgctgtgcctttacaacttatg

AmpR

pC-0_v2 (4145 bp) (from 2355-3638 bp)

tcatactcttcctttttcaatattattgaagcatttatcagggttattgtctcatgagcggatacatatttgaatgtatttagaaaaataaacaaataggggttccg
agtatgagaaggaaaaagttataataacttcgtaaatagtcccaataacagagtactcgcctatgtataaacttacataaatctttttatttgtttatccccaaggc

cgcagcatgctagcGgcagaaacgtcctagaagatgccaggaggatacttagcagagagacaataaggccggagcgaagccgtttttccataggctccgcccccctg
gcgtcgtacgatcgCcgtctttgcaggatcttctacggtcctcctatgaatcgtctctctgttattccggcctcgcttcggcaaaaaggtatccgaggcggggggac
ColA ori

acgaacatcacgaaatctgacgctcaaatcagtggtggcgaaacccgacaggactataaagataccaggcgtttccccctgatggctccctcttgcgctctcctgtt
tgcttgtagtgctttagactgcgagtttagtcaccaccgctttgggctgtcctgatatttctatggtccgcaaaggggggactaccgagggagaacgcgagaggacaa
ColA ori

cccgtcctgcggcgtccgtgttgtggtggaggctttacccaaatcaccacgtcccgttccgtgtagacagttcgctccaagctgggctgtgtgcaagaaccccccgt
gggcaggacgccgcaggcacaacaccacctccgaaatgggtttagtggtgcagggcaaggcacatctgtcaagcgaggttcgacccgacacacgttcttggggggca
ColA ori

tcagcccgactgctgcgccttatccggtaactatcatcttgagtccaacccggaaagacacgacaaaacgccactggcagcagccattggtaactgagaattagtgg
agtcgggctgacgacgcggaataggccattgatagtagaactcaggttgggcctttctgtgctgttttgcggtgaccgtcgtcggtaaccattgactcttaatcacc
ColA ori

atttagatatcgagagtcttgaagtggtggcctaacagaggctacactgaaaggacagtatttggtatctgcgctccactaaagccagttaccaggttaagcagttc
taaatctatagctctcagaacttcaccaccggattgtctccgatgtgactttcctgtcataaaccatagacgcgaggtgatttcggtcaatggtccaattcgtcaag
ColA ori

cccaactgacttaaccttcgatcaaaccgcctccccaggcggttttttcgtttacagagcaggagattacgacgatcgtaaaaggatctcaagaagatcctttacgg
gggttgactgaattggaagctagtttggcggaggggtccgccaaaaaagcaaatgtctcgtcctctaatgctgctagcattttcctagagttcttctaggaaatgcc
ColA ori

attcccgacaccatcactctagatttcagtgcaatttatctcttcaaatgtagcacctgaagtcagccccatacgatataagttgtaattctcatgttagtcatgcc
taagggctgtggtagtgagatctaaagtcacgttaaatagagaagtttacatcgtggacttcagtcggggtatgctatattcaacattaagagtacaatcagtacgg
ColA ori

ccgcgcccaccggaaggagctgactgggttgCTCCTAgGGTCTGATTCGTTACCAATTATGACAACTTGACGGCTACATCATTCACTTTTTCTTCACAACCGGCACG
ggcgcgggtggccttcctcgactgacccaacGAGGATcCCAGACTAAGCAATGGTTAATACTGTTGAACTGCCGATGTAGTAAGTGAAAAAGAAGTGTTGGCCGTGC
araC

GAACTCGCTCGGGCTGGCCCCGGTGCATTTTTTAAATACCCGCGAGAAATAGAGTTGATCGTCAAAACCAACATTGCGACCGACGGTGGCGATAGGCATCCGGGTGG
CTTGAGCGAGCCCGACCGGGGCCACGTAAAAAATTTATGGGCGCTCTTTATCTCAACTAGCAGTTTTGGTTGTAACGCTGGCTGCCACCGCTATCCGTAGGCCCACC
araC

TGCTCAAAAGCAGCTTCGCCTGGCTGATACGTTGGTCCTCGCGCCAGCTTAAGACGCTAATCCCTAACTGCTGGCGGAAAAGATGTGACAGACGCGACGGCGACAAG
ACGAGTTTTCGTCGAAGCGGACCGACTATGCAACCAGGAGCGCGGTCGAATTCTGCGATTAGGGATTGACGACCGCCTTTTCTACACTGTCTGCGCTGCCGCTGTTC
araC

CAAACATGCTGTGCGACGCTGGCGATATCAAAATTGCTGTCTGCCAGGTGATCGCTGATGTACTGACAAGCCTCGCGTACCCGATTATCCATCGGTGGATGGAGCGA
GTTTGTACGACACGCTGCGACCGCTATAGTTTTAACGACAGACGGTCCACTAGCGACTACATGACTGTTCGGAGCGCATGGGCTAATAGGTAGCCACCTACCTCGCT
araC

pC-0_v2 (4145 bp) (from 3639-4145 bp)

CTCGTTAATCGCTTCCATGCGCCGCAGTAACAATTGCTCAAGCAGATTTATCGCCAGCAGCTCCGAATAGCGCCCTTCCCCTTGCCCGGCGTTAATGATTTGCCCAA
GAGCAATTAGCGAAGGTACGCGGCGTCATTGTTAACGAGTTCGTCTAAATAGCGGTCGTCGAGGCTTATCGCGGGAAGGGGAACGGGCCGCAATTACTAAACGGGTT
araC

ACAGGTCGCTGAAATGCGGCTGGTGCGCTTCATCCGGGCGAAAGAACCCCGTATTGGCAAATATTGACGGCCAGTTAAGCCATTCATGCCAGTAGGCGCGCGGACGA
TGTCCAGCGACTTTACGCCGACCACGCGAAGTAGGCCCGCTTTCTTGGGGCATAACCGTTTATAACTGCCGGTCAATTCGGTAAGTACGGTCATCCGCGCGCCTGCT
araC

AAGTAAACCCACTGGTGATACCATTCGCGAGCCTCCGGATGACGACCGTAGTGATGAATCTCTCCTGGCGGGAACAGCAAAATATCACCCGGTCGGCAAACAAATTC
TTCATTTGGGTGACCACTATGGTAAGCGCTCGGAGGCCTACTGCTGGCATCACTACTTAGAGAGGACCGCCCTTGTCGTTTTATAGTGGGCCAGCCGTTTGTTTAAG
araC

TCGTCCCTGATTTTTCACCACCCCCTGACCGCGAATGGTGAGATTGAGAATATAACCTTTCATTCCCAGCGGTCGGTCGATAAAAAAATCGAGATAACCGTTGGCCT
AGCAGGGACTAAAAAGTGGTGGGGGACTGGCGCTTACCACTCTAACTCTTATATTGGAAAGTAAGGGTCGCCAGCCAGCTATTTTTTTAGCTCTATTGGCAACCGGA
araC

CAATCGGCGTTAAACCCGCCACCAGATGGGCATTAAACGAGTATCCCGGCAGCAGGGGATCATTTTGCGCTTCAGCCAT
GTTAGCCGCAATTTGGGCGGTGGTCTACCCGTAATTTGCTCATAGGGCCGTCGTCCCCTAGTAAAACGCGAAGTCGGTA
araC

(from 1-1177 bp)

pJ1996_v2 (7989 bp)

```
ttttctactgaaccgctctagatttcagtgcaatttatctcttcaaatgtagcacctgaagtcagccccatacgatataagttgtaattctcatgttagtcatgccc
aaaagatgacttggcgagatctaaagtcacgttaaatagagaagtttacatcgtggacttcagtcggggtatgctatattcaacattaagagtacaatcagtacggg

                                                XhoI                           EcoRI     SacI
cgcgcccaccggaaggagctgactgggttgCTCCTAGGATGCAGTGACTCGAGtTGTaAAACGACGGCCAgtcaattgGAATTCGAGCTCCAGCCTGCGGTCCGGtt
gcgcgggtggccttcctcgactgacccaacGAGGATCCTACGTCACTGAGCTCaACAtTTTGCTGCCGGTcagttaacCTTAAGCTCGAGGTCGGACGCCAGGCCaa
                                                                                                 Prefix

                              SpeI                                                                     KpnI
gacagctagctcagtcctaggtattgtActagTTCGCTGGGACGCCCGAGTGACGACTGCGAAGTAACCTCTATTTATCAGCCTGCGGTCCGGgtaccattaaaga
ctgtcgatcgagtcaggatccataacaTgatcAAGCGACCCTGCGGGCTCACTGCTGACGCTTCATTGGAGATAAATAGTCGGACGCCAGGCCccatggtaatttct
P(BBa_J23104-SpeI)  Suffix  Linker_25  Prefix  RBS

      EcoRI
ggagaaagaattcATGGATAAGAAATACTCAATAGGCTTAGCTATCGGCACAAATAGCGTCGGATGGGCGGTGATCACTGATGAATATAAGGTTCCGTCTAAAAAGT
cctctttcttaagTACCTATTCTTTATGAGTTATCCGAATCGATAGCCGTGTTTATCGCAGCCTACCCGCCACTAGTGACTACTTATATTCCAAGGCAGATTTTTCA
RBS  dCas9

TCAAGGTTCTGGGAAATACAGACCGCCACAGTATCAAAAAAAATCTTATAGGGGCTCTTTTATTTGACAGTGGAGAGACAGCGGAAGCGACTCGTCTCAAACGGACA
AGTTCCAAGACCCTTTATGTCTGGCGGTGTCATAGTTTTTTTTAGAATATCCCCGAGAAAATAAACTGTCACCTCTCTGTCGCCTTCGCTGAGCAGAGTTTGCCTGT
dCas9

GCTCGTAGAAGGTATACACGTCGGAAGAATCGTATTTGTTATCTACAGGAGATTTTTTCAAATGAGATGGCGAAAGTAGATGATAGTTTCTTTCATCGACTTGAAGA
CGAGCATCTTCCATATGTGCAGCCTTCTTAGCATAAACAATAGATGTCCTCTAAAAAAGTTTACTCTACCGCTTTCATCTACTATCAAAGAAAGTAGCTGAACTTCT
dCas9

GTCTTTTTTGGTGGAAGAAGACAAGAAGCATGAACGTCATCCTATTTTTGGAAATATAGTAGATGAAGTTGCTTATCATGAGAAATATCCAACTATCTATCATCTGC
CAGAAAAAACCACCTTCTTCTGTTCTTCGTACTTGCAGTAGGATAAAAACCTTTATATCATCTACTTCAACGAATAGTACTCTTTATAGGTTGATAGATAGTAGACG
dCas9

GAAAAAAATTGGTAGATTCTACTGATAAAGCGGATTTGCGCTTAATCTATTTGGCCTTAGCGCATATGATTAAGTTTCGTGGTCATTTTTTGATTGAGGGAGATTTA
CTTTTTTTAACCATCTAAGATGACTATTTCGCCTAAACGCGAATTAGATAAACCGGAATCGCGTATACTAATTCAAAGCACCAGTAAAAAACTAACTCCCTCTAAAT
dCas9

AATCCTGATAATAGTGATGTGGACAAACTATTTATCCAGTTGGTACAAACCTACAATCAATTATTTGAAGAAAACCCTATTAACGCAAGTGGAGTAGATGCTAAAGC
TTAGGACTATTATCACTACACCTGTTTGATAAATAGGTCAACCATGTTTGGATGTTAGTTAATAAACTTCTTTTGGGATAATTGCGTTCACCTCATCTACGATTTCG
dCas9

GATTCTTTCTGCACGATTGAGTAAATCAAGACGATTAGAAAATCTCATTGCTCAGCTCCCCGGTGAGAAGAAAAATGGCTTATTTGGGAATCTCATTGCTTTGTCAT
CTAAGAAAGACGTGCTAACTCATTTAGTTCTGCTAATCTTTTAGAGTAACGAGTCGAGGGGCCACTCTTCTTTTTACCGAATAAACCCTTAGAGTAACGAAACAGTA
dCas9

TGGGTTTGACCCCTAATTTTAAATCAAATTTTGATTTGGCAGAAGATGCTAAATTACAGCTTTCAAAAGATACTTACGATGATGATTTAGATAATTTATTGGCGCAA
ACCCAAACTGGGGATTAAAATTTAGTTTAAAACTAAACCGTCTTCTACGATTTAATGTCGAAAGTTTTCTATGAATGCTACTACTAAATCTATTAAATAACCGCGTT
dCas9
```

pJ1996_v2 (7989 bp) (from 1178-2354 bp)

ATTGGAGATCAATATGCTGATTTGTTTTTGGCAGCTAAGAATTTATCAGATGCTATTTTACTTTCAGATATCCTAAGAGTAAATACTGAAATAACTAAGGCTCCCCT
TAACCTCTAGTTATACGACTAAACAAAAACCGTCGATTCTTAAATAGTCTACGATAAAATGAAAGTCTATAGGATTCTCATTTATGACTTTATTGATTCCGAGGGGA
dCas9

HindIII
ATCAGCTTCAATGATTAAACGCTACGATGAACATCATCAAGACTTGACTCTTTTAAAAGCTTTAGTTCGACAACAACTTCCAGAAAAGTATAAAGAAATCTTTTTTG
TAGTCGAAGTTACTAATTTGCGATGCTACTTGTAGTAGTTCTGAACTGAGAAAATTTTCGAAATCAAGCTGTTGTTGAAGGTCTTTTCATATTTCTTTAGAAAAAAC
dCas9

ATCAATCAAAAAACGGATATGCAGGTTATATTGATGGGGGAGCTAGCCAAGAAGAATTTTATAAATTTATCAAACCAATTTTAGAAAAAATGGATGGTACTGAGGAA
TAGTTAGTTTTTTGCCTATACGTCCAATATAACTACCCCCTCGATCGGTTCTTCTTAAAATATTTAAATAGTTTGGTTAAAATCTTTTTTACCTACCATGACTCCTT
dCas9

TTATTGGTGAAACTAAATCGTGAAGATTTGCTGCGCAAGCAACGGACCTTTGACAACGGCTCTATTCCCCATCAAATTCACTTGGGTGAGCTGCATGCTATTTTGAG
AATAACCACTTTGATTTAGCACTTCTAAACGACGCGTTCGTTGCCTGGAAACTGTTGCCGAGATAAGGGGTAGTTTAAGTGAACCCACTCGACGTACGATAAAACTC
dCas9

EcoRI
AAGACAAGAAGACTTTTATCCATTTTTAAAAGACAATCGTGAGAAGATTGAAAAAATCTTGACTTTTCGAATTCCTTATTATGTTGGTCCATTGGCGCGTGGCAATA
TTCTGTTCTTCTGAAAATAGGTAAAAATTTTCTGTTAGCACTCTTCTAACTTTTTTAGAACTGAAAAGCTTAAGGAATAATACAACCAGGTAACCGCGCACCGTTAT
dCas9

NcoI
GTCGTTTTGCATGGATGACTCGGAAGTCTGAAGAAACAATTACCCCATGGAATTTTGAAGAAGTTGTCGATAAAGGTGCTTCAGCTCAATCATTTATTGAACGCATG
CAGCAAAACGTACCTACTGAGCCTTCAGACTTCTTTGTTAATGGGGTACCTTAAAACTTCTTCAACAGCTATTTCCACGAAGTCGAGTTAGTAAATAACTTGCGTAC
dCas9

ACAAACTTTGATAAAAATCTTCCAAATGAAAAAGTACTACCAAAACATAGTTTGCTTTATGAGTATTTTACGGTTTATAACGAATTGACAAAGGTCAAATATGTTAC
TGTTTGAAACTATTTTTAGAAGGTTTACTTTTTCATGATGGTTTTGTATCAAACGAAATACTCATAAAATGCCAAATATTGCTTAACTGTTTCCAGTTTATACAATG
dCas9

TGAAGGAATGCGAAAACCAGCATTTCTTTCAGGTGAACAGAAGAAAGCCATTGTTGATTTACTCTTCAAAACAAATCGAAAAGTAACCGTTAAGCAATTAAAAGAAG
ACTTCCTTACGCTTTTGGTCGTAAAGAAAGTCCACTTGTCTTCTTTCGGTAACAACTAAATGAGAAGTTTTGTTTAGCTTTTCATTGGCAATTCGTTAATTTTCTTC
dCas9

KpnI
ATTATTTCAAAAAAATAGAATGTTTTGATAGTGTTGAAATTTCAGGAGTTGAAGATAGATTTAATGCTTCATTAGGTACCTACCATGATTTGCTAAAAATTATTAAA
TAATAAAGTTTTTTTATCTTACAAAACTATCACAACTTTAAAGTCCTCAACTTCTATCTAAATTACGAAGTAATCCATGGATGGTACTAAACGATTTTTAATAATTT
dCas9

GATAAAGATTTTTTGGATAATGAAGAAAATGAAGATATCTTAGAGGATATTGTTTTAACATTGACCTTATTTGAAGATAGGGAGATGATTGAGGAAAGACTTAAAAC
CTATTTCTAAAAAACCTATTACTTCTTTTACTTCTATAGAATCTCCTATAACAAAATTGTAACTGGAATAAACTTCTATCCCTCTACTAACTCCTTTCTGAATTTTG
dCas9

ATATGCTCACCTCTTTGATGATAAGGTGATGAAACAGCTTAAACGTCGCCGTTATACTGGTTGGGGACGTTTGTCTCGAAAATTGATTAATGGTATTAGGGATAAGC
TATACGAGTGGAGAAACTACTATTCCACTACTTTGTCGAATTTGCAGCGGCAATATGACCAACCCCTGCAAACAGAGCTTTTAACTAATTACCATAATCCCTATTCG
dCas9

pJ1996_v2 (7989 bp) (from 2355-3638 bp)

AATCTGGCAAAACAATATTAGATTTTTTGAAATCAGATGGTTTTGCCAATCGCAATTTTATGCAGCTGATCCATGATGATAGTTTGACATTTAAAGAAGACATTCAA
TTAGACCGTTTTGTTATAATCTAAAAAACTTTAGTCTACCAAAACGGTTAGCGTTAAAATACGTCGACTAGGTACTACTATCAAACTGTAAATTTCTTCTGTAAGTT
dCas9

AAAGCACAAGTGTCTGGACAAGGCGATAGTTTACATGAACATATTGCAAATTTAGCTGGTAGCCCTGCTATTAAAAAAGGTATTTTACAGACTGTAAAAGTTGTTGA
TTTCGTGTTCACAGACCTGTTCCGCTATCAAATGTACTTGTATAACGTTTAAATCGACCATCGGGACGATAATTTTTTCCATAAAATGTCTGACATTTTCAACAACT
dCas9

TGAATTGGTCAAAGTAATGGGGCGGCATAAGCCAGAAAATATCGTTATTGAAATGGCACGTGAAAATCAGACAACTCAAAAGGGCCAGAAAAATTCGCGAGAGCGTA
ACTTAACCAGTTTCATTACCCCGCCGTATTCGGTCTTTTATAGCAATAACTTTACCGTGCACTTTTAGTCTGTTGAGTTTTCCCGGTCTTTTTAAGCGCTCTCGCAT
dCas9

TGAAACGAATCGAAGAAGGTATCAAAGAATTAGGAAGTCAGATTCTTAAAGAGCATCCTGTTGAAAATACTCAATTGCAAAATGAAAAGCTCTATCTCTATTATCTC
ACTTTGCTTAGCTTCTTCCATAGTTTCTTAATCCTTCAGTCTAAGAATTTCTCGTAGGACAACTTTTATGAGTTAACGTTTTACTTTTCGAGATAGAGATAATAGAG
dCas9

CAAAATGGAAGAGACATGTATGTGGACCAAGAATTAGATATTAATCGTTTAAGTGATTATGATGTCGATGCCATTGTTCCACAAAGTTTCCTTAAAGACGATTCAAT
GTTTTACCTTCTCTGTACATACACCTGGTTCTTAATCTATAATTAGCAAATTCACTAATACTACAGCTACGGTAACAAGGTGTTTCAAAGGAATTTCTGCTAAGTTA
dCas9

AGACAATAAGGTCTTAACGCGTTCTGATAAAAATCGTGGTAAATCGGATAACGTTCCAAGTGAAGAAGTAGTCAAAAAGATGAAAAACTATTGGAGACAACTTCTAA
TCTGTTATTCCAGAATTGCGCAAGACTATTTTTAGCACCATTTAGCCTATTGCAAGGTTCACTTCTTCATCAGTTTTTCTACTTTTTGATAACCTCTGTTGAAGATT
dCas9

ACGCCAAGTTAATCACTCAACGTAAGTTTGATAATTTAACGAAAGCTGAACGTGGAGGTTTGAGTGAACTTGATAAAGCTGGTTTTATCAAACGCCAATTGGTTGAA
TGCGGTTCAATTAGTGAGTTGCATTCAAACTATTAAATTGCTTTCGACTTGCACCTCCAAACTCACTTGAACTATTTCGACCAAAATAGTTTGCGGTTAACCAACTT
dCas9

ACTCGCCAAATCACTAAGCATGTGGCACAAATTTTGGATAGTCGCATGAATACTAAATACGATGAAAATGATAAACTTATTCGAGAGGTTAAAGTGATTACCTTAAA
TGAGCGGTTTAGTGATTCGTACACCGTGTTTAAAACCTATCAGCGTACTTATGATTTATGCTACTTTTACTATTTGAATAAGCTCTCCAATTTCACTAATGGAATTT
dCas9

ATCTAAATTAGTTTCTGACTTCCGAAAAGATTTCCAATTCTATAAAGTACGTGAGATTAACAATTACCATCATGCCCATGATGCGTATCTAAATGCCGTCGTTGGAA
TAGATTTAATCAAAGACTGAAGGCTTTTCTAAAGGTTAAGATATTTCATGCACTCTAATTGTTAATGGTAGTACGGGTACTACGCATAGATTTACGGCAGCAACCTT
dCas9

CTGCTTTGATTAAGAAATATCCAAAACTTGAATCGGAGTTTGTCTATGGTGATTATAAAGTTTATGATGTTCGTAAAATGATTGCTAAGTCTGAGCAAGAAATAGGC
GACGAAACTAATTCTTTATAGGTTTTGAACTTAGCCTCAAACAGATACCACTAATATTTCAAATACTACAAGCATTTTACTAACGATTCAGACTCGTTCTTTATCCG
dCas9

AAAGCAACCGCAAAATATTTCTTTTACTCTAATATCATGAACTTCTTCAAAACAGAAATTACACTTGCAAATGGAGAGATTCGCAAACGCCCTCTAATCGAAACTAA
TTTCGTTGGCGTTTTATAAAGAAAATGAGATTATAGTACTTGAAGAAGTTTTGTCTTTAATGTGAACGTTTACCTCTCTAAGCGTTTGCGGGAGATTAGCTTTGATT
dCas9

TGGGGAAACTGGAGAAATTGTCTGGGATAAAGGGCGAGATTTTGCCACAGTGCGCAAAGTATTGTCCATGCCCCAAGTCAATATTGTCAAGAAAACAGAAGTACAGA
ACCCCTTTGACCTCTTTAACAGACCCTATTTCCCGCTCTAAAACGGTGTCACGCGTTTCATAACAGGTACGGGGTTCAGTTATAACAGTTCTTTTGTCTTCATGTCT
dCas9

pJ1996_v2 (7989 bp) (from 3639-4815 bp)

HindIII BamHI

CAGGCGGATTCTCCAAGGAGTCAATTTTACCAAAAAGAAATTCGGACAAGCTTATTGCTCGTAAAAAAGACTGGGATCCAAAAAAATATGGTGGTTTTGATAGTCCA
GTCCGCCTAAGAGGTTCCTCAGTTAAAATGGTTTTTCTTTAAGCCTGTTCGAATAACGAGCATTTTTTCTGACCCTAGGTTTTTTTATACCACCAAAACTATCAGGT

dCas9

ACGGTAGCTTATTCAGTCCTAGTGGTTGCTAAGGTGGAAAAAGGGAAATCGAAGAAGTTAAAATCCGTTAAAGAGTTACTAGGGATCACAATTATGGAAAGAAGTTC
TGCCATCGAATAAGTCAGGATCACCAACGATTCCACCTTTTTCCCTTTAGCTTCTTCAATTTTAGGCAATTTCTCAATGATCCCTAGTGTTAATACCTTTCTTCAAG

dCas9

CTTTGAAAAAAATCCGATTGACTTTTTAGAAGCTAAAGGATATAAGGAAGTTAAAAAAGACTTAATCATTAAACTACCTAAATATAGTCTTTTTGAGTTAGAAAACG
GAAACTTTTTTTAGGCTAACTGAAAAATCTTCGATTTCCTATATTCCTTCAATTTTTTCTGAATTAGTAATTTGATGGATTTATATCAGAAAAACTCAATCTTTTGC

dCas9

GTCGTAAACGGATGCTGGCTAGTGCCGGAGAATTACAAAAAGGAAATGAGCTGGCTCTGCCAAGCAAATATGTGAATTTTTTATATTTAGCTAGTCATTATGAAAAG
CAGCATTTGCCTACGACCGATCACGGCCTCTTAATGTTTTTCCTTTACTCGACCGAGACGGTTCGTTTATACACTTAAAAAATATAAATCGATCAGTAATACTTTTC

dCas9

TTGAAGGGTAGTCCAGAAGATAACGAACAAAAACAATTGTTTGTGGAGCAGCATAAGCATTATTTAGATGAGATTATTGAGCAAATCAGTGAATTTTCTAAGCGTGT
AACTTCCCATCAGGTCTTCTATTGCTTGTTTTTGTTAACAAACACCTCGTCGTATTCGTAATAAATCTACTCTAATAACTCGTTTAGTCACTTAAAAGATTCGCACA

dCas9

TATTTTAGCAGATGCCAATTTAGATAAAGTTCTTAGTGCATATAACAAACATAGAGACAAACCAATACGTGAACAAGCAGAAAATATTATTCATTTATTTACGTTGA
ATAAAATCGTCTACGGTTAAATCTATTTCAAGAATCACGTATATTGTTTGTATCTCTGTTTGGTTATGCACTTGTTCGTCTTTTATAATAAGTAAATAAATGCAACT

dCas9

SacI

CGAATCTTGGAGCTCCCGCTGCTTTTAAATATTTTGATACAACAATTGATCGTAAACGATATACGTCTACAAAAGAAGTTTTAGATGCCACTCTTATCCATCAATCC
GCTTAGAACCTCGAGGGCGACGAAAATTTATAAAACTATGTTGTTAACTAGCATTTGCTATATGCAGATGTTTTCTTCAAAATCTACGGTGAGAATAGGTAGTTAGG

dCas9

NcoI

ATCACTGGTCTTTATGAAACACGCATTGATTTGAGTCAGCTAGGAGGTGACTAATCGCTGGGACGCCCGCCATGGTTCAGCCAAAAAACTTAAGACCGCCGGTCTTG
TAGTGACCAGAAATACTTTGTGCGTAACTAAACTCAGTCGATCCTCCACTGATTAGCGACCCTGCGGGCGGTACCAAGTCGGTTTTTTGAATTCTGGCGGCCAGAAC

dCas9 Suffix ECK120029600 Terminator

TCCACTACCTTGCAGTAATGCGGTGGACAGGATCGGCGGTTTTCTTTTCTCTTCTCAATTCTTCTGACCTGTAACGAATAATAGATAGTAAAGTAGTCTCCGATTGA
AGGTGATGGAACGTCATTACGCCACCTGTCCTAGCCGCCAAAAGAAAAGAGAAGAGTTAAGAAGACTGGACATTGCTTATTATCTATCATTTCATCAGAGGCTAACT

ECK120029600 Terminator Spacer 1

GTTTTCTCTGCCGAGTCCCACCCAGTTCTGTGATTTCAGTAAGTTGGTAATTGATACACTGTTGCGAGAACTGCTGCCTGGTAGTAGATAGGTTGTTATTGAGTAAG
CAAAAGAGACGGCTCAGGGTGGGTCAAGACACTAAAGTCATTCAACCATTAACTATGTGACAACGCTCTTGACGACGGACCATCATCTATCCAACAATAACTCATTC

Spacer 1

HindIII SbfI KpnI

AAGGTAAAGTGAACGAAATCCCTGAAACTGAGACTGTAGAAAATAAGCTTGTCCAGACTATTCCTGCAGGCTCGGTACCAAATTCCAGAAAAGAGGCCTCCCGAAAG
TTCCATTTCACTTGCTTTAGGGACTTTGACTCTGACATCTTTTATTCGAACAGGTCTGATAAGGACGTCCGAGCCATGGTTTAAGGTCTTTTCTCCGGAGGGCTTTC

Spacer 1 Spa... 7 L3S2P21 Terminator

pJ1996_v2 (7989 bp) (from 4816-5885 bp)

GGGGGCCTTTTTTCGTTTTGGTCCTACTGATTGGGTGAACTACGATACTTCCAGAATAACGAGAAATACGCTACTCTTACAGATAAAACTCACTGCCGATTACGGAT
CCCCCGGAAAAAAGCAAAACCAGGATGACTAACCCACTTGATGCTATGAAGGTCTTATTGCTCTTTATGCGATGAGAATGTCTATTTTGAGTGACGGCTAATGCCTA
L3S2P21 Terminator | Spacer 2

AGGACGAACAACACTTTGATTATCTTACGATTTACCGAAGGTTCTATTGAGTTGGACCCAGCAGTTACTACTTTTATTCAGTGATTCTGTCTTATCGGGAGATTGTT
TCCTGCTTGTTGTGAAACTAATAGAATGCTAAATGGCTTCCAAGATAACTCAACCTGGGTCGTCAATGATGAAAATAAGTCACTAAGACAGAATAGCCCTCTAACAA
Spacer 2

SalI
CGCTTACCACGTCGACCAGCCTGCGGTCCGGTTTACGGCTAGCTCAGTCCTAGGTATTATGCTAGCTCGCTGGGACGCCCGAGATAGCCGTTACACAGGTGACACTT
GCGAATGGTGCAGCTGGTCGGACGCCAGGCCAAATGCCGATCGAGTCAGGATCCATAATACGATCGAGCGACCCTGCGGGCTCTATCGGCAATGTGTCCACTGTGAA
Sp...2 | Prefix | P(BBa_J23150) | Suffix | Linker_24

KpnI EcoRI
ATTTCAGCCTGCGGTCCGGggtaccattaaagaggagaaagaattcATGGACCACTACCTCGACATTCGCTTGCGACCGGACCCGGAATTTCCCCCGGCGCAACTCA
TAAAGTCGGACGCCAGGCCccatggtaatttctcctctttcttaagTACCTGGTGATGGAGCTGTAAGCGAACGCTGGCCTGGGCCTTAAAGGGGGCCGCGTTGAGT
Prefix | RBS | Csy4

TGAGCGTGCTCTTCGGCAAGCTCCACCAGGCCCTGGTGGCACAGGGCGGGGACAGGATCGGCGTGAGCTTCCCCGACCTCGACGAAAGCCGCTCCCGGCTGGGCGAG
ACTCGCACGAGAAGCCGTTCGAGGTGGTCCGGGACCACCGTGTCCCGCCCCTGTCCTAGCCGCACTCGAAGGGGCTGGAGCTGCTTTCGGCGAGGGCCGACCCGCTC
Csy4

CGCCTGCGCATTCATGCCTCGGCGGACGACCTTCGTGCCCTGCTCGCCCGGCCCTGGCTGGAAGGGTTGCGGGACCATCTGCAATTCGGAGAACCGGCAGTCGTGCC
GCGGACGCGTAAGTACGGAGCCGCCTGCTGGAAGCACGGGACGAGCGGGCCGGGACCGACCTTCCCAACGCCCTGGTAGACGTTAAGCCTCTTGGCCGTCAGCACGG
Csy4

TCACCCCACACCGTACCGTCAGGTCAGTCGGGTTCAGGCGAAAAGCAATCCGGAACGCCTGCGGCGGCGGCTCATGCGCCGGCACGATCTGAGTGAGGAGGAGGCTC
AGTGGGGTGTGGCATGGCAGTCCAGTCAGCCCAAGTCCGCTTTTCGTTAGGCCTTGCGGACGCCGCCGCCGAGTACGCGGCCGTGCTAGACTCACTCCTCCTCCGAG
Csy4

GGAAACGCATTCCCGATACGGTCGCGAGAGCCTTGGACCTGCCCTTCGTCACGCTACGCAGCCAGAGCACCGGACAGCACTTCCGTCTCTTCATCCGCCACGGGCCG
CCTTTGCGTAAGGGCTATGCCAGCGCTCTCGGAACCTGGACGGGAAGCAGTGCGATGCGTCGGTCTCGTGGCCTGTCGTGAAGGCAGAGAAGTAGGCGGTGCCCGGC
Csy4

NotI
TTGCAGGTGACGGCAGAGGAAGGAGGATTCACCTGTTACGGGTTGAGCAAAGGAGGTTTCGTTCCCTGGTTCTGAtaaTCGCTGGGACGCCCGGCGGCCGCggaaac
AACGTCCACTGCCGTCTCCTTCCTCCTAAGTGGACAATGCCCAACTCGTTTCCTCCAAAGCAAGGGACCAAGACTattAGCGACCCTGCGGGCCGCCGGCGcctttg
Csy4 | Suffix

KasI
acagAAAAAAGCCCGCACCTGACAGTGCGGGCTTTTTTTTTcgaccaaaggTAGCGAACGACGAGTCACTGTTGAGGATAAATACTTTCTCTACTAGGCGCCTGTTA
tgtcTTTTTTCGGGCGTGGACTGTCACGCCCGAAAAAAAAAgctggtttccATCGCTTGCTGCTCAGTGACAACTCCTATTTATGAAAGAGATGATCCGCGGACAAT
ECK120033737 Terminator | Linker_8

pJ1996_v2 (7989 bp) (from 5886-7276 bp)

BbvCI AscI

CACAGGTCCTCAGCGGCGCGCCTGCTGCCACCGCTGAGCAATAACTAGCATAACCCCTTGGGGCCTCTAAACGGGTCTTGAGGGGTTTTTTGCCAGGCATCAAATAA
GTGTCCAGGAGTCGCCGCGCGGACGACGGTGGCGACTCGTTATTGATCGTATTGGGGAACCCCGGAGATTTGCCCAGAACTCCCCAAAAAACGGTCCGTAGTTTATT

AACGAAAGGCTCAGTCGGAAGACTGGGCCTTTCGTTTTATCTGTTGTTTGTCGGTGAACGCTCTCCTGAGTAGGACAAATCCGCCGGGAGCGGATTTGAACGTTGTG
TTGCTTTCCGAGTCAGCCTTCTGACCCGGAAAGCAAAATAGACAACAAACAGCCACTTGCGAGAGGACTCATCCTGTTTAGGCGGCCCTCGCCTAAACTTGCAACAC

AAGCAACGGCCCGGAGGGTGGCGGGCAGGACGCCCGCCATAAACTGCCAGGCATCAAACTAAGCAGAAGGCCATCCTGACGGATGGCCTTTTTGCGTTTCAGATCTa
TTCGTTGCCGGGCCTCCCACCGCCCGTCCTGCGGGCGGTATTTGACGGTCCGTAGTTTGATTCGTCTTCCGGTAGGACTGCCTACCGGAAAAACGCAAAGTCTAGAt

ccggtaaaccagcaatagacataagcggctatttaacgaccctgccctgaaccgacgaccgggtcatcgtggccggatcttgcggcccctcggcttgaacgaattgt
ggccatttggtcgttatctgtattcgccgataaattgctgggacgggacttggctgctggcccagtagcaccggcctagaacgccggggagccgaacttgcttaaca

tagacattatttgccgactaccttggtgatctcgcctttcacgtagtggacaaattcttccaactgatctgcgcgcgaggccaagcgatcttcttcttgtccaagat
atctgtaataaacggctgatggaaccactagagcggaaagtgcatcacctgtttaagaaggttgactagacgcgcgctccggttcgctagaagaagaacaggttcta

SpecR

aagcctgtctagcttcaagtatgacgggctgatactgggccggcaggcgctccattgcccagtcggcagcgacatccttcggcgcgattttgccggttactgcgctg
ttcggacagatcgaagttcatactgcccgactatgacccggccgtccgcgaggtaacgggtcagccgtcgctgtaggaagccgcgctaaaacggccaatgacgcgac

SpecR

taccaaatgcgggacaacgtaagcactacatttcgctcatcgccagcccagtcgggcggcgagttccatagcgttaaggtttcatttagcgcctcaaatagatcctg
atggtttacgccctgttgcattcgtgatgtaaagcgagtagcggtcgggtcagcccgccgctcaaggtatcgcaattccaaagtaaatcgcggagtttatctaggac

SpecR

ttcaggaaccggatcaaagagttcctccgccgctggacctaccaaggcaacgctatgttctcttgcttttgtcagcaagatagccagatcaatgtcgatcgtggctg
aagtccttggcctagtttctcaaggaggcggcgacctggatggttccgttgcgatacaagagaacgaaaacagtcgttctatcggtctagttacagctagcaccgac

SpecR

gctcgaagatacctgcaagaatgtcattgcgctgccattctccaaattgcagttcgcgcttagctggataacgccacggaatgatgtcgtcgtgcacaacaatggtg
cgagcttctatggacgttcttacagtaacgcgacggtaagaggtttaacgtcaagcgcgaatcgacctattgcggtgccttactacagcagcacgtgttgttaccac

SpecR

acttctacagcgcggagaatctcgctctctccaggggaagccgaagtttccaaaaggtcgttgatcaaagctcgccgcgttgtttcatcaagccttacggtcaccgt
tgaagatgtcgcgcctcttagagcgagagaggtccccttcggcttcaaaggttttccagcaactagtttcgagcggcgcaacaaagtagttcggaatgccagtggca

SpecR

aaccagcaaatcaatatcactgtgtggcttcaggccgccatccactgcggagccgtacaaatgtacggccagcaacgtcggttcgagatggcgctcgatgacgccaa
ttggtcgtttagttatagtgacacaccgaagtccggcggtaggtgacgcctcggcatgtttacatgccggtcgttgcagccaagctctaccgcgagctactgcggtt

SpecR

ctacctctgatagttgagtcgatacttcggcgatcaccgcttccctcatactcttcctttttcaatattattgaagcatttatcagggttattgtctcatgagcgga
gatggagactatcaactcagctatgaagccgctagtggcgaagggagtatgagaaggaaaaagttataataacttcgtaaatagtcccaataacagagtactcgcct

SpecR

tacatatttgaatgtatttagaaaaataaacaaatagctagctcactcggtcgctacgctccgggcgtgagactgcggcgggcgctgcggacacatacaaagttacc
atgtataaacttacataaatctttttatttgtttatcgatcgagtgagccagcgatgcgaggcccgcactctgacgccgcccgcgacgcctgtgtatgtttcaatgg

CDF ori

cacagattccgtggataagcaggggactaacatgtgaggcaaaacagcagggccgcgccggtggcgtttttccataggctccgccctcctgccagagttcacataaa
gtgtctaaggcacctattcgtcccctgattgtacactccgttttgtcgtcccggcgcggccaccgcaaaaaggtatccgaggcgggaggacggtctcaagtgtattt
CDF ori

cagacgcttttccggtgcatctgtgggagccgtgaggctcaaccatgaatctgacagtacgggcgaaacccgacaggacttaaagatccccaccgtttccggcgggt
gtctgcgaaaaggccacgtagacaccctcggcactccgagttggtacttagactgtcatgcccgctttgggctgtcctgaatttctaggggtggcaaaggccgccca
CDF ori

cgctccctcttgcgctctcctgttccgaccctgccgtttaccggatacctgttccgcctttctcccttacgggaagtgtggcgctttctcatagctcacacactggt
gcgagggagaacgcgagaggacaaggctgggacggcaaatggcctatggacaaggcggaaagagggaatgcccttcacaccgcgaaagagtatcgagtgtgtgacca
CDF ori

atctcggctcggtgtaggtcgttcgctccaagctgggctgtaagcaagaactccccgttcagcccgactgctgcgccttatccggtaactgttcacttgagtccaac
tagagccgagccacatccagcaagcgaggttcgacccgacattcgttcttgaggggcaagtcgggctgacgacgcggaataggccattgacaagtgaactcaggttg
CDF ori

ccggaaaagcacggtaaaacgccactggcagcagccattggtaactgggagttcgcagaggatttgtttagctaaacacgcggttgctcttgaagtgtgcgccaaag
ggccttttcgtgccattttgcggtgaccgtcgtcggtaaccattgaccctcaagcgtctcctaaacaaatcgatttgtgcgccaacgagaacttcacacgcggtttc
CDF ori

tccggctacactggaaggacagatttggttgctgtgctctgcgaaagccagttaccacggttaagcagttccccaactgacttaaccttcgatcaaaccacctcccc
aggccgatgtgaccttcctgtctaaaccaacgacacgagacgctttcggtcaatggtgccaattcgtcaaggggttgactgaattggaagctagtttggtggagggg
CDF ori

aggtggtttttttcgtttacagggcaaaagattacgcgcagaaaaaaaggatctcaagaagatcctttgatc
tccaccaaaaaaagcaaatgtcccgttttctaatgcgcgtcttttttttcctagagttcttctaggaaactag
CDF ori

(from 1-1177 bp)

pJ2018 (8885 bp)

ttttctactgaaccgctctagatttcagtgcaatttatctcttcaaatgtagcacctgaagtcagccccatacgatataagttgtaattctcatgttagtcatgccc

aaaagatgacttggcgagatctaaagtcacgttaaatagagaagtttacatcgtggacttcagtcggggtatgctatattcaacattaagagtacaatcagtacggg

XhoI EcoRI SacI

cgcgcccaccggaaggagctgactgggttgCTCCTAGGATGCAGTGACTCGAGtTGTaAAACGACGGCCAgtcaattgGAATTCGAGCTCCAGCCTGCGGTCCGGtt

gcgcgggtggccttcctcgactgacccaacGAGGATCCTACGTCACTGAGCTCaACAtTTTGCTGCCGGTcagttaacCTTAAGCTCGAGGTCGGACGCCAGGCCaa

Prefix

SpeI KpnI

gacagctagctcagtcctaggtattgtActagTTCGCTGGGACGCCCGAGTGACGACTGCGAAGTAACCTCTATTTATCAGCCTGCGGTCCGGgtaccattaaaga

ctgtcgatcgagtcaggatccataacaTgatcAAGCGACCCTGCGGGCTCACTGCTGACGCTTCATTGGAGATAAATAGTCGGACGCCAGGCCcatggtaatttct

P(BBa_J23104-SpeI) Suffix Linker_25 Prefix RBS

EcoRI

ggagaaagaattcATGGATAAGAAATACTCAATAGGCTTAGCTATCGGCACAAATAGCGTCGGATGGGCGGTGATCACTGATGAATATAAGGTTCCGTCTAAAAAGT

cctctttcttaagTACCTATTCTTTATGAGTTATCCGAATCGATAGCCGTGTTTATCGCAGCCTACCCGCCACTAGTGACTACTTATATTCCAAGGCAGATTTTTCA

RBS dCas9

TCAAGGTTCTGGGAAATACAGACCGCCACAGTATCAAAAAAAATCTTATAGGGGCTCTTTTATTTGACAGTGGAGAGACAGCGGAAGCGACTCGTCTCAAACGGACA

AGTTCCAAGACCCTTTATGTCTGGCGGTGTCATAGTTTTTTTTAGAATATCCCCGAGAAAATAAACTGTCACCTCTCTGTCGCCTTCGCTGAGCAGAGTTTGCCTGT

dCas9

GCTCGTAGAAGGTATACACGTCGGAAGAATCGTATTTGTTATCTACAGGAGATTTTTTCAAATGAGATGGCGAAAGTAGATGATAGTTTCTTTCATCGACTTGAAGA

CGAGCATCTTCCATATGTGCAGCCTTCTTAGCATAAACAATAGATGTCCTCTAAAAAAGTTTACTCTACCGCTTTCATCTACTATCAAAGAAAGTAGCTGAACTTCT

dCas9

GTCTTTTTTGGTGGAAGAAGACAAGAAGCATGAACGTCATCCTATTTTTGGAAATATAGTAGATGAAGTTGCTTATCATGAGAAATATCCAACTATCTATCATCTGC

CAGAAAAAACCACCTTCTTCTGTTCTTCGTACTTGCAGTAGGATAAAAACCTTTATATCATCTACTTCAACGAATAGTACTCTTTATAGGTTGATAGATAGTAGACG

dCas9

GAAAAAAATTGGTAGATTCTACTGATAAAGCGGATTTGCGCTTAATCTATTTGGCCTTAGCGCATATGATTAAGTTTCGTGGTCATTTTTTGATTGAGGGAGATTTA

CTTTTTTTAACCATCTAAGATGACTATTTCGCCTAAACGCGAATTAGATAAACCGGAATCGCGTATACTAATTCAAAGCACCAGTAAAAAACTAACTCCCTCTAAAT

dCas9

AATCCTGATAATAGTGATGTGGACAAACTATTTATCCAGTTGGTACAAACCTACAATCAATTATTTGAAGAAAACCCTATTAACGCAAGTGGAGTAGATGCTAAAGC

TTAGGACTATTATCACTACACCTGTTTGATAAATAGGTCAACCATGTTTGGATGTTAGTTAATAAACTTCTTTTGGGATAATTGCGTTCACCTCATCTACGATTTCG

dCas9

GATTCTTTCTGCACGATTGAGTAAATCAAGACGATTAGAAAATCTCATTGCTCAGCTCCCCGGTGAGAAGAAAAATGGCTTATTTGGGAATCTCATTGCTTTGTCAT

CTAAGAAAGACGTGCTAACTCATTTAGTTCTGCTAATCTTTTAGAGTAACGAGTCGAGGGGCCACTCTTCTTTTTACCGAATAAACCCTTAGAGTAACGAAACAGTA

dCas9

TGGGTTTGACCCCTAATTTTAAATCAAATTTTGATTTGGCAGAAGATGCTAAATTACAGCTTTCAAAAGATACTTACGATGATGATTTAGATAATTTATTGGCGCAA

ACCCAAACTGGGGATTAAAATTTAGTTTAAAACTAAACCGTCTTCTACGATTTAATGTCGAAAGTTTTCTATGAATGCTACTACTAAATCTATTAAATAACCGCGTT

dCas9

pJ2018 (8885 bp) (from 1178-2354 bp)

ATTGGAGATCAATATGCTGATTTGTTTTTGGCAGCTAAGAATTTATCAGATGCTATTTTACTTTCAGATATCCTAAGAGTAAATACTGAAATAACTAAGGCTCCCCT
TAACCTCTAGTTATACGACTAAACAAAAACCGTCGATTCTTAAATAGTCTACGATAAAATGAAAGTCTATAGGATTCTCATTTATGACTTTATTGATTCCGAGGGGA
dCas9

HindIII
ATCAGCTTCAATGATTAAACGCTACGATGAACATCATCAAGACTTGACTCTTTTAAAAGCTTTAGTTCGACAACAACTTCCAGAAAAGTATAAAGAAATCTTTTTTG
TAGTCGAAGTTACTAATTTGCGATGCTACTTGTAGTAGTTCTGAACTGAGAAAATTTTCGAAATCAAGCTGTTGTTGAAGGTCTTTTCATATTTCTTTAGAAAAAAC
dCas9

ATCAATCAAAAAACGGATATGCAGGTTATATTGATGGGGGAGCTAGCCAAGAAGAATTTTATAAATTTATCAAACCAATTTTAGAAAAAATGGATGGTACTGAGGAA
TAGTTAGTTTTTTGCCTATACGTCCAATATAACTACCCCCTCGATCGGTTCTTCTTAAAATATTTAAATAGTTTGGTTAAAATCTTTTTTACCTACCATGACTCCTT
dCas9

TTATTGGTGAAACTAAATCGTGAAGATTTGCTGCGCAAGCAACGGACCTTTGACAACGGCTCTATTCCCCATCAAATTCACTTGGGTGAGCTGCATGCTATTTTGAG
AATAACCACTTTGATTTAGCACTTCTAAACGACGCGTTCGTTGCCTGGAAACTGTTGCCGAGATAAGGGGTAGTTTAAGTGAACCCACTCGACGTACGATAAAACTC
dCas9

EcoRI
AAGACAAGAAGACTTTTATCCATTTTTAAAAGACAATCGTGAGAAGATTGAAAAAATCTTGACTTTTCGAATTCCTTATTATGTTGGTCCATTGGCGCGTGGCAATA
TTCTGTTCTTCTGAAAATAGGTAAAAATTTTCTGTTAGCACTCTTCTAACTTTTTTAGAACTGAAAAGCTTAAGGAATAATACAACCAGGTAACCGCGCACCGTTAT
dCas9

NcoI
GTCGTTTTGCATGGATGACTCGGAAGTCTGAAGAAACAATTACCCCATGGAATTTTGAAGAAGTTGTCGATAAAGGTGCTTCAGCTCAATCATTTATTGAACGCATG
CAGCAAAACGTACCTACTGAGCCTTCAGACTTCTTTGTTAATGGGGTACCTTAAAACTTCTTCAACAGCTATTTCCACGAAGTCGAGTTAGTAAATAACTTGCGTAC
dCas9

ACAAACTTTGATAAAAATCTTCCAAATGAAAAAGTACTACCAAAACATAGTTTGCTTTATGAGTATTTTACGGTTTATAACGAATTGACAAAGGTCAAATATGTTAC
TGTTTGAAACTATTTTTAGAAGGTTTACTTTTTCATGATGGTTTTGTATCAAACGAAATACTCATAAAATGCCAAATATTGCTTAACTGTTTCCAGTTTATACAATG
dCas9

TGAAGGAATGCGAAAACCAGCATTTCTTTCAGGTGAACAGAAGAAAGCCATTGTTGATTTACTCTTCAAAACAAATCGAAAAGTAACCGTTAAGCAATTAAAAGAAG
ACTTCCTTACGCTTTTGGTCGTAAAGAAAGTCCACTTGTCTTCTTTCGGTAACAACTAAATGAGAAGTTTTGTTTAGCTTTTCATTGGCAATTCGTTAATTTTCTTC
dCas9

KpnI
ATTATTTCAAAAAAATAGAATGTTTTGATAGTGTTGAAATTTCAGGAGTTGAAGATAGATTTAATGCTTCATTAGGTACCTACCATGATTTGCTAAAAATTATTAAA
TAATAAAGTTTTTTTATCTTACAAAACTATCACAACTTTAAAGTCCTCAACTTCTATCTAAATTACGAAGTAATCCATGGATGGTACTAAACGATTTTTAATAATTT
dCas9

GATAAAGATTTTTTGGATAATGAAGAAAATGAAGATATCTTAGAGGATATTGTTTTAACATTGACCTTATTTGAAGATAGGGAGATGATTGAGGAAAGACTTAAAAC
CTATTTCTAAAAAACCTATTACTTCTTTTACTTCTATAGAATCTCCTATAACAAAATTGTAACTGGAATAAACTTCTATCCCTCTACTAACTCCTTTCTGAATTTTG
dCas9

ATATGCTCACCTCTTTGATGATAAGGTGATGAAACAGCTTAAACGTCGCCGTTATACTGGTTGGGGACGTTTGTCTCGAAAATTGATTAATGGTATTAGGGATAAGC
TATACGAGTGGAGAAACTACTATTCCACTACTTTGTCGAATTTGCAGCGGCAATATGACCAACCCCTGCAAACAGAGCTTTTAACTAATTACCATAATCCCTATTCG
dCas9

AATCTGGCAAAACAATATTAGATTTTTTGAAATCAGATGGTTTTGCCAATCGCAATTTTATGCAGCTGATCCATGATGATAGTTTGACATTTAAAGAAGACATTCAA
TTAGACCGTTTTGTTATAATCTAAAAAACTTTAGTCTACCAAAACGGTTAGCGTTAAAATACGTCGACTAGGTACTACTATCAAACTGTAAATTTCTTCTGTAAGTT
dCas9

AAAGCACAAGTGTCTGGACAAGGCGATAGTTTACATGAACATATTGCAAATTTAGCTGGTAGCCCTGCTATTAAAAAAGGTATTTTACAGACTGTAAAAGTTGTTGA
TTTCGTGTTCACAGACCTGTTCCGCTATCAAATGTACTTGTATAACGTTTAAATCGACCATCGGGACGATAATTTTTTCCATAAAATGTCTGACATTTTCAACAACT
dCas9

TGAATTGGTCAAAGTAATGGGGCGGCATAAGCCAGAAAATATCGTTATTGAAATGGCACGTGAAAATCAGACAACTCAAAAGGGCCAGAAAAATTCGCGAGAGCGTA
ACTTAACCAGTTTCATTACCCCGCCGTATTCGGTCTTTTATAGCAATAACTTTACCGTGCACTTTTAGTCTGTTGAGTTTTCCCGGTCTTTTTAAGCGCTCTCGCAT
dCas9

TGAAACGAATCGAAGAAGGTATCAAAGAATTAGGAAGTCAGATTCTTAAAGAGCATCCTGTTGAAAATACTCAATTGCAAAATGAAAAGCTCTATCTCTATTATCTC
ACTTTGCTTAGCTTCTTCCATAGTTTCTTAATCCTTCAGTCTAAGAATTTCTCGTAGGACAACTTTTATGAGTTAACGTTTTACTTTTCGAGATAGAGATAATAGAG
dCas9

CAAAATGGAAGAGACATGTATGTGGACCAAGAATTAGATATTAATCGTTTAAGTGATTATGATGTCGATGCCATTGTTCCACAAAGTTTCCTTAAAGACGATTCAAT
GTTTTACCTTCTCTGTACATACACCTGGTTCTTAATCTATAATTAGCAAATTCACTAATACTACAGCTACGGTAACAAGGTGTTTCAAAGGAATTTCTGCTAAGTTA
dCas9

AGACAATAAGGTCTTAACGCGTTCTGATAAAAATCGTGGTAAATCGGATAACGTTCCAAGTGAAGAAGTAGTCAAAAAGATGAAAAACTATTGGAGACAACTTCTAA
TCTGTTATTCCAGAATTGCGCAAGACTATTTTTAGCACCATTTAGCCTATTGCAAGGTTCACTTCTTCATCAGTTTTTCTACTTTTTGATAACCTCTGTTGAAGATT
dCas9

ACGCCAAGTTAATCACTCAACGTAAGTTTGATAATTTAACGAAAGCTGAACGTGGAGGTTTGAGTGAACTTGATAAAGCTGGTTTTATCAAACGCCAATTGGTTGAA
TGCGGTTCAATTAGTGAGTTGCATTCAAACTATTAAATTGCTTTCGACTTGCACCTCCAAACTCACTTGAACTATTTCGACCAAAATAGTTTGCGGTTAACCAACTT
dCas9

ACTCGCCAAATCACTAAGCATGTGGCACAAATTTTGGATAGTCGCATGAATACTAAATACGATGAAAATGATAAACTTATTCGAGAGGTTAAAGTGATTACCTTAAA
TGAGCGGTTTAGTGATTCGTACACCGTGTTTAAAACCTATCAGCGTACTTATGATTTATGCTACTTTTACTATTTGAATAAGCTCTCCAATTTCACTAATGGAATTT
dCas9

ATCTAAATTAGTTTCTGACTTCCGAAAAGATTTCCAATTCTATAAAGTACGTGAGATTAACAATTACCATCATGCCCATGATGCGTATCTAAATGCCGTCGTTGGAA
TAGATTTAATCAAAGACTGAAGGCTTTTCTAAAGGTTAAGATATTTCATGCACTCTAATTGTTAATGGTAGTACGGGTACTACGCATAGATTTACGGCAGCAACCTT
dCas9

CTGCTTTGATTAAGAAATATCCAAAACTTGAATCGGAGTTTGTCTATGGTGATTATAAAGTTTATGATGTTCGTAAAATGATTGCTAAGTCTGAGCAAGAAATAGGC
GACGAAACTAATTCTTTATAGGTTTTGAACTTAGCCTCAAACAGATACCACTAATATTTCAAATACTACAAGCATTTTACTAACGATTCAGACTCGTTCTTTATCCG
dCas9

AAAGCAACCGCAAAATATTTCTTTTACTCTAATATCATGAACTTCTTCAAAACAGAAATTACACTTGCAAATGGAGAGATTCGCAAACGCCCTCTAATCGAAACTAA
TTTCGTTGGCGTTTTATAAAGAAAATGAGATTATAGTACTTGAAGAAGTTTTGTCTTTAATGTGAACGTTTACCTCTCTAAGCGTTTGCGGGAGATTAGCTTTGATT
dCas9

TGGGGAAACTGGAGAAATTGTCTGGGATAAAGGGCGAGATTTTGCCACAGTGCGCAAAGTATTGTCCATGCCCCAAGTCAATATTGTCAAGAAAACAGAAGTACAGA
ACCCCTTTGACCTCTTTAACAGACCCTATTTCCCGCTCTAAAACGGTGTCACGCGTTTCATAACAGGTACGGGGTTCAGTTATAACAGTTCTTTTGTCTTCATGTCT
dCas9

pJ2018 (8885 bp) (from 3639-4815 bp)

HindIII BamHI

CAGGCGGATTCTCCAAGGAGTCAATTTTACCAAAAAGAAATTCGGACAAGCTTATTGCTCGTAAAAAGACTGGGATCCAAAAAAATATGGTGGTTTTGATAGTCCA
GTCCGCCTAAGAGGTTCCTCAGTTAAAATGGTTTTTCTTTAAGCCTGTTCGAATAACGAGCATTTTTCTGACCCTAGGTTTTTTTATACCACCAAAACTATCAGGT
dCas9

ACGGTAGCTTATTCAGTCCTAGTGGTTGCTAAGGTGGAAAAAGGGAAATCGAAGAAGTTAAAATCCGTTAAAGAGTTACTAGGGATCACAATTATGGAAAGAAGTTC
TGCCATCGAATAAGTCAGGATCACCAACGATTCCACCTTTTTCCCTTTAGCTTCTTCAATTTTAGGCAATTTCTCAATGATCCCTAGTGTTAATACCTTTCTTCAAG
dCas9

CTTTGAAAAAAATCCGATTGACTTTTTAGAAGCTAAAGGATATAAGGAAGTTAAAAAAGACTTAATCATTAAACTACCTAAATATAGTCTTTTTGAGTTAGAAAACG
GAAACTTTTTTTAGGCTAACTGAAAAATCTTCGATTTCCTATATTCCTTCAATTTTTTCTGAATTAGTAATTTGATGGATTTATATCAGAAAAACTCAATCTTTTGC
dCas9

GTCGTAAACGGATGCTGGCTAGTGCCGGAGAATTACAAAAAGGAAATGAGCTGGCTCTGCCAAGCAAATATGTGAATTTTTTATATTTAGCTAGTCATTATGAAAAG
CAGCATTTGCCTACGACCGATCACGGCCTCTTAATGTTTTTCCTTTACTCGACCGAGACGGTTCGTTTATACACTTAAAAAATATAAATCGATCAGTAATACTTTTC
dCas9

TTGAAGGGTAGTCCAGAAGATAACGAACAAAAACAATTGTTTGTGGAGCAGCATAAGCATTATTTAGATGAGATTATTGAGCAAATCAGTGAATTTTCTAAGCGTGT
AACTTCCCATCAGGTCTTCTATTGCTTGTTTTTGTTAACAAACACCTCGTCGTATTCGTAATAAATCTACTCTAATAACTCGTTTAGTCACTTAAAAGATTCGCACA
dCas9

TATTTTAGCAGATGCCAATTTAGATAAAGTTCTTAGTGCATATAACAAACATAGAGACAAACCAATACGTGAACAAGCAGAAAATATTATTCATTTATTTACGTTGA
ATAAAATCGTCTACGGTTAAATCTATTTCAAGAATCACGTATATTGTTTGTATCTCTGTTTGGTTATGCACTTGTTCGTCTTTTATAATAAGTAAATAAATGCAACT
dCas9

SacI

CGAATCTTGGAGCTCCCGCTGCTTTTAAATATTTTGATACAACAATTGATCGTAAACGATATACGTCTACAAAAGAAGTTTTAGATGCCACTCTTATCCATCAATCC
GCTTAGAACCTCGAGGGCGACGAAAATTTATAAAACTATGTTGTTAACTAGCATTTGCTATATGCAGATGTTTTCTTCAAAATCTACGGTGAGAATAGGTAGTTAGG
dCas9

NcoI

ATCACTGGTCTTTATGAAACACGCATTGATTTGAGTCAGCTAGGAGGTGACTAATCGCTGGGACGCCCGCCATGGTTCAGCCAAAAAACTTAAGACCGCCGGTCTTG
TAGTGACCAGAAATACTTTGTGCGTAACTAAACTCAGTCGATCCTCCACTGATTAGCGACCCTGCGGGCGGTACCAAGTCGGTTTTTTGAATTCTGGCGGCCAGAAC
dCas9 Suffix ECK120029600 Terminator

TCCACTACCTTGCAGTAATGCGGTGGACAGGATCGGCGGTTTTCTTTTCTCTTCTCAATTCTTCTGACCTGTAACGAATAATAGATAGTAAAGTAGTCTCCGATTGA
AGGTGATGGAACGTCATTACGCCACCTGTCCTAGCCGCCAAAAGAAAAGAGAAGAGTTAAGAAGACTGGACATTGCTTATTATCTATCATTTCATCAGAGGCTAACT
ECK120029600 Terminator Spacer 1

GTTTTCTCTGCCGAGTCCCACCCAGTTCTGTGATTTCAGTAAGTTGGTAATTGATACACTGTTGCGAGAACTGCTGCCTGGTAGTAGATAGGTTGTTATTGAGTAAG
CAAAAGAGACGGCTCAGGGTGGGTCAAGACACTAAAGTCATTCAACCATTAACTATGTGACAACGCTCTTGACGACGGACCATCATCTATCCAACAATAACTCATTC
Spacer 1

HindIII

AAGGTAAAGTGAACGAAATCCCTGAAACTGAGACTGTAGAAAATAAGCTTCAGCCTGCGGTCCGGTTTACGGCTAGCTCAGTCCTAGGTATTATGCTAGCTCGCTGG
TTCCATTTCACTTGCTTTAGGGACTTTGACTCTGACATCTTTTATTCGAAGTCGGACGCCAGGCCAAATGCCGATCGAGTCAGGATCCATAATACGATCGAGCGACC
Spacer 1 Prefix P(BBa_J23150)

pJ2018 (8885 bp) (from 4816-5992 bp)

KpnI EcoRI

GACGCCCGGGGACTACACTTACGAAACTATTGATTGCTCAGCCTGCGGTCCGGggtaccattaaagaggagaaagaattcATGAAAAACATCAACGCCGACGATACG
CTGCGGGCCCCTGATGTGAATGCTTTGATAACTAACGAGTCGGACGCCAGGCCccatggtaatttctcctctttcttaagTACTTTTTGTAGTTGCGGCTGCTATGC

Linker_1 Prefix RBS LuxR

HindIII

TACCGCATCATCAACAAAATCAAAGCTTGTCGCTCAAACAACGACATCAACCAGTGCCTGAGCGATATGACCAAAATGGTTCATTGCGAATATTACCTGCTGGCGAT
ATGGCGTAGTAGTTGTTTTAGTTTCGAACAGCGAGTTTGTTGCTGTAGTTGGTCACGGACTCGCTATACTGGTTTTACCAAGTAACGCTTATAATGGACGACCGCTA

LuxR

TATCTATCCGCACAGTATGGTCAAATCAGATATTTCGATCCTGGACAACTACCCGAAAAAATGGCGTCAGTATTACGATGACGCCAATCTGATTAAATATGATCCGA
ATAGATAGGCGTGTCATACCAGTTTAGTCTATAAAGCTAGGACCTGTTGATGGGCTTTTTTACCGCAGTCATAATGCTACTGCGGTTAGACTAATTTATACTAGGCT

LuxR

TCGTGGACTACAGTAACTCCAATCACTCCCCGATTAACTGGAATATCTTTGAAAACAATGCGGTCAACAAAAAATCTCCGAACGTGATCAAAGAAGCCAAAACCAGC
AGCACCTGATGTCATTGAGGTTAGTGAGGGGCTAATTGACCTTATAGAAACTTTTGTTACGCCAGTTGTTTTTTAGAGGCTTGCACTAGTTTCTTCGGTTTTGGTCG

LuxR

GGCCTGATTACGGGTTTTTCGTTCCCGATCCATACCGCAAACAATGGCTTTGGTATGCTGAGCTTCGCTCACTCTGAAAAAGATAACTATATTGACAGCCTGTTCCT
CCGGACTAATGCCCAAAAAGCAAGGGCTAGGTATGGCGTTTGTTACCGAAACCATACGACTCGAAGCGAGTGAGACTTTTTCTATTGATATAACTGTCGGACAAGGA

LuxR

GCATGCATGCATGAATATTCCGCTGATCGTGCCGTCTCTGGTTGATAACTACCGTAAAATCAACATCGCTAACAACAAAAGTAACAATGACCTGACCAAACGCGAAA
CGTACGTACGTACTTATAAGGCGACTAGCACGGCAGAGACCAACTATTGATGGCATTTTAGTTGTAGCGATTGTTGTTTTCATTGTTACTGGACTGGTTTGCGCTTT

LuxR

AAGAATGCCTGGCGTGGGCCTGTGAAGGCAAAAGCTCTTGGGATATTAGTAAAATCCTGGGTTGTTCCGAACGTACCGTTACGTTTCATCTGACGAACGCGCAAATG
TTCTTACGGACCGCACCCGGACACTTCCGTTTTCGAGAACCCTATAATCATTTTAGGACCCAACAAGGCTTGCATGGCAATGCAAAGTAGACTGCTTGCGCGTTTAC

LuxR

AAACTGAACACCACGAATCGCTGCCAATCCATCTCCAAAGCAATCCTGACCGGCGCTATCGACTGTCCGTACTTCAAAAATTAATAATCGCTGGGACGCCCGCCTGC
TTTGACTTGTGGTGCTTAGCGACGGTTAGGTAGAGGTTTCGTTAGGACTGGCCGCGATAGCTGACAGGCATGAAGTTTTTAATTATTAGCGACCCTGCGGGCGGACG

LuxR Suffix

SbfI KpnI

AGGCTCGGTACCAAATTCCAGAAAAGAGGCCTCCCGAAAGGGGGGCCTTTTTTCGTTTTGGTCCTACTGATTGGGTGAACTACGATACTTCCAGAATAACGAGAAAT
TCCGAGCCATGGTTTAAGGTCTTTTCTCCGGAGGGCTTTCCCCCCGGAAAAAAGCAAAACCAGGATGACTAACCCACTTGATGCTATGAAGGTCTTATTGCTCTTTA

L3S2P21 Terminator Spacer 2

ACGCTACTCTTACAGATAAAACTCACTGCCGATTACGGATAGGACGAACAACACTTTGATTATCTTACGATTTACCGAAGGTTCTATTGAGTTGGACCCAGCAGTTA
TGCGATGAGAATGTCTATTTTGAGTGACGGCTAATGCCTATCCTGCTTGTTGTGAAACTAATAGAATGCTAAATGGCTTCCAAGATAACTCAACCTGGGTCGTCAAT

Spacer 2

SalI

CTACTTTTATTCAGTGATTCTGTCTTATCGGGAGATTGTTCGCTTACCACGTCGACCAGCCTGCGGTCCGGTTTACGGCTAGCTCAGTCCTAGGTATTATGCTAGCT
GATGAAAATAAGTCACTAAGACAGAATAGCCCTCTAACAAGCGAATGGTGCAGCTGGTCGGACGCCAGGCCAAATGCCGATCGAGTCAGGATCCATAATACGATCGA

Spacer 2 Prefix P(BBa_J23150)

pJ2018 (8885 bp) (from 5993-7276 bp)

KpnI EcoRI

```
CGCTGGGACGCCCGAGATAGCCGTTACACAGGTGACACTTATTTCAGCCTGCGGTCCGGggtaccattaaagaggagaaagaattcATGGACCACTACCTCGACATT
GCGACCCTGCGGGCTCTATCGGCAATGTGTCCACTGTGAATAAAGTCGGACGCCAGGCCccatggtaatttctcctctttcttaagTACCTGGTGATGGAGCTGTAA
```

Suffix | Linker_24 | Prefix | RBS | Csy4

```
CGCTTGCGACCGGACCCGGAATTTCCCCCGGCGCAACTCATGAGCGTGCTCTTCGGCAAGCTCCACCAGGCCCTGGTGGCACAGGGCGGGGACAGGATCGGCGTGAG
GCGAACGCTGGCCTGGGCCTTAAAGGGGGCCGCGTTGAGTACTCGCACGAGAAGCCGTTCGAGGTGGTCCGGGACCACCGTGTCCCGCCCCTGTCCTAGCCGCACTC
```

Csy4

```
CTTCCCCGACCTCGACGAAAGCCGCTCCCGGCTGGGCGAGCGCCTGCGCATTCATGCCTCGGCGGACGACCTTCGTGCCCTGCTCGCCCGGCCCTGGCTGGAAGGGT
GAAGGGGCTGGAGCTGCTTTCGGCGAGGGCCGACCCGCTCGCGGACGCGTAAGTACGGAGCCGCCTGCTGGAAGCACGGGACGAGCGGGCCGGGACCGACCTTCCCA
```

Csy4

```
TGCGGGACCATCTGCAATTCGGAGAACCGGCAGTCGTGCCTCACCCCACACCGTACCGTCAGGTCAGTCGGGTTCAGGCGAAAAGCAATCCGGAACGCCTGCGGCGG
ACGCCCTGGTAGACGTTAAGCCTCTTGGCCGTCAGCACGGAGTGGGGTGTGGCATGGCAGTCCAGTCAGCCCAAGTCCGCTTTTCGTTAGGCCTTGCGGACGCCGCC
```

Csy4

```
CGGCTCATGCGCCGGCACGATCTGAGTGAGGAGGAGGCTCGGAAACGCATTCCCGATACGGTCGCGAGAGCCTTGGACCTGCCCTTCGTCACGCTACGCAGCCAGAG
GCCGAGTACGCGGCCGTGCTAGACTCACTCCTCCTCCGAGCCTTTGCGTAAGGGCTATGCCAGCGCTCTCGGAACCTGGACGGGAAGCAGTGCGATGCGTCGGTCTC
```

Csy4

```
CACCGGACAGCACTTCCGTCTCTTCATCCGCCACGGGCCGTTGCAGGTGACGGCAGAGGAAGGAGGATTCACCTGTTACGGGTTGAGCAAAGGAGGTTTCGTTCCCT
GTGGCCTGTCGTGAAGGCAGAGAAGTAGGCGGTGCCCGGCAACGTCCACTGCCGTCTCCTTCCTCCTAAGTGGACAATGCCCAACTCGTTTCCTCCAAAGCAAGGGA
```

Csy4

NotI

```
GGTTCTGAtaaTCGCTGGGACGCCCGGCGGCCGCggaaacacagAAAAAGCCCGCACCTGACAGTGCGGGCTTTTTTTTTcgaccaaaggTAGCGAACGACGAGTC
CCAAGACTattAGCGACCCTGCGGGCCGCCGGCGcctttgtgtcTTTTTCGGGCGTGGACTGTCACGCCCGAAAAAAAAAgctggtttccATCGCTTGCTGCTCAG
```

Csy4 | Suffix | ECK120033737 Terminator | Linker_8

KasI BbvCI AscI

```
ACTGTTGAGGATAAATACTTTCTCTACTAGGCGCCTGTTACACAGGTCCTCAGCGGCGCGCCTGCTGCCACCGCTGAGCAATAACTAGCATAACCCCTTGGGGCCTC
TGACAACTCCTATTTATGAAAGAGATGATCCGCGGACAATGTGTCCAGGAGTCGCCGCGCGGACGACGGTGGCGACTCGTTATTGATCGTATTGGGGAACCCCGGAG
```

Linker_8 | Spa...15

```
TAAACGGGTCTTGAGGGGTTTTTTGCCAGGCATCAAATAAAACGAAAGGCTCAGTCGGAAGACTGGGCCTTTCGTTTTATCTGTTGTTTGTCGGTGAACGCTCTCCT
ATTTGCCCAGAACTCCCCAAAAAACGGTCCGTAGTTTATTTTGCTTTCCGAGTCAGCCTTCTGACCCGGAAAGCAAAATAGACAACAAACAGCCACTTGCGAGAGGA
```

```
GAGTAGGACAAATCCGCCGGGAGCGGATTTGAACGTTGTGAAGCAACGGCCCGGAGGGTGGCGGGCAGGACGCCCGCCATAAACTGCCAGGCATCAAACTAAGCAGA
CTCATCCTGTTTAGGCGGCCCTCGCCTAAACTTGCAACACTTCGTTGCCGGGCCTCCCACCGCCCGTCCTGCGGGCGGTATTTGACGGTCCGTAGTTTGATTCGTCT
```

```
AGGCCATCCTGACGGATGGCCTTTTTGCGTTTCAGATCTaccggtaaaccagcaatagacataagcggctatttaacgaccctgccctgaaccgacgaccgggtcat
TCCGGTAGGACTGCCTACCGGAAAAACGCAAAGTCTAGAtggccatttggtcgttatctgtattcgccgataaattgctgggacgggacttggctgctggcccagta
```

```
cgtggccggatcttgcggcccctcggcttgaacgaattgttagacattatttgccgactaccttggtgatctcgcctttcacgtagtggacaaattcttccaactga
gcaccggcctagaacgccggggagccgaacttgcttaacaatctgtaataaacggctgatggaaccactagagcggaaagtgcatcacctgtttaagaaggttgact
```

SpecR

tctgcgcgcgaggccaagcgatcttcttcttgtccaagataagcctgtctagcttcaagtatgacgggctgatactgggccggcaggcgctccattgcccagtcggc
agacgcgcgctccggttcgctagaagaagaacaggttctattcggacagatcgaagttcatactgcccgactatgacccggccgtccgcgaggtaacgggtcagccg
SpecR

agcgacatccttcggcgcgattttgccggttactgcgctgtaccaaatgcgggacaacgtaagcactacatttcgctcatcgccagcccagtcgggcggcgagttcc
tcgctgtaggaagccgcgctaaaacggccaatgacgcgacatggtttacgccctgttgcattcgtgatgtaaagcgagtagcggtcgggtcagcccgccgctcaagg
SpecR

atagcgttaaggtttcatttagcgcctcaaatagatcctgttcaggaaccggatcaaagagttcctccgccgctggacctaccaaggcaacgctatgttctcttgct
tatcgcaattccaaagtaaatcgcggagtttatctaggacaagtccttggcctagtttctcaaggaggcggcgacctggatggttccgttgcgatacaagagaacga
SpecR

tttgtcagcaagatagccagatcaatgtcgatcgtggctggctcgaagatacctgcaagaatgtcattgcgctgccattctccaaattgcagttcgcgcttagctgg
aaacagtcgttctatcggtctagttacagctagcaccgaccgagcttctatggacgttcttacagtaacgcgacggtaagaggtttaacgtcaagcgcgaatcgacc
SpecR

ataacgccacggaatgatgtcgtcgtgcacaacaatggtgacttctacagcgcggagaatctcgctctctccaggggaagccgaagtttccaaaaggtcgttgatca
tattgcggtgccttactacagcagcacgtgttgttaccactgaagatgtcgcgcctcttagagcgagagaggtccccttcggcttcaaaggttttccagcaactagt
SpecR

aagctcgccgcgttgtttcatcaagccttacggtcaccgtaaccagcaaatcaatatcactgtgtggcttcaggccgccatccactgcggagccgtacaaatgtacg
ttcgagcggcgcaacaaagtagttcggaatgccagtggcattggtcgtttagttatagtgacacaccgaagtccggcggtaggtgacgcctcggcatgtttacatgc
SpecR

gccagcaacgtcggttcgagatggcgctcgatgacgccaactacctctgatagttgagtcgatacttcggcgatcaccgcttccctcatactcttcctttttcaata
cggtcgttgcagccaagctctaccgcgagctactgcggttgatggagactatcaactcagctatgaagccgctagtggcgaagggagtatgagaaggaaaaagttat
SpecR

ttattgaagcatttatcagggttattgtctcatgagcggatacatatttgaatgtatttagaaaaataaacaaatagctagctcactcggtcgctacgctccgggcg
aataacttcgtaaatagtcccaataacagagtactcgcctatgtataaacttacataaatctttttatttgtttatcgatcgagtgagccagcgatgcgaggcccgc

tgagactgcggcgggcgctgcggacacatacaaagttacccacagattccgtggataagcaggggactaacatgtgaggcaaaacagcagggccgcgccggtggcgt
actctgacgccgcccgcgacgcctgtgtatgtttcaatgggtgtctaaggcacctattcgtcccctgattgtacactccgttttgtcgtcccggcgcggccaccgca
CDF ori

ttttccataggctccgcccctcctgccagagttcacataaacagacgcttttccggtgcatctgtgggagccgtgaggctcaaccatgaatctgacagtacgggcgaa
aaaaggtatccgaggcgggaggacggtctcaagtgtatttgtctgcgaaaaggccacgtagacaccctcggcactccgagttggtacttagactgtcatgcccgctt
CDF ori

acccgacaggacttaaagatccccaccgtttccggcgggtcgctccctcttgcgctctcctgttccgaccctgccgtttaccggatacctgttccgcctttctccct
tgggctgtcctgaatttctaggggtggcaaaggccgcccagcgagggagaacgcgagaggacaaggctgggacggcaaatggcctatggacaaggcggaaagaggga
CDF ori

tacgggaagtgtggcgctttctcatagctcacacactggtatctcggctcggtgtaggtcgttcgctccaagctgggctgtaagcaagaactccccgttcagcccga
atgcccttcacaccgcgaaagagtatcgagtgtgtgaccatagagccgagccacatccagcaagcgaggttcgacccgacattcgttcttgaggggcaagtcgggct
CDF ori

pJ2018 (8885 bp) (from 8561-8885 bp)

ctgctgcgccttatccggtaactgttcacttgagtccaacccggaaaagcacggtaaaacgccactggcagcagccattggtaactgggagttcgcagaggatttgt
gacgacgcggaataggccattgacaagtgaactcaggttgggccttttcgtgccattttgcggtgaccgtcgtcggtaaccattgaccctcaagcgtctcctaaaca

CDF ori

ttagctaaacacgcggttgctcttgaagtgtgcgccaaagtccggctacactggaaggacagatttggttgctgtgctctgcgaaagccagttaccacggttaagca
aatcgatttgtgcgccaacgagaacttcacacgcggtttcaggccgatgtgaccttcctgtctaaaccaacgacacgagacgctttcggtcaatggtgccaattcgt

CDF ori

gttccccaactgacttaaccttcgatcaaaccacctccccaggtggtttttttcgtttacagggcaaaagattacgcgcagaaaaaaaggatctcaagaagatccttt
caaggggttgactgaattggaagctagtttggtggaggggtccaccaaaaaaagcaaatgtcccgttttctaatgcgcgtcttttttttcctagagttcttctaggaaa

CDF ori

gatc
ctag

(from 1-1177 bp)

pJ2039 (7459 bp)

ACTTTTCATACTCCCGCCATTCAGAGAAGAAACCAATTGTCCATATTGCATCAGACATTGCCGTCACTGCGTCTTTTACTGGCTCTTCTCGCTAACCAAACCGGTAA
TGAAAAGTATGAGGGCGGTAAGTCTCTTCTTTGGTTAACAGGTATAACGTAGTCTGTAACGGCAGTGACGCAGAAAATGACCGAGAAGAGCGATTGGTTTGGCCATT

CCCCGCTTATTAAAAGCATTCTGTAACAAAGCGGGACCAAAGCCATGACAAAAACGCGTAACAAAAGTGTCTATAATCACGGCAGAAAAGTCCACATTGATTATTTG
GGGGCGAATAATTTTCGTAAGACATTGTTTCGCCCTGGTTTCGGTACTGTTTTTGCGCATTGTTTTCACAGATATTAGTGCCGTCTTTTCAGGTGTAACTAATAAAC

EcoRI

CACGGCGTCACACTTTGCTATGCCATAGCATTTTTATCCATAAGATTAGCGGtTCCTACCTGACGCTTTTTATCGCAACTCTCTACTGTTTCTCCATACCGAATTCA
GTGCCGCAGTGTGAAACGATACGGTATCGTAAAAATAGGTATTCTAATCGCCaAGGATGGACTGCGAAAAATAGCGTTGAGAGATGACAAAGAGGTATGGCTTAAGT

P(BAD) promoter

SacI

TAGGATAGATTCTGGAAACTTTACCGTCCGAGCTCCAGCCTGCGGTCCGGGTTCACTGCCGTATAGGCAGTGACTGAGCTAGTGTACTCTGTTTcAGAGCTATGCTG
ATCCTATCTAAGACCTTTGAAATGGCAGGCTCGAGGTCGGACGCCAGGCCCAAGTGACGGCATATCCGTCACTGACTCGATCACATGAGACAAAgTCTCGATACGAC

Linker_14 | Prefix | Csy4 site | sgRNA-3

XhoI

GAAACAGCATAGCAAGTTgAAATAAGGCTAGTCCGTTATCAACTTGAAAAAGTGGCACCGAGTCGGTGCGTTCACTGCCGTATAGGCAGTCGCTGGGACGCCCGCTC
CTTTGTCGTATCGTTCAAcTTTATTCCGATCAGGCAATAGTTGAACTTTTTCACCGTGGCTCAGCCACGCAAGTGACGGCATATCCGTCAGCGACCCTGCGGGCGAG

sgRNA-3 | Csy4 site | Suffix

GAGCAATAAACAGTTGATAGGGCTTCTCCGTTACAGCCTGCGGTCCGGGTTCACTGCCGTATAGGCAGTAATTTTGTTTAACTTTAAGAAGGAGATATACATATGGT
CTCGTTATTTGTCAACTATCCCGAAGAGGCAATGTCGGACGCCAGGCCCAAGTGACGGCATATCCGTCATTAAAACAAATTGAAATTCTTCCTCTATATGTATACCA

Linker_0 | Prefix | Csy4 site | RBS

TTCGGTTATCAAACCAGAGATGAAAATGCGTTACTATATGGATGGTTCAGTAAATGGTCACGAATTTACTATTGAGGGCGAGGGTACGGGACGCCCATACGAGGGGC
AAGCCAATAGTTTGGTCTCTACTTTTACGCAATGATATACCTACCAAGTCATTTACCAGTGCTTAAATGATAACTCCCGCTCCCATGCCCTGCGGGTATGCTCCCCG

mKO2

ACCAGGAAATGACTTTACGCGTCACAATGGCTGAAGGCGGGCCTATGCCGTTTGCGTTCGATCTTGTTAGTCATGTCTTTTGTTACGGTCACCGTGTATTTACTAAA
TGGTCCTTTACTGAAATGCGCAGTGTTACCGACTTCCGCCCGGATACGGCAAACGCAAGCTAGAACAATCAGTACAGAAAACAATGCCAGTGGCACATAAATGATTT

mKO2

TACCCCGAGGAAATTCCAGACTATTTCAAACAAGCCTTCCCGGAAGGTTTGTCTTGGGAGCGCAGTTTAGAGTTTGAAGACGGTGGCTCGGCCAGCGTGTCAGCTCA
ATGGGGCTCCTTTAAGGTCTGATAAAGTTTGTTCGGAAGGGCCTTCCAAACAGAACCCTCGCGTCAAATCTCAAACTTCTGCCACCGAGCCGGTCGCACAGTCGAGT

mKO2

TATTAGTCTTCGCGGCAATACATTTTATCACAAGTCAAAGTTCACCGGCGTGAACTTCCCCGCAGACGGCCCAATCATGCAGAATCAAAGTGTTGATTGGGAACCGT
ATAATCAGAAGCGCCGTTATGTAAAATAGTGTTCAGTTTCAAGTGGCCGCACTTGAAGGGGCGTCTGCCGGGTTAGTACGTCTTAGTTTCACAACTAACCCTTGGCA

mKO2

CCACAGAGAAGATTACAGCTTCCGATGGAGTCTTAAAGGGCGATGTAACCATGTACTTAAAATTAGAAGGGGGAGGGAACCATAAATGTCAGATGAAGACTACCTAT
GGTGTCTCTTCTAATGTCGAAGGCTACCTCAGAATTTCCCGCTACATTGGTACATGAATTTTAATCTTCCCCCTCCCTTGGTATTTACAGTCTACTTCTGATGGATA

mKO2

pJ2039 (7459 bp) (from 1178-2354 bp)

AAGGCCGCAAAAGAGATTCTTGAAATGCCCGGAGACCACTACATTGGGCATCGTTTGGTCCGTAAGACAGAAGGAAATATTACTGAACAGGTCGAAGACGCTGTGGC
TTCCGGCGTTTTCTCTAAGAACTTTACGGGCCTCTGGTGATGTAACCCGTAGCAAACCAGGCATTCTGTCTTCCTTTATAATGACTTGTCCAGCTTCTGCGACACCG
mKO2

ACACAGCATGTCCCGCCGTAATACTGACGCCATCACAATCCACAGCATCCTGGATTGGATTGAAGACAACTTGGAGTCGCCGTTGAGTTTAGAAAAAGTTAGTGAAC
TGTGTCGTACAGGGCGGCATTATGACTGCGGTAGTGTTAGGTGTCGTAGGACCTAACCTAACTTCTGTTGAACCTCAGCGGCAACTCAAATCTTTTTCAATCACTTG
MarA

GTAGTGGTTACTCAAAGTGGCACCTTCAGCGCATGTTTAAGAAGGAAACGGGTCATTCATTGGGTCAATATATTCGTTCTCGCAAGATGACTGAAATTGCCCAGAAA
CATCACCAATGAGTTTCACCGTGGAAGTCGCGTACAAATTCTTCCTTTGCCCAGTAAGTAACCCAGTTATATAAGCAAGAGCGTTCTACTGACTTTAACGGGTCTTT
MarA

TTGAAAGAGTCTAATGAACCTATTTTGTACCTGGCGGAGCGTTACGGCTTTGAAAGTCAGCAAACCCTTACACGTACCTTCAAGAATTACTTTGACGTTCCACCACA
AACTTTCTCAGATTACTTGGATAAAACATGGACCGCCTCGCAATGCCGAAACTTTCAGTCGTTTGGGAATGTGCATGGAAGTTCTTAATGAAACTGCAAGGTGGTGT
MarA

NcoI
CAAATATCGTATGACCAACATGCAGGGTGAGTCACGTTTTTTGCATCCGTTGAATCATTACAATTCCTAATAATCGCTGGGACGCCCGCCATGGTTCAGCCAAAAAA
GTTTATAGCATACTGGTTGTACGTCCCACTCAGTGCAAAAAACGTAGGCAACTTAGTAATGTTAAGGATTATTAGCGACCCTGCGGGCGGTACCAAGTCGGTTTTTT
MarA | Suffix | ECK...or

CTTAAGACCGCCGGTCTTGTCCACTACCTTGCAGTAATGCGGTGGACAGGATCGGCGGTTTTCTTTTCTCTTCTCAATTCTTCTGACCTGTAACGAATAATAGATAG
GAATTCTGGCGGCCAGAACAGGTGATGGAACGTCATTACGCCACCTGTCCTAGCCGCCAAAAGAAAAGAGAAGAGTTAAGAAGACTGGACATTGCTTATTATCTATC
ECK120029600 Terminator | Spacer 1

TAAAGTAGTCTCCGATTGAGTTTTCTCTGCCGAGTCCCACCCAGTTCTGTGATTTCAGTAAGTTGGTAATTGATACACTGTTGCGAGAACTGCTGCCTGGTAGTAGA
ATTTCATCAGAGGCTAACTCAAAAGAGACGGCTCAGGGTGGGTCAAGACACTAAAGTCATTCAACCATTAACTATGTGACAACGCTCTTGACGACGGACCATCATCT
Spacer 1

HindIII
TAGGTTGTTATTGAGTAAGAAGGTAAAGTGAACGAAATCCCTGAAACTGAGACTGTAGAAAATAAGCTTCAGCCTGCGGTCCGGTTGACAGCTAGCTCAGTCCTAGG
ATCCAACAATAACTCATTCTTCCATTTCACTTGCTTTAGGGACTTTGACTCTGACATCTTTTATTCGAAGTCGGACGCCAGGCCAACTGTCGATCGAGTCAGGATCC
Spacer 1 | Prefix | P(BBa_J23102)

TACTGTGCTAGCTCGCTGGGACGCCCGGGGACTACACTTACGAAACTATTGATTGCTCAGCCTGCGGTCCGGccaAGAGTACACTAGCTCAGTCATCGCTGGGACGC
ATGACACGATCGAGCGACCCTGCGGGCCCCTGATGTGAATGCTTTGATAACTAACGAGTCGGACGCCAGGCCggtTCTCATGTGATCGAGTCAGTAGCGACCCTGCG
P(B...2) | Suffix | Linker_1 | Prefix | bs-3 | Suffix

BamHI
CCGGGATCCAAGAGATTTCTACACGATTGAGCACTGTCTCAGCCTGCGGTCCGGGTTCACTGCCGTATAGGCAGTAATTTTGTTTAACTTTAAGAAGGAGATATACA
GGCCCTAGGTTCTCTAAAGATGTGCTAACTCGTGACAGAGTCGGACGCCAGGCCCAAGTGACGGCATATCCGTCATTAAAACAAATTGAAATTCTTCCTCTATATGT
Linker_10 | Prefix | Csy4 site | RBS

TATGCGTAAAGGCGAAGAACTGTTTACCGGTGTGGTTCCGATTCTGGTGGAACTGGACGGCGATGTTAATGGTCATAAATTCAGTGTTCGCGGCGAAGGTGAAGGCG
ATACGCATTTCCGCTTCTTGACAAATGGCCACACCAAGGCTAAGACCACCTTGACCTGCCGCTACAATTACCAGTATTTAAGTCACAAGCGCCGCTTCCACTTCCGC
sfGFP

pJ2039 (7459 bp) (from 2355-3531 bp)

ATGCGACGAACGGCAAACTGACCCTGAAATTTATCTGCACCACGGGTAAACTGCCGGTCCCGTGGCCGACGCTGGTGACCACGCTGACCTATGGCGTTCAATGTTTT
TACGCTGCTTGCCGTTTGACTGGGACTTTAAATAGACGTGGTGCCCATTTGACGGCCAGGGCACCGGCTGCGACCACTGGTGCGACTGGATACCGCAAGTTACAAAA
sfGFP

GCGCGTTACCCGGATCACATGAAACAGCACGACTTTTTCAAATCGGCCATGCCGGAAGGCTATGTGCAGGAACGTACGATTAGCTTTAAAGACGATGGTACGTATAA
CGCGCAATGGGCCTAGTGTACTTTGTCGTGCTGAAAAAGTTTAGCCGGTACGGCCTTCCGATACACGTCCTTGCATGCTAATCGAAATTTCTGCTACCATGCATATT
sfGFP

AACCCGCGCGGAAGTGAAATTCGAAGGCGATACCCTGGTTAACCGTATCGAACTGAAAGGTATCGATTTCAAAGAAGACGGCAATATTCTGGGTCATAAACTGGAAT
TTGGGCGCGCCTTCACTTTAAGCTTCCGCTATGGGACCAATTGGCATAGCTTGACTTTCCATAGCTAAAGTTTCTTCTGCCGTTATAAGACCCAGTATTTGACCTTA
sfGFP

ATAACTTCAATTCCCACAACGTGTACATCACCGCGGATAAACAGAAAAACGGCATTAAAGCCAATTTCAAAATCCGCCATAATGTGGAAGATGGTAGCGTTCAGCTG
TATTGAAGTTAAGGGTGTTGCACATGTAGTGGCGCCTATTTGTCTTTTTGCCGTAATTTCGGTTAAAGTTTTAGGCGGTATTACACCTTCTACCATCGCAAGTCGAC
sfGFP

GCCGACCACTATCAGCAAAACACGCCGATTGGTGATGGCCCGGTCCTGCTGCCGGACAATCACTACCTGAGTACCCAGTCCGTGCTGTCAAAAGATCCGAACGAAAA
CGGCTGGTGATAGTCGTTTTGTGCGGCTAACCACTACCGGGCCAGGACGACGGCCTGTTAGTGATGGACTCATGGGTCAGGCACGACAGTTTTCTAGGCTTGCTTTT
sfGFP

ACGTGACCACATGGTCCTGCTGGAATTTGTGACGGCTGCGGGTATCACCCACGGCATGGACGAACTGTATAAAATGTCCCGCCGTAATACTGACGCCATCACAATCC
TGCACTGGTGTACCAGGACGACCTTAAACACTGCCGACGCCCATAGTGGGTGCCGTACCTGCTTGACATATTTTACAGGGCGGCATTATGACTGCGGTAGTGTTAGG
sfGFP | MarAn20

SbfI | KpnI
ACAGCATCCTGGATTGGATTGAAGACTAATAATCGCTGGGACGCCCGCCTGCAGGCTCGGTACCAAATTCCAGAAAAGAGGCCTCCCGAAAGGGGGGCCTTTTTTCG
TGTCGTAGGACCTAACCTAACTTCTGATTATTAGCGACCCTGCGGGCGGACGTCCGAGCCATGGTTTAAGGTCTTTTCTCCGGAGGGCTTTCCCCCCGGAAAAAAGC
MarAn20 | Suffix | L3S2P21 Terminator

TTTTGGTCCTAATAGATAAAGGATAGGTCTGGTAGTGTTGTTCGTTCTCGCAGGTAAATCAATAATACTCAGCAGTTCCGTAGACTTTTCAGTGGGACAGGGTAGCG
AAAACCAGGATTATCTATTTCCTATCCAGACCATCACAACAAGCAAGAGCGTCCATTTAGTTATTATGAGTCGTCAAGGCATCTGAAAAGTCACCCTGTCCCATCGC
Spacer 2.5

SalI
ATAACAGATAGATTGTAATAAGACACAGTAGGTGCTCGTAGTTGCGTGAAGAGAACCGCTCAGGAAATCCAGTCAGAAGTATTGGTAATCGTTGAAAACTCAGTCGA
TATTGTCTATCTAACATTATTCTGTGTCATCCACGAGCATCAACGCACTTCTCTTGGCGAGTCCTTTAGGTCAGTCTTCATAACCATTAGCAACTTTTGAGTCAGCT
Spacer 2.5

AatII
CGCACTTACTGAAGACGTCCTATTACACTCGTCGTTGGAAACTGAAGATCAGCCTGCGGTCCGGGTTCACTGCCGTATAGGCAGTAATTTTGTTTAACTTTAAGAAG
GCGTGAATGACTTCTGCAGGATAATGTGAGCAGCAACCTTTGACTTCTAGTCGGACGCCAGGCCCAAGTGACGGCATATCCGTCATTAAAACAAATTGAAATTCTTC
Spa... 5 | Linker_11 | Prefix | Csy4 site | RBS

GAGATATACATATGAATCAGTCATTCATCTCGGACATCTTATATGCCGACATCGAATCGAAGGCTAAGGAACTTACAGTCAATTCCAACAATACTGTCCAGCCGGTC
CTCTATATGTATACTTAGTCAGTAAGTAGAGCCTGTAGAATATACGGCTGTAGCTTAGCTTCCGATTCCTTGAATGTCAGTTAAGGTTGTTATGACAGGTCGGCCAG
RBS | RepA70

pJ2039 (7459 bp) (from 3532-4815 bp)

GCGCTTATGCGCTTAGGAGTTTTCGTTCCCAAACCTTCCAAGAGCAAAGGAGAAAGTAAGGAAATTGACGCCACCAAAGCCTTCTCTCAACTGGAGATTGCTAAAGC
CGCGAATACGCGAATCCTCAAAAGCAAGGGTTTGGAAGGTTCTCGTTTCCTCTTTCATTCCTTTAACTGCGGTGGTTTCGGAAGAGAGTTGACCTCTAACGATTTCG
RepA70

AGAGGGCatggttagtaaaggagaagaaaataacatggcaCTGATTAAGGAGAACATGCACATGAAGCTGTACATGGAGGGCACCGTGAACAACCACCACTTCAAGT
TCTCCCGtaccaatcatttcctcttcttttattgtaccgtGACTAATTCCTCTTGTACGTGTACTTCGACATGTACCTCCCGTGGCACTTGTTGGTGGTGAAGTTCA
mKate2

GCACATCCGAGGGCGAAGGCAAGCCCTACGAGGGCACCCAGACCATGAGAATCAAGgccGTCGAGGGCGGCCCTCTCCCCTTCGCCTTCGACATCCTGGCTACCAGC
CGTGTAGGCTCCCGCTTCCGTTCGGGATGCTCCCGTGGGTCTGGTACTCTTAGTTCcggCAGCTCCCGCCGGGAGAGGGGAAGCGGAAGCTGTAGGACCGATGGTCG
mKate2

TTCATGTACGGCAGCAAAACCTTCATCAACCACACCCAGGGCATCCCCGACTTCTTTAAGCAGTCCTTCCCTGAGGGCTTCACATGGGAGAGAGTCACCACATACGA
AAGTACATGCCGTCGTTTTGGAAGTAGTTGGTGTGGGTCCCGTAGGGGCTGAAGAAATTCGTCAGGAAGGGACTCCCGAAGTGTACCCTCTCTCAGTGGTGTATGCT
mKate2

AGACGGGGGCGTGCTGACCGCTACCCAGGACACCAGCCTCCAGGACGGCTGCCTCATCTACAACGTCAAGATCAGAGGGGTGAACTTCCCATCCAACGGCCCTGTGA
TCTGCCCCCGCACGACTGGCGATGGGTCCTGTGGTCGGAGGTCCTGCCGACGGAGTAGATGTTGCAGTTCTAGTCTCCCCACTTGAAGGGTAGGTTGCCGGGACACT
mKate2

TGCAGAAGAAAACACTCGGCTGGGAGGCCTCCACCGAGaccCTGTACCCCGCTGACGGCGGCCTGGAAGGCAGAgcCGACATGGCCCTGAAGCTCGTGGGCGGGGGC
ACGTCTTCTTTTGTGAGCCGACCCTCCGGAGGTGGCTCtggGACATGGGGCGACTGCCGCCGGACCTTCCGTCTcgGCTGTACCGGGACTTCGAGCACCCGCCCCCG
mKate2

CACCTGATCTGCAACTTGAAGACCACATACAGATCCAAGAAACCCGCTAAGAACCTCAAGATGCCCGGCGTCTACTATGTGGACAGAAGACTGGAAAGAATCAAGGA
GTGGACTAGACGTTGAACTTCTGGTGTATGTCTAGGTTCTTTGGGCGATTCTTGGAGTTCTACGGGCCGCAGATGATACACCTGTCTTCTGACCTTTCTTAGTTCCT
mKate2

GGCCGACAAAGAGACCTACGTCGAGCAGCACGAGGTGGCTGTGGCCAGATACTGCGACCTCCCTAGCAAACTGGGGCACAgAgtctaATAATCGCTGGGACGCCCGG
CCGGCTGTTTCTCTGGATGCAGCTCGTCGTGCTCCACCGACACCGGTCTATGACGCTGGAGGGATCGTTTGACCCCGTGTcTcagatTATTAGCGACCCTGCGGGCC
mKate2 Suffix

NotI

CGGCCGCggaaacacagAAAAAAGCCCGCACCTGACAGTGCGGGCTTTTTTTTTcgaccaaaggTAGCGAACGACGAGTCACTGTTGAGGATAAATACTTTCTCTAC
GCCGGCGcctttgtgtcTTTTTTCGGGCGTGGACTGTCACGCCCGAAAAAAAAAgctggtttccATCGCTTGCTGCTCAGTGACAACTCCTATTTATGAAAGAGATG
ECK120033737 Terminator Linker_8

KasI BbvCI AscI

TAGGCGCCTGTTACACAGGTCCTCAGCGGCGCGCCTTTGTCGGTGAACGCTCTCCTGAGTAGGACAAATCCGCCGGGAGCGGATTTGAACGTTGTGAAGCAACGGCC
ATCCGCGGACAATGTGTCCAGGAGTCGCCGCGCGGAAACAGCCACTTGCGAGAGGACTCATCCTGTTTAGGCGGCCCTCGCCTAAACTTGCAACACTTCGTTGCCGG
Spa...15

CGGAGGGTGGCGGGCAGGACGCCCGCCATAAACTGCCAGGCATCAAACTAAGCAGAAGGCCATCCTGACGGATGGCCTTTTTGCGTTTCAGATCTACCGGTaaacca
GCCTCCCACCGCCCGTCCTGCGGGCGGTATTTGACGGTCCGTAGTTTGATTCGTCTTCCGGTAGGACTGCCTACCGGAAAAACGCAAAGTCTAGATGGCCAtttggt

gcaatagacataagcggctatttaacgaccctgccctgaaccgacgacaagctgacgaccgggtctccgcaagtggcacttttcggggaaatgtgcgcggaacccct
cgttatctgtattcgccgataaattgctgggacgggacttggctgctgttcgactgctggcccagaggcgttcaccgtgaaaagcccctttacacgcgccttgggga

pJ2039 (7459 bp) (from 4816-6099 bp)

atttgtttatttttctaaatacattcaaatatgtatccgctcatgaattaattcttagaaaaactcatcgagcatcaaatgaaactgcaatttattcatatcaggat
taaacaaataaaaagatttatgtaagtttatacataggcgagtacttaattaagaatctttttgagtagctcgtagtttactttgacgttaaataagtatagtccta
KanR

tatcaataccatatttttgaaaaagccgtttctgtaatgaaggagaaaactcaccgaggcagttccataggatggcaagatcctggtatcggtctgcgattccgact
atagttatggtataaaaactttttcggcaaagacattacttcctcttttgagtggctccgtcaaggtatcctaccgttctaggaccatagccagacgctaaggctga
KanR

cgtccaacatcaatacaacctattaatttcccctcgtcaaaaataaggttatcaagtgagaaatcaccatgagtgacgactgaatccggtgagaatggcaaaagttt
gcaggttgtagttatgttggataattaaaggggagcagtttttattccaatagttcactctttagtggtactcactgctgacttaggccactcttaccgttttcaaa
KanR

atgcatttctttccagacttgttcaacaggccagccattacgctcgtcatcaaaatcactcgcatcaaccaaaccgttattcattcgtgattgcgcctgagcgagac
tacgtaaagaaaggtctgaacaagttgtccggtcggtaatgcgagcagtagttttagtgagcgtagttggtttggcaataagtaagcactaacgcggactcgctctg
KanR

gaaatacgcggtcgctgttaaaaggacaattacaaacaggaatcgaatgcaaccggcgcaggaacactgccagcgcatcaacaatattttcacctgaatcaggatat
ctttatgcgccagcgacaattttcctgttaatgtttgtccttagcttacgttggccgcgtccttgtgacggtcgcgtagttgttataaaagtggacttagtcctata
KanR

tcttctaatacctggaatgctgttttcccggggatcgcagtggtgagtaaccatgcatcatcaggagtacggataaaatgcttgatggtcggaagaggcataaattc
agaagattatggaccttacgacaaaagggcccctagcgtcaccactcattggtacgtagtagtcctcatgcctattttacgaactaccagccttctccgtatttaag
KanR

cgtcagccagtttagtctgaccatctcatctgtaacatcattggcaacgctacctttgccatgtttcagaaacaactctggcgcatcgggcttcccatacaatcgat
gcagtcggtcaaatcagactggtagagtagacattgtagtaaccgttgcgatggaaacggtacaaagtctttgttgagaccgcgtagcccgaagggtatgttagcta
KanR

agattgtcgcacctgattgcccgacattatcgcgagcccatttatacccatataaatcagcatccatgttggaatttaatcgcggcctagagcaagacgtttcccgt
tctaacagcgtggactaacgggctgtaatagcgctcgggtaaatatgggtatatttagtcgtaggtacaaccttaaattagcgccggatctcgttctgcaaagggca
KanR

tgaatatggctcatactcttcctttttcaatattattgaagcatttatcagggttattgtctcatgagcggatacatatttgaatgtatttagaaaaataaacaaat
acttataccgagtatgagaaggaaaaagttataataacttcgtaaatagtcccaataacagagtactcgcctatgtataaacttacataaatctttttatttgttta
KanR

aggcatgctagcgcagaaacgtcctagaagatgccaggaggatacttagcagagagacaataaggccggagcgaagccgtttttccataggctccgcccccctgacg
tccgtacgatcgcgtctttgcaggatcttctacggtcctcctatgaatcgtctctctgttattccggcctcgcttcggcaaaaaggtatccgaggcggggggactgc
ColA ori

aacatcacgaaatctgacgctcaaatcagtggtggcgaaacccgacaggactataaagataccaggcgtttccccctgatggctccctcttgcgctctcctgttccc
ttgtagtgctttagactgcgagtttagtcaccaccgctttgggctgtcctgatatttctatggtccgcaaagggggactaccgagggagaacgcgagaggacaaggg
ColA ori

gtcctgcggcgtccgtgttgtggtggaggctttacccaaatcaccacgtcccgttccgtgtagacagttcgctccaagctgggctgtgtgcaagaaccccccgttca
caggacgccgcaggcacaacaccacctccgaaatgggtttagtggtgcagggcaaggcacatctgtcaagcgaggttcgacccgacacacgttcttggggggcaagt
ColA ori

gcccgactgctgcgccttatccggtaactatcatcttgagtccaacccggaaagacacgacaaaacgccactggcagcagccattggtaactgagaattagtggatt
cgggctgacgacgcggaataggccattgatagtagaactcaggttgggcctttctgtgctgttttgcggtgaccgtcgtcggtaaccattgactcttaatcacctaa
ColA ori

tagatatcgagagtcttgaagtggtggcctaacagaggctacactgaaggacagtatttggtatctgcgctccactaaagccagttaccaggttaagcagttcccc
atctatagctctcagaacttcaccaccggattgtctccgatgtgactttcctgtcataaaccatagacgcgaggtgatttcggtcaatggtccaattcgtcaagggg
ColA ori

aactgacttaaccttcgatcaaaccgcctccccaggcggttttttcgtttacagagcaggagattacgacgatcgtaaaaggatctcaagaagatcctttacggatt
ttgactgaattggaagctagtttggcggaggggtccgccaaaaaagcaaatgtctcgtcctctaatgctgctagcattttcctagagttcttctaggaaatgcctaa
ColA ori

cccgacaccatcactctagatttcagtgcaatttatctcttcaaatgtagcacctgaagtcagccccatacgatataagttgtaattctcatgttagtcatgccccg
gggctgtggtagtgagatctaaagtcacgttaaatagagaagtttacatcgtggacttcagtcggggtatgctatattcaacattaagagtacaatcagtacggggc
Co...i

cgcccaccggaaggagctgactgggttgCTCCTAgGGTCTGATTCGTTACCAATTATGACAACTTGACGGCTACATCATTCACTTTTTCTTCACAACCGGCACGGAA
gcgggtggccttcctcgactgacccaacGAGGATcCCAGACTAAGCAATGGTTAATACTGTTGAACTGCCGATGTAGTAAGTGAAAAAGAAGTGTTGGCCGTGCCTT
araC

CTCGCTCGGGCTGGCCCCGGTGCATTTTTTAAATACCCGCGAGAAATAGAGTTGATCGTCAAAACCAACATTGCGACCGACGGTGGCGATAGGCATCCGGGTGGTGC
GAGCGAGCCCGACCGGGGCCACGTAAAAAATTTATGGGCGCTCTTTATCTCAACTAGCAGTTTTGGTTGTAACGCTGGCTGCCACCGCTATCCGTAGGCCCACCACG
araC

TCAAAAGCAGCTTCGCCTGGCTGATACGTTGGTCCTCGCGCCAGCTTAAGACGCTAATCCCTAACTGCTGGCGGAAAAGATGTGACAGACGCGACGGCGACAAGCAA
AGTTTTCGTCGAAGCGGACCGACTATGCAACCAGGAGCGCGGTCGAATTCTGCGATTAGGGATTGACGACCGCCTTTTCTACACTGTCTGCGCTGCCGCTGTTCGTT
araC

ACATGCTGTGCGACGCTGGCGATATCAAAATTGCTGTCTGCCAGGTGATCGCTGATGTACTGACAAGCCTCGCGTACCCGATTATCCATCGGTGGATGGAGCGACTC
TGTACGACACGCTGCGACCGCTATAGTTTTAACGACAGACGGTCCACTAGCGACTACATGACTGTTCGGAGCGCATGGGCTAATAGGTAGCCACCTACCTCGCTGAG
araC

GTTAATCGCTTCCATGCGCCGCAGTAACAATTGCTCAAGCAGATTTATCGCCAGCAGCTCCGAATAGCGCCCTTCCCCTTGCCCGGCGTTAATGATTTGCCCAAACA
CAATTAGCGAAGGTACGCGGCGTCATTGTTAACGAGTTCGTCTAAATAGCGGTCGTCGAGGCTTATCGCGGGAAGGGGAACGGGCCGCAATTACTAAACGGGTTTGT
araC

GGTCGCTGAAATGCGGCTGGTGCGCTTCATCCGGGCGAAAGAACCCCGTATTGGCAAATATTGACGGCCAGTTAAGCCATTCATGCCAGTAGGCGCGCGGACGAAAG
CCAGCGACTTTACGCCGACCACGCGAAGTAGGCCCGCTTTCTTGGGGCATAACCGTTTATAACTGCCGGTCAATTCGGTAAGTACGGTCATCCGCGCGCCTGCTTTC
araC

TAAACCCACTGGTGATACCATTCGCGAGCCTCCGGATGACGACCGTAGTGATGAATCTCTCCTGGCGGGAACAGCAAAATATCACCCGGTCGGCAAACAAATTCTCG
ATTTGGGTGACCACTATGGTAAGCGCTCGGAGGCCTACTGCTGGCATCACTACTTAGAGAGGACCGCCCTTGTCGTTTTATAGTGGGCCAGCCGTTTGTTTAAGAGC
araC

TCCCTGATTTTTCACCACCCCCTGACCGCGAATGGTGAGATTGAGAATATAACCTTTCATTCCCAGCGGTCGGTCGATAAAAAAATCGAGATAACCGTTGGCCTCAA
AGGGACTAAAAAGTGGTGGGGGACTGGCGCTTACCACTCTAACTCTTATATTGGAAAGTAAGGGTCGCCAGCCAGCTATTTTTTTAGCTCTATTGGCAACCGGAGTT
araC

TCGGCGTTAAACCCGCCACCAGATGGGCATTAAACGAGTATCCCGGCAGCAGGGGATCATTTTGCGCTTCAGCCAT
AGCCGCAATTTGGGCGGTGGTCTACCCGTAATTTGCTCATAGGGCCGTCGTCCCCTAGTAAAACGCGAAGTCGGTA

araC

(from 1-1284 bp)

pJ2039_N2only (7295 bp)

ACTTTTCATACTCCCGCCATTCAGAGAAGAAACCAATTGTCCATATTGCATCAGACATTGCCGTCACTGCGTCTTTTACTGGCTCTTCTCGCTAACCAAACCGGTAA
TGAAAAGTATGAGGGCGGTAAGTCTCTTCTTTGGTTAACAGGTATAACGTAGTCTGTAACGGCAGTGACGCAGAAAATGACCGAGAAGAGCGATTGGTTTGGCCATT

CCCCGCTTATTAAAAGCATTCTGTAACAAAGCGGGACCAAAGCCATGACAAAAACGCGTAACAAAAGTGTCTATAATCACGGCAGAAAAGTCCACATTGATTATTTG
GGGGCGAATAATTTTCGTAAGACATTGTTTCGCCCTGGTTTCGGTACTGTTTTTGCGCATTGTTTTCACAGATATTAGTGCCGTCTTTTCAGGTGTAACTAATAAAC

EcoRI

CACGGCGTCACACTTTGCTATGCCATAGCATTTTTATCCATAAGATTAGCGGtTCCTACCTGACGCTTTTTATCGCAACTCTCTACTGTTTCTCCATACCGAATTCA
GTGCCGCAGTGTGAAACGATACGGTATCGTAAAAATAGGTATTCTAATCGCCaAGGATGGACTGCGAAAAATAGCGTTGAGAGATGACAAAGAGGTATGGCTTAAGT

P(BAD) promoter

SacI XhoI

TAGGATAGATTCTGGAAACTTTACCGTCCGAGCTCAGGCTTACCTTACTCGAGCAATAAACAGTTGATAGGGCTTCTCCGTTACAGCCTGCGGTCCGGGTTCACTGC
ATCCTATCTAAGACCTTTGAAATGGCAGGCTCGAGTCCGAATGGAATGAGCTCGTTATTTGTCAACTATCCCGAAGAGGCAATGTCGGACGCCAGGCCCAAGTGACG

Linker_14 REs..._8 Linker_0 Prefix

CGTATAGGCAGTAATTTTGTTTAACTTTAAGAAGGAGATATACATATGGTTTCGGTTATCAAACCAGAGATGAAAATGCGTTACTATATGGATGGTTCAGTAAATGG
GCATATCCGTCATTAAAACAAATTGAAATTCTTCCTCTATATGTATACCAAAGCCAATAGTTTGGTCTCTACTTTTACGCAATGATATACCTACCAAGTCATTTACC

Cs...e RBS mKO2

TCACGAATTTACTATTGAGGGCGAGGGTACGGGACGCCCATACGAGGGGCACCAGGAAATGACTTTACGCGTCACAATGGCTGAAGGCGGGCCTATGCCGTTTGCGT
AGTGCTTAAATGATAACTCCCGCTCCCATGCCCTGCGGGTATGCTCCCCGTGGTCCTTTACTGAAATGCGCAGTGTTACCGACTTCCGCCCGGATACGGCAAACGCA

mKO2

TCGATCTTGTTAGTCATGTCTTTTGTTACGGTCACCGTGTATTTACTAAATACCCCGAGGAAATTCCAGACTATTTCAAACAAGCCTTCCCGGAAGGTTTGTCTTGG
AGCTAGAACAATCAGTACAGAAAACAATGCCAGTGGCACATAAATGATTTATGGGGCTCCTTTAAGGTCTGATAAAGTTTGTTCGGAAGGGCCTTCCAAACAGAACC

mKO2

GAGCGCAGTTTAGAGTTTGAAGACGGTGGCTCGGCCAGCGTGTCAGCTCATATTAGTCTTCGCGGCAATACATTTTATCACAAGTCAAAGTTCACCGGCGTGAACTT
CTCGCGTCAAATCTCAAACTTCTGCCACCGAGCCGGTCGCACAGTCGAGTATAATCAGAAGCGCCGTTATGTAAAATAGTGTTCAGTTTCAAGTGGCCGCACTTGAA

mKO2

CCCCGCAGACGGCCCAATCATGCAGAATCAAAGTGTTGATTGGGAACCGTCCACAGAGAAGATTACAGCTTCCGATGGAGTCTTAAAGGGCGATGTAACCATGTACT
GGGGCGTCTGCCGGGTTAGTACGTCTTAGTTTCACAACTAACCCTTGGCAGGTGTCTCTTCTAATGTCGAAGGCTACCTCAGAATTTCCCGCTACATTGGTACATGA

mKO2

TAAAATTAGAAGGGGGAGGGAACCATAAATGTCAGATGAAGACTACCTATAAGGCCGCAAAAGAGATTCTTGAAATGCCCGGAGACCACTACATTGGGCATCGTTTG
ATTTTAATCTTCCCCCTCCCTTGGTATTTACAGTCTACTTCTGATGGATATTCCGGCGTTTTCTCTAAGAACTTTACGGGCCTCTGGTGATGTAACCCGTAGCAAAC

mKO2

GTCCGTAAGACAGAAGGAAATATTACTGAACAGGTCGAAGACGCTGTGGCACACAGCATGTCCCGCCGTAATACTGACGCCATCACAATCCACAGCATCCTGGATTG
CAGGCATTCTGTCTTCCTTTATAATGACTTGTCCAGCTTCTGCGACACCGTGTGTCGTACAGGGCGGCATTATGACTGCGGTAGTGTTAGGTGTCGTAGGACCTAAC

mKO2 MarA

GATTGAAGACAACTTGGAGTCGCCGTTGAGTTTAGAAAAAGTTAGTGAACGTAGTGGTTACTCAAAGTGGCACCTTCAGCGCATGTTTAAGAAGGAAACGGGTCATT
CTAACTTCTGTTGAACCTCAGCGGCAACTCAAATCTTTTTCAATCACTTGCATCACCAATGAGTTTCACCGTGGAAGTCGCGTACAAATTCTTCCTTTGCCCAGTAA

MarA

pJ2039_N2only (7295 bp) (from 1285-2461 bp)

CATTGGGTCAATATATTCGTTCTCGCAAGATGACTGAAATTGCCCAGAAATTGAAAGAGTCTAATGAACCTATTTTGTACCTGGCGGAGCGTTACGGCTTTGAAAGT
GTAACCCAGTTATATAAGCAAGAGCGTTCTACTGACTTTAACGGGTCTTTAACTTTCTCAGATTACTTGGATAAAACATGGACCGCCTCGCAATGCCGAAACTTTCA
MarA

CAGCAAACCCTTACACGTACCTTCAAGAATTACTTTGACGTTCCACCACACAAATATCGTATGACCAACATGCAGGGTGAGTCACGTTTTTTGCATCCGTTGAATCA
GTCGTTTGGGAATGTGCATGGAAGTTCTTAATGAAACTGCAAGGTGGTGTGTTTATAGCATACTGGTTGTACGTCCCACTCAGTGCAAAAAACGTAGGCAACTTAGT
MarA

NcoI
TTACAATTCCTAATAATCGCTGGGACGCCCGCCATGGTTCAGCCAAAAAACTTAAGACCGCCGGTCTTGTCCACTACCTTGCAGTAATGCGGTGGACAGGATCGGCG
AATGTTAAGGATTATTAGCGACCCTGCGGGCGGTACCAAGTCGGTTTTTTGAATTCTGGCGGCCAGAACAGGTGATGGAACGTCATTACGCCACCTGTCCTAGCCGC
MarA | Suffix | | ECK120029600 Terminator

GTTTTCTTTTCTCTTCTCAATTCTTCTGACCTGTAACGAATAATAGATAGTAAAGTAGTCTCCGATTGAGTTTTCTCTGCCGAGTCCCACCCAGTTCTGTGATTTCA
CAAAAGAAAAGAGAAGAGTTAAGAAGACTGGACATTGCTTATTATCTATCATTTCATCAGAGGCTAACTCAAAAGAGACGGCTCAGGGTGGGTCAAGACACTAAAGT
ECK1200...inator | Spacer 1

GTAAGTTGGTAATTGATACACTGTTGCGAGAACTGCTGCCTGGTAGTAGATAGGTTGTTATTGAGTAAGAAGGTAAAGTGAACGAAATCCCTGAAACTGAGACTGTA
CATTCAACCATTAACTATGTGACAACGCTCTTGACGACGGACCATCATCTATCCAACAATAACTCATTCTTCCATTTCACTTGCTTTAGGGACTTTGACTCTGACAT
Spacer 1

HindIII
GAAAATAAGCTTCAGCCTGCGGTCCGGTTGACAGCTAGCTCAGTCCTAGGTACTGTGCTAGCTCGCTGGGACGCCCGGGGACTACACTTACGAAACTATTGATTGCT
CTTTTATTCGAAGTCGGACGCCAGGCCAACTGTCGATCGAGTCAGGATCCATGACACGATCGAGCGACCCTGCGGGCCCCTGATGTGAATGCTTTGATAACTAACGA
| | Prefix | P(BBa_J23102) | Suffix | Linker_1

BamHI
CAGCCTGCGGTCCGGccaAGAGTACACTAGCTCAGTCATCGCTGGGACGCCCGGGATCCAAGAGATTTCTACACGATTGAGCACTGTCTCAGCCTGCGGTCCGGGTT
GTCGGACGCCAGGCCggtTCTCATGTGATCGAGTCAGTAGCGACCCTGCGGGCCCTAGGTTCTCTAAAGATGTGCTAACTCGTGACAGAGTCGGACGCCAGGCCCAA
Prefix | bs-3 | Suffix | | Linker_10 | Prefix |

CACTGCCGTATAGGCAGTAATTTTGTTTAACTTTAAGAAGGAGATATACATATGCGTAAAGGCGAAGAACTGTTTACCGGTGTGGTTCCGATTCTGGTGGAACTGGA
GTGACGGCATATCCGTCATTAAAACAAATTGAAATTCTTCCTCTATATGTATACGCATTTCCGCTTCTTGACAAATGGCCACACCAAGGCTAAGACCACCTTGACCT
Csy4 site | RBS | sfGFP

CGGCGATGTTAATGGTCATAAATTCAGTGTTCGCGGCGAAGGTGAAGGCGATGCGACGAACGGCAAACTGACCCTGAAATTTATCTGCACCACGGGTAAACTGCCGG
GCCGCTACAATTACCAGTATTTAAGTCACAAGCGCCGCTTCCACTTCCGCTACGCTGCTTGCCGTTTGACTGGGACTTTAAATAGACGTGGTGCCCATTTGACGGCC
sfGFP

TCCCGTGGCCGACGCTGGTGACCACGCTGACCTATGGCGTTCAATGTTTTGCGCGTTACCCGGATCACATGAAACAGCACGACTTTTTCAAATCGGCCATGCCGGAA
AGGGCACCGGCTGCGACCACTGGTGCGACTGGATACCGCAAGTTACAAAACGCGCAATGGGCCTAGTGTACTTTGTCGTGCTGAAAAAGTTTAGCCGGTACGGCCTT
sfGFP

GGCTATGTGCAGGAACGTACGATTAGCTTTAAAGACGATGGTACGTATAAAACCCGCGCGGAAGTGAAATTCGAAGGCGATACCCTGGTTAACCGTATCGAACTGAA
CCGATACACGTCCTTGCATGCTAATCGAAATTTCTGCTACCATGCATATTTTGGGCGCGCCTTCACTTTAAGCTTCCGCTATGGGACCAATTGGCATAGCTTGACTT
sfGFP

pJ2039_N2only (7295 bp) (from 2462-3638 bp)

AGGTATCGATTTCAAAGAAGACGGCAATATTCTGGGTCATAAACTGGAATATAACTTCAATTCCCACAACGTGTACATCACCGCGGATAAACAGAAAAACGGCATTA
TCCATAGCTAAAGTTTCTTCTGCCGTTATAAGACCCAGTATTTGACCTTATATTGAAGTTAAGGGTGTTGCACATGTAGTGGCGCCTATTTGTCTTTTTGCCGTAAT
sfGFP

AAGCCAATTTCAAAATCCGCCATAATGTGGAAGATGGTAGCGTTCAGCTGGCCGACCACTATCAGCAAAACACGCCGATTGGTGATGGCCCGGTCCTGCTGCCGGAC
TTCGGTTAAAGTTTTAGGCGGTATTACACCTTCTACCATCGCAAGTCGACCGGCTGGTGATAGTCGTTTTGTGCGGCTAACCACTACCGGGCCAGGACGACGGCCTG
sfGFP

AATCACTACCTGAGTACCCAGTCCGTGCTGTCAAAAGATCCGAACGAAAAACGTGACCACATGGTCCTGCTGGAATTTGTGACGGCTGCGGGTATCACCCACGGCAT
TTAGTGATGGACTCATGGGTCAGGCACGACAGTTTTCTAGGCTTGCTTTTTGCACTGGTGTACCAGGACGACCTTAAACACTGCCGACGCCCATAGTGGGTGCCGTA
sfGFP

SbfI
GGACGAACTGTATAAAATGTCCCGCCGTAATACTGACGCCATCACAATCCACAGCATCCTGGATTGGATTGAAGACTAATAATCGCTGGGACGCCCGCCTGCAGGCT
CCTGCTTGACATATTTTACAGGGCGGCATTATGACTGCGGTAGTGTTAGGTGTCGTAGGACCTAACCTAACTTCTGATTATTAGCGACCCTGCGGGCGGACGTCCGA
sfGFP MarAn20 Suffix

KpnI
CGGTACCAAATTCCAGAAAAGAGGCCTCCCGAAAGGGGGGCCTTTTTTCGTTTTGGTCCTAATAGATAAAGGATAGGTCTGGTAGTGTTGTTCGTTCTCGCAGGTAA
GCCATGGTTTAAGGTCTTTTCTCCGGAGGGCTTTCCCCCCGGAAAAAAGCAAAACCAGGATTATCTATTTCCTATCCAGACCATCACAACAAGCAAGAGCGTCCATT
L3S2P21 Terminator Spacer 2.5

ATCAATAATACTCAGCAGTTCCGTAGACTTTTCAGTGGGACAGGGTAGCGATAACAGATAGATTGTAATAAGACACAGTAGGTGCTCGTAGTTGCGTGAAGAGAACC
TAGTTATTATGAGTCGTCAAGGCATCTGAAAAGTCACCCTGTCCCATCGCTATTGTCTATCTAACATTATTCTGTGTCATCCACGAGCATCAACGCACTTCTCTTGG
Spacer 2.5

SalI AatII
GCTCAGGAAATCCAGTCAGAAGTATTGGTAATCGTTGAAAACTCAGTCGACGCACTTACTGAAGACGTCCTATTACACTCGTCGTTGGAAACTGAAGATCAGCCTGC
CGAGTCCTTTAGGTCAGTCTTCATAACCATTAGCAACTTTTGAGTCAGCTGCGTGAATGACTTCTGCAGGATAATGTGAGCAGCAACCTTTGACTTCTAGTCGGACG
Spacer 2.5 Spa... 5 Linker_11

GGTCCGGGTTCACTGCCGTATAGGCAGTAATTTTGTTTAACTTTAAGAAGGAGATATACATATGAATCAGTCATTCATCTCGGACATCTTATATGCCGACATCGAAT
CCAGGCCCAAGTGACGGCATATCCGTCATTAAAACAAATTGAAATTCTTCCTCTATATGTATACTTAGTCAGTAAGTAGAGCCTGTAGAATATACGGCTGTAGCTTA
Csy4 site RBS RepA70

CGAAGGCTAAGGAACTTACAGTCAATTCCAACAATACTGTCCAGCCGGTCGCGCTTATGCGCTTAGGAGTTTTCGTTCCCAAACCTTCCAAGAGCAAAGGAGAAAGT
GCTTCCGATTCCTTGAATGTCAGTTAAGGTTGTTATGACAGGTCGGCCAGCGCGAATACGCGAATCCTCAAAAGCAAGGGTTTGGAAGGTTCTCGTTTCCTCTTTCA
RepA70

AAGGAAATTGACGCCACCAAAGCCTTCTCTCAACTGGAGATTGCTAAAGCAGAGGGCatggttagtaaaggagaagaaaataacatggcaCTGATTAAGGAGAACAT
TTCCTTTAACTGCGGTGGTTTCGGAAGAGAGTTGACCTCTAACGATTTCGTCTCCCGtaccaatcatttcctcttcttttattgtaccgtGACTAATTCCTCTTGTA
RepA70 mKate2

GCACATGAAGCTGTACATGGAGGGCACCGTGAACAACCACCACTTCAAGTGCACATCCGAGGGCGAAGGCAAGCCCTACGAGGGCACCCAGACCATGAGAATCAAGg
CGTGTACTTCGACATGTACCTCCCGTGGCACTTGTTGGTGGTGAAGTTCACGTGTAGGCTCCCGCTTCCGTTCGGGATGCTCCCGTGGGTCTGGTACTCTTAGTTCc
mKate2

pJ2039_N2only (7295 bp) (from 3639-4922 bp)

```
ccGTCGAGGGCGGCCCTCTCCCCTTCGCCTTCGACATCCTGGCTACCAGCTTCATGTACGGCAGCAAAACCTTCATCAACCACACCCAGGGCATCCCCGACTTCTTT
ggCAGCTCCCGCCGGGAGAGGGGAAGCGGAAGCTGTAGGACCGATGGTCGAAGTACATGCCGTCGTTTTGGAAGTAGTTGGTGTGGGTCCCGTAGGGGCTGAAGAAA
mKate2

AAGCAGTCCTTCCCTGAGGGCTTCACATGGGAGAGAGTCACCACATACGAAGACGGGGGCGTGCTGACCGCTACCCAGGACACCAGCCTCCAGGACGGCTGCCTCAT
TTCGTCAGGAAGGGACTCCCGAAGTGTACCCTCTCTCAGTGGTGTATGCTTCTGCCCCCGCACGACTGGCGATGGGTCCTGTGGTCGGAGGTCCTGCCGACGGAGTA
mKate2

CTACAACGTCAAGATCAGAGGGGTGAACTTCCCATCCAACGGCCCTGTGATGCAGAAGAAAACACTCGGCTGGGAGGCCTCCACCGAGaccCTGTACCCCGCTGACG
GATGTTGCAGTTCTAGTCTCCCCACTTGAAGGGTAGGTTGCCGGGACACTACGTCTTCTTTTGTGAGCCGACCCTCCGGAGGTGGCTCtggGACATGGGGCGACTGC
mKate2

GCGGCCTGGAAGGCAGAgcCGACATGGCCCTGAAGCTCGTGGGCGGGGGCCACCTGATCTGCAACTTGAAGACCACATACAGATCCAAGAAACCCGCTAAGAACCTC
CGCCGGACCTTCCGTCTcgGCTGTACCGGGACTTCGAGCACCCGCCCCCGGTGGACTAGACGTTGAACTTCTGGTGTATGTCTAGGTTCTTTGGGCGATTCTTGGAG
mKate2

AAGATGCCCGGCGTCTACTATGTGGACAGAAGACTGGAAAGAATCAAGGAGGCCGACAAAGAGACCTACGTCGAGCAGCACGAGGTGGCTGTGGCCAGATACTGCGA
TTCTACGGGCCGCAGATGATACACCTGTCTTCTGACCTTTCTTAGTTCCTCCGGCTGTTTCTCTGGATGCAGCTCGTCGTGCTCCACCGACACCGGTCTATGACGCT
mKate2

NotI
CCTCCCTAGCAAACTGGGGCACAgAgtctaATAATCGCTGGGACGCCCGGCGGCCGCggaaacacagAAAAAAGCCCGCACCTGACAGTGCGGGCTTTTTTTTTcga
GGAGGGATCGTTTGACCCCGTGTcTcagatTATTAGCGACCCTGCGGGCCGCCGGCGcctttgtgtcTTTTTTCGGGCGTGGACTGTCACGCCCGAAAAAAAAAgct
mKate2  Suffix  ECK120033737 Terminator

KasI  BbvCI  AscI
ccaaaggTAGCGAACGACGAGTCACTGTTGAGGATAAATACTTTCTCTACTAGGCGCCTGTTACACAGGTCCTCAGCGGCGCGCCTTTGTCGGTGAACGCTCTCCTG
ggtttccATCGCTTGCTGCTCAGTGACAACTCCTATTTATGAAAGAGATGATCCGCGGACAATGTGTCCAGGAGTCGCCGCGCGGAAACAGCCACTTGCGAGAGGAC
Linker_8  Spa...15

AGTAGGACAAATCCGCCGGGAGCGGATTTGAACGTTGTGAAGCAACGGCCCGGAGGGTGGCGGGCAGGACGCCCGCCATAAACTGCCAGGCATCAAACTAAGCAGAA
TCATCCTGTTTAGGCGGCCCTCGCCTAAACTTGCAACACTTCGTTGCCGGGCCTCCCACCGCCCGTCCTGCGGGCGGTATTTGACGGTCCGTAGTTTGATTCGTCTT

GGCCATCCTGACGGATGGCCTTTTTGCGTTTCAGATCTACCGGTaaaccagcaatagacataagcggctatttaacgaccctgccctgaaccgacgacaagctgacg
CCGGTAGGACTGCCTACCGGAAAAACGCAAAGTCTAGATGGCCAtttggtcgttatctgtattcgccgataaattgctgggacgggacttggctgctgttcgactgc

accgggtctccgcaagtggcacttttcggggaaatgtgcgcggaacccctatttgtttatttttctaaatacattcaaatatgtatccgctcatgaattaattctta
tggcccagaggcgttcaccgtgaaaagcccctttacacgcgccttggggataaacaaataaaaagatttatgtaagtttatacataggcgagtacttaattaagaat

gaaaaactcatcgagcatcaaatgaaactgcaatttattcatatcaggattatcaataccatatttttgaaaaagccgtttctgtaatgaaggagaaaactcaccga
ctttttgagtagctcgtagtttactttgacgttaaataagtatagtcctaatagttatggtataaaaactttttcggcaaagacattacttcctcttttgagtggct
KanR

ggcagttccataggatggcaagatcctggtatcggtctgcgattccgactcgtccaacatcaatacaacctattaatttcccctcgtcaaaaataaggttatcaagt
ccgtcaaggtatcctaccgttctaggaccatagccagacgctaaggctgagcaggttgtagttatgttggataattaaaggggagcagtttttattccaatagttca
KanR
```

pJ2039_N2only (7295 bp) (from 4923-6206 bp)

```
gagaaatcaccatgagtgacgactgaatccggtgagaatggcaaaagtttatgcatttctttccagacttgttcaacaggccagccattacgctcgtcatcaaaatc
ctctttagtggtactcactgctgacttaggccactcttaccgttttcaaatacgtaaagaaaggtctgaacaagttgtccggtcggtaatgcgagcagtagttttag
                                                KanR

actcgcatcaaccaaaccgttattcattcgtgattgcgcctgagcgagacgaaatacgcggtcgctgttaaaaggacaattacaaacaggaatcgaatgcaaccggc
tgagcgtagttggtttggcaataagtaagcactaacgcggactcgctctgctttatgcgccagcgacaattttcctgttaatgtttgtccttagcttacgttggccg
                                                KanR

gcaggaacactgccagcgcatcaacaatattttcacctgaatcaggatattcttctaatacctggaatgctgttttcccggggatcgcagtggtgagtaaccatgca
cgtccttgtgacggtcgcgtagttgttataaaagtggacttagtcctataagaagattatggaccttacgacaaaagggccccctagcgtcaccactcattggtacgt
                                                KanR

tcatcaggagtacggataaaatgcttgatggtcggaagaggcataaattccgtcagccagtttagtctgaccatctcatctgtaacatcattggcaacgctaccttt
agtagtcctcatgcctattttacgaactaccagccttctccgtatttaaggcagtcggtcaaatcagactggtagagtagacattgtagtaaccgttgcgatggaaa
                                                KanR

gccatgtttcagaaacaactctggcgcatcgggcttcccatacaatcgatagattgtcgcacctgattgcccgacattatcgcgagcccatttatacccatataaat
cggtacaaagtctttgttgagaccgcgtagcccgaagggtatgttagctatctaacagcgtggactaacgggctgtaatagcgctcgggtaaatatgggtatattta
                                                KanR

cagcatccatgttggaatttaatcgcggcctagagcaagacgtttcccgttgaatatggctcatactcttcctttttcaatattattgaagcatttatcagggttat
gtcgtaggtacaaccttaaattagcgccggatctcgttctgcaaagggcaacttataccgagtatgagaaggaaaaagttataataacttcgtaaatagtcccaata
                                  KanR

tgtctcatgagcggatacatatttgaatgtatttagaaaaataaacaaataggcatgctagcgcagaaacgtcctagaagatgccaggaggatacttagcagagaga
acagagtactcgcctatgtataaacttacataaatctttttatttgtttatccgtacgatcgcgtctttgcaggatcttctacggtcctcctatgaatcgtctctct
                                                                                ColA ori

caataaggccggagcgaagccgtttttccataggctccgcccccctgacgaacatcacgaaatctgacgctcaaatcagtggtggcgaaacccgacaggactataaa
gttattccggcctcgcttcggcaaaaaggtatccgaggcggggggactgcttgtagtgctttagactgcgagtttagtcaccaccgctttgggctgtcctgatattt
                                                ColA ori

gataccaggcgtttccccctgatggctccctcttgcgctctcctgttcccgtcctgcggcgtccgtgttgtggtggaggctttacccaaatcaccacgtcccgttcc
ctatggtccgcaaaggggggactaccgagggagaacgcgagaggacaagggcaggacgccgcaggcacaacaccacctccgaaatgggtttagtggtgcagggcaagg
                                                ColA ori

gtgtagacagttcgctccaagctgggctgtgtgcaagaacccccccgttcagcccgactgctgcgccttatccggtaactatcatcttgagtccaacccggaaagaca
cacatctgtcaagcgaggttcgacccgacacacgttcttgggggggcaagtcgggctgacgacgcggaataggccattgatagtagaactcaggttgggcctttctgt
                                                ColA ori

cgacaaaacgccactggcagcagccattggtaactgagaattagtggatttagatatcgagagtcttgaagtggtggcctaacagaggctacactgaaggacagta
gctgttttgcggtgaccgtcgtcggtaaccattgactcttaatcacctaaatctatagctctcagaacttcaccaccggattgtctccgatgtgactttcctgtcat
                                                ColA ori

tttggtatctgcgctccactaaagccagttaccaggttaagcagttccccaactgacttaaccttcgatcaaaccgcctccccaggcggttttttcgtttacagagc
aaaccatagacgcgaggtgatttcggtcaatggtccaattcgtcaaggggttgactgaattggaagctagtttggcggaggggtccgccaaaaaagcaaatgtctcg
                                                ColA ori
```

pJ2039_N2only (7295 bp) (from 6207-7295 bp)

aggagattacgacgatcgtaaaaggatctcaagaagatcctttacggattcccgacaccatcactctagatttcagtgcaatttatctcttcaaatgtagcacctga
tcctctaatgctgctagcattttcctagagttcttctaggaaatgcctaagggctgtggtagtgagatctaaagtcacgttaaatagagaagtttacatcgtggact
ColA ori

agtcagccccatacgatataagttgtaattctcatgttagtcatgccccgcgcccaccggaaggagctgactgggttgCTCCTAgGGTCTGATTCGTTACCAATTAT
tcagtcggggtatgctatattcaacattaagagtacaatcagtacggggcgcgggtggccttcctcgactgacccaacGAGGATcCCAGACTAAGCAATGGTTAATA

GACAACTTGACGGCTACATCATTCACTTTTTCTTCACAACCGGCACGGAACTCGCTCGGGCTGGCCCCGGTGCATTTTTTAAATACCCGCGAGAAATAGAGTTGATC
CTGTTGAACTGCCGATGTAGTAAGTGAAAAAGAAGTGTTGGCCGTGCCTTGAGCGAGCCCGACCGGGGCCACGTAAAAAATTTATGGGCGCTCTTTATCTCAACTAG
araC

GTCAAAACCAACATTGCGACCGACGGTGGCGATAGGCATCCGGGTGGTGCTCAAAAGCAGCTTCGCCTGGCTGATACGTTGGTCCTCGCGCCAGCTTAAGACGCTAA
CAGTTTTGGTTGTAACGCTGGCTGCCACCGCTATCCGTAGGCCCACCACGAGTTTTCGTCGAAGCGGACCGACTATGCAACCAGGAGCGCGGTCGAATTCTGCGATT
araC

TCCCTAACTGCTGGCGGAAAAGATGTGACAGACGCGACGGCGACAAGCAAACATGCTGTGCGACGCTGGCGATATCAAAATTGCTGTCTGCCAGGTGATCGCTGATG
AGGGATTGACGACCGCCTTTTCTACACTGTCTGCGCTGCCGCTGTTCGTTTGTACGACACGCTGCGACCGCTATAGTTTTAACGACAGACGGTCCACTAGCGACTAC
araC

TACTGACAAGCCTCGCGTACCCGATTATCCATCGGTGGATGGAGCGACTCGTTAATCGCTTCCATGCGCCGCAGTAACAATTGCTCAAGCAGATTTATCGCCAGCAG
ATGACTGTTCGGAGCGCATGGGCTAATAGGTAGCCACCTACCTCGCTGAGCAATTAGCGAAGGTACGCGGCGTCATTGTTAACGAGTTCGTCTAAATAGCGGTCGTC
araC

CTCCGAATAGCGCCCTTCCCCTTGCCCGGCGTTAATGATTTGCCCAAACAGGTCGCTGAAATGCGGCTGGTGCGCTTCATCCGGGCGAAAGAACCCCGTATTGGCAA
GAGGCTTATCGCGGGAAGGGGAACGGGCCGCAATTACTAAACGGGTTTGTCCAGCGACTTTACGCCGACCACGCGAAGTAGGCCCGCTTTCTTGGGGCATAACCGTT
araC

ATATTGACGGCCAGTTAAGCCATTCATGCCAGTAGGCGCGCGGACGAAAGTAAACCCACTGGTGATACCATTCGCGAGCCTCCGGATGACGACCGTAGTGATGAATC
TATAACTGCCGGTCAATTCGGTAAGTACGGTCATCCGCGCGCCTGCTTTCATTTGGGTGACCACTATGGTAAGCGCTCGGAGGCCTACTGCTGGCATCACTACTTAG
araC

TCTCCTGGCGGGAACAGCAAAATATCACCCGGTCGGCAAACAAATTCTCGTCCCTGATTTTTCACCACCCCCTGACCGCGAATGGTGAGATTGAGAATATAACCTTT
AGAGGACCGCCCTTGTCGTTTTATAGTGGGCCAGCCGTTTGTTTAAGAGCAGGGACTAAAAAGTGGTGGGGGACTGGCGCTTACCACTCTAACTCTTATATTGGAAA
araC

CATTCCCAGCGGTCGGTCGATAAAAAAATCGAGATAACCGTTGGCCTCAATCGGCGTTAAACCCGCCACCAGATGGGCATTAAACGAGTATCCCGGCAGCAGGGGAT
GTAAGGGTCGCCAGCCAGCTATTTTTTTAGCTCTATTGGCAACCGGAGTTAGCCGCAATTTGGGCGGTGGTCTACCCGTAATTTGCTCATAGGGCCGTCGTCCCCTA
araC

CATTTTGCGCTTCAGCCAT
GTAAAACGCGAAGTCGGTA
araC

(from 1-1177 bp)

pJ2040 (7459 bp)

ACTTTTCATACTCCCGCCATTCAGAGAAGAAACCAATTGTCCATATTGCATCAGACATTGCCGTCACTGCGTCTTTTACTGGCTCTTCTCGCTAACCAAACCGGTAA
TGAAAAGTATGAGGGCGGTAAGTCTCTTCTTTGGTTAACAGGTATAACGTAGTCTGTAACGGCAGTGACGCAGAAAATGACCGAGAAGAGCGATTGGTTTGGCCATT

CCCCGCTTATTAAAAGCATTCTGTAACAAAGCGGGACCAAAGCCATGACAAAAACGCGTAACAAAAGTGTCTATAATCACGGCAGAAAAGTCCACATTGATTATTTG
GGGGCGAATAATTTTCGTAAGACATTGTTTCGCCCTGGTTTCGGTACTGTTTTTGCGCATTGTTTTCACAGATATTAGTGCCGTCTTTTCAGGTGTAACTAATAAAC

EcoRI

CACGGCGTCACACTTTGCTATGCCATAGCATTTTTATCCATAAGATTAGCGGtTCCTACCTGACGCTTTTTATCGCAACTCTCTACTGTTTCTCCATACCGAATTCA
GTGCCGCAGTGTGAAACGATACGGTATCGTAAAAATAGGTATTCTAATCGCCaAGGATGGACTGCGAAAAATAGCGTTGAGAGATGACAAAGAGGTATGGCTTAAGT

P(BAD) promoter

SacI

TAGGATAGATTCTGGAAACTTTACCGTCCGAGCTCCAGCCTGCGGTCCGGGTTCACTGCCGTATAGGCAGGACACATCTTAGAGTATGTAGTTTcAGAGCTATGCTG
ATCCTATCTAAGACCTTTGAAATGGCAGGCTCGAGGTCGGACGCCAGGCCCAAGTGACGGCATATCCGTCCTGTGTAGAATCTCATACATCAAAgTCTCGATACGAC

Linker_14 Prefix Csy4 site sgRNA-4

XhoI

GAAACAGCATAGCAAGTTgAAATAAGGCTAGTCCGTTATCAACTTGAAAAAGTGGCACCGAGTCGGTGCGTTCACTGCCGTATAGGCAGTCGCTGGGACGCCCGCTC
CTTTGTCGTATCGTTCAAcTTTATTCCGATCAGGCAATAGTTGAACTTTTTCACCGTGGCTCAGCCACGCAAGTGACGGCATATCCGTCAGCGACCCTGCGGGCGAG

sgRNA-4 Csy4 site Suffix

GAGCAATAAACAGTTGATAGGGCTTCTCCGTTACAGCCTGCGGTCCGGGTTCACTGCCGTATAGGCAGTAATTTTGTTTAACTTTAAGAAGGAGATATACATATGGT
CTCGTTATTTGTCAACTATCCCGAAGAGGCAATGTCGGACGCCAGGCCCAAGTGACGGCATATCCGTCATTAAAACAAATTGAAATTCTTCCTCTATATGTATACCA

Linker_0 Prefix Csy4 site RBS

TTCGGTTATCAAACCAGAGATGAAAATGCGTTACTATATGGATGGTTCAGTAAATGGTCACGAATTTACTATTGAGGGCGAGGGTACGGGACGCCCATACGAGGGGC
AAGCCAATAGTTTGGTCTCTACTTTTACGCAATGATATACCTACCAAGTCATTTACCAGTGCTTAAATGATAACTCCCGCTCCCATGCCCTGCGGGTATGCTCCCCG

mKO2

ACCAGGAAATGACTTTACGCGTCACAATGGCTGAAGGCGGGCCTATGCCGTTTGCGTTCGATCTTGTTAGTCATGTCTTTTGTTACGGTCACCGTGTATTTACTAAA
TGGTCCTTTACTGAAATGCGCAGTGTTACCGACTTCCGCCCGGATACGGCAAACGCAAGCTAGAACAATCAGTACAGAAAACAATGCCAGTGGCACATAAATGATTT

mKO2

TACCCCGAGGAAATTCCAGACTATTTCAAACAAGCCTTCCCGGAAGGTTTGTCTTGGGAGCGCAGTTTAGAGTTTGAAGACGGTGGCTCGGCCAGCGTGTCAGCTCA
ATGGGGCTCCTTTAAGGTCTGATAAAGTTTGTTCGGAAGGGCCTTCCAAACAGAACCCTCGCGTCAAATCTCAAACTTCTGCCACCGAGCCGGTCGCACAGTCGAGT

mKO2

TATTAGTCTTCGCGGCAATACATTTTATCACAAGTCAAAGTTCACCGGCGTGAACTTCCCCGCAGACGGCCCAATCATGCAGAATCAAAGTGTTGATTGGGAACCGT
ATAATCAGAAGCGCCGTTATGTAAAATAGTGTTCAGTTTCAAGTGGCCGCACTTGAAGGGGCGTCTGCCGGGTTAGTACGTCTTAGTTTCACAACTAACCCTTGGCA

mKO2

CCACAGAGAAGATTACAGCTTCCGATGGAGTCTTAAAGGGCGATGTAACCATGTACTTAAAATTAGAAGGGGGAGGGAACCATAAATGTCAGATGAAGACTACCTAT
GGTGTCTCTTCTAATGTCGAAGGCTACCTCAGAATTTCCCGCTACATTGGTACATGAATTTTAATCTTCCCCCTCCCTTGGTATTTACAGTCTACTTCTGATGGATA

mKO2

pJ2040 (7459 bp) (from 1178-2354 bp)

```
AAGGCCGCAAAAGAGATTCTTGAAATGCCCGGAGACCACTACATTGGGCATCGTTTGGTCCGTAAGACAGAAGGAAATATTACTGAACAGGTCGAAGACGCTGTGGC
TTCCGGCGTTTTCTCTAAGAACTTTACGGGCCTCTGGTGATGTAACCCGTAGCAAACCAGGCATTCTGTCTTCCTTTATAATGACTTGTCCAGCTTCTGCGACACCG
mKO2
```

```
ACACAGCATGTCCCGCCGTAATACTGACGCCATCACAATCCACAGCATCCTGGATTGGATTGAAGACAACTTGGAGTCGCCGTTGAGTTTAGAAAAAGTTAGTGAAC
TGTGTCGTACAGGGCGGCATTATGACTGCGGTAGTGTTAGGTGTCGTAGGACCTAACCTAACTTCTGTTGAACCTCAGCGGCAACTCAAATCTTTTTCAATCACTTG
MarA
```

```
GTAGTGGTTACTCAAAGTGGCACCTTCAGCGCATGTTTAAGAAGGAAACGGGTCATTCATTGGGTCAATATATTCGTTCTCGCAAGATGACTGAAATTGCCCAGAAA
CATCACCAATGAGTTTCACCGTGGAAGTCGCGTACAAATTCTTCCTTTGCCCAGTAAGTAACCCAGTTATATAAGCAAGAGCGTTCTACTGACTTTAACGGGTCTTT
MarA
```

```
TTGAAAGAGTCTAATGAACCTATTTTGTACCTGGCGGAGCGTTACGGCTTTGAAAGTCAGCAAACCCTTACACGTACCTTCAAGAATTACTTTGACGTTCCACCACA
AACTTTCTCAGATTACTTGGATAAAACATGGACCGCCTCGCAATGCCGAAACTTTCAGTCGTTTGGGAATGTGCATGGAAGTTCTTAATGAAACTGCAAGGTGGTGT
MarA
```

```
NcoI
CAAATATCGTATGACCAACATGCAGGGTGAGTCACGTTTTTTGCATCCGTTGAATCATTACAATTCCTAATAATCGCTGGGACGCCCGCCATGGTTCAGCCAAAAAA
GTTTATAGCATACTGGTTGTACGTCCCACTCAGTGCAAAAAACGTAGGCAACTTAGTAATGTTAAGGATTATTAGCGACCCTGCGGGCGGTACCAAGTCGGTTTTTT
MarA  Suffix  ECK...or
```

```
CTTAAGACCGCCGGTCTTGTCCACTACCTTGCAGTAATGCGGTGGACAGGATCGGCGGTTTTCTTTTCTCTTCTCAATTCTTCTGACCTGTAACGAATAATAGATAG
GAATTCTGGCGGCCAGAACAGGTGATGGAACGTCATTACGCCACCTGTCCTAGCCGCCAAAAGAAAAGAGAAGAGTTAAGAAGACTGGACATTGCTTATTATCTATC
ECK120029600 Terminator  Spacer 1
```

```
TAAAGTAGTCTCCGATTGAGTTTTCTCTGCCGAGTCCCACCCAGTTCTGTGATTTCAGTAAGTTGGTAATTGATACACTGTTGCGAGAACTGCTGCCTGGTAGTAGA
ATTTCATCAGAGGCTAACTCAAAAGAGACGGCTCAGGGTGGGTCAAGACACTAAAGTCATTCAACCATTAACTATGTGACAACGCTCTTGACGACGGACCATCATCT
Spacer 1
```

```
HindIII
TAGGTTGTTATTGAGTAAGAAGGTAAAGTGAACGAAATCCCTGAAACTGAGACTGTAGAAAATAAGCTTCAGCCTGCGGTCCGGTTGACAGCTAGCTCAGTCCTAGG
ATCCAACAATAACTCATTCTTCCATTTCACTTGCTTTAGGGACTTTGACTCTGACATCTTTTATTCGAAGTCGGACGCCAGGCCAACTGTCGATCGAGTCAGGATCC
Spacer 1  Prefix  P(BBa_J23102)
```

```
TACTGTGCTAGCTCGCTGGGACGCCCGGGGACTACACTTACGAAACTATTGATTGCTCAGCCTGCGGTCCGGccaTACATACTCTAAGATGTGTCTCGCTGGGACGC
ATGACACGATCGAGCGACCCTGCGGGCCCCTGATGTGAATGCTTTGATAACTAACGAGTCGGACGCCAGGCCggtATGTATGAGATTCTACACAGAGCGACCCTGCG
P(B...2)  Suffix  Linker_1  Prefix  bs-4  Suffix
```

```
BamHI
CCGGGATCCAAGAGATTTCTACACGATTGAGCACTGTCTCAGCCTGCGGTCCGGGTTCACTGCCGTATAGGCAGTAATTTTGTTTAACTTTAAGAAGGAGATATACA
GGCCCTAGGTTCTCTAAAGATGTGCTAACTCGTGACAGAGTCGGACGCCAGGCCCAAGTGACGGCATATCCGTCATTAAAACAAATTGAAATTCTTCCTCTATATGT
Linker_10  Prefix  Csy4 site  RBS
```

```
TATGCGTAAAGGCGAAGAACTGTTTACCGGTGTGGTTCCGATTCTGGTGGAACTGGACGGCGATGTTAATGGTCATAAATTCAGTGTTCGCGGCGAAGGTGAAGGCG
ATACGCATTTCCGCTTCTTGACAAATGGCCACACCAAGGCTAAGACCACCTTGACCTGCCGCTACAATTACCAGTATTTAAGTCACAAGCGCCGCTTCCACTTCCGC
sfGFP
```

pJ2040 (7459 bp) (from 2355-3531 bp)

ATGCGACGAACGGCAAACTGACCCTGAAATTTATCTGCACCACGGGTAAACTGCCGGTCCCGTGGCCGACGCTGGTGACCACGCTGACCTATGGCGTTCAATGTTTT
TACGCTGCTTGCCGTTTGACTGGGACTTTAAATAGACGTGGTGCCCATTTGACGGCCAGGGCACCGGCTGCGACCACTGGTGCGACTGGATACCGCAAGTTACAAAA
sfGFP

GCGCGTTACCCGGATCACATGAAACAGCACGACTTTTTCAAATCGGCCATGCCGGAAGGCTATGTGCAGGAACGTACGATTAGCTTTAAAGACGATGGTACGTATAA
CGCGCAATGGGCCTAGTGTACTTTGTCGTGCTGAAAAAGTTTAGCCGGTACGGCCTTCCGATACACGTCCTTGCATGCTAATCGAAATTTCTGCTACCATGCATATT
sfGFP

AACCCGCGCGGAAGTGAAATTCGAAGGCGATACCCTGGTTAACCGTATCGAACTGAAAGGTATCGATTTCAAAGAAGACGGCAATATTCTGGGTCATAAACTGGAAT
TTGGGCGCGCCTTCACTTTAAGCTTCCGCTATGGGACCAATTGGCATAGCTTGACTTTCCATAGCTAAAGTTTCTTCTGCCGTTATAAGACCCAGTATTTGACCTTA
sfGFP

ATAACTTCAATTCCCACAACGTGTACATCACCGCGGATAAACAGAAAAACGGCATTAAAGCCAATTTCAAAATCCGCCATAATGTGGAAGATGGTAGCGTTCAGCTG
TATTGAAGTTAAGGGTGTTGCACATGTAGTGGCGCCTATTTGTCTTTTTGCCGTAATTTCGGTTAAAGTTTTAGGCGGTATTACACCTTCTACCATCGCAAGTCGAC
sfGFP

GCCGACCACTATCAGCAAAACACGCCGATTGGTGATGGCCCGGTCCTGCTGCCGGACAATCACTACCTGAGTACCCAGTCCGTGCTGTCAAAAGATCCGAACGAAAA
CGGCTGGTGATAGTCGTTTTGTGCGGCTAACCACTACCGGGCCAGGACGACGGCCTGTTAGTGATGGACTCATGGGTCAGGCACGACAGTTTTCTAGGCTTGCTTTT
sfGFP

ACGTGACCACATGGTCCTGCTGGAATTTGTGACGGCTGCGGGTATCACCCACGGCATGGACGAACTGTATAAAATGTCCCGCCGTAATACTGACGCCATCACAATCC
TGCACTGGTGTACCAGGACGACCTTAAACACTGCCGACGCCCATAGTGGGTGCCGTACCTGCTTGACATATTTTACAGGGCGGCATTATGACTGCGGTAGTGTTAGG
sfGFP MarAn20

SbfI KpnI
ACAGCATCCTGGATTGGATTGAAGACTAATAATCGCTGGGACGCCCGCCTGCAGGCTCGGTACCAAATTCCAGAAAAGAGGCCTCCCGAAAGGGGGGCCTTTTTTCG
TGTCGTAGGACCTAACCTAACTTCTGATTATTAGCGACCCTGCGGGCGGACGTCCGAGCCATGGTTTAAGGTCTTTTCTCCGGAGGGCTTTCCCCCCGGAAAAAAGC
MarAn20 Suffix L3S2P21 Terminator

TTTTGGTCCTAATAGATAAAGGATAGGTCTGGTAGTGTTGTTCGTTCTCGCAGGTAAATCAATAATACTCAGCAGTTCCGTAGACTTTTCAGTGGGACAGGGTAGCG
AAAACCAGGATTATCTATTTCCTATCCAGACCATCACAACAAGCAAGAGCGTCCATTTAGTTATTATGAGTCGTCAAGGCATCTGAAAAGTCACCCTGTCCCATCGC
Spacer 2.5

SalI
ATAACAGATAGATTGTAATAAGACACAGTAGGTGCTCGTAGTTGCGTGAAGAGAACCGCTCAGGAAATCCAGTCAGAAGTATTGGTAATCGTTGAAAACTCAGTCGA
TATTGTCTATCTAACATTATTCTGTGTCATCCACGAGCATCAACGCACTTCTCTTGGCGAGTCCTTTAGGTCAGTCTTCATAACCATTAGCAACTTTTGAGTCAGCT
Spacer 2.5

AatII
CGCACTTACTGAAGACGTCCTATTACACTCGTCGTTGGAAACTGAAGATCAGCCTGCGGTCCGGGTTCACTGCCGTATAGGCAGTAATTTTGTTTAACTTTAAGAAG
GCGTGAATGACTTCTGCAGGATAATGTGAGCAGCAACCTTTGACTTCTAGTCGGACGCCAGGCCCAAGTGACGGCATATCCGTCATTAAAACAAATTGAAATTCTTC
Spa... 5 Linker_11 Prefix Csy4 site RBS

GAGATATACATATGAATCAGTCATTCATCTCGGACATCTTATATGCCGACATCGAATCGAAGGCTAAGGAACTTACAGTCAATTCCAACAATACTGTCCAGCCGGTC
CTCTATATGTATACTTAGTCAGTAAGTAGAGCCTGTAGAATATACGGCTGTAGCTTAGCTTCCGATTCCTTGAATGTCAGTTAAGGTTGTTATGACAGGTCGGCCAG
RBS RepA70

pJ2040 (7459 bp) (from 3532-4815 bp)

```
GCGCTTATGCGCTTAGGAGTTTTCGTTCCCAAACCTTCCAAGAGCAAAGGAGAAAGTAAGGAAATTGACGCCACCAAAGCCTTCTCTCAACTGGAGATTGCTAAAGC
CGCGAATACGCGAATCCTCAAAAGCAAGGGTTTGGAAGGTTCTCGTTTCCTCTTTCATTCCTTTAACTGCGGTGGTTTCGGAAGAGAGTTGACCTCTAACGATTTCG
RepA70

AGAGGGCatggttagtaaaggagaagaaaataacatggcaCTGATTAAGGAGAACATGCACATGAAGCTGTACATGGAGGGCACCGTGAACAACCACCACTTCAAGT
TCTCCCGtaccaatcatttcctcttcttttattgtaccgtGACTAATTCCTCTTGTACGTGTACTTCGACATGTACCTCCCGTGGCACTTGTTGGTGGTGAAGTTCA
mKate2

GCACATCCGAGGGCGAAGGCAAGCCCTACGAGGGCACCCAGACCATGAGAATCAAGgccGTCGAGGGCGGCCCTCTCCCCTTCGCCTTCGACATCCTGGCTACCAGC
CGTGTAGGCTCCCGCTTCCGTTCGGGATGCTCCCGTGGGTCTGGTACTCTTAGTTCcggCAGCTCCCGCCGGGAGAGGGGAAGCGGAAGCTGTAGGACCGATGGTCG
mKate2

TTCATGTACGGCAGCAAAACCTTCATCAACCACACCCAGGGCATCCCCGACTTCTTTAAGCAGTCCTTCCCTGAGGGCTTCACATGGGAGAGAGTCACCACATACGA
AAGTACATGCCGTCGTTTTGGAAGTAGTTGGTGTGGGTCCCGTAGGGGCTGAAGAAATTCGTCAGGAAGGGACTCCCGAAGTGTACCCTCTCTCAGTGGTGTATGCT
mKate2

AGACGGGGGCGTGCTGACCGCTACCCAGGACACCAGCCTCCAGGACGGCTGCCTCATCTACAACGTCAAGATCAGAGGGGTGAACTTCCCATCCAACGGCCCTGTGA
TCTGCCCCCGCACGACTGGCGATGGGTCCTGTGGTCGGAGGTCCTGCCGACGGAGTAGATGTTGCAGTTCTAGTCTCCCCACTTGAAGGGTAGGTTGCCGGGACACT
mKate2

TGCAGAAGAAAACACTCGGCTGGGAGGCCTCCACCGAGaccCTGTACCCCGCTGACGGCGGCCTGGAAGGCAGAgcCGACATGGCCCTGAAGCTCGTGGGCGGGGGC
ACGTCTTCTTTTGTGAGCCGACCCTCCGGAGGTGGCTCtggGACATGGGGCGACTGCCGCCGGACCTTCCGTCTcgGCTGTACCGGGACTTCGAGCACCCGCCCCCG
mKate2

CACCTGATCTGCAACTTGAAGACCACATACAGATCCAAGAAACCCGCTAAGAACCTCAAGATGCCCGGCGTCTACTATGTGGACAGAAGACTGGAAAGAATCAAGGA
GTGGACTAGACGTTGAACTTCTGGTGTATGTCTAGGTTCTTTGGGCGATTCTTGGAGTTCTACGGGCCGCAGATGATACACCTGTCTTCTGACCTTTCTTAGTTCCT
mKate2

GGCCGACAAAGAGACCTACGTCGAGCAGCACGAGGTGGCTGTGGCCAGATACTGCGACCTCCCTAGCAAACTGGGGCACAgAgtctaATAATCGCTGGGACGCCCGG
CCGGCTGTTTCTCTGGATGCAGCTCGTCGTGCTCCACCGACACCGGTCTATGACGCTGGAGGGATCGTTTGACCCCGTGTcTcagatTATTAGCGACCCTGCGGGCC
mKate2    Suffix

NotI
CGGCCGCggaaacacagAAAAAAGCCCGCACCTGACAGTGCGGGCTTTTTTTTTcgaccaaaggTAGCGAACGACGAGTCACTGTTGAGGATAAATACTTTCTCTAC
GCCGGCGcctttgtgtcTTTTTTCGGGCGTGGACTGTCACGCCCGAAAAAAAAAgctggtttccATCGCTTGCTGCTCAGTGACAACTCCTATTTATGAAAGAGATG
ECK120033737 Terminator    Linker_8

KasI    BbvCI    AscI
TAGGCGCCTGTTACACAGGTCCTCAGCGGCGCGCCTTTGTCGGTGAACGCTCTCCTGAGTAGGACAAATCCGCCGGGAGCGGATTTGAACGTTGTGAAGCAACGGCC
ATCCGCGGACAATGTGTCCAGGAGTCGCCGCGCGGAAACAGCCACTTGCGAGAGGACTCATCCTGTTTAGGCGGCCCTCGCCTAAACTTGCAACACTTCGTTGCCGG
Spa...15

CGGAGGGTGGCGGGCAGGACGCCCGCCATAAACTGCCAGGCATCAAACTAAGCAGAAGGCCATCCTGACGGATGGCCTTTTTGCGTTTCAGATCTACCGGTaaacca
GCCTCCCACCGCCCGTCCTGCGGGCGGTATTTGACGGTCCGTAGTTTGATTCGTCTTCCGGTAGGACTGCCTACCGGAAAAACGCAAAGTCTAGATGGCCAtttggt

gcaatagacataagcggctatttaacgaccctgccctgaaccgacgacaagctgacgaccgggtctccgcaagtggcacttttcggggaaatgtgcgcggaacccct
cgttatctgtattcgccgataaattgctgggacgggacttggctgctgttcgactgctggcccagaggcgttcaccgtgaaaagcccctttacacgcgccttggga
```

pJ2040 (7459 bp) (from 4816-6099 bp)

atttgtttatttttctaaatacattcaaatatgtatccgctcatgaattaattcttagaaaaactcatcgagcatcaaatgaaactgcaatttattcatatcaggat
taaacaaataaaaagatttatgtaagtttatacataggcgagtacttaattaagaatctttttgagtagctcgtagtttactttgacgttaaataagtatagtccta
KanR

tatcaataccatatttttgaaaaagccgtttctgtaatgaaggagaaaactcaccgaggcagttccataggatggcaagatcctggtatcggtctgcgattccgact
atagttatggtataaaaactttttcggcaaagacattacttcctcttttgagtggctccgtcaaggtatcctaccgttctaggaccatagccagacgctaaggctga
KanR

cgtccaacatcaatacaacctattaatttcccctcgtcaaaaataaggttatcaagtgagaaatcaccatgagtgacgactgaatccggtgagaatggcaaaagttt
gcaggttgtagttatgttggataattaaaggggagcagtttttattccaatagttcactctttagtggtactcactgctgacttaggccactcttaccgttttcaaa
KanR

atgcatttctttccagacttgttcaacaggccagccattacgctcgtcatcaaaatcactcgcatcaaccaaaccgttattcattcgtgattgcgcctgagcgagac
tacgtaaagaaaggtctgaacaagttgtccggtcggtaatgcgagcagtagttttagtgagcgtagttggtttggcaataagtaagcactaacgcggactcgctctg
KanR

gaaatacgcggtcgctgttaaaaggacaattacaaacaggaatcgaatgcaaccggcgcaggaacactgccagcgcatcaacaatattttcacctgaatcaggatat
ctttatgcgccagcgacaattttcctgttaatgtttgtccttagcttacgttggccgcgtccttgtgacggtcgcgtagttgttataaaagtggacttagtcctata
KanR

tcttctaatacctggaatgctgttttcccggggatcgcagtggtgagtaaccatgcatcatcaggagtacggataaaatgcttgatggtcggaagaggcataaattc
agaagattatggaccttacgacaaaagggcccctagcgtcaccactcattggtacgtagtagtcctcatgcctattttacgaactaccagccttctccgtatttaag
KanR

cgtcagccagtttagtctgaccatctcatctgtaacatcattggcaacgctacctttgccatgtttcagaaacaactctggcgcatcgggcttcccatacaatcgat
gcagtcggtcaaatcagactggtagagtagacattgtagtaaccgttgcgatggaaacggtacaaagtctttgttgagaccgcgtagcccgaagggtatgttagcta
KanR

agattgtcgcacctgattgcccgacattatcgcgagcccatttatacccatataaatcagcatccatgttggaatttaatcgcggcctagagcaagacgtttcccgt
tctaacagcgtggactaacgggctgtaatagcgctcgggtaaatatgggtatatttagtcgtaggtacaaccttaaattagcgccggatctcgttctgcaaagggca
KanR

tgaatatggctcatactcttcctttttcaatattattgaagcatttatcagggttattgtctcatgagcggatacatatttgaatgtatttagaaaaataaacaaat
acttataccgagtatgagaaggaaaaagttataataacttcgtaaatagtcccaataacagagtactcgcctatgtataaacttacataaatctttttatttgttta
KanR

aggcatgctagcgcagaaacgtcctagaagatgccaggaggatacttagcagagagacaataaggccggagcgaagccgtttttccataggctccgcccccctgacg
tccgtacgatcgcgtctttgcaggatcttctacggtcctcctatgaatcgtctctctgttattccggcctcgcttcggcaaaaaggtatccgaggcggggggactgc
ColA ori

aacatcacgaaatctgacgctcaaatcagtggtggcgaaacccgacaggactataaagataccaggcgtttccccctgatggctccctcttgcgctctcctgttccc
ttgtagtgctttagactgcgagtttagtcaccaccgctttgggctgtcctgatatttctatggtccgcaaagggggactaccgagggagaacgcgagaggacaaggg
ColA ori

gtcctgcggcgtccgtgttgtggtggaggctttacccaaatcaccacgtcccgttccgtgtagacagttcgctccaagctgggctgtgtgcaagaaccccccgttca
caggacgccgcaggcacaacaccacctccgaaatgggtttagtggtgcagggcaaggcacatctgtcaagcgaggttcgacccgacacacgttcttggggggcaagt
ColA ori

pJ2040 (7459 bp) (from 6100-7383 bp)

gcccgactgctgcgccttatccggtaactatcatcttgagtccaacccggaaagacacgacaaaacgccactggcagcagccattggtaactgagaattagtggatt
cgggctgacgacgcggaataggccattgatagtagaactcaggttgggcctttctgtgctgttttgcggtgaccgtcgtcggtaaccattgactcttaatcacctaa

ColA ori

tagatatcgagagtcttgaagtggtggcctaacagaggctacactgaaaggacagtatttggtatctgcgctccactaaagccagttaccaggttaagcagttcccc
atctatagctctcagaacttcaccaccggattgtctccgatgtgactttcctgtcataaaccatagacgcgaggtgatttcggtcaatggtccaattcgtcaagggg

ColA ori

aactgacttaaccttcgatcaaaccgcctccccaggcggttttttcgtttacagagcaggagattacgacgatcgtaaaaggatctcaagaagatcctttacggatt
ttgactgaattggaagctagtttggcggaggggtccgccaaaaaagcaaatgtctcgtcctctaatgctgctagcattttcctagagttcttctaggaaatgcctaa

ColA ori

cccgacaccatcactctagatttcagtgcaatttatctcttcaaatgtagcacctgaagtcagccccatacgatataagttgtaattctcatgttagtcatgccccg
gggctgtggtagtgagatctaaagtcacgttaaatagagaagtttacatcgtggacttcagtcggggtatgctatattcaacattaagagtacaatcagtacggggc

Co...i

cgcccaccggaaggagctgactgggttgCTCCTAgGGTCTGATTCGTTACCAATTATGACAACTTGACGGCTACATCATTCACTTTTTCTTCACAACCGGCACGGAA
gcgggtggccttcctcgactgacccaacGAGGATcCCAGACTAAGCAATGGTTAATACTGTTGAACTGCCGATGTAGTAAGTGAAAAAGAAGTGTTGGCCGTGCCTT

araC

CTCGCTCGGGCTGGCCCCGGTGCATTTTTTAAATACCCGCGAGAAATAGAGTTGATCGTCAAAACCAACATTGCGACCGACGGTGGCGATAGGCATCCGGGTGGTGC
GAGCGAGCCCGACCGGGGCCACGTAAAAAATTTATGGGCGCTCTTTATCTCAACTAGCAGTTTTGGTTGTAACGCTGGCTGCCACCGCTATCCGTAGGCCCACCACG

araC

TCAAAAGCAGCTTCGCCTGGCTGATACGTTGGTCCTCGCGCCAGCTTAAGACGCTAATCCCTAACTGCTGGCGGAAAAGATGTGACAGACGCGACGGCGACAAGCAA
AGTTTTCGTCGAAGCGGACCGACTATGCAACCAGGAGCGCGGTCGAATTCTGCGATTAGGGATTGACGACCGCCTTTTCTACACTGTCTGCGCTGCCGCTGTTCGTT

araC

ACATGCTGTGCGACGCTGGCGATATCAAAATTGCTGTCTGCCAGGTGATCGCTGATGTACTGACAAGCCTCGCGTACCCGATTATCCATCGGTGGATGGAGCGACTC
TGTACGACACGCTGCGACCGCTATAGTTTTAACGACAGACGGTCCACTAGCGACTACATGACTGTTCGGAGCGCATGGGCTAATAGGTAGCCACCTACCTCGCTGAG

araC

GTTAATCGCTTCCATGCGCCGCAGTAACAATTGCTCAAGCAGATTTATCGCCAGCAGCTCCGAATAGCGCCCTTCCCCTTGCCCGGCGTTAATGATTTGCCCAAACA
CAATTAGCGAAGGTACGCGGCGTCATTGTTAACGAGTTCGTCTAAATAGCGGTCGTCGAGGCTTATCGCGGGAAGGGGAACGGGCCGCAATTACTAAACGGGTTTGT

araC

GGTCGCTGAAATGCGGCTGGTGCGCTTCATCCGGGCGAAAGAACCCCGTATTGGCAAATATTGACGGCCAGTTAAGCCATTCATGCCAGTAGGCGCGCGGACGAAAG
CCAGCGACTTTACGCCGACCACGCGAAGTAGGCCCGCTTTCTTGGGGCATAACCGTTTATAACTGCCGGTCAATTCGGTAAGTACGGTCATCCGCGCGCCTGCTTTC

araC

TAAACCCACTGGTGATACCATTCGCGAGCCTCCGGATGACGACCGTAGTGATGAATCTCTCCTGGCGGGAACAGCAAAATATCACCCGGTCGGCAAACAAATTCTCG
ATTTGGGTGACCACTATGGTAAGCGCTCGGAGGCCTACTGCTGGCATCACTACTTAGAGAGGACCGCCCTTGTCGTTTTATAGTGGGCCAGCCGTTTGTTTAAGAGC

araC

TCCCTGATTTTTCACCACCCCCTGACCGCGAATGGTGAGATTGAGAATATAACCTTTCATTCCCAGCGGTCGGTCGATAAAAAAATCGAGATAACCGTTGGCCTCAA
AGGGACTAAAAAGTGGTGGGGGACTGGCGCTTACCACTCTAACTCTTATATTGGAAAGTAAGGGTCGCCAGCCAGCTATTTTTTTAGCTCTATTGGCAACCGGAGTT

araC

pJ2040 (7459 bp) (from 7384-7459 bp)

```
TCGGCGTTAAACCCGCCACCAGATGGGCATTAAACGAGTATCCCGGCAGCAGGGGATCATTTTGCGCTTCAGCCAT
AGCCGCAATTTGGGCGGTGGTCTACCCGTAATTTGCTCATAGGGCCGTCGTCCCCTAGTAAAACGCGAAGTCGGTA
```

« araC

(from 1-1284 bp)

pJ2040_N2only (7295 bp)

```
ACTTTTCATACTCCCGCCATTCAGAGAAGAAACCAATTGTCCATATTGCATCAGACATTGCCGTCACTGCGTCTTTTACTGGCTCTTCTCGCTAACCAAACCGGTAA
TGAAAAGTATGAGGGCGGTAAGTCTCTTCTTTGGTTAACAGGTATAACGTAGTCTGTAACGGCAGTGACGCAGAAAATGACCGAGAAGAGCGATTGGTTTGGCCATT

CCCCGCTTATTAAAAGCATTCTGTAACAAAGCGGGACCAAAGCCATGACAAAAACGCGTAACAAAAGTGTCTATAATCACGGCAGAAAAGTCCACATTGATTATTTG
GGGGCGAATAATTTTCGTAAGACATTGTTTCGCCCTGGTTTCGGTACTGTTTTTGCGCATTGTTTTCACAGATATTAGTGCCGTCTTTTCAGGTGTAACTAATAAAC

                                                                                                         EcoRI
CACGGCGTCACACTTTGCTATGCCATAGCATTTTTATCCATAAGATTAGCGGtTCCTACCTGACGCTTTTTATCGCAACTCTCTACTGTTTCTCCATACCGAATTCA
GTGCCGCAGTGTGAAACGATACGGTATCGTAAAAATAGGTATTCTAATCGCCaAGGATGGACTGCGAAAAATAGCGTTGAGAGATGACAAAGAGGTATGGCTTAAGT
                                                                P(BAD) promoter

                                   SacI          XhoI
TAGGATAGATTCTGGAAACTTTACCGTCCGAGCTCAGGCTTACCTTACTCGAGCAATAAACAGTTGATAGGGCTTCTCCGTTACAGCCTGCGGTCCGGGTTCACTGC
ATCCTATCTAAGACCTTTGAAATGGCAGGCTCGAGTCCGAATGGAATGAGCTCGTTATTTGTCAACTATCCCGAAGAGGCAATGTCGGACGCCAGGCCCAAGTGACG
     Linker_14                       REs..._8                 Linker_0                   Prefix

CGTATAGGCAGTAATTTTGTTTAACTTTAAGAAGGAGATATACATATGGTTTCGGTTATCAAACCAGAGATGAAAATGCGTTACTATATGGATGGTTCAGTAAATGG
GCATATCCGTCATTAAAACAAATTGAAATTCTTCCTCTATATGTATACCAAAGCCAATAGTTTGGTCTCTACTTTTACGCAATGATATACCTACCAAGTCATTTACC
 Cs...e              RBS                                            mKO2

TCACGAATTTACTATTGAGGGCGAGGGTACGGGACGCCCATACGAGGGGCACCAGGAAATGACTTTACGCGTCACAATGGCTGAAGGCGGGCCTATGCCGTTTGCGT
AGTGCTTAAATGATAACTCCCGCTCCCATGCCCTGCGGGTATGCTCCCCGTGGTCCTTTACTGAAATGCGCAGTGTTACCGACTTCCGCCCGGATACGGCAAACGCA
                                                    mKO2

TCGATCTTGTTAGTCATGTCTTTTGTTACGGTCACCGTGTATTTACTAAATACCCCGAGGAAATTCCAGACTATTTCAAACAAGCCTTCCCGGAAGGTTTGTCTTGG
AGCTAGAACAATCAGTACAGAAAACAATGCCAGTGGCACATAAATGATTTATGGGGCTCCTTTAAGGTCTGATAAAGTTTGTTCGGAAGGGCCTTCCAAACAGAACC
                                                    mKO2

GAGCGCAGTTTAGAGTTTGAAGACGGTGGCTCGGCCAGCGTGTCAGCTCATATTAGTCTTCGCGGCAATACATTTTATCACAAGTCAAAGTTCACCGGCGTGAACTT
CTCGCGTCAAATCTCAAACTTCTGCCACCGAGCCGGTCGCACAGTCGAGTATAATCAGAAGCGCCGTTATGTAAAATAGTGTTCAGTTTCAAGTGGCCGCACTTGAA
                                                    mKO2

CCCCGCAGACGGCCCAATCATGCAGAATCAAAGTGTTGATTGGGAACCGTCCACAGAGAAGATTACAGCTTCCGATGGAGTCTTAAAGGGCGATGTAACCATGTACT
GGGGCGTCTGCCGGGTTAGTACGTCTTAGTTTCACAACTAACCCTTGGCAGGTGTCTCTTCTAATGTCGAAGGCTACCTCAGAATTTCCCGCTACATTGGTACATGA
                                                    mKO2

TAAAATTAGAAGGGGGAGGGAACCATAAATGTCAGATGAAGACTACCTATAAGGCCGCAAAAGAGATTCTTGAAATGCCCGGAGACCACTACATTGGGCATCGTTTG
ATTTTAATCTTCCCCCTCCCTTGGTATTTACAGTCTACTTCTGATGGATATTCCGGCGTTTTCTCTAAGAACTTTACGGGCCTCTGGTGATGTAACCCGTAGCAAAC
                                                    mKO2

GTCCGTAAGACAGAAGGAAATATTACTGAACAGGTCGAAGACGCTGTGGCACACAGCATGTCCCGCCGTAATACTGACGCCATCACAATCCACAGCATCCTGGATTG
CAGGCATTCTGTCTTCCTTTATAATGACTTGTCCAGCTTCTGCGACACCGTGTGTCGTACAGGGCGGCATTATGACTGCGGTAGTGTTAGGTGTCGTAGGACCTAAC
                            mKO2                                                MarA

GATTGAAGACAACTTGGAGTCGCCGTTGAGTTTAGAAAAAGTTAGTGAACGTAGTGGTTACTCAAAGTGGCACCTTCAGCGCATGTTTAAGAAGGAAACGGGTCATT
CTAACTTCTGTTGAACCTCAGCGGCAACTCAAATCTTTTTCAATCACTTGCATCACCAATGAGTTTCACCGTGGAAGTCGCGTACAAATTCTTCCTTTGCCCAGTAA
                                                    MarA
```

pJ2040_N2only (7295 bp) (from 1285-2461 bp)

CATTGGGTCAATATATTCGTTCTCGCAAGATGACTGAAATTGCCCAGAAATTGAAAGAGTCTAATGAACCTATTTTGTACCTGGCGGAGCGTTACGGCTTTGAAAGT
GTAACCCAGTTATATAAGCAAGAGCGTTCTACTGACTTTAACGGGTCTTTAACTTTCTCAGATTACTTGGATAAAACATGGACCGCCTCGCAATGCCGAAACTTTCA
MarA

CAGCAAACCCTTACACGTACCTTCAAGAATTACTTTGACGTTCCACCACACAAATATCGTATGACCAACATGCAGGGTGAGTCACGTTTTTTGCATCCGTTGAATCA
GTCGTTTGGGAATGTGCATGGAAGTTCTTAATGAAACTGCAAGGTGGTGTGTTTATAGCATACTGGTTGTACGTCCCACTCAGTGCAAAAAACGTAGGCAACTTAGT
MarA

NcoI
TTACAATTCCTAATAATCGCTGGGACGCCCGCCATGGTTCAGCCAAAAAACTTAAGACCGCCGGTCTTGTCCACTACCTTGCAGTAATGCGGTGGACAGGATCGGCG
AATGTTAAGGATTATTAGCGACCCTGCGGGCGGTACCAAGTCGGTTTTTTGAATTCTGGCGGCCAGAACAGGTGATGGAACGTCATTACGCCACCTGTCCTAGCCGC
MarA | Suffix | ECK120029600 Terminator

GTTTTCTTTTCTCTTCTCAATTCTTCTGACCTGTAACGAATAATAGATAGTAAAGTAGTCTCCGATTGAGTTTTCTCTGCCGAGTCCCACCCAGTTCTGTGATTTCA
CAAAAGAAAAGAGAAGAGTTAAGAAGACTGGACATTGCTTATTATCTATCATTTCATCAGAGGCTAACTCAAAAGAGACGGCTCAGGGTGGGTCAAGACACTAAAGT
ECK1200...inator | Spacer 1

GTAAGTTGGTAATTGATACACTGTTGCGAGAACTGCTGCCTGGTAGTAGATAGGTTGTTATTGAGTAAGAAGGTAAAGTGAACGAAATCCCTGAAACTGAGACTGTA
CATTCAACCATTAACTATGTGACAACGCTCTTGACGACGGACCATCATCTATCCAACAATAACTCATTCTTCCATTTCACTTGCTTTAGGGACTTTGACTCTGACAT
Spacer 1

HindIII
GAAAATAAGCTTCAGCCTGCGGTCCGGTTGACAGCTAGCTCAGTCCTAGGTACTGTGCTAGCTCGCTGGGACGCCCGGGGACTACACTTACGAAACTATTGATTGCT
CTTTTATTCGAAGTCGGACGCCAGGCCAACTGTCGATCGAGTCAGGATCCATGACACGATCGAGCGACCCTGCGGGCCCCTGATGTGAATGCTTTGATAACTAACGA
Prefix | P(BBa_J23102) | Suffix | Linker_1

BamHI
CAGCCTGCGGTCCGGccaTACATACTCTAAGATGTGTCTCGCTGGGACGCCCGGGATCCAAGAGATTTCTACACGATTGAGCACTGTCTCAGCCTGCGGTCCGGGTT
GTCGGACGCCAGGCCggtATGTATGAGATTCTACACAGAGCGACCCTGCGGGCCCTAGGTTCTCTAAAGATGTGCTAACTCGTGACAGAGTCGGACGCCAGGCCCAA
Prefix | bs-4 | Suffix | Linker_10 | Prefix

CACTGCCGTATAGGCAGTAATTTTGTTTAACTTTAAGAAGGAGATATACATATGCGTAAAGGCGAAGAACTGTTTACCGGTGTGGTTCCGATTCTGGTGGAACTGGA
GTGACGGCATATCCGTCATTAAAACAAATTGAAATTCTTCCTCTATATGTATACGCATTTCCGCTTCTTGACAAATGGCCACACCAAGGCTAAGACCACCTTGACCT
Csy4 site | RBS | sfGFP

CGGCGATGTTAATGGTCATAAATTCAGTGTTCGCGGCGAAGGTGAAGGCGATGCGACGAACGGCAAACTGACCCTGAAATTTATCTGCACCACGGGTAAACTGCCGG
GCCGCTACAATTACCAGTATTTAAGTCACAAGCGCCGCTTCCACTTCCGCTACGCTGCTTGCCGTTTGACTGGGACTTTAAATAGACGTGGTGCCCATTTGACGGCC
sfGFP

TCCCGTGGCCGACGCTGGTGACCACGCTGACCTATGGCGTTCAATGTTTTGCGCGTTACCCGGATCACATGAAACAGCACGACTTTTTCAAATCGGCCATGCCGGAA
AGGGCACCGGCTGCGACCACTGGTGCGACTGGATACCGCAAGTTACAAAACGCGCAATGGGCCTAGTGTACTTTGTCGTGCTGAAAAAGTTTAGCCGGTACGGCCTT
sfGFP

GGCTATGTGCAGGAACGTACGATTAGCTTTAAAGACGATGGTACGTATAAAACCCGCGCGGAAGTGAAATTCGAAGGCGATACCCTGGTTAACCGTATCGAACTGAA
CCGATACACGTCCTTGCATGCTAATCGAAATTTCTGCTACCATGCATATTTTGGGCGCGCCTTCACTTTAAGCTTCCGCTATGGGACCAATTGGCATAGCTTGACTT
sfGFP

pJ2040_N2only (7295 bp) (from 2462-3638 bp)

AGGTATCGATTTCAAAGAAGACGGCAATATTCTGGGTCATAAACTGGAATATAACTTCAATTCCCACAACGTGTACATCACCGCGGATAAACAGAAAAACGGCATTA
TCCATAGCTAAAGTTTCTTCTGCCGTTATAAGACCCAGTATTTGACCTTATATTGAAGTTAAGGGTGTTGCACATGTAGTGGCGCCTATTTGTCTTTTTGCCGTAAT
sfGFP

AAGCCAATTTCAAAATCCGCCATAATGTGGAAGATGGTAGCGTTCAGCTGGCCGACCACTATCAGCAAAACACGCCGATTGGTGATGGCCCGGTCCTGCTGCCGGAC
TTCGGTTAAAGTTTTAGGCGGTATTACACCTTCTACCATCGCAAGTCGACCGGCTGGTGATAGTCGTTTTGTGCGGCTAACCACTACCGGGCCAGGACGACGGCCTG
sfGFP

AATCACTACCTGAGTACCCAGTCCGTGCTGTCAAAAGATCCGAACGAAAAACGTGACCACATGGTCCTGCTGGAATTTGTGACGGCTGCGGGTATCACCCACGGCAT
TTAGTGATGGACTCATGGGTCAGGCACGACAGTTTTCTAGGCTTGCTTTTTGCACTGGTGTACCAGGACGACCTTAAACACTGCCGACGCCCATAGTGGGTGCCGTA
sfGFP

SbfI
GGACGAACTGTATAAAATGTCCCGCCGTAATACTGACGCCATCACAATCCACAGCATCCTGGATTGGATTGAAGACTAATAATCGCTGGGACGCCCGCCTGCAGGCT
CCTGCTTGACATATTTTACAGGGCGGCATTATGACTGCGGTAGTGTTAGGTGTCGTAGGACCTAACCTAACTTCTGATTATTAGCGACCCTGCGGGCGGACGTCCGA
sfGFP MarAn20 Suffix

KpnI
CGGTACCAAATTCCAGAAAAGAGGCCTCCCGAAAGGGGGGCCTTTTTTCGTTTTGGTCCTAATAGATAAAGGATAGGTCTGGTAGTGTTGTTCGTTCTCGCAGGTAA
GCCATGGTTTAAGGTCTTTTCTCCGGAGGGCTTTCCCCCCGGAAAAAAGCAAAACCAGGATTATCTATTTCCTATCCAGACCATCACAACAAGCAAGAGCGTCCATT
L3S2P21 Terminator Spacer 2.5

ATCAATAATACTCAGCAGTTCCGTAGACTTTTCAGTGGGACAGGGTAGCGATAACAGATAGATTGTAATAAGACACAGTAGGTGCTCGTAGTTGCGTGAAGAGAACC
TAGTTATTATGAGTCGTCAAGGCATCTGAAAAGTCACCCTGTCCCATCGCTATTGTCTATCTAACATTATTCTGTGTCATCCACGAGCATCAACGCACTTCTCTTGG
Spacer 2.5

SalI AatII
GCTCAGGAAATCCAGTCAGAAGTATTGGTAATCGTTGAAAACTCAGTCGACGCACTTACTGAAGACGTCCTATTACACTCGTCGTTGGAAACTGAAGATCAGCCTGC
CGAGTCCTTTAGGTCAGTCTTCATAACCATTAGCAACTTTTGAGTCAGCTGCGTGAATGACTTCTGCAGGATAATGTGAGCAGCAACCTTTGACTTCTAGTCGGACG
Spacer 2.5 Spa... 5 Linker_11

GGTCCGGGTTCACTGCCGTATAGGCAGTAATTTTGTTTAACTTTAAGAAGGAGATATACATATGAATCAGTCATTCATCTCGGACATCTTATATGCCGACATCGAAT
CCAGGCCCAAGTGACGGCATATCCGTCATTAAAACAAATTGAAATTCTTCCTCTATATGTATACTTAGTCAGTAAGTAGAGCCTGTAGAATATACGGCTGTAGCTTA
Csy4 site RBS RepA70

CGAAGGCTAAGGAACTTACAGTCAATTCCAACAATACTGTCCAGCCGGTCGCGCTTATGCGCTTAGGAGTTTTCGTTCCCAAACCTTCCAAGAGCAAAGGAGAAAGT
GCTTCCGATTCCTTGAATGTCAGTTAAGGTTGTTATGACAGGTCGGCCAGCGCGAATACGCGAATCCTCAAAAGCAAGGGTTTGGAAGGTTCTCGTTTCCTCTTTCA
RepA70

AAGGAAATTGACGCCACCAAAGCCTTCTCTCAACTGGAGATTGCTAAAGCAGAGGGCatggttagtaaaggagaagaaaataacatggcaCTGATTAAGGAGAACAT
TTCCTTTAACTGCGGTGGTTTCGGAAGAGAGTTGACCTCTAACGATTTCGTCTCCCGtaccaatcatttcctcttcttttattgtaccgtGACTAATTCCTCTTGTA
RepA70 mKate2

GCACATGAAGCTGTACATGGAGGGCACCGTGAACAACCACCACTTCAAGTGCACATCCGAGGGCGAAGGCAAGCCCTACGAGGGCACCCAGACCATGAGAATCAAGg
CGTGTACTTCGACATGTACCTCCCGTGGCACTTGTTGGTGGTGAAGTTCACGTGTAGGCTCCCGCTTCCGTTCGGGATGCTCCCGTGGGTCTGGTACTCTTAGTTCc
mKate2

pJ2040_N2only (7295 bp) (from 3639-4922 bp)

ccGTCGAGGGCGGCCCTCTCCCCTTCGCCTTCGACATCCTGGCTACCAGCTTCATGTACGGCAGCAAAACCTTCATCAACCACACCCAGGGCATCCCCGACTTCTTT
ggCAGCTCCCGCCGGGAGAGGGGAAGCGGAAGCTGTAGGACCGATGGTCGAAGTACATGCCGTCGTTTTGGAAGTAGTTGGTGTGGGTCCCGTAGGGGCTGAAGAAA
mKate2

AAGCAGTCCTTCCCTGAGGGCTTCACATGGGAGAGAGTCACCACATACGAAGACGGGGGCGTGCTGACCGCTACCCAGGACACCAGCCTCCAGGACGGCTGCCTCAT
TTCGTCAGGAAGGGACTCCCGAAGTGTACCCTCTCTCAGTGGTGTATGCTTCTGCCCCCGCACGACTGGCGATGGGTCCTGTGGTCGGAGGTCCTGCCGACGGAGTA
mKate2

CTACAACGTCAAGATCAGAGGGGTGAACTTCCCATCCAACGGCCCTGTGATGCAGAAGAAAACACTCGGCTGGGAGGCCTCCACCGAGaccCTGTACCCCGCTGACG
GATGTTGCAGTTCTAGTCTCCCCACTTGAAGGGTAGGTTGCCGGGACACTACGTCTTCTTTTGTGAGCCGACCCTCCGGAGGTGGCTCtggGACATGGGGCGACTGC
mKate2

GCGGCCTGGAAGGCAGAgcCGACATGGCCCTGAAGCTCGTGGGCGGGGGCCACCTGATCTGCAACTTGAAGACCACATACAGATCCAAGAAACCCGCTAAGAACCTC
CGCCGGACCTTCCGTCTcgGCTGTACCGGGACTTCGAGCACCCGCCCCCGGTGGACTAGACGTTGAACTTCTGGTGTATGTCTAGGTTCTTTGGGCGATTCTTGGAG
mKate2

AAGATGCCCGGCGTCTACTATGTGGACAGAAGACTGGAAAGAATCAAGGAGGCCGACAAAGAGACCTACGTCGAGCAGCACGAGGTGGCTGTGGCCAGATACTGCGA
TTCTACGGGCCGCAGATGATACACCTGTCTTCTGACCTTTCTTAGTTCCTCCGGCTGTTTCTCTGGATGCAGCTCGTCGTGCTCCACCGACACCGGTCTATGACGCT
mKate2

NotI
CCTCCCTAGCAAACTGGGGCACAgAgtctaATAATCGCTGGGACGCCCGGCGGCCGCggaaacacagAAAAAAGCCCGCACCTGACAGTGCGGGCTTTTTTTTTcga
GGAGGGATCGTTTGACCCCGTGTcTcagatTATTAGCGACCCTGCGGGCCGCCGGCGcctttgtgtcTTTTTTCGGGCGTGGACTGTCACGCCCGAAAAAAAAAgct
mKate2 | Suffix | ECK120033737 Terminator

KasI BbvCI AscI
ccaaaggTAGCGAACGACGAGTCACTGTTGAGGATAAATACTTTCTCTACTAGGCGCCTGTTACACAGGTCCTCAGCGGCGCGCCTTTGTCGGTGAACGCTCTCCTG
ggtttccATCGCTTGCTGCTCAGTGACAACTCCTATTTATGAAAGAGATGATCCGCGGACAATGTGTCCAGGAGTCGCCGCGCGGAAACAGCCACTTGCGAGAGGAC
Linker_8 | Spa...15

AGTAGGACAAATCCGCCGGGAGCGGATTTGAACGTTGTGAAGCAACGGCCCGGAGGGTGGCGGGCAGGACGCCCGCCATAAACTGCCAGGCATCAAACTAAGCAGAA
TCATCCTGTTTAGGCGGCCCTCGCCTAAACTTGCAACACTTCGTTGCCGGGCCTCCCACCGCCCGTCCTGCGGGCGGTATTTGACGGTCCGTAGTTTGATTCGTCTT

GGCCATCCTGACGGATGGCCTTTTTGCGTTTCAGATCTACCGGTaaaccagcaatagacataagcggctatttaacgaccctgccctgaaccgacgacaagctgacg
CCGGTAGGACTGCCTACCGGAAAAACGCAAAGTCTAGATGGCCAtttggtcgttatctgtattcgccgataaattgctgggacgggacttggctgctgttcgactgc

accgggtctccgcaagtggcacttttcggggaaatgtgcgcggaacccctatttgtttatttttctaaatacattcaaatatgtatccgctcatgaattaattctta
tggcccagaggcgttcaccgtgaaaagcccctttacacgcgccttggggataaacaaataaaaagatttatgtaagtttatacataggcgagtacttaattaagaat

gaaaaactcatcgagcatcaaatgaaactgcaatttattcatatcaggattatcaataccatatttttgaaaaagccgtttctgtaatgaaggagaaaactcaccga
ctttttgagtagctcgtagtttactttgacgttaaataagtatagtcctaatagttatggtataaaaactttttcggcaaagacattacttcctcttttgagtggct
KanR

ggcagttccataggatggcaagatcctggtatcggtctgcgattccgactcgtccaacatcaatacaacctattaatttcccctcgtcaaaaataaggttatcaagt
ccgtcaaggtatcctaccgttctaggaccatagccagacgctaaggctgagcaggttgtagttatgttggataattaaaggggagcagtttttattccaatagttca
KanR

pJ2040_N2only (7295 bp) (from 4923-6206 bp)

```
gagaaatcaccatgagtgacgactgaatccggtgagaatggcaaaagtttatgcatttctttccagacttgttcaacaggccagccattacgctcgtcatcaaaatc
ctctttagtggtactcactgctgacttaggccactcttaccgttttcaaatacgtaaagaaaggtctgaacaagttgtccggtcggtaatgcgagcagtagttttag
KanR

actcgcatcaaccaaaccgttattcattcgtgattgcgcctgagcgagacgaaatacgcggtcgctgttaaaaggacaattacaaacaggaatcgaatgcaaccggc
tgagcgtagttggtttggcaataagtaagcactaacgcggactcgctctgctttatgcgccagcgacaattttcctgttaatgtttgtccttagcttacgttggccg
KanR

gcaggaacactgccagcgcatcaacaatattttcacctgaatcaggatattcttctaatacctggaatgctgttttcccggggatcgcagtggtgagtaaccatgca
cgtccttgtgacggtcgcgtagttgttataaaagtggacttagtcctataagaagattatggaccttacgacaaaagggcccctagcgtcaccactcattggtacgt
KanR

tcatcaggagtacggataaaatgcttgatggtcggaagaggcataaattccgtcagccagtttagtctgaccatctcatctgtaacatcattggcaacgctaccttt
agtagtcctcatgcctattttacgaactaccagccttctccgtatttaaggcagtcggtcaaatcagactggtagagtagacattgtagtaaccgttgcgatggaaa
KanR

gccatgtttcagaaacaactctggcgcatcgggcttcccatacaatcgatagattgtcgcacctgattgcccgacattatcgcgagcccatttatacccatataaat
cggtacaaagtctttgttgagaccgcgtagcccgaagggtatgttagctatctaacagcgtggactaacgggctgtaatagcgctcgggtaaatatgggtatattta
KanR

cagcatccatgttggaatttaatcgcggcctagagcaagacgtttcccgttgaatatggctcatactcttcctttttcaatattattgaagcatttatcagggttat
gtcgtaggtacaaccttaaattagcgccggatctcgttctgcaaagggcaacttataccgagtatgagaaggaaaaagttataataacttcgtaaatagtcccaata
KanR

tgtctcatgagcggatacatatttgaatgtatttagaaaaataaacaaataggcatgctagcgcagaaacgtcctagaagatgccaggaggatacttagcagagaga
acagagtactcgcctatgtataaacttacataaatctttttatttgtttatccgtacgatcgcgtctttgcaggatcttctacggtcctcctatgaatcgtctctct
ColA ori

caataaggccggagcgaagccgtttttccataggctccgcccccctgacgaacatcacgaaatctgacgctcaaatcagtggtggcgaaacccgacaggactataaa
gttattccggcctcgcttcggcaaaaaggtatccgaggcggggggactgcttgtagtgctttagactgcgagtttagtcaccaccgctttgggctgtcctgatattt
ColA ori

gataccaggcgtttccccctgatggctccctcttgcgctctcctgttcccgtcctgcggcgtccgtgttgtggtggaggctttacccaaatcaccacgtcccgttcc
ctatggtccgcaaagggggactaccgagggagaacgcgagaggacaagggcaggacgccgcaggcacaacaccacctccgaaatgggtttagtggtgcagggcaagg
ColA ori

gtgtagacagttcgctccaagctgggctgtgtgcaagaaccccccgttcagcccgactgctgcgccttatccggtaactatcatcttgagtccaacccggaaagaca
cacatctgtcaagcgaggttcgacccgacacacgttcttggggggcaagtcgggctgacgacgcggaataggccattgatagtagaactcaggttgggcctttctgt
ColA ori

cgacaaaacgccactggcagcagccattggtaactgagaattagtggatttagatatcgagagtcttgaagtggtggcctaacagaggctacactgaaggacagta
gctgttttgcggtgaccgtcgtcggtaaccattgactcttaatcacctaaatctatagctctcagaacttcaccaccggattgtctccgatgtgactttcctgtcat
ColA ori

tttggtatctgcgctccactaaagccagttaccaggttaagcagttccccaactgacttaaccttcgatcaaaccgcctccccaggcggtttttcgtttacagagc
aaaccatagacgcgaggtgatttcggtcaatggtccaattcgtcaaggggttgactgaattggaagctagtttggcggaggggtccgccaaaaagcaaatgtctcg
ColA ori
```

pJ2040_N2only (7295 bp) (from 6207-7295 bp)

aggagattacgacgatcgtaaaaggatctcaagaagatcctttacggattcccgacaccatcactctagatttcagtgcaatttatctcttcaaatgtagcacctga
tcctctaatgctgctagcattttcctagagttcttctaggaaatgcctaagggctgtggtagtgagatctaaagtcacgttaaatagagaagtttacatcgtggact
ColA ori

agtcagccccatacgatataagttgtaattctcatgttagtcatgccccgcgcccaccggaaggagctgactgggttgCTCCTAgGGTCTGATTCGTTACCAATTAT
tcagtcggggtatgctatattcaacattaagagtacaatcagtacggggcgcgggtggccttcctcgactgacccaacGAGGATcCCAGACTAAGCAATGGTTAATA

GACAACTTGACGGCTACATCATTCACTTTTTCTTCACAACCGGCACGGAACTCGCTCGGGCTGGCCCCGGTGCATTTTTTAAATACCCGCGAGAAATAGAGTTGATC
CTGTTGAACTGCCGATGTAGTAAGTGAAAAAGAAGTGTTGGCCGTGCCTTGAGCGAGCCCGACCGGGGCCACGTAAAAAATTTATGGGCGCTCTTTATCTCAACTAG
araC

GTCAAAACCAACATTGCGACCGACGGTGGCGATAGGCATCCGGGTGGTGCTCAAAAGCAGCTTCGCCTGGCTGATACGTTGGTCCTCGCGCCAGCTTAAGACGCTAA
CAGTTTTGGTTGTAACGCTGGCTGCCACCGCTATCCGTAGGCCCACCACGAGTTTTCGTCGAAGCGGACCGACTATGCAACCAGGAGCGCGGTCGAATTCTGCGATT
araC

TCCCTAACTGCTGGCGGAAAAGATGTGACAGACGCGACGGCGACAAGCAAACATGCTGTGCGACGCTGGCGATATCAAAATTGCTGTCTGCCAGGTGATCGCTGATG
AGGGATTGACGACCGCCTTTTCTACACTGTCTGCGCTGCCGCTGTTCGTTTGTACGACACGCTGCGACCGCTATAGTTTTAACGACAGACGGTCCACTAGCGACTAC
araC

TACTGACAAGCCTCGCGTACCCGATTATCCATCGGTGGATGGAGCGACTCGTTAATCGCTTCCATGCGCCGCAGTAACAATTGCTCAAGCAGATTTATCGCCAGCAG
ATGACTGTTCGGAGCGCATGGGCTAATAGGTAGCCACCTACCTCGCTGAGCAATTAGCGAAGGTACGCGGCGTCATTGTTAACGAGTTCGTCTAAATAGCGGTCGTC
araC

CTCCGAATAGCGCCCTTCCCCTTGCCCGGCGTTAATGATTTGCCCAAACAGGTCGCTGAAATGCGGCTGGTGCGCTTCATCCGGGCGAAAGAACCCCGTATTGGCAA
GAGGCTTATCGCGGGAAGGGGAACGGGCCGCAATTACTAAACGGGTTTGTCCAGCGACTTTACGCCGACCACGCGAAGTAGGCCCGCTTTCTTGGGGCATAACCGTT
araC

ATATTGACGGCCAGTTAAGCCATTCATGCCAGTAGGCGCGCGGACGAAAGTAAACCCACTGGTGATACCATTCGCGAGCCTCCGGATGACGACCGTAGTGATGAATC
TATAACTGCCGGTCAATTCGGTAAGTACGGTCATCCGCGCGCCTGCTTTCATTTGGGTGACCACTATGGTAAGCGCTCGGAGGCCTACTGCTGGCATCACTACTTAG
araC

TCTCCTGGCGGGAACAGCAAAATATCACCCGGTCGGCAAACAAATTCTCGTCCCTGATTTTTCACCACCCCCTGACCGCGAATGGTGAGATTGAGAATATAACCTTT
AGAGGACCGCCCTTGTCGTTTTATAGTGGGCCAGCCGTTTGTTTAAGAGCAGGGACTAAAAAGTGGTGGGGGACTGGCGCTTACCACTCTAACTCTTATATTGGAAA
araC

CATTCCCAGCGGTCGGTCGATAAAAAAATCGAGATAACCGTTGGCCTCAATCGGCGTTAAACCCGCCACCAGATGGGCATTAAACGAGTATCCCGGCAGCAGGGGAT
GTAAGGGTCGCCAGCCAGCTATTTTTTTAGCTCTATTGGCAACCGGAGTTAGCCGCAATTTGGGCGGTGGTCTACCCGTAATTTGCTCATAGGGCCGTCGTCCCCTA
araC

CATTTTGCGCTTCAGCCAT
GTAAAACGCGAAGTCGGTA
araC

(from 1-1177 bp)

pJ2040_t4 (7455 bp)

ACTTTTCATACTCCCGCCATTCAGAGAAGAAACCAATTGTCCATATTGCATCAGACATTGCCGTCACTGCGTCTTTTACTGGCTCTTCTCGCTAACCAAACCGGTAA
TGAAAAGTATGAGGGCGGTAAGTCTCTTCTTTGGTTAACAGGTATAACGTAGTCTGTAACGGCAGTGACGCAGAAAATGACCGAGAAGAGCGATTGGTTTGGCCATT

CCCCGCTTATTAAAAGCATTCTGTAACAAAGCGGGACCAAAGCCATGACAAAAACGCGTAACAAAAGTGTCTATAATCACGGCAGAAAAGTCCACATTGATTATTTG
GGGGCGAATAATTTTCGTAAGACATTGTTTCGCCCTGGTTTCGGTACTGTTTTTGCGCATTGTTTTCACAGATATTAGTGCCGTCTTTTCAGGTGTAACTAATAAAC

EcoRI
CACGGCGTCACACTTTGCTATGCCATAGCATTTTTATCCATAAGATTAGCGGtTCCTACCTGACGCTTTTTATCGCAACTCTCTACTGTTTCTCCATACCGAATTCA
GTGCCGCAGTGTGAAACGATACGGTATCGTAAAAATAGGTATTCTAATCGCCaAGGATGGACTGCGAAAAATAGCGTTGAGAGATGACAAAGAGGTATGGCTTAAGT
P(BAD) promoter

SacI
TAGGATAGATTCTGGAAACTTTACCGTCCGAGCTCCAGCCTGCGGTCCGGGTTCACTGCCGTATAGGCAGCATCTTAGAGTATGTAGTTTcAGAGCTATGCTGGAAA
ATCCTATCTAAGACCTTTGAAATGGCAGGCTCGAGGTCGGACGCCAGGCCCAAGTGACGGCATATCCGTCGTAGAATCTCATACATCAAAgTCTCGATACGACCTTT
Linker_14 Prefix Csy4 site sgRNA-4t4

XhoI
CAGCATAGCAAGTTgAAATAAGGCTAGTCCGTTATCAACTTGAAAAAGTGGCACCGAGTCGGTGCGTTCACTGCCGTATAGGCAGTCGCTGGGACGCCCGCTCGAGC
GTCGTATCGTTCAAcTTTATTCCGATCAGGCAATAGTTGAACTTTTTCACCGTGGCTCAGCCACGCAAGTGACGGCATATCCGTCAGCGACCCTGCGGGCGAGCTCG
sgRNA-4t4 Csy4 site Suffix

AATAAACAGTTGATAGGGCTTCTCCGTTACAGCCTGCGGTCCGGGTTCACTGCCGTATAGGCAGTAATTTTGTTTAACTTTAAGAAGGAGATATACATATGGTTTCG
TTATTTGTCAACTATCCCGAAGAGGCAATGTCGGACGCCAGGCCCAAGTGACGGCATATCCGTCATTAAAACAAATTGAAATTCTTCCTCTATATGTATACCAAAGC
Linker_0 Prefix Csy4 site RBS mKO2

GTTATCAAACCAGAGATGAAAATGCGTTACTATATGGATGGTTCAGTAAATGGTCACGAATTTACTATTGAGGGCGAGGGTACGGGACGCCCATACGAGGGGCACCA
CAATAGTTTGGTCTCTACTTTTACGCAATGATATACCTACCAAGTCATTTACCAGTGCTTAAATGATAACTCCCGCTCCCATGCCCTGCGGGTATGCTCCCCGTGGT
mKO2

GGAAATGACTTTACGCGTCACAATGGCTGAAGGCGGGCCTATGCCGTTTGCGTTCGATCTTGTTAGTCATGTCTTTTGTTACGGTCACCGTGTATTTACTAAATACC
CCTTTACTGAAATGCGCAGTGTTACCGACTTCCGCCCGGATACGGCAAACGCAAGCTAGAACAATCAGTACAGAAAACAATGCCAGTGGCACATAAATGATTTATGG
mKO2

CCGAGGAAATTCCAGACTATTTCAAACAAGCCTTCCCGGAAGGTTTGTCTTGGGAGCGCAGTTTAGAGTTTGAAGACGGTGGCTCGGCCAGCGTGTCAGCTCATATT
GGCTCCTTTAAGGTCTGATAAAGTTTGTTCGGAAGGGCCTTCCAAACAGAACCCTCGCGTCAAATCTCAAACTTCTGCCACCGAGCCGGTCGCACAGTCGAGTATAA
mKO2

AGTCTTCGCGGCAATACATTTTATCACAAGTCAAAGTTCACCGGCGTGAACTTCCCCGCAGACGGCCCAATCATGCAGAATCAAAGTGTTGATTGGGAACCGTCCAC
TCAGAAGCGCCGTTATGTAAAATAGTGTTCAGTTTCAAGTGGCCGCACTTGAAGGGGCGTCTGCCGGGTTAGTACGTCTTAGTTTCACAACTAACCCTTGGCAGGTG
mKO2

AGAGAAGATTACAGCTTCCGATGGAGTCTTAAAGGGCGATGTAACCATGTACTTAAAATTAGAAGGGGGAGGGAACCATAAATGTCAGATGAAGACTACCTATAAGG
TCTCTTCTAATGTCGAAGGCTACCTCAGAATTTCCCGCTACATTGGTACATGAATTTTAATCTTCCCCCTCCCTTGGTATTTACAGTCTACTTCTGATGGATATTCC
mKO2

pJ2040_t4 (7455 bp) (from 1178-2354 bp)

CCGCAAAAGAGATTCTTGAAATGCCCGGAGACCACTACATTGGGCATCGTTTGGTCCGTAAGACAGAAGGAAATATTACTGAACAGGTCGAAGACGCTGTGGCACAC
GGCGTTTTCTCTAAGAACTTTACGGGCCTCTGGTGATGTAACCCGTAGCAAACCAGGCATTCTGTCTTCCTTTATAATGACTTGTCCAGCTTCTGCGACACCGTGTG
mKO2

AGCATGTCCCGCCGTAATACTGACGCCATCACAATCCACAGCATCCTGGATTGGATTGAAGACAACTTGGAGTCGCCGTTGAGTTTAGAAAAAGTTAGTGAACGTAG
TCGTACAGGGCGGCATTATGACTGCGGTAGTGTTAGGTGTCGTAGGACCTAACCTAACTTCTGTTGAACCTCAGCGGCAACTCAAATCTTTTTCAATCACTTGCATC
MarA

TGGTTACTCAAAGTGGCACCTTCAGCGCATGTTTAAGAAGGAAACGGGTCATTCATTGGGTCAATATATTCGTTCTCGCAAGATGACTGAAATTGCCCAGAAATTGA
ACCAATGAGTTTCACCGTGGAAGTCGCGTACAAATTCTTCCTTTGCCCAGTAAGTAACCCAGTTATATAAGCAAGAGCGTTCTACTGACTTTAACGGGTCTTTAACT
MarA

AAGAGTCTAATGAACCTATTTTGTACCTGGCGGAGCGTTACGGCTTTGAAAGTCAGCAAACCCTTACACGTACCTTCAAGAATTACTTTGACGTTCCACCACACAAA
TTCTCAGATTACTTGGATAAAACATGGACCGCCTCGCAATGCCGAAACTTTCAGTCGTTTGGGAATGTGCATGGAAGTTCTTAATGAAACTGCAAGGTGGTGTGTTT
MarA

NcoI
TATCGTATGACCAACATGCAGGGTGAGTCACGTTTTTTGCATCCGTTGAATCATTACAATTCCTAATAATCGCTGGGACGCCCGCCATGGTTCAGCCAAAAAAACTTA
ATAGCATACTGGTTGTACGTCCCACTCAGTGCAAAAAACGTAGGCAACTTAGTAATGTTAAGGATTATTAGCGACCCTGCGGGCGGTACCAAGTCGGTTTTTTTGAAT
MarA Suffix ECK12...ator

AGACCGCCGGTCTTGTCCACTACCTTGCAGTAATGCGGTGGACAGGATCGGCGGTTTTCTTTTCTCTTCTCAATTCTTCTGACCTGTAACGAATAATAGATAGTAAA
TCTGGCGGCCAGAACAGGTGATGGAACGTCATTACGCCACCTGTCCTAGCCGCCAAAAGAAAAGAGAAGAGTTAAGAAGACTGGACATTGCTTATTATCTATCATTT
ECK120029600 Terminator Spacer 1

GTAGTCTCCGATTGAGTTTTCTCTGCCGAGTCCCACCCAGTTCTGTGATTTCAGTAAGTTGGTAATTGATACACTGTTGCGAGAACTGCTGCCTGGTAGTAGATAGG
CATCAGAGGCTAACTCAAAAGAGACGGCTCAGGGTGGGTCAAGACACTAAAGTCATTCAACCATTAACTATGTGACAACGCTCTTGACGACGGACCATCATCTATCC
Spacer 1

HindIII
TTGTTATTGAGTAAGAAGGTAAAGTGAACGAAATCCCTGAAACTGAGACTGTAGAAAATAAGCTTCAGCCTGCGGTCCGGTTGACAGCTAGCTCAGTCCTAGGTACT
AACAATAACTCATTCTTCCATTTCACTTGCTTTAGGGACTTTGACTCTGACATCTTTTATTCGAAGTCGGACGCCAGGCCAACTGTCGATCGAGTCAGGATCCATGA
Spacer 1 Prefix P(BBa_J23102)

GTGCTAGCTCGCTGGGACGCCCGGGGACTACACTTACGAAACTATTGATTGCTCAGCCTGCGGTCCGGccaTACATACTCTAAGATGTGTCTCGCTGGGACGCCCGG
CACGATCGAGCGACCCTGCGGGCCCCTGATGTGAATGCTTTGATAACTAACGAGTCGGACGCCAGGCCggtATGTATGAGATTCTACACAGAGCGACCCTGCGGGCC
Suffix Linker_1 Prefix bs-4 Suffix

BamHI
GATCCAAGAGATTTCTACACGATTGAGCACTGTCTCAGCCTGCGGTCCGGGTTCACTGCCGTATAGGCAGTAATTTTGTTTAACTTTAAGAAGGAGATATACATATG
CTAGGTTCTCTAAAGATGTGCTAACTCGTGACAGAGTCGGACGCCAGGCCCAAGTGACGGCATATCCGTCATTAAAACAAATTGAAATTCTTCCTCTATATGTATAC
Linker_10 Prefix Csy4 site RBS

CGTAAAGGCGAAGAACTGTTTACCGGTGTGGTTCCGATTCTGGTGGAACTGGACGGCGATGTTAATGGTCATAAATTCAGTGTTCGCGGCGAAGGTGAAGGCGATGC
GCATTTCCGCTTCTTGACAAATGGCCACACCAAGGCTAAGACCACCTTGACCTGCCGCTACAATTACCAGTATTTAAGTCACAAGCGCCGCTTCCACTTCCGCTACG
sfGFP

pJ2040_t4 (7455 bp) (from 2355-3531 bp)

GACGAACGGCAAACTGACCCTGAAATTTATCTGCACCACGGGTAAACTGCCGGTCCCGTGGCCGACGCTGGTGACCACGCTGACCTATGGCGTTCAATGTTTTGCGC
CTGCTTGCCGTTTGACTGGGACTTTAAATAGACGTGGTGCCCATTTGACGGCCAGGGCACCGGCTGCGACCACTGGTGCGACTGGATACCGCAAGTTACAAAACGCG
sfGFP

GTTACCCGGATCACATGAAACAGCACGACTTTTTCAAATCGGCCATGCCGGAAGGCTATGTGCAGGAACGTACGATTAGCTTTAAAGACGATGGTACGTATAAAACC
CAATGGGCCTAGTGTACTTTGTCGTGCTGAAAAAGTTTAGCCGGTACGGCCTTCCGATACACGTCCTTGCATGCTAATCGAAATTTCTGCTACCATGCATATTTTGG
sfGFP

CGCGCGGAAGTGAAATTCGAAGGCGATACCCTGGTTAACCGTATCGAACTGAAAGGTATCGATTTCAAAGAAGACGGCAATATTCTGGGTCATAAACTGGAATATAA
GCGCGCCTTCACTTTAAGCTTCCGCTATGGGACCAATTGGCATAGCTTGACTTTCCATAGCTAAAGTTTCTTCTGCCGTTATAAGACCCAGTATTTGACCTTATATT
sfGFP

CTTCAATTCCCACAACGTGTACATCACCGCGGATAAACAGAAAAACGGCATTAAAGCCAATTTCAAAATCCGCCATAATGTGGAAGATGGTAGCGTTCAGCTGGCCG
GAAGTTAAGGGTGTTGCACATGTAGTGGCGCCTATTTGTCTTTTTGCCGTAATTTCGGTTAAAGTTTTAGGCGGTATTACACCTTCTACCATCGCAAGTCGACCGGC
sfGFP

ACCACTATCAGCAAAACACGCCGATTGGTGATGGCCCGGTCCTGCTGCCGGACAATCACTACCTGAGTACCCAGTCCGTGCTGTCAAAAGATCCGAACGAAAAACGT
TGGTGATAGTCGTTTTGTGCGGCTAACCACTACCGGGCCAGGACGACGGCCTGTTAGTGATGGACTCATGGGTCAGGCACGACAGTTTTCTAGGCTTGCTTTTTGCA
sfGFP

GACCACATGGTCCTGCTGGAATTTGTGACGGCTGCGGGTATCACCCACGGCATGGACGAACTGTATAAAATGTCCCGCCGTAATACTGACGCCATCACAATCCACAG
CTGGTGTACCAGGACGACCTTAAACACTGCCGACGCCCATAGTGGGTGCCGTACCTGCTTGACATATTTTACAGGGCGGCATTATGACTGCGGTAGTGTTAGGTGTC
sfGFP MarAn20

SbfI KpnI
CATCCTGGATTGGATTGAAGACTAATAATCGCTGGGACGCCCGCCTGCAGGCTCGGTACCAAATTCCAGAAAAGAGGCCTCCCGAAAGGGGGGCCTTTTTTCGTTTT
GTAGGACCTAACCTAACTTCTGATTATTAGCGACCCTGCGGGCGGACGTCCGAGCCATGGTTTAAGGTCTTTTCTCCGGAGGGCTTTCCCCCCGGAAAAAAGCAAAA
MarAn20 Suffix L3S2P21 Terminator

GGTCCTAATAGATAAAGGATAGGTCTGGTAGTGTTGTTCGTTCTCGCAGGTAAATCAATAATACTCAGCAGTTCCGTAGACTTTTCAGTGGGACAGGGTAGCGATAA
CCAGGATTATCTATTTCCTATCCAGACCATCACAACAAGCAAGAGCGTCCATTTAGTTATTATGAGTCGTCAAGGCATCTGAAAAGTCACCCTGTCCCATCGCTATT
Spacer 2.5

SalI
CAGATAGATTGTAATAAGACACAGTAGGTGCTCGTAGTTGCGTGAAGAGAACCGCTCAGGAAATCCAGTCAGAAGTATTGGTAATCGTTGAAAACTCAGTCGACGCA
GTCTATCTAACATTATTCTGTGTCATCCACGAGCATCAACGCACTTCTCTTGGCGAGTCCTTTAGGTCAGTCTTCATAACCATTAGCAACTTTTGAGTCAGCTGCGT
Spacer 2.5

AatII
CTTACTGAAGACGTCCTATTACACTCGTCGTTGGAAACTGAAGATCAGCCTGCGGTCCGGGTTCACTGCCGTATAGGCAGTAATTTTGTTTAACTTTAAGAAGGAGA
GAATGACTTCTGCAGGATAATGTGAGCAGCAACCTTTGACTTCTAGTCGGACGCCAGGCCCAAGTGACGGCATATCCGTCATTAAAACAAATTGAAATTCTTCCTCT
Linker_11 Prefix Csy4 site RBS

TATACATATGAATCAGTCATTCATCTCGGACATCTTATATGCCGACATCGAATCGAAGGCTAAGGAACTTACAGTCAATTCCAACAATACTGTCCAGCCGGTCGCGC
ATATGTATACTTAGTCAGTAAGTAGAGCCTGTAGAATATACGGCTGTAGCTTAGCTTCCGATTCCTTGAATGTCAGTTAAGGTTGTTATGACAGGTCGGCCAGCGCG
RepA70

pJ2040_t4 (7455 bp) (from 3532-4815 bp)

TTATGCGCTTAGGAGTTTTCGTTCCCAAACCTTCCAAGAGCAAAGGAGAAAGTAAGGAAATTGACGCCACCAAAGCCTTCTCTCAACTGGAGATTGCTAAAGCAGAG
AATACGCGAATCCTCAAAAGCAAGGGTTTGGAAGGTTCTCGTTTCCTCTTTCATTCCTTTAACTGCGGTGGTTTCGGAAGAGAGTTGACCTCTAACGATTTCGTCTC
RepA70

GGCatggttagtaaaggagaagaaaataacatggcaCTGATTAAGGAGAACATGCACATGAAGCTGTACATGGAGGGCACCGTGAACAACCACCACTTCAAGTGCAC
CCGtaccaatcatttcctcttcttttattgtaccgtGACTAATTCCTCTTGTACGTGTACTTCGACATGTACCTCCCGTGGCACTTGTTGGTGGTGAAGTTCACGTG
mKate2

ATCCGAGGGCGAAGGCAAGCCCTACGAGGGCACCCAGACCATGAGAATCAAGgccGTCGAGGGCGGCCCTCTCCCCTTCGCCTTCGACATCCTGGCTACCAGCTTCA
TAGGCTCCCGCTTCCGTTCGGGATGCTCCCGTGGGTCTGGTACTCTTAGTTCcggCAGCTCCCGCCGGGAGAGGGGAAGCGGAAGCTGTAGGACCGATGGTCGAAGT
mKate2

TGTACGGCAGCAAAACCTTCATCAACCACACCCAGGGCATCCCCGACTTCTTTAAGCAGTCCTTCCCTGAGGGCTTCACATGGGAGAGAGTCACCACATACGAAGAC
ACATGCCGTCGTTTTGGAAGTAGTTGGTGTGGGTCCCGTAGGGGCTGAAGAAATTCGTCAGGAAGGGACTCCCGAAGTGTACCCTCTCTCAGTGGTGTATGCTTCTG
mKate2

GGGGGCGTGCTGACCGCTACCCAGGACACCAGCCTCCAGGACGGCTGCCTCATCTACAACGTCAAGATCAGAGGGGTGAACTTCCCATCCAACGGCCCTGTGATGCA
CCCCCGCACGACTGGCGATGGGTCCTGTGGTCGGAGGTCCTGCCGACGGAGTAGATGTTGCAGTTCTAGTCTCCCCACTTGAAGGGTAGGTTGCCGGGACACTACGT
mKate2

GAAGAAAACACTCGGCTGGGAGGCCTCCACCGAGaccCTGTACCCCGCTGACGGCGGCCTGGAAGGCAGAgcCGACATGGCCCTGAAGCTCGTGGGCGGGGGCCACC
CTTCTTTTGTGAGCCGACCCTCCGGAGGTGGCTCtggGACATGGGGCGACTGCCGCCGGACCTTCCGTCTcgGCTGTACCGGGACTTCGAGCACCCGCCCCCGGTGG
mKate2

TGATCTGCAACTTGAAGACCACATACAGATCCAAGAAACCCGCTAAGAACCTCAAGATGCCCGGCGTCTACTATGTGGACAGAAGACTGGAAAGAATCAAGGAGGCC
ACTAGACGTTGAACTTCTGGTGTATGTCTAGGTTCTTTGGGCGATTCTTGGAGTTCTACGGGCCGCAGATGATACACCTGTCTTCTGACCTTTCTTAGTTCCTCCGG
mKate2

NotI

GACAAAGAGACCTACGTCGAGCAGCACGAGGTGGCTGTGGCCAGATACTGCGACCTCCCTAGCAAACTGGGGCACAgAgtctaATAATCGCTGGGACGCCCGGCGGC
CTGTTTCTCTGGATGCAGCTCGTCGTGCTCCACCGACACCGGTCTATGACGCTGGAGGGATCGTTTGACCCCGTGTcTcagatTATTAGCGACCCTGCGGGCCGCCG
mKate2 Suffix

KasI

CGCggaaacacagAAAAAAGCCCGCACCTGACAGTGCGGGCTTTTTTTTTcgaccaaaggTAGCGAACGACGAGTCACTGTTGAGGATAAATACTTTCTCTACTAGG
GCGcctttgtgtcTTTTTTCGGGCGTGGACTGTCACGCCCGAAAAAAAAAgctggtttccATCGCTTGCTGCTCAGTGACAACTCCTATTTATGAAAGAGATGATCC
ECK120033737 Terminator Linker_8

BbvCI AscI

CGCCTGTTACACAGGTCCTCAGCGGCGCGCCTTTGTCGGTGAACGCTCTCCTGAGTAGGACAAATCCGCCGGGAGCGGATTTGAACGTTGTGAAGCAACGGCCCGGA
GCGGACAATGTGTCCAGGAGTCGCCGCGCGGAAACAGCCACTTGCGAGAGGACTCATCCTGTTTAGGCGGCCCTCGCCTAAACTTGCAACACTTCGTTGCCGGGCCT
Spa...15

GGGTGGCGGGCAGGACGCCCGCCATAAACTGCCAGGCATCAAACTAAGCAGAAGGCCATCCTGACGGATGGCCTTTTTGCGTTTCAGATCTACCGGTaaaccagcaa
CCCACCGCCCGTCCTGCGGGCGGTATTTGACGGTCCGTAGTTTGATTCGTCTTCCGGTAGGACTGCCTACCGGAAAAACGCAAAGTCTAGATGGCCAtttggtcgtt

tagacataagcggctatttaacgaccctgccctgaaccgacgacaagctgacgaccgggtctccgcaagtggcacttttcggggaaatgtgcgcggaacccctattt
atctgtattcgccgataaattgctgggacgggacttggctgctgttcgactgctggcccagaggcgttcaccgtgaaaagccccttttacacgcgccttggggataaa

pJ2040_t4 (7455 bp) (from 4816-6099 bp)

```
gtttatttttctaaatacattcaaatatgtatccgctcatgaattaattcttagaaaaactcatcgagcatcaaatgaaactgcaatttattcatatcaggattatc
caaataaaaagatttatgtaagtttatacataggcgagtacttaattaagaatctttttgagtagctcgtagtttactttgacgttaaataagtatagtcctaatag
                                                                              KanR

aataccatatttttgaaaaagccgtttctgtaatgaaggagaaaactcaccgaggcagttccataggatggcaagatcctggtatcggtctgcgattccgactcgtc
ttatggtataaaaactttttcggcaaagacattacttcctcttttgagtggctccgtcaaggtatcctaccgttctaggaccatagccagacgctaaggctgagcag
                                                   KanR

caacatcaatacaacctattaatttcccctcgtcaaaaataaggttatcaagtgagaaatcaccatgagtgacgactgaatccggtgagaatggcaaaagtttatgc
gttgtagttatgttggataattaaaggggagcagtttttattccaatagttcactctttagtggtactcactgctgacttaggccactcttaccgttttcaaatacg
                                                   KanR

atttctttccagacttgttcaacaggccagccattacgctcgtcatcaaaatcactcgcatcaaccaaaccgttattcattcgtgattgcgcctgagcgagacgaaa
taaagaaaggtctgaacaagttgtccggtcggtaatgcgagcagtagttttagtgagcgtagttggtttggcaataagtaagcactaacgcggactcgctctgcttt
                                                   KanR

tacgcggtcgctgttaaaaggacaattacaaacaggaatcgaatgcaaccggcgcaggaacactgccagcgcatcaacaatattttcacctgaatcaggatattctt
atgcgccagcgacaattttcctgttaatgtttgtccttagcttacgttggccgcgtccttgtgacggtcgcgtagttgttataaaagtggacttagtcctataagaa
                                                   KanR

ctaatacctggaatgctgttttcccggggatcgcagtggtgagtaaccatgcatcatcaggagtacggataaaatgcttgatggtcggaagaggcataaattccgtc
gattatggaccttacgacaaaagggcccctagcgtcaccactcattggtacgtagtagtcctcatgcctattttacgaactaccagccttctccgtatttaaggcag
                                                   KanR

agccagtttagtctgaccatctcatctgtaacatcattggcaacgctacctttgccatgtttcagaaacaactctggcgcatcgggcttcccatacaatcgatagat
tcggtcaaatcagactggtagagtagacattgtagtaaccgttgcgatggaaacggtacaaagtctttgttgagaccgcgtagcccgaagggtatgttagctatcta
                                                   KanR

tgtcgcacctgattgcccgacattatcgcgagcccatttatacccatataaatcagcatccatgttggaatttaatcgcggcctagagcaagacgtttcccgttgaa
acagcgtggactaacgggctgtaatagcgctcgggtaaatatgggtatatttagtcgtaggtacaaccttaaattagcgccggatctcgttctgcaaagggcaactt
                                                   KanR

tatggctcatactcttcctttttcaatattattgaagcatttatcagggttattgtctcatgagcggatacatatttgaatgtatttagaaaaataaacaaataggc
ataccgagtatgagaaggaaaaagttataataacttcgtaaatagtcccaataacagagtactcgcctatgtataaacttacataaatctttttatttgtttatccg
   KanR

atgctagcgcagaaacgtcctagaagatgccaggaggatacttagcagagagacaataaggccggagcgaagccgtttttccataggctccgcccccctgacgaaca
tacgatcgcgtctttgcaggatcttctacggtcctcctatgaatcgtctctctgttattccggcctcgcttcggcaaaaaggtatccgaggcggggggactgcttgt
                                                        ColA ori

tcacgaaatctgacgctcaaatcagtggtggcgaaacccgacaggactataaagataccaggcgtttccccctgatggctccctcttgcgctctcctgttcccgtcc
agtgctttagactgcgagtttagtcaccaccgctttgggctgtcctgatatttctatggtccgcaaagggggactaccgagggagaacgcgagaggacaagggcagg
                                                   ColA ori

tgcggcgtccgtgttgtggtggaggctttacccaaatcaccacgtcccgttccgtgtagacagttcgctccaagctgggctgtgtgcaagaaccccccgttcagccc
acgccgcaggcacaacaccacctccgaaatgggtttagtggtgcagggcaaggcacatctgtcaagcgaggttcgacccgacacacgttcttggggggcaagtcggg
                                                   ColA ori
```

pJ2040_t4 (7455 bp) (from 6100-7383 bp)

```
gactgctgcgccttatccggtaactatcatcttgagtccaacccggaaagacacgacaaaacgccactggcagcagccattggtaactgagaattagtggatttaga
ctgacgacgcggaataggccattgatagtagaactcaggttgggcctttctgtgctgttttgcggtgaccgtcgtcggtaaccattgactcttaatcacctaaatct
                                              ColA ori

tatcgagagtcttgaagtggtggcctaacagaggctacactgaaaggacagtatttggtatctgcgctccactaaagccagttaccaggttaagcagttccccaact
atagctctcagaacttcaccaccggattgtctccgatgtgactttcctgtcataaaccatagacgcgaggtgatttcggtcaatggtccaattcgtcaaggggttga
                                              ColA ori

gacttaaccttcgatcaaaccgcctccccaggcggtttttcgtttacagagcaggagattacgacgatcgtaaaaggatctcaagaagatcctttacggattcccg
ctgaattggaagctagtttggcggaggggtccgccaaaaagcaaatgtctcgtcctctaatgctgctagcattttcctagagttcttctaggaaatgcctaagggc
                                              ColA ori

acaccatcactctagatttcagtgcaatttatctcttcaaatgtagcacctgaagtcagccccatacgatataagttgtaattctcatgttagtcatgccccgcgcc
tgtggtagtgagatctaaagtcacgttaaatagagaagtttacatcgtggacttcagtcggggtatgctatattcaacattaagagtacaatcagtacggggcgcgg

caccggaaggagctgactgggttgCTCCTAgGGTCTGATTCGTTACCAATTATGACAACTTGACGGCTACATCATTCACTTTTTCTTCACAACCGGCACGGAACTCG
gtggccttcctcgactgacccaacGAGGATcCCAGACTAAGCAATGGTTAATACTGTTGAACTGCCGATGTAGTAAGTGAAAAAGAAGTGTTGGCCGTGCCTTGAGC
                                                                          araC

CTCGGGCTGGCCCCGGTGCATTTTTTAAATACCCGCGAGAAATAGAGTTGATCGTCAAAACCAACATTGCGACCGACGGTGGCGATAGGCATCCGGGTGGTGCTCAA
GAGCCCGACCGGGGCCACGTAAAAAATTTATGGGCGCTCTTTATCTCAACTAGCAGTTTTGGTTGTAACGCTGGCTGCCACCGCTATCCGTAGGCCCACCACGAGTT
                                                araC

AAGCAGCTTCGCCTGGCTGATACGTTGGTCCTCGCGCCAGCTTAAGACGCTAATCCCTAACTGCTGGCGGAAAAGATGTGACAGACGCGACGGCGACAAGCAAACAT
TTCGTCGAAGCGGACCGACTATGCAACCAGGAGCGCGGTCGAATTCTGCGATTAGGGATTGACGACCGCCTTTTCTACACTGTCTGCGCTGCCGCTGTTCGTTTGTA
                                                araC

GCTGTGCGACGCTGGCGATATCAAAATTGCTGTCTGCCAGGTGATCGCTGATGTACTGACAAGCCTCGCGTACCCGATTATCCATCGGTGGATGGAGCGACTCGTTA
CGACACGCTGCGACCGCTATAGTTTTAACGACAGACGGTCCACTAGCGACTACATGACTGTTCGGAGCGCATGGGCTAATAGGTAGCCACCTACCTCGCTGAGCAAT
                                                araC

ATCGCTTCCATGCGCCGCAGTAACAATTGCTCAAGCAGATTTATCGCCAGCAGCTCCGAATAGCGCCCTTCCCCTTGCCCGGCGTTAATGATTTGCCCAAACAGGTC
TAGCGAAGGTACGCGGCGTCATTGTTAACGAGTTCGTCTAAATAGCGGTCGTCGAGGCTTATCGCGGGAAGGGGAACGGGCCGCAATTACTAAACGGGTTTGTCCAG
                                                araC

GCTGAAATGCGGCTGGTGCGCTTCATCCGGGCGAAAGAACCCCGTATTGGCAAATATTGACGGCCAGTTAAGCCATTCATGCCAGTAGGCGCGCGGACGAAAGTAAA
CGACTTTACGCCGACCACGCGAAGTAGGCCCGCTTTCTTGGGGCATAACCGTTTATAACTGCCGGTCAATTCGGTAAGTACGGTCATCCGCGCGCCTGCTTTCATTT
                                                araC

CCCACTGGTGATACCATTCGCGAGCCTCCGGATGACGACCGTAGTGATGAATCTCTCCTGGCGGGAACAGCAAAATATCACCCGGTCGGCAAACAAATTCTCGTCCC
GGGTGACCACTATGGTAAGCGCTCGGAGGCCTACTGCTGGCATCACTACTTAGAGAGGACCGCCCTTGTCGTTTTATAGTGGGCCAGCCGTTTGTTTAAGAGCAGGG
                                                araC

TGATTTTTCACCACCCCCTGACCGCGAATGGTGAGATTGAGAATATAACCTTTCATTCCCAGCGGTCGGTCGATAAAAAAATCGAGATAACCGTTGGCCTCAATCGG
ACTAAAAAGTGGTGGGGGACTGGCGCTTACCACTCTAACTCTTATATTGGAAAGTAAGGGTCGCCAGCCAGCTATTTTTTTAGCTCTATTGGCAACCGGAGTTAGCC
                                                araC
```

pJ2040_t4 (7455 bp) (from 7384-7455 bp)

CGTTAAACCCGCCACCAGATGGGCATTAAACGAGTATCCCGGCAGCAGGGGATCATTTTGCGCTTCAGCCAT
GCAATTTGGGCGGTGGTCTACCCGTAATTTGCTCATAGGGCCGTCGTCCCCTAGTAAAACGCGAAGTCGGTA

araC

(from 1-1177 bp)

pJ2042.2 (8003 bp)

ACTTTTCATACTCCCGCCATTCAGAGAAGAAACCAATTGTCCATATTGCATCAGACATTGCCGTCACTGCGTCTTTTACTGGCTCTTCTCGCTAACCAAACCGGTAA
TGAAAAGTATGAGGGCGGTAAGTCTCTTCTTTGGTTAACAGGTATAACGTAGTCTGTAACGGCAGTGACGCAGAAAATGACCGAGAAGAGCGATTGGTTTGGCCATT

CCCCGCTTATTAAAAGCATTCTGTAACAAAGCGGGACCAAAGCCATGACAAAAACGCGTAACAAAAGTGTCTATAATCACGGCAGAAAAGTCCACATTGATTATTTG
GGGGCGAATAATTTTCGTAAGACATTGTTTCGCCCTGGTTTCGGTACTGTTTTTGCGCATTGTTTTCACAGATATTAGTGCCGTCTTTTCAGGTGTAACTAATAAAC

EcoRI

CACGGCGTCACACTTTGCTATGCCATAGCATTTTTATCCATAAGATTAGCGGtTCCTACCTGACGCTTTTTATCGCAACTCTCTACTGTTTCTCCATACCGAATTCA
GTGCCGCAGTGTGAAACGATACGGTATCGTAAAAATAGGTATTCTAATCGCCaAGGATGGACTGCGAAAAATAGCGTTGAGAGATGACAAAGAGGTATGGCTTAAGT

P(BAD) promoter

SacI

TAGGATAGATTCTGGAAACTTTACCGTCCGAGCTCCAGCCTGCGGTCCGGGTTCACTGCCGTATAGGCAGAAGCTAGACTCTAGTGGTTTcAGAGCTATGCTGGAAA
ATCCTATCTAAGACCTTTGAAATGGCAGGCTCGAGGTCGGACGCCAGGCCCAAGTGACGGCATATCCGTCTTCGATCTGAGATCACCAAAgTCTCGATACGACCTTT

Linker_14 | Prefix | Csy4 site | sgRNA-1t4

CAGCATAGCAAGTTgAAATAAGGCTAGTCCGTTATCAACTTGAAAAAGTGGCACCGAGTCGGTGCGTTCACTGCCGTATAGGCAGTCGCTGGGACGCCCGGGGACTA
GTCGTATCGTTCAAcTTTATTCCGATCAGGCAATAGTTGAACTTTTTCACCGTGGCTCAGCCACGCAAGTGACGGCATATCCGTCAGCGACCCTGCGGGCCCCTGAT

sgRNA-1t4 | Csy4 site | Suffix

CACTTACGAAACTATTGATTGCTCAGCCTGCGGTCCGGGTTCACTGCCGTATAGGCAGATCAGTGTGTACTAAGTACTGTTTcAGAGCTATGCTGGAAACAGCATAG
GTGAATGCTTTGATAACTAACGAGTCGGACGCCAGGCCCAAGTGACGGCATATCCGTCTAGTCACACATGATTCATGACAAAgTCTCGATACGACCTTTGTCGTATC

Linker_1 | Prefix | Csy4 site | sgRNA-2

XhoI

CAAGTTgAAATAAGGCTAGTCCGTTATCAACTTGAAAAAGTGGCACCGAGTCGGTGCGTTCACTGCCGTATAGGCAGTCGCTGGGACGCCCGCTCGAGCAATAAACA
GTTCAAcTTTATTCCGATCAGGCAATAGTTGAACTTTTTCACCGTGGCTCAGCCACGCAAGTGACGGCATATCCGTCAGCGACCCTGCGGGCGAGCTCGTTATTTGT

sgRNA-2 | Csy4 site | Suffix

GTTGATAGGGCTTCTCCGTTACAGCCTGCGGTCCGGGTTCACTGCCGTATAGGCAGTAATTTTGTTTAACTTTAAGAAGGAGATATACATATGGTTTCGGTTATCAA
CAACTATCCCGAAGAGGCAATGTCGGACGCCAGGCCCAAGTGACGGCATATCCGTCATTAAAACAAATTGAAATTCTTCCTCTATATGTATACCAAAGCCAATAGTT

Linker_0 | Prefix | Csy4 site | RBS | mKO2

ACCAGAGATGAAAATGCGTTACTATATGGATGGTTCAGTAAATGGTCACGAATTTACTATTGAGGGCGAGGGTACGGGACGCCCATACGAGGGGCACCAGGAAATGA
TGGTCTCTACTTTTACGCAATGATATACCTACCAAGTCATTTACCAGTGCTTAAATGATAACTCCCGCTCCCATGCCCTGCGGGTATGCTCCCCGTGGTCCTTTACT

mKO2

CTTTACGCGTCACAATGGCTGAAGGCGGGCCTATGCCGTTTGCGTTCGATCTTGTTAGTCATGTCTTTTGTTACGGTCACCGTGTATTTACTAAATACCCCGAGGAA
GAAATGCGCAGTGTTACCGACTTCCGCCCGGATACGGCAAACGCAAGCTAGAACAATCAGTACAGAAAACAATGCCAGTGGCACATAAATGATTTATGGGGCTCCTT

mKO2

ATTCCAGACTATTTCAAACAAGCCTTCCCGGAAGGTTTGTCTTGGGAGCGCAGTTTAGAGTTTGAAGACGGTGGCTCGGCCAGCGTGTCAGCTCATATTAGTCTTCG
TAAGGTCTGATAAAGTTTGTTCGGAAGGGCCTTCCAAACAGAACCCTCGCGTCAAATCTCAAACTTCTGCCACCGAGCCGGTCGCACAGTCGAGTATAATCAGAAGC

mKO2

pJ2042.2 (8003 bp) (from 1178-2354 bp)

CGGCAATACATTTTATCACAAGTCAAAGTTCACCGGCGTGAACTTCCCCGCAGACGGCCCAATCATGCAGAATCAAAGTGTTGATTGGGAACCGTCCACAGAGAAGA
GCCGTTATGTAAAATAGTGTTCAGTTTCAAGTGGCCGCACTTGAAGGGGCGTCTGCCGGGTTAGTACGTCTTAGTTTCACAACTAACCCTTGGCAGGTGTCTCTTCT
mKO2

TTACAGCTTCCGATGGAGTCTTAAAGGGCGATGTAACCATGTACTTAAAATTAGAAGGGGGAGGGAACCATAAATGTCAGATGAAGACTACCTATAAGGCCGCAAAA
AATGTCGAAGGCTACCTCAGAATTTCCCGCTACATTGGTACATGAATTTTAATCTTCCCCCTCCCTTGGTATTTACAGTCTACTTCTGATGGATATTCCGGCGTTTT
mKO2

GAGATTCTTGAAATGCCCGGAGACCACTACATTGGGCATCGTTTGGTCCGTAAGACAGAAGGAAATATTACTGAACAGGTCGAAGACGCTGTGGCACACAGCATGTC
CTCTAAGAACTTTACGGGCCTCTGGTGATGTAACCCGTAGCAAACCAGGCATTCTGTCTTCCTTTATAATGACTTGTCCAGCTTCTGCGACACCGTGTGTCGTACAG
mKO2

CCGCCGTAATACTGACGCCATCACAATCCACAGCATCCTGGATTGGATTGAAGACAACTTGGAGTCGCCGTTGAGTTTAGAAAAAGTTAGTGAACGTAGTGGTTACT
GGCGGCATTATGACTGCGGTAGTGTTAGGTGTCGTAGGACCTAACCTAACTTCTGTTGAACCTCAGCGGCAACTCAAATCTTTTTCAATCACTTGCATCACCAATGA
MarA

CAAAGTGGCACCTTCAGCGCATGTTTAAGAAGGAAACGGGTCATTCATTGGGTCAATATATTCGTTCTCGCAAGATGACTGAAATTGCCCAGAAATTGAAAGAGTCT
GTTTCACCGTGGAAGTCGCGTACAAATTCTTCCTTTGCCCAGTAAGTAACCCAGTTATATAAGCAAGAGCGTTCTACTGACTTTAACGGGTCTTTAACTTTCTCAGA
MarA

AATGAACCTATTTTGTACCTGGCGGAGCGTTACGGCTTTGAAAGTCAGCAAACCCTTACACGTACCTTCAAGAATTACTTTGACGTTCCACCACACAAATATCGTAT
TTACTTGGATAAAACATGGACCGCCTCGCAATGCCGAAACTTTCAGTCGTTTGGGAATGTGCATGGAAGTTCTTAATGAAACTGCAAGGTGGTGTGTTTATAGCATA
MarA

NcoI
GACCAACATGCAGGGTGAGTCACGTTTTTTGCATCCGTTGAATCATTACAATTCCTAATAATCGCTGGGACGCCCGCCATGGTTCAGCCAAAAAACTTAAGACCGCC
CTGGTTGTACGTCCCACTCAGTGCAAAAAACGTAGGCAACTTAGTAATGTTAAGGATTATTAGCGACCCTGCGGGCGGTACCAAGTCGGTTTTTTGAATTCTGGCGG
MarA | Suffix | ECK120029...rminator

GGTCTTGTCCACTACCTTGCAGTAATGCGGTGGACAGGATCGGCGGTTTTCTTTTCTCTTCTCAATTCTTCTGACCTGTAACGAATAATAGATAGTAAAGTAGTCTC
CCAGAACAGGTGATGGAACGTCATTACGCCACCTGTCCTAGCCGCCAAAAGAAAAGAGAAGAGTTAAGAAGACTGGACATTGCTTATTATCTATCATTTCATCAGAG
ECK120029600 Terminator | Spacer 1

CGATTGAGTTTTCTCTGCCGAGTCCCACCCAGTTCTGTGATTTCAGTAAGTTGGTAATTGATACACTGTTGCGAGAACTGCTGCCTGGTAGTAGATAGGTTGTTATT
GCTAACTCAAAAGAGACGGCTCAGGGTGGGTCAAGACACTAAAGTCATTCAACCATTAACTATGTGACAACGCTCTTGACGACGGACCATCATCTATCCAACAATAA
Spacer 1

HindIII
GAGTAAGAAGGTAAAGTGAACGAAATCCCTGAAACTGAGACTGTAGAAAATAAGCTTCAGCCTGCGGTCCGGttgacggctagctcagtcctaggtacagtgctagc
CTCATTCTTCCATTTCACTTGCTTTAGGGACTTTGACTCTGACATCTTTTATTCGAAGTCGGACGCCAGGCCaactgccgatcgagtcaggatccatgtcacgatcg
Spacer 1 | Prefix | P(BBa_J23100)

BamHI
TCGCTGGGACGCCCGAGATAGCCGTTACACAGGTGACACTTATTTCAGCCTGCGGTCCGGccaCACTAGAGTCTAGCTTGAGATCGCTGGGACGCCCGGGATCCAAG
AGCGACCCTGCGGGCTCTATCGGCAATGTGTCCACTGTGAATAAAGTCGGACGCCAGGCCggtGTGATCTCAGATCGAACTCTAGCGACCCTGCGGGCCCTAGGTTC
Suffix | Linker_24 | Prefix | bs-1 | Suffix

pJ2042.2 (8003 bp) (from 2355-3531 bp)

AGATTTCTACACGATTGAGCACTGTCTCAGCCTGCGGTCCGGGTTCACTGCCGTATAGGCAGTAATTTTGTTTAACTTTAAGAAGGAGATATACATATGCGTAAAGG
TCTAAAGATGTGCTAACTCGTGACAGAGTCGGACGCCAGGCCCAAGTGACGGCATATCCGTCATTAAAACAAATTGAAATTCTTCCTCTATATGTATACGCATTTCC

Linker_10 | Prefix | Csy4 site | RBS | sfGFP

CGAAGAACTGTTTACCGGTGTGGTTCCGATTCTGGTGGAACTGGACGGCGATGTTAATGGTCATAAATTCAGTGTTCGCGGCGAAGGTGAAGGCGATGCGACGAACG
GCTTCTTGACAAATGGCCACACCAAGGCTAAGACCACCTTGACCTGCCGCTACAATTACCAGTATTTAAGTCACAAGCGCCGCTTCCACTTCCGCTACGCTGCTTGC

sfGFP

GCAAACTGACCCTGAAATTTATCTGCACCACGGGTAAACTGCCGGTCCCGTGGCCGACGCTGGTGACCACGCTGACCTATGGCGTTCAATGTTTTGCGCGTTACCCG
CGTTTGACTGGGACTTTAAATAGACGTGGTGCCCATTTGACGGCCAGGGCACCGGCTGCGACCACTGGTGCGACTGGATACCGCAAGTTACAAAACGCGCAATGGGC

sfGFP

GATCACATGAAACAGCACGACTTTTTCAAATCGGCCATGCCGGAAGGCTATGTGCAGGAACGTACGATTAGCTTTAAAGACGATGGTACGTATAAAACCCGCGCGGA
CTAGTGTACTTTGTCGTGCTGAAAAAGTTTAGCCGGTACGGCCTTCCGATACACGTCCTTGCATGCTAATCGAAATTTCTGCTACCATGCATATTTTGGGCGCGCCT

sfGFP

AGTGAAATTCGAAGGCGATACCCTGGTTAACCGTATCGAACTGAAAGGTATCGATTTCAAAGAAGACGGCAATATTCTGGGTCATAAACTGGAATATAACTTCAATT
TCACTTTAAGCTTCCGCTATGGGACCAATTGGCATAGCTTGACTTTCCATAGCTAAAGTTTCTTCTGCCGTTATAAGACCCAGTATTTGACCTTATATTGAAGTTAA

sfGFP

CCCACAACGTGTACATCACCGCGGATAAACAGAAAAACGGCATTAAAGCCAATTTCAAAATCCGCCATAATGTGGAAGATGGTAGCGTTCAGCTGGCCGACCACTAT
GGGTGTTGCACATGTAGTGGCGCCTATTTGTCTTTTTGCCGTAATTTCGGTTAAAGTTTTAGGCGGTATTACACCTTCTACCATCGCAAGTCGACCGGCTGGTGATA

sfGFP

CAGCAAAACACGCCGATTGGTGATGGCCCGGTCCTGCTGCCGGACAATCACTACCTGAGTACCCAGTCCGTGCTGTCAAAAGATCCGAACGAAAAACGTGACCACAT
GTCGTTTTGTGCGGCTAACCACTACCGGGCCAGGACGACGGCCTGTTAGTGATGGACTCATGGGTCAGGCACGACAGTTTTCTAGGCTTGCTTTTTGCACTGGTGTA

sfGFP

GGTCCTGCTGGAATTTGTGACGGCTGCGGGTATCACCCACGGCATGGACGAACTGTATAAAATGTCCCGCCGTAATACTGACGCCATCACAATCCACAGCATCCTGG
CCAGGACGACCTTAAACACTGCCGACGCCCATAGTGGGTGCCGTACCTGCTTGACATATTTTACAGGGCGGCATTATGACTGCGGTAGTGTTAGGTGTCGTAGGACC

sfGFP | MarAn20

SbfI KpnI

ATTGGATTGAAGACTAATAATCGCTGGGACGCCCGCCTGCAGGCTCGGTACCAAATTCCAGAAAAGAGGCCTCCCGAAAGGGGGGCCTTTTTTCGTTTTGGTCCTAA
TAACCTAACTTCTGATTATTAGCGACCCTGCGGGCGGACGTCCGAGCCATGGTTTAAGGTCTTTTCTCCGGAGGGCTTTCCCCCCGGAAAAAAGCAAAACCAGGATT

MarAn20 | Suffix | | L3S2P21 Terminator |

TAGATAAAGGATAGGTCTGGTAGTGTTGTTCGTTCTCGCAGGTAAATCAATAATACTCAGCAGTTCCGTAGACTTTTCAGTGGGACAGGGTAGCGATAACAGATAGA
ATCTATTTCCTATCCAGACCATCACAACAAGCAAGAGCGTCCATTTAGTTATTATGAGTCGTCAAGGCATCTGAAAAGTCACCCTGTCCCATCGCTATTGTCTATCT

Spacer 2.5

SalI

TTGTAATAAGACACAGTAGGTGCTCGTAGTTGCGTGAAGAGAACCGCTCAGGAAATCCAGTCAGAAGTATTGGTAATCGTTGAAAACTCAGTCGACCAGCCTGCGGT
AACATTATTCTGTGTCATCCACGAGCATCAACGCACTTCTCTTGGCGAGTCCTTTAGGTCAGTCTTCATAACCATTAGCAACTTTTGAGTCAGCTGGTCGGACGCCA

Spacer 2.5 | | Prefix

pJ2042.2 (8003 bp) (from 3532-4815 bp)

CCGGTTTACGGCTAGCTCAGTCCTAGGTATTATGCTAGCTCGCTGGGACGCCCGAGTGACGACTGCGAAGTAACCTCTATTTATCAGCCTGCGGTCCGGccaAGTAC
GGCCAAATGCCGATCGAGTCAGGATCCATAATACGATCGAGCGACCCTGCGGGCTCACTGCTGACGCTTCATTGGAGATAAATAGTCGGACGCCAGGCCggtTCATG
P(BBa_J23150) | Suffix | Linker_25 | Prefix

TTAGTACACACTGATTCGCTGGGACGCCCGGTAGTGCTTATCAGACCCAATACTGTTGAACAGCCTGCGGTCCGGGTTCACTGCCGTATAGGCAGTCTCAAGCTAGA
AATCATGTGTGACTAAGCGACCCTGCGGGCCATCACGAATAGTCTGGGTTATGACAACTTGTCGGACGCCAGGCCCAAGTGACGGCATATCCGTCAGAGTTCGATCT
bs-2 | Suffix | Linker_17 | Prefix | Csy4 site | sgRNA-1

CTCTAGTGGTTTcAGAGCTATGCTGGAAACAGCATAGCAAGTTgAAATAAGGCTAGTCCGTTATCAACTTGAAAAAGTGGCACCGAGTCGGTGCGTTCACTGCCGTA
GAGATCACCAAAgTCTCGATACGACCTTTGTCGTATCGTTCAAcTTTATTCCGATCAGGCAATAGTTGAACTTTTTCACCGTGGCTCAGCCACGCAAGTGACGGCAT
sgRNA-1 | Csy...te

AatII

TAGGCAGTCGCTGGGACGCCCGGACGTCCTATTACACTCGTCGTTGGAAACTGAAGATCAGCCTGCGGTCCGGGTTCACTGCCGTATAGGCAGTAATTTTGTTTAAC
ATCCGTCAGCGACCCTGCGGGCCTGCAGGATAATGTGAGCAGCAACCTTTGACTTCTAGTCGGACGCCAGGCCCAAGTGACGGCATATCCGTCATTAAAACAAATTG
Suffix | Linker_11 | Prefix | Csy4 site | RBS

TTTAAGAAGGAGATATACATATGAATCAGTCATTCATCTCGGACATCTTATATGCCGACATCGAATCGAAGGCTAAGGAACTTACAGTCAATTCCAACAATACTGTC
AAATTCTTCCTCTATATGTATACTTAGTCAGTAAGTAGAGCCTGTAGAATATACGGCTGTAGCTTAGCTTCCGATTCCTTGAATGTCAGTTAAGGTTGTTATGACAG
RBS | RepA70

CAGCCGGTCGCGCTTATGCGCTTAGGAGTTTTCGTTCCCAAACCTTCCAAGAGCAAAGGAGAAAGTAAGGAAATTGACGCCACCAAAGCCTTCTCTCAACTGGAGAT
GTCGGCCAGCGCGAATACGCGAATCCTCAAAAGCAAGGGTTTGGAAGGTTCTCGTTTCCTCTTTCATTCCTTTAACTGCGGTGGTTTCGGAAGAGAGTTGACCTCTA
RepA70

TGCTAAAGCAGAGGGCatggttagtaaaggagaagaaaataacatggcaCTGATTAAGGAGAACATGCACATGAAGCTGTACATGGAGGGCACCGTGAACAACCACC
ACGATTTCGTCTCCCGtaccaatcatttcctcttcttttattgtaccgtGACTAATTCCTCTTGTACGTGTACTTCGACATGTACCTCCCGTGGCACTTGTTGGTGG
RepA70 | mKate2

ACTTCAAGTGCACATCCGAGGGCGAAGGCAAGCCCTACGAGGGCACCCAGACCATGAGAATCAAGgccGTCGAGGGCGGCCCTCTCCCCTTCGCCTTCGACATCCTG
TGAAGTTCACGTGTAGGCTCCCGCTTCCGTTCGGGATGCTCCCGTGGGTCTGGTACTCTTAGTTCcggCAGCTCCCGCCGGGAGAGGGGAAGCGGAAGCTGTAGGAC
mKate2

GCTACCAGCTTCATGTACGGCAGCAAAACCTTCATCAACCACACCCAGGGCATCCCCGACTTCTTTAAGCAGTCCTTCCCTGAGGGCTTCACATGGGAGAGAGTCAC
CGATGGTCGAAGTACATGCCGTCGTTTTGGAAGTAGTTGGTGTGGGTCCCGTAGGGGCTGAAGAAATTCGTCAGGAAGGGACTCCCGAAGTGTACCCTCTCTCAGTG
mKate2

CACATACGAAGACGGGGGCGTGCTGACCGCTACCCAGGACACCAGCCTCCAGGACGGCTGCCTCATCTACAACGTCAAGATCAGAGGGGTGAACTTCCCATCCAACG
GTGTATGCTTCTGCCCCCGCACGACTGGCGATGGGTCCTGTGGTCGGAGGTCCTGCCGACGGAGTAGATGTTGCAGTTCTAGTCTCCCCACTTGAAGGGTAGGTTGC
mKate2

GCCCTGTGATGCAGAAGAAAACACTCGGCTGGGAGGCCTCCACCGAGaccCTGTACCCCGCTGACGGCGGCCTGGAAGGCAGAgcCGACATGGCCCTGAAGCTCGTG
CGGGACACTACGTCTTCTTTTGTGAGCCGACCCTCCGGAGGTGGCTCtggGACATGGGGCGACTGCCGCCGGACCTTCCGTCTcgGCTGTACCGGGACTTCGAGCAC
mKate2

GGCGGGGGCCACCTGATCTGCAACTTGAAGACCACATACAGATCCAAGAAACCCGCTAAGAACCTCAAGATGCCCGGCGTCTACTATGTGGACAGAAGACTGGAAAG
CCGCCCCCGGTGGACTAGACGTTGAACTTCTGGTGTATGTCTAGGTTCTTTGGGCGATTCTTGGAGTTCTACGGGCCGCAGATGATACACCTGTCTTCTGACCTTTC
mKate2

pJ2042.2 (8003 bp) (from 4816-6099 bp)

AATCAAGGAGGCCGACAAAGAGACCTACGTCGAGCAGCACGAGGTGGCTGTGGCCAGATACTGCGACCTCCCTAGCAAACTGGGGCACAgAgtctaATAATCGCTGG
TTAGTTCCTCCGGCTGTTTCTCTGGATGCAGCTCGTCGTGCTCCACCGACACCGGTCTATGACGCTGGAGGGATCGTTTGACCCCGTGTcTcagatTATTAGCGACC
mKate2

NotI

GACGCCCGGCGGCCGCggaaacacagAAAAAAGCCCGCACCTGACAGTGCGGGCTTTTTTTTTcgaccaaaggTAGCGAACGACGAGTCACTGTTGAGGATAAATAC
CTGCGGGCCGCCGGCGcctttgtgtcTTTTTTCGGGCGTGGACTGTCACGCCCGAAAAAAAAAgctggtttccATCGCTTGCTGCTCAGTGACAACTCCTATTTATG
ECK120033737 Terminator
Linker_8

KasI BbvCI AscI

TTTCTCTACTAGGCGCCTGTTACACAGGTCCTCAGCGGCGCGCCTTTGTCGGTGAACGCTCTCCTGAGTAGGACAAATCCGCCGGGAGCGGATTTGAACGTTGTGAA
AAAGAGATGATCCGCGGACAATGTGTCCAGGAGTCGCCGCGCGGAAACAGCCACTTGCGAGAGGACTCATCCTGTTTAGGCGGCCCTCGCCTAAACTTGCAACACTT
Li...8
Spa...15

GCAACGGCCCGGAGGGTGGCGGGCAGGACGCCCGCCATAAACTGCCAGGCATCAAACTAAGCAGAAGGCCATCCTGACGGATGGCCTTTTTGCGTTTCAGATCTACC
CGTTGCCGGGCCTCCCACCGCCCGTCCTGCGGGCGGTATTTGACGGTCCGTAGTTTGATTCGTCTTCCGGTAGGACTGCCTACCGGAAAAACGCAAAGTCTAGATGG

GGTaaaccagcaatagacataagcggctatttaacgaccctgccctgaaccgacgacaagctgacgaccgggtctccgcaagtggcacttttcggggaaatgtgcgc
CCAtttggtcgttatctgtattcgccgataaattgctgggacgggacttggctgctgttcgactgctggcccagaggcgttcaccgtgaaaagcccctttacacgcg

ggaacccctatttgtttatttttctaaatacattcaaatatgtatccgctcatgaattaattcttagaaaaactcatcgagcatcaaatgaaactgcaatttattca
ccttggggataaacaaataaaaagatttatgtaagtttatacataggcgagtacttaattaagaatctttttgagtagctcgtagtttactttgacgttaaataagt
KanR

tatcaggattatcaataccatatttttgaaaaagccgtttctgtaatgaaggagaaaactcaccgaggcagttccataggatggcaagatcctggtatcggtctgcg
atagtcctaatagttatggtataaaaactttttcggcaaagacattacttcctcttttgagtggctccgtcaaggtatcctaccgttctaggaccatagccagacgc
KanR

attccgactcgtccaacatcaatacaacctattaatttcccctcgtcaaaaataaggttatcaagtgagaaatcaccatgagtgacgactgaatccggtgagaatgg
taaggctgagcaggttgtagttatgttggataattaaaggggagcagtttttattccaatagttcactctttagtggtactcactgctgacttaggccactcttacc
KanR

caaaagtttatgcatttctttccagacttgttcaacaggccagccattacgctcgtcatcaaaatcactcgcatcaaccaaaccgttattcattcgtgattgcgcct
gttttcaaatacgtaaagaaaggtctgaacaagttgtccggtcggtaatgcgagcagtagttttagtgagcgtagttggtttggcaataagtaagcactaacgcgga
KanR

gagcgagacgaaatacgcggtcgctgttaaaaggacaattacaaacaggaatcgaatgcaaccggcgcaggaacactgccagcgcatcaacaatattttcacctgaa
ctcgctctgctttatgcgccagcgacaattttcctgttaatgtttgtccttagcttacgttggccgcgtccttgtgacggtcgcgtagttgttataaaagtggactt
KanR

tcaggatattcttctaatacctggaatgctgttttcccggggatcgcagtggtgagtaaccatgcatcatcaggagtacggataaaatgcttgatggtcggaagagg
agtcctataagaagattatggaccttacgacaaaagggcccctagcgtcaccactcattggtacgtagtagtcctcatgcctattttacgaactaccagccttctcc
KanR

cataaattccgtcagccagtttagtctgaccatctcatctgtaacatcattggcaacgctacctttgccatgtttcagaaacaactctggcgcatcgggcttcccat
gtatttaaggcagtcggtcaaatcagactggtagagtagacattgtagtaaccgttgcgatggaaacggtacaaagtctttgttgagaccgcgtagcccgaagggta
KanR

pJ2042.2 (8003 bp) (from 6100-7383 bp)

acaatcgatagattgtcgcacctgattgcccgacattatcgcgagcccatttatacccatataaatcagcatccatgttggaatttaatcgcggcctagagcaagac
tgttagctatctaacagcgtggactaacgggctgtaatagcgctcgggtaaatatgggtatatttagtcgtaggtacaaccttaaattagcgccggatctcgttctg
KanR

gtttcccgttgaatatggctcatactcttcctttttcaatattattgaagcatttatcagggttattgtctcatgagcggatacatatttgaatgtatttagaaaaa
caaagggcaacttataccgagtatgagaaggaaaaagttataataacttcgtaaatagtcccaataacagagtactcgcctatgtataaacttacataaatcttttt
KanR

taaacaaataggcatgctagcgcagaaacgtcctagaagatgccaggaggatacttagcagagagacaataaggccggagcgaagccgtttttccataggctccgcc
atttgtttatccgtacgatcgcgtctttgcaggatcttctacggtcctcctatgaatcgtctctctgttattccggcctcgcttcggcaaaaaggtatccgaggcgg
ColA ori

cccctgacgaacatcacgaaatctgacgctcaaatcagtggtggcgaaacccgacaggactataaagataccaggcgtttccccctgatggctccctcttgcgctct
ggggactgcttgtagtgctttagactgcgagtttagtcaccaccgctttgggctgtcctgatatttctatggtccgcaaagggggactaccgagggagaacgcgaga
ColA ori

cctgttcccgtcctgcggcgtccgtgttgtggtggaggctttacccaaatcaccacgtcccgttccgtgtagacagttcgctccaagctgggctgtgtgcaagaacc
ggacaagggcaggacgccgcaggcacaacaccacctccgaaatgggtttagtggtgcagggcaaggcacatctgtcaagcgaggttcgacccgacacacgttcttgg
ColA ori

ccccgttcagcccgactgctgcgccttatccggtaactatcatcttgagtccaacccggaaagacacgacaaaacgccactggcagcagccattggtaactgagaat
ggggcaagtcgggctgacgacgcggaataggccattgatagtagaactcaggttgggcctttctgtgctgttttgcggtgaccgtcgtcggtaaccattgactctta
ColA ori

tagtggatttagatatcgagagtcttgaagtggtggcctaacagaggctacactgaaaggacagtatttggtatctgcgctccactaaagccagttaccaggttaag
atcacctaaatctatagctctcagaacttcaccaccggattgtctccgatgtgactttcctgtcataaaccatagacgcgaggtgatttcggtcaatggtccaattc
ColA ori

cagttccccaactgacttaaccttcgatcaaaccgcctccccaggcggttttttcgtttacagagcaggagattacgacgatcgtaaaaggatctcaagaagatcct
gtcaaggggttgactgaattggaagctagtttggcggaggggtccgccaaaaaagcaaatgtctcgtcctctaatgctgctagcattttcctagagttcttctagga
ColA ori

ttacggattcccgacaccatcactctagatttcagtgcaatttatctcttcaaatgtagcacctgaagtcagccccatacgatataagttgtaattctcatgttagt
aatgcctaagggctgtggtagtgagatctaaagtcacgttaaatagagaagtttacatcgtggacttcagtcggggtatgctatattcaacattaagagtacaatca
ColA ori

catgccccgcgcccaccggaaggagctgactgggttgCTCCTAgGGTCTGATTCGTTACCAATTATGACAACTTGACGGCTACATCATTCACTTTTTCTTCACAACC
gtacggggcgcgggtggccttcctcgactgacccaacGAGGATcCCAGACTAAGCAATGGTTAATACTGTTGAACTGCCGATGTAGTAAGTGAAAAAGAAGTGTTGG
araC

GGCACGGAACTCGCTCGGGCTGGCCCCGGTGCATTTTTTAAATACCCGCGAGAAATAGAGTTGATCGTCAAAACCAACATTGCGACCGACGGTGGCGATAGGCATCC
CCGTGCCTTGAGCGAGCCCGACCGGGGCCACGTAAAAAATTTATGGGCGCTCTTTATCTCAACTAGCAGTTTTGGTTGTAACGCTGGCTGCCACCGCTATCCGTAGG
araC

GGGTGGTGCTCAAAAGCAGCTTCGCCTGGCTGATACGTTGGTCCTCGCGCCAGCTTAAGACGCTAATCCCTAACTGCTGGCGGAAAAGATGTGACAGACGCGACGGC
CCCACCACGAGTTTTCGTCGAAGCGGACCGACTATGCAACCAGGAGCGCGGTCGAATTCTGCGATTAGGGATTGACGACCGCCTTTTCTACACTGTCTGCGCTGCCG
araC

pJ2042.2 (8003 bp) (from 7384-8003 bp)

GACAAGCAAACATGCTGTGCGACGCTGGCGATATCAAAATTGCTGTCTGCCAGGTGATCGCTGATGTACTGACAAGCCTCGCGTACCCGATTATCCATCGGTGGATG
CTGTTCGTTTGTACGACACGCTGCGACCGCTATAGTTTTAACGACAGACGGTCCACTAGCGACTACATGACTGTTCGGAGCGCATGGGCTAATAGGTAGCCACCTAC
araC

GAGCGACTCGTTAATCGCTTCCATGCGCCGCAGTAACAATTGCTCAAGCAGATTTATCGCCAGCAGCTCCGAATAGCGCCCTTCCCCTTGCCCGGCGTTAATGATTT
CTCGCTGAGCAATTAGCGAAGGTACGCGGCGTCATTGTTAACGAGTTCGTCTAAATAGCGGTCGTCGAGGCTTATCGCGGGAAGGGGAACGGGCCGCAATTACTAAA
araC

GCCCAAACAGGTCGCTGAAATGCGGCTGGTGCGCTTCATCCGGGCGAAAGAACCCCGTATTGGCAAATATTGACGGCCAGTTAAGCCATTCATGCCAGTAGGCGCGC
CGGGTTTGTCCAGCGACTTTACGCCGACCACGCGAAGTAGGCCCGCTTTCTTGGGGCATAACCGTTTATAACTGCCGGTCAATTCGGTAAGTACGGTCATCCGCGCG
araC

GGACGAAAGTAAACCCACTGGTGATACCATTCGCGAGCCTCCGGATGACGACCGTAGTGATGAATCTCTCCTGGCGGGAACAGCAAAATATCACCCGGTCGGCAAAC
CCTGCTTTCATTTGGGTGACCACTATGGTAAGCGCTCGGAGGCCTACTGCTGGCATCACTACTTAGAGAGGACCGCCCTTGTCGTTTTATAGTGGGCCAGCCGTTTG
araC

AAATTCTCGTCCCTGATTTTTCACCACCCCCTGACCGCGAATGGTGAGATTGAGAATATAACCTTTCATTCCCAGCGGTCGGTCGATAAAAAAATCGAGATAACCGT
TTTAAGAGCAGGGACTAAAAAGTGGTGGGGGACTGGCGCTTACCACTCTAACTCTTATATTGGAAAGTAAGGGTCGCCAGCCAGCTATTTTTTTAGCTCTATTGGCA
araC

TGGCCTCAATCGGCGTTAAACCCGCCACCAGATGGGCATTAAACGAGTATCCCGGCAGCAGGGGATCATTTTGCGCTTCAGCCAT
ACCGGAGTTAGCCGCAATTTGGGCGGTGGTCTACCCGTAATTTGCTCATAGGGCCGTCGTCCCCTAGTAAAACGCGAAGTCGGTA
araC

(from 1-1177 bp)

pJ2042.2_Brocc (6140 bp)

```
ACTTTTCATACTCCCGCCATTCAGAGAAGAAACCAATTGTCCATATTGCATCAGACATTGCCGTCACTGCGTCTTTTACTGGCTCTTCTCGCTAACCAAACCGGTAA
TGAAAAGTATGAGGGCGGTAAGTCTCTTCTTTGGTTAACAGGTATAACGTAGTCTGTAACGGCAGTGACGCAGAAAATGACCGAGAAGAGCGATTGGTTTGGCCATT

CCCCGCTTATTAAAAGCATTCTGTAACAAAGCGGGACCAAAGCCATGACAAAAACGCGTAACAAAAGTGTCTATAATCACGGCAGAAAAGTCCACATTGATTATTTG
GGGGCGAATAATTTTCGTAAGACATTGTTTCGCCCTGGTTTCGGTACTGTTTTTGCGCATTGTTTTCACAGATATTAGTGCCGTCTTTTCAGGTGTAACTAATAAAC

EcoRI
CACGGCGTCACACTTTGCTATGCCATAGCATTTTTATCCATAAGATTAGCGGtTCCTACCTGACGCTTTTTATCGCAACTCTCTACTGTTTCTCCATACCGAATTCA
GTGCCGCAGTGTGAAACGATACGGTATCGTAAAAATAGGTATTCTAATCGCCaAGGATGGACTGCGAAAAATAGCGTTGAGAGATGACAAAGAGGTATGGCTTAAGT
P(BAD) promoter

SacI
TAGGATAGATTCTGGAAACTTTACCGTCCGAGCTCCAGCCTGCGGTCCGGGTTCACTGCCGTATAGGCAGAAGCTAGACTCTAGTGGTTTcAGAGCTATGCTGGAAA
ATCCTATCTAAGACCTTTGAAATGGCAGGCTCGAGGTCGGACGCCAGGCCCAAGTGACGGCATATCCGTCTTCGATCTGAGATCACCAAAgTCTCGATACGACCTTT
Linker_14  Prefix  Csy4 site  sgRNA-1t4

CAGCATAGCAAGTTgAAATAAGGCTAGTCCGTTATCAACTTGAAAAAGTGGCACCGAGTCGGTGCGTTCACTGCCGTATAGGCAGTCGCTGGGACGCCCGGGGACTA
GTCGTATCGTTCAAcTTTATTCCGATCAGGCAATAGTTGAACTTTTTCACCGTGGCTCAGCCACGCAAGTGACGGCATATCCGTCAGCGACCCTGCGGGCCCCTGAT
sgRNA-1t4  Csy4 site  Suffix

CACTTACGAAACTATTGATTGCTCAGCCTGCGGTCCGGGTTCACTGCCGTATAGGCAGATCAGTGTGTACTAAGTACTGTTTcAGAGCTATGCTGGAAACAGCATAG
GTGAATGCTTTGATAACTAACGAGTCGGACGCCAGGCCCAAGTGACGGCATATCCGTCTAGTCACACATGATTCATGACAAAgTCTCGATACGACCTTTGTCGTATC
Linker_1  Prefix  Csy4 site  sgRNA-2

XhoI
CAAGTTgAAATAAGGCTAGTCCGTTATCAACTTGAAAAAGTGGCACCGAGTCGGTGCGTTCACTGCCGTATAGGCAGTCGCTGGGACGCCCGCTCGAGCAATAAACA
GTTCAAcTTTATTCCGATCAGGCAATAGTTGAACTTTTTCACCGTGGCTCAGCCACGCAAGTGACGGCATATCCGTCAGCGACCCTGCGGGCGAGCTCGTTATTTGT
sgRNA-2  Csy4 site  Suffix

NcoI
GTTGATAGGGCTTCTCCGTTACCATGGTTCAGCCAAAAAACTTAAGACCGCCGGTCTTGTCCACTACCTTGCAGTAATGCGGTGGACAGGATCGGCGGTTTTCTTTT
CAACTATCCCGAAGAGGCAATGGTACCAAGTCGGTTTTTTGAATTCTGGCGGCCAGAACAGGTGATGGAACGTCATTACGCCACCTGTCCTAGCCGCCAAAAGAAAA
Linker_0  ECK120029600 Terminator

CTCTTCTCAATTCTTCTGACCTGTAACGAATAATAGATAGTAAAGTAGTCTCCGATTGAGTTTTCTCTGCCGAGTCCCACCCAGTTCTGTGATTTCAGTAAGTTGGT
GAGAAGAGTTAAGAAGACTGGACATTGCTTATTATCTATCATTTCATCAGAGGCTAACTCAAAAGAGACGGCTCAGGGTGGGTCAAGACACTAAAGTCATTCAACCA
EC...r  Spacer 1

HindIII
AATTGATACACTGTTGCGAGAACTGCTGCCTGGTAGTAGATAGGTTGTTATTGAGTAAGAAGGTAAAGTGAACGAAATCCCTGAAACTGAGACTGTAGAAAATAAGC
TTAACTATGTGACAACGCTCTTGACGACGGACCATCATCTATCCAACAATAACTCATTCTTCCATTTCACTTGCTTTAGGGACTTTGACTCTGACATCTTTTATTCG
Spacer 1

TTCAGCCTGCGGTCCGGttgacggctagctcagtcctaggtacagtgctagcTCGCTGGGACGCCCGAGATAGCCGTTACACAGGTGACACTTATTTCAGCCTGCGG
AAGTCGGACGCCAGGCCaactgccgatcgagtcaggatccatgtcacgatcgAGCGACCCTGCGGGCTCTATCGGCAATGTGTCCACTGAATAAAGTCGGACGCC
Prefix  P(BBa_J23100)  Suffix  Linker_24  Pr...x
```

pJ2042.2_Brocc (6140 bp) (from 1178-2354 bp)

BamHI

TCCGGccaCACTAGAGTCTAGCTTGAGATCGCTGGGACGCCCGGGATCCAAGAGATTTCTACACGATTGAGCACTGTCTCAGCCTGCGGTCCGGGTTCACTGCCGTA
AGGCCggtGTGATCTCAGATCGAACTCTAGCGACCCTGCGGGCCCTAGGTTCTCTAAAGATGTGCTAACTCGTGACAGAGTCGGACGCCAGGCCCAAGTGACGGCAT

bs-1 | Suffix | Linker_10 | Prefix | Csy...te

TAGGCAGttgccatgtgtatgtgggagacggtcgggtccatctgagacggtcgggtccagatattcgtatctgtcgagtagagtgtgggctcagatgtcgagtagag
ATCCGTCaacggtacacatacaccctctgccagcccaggtagactctgccagcccaggtctataagcatagacagctcatctcacacccgagtctacagctcatctc

F30-2xdBroccoli

tgtgggctcccacatactctgatgatccagacggtcgggtccatctgagacggtcgggtccagatattcgtatctgtcgagtagagtgtgggctcagatgtcgagta
acacccgagggtgtatgagactactaggtctgccagcccaggtagactctgccagcccaggtctataagcatagacagctcatctcacacccgagtctacagctcat

F30-2xdBroccoli

gagtgtgggctggatcattcatggcaaGTTCACTGCCGTATAGGCAGttgccatgtgtatgtgggagacggtcgggtccatctgagacggtcgggtccagatattcg
ctcacacccgacctagtaagtaccgttCAAGTGACGGCATATCCGTCaacggtacacatacaccctctgccagcccaggtagactctgccagcccaggtctataagc

F30-2xdBroccoli | Csy4 site | F30-2xdBroccoli

tatctgtcgagtagagtgtgggctcagatgtcgagtagagtgtgggctcccacatactctgatgatccagacggtcgggtccatctgagacggtcgggtccagatat
atagacagctcatctcacacccgagtctacagctcatctcacacccgagggtgtatgagactactaggtctgccagcccaggtagactctgccagcccaggtctata

F30-2xdBroccoli

tcgtatctgtcgagtagagtgtgggctcagatgtcgagtagagtgtgggctggatcattcatggcaaGTTCACTGCCGTATAGGCAGTCGCTGGGACGCCCGAGAGC
agcatagacagctcatctcacacccgagtctacagctcatctcacacccgacctagtaagtaccgttCAAGTGACGGCATATCCGTCAGCGACCCTGCGGGCTCTCG

F30-2xdBroccoli | Csy4 site | Suffix

CGAATCGCACTTATTTACAGTAGTTCAGCCTGCGGTCCGGGTTCACTGCCGTATAGGCAGttgccatgtgtatgtgggagacggtcgggtccatctgagacggtcgg
GCTTAGCGTGAATAAATGTCATCAAGTCGGACGCCAGGCCCAAGTGACGGCATATCCGTCaacggtacacatacaccctctgccagcccaggtagactctgccagcc

Linker_21 | Prefix | Csy4 site | F30-2xdBroccoli

gtccagatattcgtatctgtcgagtagagtgtgggctcagatgtcgagtagagtgtgggctcccacatactctgatgatccagacggtcgggtccatctgagacggt
caggtctataagcatagacagctcatctcacacccgagtctacagctcatctcacacccgagggtgtatgagactactaggtctgccagcccaggtagactctgcca

F30-2xdBroccoli

cgggtccagatattcgtatctgtcgagtagagtgtgggctcagatgtcgagtagagtgtgggctggatcattcatggcaaGTTCACTGCCGTATAGGCAGttgccat
gcccaggtctataagcatagacagctcatctcacacccgagtctacagctcatctcacacccgacctagtaagtaccgttCAAGTGACGGCATATCCGTCaacggta

F30-2xdBroccoli | Csy4 site

gtgtatgtgggagacggtcgggtccatctgagacggtcgggtccagatattcgtatctgtcgagtagagtgtgggctcagatgtcgagtagagtgtgggctcccaca
cacatacaccctctgccagcccaggtagactctgccagcccaggtctataagcatagacagctcatctcacacccgagtctacagctcatctcacacccgagggtgt

F30-2xdBroccoli

tactctgatgatccagacggtcgggtccatctgagacggtcgggtccagatattcgtatctgtcgagtagagtgtgggctcagatgtcgagtagagtgtgggctgga
atgagactactaggtctgccagcccaggtagactctgccagcccaggtctataagcatagacagctcatctcacacccgagtctacagctcatctcacacccgacct

F30-2xdBroccoli

pJ2042.2_Brocc (6140 bp) (from 2355-3531 bp)

SbfI KpnI

tcattcatggcaaGTTCACTGCCGTATAGGCAGTCGCTGGGACGCCCGCCTGCAGGCTCGGTACCAAATTCCAGAAAAGAGGCCTCCCGAAAGGGGGGCCTTTTTTC
agtaagtaccgttCAAGTGACGGCATATCCGTCAGCGACCCTGCGGGCGGACGTCCGAGCCATGGTTTAAGGTCTTTTCTCCGGAGGGCTTTCCCCCCGGAAAAAAG

F30...li | Csy4 site | Suffix | L3S2P21 Terminator

GTTTTGGTCCTAATAGATAAAGGATAGGTCTGGTAGTGTTGTTCGTTCTCGCAGGTAAATCAATAATACTCAGCAGTTCCGTAGACTTTTCAGTGGGACAGGGTAGC
CAAAACCAGGATTATCTATTTCCTATCCAGACCATCACAACAAGCAAGAGCGTCCATTTAGTTATTATGAGTCGTCAAGGCATCTGAAAAGTCACCCTGTCCCATCG

L3...r | Spacer 2.5

SalI

GATAACAGATAGATTGTAATAAGACACAGTAGGTGCTCGTAGTTGCGTGAAGAGAACCGCTCAGGAAATCCAGTCAGAAGTATTGGTAATCGTTGAAAACTCAGTCG
CTATTGTCTATCTAACATTATTCTGTGTCATCCACGAGCATCAACGCACTTCTCTTGGCGAGTCCTTTAGGTCAGTCTTCATAACCATTAGCAACTTTTGAGTCAGC

Spacer 2.5

ACCAGCCTGCGGTCCGGTTTACGGCTAGCTCAGTCCTAGGTATTATGCTAGCTCGCTGGGACGCCCGAGTGACGACTGCGAAGTAACCTCTATTTATCAGCCTGCGG
TGGTCGGACGCCAGGCCAAATGCCGATCGAGTCAGGATCCATAATACGATCGAGCGACCCTGCGGGCTCACTGCTGACGCTTCATTGGAGATAAATAGTCGGACGCC

Prefix | P(BBa_J23150) | Suffix | Linker_25 | Pr...x

TCCGGccaAGTACTTAGTACACACTGATTCGCTGGGACGCCCGGTAGTGCTTATCAGACCCAATACTGTTGAACAGCCTGCGGTCCGGGTTCACTGCCGTATAGGCA
AGGCCggtTCATGAATCATGTGTGACTAAGCGACCCTGCGGGCCATCACGAATAGTCTGGGTTATGACAACTTGTCGGACGCCAGGCCCAAGTGACGGCATATCCGT

bs-2 | Suffix | Linker_17 | Prefix | Csy4 site

GTCTCAAGCTAGACTCTAGTGGTTTcAGAGCTATGCTGGAAACAGCATAGCAAGTTgAAATAAGGCTAGTCCGTTATCAACTTGAAAAAGTGGCACCGAGTCGGTGC
CAGAGTTCGATCTGAGATCACCAAAgTCTCGATACGACCTTTGTCGTATCGTTCAAcTTTATTCCGATCAGGCAATAGTTGAACTTTTTCACCGTGGCTCAGCCACG

sgRNA-1

AatII NotI

GTTCACTGCCGTATAGGCAGTCGCTGGGACGCCCGGACGTCCTATTACACTCGTCGTTGGAAACTGAAGATGCGGCCGCggaaacacagAAAAAAGCCCGCACCTGA
CAAGTGACGGCATATCCGTCAGCGACCCTGCGGGCCTGCAGGATAATGTGAGCAGCAACCTTTGACTTCTACGCCGGCGcctttgtgtcTTTTTTCGGGCGTGGACT

Csy4 site | Suffix | Linker_11 | ECK120033737 Terminator

KasI BbvCI AscI

CAGTGCGGGCTTTTTTTTTcgaccaaaggTAGCGAACGACGAGTCACTGTTGAGGATAAATACTTTCTCTACTAGGCGCCTGTTACACAGGTCCTCAGCGGCGCGCC
GTCACGCCCGAAAAAAAAAgctggtttccATCGCTTGCTGCTCAGTGACAACTCCTATTTATGAAAGAGATGATCCGCGGACAATGTGTCCAGGAGTCGCCGCGCGG

ECK120033737 Terminator | Linker_8 | Spa...15

TTTGTCGGTGAACGCTCTCCTGAGTAGGACAAATCCGCCGGGAGCGGATTTGAACGTTGTGAAGCAACGGCCCGGAGGGTGGCGGGCAGGACGCCCGCCATAAACTG
AAACAGCCACTTGCGAGAGGACTCATCCTGTTTAGGCGGCCCTCGCCTAAACTTGCAACACTTCGTTGCCGGGCCTCCCACCGCCCGTCCTGCGGGCGGTATTTGAC

CCAGGCATCAAACTAAGCAGAAGGCCATCCTGACGGATGGCCTTTTTGCGTTTCAGATCTACCGGTaaaccagcaatagacataagcggctatttaacgaccctgcc
GGTCCGTAGTTTGATTCGTCTTCCGGTAGGACTGCCTACCGGAAAAACGCAAAGTCTAGATGGCCAtttggtcgttatctgtattcgccgataaattgctgggacgg

ctgaaccgacgacaagctgacgaccgggtctccgcaagtggcacttttcggggaaatgtgcgcggaacccctatttgtttatttttctaaatacattcaaatatgta
gacttggctgctgttcgactgctggcccagaggcgttcaccgtgaaaagcccctttacacgcgccttggggataaacaaataaaaagatttatgtaagtttatacat

pJ2042.2_Brocc (6140 bp) (from 3532-4815 bp)

tccgctcatgaattaattcttagaaaaactcatcgagcatcaaatgaaactgcaatttattcatatcaggattatcaataccatatttttgaaaaagccgtttctgt
aggcgagtacttaattaagaatctttttgagtagctcgtagtttactttgacgttaaataagtatagtcctaatagttatggtataaaaactttttcggcaaagaca
KanR

aatgaaggagaaaactcaccgaggcagttccataggatggcaagatcctggtatcggtctgcgattccgactcgtccaacatcaatacaacctattaatttcccctc
ttacttcctcttttgagtggctccgtcaaggtatcctaccgttctaggaccatagccagacgctaaggctgagcaggttgtagttatgttggataattaaaggggag
KanR

gtcaaaaataaggttatcaagtgagaaatcaccatgagtgacgactgaatccggtgagaatggcaaaagtttatgcatttctttccagacttgttcaacaggccagc
cagtttttattccaatagttcactctttagtggtactcactgctgacttaggccactcttaccgttttcaaatacgtaaagaaaggtctgaacaagttgtccggtcg
KanR

cattacgctcgtcatcaaaatcactcgcatcaaccaaaccgttattcattcgtgattgcgcctgagcgagacgaaatacgcggtcgctgttaaaaggacaattacaa
gtaatgcgagcagtagttttagtgagcgtagttggtttggcaataagtaagcactaacgcggactcgctctgctttatgcgccagcgacaattttcctgttaatgtt
KanR

acaggaatcgaatgcaaccggcgcaggaacactgccagcgcatcaacaatattttcacctgaatcaggatattcttctaatacctggaatgctgttttcccggggat
tgtccttagcttacgttggccgcgtccttgtgacggtcgcgtagttgttataaaagtggacttagtcctataagaagattatggaccttacgacaaaagggccccta
KanR

cgcagtggtgagtaaccatgcatcatcaggagtacggataaaatgcttgatggtcggaagaggcataaattccgtcagccagtttagtctgaccatctcatctgtaa
gcgtcaccactcattggtacgtagtagtcctcatgcctattttacgaactaccagccttctccgtatttaaggcagtcggtcaaatcagactggtagagtagacatt
KanR

catcattggcaacgctacctttgccatgtttcagaaacaactctggcgcatcgggcttcccatacaatcgatagattgtcgcacctgattgcccgacattatcgcga
gtagtaaccgttgcgatggaaacggtacaaagtctttgttgagaccgcgtagcccgaagggtatgttagctatctaacagcgtggactaacgggctgtaatagcgct
KanR

gcccatttatacccatataaatcagcatccatgttggaatttaatcgcggcctagagcaagacgtttcccgttgaatatggctcatactcttcctttttcaatatta
cgggtaaatatgggtatatttagtcgtaggtacaaccttaaattagcgccggatctcgttctgcaaagggcaacttataccgagtatgagaaggaaaaagttataat
KanR

ttgaagcatttatcagggttattgtctcatgagcggatacatatttgaatgtatttagaaaaataaacaaataggcatgctagcgcagaaacgtcctagaagatgcc
aacttcgtaaatagtcccaataacagagtactcgcctatgtataaacttacataaatctttttatttgtttatccgtacgatcgcgtctttgcaggatcttctacgg
ColA ori

aggaggatacttagcagagagacaataaggccggagcgaagccgtttttccataggctccgcccccctgacgaacatcacgaaatctgacgctcaaatcagtggtgg
tcctcctatgaatcgtctctctgttattccggcctcgcttcggcaaaaaggtatccgaggcggggggactgcttgtagtgctttagactgcgagtttagtcaccacc
ColA ori

cgaaacccgacaggactataaagataccaggcgtttccccctgatggctccctcttgcgctctcctgttcccgtcctgcggcgtccgtgttgtggtggaggctttac
gctttgggctgtcctgatatttctatggtccgcaaaggggggactaccgagggagaacgcgagaggacaagggcaggacgccgcaggcacaacaccacctccgaaatg
ColA ori

ccaaatcaccacgtcccgttccgtgtagacagttcgctccaagctgggctgtgtgcaagaacccccgttcagcccgactgctgcgccttatccggtaactatcatc
ggtttagtggtgcagggcaaggcacatctgtcaagcgaggttcgacccgacacacgttcttgggggcaagtcgggctgacgacgcggaataggccattgatagtag
ColA ori

pJ2042.2_Brocc (6140 bp) (from 4816-6099 bp)

```
ttgagtccaacccggaaagacacgacaaaacgccactggcagcagccattggtaactgagaattagtggatttagatatcgagagtcttgaagtggtggcctaacag
aactcaggttgggcctttctgtgctgttttgcggtgaccgtcgtcggtaaccattgactcttaatcacctaaatctatagctctcagaacttcaccaccggattgtc
ColA ori

aggctacactgaaaggacagtatttggtatctgcgctccactaaagccagttaccaggttaagcagttccccaactgacttaaccttcgatcaaaccgcctccccag
tccgatgtgactttcctgtcataaaccatagacgcgaggtgatttcggtcaatggtccaattcgtcaaggggttgactgaattggaagctagtttggcggaggggtc
ColA ori

gcggtttttttcgtttacagagcaggagattacgacgatcgtaaaaggatctcaagaagatcctttacggattcccgacaccatcactctagatttcagtgcaattta
cgccaaaaaagcaaatgtctcgtcctctaatgctgctagcattttcctagagttcttctaggaaatgcctaagggctgtggtagtgagatctaaagtcacgttaaat
ColA ori

tctcttcaaatgtagcacctgaagtcagccccatacgatataagttgtaattctcatgttagtcatgccccgcgcccaccggaaggagctgactgggttgCTCCTAg
agagaagtttacatcgtggacttcagtcggggtatgctatattcaacattaagagtacaatcagtacggggcgcgggtggccttcctcgactgacccaacGAGGATc

GGTCTGATTCGTTACCAATTATGACAACTTGACGGCTACATCATTCACTTTTTCTTCACAACCGGCACGGAACTCGCTCGGGCTGGCCCCGGTGCATTTTTTAAATA
CCAGACTAAGCAATGGTTAATACTGTTGAACTGCCGATGTAGTAAGTGAAAAAGAAGTGTTGGCCGTGCCTTGAGCGAGCCCGACCGGGGCCACGTAAAAAATTTAT
araC

CCCGCGAGAAATAGAGTTGATCGTCAAAACCAACATTGCGACCGACGGTGGCGATAGGCATCCGGGTGGTGCTCAAAAGCAGCTTCGCCTGGCTGATACGTTGGTCC
GGGCGCTCTTTATCTCAACTAGCAGTTTTGGTTGTAACGCTGGCTGCCACCGCTATCCGTAGGCCCACCACGAGTTTTCGTCGAAGCGGACCGACTATGCAACCAGG
araC

TCGCGCCAGCTTAAGACGCTAATCCCTAACTGCTGGCGGAAAAGATGTGACAGACGCGACGGCGACAAGCAAACATGCTGTGCGACGCTGGCGATATCAAAATTGCT
AGCGCGGTCGAATTCTGCGATTAGGGATTGACGACCGCCTTTTCTACACTGTCTGCGCTGCCGCTGTTCGTTTGTACGACACGCTGCGACCGCTATAGTTTTAACGA
araC

GTCTGCCAGGTGATCGCTGATGTACTGACAAGCCTCGCGTACCCGATTATCCATCGGTGGATGGAGCGACTCGTTAATCGCTTCCATGCGCCGCAGTAACAATTGCT
CAGACGGTCCACTAGCGACTACATGACTGTTCGGAGCGCATGGGCTAATAGGTAGCCACCTACCTCGCTGAGCAATTAGCGAAGGTACGCGGCGTCATTGTTAACGA
araC

CAAGCAGATTTATCGCCAGCAGCTCCGAATAGCGCCCTTCCCCTTGCCCGGCGTTAATGATTTGCCCAAACAGGTCGCTGAAATGCGGCTGGTGCGCTTCATCCGGG
GTTCGTCTAAATAGCGGTCGTCGAGGCTTATCGCGGGAAGGGGAACGGGCCGCAATTACTAAACGGGTTTGTCCAGCGACTTTACGCCGACCACGCGAAGTAGGCCC
araC

CGAAAGAACCCCGTATTGGCAAATATTGACGGCCAGTTAAGCCATTCATGCCAGTAGGCGCGCGGACGAAAGTAAACCCACTGGTGATACCATTCGCGAGCCTCCGG
GCTTTCTTGGGGCATAACCGTTTATAACTGCCGGTCAATTCGGTAAGTACGGTCATCCGCGCGCCTGCTTTCATTTGGGTGACCACTATGGTAAGCGCTCGGAGGCC
araC

ATGACGACCGTAGTGATGAATCTCTCCTGGCGGGAACAGCAAAATATCACCCGGTCGGCAAACAAATTCTCGTCCCTGATTTTTCACCACCCCCTGACCGCGAATGG
TACTGCTGGCATCACTACTTAGAGAGGACCGCCCTTGTCGTTTTATAGTGGGCCAGCCGTTTGTTTAAGAGCAGGGACTAAAAAGTGGTGGGGGACTGGCGCTTACC
araC

TGAGATTGAGAATATAACCTTTCATTCCCAGCGGTCGGTCGATAAAAAAATCGAGATAACCGTTGGCCTCAATCGGCGTTAAACCCGCCACCAGATGGGCATTAAAC
ACTCTAACTCTTATATTGGAAAGTAAGGGTCGCCAGCCAGCTATTTTTTTAGCTCTATTGGCAACCGGAGTTAGCCGCAATTTGGGCGGTGGTCTACCCGTAATTTG
araC
```

pJ2042.2_Brocc (6140 bp) (from 6100-6140 bp)

GAGTATCCCGGCAGCAGGGGATCATTTTGCGCTTCAGCCAT
CTCATAGGGCCGTCGTCCCCTAGTAAAACGCGAAGTCGGTA

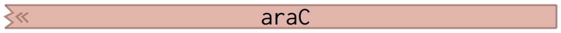

(from 1-1177 bp)

pJ2042.2_Bs (5864 bp)

ACTTTTCATACTCCCGCCATTCAGAGAAGAAACCAATTGTCCATATTGCATCAGACATTGCCGTCACTGCGTCTTTTACTGGCTCTTCTCGCTAACCAAACCGGTAA
TGAAAAGTATGAGGGCGGTAAGTCTCTTCTTTGGTTAACAGGTATAACGTAGTCTGTAACGGCAGTGACGCAGAAAATGACCGAGAAGAGCGATTGGTTTGGCCATT

CCCCGCTTATTAAAAGCATTCTGTAACAAAGCGGGACCAAAGCCATGACAAAAACGCGTAACAAAAGTGTCTATAATCACGGCAGAAAAGTCCACATTGATTATTTG
GGGGCGAATAATTTTCGTAAGACATTGTTTCGCCCTGGTTTCGGTACTGTTTTTGCGCATTGTTTTCACAGATATTAGTGCCGTCTTTTCAGGTGTAACTAATAAAC

EcoRI

CACGGCGTCACACTTTGCTATGCCATAGCATTTTTATCCATAAGATTAGCGGtTCCTACCTGACGCTTTTTATCGCAACTCTCTACTGTTTCTCCATACCGAATTCA
GTGCCGCAGTGTGAAACGATACGGTATCGTAAAAATAGGTATTCTAATCGCCaAGGATGGACTGCGAAAAATAGCGTTGAGAGATGACAAAGAGGTATGGCTTAAGT

P(BAD) promoter

SacI

TAGGATAGATTCTGGAAACTTTACCGTCCGAGCTCCAGCCTGCGGTCCGGGTTCACTGCCGTATAGGCAGAAGCTAGACTCTAGTGGTTTcAGAGCTATGCTGGAAA
ATCCTATCTAAGACCTTTGAAATGGCAGGCTCGAGGTCGGACGCCAGGCCCAAGTGACGGCATATCCGTCTTCGATCTGAGATCACCAAAgTCTCGATACGACCTTT

Linker_14 | Prefix | Csy4 site | sgRNA-1t4

CAGCATAGCAAGTTgAAATAAGGCTAGTCCGTTATCAACTTGAAAAAGTGGCACCGAGTCGGTGCGTTCACTGCCGTATAGGCAGTCGCTGGGACGCCCGGGGACTA
GTCGTATCGTTCAAcTTTATTCCGATCAGGCAATAGTTGAACTTTTTCACCGTGGCTCAGCCACGCAAGTGACGGCATATCCGTCAGCGACCCTGCGGGCCCCTGAT

sgRNA-1t4 | Csy4 site | Suffix

CACTTACGAAACTATTGATTGCTCAGCCTGCGGTCCGGGTTCACTGCCGTATAGGCAGATCAGTGTGTACTAAGTACTGTTTcAGAGCTATGCTGGAAACAGCATAG
GTGAATGCTTTGATAACTAACGAGTCGGACGCCAGGCCCAAGTGACGGCATATCCGTCTAGTCACACATGATTCATGACAAAgTCTCGATACGACCTTTGTCGTATC

Linker_1 | Prefix | Csy4 site | sgRNA-2

XhoI

CAAGTTgAAATAAGGCTAGTCCGTTATCAACTTGAAAAAGTGGCACCGAGTCGGTGCGTTCACTGCCGTATAGGCAGTCGCTGGGACGCCCGCTCGAGCAATAAACA
GTTCAAcTTTATTCCGATCAGGCAATAGTTGAACTTTTTCACCGTGGCTCAGCCACGCAAGTGACGGCATATCCGTCAGCGACCCTGCGGGCGAGCTCGTTATTTGT

sgRNA-2 | Csy4 site | Suffix

NcoI

GTTGATAGGGCTTCTCCGTTACCATGGTTCAGCCAAAAAACTTAAGACCGCCGGTCTTGTCCACTACCTTGCAGTAATGCGGTGGACAGGATCGGCGGTTTTCTTTT
CAACTATCCCGAAGAGGCAATGGTACCAAGTCGGTTTTTTGAATTCTGGCGGCCAGAACAGGTGATGGAACGTCATTACGCCACCTGTCCTAGCCGCCAAAAGAAAA

Linker_0 | ECK120029600 Terminator

CTCTTCTCAATTCTTCTGACCTGTAACGAATAATAGATAGTAAAGTAGTCTCCGATTGAGTTTTCTCTGCCGAGTCCCACCCAGTTCTGTGATTTCAGTAAGTTGGT
GAGAAGAGTTAAGAAGACTGGACATTGCTTATTATCTATCATTTCATCAGAGGCTAACTCAAAAGAGACGGCTCAGGGTGGGTCAAGACACTAAAGTCATTCAACCA

EC...r | Spacer 1

HindIII

AATTGATACACTGTTGCGAGAACTGCTGCCTGGTAGTAGATAGGTTGTTATTGAGTAAGAAGGTAAAGTGAACGAAATCCCTGAAACTGAGACTGTAGAAAATAAGC
TTAACTATGTGACAACGCTCTTGACGACGGACCATCATCTATCCAACAATAACTCATTCTTCCATTTCACTTGCTTTAGGGACTTTGACTCTGACATCTTTTATTCG

Spacer 1

TTCAGCCTGCGGTCCGGttgacggctagctcagtcctaggtacagtgctagcTCGCTGGGACGCCCGAGATAGCCGTTACACAGGTGACACTTATTTCAGCCTGCGG
AAGTCGGACGCCAGGCCAactgccgatcgagtcaggatccatgtcacgatcgAGCGACCCTGCGGGCTCTATCGGCAATGTGTCCACTGTGAATAAAGTCGGACGCC

Prefix | P(BBa_J23100) | Suffix | Linker_24 | Pr...x

pJ2042.2_Bs (5864 bp) (from 1178-2354 bp)

BamHI

TCCGGccaCACTAGAGTCTAGCTTGAGATCGCTGGGACGCCCGGGATCCAAGAGATTTCTACACGATTGAGCACTGTCTCAGCCTGCGGTCCGGGTTCACTGCCGTA
AGGCCggtGTGATCTCAGATCGAACTCTAGCGACCCTGCGGGCCCTAGGTTCTCTAAAGATGTGCTAACTCGTGACAGAGTCGGACGCCAGGCCCAAGTGACGGCAT

bs-1 Suffix Linker_10 Prefix Csy...te

TAGGCAGTAATTTTGTTTAACTTTAAGAAGGAGATATACATatggttagtaaaggagaagaaaataacatggcaCTGATTAAGGAGAACATGCACATGAAGCTGTAC
ATCCGTCATTAAAACAAATTGAAATTCTTCCTCTATATGTAtaccaatcatttcctcttcttttattgtaccgtGACTAATTCCTCTTGTACGTGTACTTCGACATG

RBS mKate2

ATGGAGGGCACCGTGAACAACCACCACTTCAAGTGCACATCCGAGGGCGAAGGCAAGCCCTACGAGGGCACCCAGACCATGAGAATCAAGgccGTCGAGGGCGGCCC
TACCTCCCGTGGCACTTGTTGGTGGTGAAGTTCACGTGTAGGCTCCCGCTTCCGTTCGGGATGCTCCCGTGGGTCTGGTACTCTTAGTTCcggCAGCTCCCGCCGGG

mKate2

TCTCCCCTTCGCCTTCGACATCCTGGCTACCAGCTTCATGTACGGCAGCAAAACCTTCATCAACCACACCCAGGGCATCCCCGACTTCTTTAAGCAGTCCTTCCCTG
AGAGGGGAAGCGGAAGCTGTAGGACCGATGGTCGAAGTACATGCCGTCGTTTTGGAAGTAGTTGGTGTGGGTCCCGTAGGGGCTGAAGAAATTCGTCAGGAAGGGAC

mKate2

AGGGCTTCACATGGGAGAGAGTCACCACATACGAAGACGGGGGCGTGCTGACCGCTACCCAGGACACCAGCCTCCAGGACGGCTGCCTCATCTACAACGTCAAGATC
TCCCGAAGTGTACCCTCTCTCAGTGGTGTATGCTTCTGCCCCCGCACGACTGGCGATGGGTCCTGTGGTCGGAGGTCCTGCCGACGGAGTAGATGTTGCAGTTCTAG

mKate2

AGAGGGGTGAACTTCCCATCCAACGGCCCTGTGATGCAGAAGAAAACACTCGGCTGGGAGGCCTCCACCGAGaccCTGTACCCCGCTGACGGCGGCCTGGAAGGCAG
TCTCCCCACTTGAAGGGTAGGTTGCCGGGACACTACGTCTTCTTTTGTGAGCCGACCCTCCGGAGGTGGCTCtggGACATGGGGCGACTGCCGCCGGACCTTCCGTC

mKate2

AgcCGACATGGCCCTGAAGCTCGTGGGCGGGGGCCACCTGATCTGCAACTTGAAGACCACATACAGATCCAAGAAACCCGCTAAGAACCTCAAGATGCCCGGCGTCT
TcgGCTGTACCGGGACTTCGAGCACCCGCCCCCGGTGGACTAGACGTTGAACTTCTGGTGTATGTCTAGGTTCTTTGGGCGATTCTTGGAGTTCTACGGGCCGCAGA

mKate2

ACTATGTGGACAGAAGACTGGAAAGAATCAAGGAGGCCGACAAAGAGACCTACGTCGAGCAGCACGAGGTGGCTGTGGCCAGATACTGCGACCTCCCTAGCAAACTG
TGATACACCTGTCTTCTGACCTTTCTTAGTTCCTCCGGCTGTTTCTCTGGATGCAGCTCGTCGTGCTCCACCGACACCGGTCTATGACGCTGGAGGGATCGTTTGAC

mKate2

SbfI

GGGCACAgAgtcATGTCCCGCCGTAATACTGACGCCATCACAATCCACAGCATCCTGGATTGGATTGAAGACTAATAATCGCTGGGACGCCCGCCTGCAGGCTCGGT
CCCGTGTcTcagTACAGGGCGGCATTATGACTGCGGTAGTGTTAGGTGTCGTAGGACCTAACCTAACTTCTGATTATTAGCGACCCTGCGGGCGGACGTCCGAGCCA

mKate2 MarAn20 Suffix

KpnI

ACCAAATTCCAGAAAAGAGGCCTCCCGAAAGGGGGGCCTTTTTTCGTTTTGGTCCTAATAGATAAAGGATAGGTCTGGTAGTGTTGTTCGTTCTCGCAGGTAAATCA
TGGTTTAAGGTCTTTTCTCCGGAGGGCTTTCCCCCCGGAAAAAAGCAAAACCAGGATTATCTATTTCCTATCCAGACCATCACAACAAGCAAGAGCGTCCATTTAGT

L3S2P21 Terminator Spacer 2.5

ATAATACTCAGCAGTTCCGTAGACTTTTCAGTGGGACAGGGTAGCGATAACAGATAGATTGTAATAAGACACAGTAGGTGCTCGTAGTTGCGTGAAGAGAACCGCTC
TATTATGAGTCGTCAAGGCATCTGAAAAGTCACCCTGTCCCATCGCTATTGTCTATCTAACATTATTCTGTGTCATCCACGAGCATCAACGCACTTCTCTTGGCGAG

Spacer 2.5

pJ2042.2_Bs (5864 bp) (from 2355-3531 bp)

SalI

AGGAAATCCAGTCAGAAGTATTGGTAATCGTTGAAAACTCAGTCGACCAGCCTGCGGTCCGGTTTACGGCTAGCTCAGTCCTAGGTATTATGCTAGCTCGCTGGGAC
TCCTTTAGGTCAGTCTTCATAACCATTAGCAACTTTTGAGTCAGCTGGTCGGACGCCAGGCCAAATGCCGATCGAGTCAGGATCCATAATACGATCGAGCGACCCTG

Spacer 2.5 | Prefix | P(BBa_J23150) | Su...x

GCCCGAGTGACGACTGCGAAGTAACCTCTATTTATCAGCCTGCGGTCCGGccaAGTACTTAGTACACACTGATTCGCTGGGACGCCCGGTAGTGCTTATCAGACCCA
CGGGCTCACTGCTGACGCTTCATTGGAGATAAATAGTCGGACGCCAGGCCggtTCATGAATCATGTGTGACTAAGCGACCCTGCGGGCCATCACGAATAGTCTGGGT

Linker_25 | Prefix | bs-2 | Suffix | Linker_17

ATACTGTTGAACAGCCTGCGGTCCGGGTTCACTGCCGTATAGGCAGTCTCAAGCTAGACTCTAGTGGTTTcAGAGCTATGCTGGAAACAGCATAGCAAGTTgAAATA
TATGACAACTTGTCGGACGCCAGGCCCAAGTGACGGCATATCCGTCAGAGTTCGATCTGAGATCACCAAAgTCTCGATACGACCTTTGTCGTATCGTTCAAcTTTAT

Li...7 | Prefix | Csy4 site | sgRNA-1

AatII

AGGCTAGTCCGTTATCAACTTGAAAAAGTGGCACCGAGTCGGTGCGTTCACTGCCGTATAGGCAGTCGCTGGGACGCCCGGACGTCCTATTACACTCGTCGTTGGAA
TCCGATCAGGCAATAGTTGAACTTTTTCACCGTGGCTCAGCCACGCAAGTGACGGCATATCCGTCAGCGACCCTGCGGGCCTGCAGGATAATGTGAGCAGCAACCTT

sgRNA-1 | Csy4 site | Suffix | Linker_11

NotI

ACTGAAGATGCGGCCGCggaaacacagAAAAAAGCCCGCACCTGACAGTGCGGGCTTTTTTTTTcgaccaaaggTAGCGAACGACGAGTCACTGTTGAGGATAAATA
TGACTTCTACGCCGGCGcctttgtgtcTTTTTTCGGGCGTGGACTGTCACGCCCGAAAAAAAAAgctggtttccATCGCTTGCTGCTCAGTGACAACTCCTATTTAT

ECK120033737 Terminator | Linker_8

KasI BbvCI AscI

CTTTCTCTACTAGGCGCCTGTTACACAGGTCCTCAGCGGCGCGCCTTTGTCGGTGAACGCTCTCCTGAGTAGGACAAATCCGCCGGGAGCGGATTTGAACGTTGTGA
GAAAGAGATGATCCGCGGACAATGTGTCCAGGAGTCGCCGCGCGGAAACAGCCACTTGCGAGAGGACTCATCCTGTTTAGGCGGCCCTCGCCTAAACTTGCAACACT

Lin..._8 | Spa...15

AGCAACGGCCCGGAGGGTGGCGGGCAGGACGCCCGCCATAAACTGCCAGGCATCAAACTAAGCAGAAGGCCATCCTGACGGATGGCCTTTTTGCGTTTCAGATCTAC
TCGTTGCCGGGCCTCCCACCGCCCGTCCTGCGGGCGGTATTTGACGGTCCGTAGTTTGATTCGTCTTCCGGTAGGACTGCCTACCGGAAAAACGCAAAGTCTAGATG

CGGTaaaccagcaatagacataagcggctatttaacgaccctgccctgaaccgacgacaagctgacgaccgggtctccgcaagtggcacttttcggggaaatgtgcg
GCCAtttggtcgttatctgtattcgccgataaattgctgggacgggacttggctgctgttcgactgctggcccagaggcgttcaccgtgaaaagcccctttacacgc

cggaacccctatttgtttatttttctaaatacattcaaatatgtatccgctcatgaattaattcttagaaaaactcatcgagcatcaaatgaaactgcaatttattc
gccttggggataaacaaataaaaagatttatgtaagtttatacataggcgagtacttaattaagaatctttttgagtagctcgtagtttactttgacgttaaataag

KanR

atatcaggattatcaataccatatttttgaaaaagccgtttctgtaatgaaggagaaaactcaccgaggcagttccataggatggcaagatcctggtatcggtctgc
tatagtcctaatagttatggtataaaaactttttcggcaaagacattacttcctcttttgagtggctccgtcaaggtatcctaccgttctaggaccatagccagacg

KanR

gattccgactcgtccaacatcaatacaacctattaatttcccctcgtcaaaaataaggttatcaagtgagaaatcaccatgagtgacgactgaatccggtgagaatg
ctaaggctgagcaggttgtagttatgttggataattaaaggggagcagtttttattccaatagttcactctttagtggtactcactgctgacttaggccactcttac

KanR

pJ2042.2_Bs (5864 bp) (from 3532-4815 bp)

gcaaaagtttatgcatttctttccagacttgttcaacaggccagccattacgctcgtcatcaaaatcactcgcatcaaccaaaccgttattcattcgtgattgcgcc
cgttttcaaatacgtaaagaaaggtctgaacaagttgtccggtcggtaatgcgagcagtagttttagtgagcgtagttggtttggcaataagtaagcactaacgcgg
KanR

tgagcgagacgaaatacgcggtcgctgttaaaaggacaattacaaacaggaatcgaatgcaaccggcgcaggaacactgccagcgcatcaacaatattttcacctga
actcgctctgctttatgcgccagcgacaattttcctgttaatgtttgtccttagcttacgttggccgcgtccttgtgacggtcgcgtagttgttataaaagtggact
KanR

atcaggatattcttctaatacctggaatgctgttttcccggggatcgcagtggtgagtaaccatgcatcatcaggagtacggataaaatgcttgatggtcggaagag
tagtcctataagaagattatggaccttacgacaaaagggcccctagcgtcaccactcattggtacgtagtagtcctcatgcctattttacgaactaccagccttctc
KanR

gcataaattccgtcagccagtttagtctgaccatctcatctgtaacatcattggcaacgctacctttgccatgtttcagaaacaactctggcgcatcgggcttccca
cgtatttaaggcagtcggtcaaatcagactggtagagtagacattgtagtaaccgttgcgatggaaacggtacaaagtctttgttgagaccgcgtagcccgaagggt
KanR

tacaatcgatagattgtcgcacctgattgcccgacattatcgcgagcccatttatacccatataaatcagcatccatgttggaatttaatcgcggcctagagcaaga
atgttagctatctaacagcgtggactaacgggctgtaatagcgctcgggtaaatatgggtatatttagtcgtaggtacaaccttaaattagcgccggatctcgttct
KanR

cgtttcccgttgaatatggctcatactcttcctttttcaatattattgaagcatttatcagggttattgtctcatgagcggatacatatttgaatgtatttagaaaa
gcaaagggcaacttataccgagtatgagaaggaaaaagttataataacttcgtaaatagtcccaataacagagtactcgcctatgtataaacttacataaatctttt
KanR

ataaacaaataggcatgctagcgcagaaacgtcctagaagatgccaggaggatacttagcagagagacaataaggccggagcgaagccgtttttccataggctccgc
tatttgtttatccgtacgatcgcgtctttgcaggatcttctacggtcctcctatgaatcgtctctctgttattccggcctcgcttcggcaaaaaggtatccgaggcg
ColA ori

ccccctgacgaacatcacgaaatctgacgctcaaatcagtggtggcgaaacccgacaggactataaagataccaggcgtttccccctgatggctccctcttgcgctc
gggggactgcttgtagtgctttagactgcgagtttagtcaccaccgctttgggctgtcctgatatttctatggtccgcaaagggggactaccgagggagaacgcgag
ColA ori

tcctgttcccgtcctgcggcgtccgtgttgtggtggaggctttacccaaatcaccacgtcccgttccgtgtagacagttcgctccaagctgggctgtgtgcaagaac
aggacaagggcaggacgccgcaggcacaacaccacctccgaaatgggtttagtggtgcagggcaaggcacatctgtcaagcgaggttcgacccgacacacgttcttg
ColA ori

cccccgttcagcccgactgctgcgccttatccggtaactatcatcttgagtccaacccggaaagacacgacaaaacgccactggcagcagccattggtaactgagaa
gggggcaagtcgggctgacgacgcggaataggccattgatagtagaactcaggttgggcctttctgtgctgttttgcggtgaccgtcgtcggtaaccattgactctt
ColA ori

ttagtggatttagatatcgagagtcttgaagtggtggcctaacagaggctacactgaaaggacagtatttggtatctgcgctccactaaagccagttaccaggttaa
aatcacctaaatctatagctctcagaacttcaccaccggattgtctccgatgtgactttcctgtcataaaccatagacgcgaggtgatttcggtcaatggtccaatt
ColA ori

gcagttccccaactgacttaaccttcgatcaaaccgcctccccaggcggttttttcgtttacagagcaggagattacgacgatcgtaaaaggatctcaagaagatcc
cgtcaaggggttgactgaattggaagctagtttggcggaggggtccgccaaaaaagcaaatgtctcgtcctctaatgctgctagcattttcctagagttcttctagg
ColA ori

tttacggattcccgacaccatcactctagatttcagtgcaatttatctcttcaaatgtagcacctgaagtcagccccatacgatataagttgtaattctcatgttag
aaatgcctaagggctgtggtagtgagatctaaagtcacgttaaatagagaagtttacatcgtggacttcagtcggggtatgctatattcaacattaagagtacaatc
ColA ori

tcatgccccgcgcccaccggaaggagctgactgggttgCTCCTAgGGTCTGATTCGTTACCAATTATGACAACTTGACGGCTACATCATTCACTTTTTCTTCACAAC
agtacggggcgcgggtggccttcctcgactgacccaacGAGGATcCCAGACTAAGCAATGGTTAATACTGTTGAACTGCCGATGTAGTAAGTGAAAAAGAAGTGTTG
araC

CGGCACGGAACTCGCTCGGGCTGGCCCCGGTGCATTTTTTAAATACCCGCGAGAAATAGAGTTGATCGTCAAAACCAACATTGCGACCGACGGTGGCGATAGGCATC
GCCGTGCCTTGAGCGAGCCCGACCGGGGCCACGTAAAAAATTTATGGGCGCTCTTTATCTCAACTAGCAGTTTTGGTTGTAACGCTGGCTGCCACCGCTATCCGTAG
araC

CGGGTGGTGCTCAAAAGCAGCTTCGCCTGGCTGATACGTTGGTCCTCGCGCCAGCTTAAGACGCTAATCCCTAACTGCTGGCGGAAAAGATGTGACAGACGCGACGG
GCCCACCACGAGTTTTCGTCGAAGCGGACCGACTATGCAACCAGGAGCGCGGTCGAATTCTGCGATTAGGGATTGACGACCGCCTTTTCTACACTGTCTGCGCTGCC
araC

CGACAAGCAAACATGCTGTGCGACGCTGGCGATATCAAAATTGCTGTCTGCCAGGTGATCGCTGATGTACTGACAAGCCTCGCGTACCCGATTATCCATCGGTGGAT
GCTGTTCGTTTGTACGACACGCTGCGACCGCTATAGTTTTAACGACAGACGGTCCACTAGCGACTACATGACTGTTCGGAGCGCATGGGCTAATAGGTAGCCACCTA
araC

GGAGCGACTCGTTAATCGCTTCCATGCGCCGCAGTAACAATTGCTCAAGCAGATTTATCGCCAGCAGCTCCGAATAGCGCCCTTCCCCTTGCCCGGCGTTAATGATT
CCTCGCTGAGCAATTAGCGAAGGTACGCGGCGTCATTGTTAACGAGTTCGTCTAAATAGCGGTCGTCGAGGCTTATCGCGGGAAGGGGAACGGGCCGCAATTACTAA
araC

TGCCCAAACAGGTCGCTGAAATGCGGCTGGTGCGCTTCATCCGGGCGAAAGAACCCCGTATTGGCAAATATTGACGGCCAGTTAAGCCATTCATGCCAGTAGGCGCG
ACGGGTTTGTCCAGCGACTTTACGCCGACCACGCGAAGTAGGCCCGCTTTCTTGGGGCATAACCGTTTATAACTGCCGGTCAATTCGGTAAGTACGGTCATCCGCGC
araC

CGGACGAAAGTAAACCCACTGGTGATACCATTCGCGAGCCTCCGGATGACGACCGTAGTGATGAATCTCTCCTGGCGGGAACAGCAAAATATCACCCGGTCGGCAAA
GCCTGCTTTCATTTGGGTGACCACTATGGTAAGCGCTCGGAGGCCTACTGCTGGCATCACTACTTAGAGAGGACCGCCCTTGTCGTTTTATAGTGGGCCAGCCGTTT
araC

CAAATTCTCGTCCCTGATTTTTCACCACCCCCTGACCGCGAATGGTGAGATTGAGAATATAACCTTTCATTCCCAGCGGTCGGTCGATAAAAAAATCGAGATAACCG
GTTTAAGAGCAGGGACTAAAAAGTGGTGGGGGACTGGCGCTTACCACTCTAACTCTTATATTGGAAAGTAAGGGTCGCCAGCCAGCTATTTTTTTAGCTCTATTGGC
araC

TTGGCCTCAATCGGCGTTAAACCCGCCACCAGATGGGCATTAAACGAGTATCCCGGCAGCAGGGGATCATTTTGCGCTTCAGCCAT
AACCGGAGTTAGCCGCAATTTGGGCGGTGGTCTACCCGTAATTTGCTCATAGGGCCGTCGTCCCCTAGTAAAACGCGAAGTCGGTA
araC

(from 1-1177 bp)

pJ2042.2_GFPonly (5858 bp)

ACTTTTCATACTCCCGCCATTCAGAGAAGAAACCAATTGTCCATATTGCATCAGACATTGCCGTCACTGCGTCTTTTACTGGCTCTTCTCGCTAACCAAACCGGTAA
TGAAAAGTATGAGGGCGGTAAGTCTCTTCTTTGGTTAACAGGTATAACGTAGTCTGTAACGGCAGTGACGCAGAAAATGACCGAGAAGAGCGATTGGTTTGGCCATT

CCCCGCTTATTAAAAGCATTCTGTAACAAAGCGGGACCAAAGCCATGACAAAAACGCGTAACAAAAGTGTCTATAATCACGGCAGAAAAGTCCACATTGATTATTTG
GGGGCGAATAATTTTCGTAAGACATTGTTTCGCCCTGGTTTCGGTACTGTTTTTGCGCATTGTTTTCACAGATATTAGTGCCGTCTTTTCAGGTGTAACTAATAAAC

EcoRI
CACGGCGTCACACTTTGCTATGCCATAGCATTTTTATCCATAAGATTAGCGGtTCCTACCTGACGCTTTTTATCGCAACTCTCTACTGTTTCTCCATACCGAATTCA
GTGCCGCAGTGTGAAACGATACGGTATCGTAAAAATAGGTATTCTAATCGCCaAGGATGGACTGCGAAAAATAGCGTTGAGAGATGACAAAGAGGTATGGCTTAAGT
P(BAD) promoter

SacI
TAGGATAGATTCTGGAAACTTTACCGTCCGAGCTCCAGCCTGCGGTCCGGGTTCACTGCCGTATAGGCAGAAGCTAGACTCTAGTGGTTTcAGAGCTATGCTGGAAA
ATCCTATCTAAGACCTTTGAAATGGCAGGCTCGAGGTCGGACGCCAGGCCCAAGTGACGGCATATCCGTCTTCGATCTGAGATCACCAAAgTCTCGATACGACCTTT
Linker_14 Prefix Csy4 site sgRNA-1t4

CAGCATAGCAAGTTgAAATAAGGCTAGTCCGTTATCAACTTGAAAAAGTGGCACCGAGTCGGTGCGTTCACTGCCGTATAGGCAGTCGCTGGGACGCCCGGGGACTA
GTCGTATCGTTCAAcTTTATTCCGATCAGGCAATAGTTGAACTTTTTCACCGTGGCTCAGCCACGCAAGTGACGGCATATCCGTCAGCGACCCTGCGGGCCCCTGAT
sgRNA-1t4 Csy4 site Suffix

CACTTACGAAACTATTGATTGCTCAGCCTGCGGTCCGGGTTCACTGCCGTATAGGCAGATCAGTGTGTACTAAGTACTGTTTcAGAGCTATGCTGGAAACAGCATAG
GTGAATGCTTTGATAACTAACGAGTCGGACGCCAGGCCCAAGTGACGGCATATCCGTCTAGTCACACATGATTCATGACAAAgTCTCGATACGACCTTTGTCGTATC
Linker_1 Prefix Csy4 site sgRNA-2

XhoI
CAAGTTgAAATAAGGCTAGTCCGTTATCAACTTGAAAAAGTGGCACCGAGTCGGTGCGTTCACTGCCGTATAGGCAGTCGCTGGGACGCCCGCTCGAGCAATAAACA
GTTCAAcTTTATTCCGATCAGGCAATAGTTGAACTTTTTCACCGTGGCTCAGCCACGCAAGTGACGGCATATCCGTCAGCGACCCTGCGGGCGAGCTCGTTATTTGT
sgRNA-2 Csy4 site Suffix

NcoI
GTTGATAGGGCTTCTCCGTTACCATGGTTCAGCCAAAAAACTTAAGACCGCCGGTCTTGTCCACTACCTTGCAGTAATGCGGTGGACAGGATCGGCGGTTTTCTTTT
CAACTATCCCGAAGAGGCAATGGTACCAAGTCGGTTTTTTGAATTCTGGCGGCCAGAACAGGTGATGGAACGTCATTACGCCACCTGTCCTAGCCGCCAAAAGAAAA
Linker_0 ECK120029600 Terminator

CTCTTCTCAATTCTTCTGACCTGTAACGAATAATAGATAGTAAAGTAGTCTCCGATTGAGTTTTCTCTGCCGAGTCCCACCCAGTTCTGTGATTTCAGTAAGTTGGT
GAGAAGAGTTAAGAAGACTGGACATTGCTTATTATCTATCATTTCATCAGAGGCTAACTCAAAAGAGACGGCTCAGGGTGGGTCAAGACACTAAAGTCATTCAACCA
EC...r Spacer 1

HindIII
AATTGATACACTGTTGCGAGAACTGCTGCCTGGTAGTAGATAGGTTGTTATTGAGTAAGAAGGTAAAGTGAACGAAATCCCTGAAACTGAGACTGTAGAAAATAAGC
TTAACTATGTGACAACGCTCTTGACGACGGACCATCATCTATCCAACAATAACTCATTCTTCCATTTCACTTGCTTTAGGGACTTTGACTCTGACATCTTTTATTCG
Spacer 1

TTCAGCCTGCGGTCCGGttgacggctagctcagtcctaggtacagtgctagcTCGCTGGGACGCCCGAGATAGCCGTTACACAGGTGACACTTATTTCAGCCTGCGG
AAGTCGGACGCCAGGCCaactgccgatcgagtcaggatccatgtcacgatcgAGCGACCCTGCGGGCTCTATCGGCAATGTGTCCACTGAATAAAGTCGGACGCC
Prefix P(BBa_J23100) Suffix Linker_24 Pr...x

pJ2042.2_GFPonly (5858 bp) (from 1178-2354 bp)

BamHI

TCCGGccaCACTAGAGTCTAGCTTGAGATCGCTGGGACGCCCGGGATCCAAGAGATTTCTACACGATTGAGCACTGTCTCAGCCTGCGGTCCGGGTTCACTGCCGTA
AGGCCggtGTGATCTCAGATCGAACTCTAGCGACCCTGCGGGCCCTAGGTTCTCTAAAGATGTGCTAACTCGTGACAGAGTCGGACGCCAGGCCCAAGTGACGGCAT

bs-1 | Suffix | Linker_10 | Prefix | Csy...te

TAGGCAGTAATTTTGTTTAACTTTAAGAAGGAGATATACATATGCGTAAAGGCGAAGAACTGTTTACCGGTGTGGTTCCGATTCTGGTGGAACTGGACGGCGATGTT
ATCCGTCATTAAAACAAATTGAAATTCTTCCTCTATATGTATACGCATTTCCGCTTCTTGACAAATGGCCACACCAAGGCTAAGACCACCTTGACCTGCCGCTACAA

RBS | sfGFP

AATGGTCATAAATTCAGTGTTCGCGGCGAAGGTGAAGGCGATGCGACGAACGGCAAACTGACCCTGAAATTTATCTGCACCACGGGTAAACTGCCGGTCCCGTGGCC
TTACCAGTATTTAAGTCACAAGCGCCGCTTCCACTTCCGCTACGCTGCTTGCCGTTTGACTGGGACTTTAAATAGACGTGGTGCCCATTTGACGGCCAGGGCACCGG

sfGFP

GACGCTGGTGACCACGCTGACCTATGGCGTTCAATGTTTTGCGCGTTACCCGGATCACATGAAACAGCACGACTTTTTCAAATCGGCCATGCCGGAAGGCTATGTGC
CTGCGACCACTGGTGCGACTGGATACCGCAAGTTACAAAACGCGCAATGGGCCTAGTGTACTTTGTCGTGCTGAAAAAGTTTAGCCGGTACGGCCTTCCGATACACG

sfGFP

AGGAACGTACGATTAGCTTTAAAGACGATGGTACGTATAAAACCCGCGCGGAAGTGAAATTCGAAGGCGATACCCTGGTTAACCGTATCGAACTGAAAGGTATCGAT
TCCTTGCATGCTAATCGAAATTTCTGCTACCATGCATATTTTGGGCGCGCCTTCACTTTAAGCTTCCGCTATGGGACCAATTGGCATAGCTTGACTTTCCATAGCTA

sfGFP

TTCAAAGAAGACGGCAATATTCTGGGTCATAAACTGGAATATAACTTCAATTCCCACAACGTGTACATCACCGCGGATAAACAGAAAAACGGCATTAAAGCCAATTT
AAGTTTCTTCTGCCGTTATAAGACCCAGTATTTGACCTTATATTGAAGTTAAGGGTGTTGCACATGTAGTGGCGCCTATTTGTCTTTTTGCCGTAATTTCGGTTAAA

sfGFP

CAAAATCCGCCATAATGTGGAAGATGGTAGCGTTCAGCTGGCCGACCACTATCAGCAAAACACGCCGATTGGTGATGGCCCGGTCCTGCTGCCGGACAATCACTACC
GTTTTAGGCGGTATTACACCTTCTACCATCGCAAGTCGACCGGCTGGTGATAGTCGTTTTGTGCGGCTAACCACTACCGGGCCAGGACGACGGCCTGTTAGTGATGG

sfGFP

TGAGTACCCAGTCCGTGCTGTCAAAAGATCCGAACGAAAAACGTGACCACATGGTCCTGCTGGAATTTGTGACGGCTGCGGGTATCACCCACGGCATGGACGAACTG
ACTCATGGGTCAGGCACGACAGTTTTCTAGGCTTGCTTTTTGCACTGGTGTACCAGGACGACCTTAAACACTGCCGACGCCCATAGTGGGTGCCGTACCTGCTTGAC

sfGFP

SbfI | KpnI

TATAAAATGTCCCGCCGTAATACTGACGCCATCACAATCCACAGCATCCTGGATTGGATTGAAGACTAATAATCGCTGGGACGCCCGCCTGCAGGCTCGGTACCAAA
ATATTTTACAGGGCGGCATTATGACTGCGGTAGTGTTAGGTGTCGTAGGACCTAACCTAACTTCTGATTATTAGCGACCCTGCGGGCGGACGTCCGAGCCATGGTTT

MarAn20 | Suffix | L3S...or

TTCCAGAAAAGAGGCCTCCCGAAAGGGGGGCCTTTTTTCGTTTTGGTCCTAATAGATAAAGGATAGGTCTGGTAGTGTTGTTCGTTCTCGCAGGTAAATCAATAATA
AAGGTCTTTTCTCCGGAGGGCTTTCCCCCCGGAAAAAAGCAAAACCAGGATTATCTATTTCCTATCCAGACCATCACAACAAGCAAGAGCGTCCATTTAGTTATTAT

L3S2P21 Terminator | Spacer 2.5

CTCAGCAGTTCCGTAGACTTTTCAGTGGGACAGGGTAGCGATAACAGATAGATTGTAATAAGACACAGTAGGTGCTCGTAGTTGCGTGAAGAGAACCGCTCAGGAAA
GAGTCGTCAAGGCATCTGAAAAGTCACCCTGTCCCATCGCTATTGTCTATCTAACATTATTCTGTGTCATCCACGAGCATCAACGCACTTCTCTTGGCGAGTCCTTT

Spacer 2.5

pJ2042.2_GFPonly (5858 bp) (from 2355-3531 bp)

SalI

TCCAGTCAGAAGTATTGGTAATCGTTGAAAACTCAGTCGACCAGCCTGCGGTCCGGTTTACGGCTAGCTCAGTCCTAGGTATTATGCTAGCTCGCTGGGACGCCCGA
AGGTCAGTCTTCATAACCATTAGCAACTTTTGAGTCAGCTGGTCGGACGCCAGGCCAAATGCCGATCGAGTCAGGATCCATAATACGATCGAGCGACCCTGCGGGCT

Spacer 2.5 | Prefix | P(BBa_J23150) | Suffix

GTGACGACTGCGAAGTAACCTCTATTTATCAGCCTGCGGTCCGGccaAGTACTTAGTACACACTGATTCGCTGGGACGCCCGGTAGTGCTTATCAGACCCAATACTG
CACTGCTGACGCTTCATTGGAGATAAATAGTCGGACGCCAGGCCggtTCATGAATCATGTGTGACTAAGCGACCCTGCGGGCCATCACGAATAGTCTGGGTTATGAC

Linker_25 | Prefix | bs-2 | Suffix | Linker_17

TTGAACAGCCTGCGGTCCGGGTTCACTGCCGTATAGGCAGTCTCAAGCTAGACTCTAGTGGTTTcAGAGCTATGCTGGAAACAGCATAGCAAGTTgAAATAAGGCTA
AACTTGTCGGACGCCAGGCCCAAGTGACGGCATATCCGTCAGAGTTCGATCTGAGATCACCAAAgTCTCGATACGACCTTTGTCGTATCGTTCAAcTTTATTCCGAT

Prefix | Csy4 site | sgRNA-1

AatII

GTCCGTTATCAACTTGAAAAAGTGGCACCGAGTCGGTGCGTTCACTGCCGTATAGGCAGTCGCTGGGACGCCCGGACGTCCTATTACACTCGTCGTTGGAAACTGAA
CAGGCAATAGTTGAACTTTTTCACCGTGGCTCAGCCACGCAAGTGACGGCATATCCGTCAGCGACCCTGCGGGCCTGCAGGATAATGTGAGCAGCAACCTTTGACTT

sgRNA-1 | Csy4 site | Suffix | Linker_11

NotI

GATGCGGCCGCggaaacacagAAAAAAGCCCGCACCTGACAGTGCGGGCTTTTTTTTTcgaccaaaggTAGCGAACGACGAGTCACTGTTGAGGATAAATACTTTCT
CTACGCCGGCGcctttgtgtcTTTTTTCGGGCGTGGACTGTCACGCCCGAAAAAAAAAgctggtttccATCGCTTGCTGCTCAGTGACAACTCCTATTTATGAAAGA

ECK120033737 Terminator | Linker_8

KasI | BbvCI | AscI

CTACTAGGCGCCTGTTACACAGGTCCTCAGCGGCGCGCCTTTGTCGGTGAACGCTCTCCTGAGTAGGACAAATCCGCCGGGAGCGGATTTGAACGTTGTGAAGCAAC
GATGATCCGCGGACAATGTGTCCAGGAGTCGCCGCGCGGAAACAGCCACTTGCGAGAGGACTCATCCTGTTTAGGCGGCCCTCGCCTAAACTTGCAACACTTCGTTG

Spa...15

GGCCCGGAGGGTGGCGGGCAGGACGCCCGCCATAAACTGCCAGGCATCAAACTAAGCAGAAGGCCATCCTGACGGATGGCCTTTTTGCGTTTCAGATCTACCGGTaa
CCGGGCCTCCCACCGCCCGTCCTGCGGGCGGTATTTGACGGTCCGTAGTTTGATTCGTCTTCCGGTAGGACTGCCTACCGGAAAAACGCAAAGTCTAGATGGCCAtt

accagcaatagacataagcggctatttaacgaccctgccctgaaccgacgacaagctgacgaccgggtctccgcaagtggcacttttcggggaaatgtgcgcggaac
tggtcgttatctgtattcgccgataaattgctgggacgggacttggctgctgttcgactgctggcccagaggcgttcaccgtgaaaagcccctttacacgcgccttg

ccctatttgtttatttttctaaatacattcaaatatgtatccgctcatgaattaattcttagaaaaactcatcgagcatcaaatgaaactgcaatttattcatatca
gggataaacaaataaaaagatttatgtaagtttatacataggcgagtacttaattaagaatctttttgagtagctcgtagtttactttgacgttaaataagtatagt

KanR

ggattatcaataccatatttttgaaaaagccgtttctgtaatgaaggagaaaactcaccgaggcagttccataggatggcaagatcctggtatcggtctgcgattcc
cctaatagttatggtataaaaactttttcggcaaagacattacttcctcttttgagtggctccgtcaaggtatcctaccgttctaggaccatagccagacgctaagg

KanR

gactcgtccaacatcaatacaacctattaatttcccctcgtcaaaaataaggttatcaagtgagaaatcaccatgagtgacgactgaatccggtgagaatggcaaaa
ctgagcaggttgtagttatgttggataattaaaggggagcagtttttattccaatagttcactctttagtggtactcactgctgacttaggccactcttaccgtttt

KanR

gtttatgcatttctttccagacttgttcaacaggccagccattacgctcgtcatcaaaatcactcgcatcaaccaaaccgttattcattcgtgattgcgcctgagcg
caaatacgtaaagaaaggtctgaacaagttgtccggtcggtaatgcgagcagtagttttagtgagcgtagttggtttggcaataagtaagcactaacgcggactcgc
KanR

agacgaaatacgcggtcgctgttaaaaggacaattacaaacaggaatcgaatgcaaccggcgcaggaacactgccagcgcatcaacaatattttcacctgaatcagg
tctgctttatgcgccagcgacaattttcctgttaatgtttgtccttagcttacgttggccgcgtccttgtgacggtcgcgtagttgttataaaagtggacttagtcc
KanR

atattcttctaatacctggaatgctgttttcccggggatcgcagtggtgagtaaccatgcatcatcaggagtacggataaaatgcttgatggtcggaagaggcataa
tataagaagattatggaccttacgacaaaagggcccctagcgtcaccactcattggtacgtagtagtcctcatgcctattttacgaactaccagccttctccgtatt
KanR

attccgtcagccagtttagtctgaccatctcatctgtaacatcattggcaacgctacctttgccatgtttcagaaacaactctggcgcatcgggcttcccatacaat
taaggcagtcggtcaaatcagactggtagagtagacattgtagtaaccgttgcgatggaaacggtacaaagtctttgttgagaccgcgtagcccgaagggtatgtta
KanR

cgatagattgtcgcacctgattgcccgacattatcgcgagcccatttatacccatataaatcagcatccatgttggaatttaatcgcggcctagagcaagacgtttc
gctatctaacagcgtggactaacgggctgtaatagcgctcgggtaaatatgggtatatttagtcgtaggtacaaccttaaattagcgccggatctcgttctgcaaag
KanR

ccgttgaatatggctcatactcttcctttttcaatattattgaagcatttatcagggttattgtctcatgagcggatacatatttgaatgtatttagaaaaataaac
ggcaacttataccgagtatgagaaggaaaaagttataataacttcgtaaatagtcccaataacagagtactcgcctatgtataaacttacataaatctttttatttg
KanR

aaataggcatgctagcgcagaaacgtcctagaagatgccaggaggatacttagcagagagacaataaggccggagcgaagccgtttttccataggctccgcccccct
tttatccgtacgatcgcgtctttgcaggatcttctacggtcctcctatgaatcgtctctctgttattccggcctcgcttcggcaaaaaggtatccgaggcgggggga
ColA ori

gacgaacatcacgaaatctgacgctcaaatcagtggtggcgaaacccgacaggactataaagataccaggcgtttccccctgatggctccctcttgcgctctcctgt
ctgcttgtagtgctttagactgcgagtttagtcaccaccgctttgggctgtcctgatatttctatggtccgcaaagggggactaccgagggagaacgcgagaggaca
ColA ori

tcccgtcctgcggcgtccgtgttgtggtggaggctttacccaaatcaccacgtcccgttccgtgtagacagttcgctccaagctgggctgtgtgcaagaaccccccg
agggcaggacgccgcaggcacaacaccacctccgaaatgggtttagtggtgcagggcaaggcacatctgtcaagcgaggttcgacccgacacacgttcttggggggc
ColA ori

ttcagcccgactgctgcgccttatccggtaactatcatcttgagtccaacccggaaagacacgacaaaacgccactggcagcagccattggtaactgagaattagtg
aagtcgggctgacgacgcggaataggccattgatagtagaactcaggttgggcctttctgtgctgttttgcggtgaccgtcgtcggtaaccattgactcttaatcac
ColA ori

gatttagatatcgagagtcttgaagtggtggcctaacagaggctacactgaaaggacagtatttggtatctgcgctccactaaagccagttaccaggttaagcagtt
ctaaatctatagctctcagaacttcaccaccggattgtctccgatgtgactttcctgtcataaaccatagacgcgaggtgatttcggtcaatggtccaattcgtcaa
ColA ori

ccccaactgacttaaccttcgatcaaaccgcctccccaggcggttttttcgtttacagagcaggagattacgacgatcgtaaaaggatctcaagaagatcctttacg
ggggttgactgaattggaagctagtttggcggaggggtccgccaaaaaagcaaatgtctcgtcctctaatgctgctagcattttcctagagttcttctaggaaatgc
ColA ori

pJ2042.2_GFPonly (5858 bp) (from 4816-5858 bp)

gattcccgacaccatcactctagatttcagtgcaatttatctcttcaaatgtagcacctgaagtcagccccatacgatataagttgtaattctcatgttagtcatgc
ctaagggctgtggtagtgagatctaaagtcacgttaaatagagaagtttacatcgtggacttcagtcggggtatgctatattcaacattaagagtacaatcagtacg
ColA ori

cccgcgcccaccggaaggagctgactgggttgCTCCTAgGGTCTGATTCGTTACCAATTATGACAACTTGACGGCTACATCATTCACTTTTTCTTCACAACCGGCAC
gggcgcgggtggccttcctcgactgacccaacGAGGATcCCAGACTAAGCAATGGTTAATACTGTTGAACTGCCGATGTAGTAAGTGAAAAAGAAGTGTTGGCCGTG
araC

GGAACTCGCTCGGGCTGGCCCCGGTGCATTTTTTAAATACCCGCGAGAAATAGAGTTGATCGTCAAAACCAACATTGCGACCGACGGTGGCGATAGGCATCCGGGTG
CCTTGAGCGAGCCCGACCGGGGCCACGTAAAAAATTTATGGGCGCTCTTTATCTCAACTAGCAGTTTTGGTTGTAACGCTGGCTGCCACCGCTATCCGTAGGCCCAC
araC

GTGCTCAAAAGCAGCTTCGCCTGGCTGATACGTTGGTCCTCGCGCCAGCTTAAGACGCTAATCCCTAACTGCTGGCGGAAAAGATGTGACAGACGCGACGGCGACAA
CACGAGTTTTCGTCGAAGCGGACCGACTATGCAACCAGGAGCGCGGTCGAATTCTGCGATTAGGGATTGACGACCGCCTTTTCTACACTGTCTGCGCTGCCGCTGTT
araC

GCAAACATGCTGTGCGACGCTGGCGATATCAAAATTGCTGTCTGCCAGGTGATCGCTGATGTACTGACAAGCCTCGCGTACCCGATTATCCATCGGTGGATGGAGCG
CGTTTGTACGACACGCTGCGACCGCTATAGTTTTAACGACAGACGGTCCACTAGCGACTACATGACTGTTCGGAGCGCATGGGCTAATAGGTAGCCACCTACCTCGC
araC

ACTCGTTAATCGCTTCCATGCGCCGCAGTAACAATTGCTCAAGCAGATTTATCGCCAGCAGCTCCGAATAGCGCCCTTCCCCTTGCCCGGCGTTAATGATTTGCCCA
TGAGCAATTAGCGAAGGTACGCGGCGTCATTGTTAACGAGTTCGTCTAAATAGCGGTCGTCGAGGCTTATCGCGGGAAGGGGAACGGGCCGCAATTACTAAACGGGT
araC

AACAGGTCGCTGAAATGCGGCTGGTGCGCTTCATCCGGGCGAAAGAACCCCGTATTGGCAAATATTGACGGCCAGTTAAGCCATTCATGCCAGTAGGCGCGCGGACG
TTGTCCAGCGACTTTACGCCGACCACGCGAAGTAGGCCCGCTTTCTTGGGGCATAACCGTTTATAACTGCCGGTCAATTCGGTAAGTACGGTCATCCGCGCGCCTGC
araC

AAAGTAAACCCACTGGTGATACCATTCGCGAGCCTCCGGATGACGACCGTAGTGATGAATCTCTCCTGGCGGGAACAGCAAAATATCACCCGGTCGGCAAACAAATT
TTTCATTTGGGTGACCACTATGGTAAGCGCTCGGAGGCCTACTGCTGGCATCACTACTTAGAGAGGACCGCCCTTGTCGTTTTATAGTGGGCCAGCCGTTTGTTTAA
araC

CTCGTCCCTGATTTTTCACCACCCCCTGACCGCGAATGGTGAGATTGAGAATATAACCTTTCATTCCCAGCGGTCGGTCGATAAAAAAATCGAGATAACCGTTGGCC
GAGCAGGGACTAAAAAGTGGTGGGGGACTGGCGCTTACCACTCTAACTCTTATATTGGAAAGTAAGGGTCGCCAGCCAGCTATTTTTTTAGCTCTATTGGCAACCGG
araC

TCAATCGGCGTTAAACCCGCCACCAGATGGGCATTAAACGAGTATCCCGGCAGCAGGGGATCATTTTGCGCTTCAGCCAT
AGTTAGCCGCAATTTGGGCGGTGGTCTACCCGTAATTTGCTCATAGGGCCGTCGTCCCCTAGTAAAACGCGAAGTCGGTA
araC

(from 1-1177 bp)

pJ2042.2_invRep (8003 bp)

ACTTTTCATACTCCCGCCATTCAGAGAAGAAACCAATTGTCCATATTGCATCAGACATTGCCGTCACTGCGTCTTTTACTGGCTCTTCTCGCTAACCAAACCGGTAA
TGAAAAGTATGAGGGCGGTAAGTCTCTTCTTTGGTTAACAGGTATAACGTAGTCTGTAACGGCAGTGACGCAGAAAATGACCGAGAAGAGCGATTGGTTTGGCCATT

CCCCGCTTATTAAAAGCATTCTGTAACAAAGCGGGACCAAAGCCATGACAAAAACGCGTAACAAAAGTGTCTATAATCACGGCAGAAAAGTCCACATTGATTATTTG
GGGGCGAATAATTTTCGTAAGACATTGTTTCGCCCTGGTTTCGGTACTGTTTTTGCGCATTGTTTTCACAGATATTAGTGCCGTCTTTTCAGGTGTAACTAATAAAC

EcoRI
CACGGCGTCACACTTTGCTATGCCATAGCATTTTTATCCATAAGATTAGCGGtTCCTACCTGACGCTTTTTATCGCAACTCTCTACTGTTTCTCCATACCGAATTCA
GTGCCGCAGTGTGAAACGATACGGTATCGTAAAAATAGGTATTCTAATCGCCaAGGATGGACTGCGAAAAATAGCGTTGAGAGATGACAAAGAGGTATGGCTTAAGT
P(BAD) promoter

SacI
TAGGATAGATTCTGGAAACTTTACCGTCCGAGCTCCAGCCTGCGGTCCGGGTTCACTGCCGTATAGGCAGAAGCTAGACTCTAGTGGTTTcAGAGCTATGCTGGAAA
ATCCTATCTAAGACCTTTGAAATGGCAGGCTCGAGGTCGGACGCCAGGCCCAAGTGACGGCATATCCGTCTTCGATCTGAGATCACCAAAgTCTCGATACGACCTTT
Linker_14 Prefix Csy4 site sgRNA-1t4

CAGCATAGCAAGTTgAAATAAGGCTAGTCCGTTATCAACTTGAAAAAGTGGCACCGAGTCGGTGCGTTCACTGCCGTATAGGCAGTCGCTGGGACGCCCGGGGACTA
GTCGTATCGTTCAAcTTTATTCCGATCAGGCAATAGTTGAACTTTTTCACCGTGGCTCAGCCACGCAAGTGACGGCATATCCGTCAGCGACCCTGCGGGCCCCTGAT
sgRNA-1t4 Csy4 site Suffix

CACTTACGAAACTATTGATTGCTCAGCCTGCGGTCCGGGTTCACTGCCGTATAGGCAGATCAGTGTGTACTAAGTACTGTTTcAGAGCTATGCTGGAAACAGCATAG
GTGAATGCTTTGATAACTAACGAGTCGGACGCCAGGCCCAAGTGACGGCATATCCGTCTAGTCACACATGATTCATGACAAAgTCTCGATACGACCTTTGTCGTATC
Linker_1 Prefix Csy4 site sgRNA-2

XhoI
CAAGTTgAAATAAGGCTAGTCCGTTATCAACTTGAAAAAGTGGCACCGAGTCGGTGCGTTCACTGCCGTATAGGCAGTCGCTGGGACGCCCGCTCGAGCAATAAACA
GTTCAAcTTTATTCCGATCAGGCAATAGTTGAACTTTTTCACCGTGGCTCAGCCACGCAAGTGACGGCATATCCGTCAGCGACCCTGCGGGCGAGCTCGTTATTTGT
sgRNA-2 Csy4 site Suffix

GTTGATAGGGCTTCTCCGTTACAGCCTGCGGTCCGGGTTCACTGCCGTATAGGCAGTAATTTTGTTTAACTTTAAGAAGGAGATATACATATGGTTTCGGTTATCAA
CAACTATCCCGAAGAGGCAATGTCGGACGCCAGGCCCAAGTGACGGCATATCCGTCATTAAAACAAATTGAAATTCTTCCTCTATATGTATACCAAAGCCAATAGTT
Linker_0 Prefix Csy4 site RBS mKO2

ACCAGAGATGAAAATGCGTTACTATATGGATGGTTCAGTAAATGGTCACGAATTTACTATTGAGGGCGAGGGTACGGGACGCCCATACGAGGGGCACCAGGAAATGA
TGGTCTCTACTTTTACGCAATGATATACCTACCAAGTCATTTACCAGTGCTTAAATGATAACTCCCGCTCCCATGCCCTGCGGGTATGCTCCCCGTGGTCCTTTACT
mKO2

CTTTACGCGTCACAATGGCTGAAGGCGGGCCTATGCCGTTTGCGTTCGATCTTGTTAGTCATGTCTTTTGTTACGGTCACCGTGTATTTACTAAATACCCCGAGGAA
GAAATGCGCAGTGTTACCGACTTCCGCCCGGATACGGCAAACGCAAGCTAGAACAATCAGTACAGAAAACAATGCCAGTGGCACATAAATGATTTATGGGGCTCCTT
mKO2

ATTCCAGACTATTTCAAACAAGCCTTCCCGGAAGGTTTGTCTTGGGAGCGCAGTTTAGAGTTTGAAGACGGTGGCTCGGCCAGCGTGTCAGCTCATATTAGTCTTCG
TAAGGTCTGATAAAGTTTGTTCGGAAGGGCCTTCCAAACAGAACCCTCGCGTCAAATCTCAAACTTCTGCCACCGAGCCGGTCGCACAGTCGAGTATAATCAGAAGC
mKO2

pJ2042.2_invRep (8003 bp) (from 1178-2354 bp)

CGGCAATACATTTTATCACAAGTCAAAGTTCACCGGCGTGAACTTCCCCGCAGACGGCCCAATCATGCAGAATCAAAGTGTTGATTGGGAACCGTCCACAGAGAAGA
GCCGTTATGTAAAATAGTGTTCAGTTTCAAGTGGCCGCACTTGAAGGGGCGTCTGCCGGGTTAGTACGTCTTAGTTTCACAACTAACCCTTGGCAGGTGTCTCTTCT
mKO2

TTACAGCTTCCGATGGAGTCTTAAAGGGCGATGTAACCATGTACTTAAAATTAGAAGGGGGAGGGAACCATAAATGTCAGATGAAGACTACCTATAAGGCCGCAAAA
AATGTCGAAGGCTACCTCAGAATTTCCCGCTACATTGGTACATGAATTTTAATCTTCCCCCTCCCTTGGTATTTACAGTCTACTTCTGATGGATATTCCGGCGTTTT
mKO2

GAGATTCTTGAAATGCCCGGAGACCACTACATTGGGCATCGTTTGGTCCGTAAGACAGAAGGAAATATTACTGAACAGGTCGAAGACGCTGTGGCACACAGCATGTC
CTCTAAGAACTTTACGGGCCTCTGGTGATGTAACCCGTAGCAAACCAGGCATTCTGTCTTCCTTTATAATGACTTGTCCAGCTTCTGCGACACCGTGTGTCGTACAG
mKO2

CCGCCGTAATACTGACGCCATCACAATCCACAGCATCCTGGATTGGATTGAAGACAACTTGGAGTCGCCGTTGAGTTTAGAAAAAGTTAGTGAACGTAGTGGTTACT
GGCGGCATTATGACTGCGGTAGTGTTAGGTGTCGTAGGACCTAACCTAACTTCTGTTGAACCTCAGCGGCAACTCAAATCTTTTTCAATCACTTGCATCACCAATGA
MarA

CAAAGTGGCACCTTCAGCGCATGTTTAAGAAGGAAACGGGTCATTCATTGGGTCAATATATTCGTTCTCGCAAGATGACTGAAATTGCCCAGAAATTGAAAGAGTCT
GTTTCACCGTGGAAGTCGCGTACAAATTCTTCCTTTGCCCAGTAAGTAACCCAGTTATATAAGCAAGAGCGTTCTACTGACTTTAACGGGTCTTTAACTTTCTCAGA
MarA

AATGAACCTATTTTGTACCTGGCGGAGCGTTACGGCTTTGAAAGTCAGCAAACCCTTACACGTACCTTCAAGAATTACTTTGACGTTCCACCACACAAATATCGTAT
TTACTTGGATAAAACATGGACCGCCTCGCAATGCCGAAACTTTCAGTCGTTTGGGAATGTGCATGGAAGTTCTTAATGAAACTGCAAGGTGGTGTGTTTATAGCATA
MarA

NcoI
GACCAACATGCAGGGTGAGTCACGTTTTTTGCATCCGTTGAATCATTACAATTCCTAATAATCGCTGGGACGCCCGCCATGGTTCAGCCAAAAAACTTAAGACCGCC
CTGGTTGTACGTCCCACTCAGTGCAAAAAACGTAGGCAACTTAGTAATGTTAAGGATTATTAGCGACCCTGCGGGCGGTACCAAGTCGGTTTTTTGAATTCTGGCGG
MarA Suffix ECK120029...rminator

GGTCTTGTCCACTACCTTGCAGTAATGCGGTGGACAGGATCGGCGGTTTTCTTTTCTCTTCTCAATTCTTCTGACCTGTAACGAATAATAGATAGTAAAGTAGTCTC
CCAGAACAGGTGATGGAACGTCATTACGCCACCTGTCCTAGCCGCCAAAAGAAAAGAGAAGAGTTAAGAAGACTGGACATTGCTTATTATCTATCATTTCATCAGAG
ECK120029600 Terminator Spacer 1

CGATTGAGTTTTCTCTGCCGAGTCCCACCCAGTTCTGTGATTTCAGTAAGTTGGTAATTGATACACTGTTGCGAGAACTGCTGCCTGGTAGTAGATAGGTTGTTATT
GCTAACTCAAAAGAGACGGCTCAGGGTGGGTCAAGACACTAAAGTCATTCAACCATTAACTATGTGACAACGCTCTTGACGACGGACCATCATCTATCCAACAATAA
Spacer 1

HindIII
GAGTAAGAAGGTAAAGTGAACGAAATCCCTGAAACTGAGACTGTAGAAAATAAGCTTCAGCCTGCGGTCCGGttgacggctagctcagtcctaggtacagtgctagc
CTCATTCTTCCATTTCACTTGCTTTAGGGACTTTGACTCTGACATCTTTTATTCGAAGTCGGACGCCAGGCCaactgccgatcgagtcaggatccatgtcacgatcg
Spacer 1 Prefix P(BBa_J23100)

BamHI
TCGCTGGGACGCCCGAGATAGCCGTTACACAGGTGACACTTATTTCAGCCTGCGGTCCGGccaCACTAGAGTCTAGCTTGAGATCGCTGGGACGCCCGGGATCCAAG
AGCGACCCTGCGGGCTCTATCGGCAATGTGTCCACTGTGAATAAAGTCGGACGCCAGGCCggtGTGATCTCAGATCGAACTCTAGCGACCCTGCGGGCCCTAGGTTC
Suffix Linker_24 Prefix bs-1 Suffix

pJ2042.2_invRep (8003 bp) (from 2355-3638 bp)

AGATTTCTACACGATTGAGCACTGTCTCAGCCTGCGGTCCGGGTTCACTGCCGTATAGGCAGTAATTTTGTTTAACTTTAAGAAGGAGATATACATATGAATCAGTC
TCTAAAGATGTGCTAACTCGTGACAGAGTCGGACGCCAGGCCCAAGTGACGGCATATCCGTCATTAAAACAAATTGAAATTCTTCCTCTATATGTATACTTAGTCAG
Linker_10 | Prefix | Csy4 site | RBS | RepA70

ATTCATCTCGGACATCTTATATGCCGACATCGAATCGAAGGCTAAGGAACTTACAGTCAATTCCAACAATACTGTCCAGCCGGTCGCGCTTATGCGCTTAGGAGTTT
TAAGTAGAGCCTGTAGAATATACGGCTGTAGCTTAGCTTCCGATTCCTTGAATGTCAGTTAAGGTTGTTATGACAGGTCGGCCAGCGCGAATACGCGAATCCTCAAA
RepA70

TCGTTCCCAAACCTTCCAAGAGCAAAGGAGAAAGTAAGGAAATTGACGCCACCAAAGCCTTCTCTCAACTGGAGATTGCTAAAGCAGAGGGCatggttagtaaagga
AGCAAGGGTTTGGAAGGTTCTCGTTTCCTCTTTCATTCCTTTAACTGCGGTGGTTTCGGAAGAGAGTTGACCTCTAACGATTTCGTCTCCCGtaccaatcatttcct
RepA70 | mKate2

gaagaaaataacatggcaCTGATTAAGGAGAACATGCACATGAAGCTGTACATGGAGGGCACCGTGAACAACCACCACTTCAAGTGCACATCCGAGGGCGAAGGCAA
cttcttttattgtaccgtGACTAATTCCTCTTGTACGTGTACTTCGACATGTACCTCCCGTGGCACTTGTTGGTGGTGAAGTTCACGTGTAGGCTCCCGCTTCCGTT
mKate2

GCCCTACGAGGGCACCCAGACCATGAGAATCAAGgccGTCGAGGGCGGCCCTCTCCCCTTCGCCTTCGACATCCTGGCTACCAGCTTCATGTACGGCAGCAAAACCT
CGGGATGCTCCCGTGGGTCTGGTACTCTTAGTTCcggCAGCTCCCGCCGGGAGAGGGGAAGCGGAAGCTGTAGGACCGATGGTCGAAGTACATGCCGTCGTTTTGGA
mKate2

TCATCAACCACACCCAGGGCATCCCCGACTTCTTTAAGCAGTCCTTCCCTGAGGGCTTCACATGGGAGAGAGTCACCACATACGAAGACGGGGGCGTGCTGACCGCT
AGTAGTTGGTGTGGGTCCCGTAGGGGCTGAAGAAATTCGTCAGGAAGGGACTCCCGAAGTGTACCCTCTCTCAGTGGTGTATGCTTCTGCCCCCGCACGACTGGCGA
mKate2

ACCCAGGACACCAGCCTCCAGGACGGCTGCCTCATCTACAACGTCAAGATCAGAGGGGTGAACTTCCCATCCAACGGCCCTGTGATGCAGAAGAAAACACTCGGCTG
TGGGTCCTGTGGTCGGAGGTCCTGCCGACGGAGTAGATGTTGCAGTTCTAGTCTCCCCACTTGAAGGGTAGGTTGCCGGGACACTACGTCTTCTTTTGTGAGCCGAC
mKate2

GGAGGCCTCCACCGAGaccCTGTACCCCGCTGACGGCGGCCTGGAAGGCAGAgcCGACATGGCCCTGAAGCTCGTGGGCGGGGGCCACCTGATCTGCAACTTGAAGA
CCTCCGGAGGTGGCTCtggGACATGGGGCGACTGCCGCCGGACCTTCCGTCTcgGCTGTACCGGGACTTCGAGCACCCGCCCCCGGTGGACTAGACGTTGAACTTCT
mKate2

CCACATACAGATCCAAGAAACCCGCTAAGAACCTCAAGATGCCCGGCGTCTACTATGTGGACAGAAGACTGGAAAGAATCAAGGAGGCCGACAAAGAGACCTACGTC
GGTGTATGTCTAGGTTCTTTGGGCGATTCTTGGAGTTCTACGGGCCGCAGATGATACACCTGTCTTCTGACCTTTCTTAGTTCCTCCGGCTGTTTCTCTGGATGCAG
mKate2

SbfI KpnI
GAGCAGCACGAGGTGGCTGTGGCCAGATACTGCGACCTCCCTAGCAAACTGGGGCACAgAgtctaATAATCGCTGGGACGCCCGCCTGCAGGCTCGGTACCAAATTC
CTCGTCGTGCTCCACCGACACCGGTCTATGACGCTGGAGGGATCGTTTGACCCCGTGTcTcagatTATTAGCGACCCTGCGGGCGGACGTCCGAGCCATGGTTTAAG
mKate2 | Suffix | | L3S2...tor

CAGAAAAGAGGCCTCCCGAAAGGGGGGCCTTTTTTCGTTTTGGTCCTAATAGATAAAGGATAGGTCTGGTAGTGTTGTTCGTTCTCGCAGGTAAATCAATAATACTC
GTCTTTTCTCCGGAGGGCTTTCCCCCCGGAAAAAAGCAAAACCAGGATTATCTATTTCCTATCCAGACCATCACAACAAGCAAGAGCGTCCATTTAGTTATTATGAG
L3S2P21 Terminator | Spacer 2.5

AGCAGTTCCGTAGACTTTTCAGTGGGACAGGGTAGCGATAACAGATAGATTGTAATAAGACACAGTAGGTGCTCGTAGTTGCGTGAAGAGAACCGCTCAGGAAATCC
TCGTCAAGGCATCTGAAAAGTCACCCTGTCCCATCGCTATTGTCTATCTAACATTATTCTGTGTCATCCACGAGCATCAACGCACTTCTCTTGGCGAGTCCTTTAGG
Spacer 2.5

pJ2042.2_invRep (8003 bp) (from 3639-4815 bp)

SalI

AGTCAGAAGTATTGGTAATCGTTGAAAACTCAGTCGACCAGCCTGCGGTCCGGTTTACGGCTAGCTCAGTCCTAGGTATTATGCTAGCTCGCTGGGACGCCCGAGTG

TCAGTCTTCATAACCATTAGCAACTTTTGAGTCAGCTGGTCGGACGCCAGGCCAAATGCCGATCGAGTCAGGATCCATAATACGATCGAGCGACCCTGCGGGCTCAC

Spacer 2.5 | Prefix | P(BBa_J23150) | Suffix

ACGACTGCGAAGTAACCTCTATTTATCAGCCTGCGGTCCGGccaAGTACTTAGTACACACTGATTCGCTGGGACGCCCGGTAGTGCTTATCAGACCCAATACTGTTG

TGCTGACGCTTCATTGGAGATAAATAGTCGGACGCCAGGCCggtTCATGAATCATGTGTGACTAAGCGACCCTGCGGGCCATCACGAATAGTCTGGGTTATGACAAC

Linker_25 | Prefix | bs-2 | Suffix | Linker_17

AACAGCCTGCGGTCCGGGTTCACTGCCGTATAGGCAGTCTCAAGCTAGACTCTAGTGGTTTcAGAGCTATGCTGGAAACAGCATAGCAAGTTgAAATAAGGCTAGTC

TTGTCGGACGCCAGGCCCAAGTGACGGCATATCCGTCAGAGTTCGATCTGAGATCACCAAAgTCTCGATACGACCTTTGTCGTATCGTTCAAcTTTATTCCGATCAG

Prefix | Csy4 site | sgRNA-1

AatII

CGTTATCAACTTGAAAAAGTGGCACCGAGTCGGTGCGTTCACTGCCGTATAGGCAGTCGCTGGGACGCCCGGACGTCCTATTACACTCGTCGTTGGAAACTGAAGAT

GCAATAGTTGAACTTTTTCACCGTGGCTCAGCCACGCAAGTGACGGCATATCCGTCAGCGACCCTGCGGGCCTGCAGGATAATGTGAGCAGCAACCTTTGACTTCTA

sgRNA-1 | Csy4 site | Suffix | Linker_11

CAGCCTGCGGTCCGGGTTCACTGCCGTATAGGCAGTAATTTTGTTTAACTTTAAGAAGGAGATATACATATGCGTAAAGGCGAAGAACTGTTTACCGGTGTGGTTCC

GTCGGACGCCAGGCCCAAGTGACGGCATATCCGTCATTAAAACAAATTGAAATTCTTCCTCTATATGTATACGCATTTCCGCTTCTTGACAAATGGCCACACCAAGG

Prefix | Csy4 site | RBS | sfGFP

GATTCTGGTGGAACTGGACGGCGATGTTAATGGTCATAAATTCAGTGTTCGCGGCGAAGGTGAAGGCGATGCGACGAACGGCAAACTGACCCTGAAATTTATCTGCA

CTAAGACCACCTTGACCTGCCGCTACAATTACCAGTATTTAAGTCACAAGCGCCGCTTCCACTTCCGCTACGCTGCTTGCCGTTTGACTGGGACTTTAAATAGACGT

sfGFP

CCACGGGTAAACTGCCGGTCCCGTGGCCGACGCTGGTGACCACGCTGACCTATGGCGTTCAATGTTTTGCGCGTTACCCGGATCACATGAAACAGCACGACTTTTTC

GGTGCCCATTTGACGGCCAGGGCACCGGCTGCGACCACTGGTGCGACTGGATACCGCAAGTTACAAAACGCGCAATGGGCCTAGTGTACTTTGTCGTGCTGAAAAAG

sfGFP

AAATCGGCCATGCCGGAAGGCTATGTGCAGGAACGTACGATTAGCTTTAAAGACGATGGTACGTATAAAACCCGCGCGGAAGTGAAATTCGAAGGCGATACCCTGGT

TTTAGCCGGTACGGCCTTCCGATACACGTCCTTGCATGCTAATCGAAATTTCTGCTACCATGCATATTTTGGGCGCGCCTTCACTTTAAGCTTCCGCTATGGGACCA

sfGFP

TAACCGTATCGAACTGAAAGGTATCGATTTCAAAGAAGACGGCAATATTCTGGGTCATAAACTGGAATATAACTTCAATTCCCACAACGTGTACATCACCGCGGATA

ATTGGCATAGCTTGACTTTCCATAGCTAAAGTTTCTTCTGCCGTTATAAGACCCAGTATTTGACCTTATATTGAAGTTAAGGGTGTTGCACATGTAGTGGCGCCTAT

sfGFP

AACAGAAAAACGGCATTAAAGCCAATTTCAAAATCCGCCATAATGTGGAAGATGGTAGCGTTCAGCTGGCCGACCACTATCAGCAAAACACGCCGATTGGTGATGGC

TTGTCTTTTTGCCGTAATTTCGGTTAAAGTTTTAGGCGGTATTACACCTTCTACCATCGCAAGTCGACCGGCTGGTGATAGTCGTTTTGTGCGGCTAACCACTACCG

sfGFP

CCGGTCCTGCTGCCGGACAATCACTACCTGAGTACCCAGTCCGTGCTGTCAAAAGATCCGAACGAAAAACGTGACCACATGGTCCTGCTGGAATTTGTGACGGCTGC

GGCCAGGACGACGGCCTGTTAGTGATGGACTCATGGGTCAGGCACGACAGTTTTCTAGGCTTGCTTTTTGCACTGGTGTACCAGGACGACCTTAAACACTGCCGACG

sfGFP

pJ2042.2_invRep (8003 bp) (from 4816-6099 bp)

GGGTATCACCCACGGCATGGACGAACTGTATAAAATGTCCCGCCGTAATACTGACGCCATCACAATCCACAGCATCCTGGATTGGATTGAAGACTAATAATCGCTGG
CCCATAGTGGGTGCCGTACCTGCTTGACATATTTTACAGGGCGGCATTATGACTGCGGTAGTGTTAGGTGTCGTAGGACCTAACCTAACTTCTGATTATTAGCGACC
sfGFP MarAn20

NotI
GACGCCCGGCGGCCGCggaaacacagAAAAAAGCCCGCACCTGACAGTGCGGGCTTTTTTTTTcgaccaaaggTAGCGAACGACGAGTCACTGTTGAGGATAAATAC
CTGCGGGCCGCCGGCGcctttgtgtcTTTTTTCGGGCGTGGACTGTCACGCCCGAAAAAAAAAgctggtttccATCGCTTGCTGCTCAGTGACAACTCCTATTTATG
ECK120033737 Terminator Linker_8

KasI BbvCI AscI
TTTCTCTACTAGGCGCCTGTTACACAGGTCCTCAGCGGCGCGCCTTTGTCGGTGAACGCTCTCCTGAGTAGGACAAATCCGCCGGGAGCGGATTTGAACGTTGTGAA
AAAGAGATGATCCGCGGACAATGTGTCCAGGAGTCGCCGCGCGGAAACAGCCACTTGCGAGAGGACTCATCCTGTTTAGGCGGCCCTCGCCTAAACTTGCAACACTT
Li...8 Spa...15

GCAACGGCCCGGAGGGTGGCGGGCAGGACGCCCGCCATAAACTGCCAGGCATCAAACTAAGCAGAAGGCCATCCTGACGGATGGCCTTTTTGCGTTTCAGATCTACC
CGTTGCCGGGCCTCCCACCGCCCGTCCTGCGGGCGGTATTTGACGGTCCGTAGTTTGATTCGTCTTCCGGTAGGACTGCCTACCGGAAAAACGCAAAGTCTAGATGG

GGTaaaccagcaatagacataagcggctatttaacgaccctgccctgaaccgacgacaagctgacgaccgggtctccgcaagtggcacttttcggggaaatgtgcgc
CCAtttggtcgttatctgtattcgccgataaattgctgggacgggacttggctgctgttcgactgctggcccagaggcgttcaccgtgaaaagcccctttacacgcg

ggaacccctatttgtttatttttctaaatacattcaaatatgtatccgctcatgaattaattcttagaaaaactcatcgagcatcaaatgaaactgcaatttattca
ccttggggataaacaaataaaaagatttatgtaagtttatacataggcgagtacttaattaagaatctttttgagtagctcgtagtttactttgacgttaaataagt
KanR

tatcaggattatcaataccatatttttgaaaaagccgtttctgtaatgaaggagaaaactcaccgaggcagttccataggatggcaagatcctggtatcggtctgcg
atagtcctaatagttatggtataaaaactttttcggcaaagacattacttcctcttttgagtggctccgtcaaggtatcctaccgttctaggaccatagccagacgc
KanR

attccgactcgtccaacatcaatacaacctattaatttcccctcgtcaaaaataaggttatcaagtgagaaatcaccatgagtgacgactgaatccggtgagaatgg
taaggctgagcaggttgtagttatgttggataattaaaggggagcagtttttattccaatagttcactctttagtggtactcactgctgacttaggccactcttacc
KanR

caaaagtttatgcatttctttccagacttgttcaacaggccagccattacgctcgtcatcaaaatcactcgcatcaaccaaaccgttattcattcgtgattgcgcct
gttttcaaatacgtaaagaaaggtctgaacaagttgtccggtcggtaatgcgagcagtagttttagtgagcgtagttggtttggcaataagtaagcactaacgcgga
KanR

gagcgagacgaaatacgcggtcgctgttaaaaggacaattacaaacaggaatcgaatgcaaccggcgcaggaacactgccagcgcatcaacaatattttcacctgaa
ctcgctctgctttatgcgccagcgacaattttcctgttaatgtttgtccttagcttacgttggccgcgtccttgtgacggtcgcgtagttgttataaaagtggactt
KanR

tcaggatattcttctaatacctggaatgctgttttcccggggatcgcagtggtgagtaaccatgcatcatcaggagtacggataaaatgcttgatggtcggaagagg
agtcctataagaagattatggaccttacgacaaaagggcccctagcgtcaccactcattggtacgtagtagtcctcatgcctattttacgaactaccagccttctcc
KanR

cataaattccgtcagccagtttagtctgaccatctcatctgtaacatcattggcaacgctacctttgccatgtttcagaaacaactctggcgcatcgggcttcccat
gtatttaaggcagtcggtcaaatcagactggtagagtagacattgtagtaaccgttgcgatggaaacggtacaaagtctttgttgagaccgcgtagcccgaagggta
KanR

pJ2042.2_invRep (8003 bp) (from 6100-7383 bp)

acaatcgatagattgtcgcacctgattgcccgacattatcgcgagcccatttatacccatataaatcagcatccatgttggaatttaatcgcggcctagagcaagac
tgttagctatctaacagcgtggactaacgggctgtaatagcgctcgggtaaatatgggtatatttagtcgtaggtacaaccttaaattagcgccggatctcgttctg

KanR

gtttcccgttgaatatggctcatactcttcctttttcaatattattgaagcatttatcagggttattgtctcatgagcggatacatatttgaatgtatttagaaaaa
caaagggcaacttataccgagtatgagaaggaaaaagttataataacttcgtaaatagtcccaataacagagtactcgcctatgtataaacttacataaatcttttt

KanR

taaacaaataggcatgctagcgcagaaacgtcctagaagatgccaggaggatacttagcagagagacaataaggccggagcgaagccgtttttccataggctccgcc
atttgtttatccgtacgatcgcgtctttgcaggatcttctacggtcctcctatgaatcgtctctctgttattccggcctcgcttcggcaaaaaggtatccgaggcgg

ColA ori

cccctgacgaacatcacgaaatctgacgctcaaatcagtggtggcgaaacccgacaggactataaagataccaggcgtttccccctgatggctccctcttgcgctct
ggggactgcttgtagtgctttagactgcgagtttagtcaccaccgctttgggctgtcctgatatttctatggtccgcaaagggggactaccgagggagaacgcgaga

ColA ori

cctgttcccgtcctgcggcgtccgtgttgtggtggaggctttacccaaatcaccacgtcccgttccgtgtagacagttcgctccaagctgggctgtgtgcaagaacc
ggacaagggcaggacgccgcaggcacaacaccacctccgaaatgggtttagtggtgcagggcaaggcacatctgtcaagcgaggttcgacccgacacacgttcttgg

ColA ori

ccccgttcagcccgactgctgcgccttatccggtaactatcatcttgagtccaacccggaaagacacgacaaaacgccactggcagcagccattggtaactgagaat
ggggcaagtcgggctgacgacgcggaataggccattgatagtagaactcaggttgggcctttctgtgctgttttgcggtgaccgtcgtcggtaaccattgactctta

ColA ori

tagtggatttagatatcgagagtcttgaagtggtggcctaacagaggctacactgaaaggacagtatttggtatctgcgctccactaaagccagttaccaggttaag
atcacctaaatctatagctctcagaacttcaccaccggattgtctccgatgtgactttcctgtcataaaccatagacgcgaggtgatttcggtcaatggtccaattc

ColA ori

cagttccccaactgacttaaccttcgatcaaaccgcctccccaggcggttttttcgtttacagagcaggagattacgacgatcgtaaaaggatctcaagaagatcct
gtcaaggggttgactgaattggaagctagtttggcggaggggtccgccaaaaaagcaaatgtctcgtcctctaatgctgctagcattttcctagagttcttctagga

ColA ori

ttacggattcccgacaccatcactctagatttcagtgcaatttatctcttcaaatgtagcacctgaagtcagccccatacgatataagttgtaattctcatgttagt
aatgcctaagggctgtggtagtgagatctaaagtcacgttaaatagagaagtttacatcgtggacttcagtcggggtatgctatattcaacattaagagtacaatca

ColA ori

catgccccgcgcccaccggaaggagctgactgggttgCTCCTAgGGTCTGATTCGTTACCAATTATGACAACTTGACGGCTACATCATTCACTTTTTCTTCACAACC
gtacggggcgcgggtggccttcctcgactgacccaacGAGGATcCCAGACTAAGCAATGGTTAATACTGTTGAACTGCCGATGTAGTAAGTGAAAAAGAAGTGTTGG

araC

GGCACGGAACTCGCTCGGGCTGGCCCCGGTGCATTTTTTAAATACCCGCGAGAAATAGAGTTGATCGTCAAAACCAACATTGCGACCGACGGTGGCGATAGGCATCC
CCGTGCCTTGAGCGAGCCCGACCGGGGCCACGTAAAAAATTTATGGGCGCTCTTTATCTCAACTAGCAGTTTTGGTTGTAACGCTGGCTGCCACCGCTATCCGTAGG

araC

GGGTGGTGCTCAAAAGCAGCTTCGCCTGGCTGATACGTTGGTCCTCGCGCCAGCTTAAGACGCTAATCCCTAACTGCTGGCGGAAAAGATGTGACAGACGCGACGGC
CCCACCACGAGTTTTCGTCGAAGCGGACCGACTATGCAACCAGGAGCGCGGTCGAATTCTGCGATTAGGGATTGACGACCGCCTTTTCTACACTGTCTGCGCTGCCG

araC

pJ2042.2_invRep (8003 bp) (from 7384-8003 bp)

GACAAGCAAACATGCTGTGCGACGCTGGCGATATCAAAATTGCTGTCTGCCAGGTGATCGCTGATGTACTGACAAGCCTCGCGTACCCGATTATCCATCGGTGGATG
CTGTTCGTTTGTACGACACGCTGCGACCGCTATAGTTTTAACGACAGACGGTCCACTAGCGACTACATGACTGTTCGGAGCGCATGGGCTAATAGGTAGCCACCTAC
araC

GAGCGACTCGTTAATCGCTTCCATGCGCCGCAGTAACAATTGCTCAAGCAGATTTATCGCCAGCAGCTCCGAATAGCGCCCTTCCCCTTGCCCGGCGTTAATGATTT
CTCGCTGAGCAATTAGCGAAGGTACGCGGCGTCATTGTTAACGAGTTCGTCTAAATAGCGGTCGTCGAGGCTTATCGCGGGAAGGGGAACGGGCCGCAATTACTAAA
araC

GCCCAAACAGGTCGCTGAAATGCGGCTGGTGCGCTTCATCCGGGCGAAAGAACCCCGTATTGGCAAATATTGACGGCCAGTTAAGCCATTCATGCCAGTAGGCGCGC
CGGGTTTGTCCAGCGACTTTACGCCGACCACGCGAAGTAGGCCCGCTTTCTTGGGGCATAACCGTTTATAACTGCCGGTCAATTCGGTAAGTACGGTCATCCGCGCG
araC

GGACGAAAGTAAACCCACTGGTGATACCATTCGCGAGCCTCCGGATGACGACCGTAGTGATGAATCTCTCCTGGCGGGAACAGCAAAATATCACCCGGTCGGCAAAC
CCTGCTTTCATTTGGGTGACCACTATGGTAAGCGCTCGGAGGCCTACTGCTGGCATCACTACTTAGAGAGGACCGCCCTTGTCGTTTTATAGTGGGCCAGCCGTTTG
araC

AAATTCTCGTCCCTGATTTTTCACCACCCCCTGACCGCGAATGGTGAGATTGAGAATATAACCTTTCATTCCCAGCGGTCGGTCGATAAAAAAATCGAGATAACCGT
TTTAAGAGCAGGGACTAAAAAGTGGTGGGGGACTGGCGCTTACCACTCTAACTCTTATATTGGAAAGTAAGGGTCGCCAGCCAGCTATTTTTTTAGCTCTATTGGCA
araC

TGGCCTCAATCGGCGTTAAACCCGCCACCAGATGGGCATTAAACGAGTATCCCGGCAGCAGGGGATCATTTTGCGCTTCAGCCAT
ACCGGAGTTAGCCGCAATTTGGGCGGTGGTCTACCCGTAATTTGCTCATAGGGCCGTCGTCCCCTAGTAAAACGCGAAGTCGGTA
araC

(from 1-1177 bp)

pJ2043 (7459 bp)

ACTTTTCATACTCCCGCCATTCAGAGAAGAAACCAATTGTCCATATTGCATCAGACATTGCCGTCACTGCGTCTTTTACTGGCTCTTCTCGCTAACCAAACCGGTAA
TGAAAAGTATGAGGGCGGTAAGTCTCTTCTTTGGTTAACAGGTATAACGTAGTCTGTAACGGCAGTGACGCAGAAAATGACCGAGAAGAGCGATTGGTTTGGCCATT

CCCCGCTTATTAAAAGCATTCTGTAACAAAGCGGGACCAAAGCCATGACAAAAACGCGTAACAAAAGTGTCTATAATCACGGCAGAAAAGTCCACATTGATTATTTG
GGGGCGAATAATTTTCGTAAGACATTGTTTCGCCCTGGTTTCGGTACTGTTTTTGCGCATTGTTTTCACAGATATTAGTGCCGTCTTTTCAGGTGTAACTAATAAAC

EcoRI

CACGGCGTCACACTTTGCTATGCCATAGCATTTTTATCCATAAGATTAGCGGtTCCTACCTGACGCTTTTTATCGCAACTCTCTACTGTTTCTCCATACCGAATTCA
GTGCCGCAGTGTGAAACGATACGGTATCGTAAAAATAGGTATTCTAATCGCCaAGGATGGACTGCGAAAAATAGCGTTGAGAGATGACAAAGAGGTATGGCTTAAGT

P(BAD) promoter

SacI

TAGGATAGATTCTGGAAACTTTACCGTCCGAGCTCCAGCCTGCGGTCCGGGTTCACTGCCGTATAGGCAGATCAGTGTGTACTAAGTACTGTTTcAGAGCTATGCTG
ATCCTATCTAAGACCTTTGAAATGGCAGGCTCGAGGTCGGACGCCAGGCCCAAGTGACGGCATATCCGTCTAGTCACACATGATTCATGACAAAgTCTCGATACGAC

Linker_14 | Prefix | Csy4 site | sgRNA-2

XhoI

GAAACAGCATAGCAAGTTgAAATAAGGCTAGTCCGTTATCAACTTGAAAAAGTGGCACCGAGTCGGTGCGTTCACTGCCGTATAGGCAGTCGCTGGGACGCCCGCTC
CTTTGTCGTATCGTTCAAcTTTATTCCGATCAGGCAATAGTTGAACTTTTTCACCGTGGCTCAGCCACGCAAGTGACGGCATATCCGTCAGCGACCCTGCGGGCGAG

sgRNA-2 | Csy4 site | Suffix

GAGCAATAAACAGTTGATAGGGCTTCTCCGTTACAGCCTGCGGTCCGGGTTCACTGCCGTATAGGCAGTAATTTTGTTTAACTTTAAGAAGGAGATATACATATGGT
CTCGTTATTTGTCAACTATCCCGAAGAGGCAATGTCGGACGCCAGGCCCAAGTGACGGCATATCCGTCATTAAAACAAATTGAAATTCTTCCTCTATATGTATACCA

Linker_0 | Prefix | Csy4 site | RBS

TTCGGTTATCAAACCAGAGATGAAAATGCGTTACTATATGGATGGTTCAGTAAATGGTCACGAATTTACTATTGAGGGCGAGGGTACGGGACGCCCATACGAGGGGC
AAGCCAATAGTTTGGTCTCTACTTTTACGCAATGATATACCTACCAAGTCATTTACCAGTGCTTAAATGATAACTCCCGCTCCCATGCCCTGCGGGTATGCTCCCCG

mKO2

ACCAGGAAATGACTTTACGCGTCACAATGGCTGAAGGCGGGCCTATGCCGTTTGCGTTCGATCTTGTTAGTCATGTCTTTTGTTACGGTCACCGTGTATTTACTAAA
TGGTCCTTTACTGAAATGCGCAGTGTTACCGACTTCCGCCCGGATACGGCAAACGCAAGCTAGAACAATCAGTACAGAAAACAATGCCAGTGGCACATAAATGATTT

mKO2

TACCCCGAGGAAATTCCAGACTATTTCAAACAAGCCTTCCCGGAAGGTTTGTCTTGGGAGCGCAGTTTAGAGTTTGAAGACGGTGGCTCGGCCAGCGTGTCAGCTCA
ATGGGGCTCCTTTAAGGTCTGATAAAGTTTGTTCGGAAGGGCCTTCCAAACAGAACCCTCGCGTCAAATCTCAAACTTCTGCCACCGAGCCGGTCGCACAGTCGAGT

mKO2

TATTAGTCTTCGCGGCAATACATTTTATCACAAGTCAAAGTTCACCGGCGTGAACTTCCCCGCAGACGGCCCAATCATGCAGAATCAAAGTGTTGATTGGGAACCGT
ATAATCAGAAGCGCCGTTATGTAAAATAGTGTTCAGTTTCAAGTGGCCGCACTTGAAGGGGCGTCTGCCGGGTTAGTACGTCTTAGTTTCACAACTAACCCTTGGCA

mKO2

CCACAGAGAAGATTACAGCTTCCGATGGAGTCTTAAAGGGCGATGTAACCATGTACTTAAAATTAGAAGGGGGAGGGAACCATAAATGTCAGATGAAGACTACCTAT
GGTGTCTCTTCTAATGTCGAAGGCTACCTCAGAATTTCCCGCTACATTGGTACATGAATTTTAATCTTCCCCCTCCCTTGGTATTTACAGTCTACTTCTGATGGATA

mKO2

pJ2043 (7459 bp) (from 1178-2354 bp)

AAGGCCGCAAAAGAGATTCTTGAAATGCCCGGAGACCACTACATTGGGCATCGTTTGGTCCGTAAGACAGAAGGAAATATTACTGAACAGGTCGAAGACGCTGTGGC
TTCCGGCGTTTTCTCTAAGAACTTTACGGGCCTCTGGTGATGTAACCCGTAGCAAACCAGGCATTCTGTCTTCCTTTATAATGACTTGTCCAGCTTCTGCGACACCG
mKO2

ACACAGCATGTCCCGCCGTAATACTGACGCCATCACAATCCACAGCATCCTGGATTGGATTGAAGACAACTTGGAGTCGCCGTTGAGTTTAGAAAAAGTTAGTGAAC
TGTGTCGTACAGGGCGGCATTATGACTGCGGTAGTGTTAGGTGTCGTAGGACCTAACCTAACTTCTGTTGAACCTCAGCGGCAACTCAAATCTTTTTCAATCACTTG
MarA

GTAGTGGTTACTCAAAGTGGCACCTTCAGCGCATGTTTAAGAAGGAAACGGGTCATTCATTGGGTCAATATATTCGTTCTCGCAAGATGACTGAAATTGCCCAGAAA
CATCACCAATGAGTTTCACCGTGGAAGTCGCGTACAAATTCTTCCTTTGCCCAGTAAGTAACCCAGTTATATAAGCAAGAGCGTTCTACTGACTTTAACGGGTCTTT
MarA

TTGAAAGAGTCTAATGAACCTATTTTGTACCTGGCGGAGCGTTACGGCTTTGAAAGTCAGCAAACCCTTACACGTACCTTCAAGAATTACTTTGACGTTCCACCACA
AACTTTCTCAGATTACTTGGATAAAACATGGACCGCCTCGCAATGCCGAAACTTTCAGTCGTTTGGGAATGTGCATGGAAGTTCTTAATGAAACTGCAAGGTGGTGT
MarA

NcoI
CAAATATCGTATGACCAACATGCAGGGTGAGTCACGTTTTTTGCATCCGTTGAATCATTACAATTCCTAATAATCGCTGGGACGCCCGCCATGGTTCAGCCAAAAAA
GTTTATAGCATACTGGTTGTACGTCCCACTCAGTGCAAAAAACGTAGGCAACTTAGTAATGTTAAGGATTATTAGCGACCCTGCGGGCGGTACCAAGTCGGTTTTTT
MarA | Suffix | ECK...or

CTTAAGACCGCCGGTCTTGTCCACTACCTTGCAGTAATGCGGTGGACAGGATCGGCGGTTTTCTTTTCTCTTCTCAATTCTTCTGACCTGTAACGAATAATAGATAG
GAATTCTGGCGGCCAGAACAGGTGATGGAACGTCATTACGCCACCTGTCCTAGCCGCCAAAAGAAAAGAGAAGAGTTAAGAAGACTGGACATTGCTTATTATCTATC
ECK120029600 Terminator | Spacer 1

TAAAGTAGTCTCCGATTGAGTTTTCTCTGCCGAGTCCCACCCAGTTCTGTGATTTCAGTAAGTTGGTAATTGATACACTGTTGCGAGAACTGCTGCCTGGTAGTAGA
ATTTCATCAGAGGCTAACTCAAAAGAGACGGCTCAGGGTGGGTCAAGACACTAAAGTCATTCAACCATTAACTATGTGACAACGCTCTTGACGACGGACCATCATCT
Spacer 1

HindIII
TAGGTTGTTATTGAGTAAGAAGGTAAAGTGAACGAAATCCCTGAAACTGAGACTGTAGAAAATAAGCTTCAGCCTGCGGTCCGGTTGACAGCTAGCTCAGTCCTAGG
ATCCAACAATAACTCATTCTTCCATTTCACTTGCTTTAGGGACTTTGACTCTGACATCTTTTATTCGAAGTCGGACGCCAGGCCAACTGTCGATCGAGTCAGGATCC
Spacer 1 | Prefix | P(BBa_J23102)

TACTGTGCTAGCTCGCTGGGACGCCCGGGGACTACACTTACGAAACTATTGATTGCTCAGCCTGCGGTCCGGccaAGTACTTAGTACACACTGATTCGCTGGGACGC
ATGACACGATCGAGCGACCCTGCGGGCCCCTGATGTGAATGCTTTGATAACTAACGAGTCGGACGCCAGGCCggtTCATGAATCATGTGTGACTAAGCGACCCTGCG
P(B...2) | Suffix | Linker_1 | Prefix | bs-2 | Suffix

BamHI
CCGGGATCCAAGAGATTTCTACACGATTGAGCACTGTCTCAGCCTGCGGTCCGGGTTCACTGCCGTATAGGCAGTAATTTTGTTTAACTTTAAGAAGGAGATATACA
GGCCCTAGGTTCTCTAAAGATGTGCTAACTCGTGACAGAGTCGGACGCCAGGCCCAAGTGACGGCATATCCGTCATTAAAACAAATTGAAATTCTTCCTCTATATGT
Linker_10 | Prefix | Csy4 site | RBS

TATGCGTAAAGGCGAAGAACTGTTTACCGGTGTGGTTCCGATTCTGGTGGAACTGGACGGCGATGTTAATGGTCATAAATTCAGTGTTCGCGGCGAAGGTGAAGGCG
ATACGCATTTCCGCTTCTTGACAAATGGCCACACCAAGGCTAAGACCACCTTGACCTGCCGCTACAATTACCAGTATTTAAGTCACAAGCGCCGCTTCCACTTCCGC
sfGFP

pJ2043 (7459 bp) (from 2355-3531 bp)

```
ATGCGACGAACGGCAAACTGACCCTGAAATTTATCTGCACCACGGGTAAACTGCCGGTCCCGTGGCCGACGCTGGTGACCACGCTGACCTATGGCGTTCAATGTTTT
TACGCTGCTTGCCGTTTGACTGGGACTTTAAATAGACGTGGTGCCCATTTGACGGCCAGGGCACCGGCTGCGACCACTGGTGCGACTGGATACCGCAAGTTACAAAA
```
sfGFP

```
GCGCGTTACCCGGATCACATGAAACAGCACGACTTTTTCAAATCGGCCATGCCGGAAGGCTATGTGCAGGAACGTACGATTAGCTTTAAAGACGATGGTACGTATAA
CGCGCAATGGGCCTAGTGTACTTTGTCGTGCTGAAAAAGTTTAGCCGGTACGGCCTTCCGATACACGTCCTTGCATGCTAATCGAAATTTCTGCTACCATGCATATT
```
sfGFP

```
AACCCGCGCGGAAGTGAAATTCGAAGGCGATACCCTGGTTAACCGTATCGAACTGAAAGGTATCGATTTCAAAGAAGACGGCAATATTCTGGGTCATAAACTGGAAT
TTGGGCGCGCCTTCACTTTAAGCTTCCGCTATGGGACCAATTGGCATAGCTTGACTTTCCATAGCTAAAGTTTCTTCTGCCGTTATAAGACCCAGTATTTGACCTTA
```
sfGFP

```
ATAACTTCAATTCCCACAACGTGTACATCACCGCGGATAAACAGAAAAACGGCATTAAAGCCAATTTCAAAATCCGCCATAATGTGGAAGATGGTAGCGTTCAGCTG
TATTGAAGTTAAGGGTGTTGCACATGTAGTGGCGCCTATTTGTCTTTTTGCCGTAATTTCGGTTAAAGTTTTAGGCGGTATTACACCTTCTACCATCGCAAGTCGAC
```
sfGFP

```
GCCGACCACTATCAGCAAAACACGCCGATTGGTGATGGCCCGGTCCTGCTGCCGGACAATCACTACCTGAGTACCCAGTCCGTGCTGTCAAAAGATCCGAACGAAAA
CGGCTGGTGATAGTCGTTTTGTGCGGCTAACCACTACCGGGCCAGGACGACGGCCTGTTAGTGATGGACTCATGGGTCAGGCACGACAGTTTTCTAGGCTTGCTTTT
```
sfGFP

```
ACGTGACCACATGGTCCTGCTGGAATTTGTGACGGCTGCGGGTATCACCCACGGCATGGACGAACTGTATAAAATGTCCCGCCGTAATACTGACGCCATCACAATCC
TGCACTGGTGTACCAGGACGACCTTAAACACTGCCGACGCCCATAGTGGGTGCCGTACCTGCTTGACATATTTTACAGGGCGGCATTATGACTGCGGTAGTGTTAGG
```
sfGFP | MarAn20

SbfI | KpnI

```
ACAGCATCCTGGATTGGATTGAAGACTAATAATCGCTGGGACGCCCGCCTGCAGGCTCGGTACCAAATTCCAGAAAAGAGGCCTCCCGAAAGGGGGGCCTTTTTTCG
TGTCGTAGGACCTAACCTAACTTCTGATTATTAGCGACCCTGCGGGCGGACGTCCGAGCCATGGTTTAAGGTCTTTTCTCCGGAGGGCTTTCCCCCCGGAAAAAAGC
```
MarAn20 | Suffix | L3S2P21 Terminator

```
TTTTGGTCCTAATAGATAAAGGATAGGTCTGGTAGTGTTGTTCGTTCTCGCAGGTAAATCAATAATACTCAGCAGTTCCGTAGACTTTTCAGTGGGACAGGGTAGCG
AAAACCAGGATTATCTATTTCCTATCCAGACCATCACAACAAGCAAGAGCGTCCATTTAGTTATTATGAGTCGTCAAGGCATCTGAAAAGTCACCCTGTCCCATCGC
```
Spacer 2.5

SalI

```
ATAACAGATAGATTGTAATAAGACACAGTAGGTGCTCGTAGTTGCGTGAAGAGAACCGCTCAGGAAATCCAGTCAGAAGTATTGGTAATCGTTGAAAACTCAGTCGA
TATTGTCTATCTAACATTATTCTGTGTCATCCACGAGCATCAACGCACTTCTCTTGGCGAGTCCTTTAGGTCAGTCTTCATAACCATTAGCAACTTTTGAGTCAGCT
```
Spacer 2.5

AatII

```
CGCACTTACTGAAGACGTCCTATTACACTCGTCGTTGGAAACTGAAGATCAGCCTGCGGTCCGGGTTCACTGCCGTATAGGCAGTAATTTTGTTTAACTTTAAGAAG
GCGTGAATGACTTCTGCAGGATAATGTGAGCAGCAACCTTTGACTTCTAGTCGGACGCCAGGCCCAAGTGACGGCATATCCGTCATTAAAACAAATTGAAATTCTTC
```
Spa... 5 | Linker_11 | Prefix | Csy4 site | RBS

```
GAGATATACATATGAATCAGTCATTCATCTCGGACATCTTATATGCCGACATCGAATCGAAGGCTAAGGAACTTACAGTCAATTCCAACAATACTGTCCAGCCGGTC
CTCTATATGTATACTTAGTCAGTAAGTAGAGCCTGTAGAATATACGGCTGTAGCTTAGCTTCCGATTCCTTGAATGTCAGTTAAGGTTGTTATGACAGGTCGGCCAG
```
RBS | RepA70

pJ2043 (7459 bp) (from 3532-4815 bp)

GCGCTTATGCGCTTAGGAGTTTTCGTTCCCAAACCTTCCAAGAGCAAAGGAGAAAGTAAGGAAATTGACGCCACCAAAGCCTTCTCTCAACTGGAGATTGCTAAAGC
CGCGAATACGCGAATCCTCAAAAGCAAGGGTTTGGAAGGTTCTCGTTTCCTCTTTCATTCCTTTAACTGCGGTGGTTTCGGAAGAGAGTTGACCTCTAACGATTTCG
RepA70

AGAGGGCatggttagtaaaggagaagaaaataacatggcaCTGATTAAGGAGAACATGCACATGAAGCTGTACATGGAGGGCACCGTGAACAACCACCACTTCAAGT
TCTCCCGtaccaatcatttcctcttcttttattgtaccgtGACTAATTCCTCTTGTACGTGTACTTCGACATGTACCTCCCGTGGCACTTGTTGGTGGTGAAGTTCA
mKate2

GCACATCCGAGGGCGAAGGCAAGCCCTACGAGGGCACCCAGACCATGAGAATCAAGgccGTCGAGGGCGGCCCTCTCCCCTTCGCCTTCGACATCCTGGCTACCAGC
CGTGTAGGCTCCCGCTTCCGTTCGGGATGCTCCCGTGGGTCTGGTACTCTTAGTTCcggCAGCTCCCGCCGGGAGAGGGGAAGCGGAAGCTGTAGGACCGATGGTCG
mKate2

TTCATGTACGGCAGCAAAACCTTCATCAACCACACCCAGGGCATCCCCGACTTCTTTAAGCAGTCCTTCCCTGAGGGCTTCACATGGGAGAGAGTCACCACATACGA
AAGTACATGCCGTCGTTTTGGAAGTAGTTGGTGTGGGTCCCGTAGGGGCTGAAGAAATTCGTCAGGAAGGGACTCCCGAAGTGTACCCTCTCTCAGTGGTGTATGCT
mKate2

AGACGGGGGCGTGCTGACCGCTACCCAGGACACCAGCCTCCAGGACGGCTGCCTCATCTACAACGTCAAGATCAGAGGGGTGAACTTCCCATCCAACGGCCCTGTGA
TCTGCCCCCGCACGACTGGCGATGGGTCCTGTGGTCGGAGGTCCTGCCGACGGAGTAGATGTTGCAGTTCTAGTCTCCCCACTTGAAGGGTAGGTTGCCGGGACACT
mKate2

TGCAGAAGAAAACACTCGGCTGGGAGGCCTCCACCGAGaccCTGTACCCCGCTGACGGCGGCCTGGAAGGCAGAgcCGACATGGCCCTGAAGCTCGTGGGCGGGGGC
ACGTCTTCTTTTGTGAGCCGACCCTCCGGAGGTGGCTCtggGACATGGGGCGACTGCCGCCGGACCTTCCGTCTcgGCTGTACCGGGACTTCGAGCACCCGCCCCCG
mKate2

CACCTGATCTGCAACTTGAAGACCACATACAGATCCAAGAAACCCGCTAAGAACCTCAAGATGCCCGGCGTCTACTATGTGGACAGAAGACTGGAAAGAATCAAGGA
GTGGACTAGACGTTGAACTTCTGGTGTATGTCTAGGTTCTTTGGGCGATTCTTGGAGTTCTACGGGCCGCAGATGATACACCTGTCTTCTGACCTTTCTTAGTTCCT
mKate2

GGCCGACAAAGAGACCTACGTCGAGCAGCACGAGGTGGCTGTGGCCAGATACTGCGACCTCCCTAGCAAACTGGGGCACAgAgtctaATAATCGCTGGGACGCCCGG
CCGGCTGTTTCTCTGGATGCAGCTCGTCGTGCTCCACCGACACCGGTCTATGACGCTGGAGGGATCGTTTGACCCCGTGTcTcagatTATTAGCGACCCTGCGGGCC
mKate2 Suffix

NotI

CGGCCGCggaaacacagAAAAAAGCCCGCACCTGACAGTGCGGGCTTTTTTTTTcgaccaaaggTAGCGAACGACGAGTCACTGTTGAGGATAAATACTTTCTCTAC
GCCGGCGcctttgtgtcTTTTTTCGGGCGTGGACTGTCACGCCCGAAAAAAAAAgctggtttccATCGCTTGCTGCTCAGTGACAACTCCTATTTATGAAAGAGATG
ECK120033737 Terminator Linker_8

KasI BbvCI AscI

TAGGCGCCTGTTACACAGGTCCTCAGCGGCGCGCCTTTGTCGGTGAACGCTCTCCTGAGTAGGACAAATCCGCCGGGAGCGGATTTGAACGTTGTGAAGCAACGGCC
ATCCGCGGACAATGTGTCCAGGAGTCGCCGCGCGGAAACAGCCACTTGCGAGAGGACTCATCCTGTTTAGGCGGCCCTCGCCTAAACTTGCAACACTTCGTTGCCGG
Spa...15

CGGAGGGTGGCGGGCAGGACGCCCGCCATAAACTGCCAGGCATCAAACTAAGCAGAAGGCCATCCTGACGGATGGCCTTTTTGCGTTTCAGATCTACCGGTaaacca
GCCTCCCACCGCCCGTCCTGCGGGCGGTATTTGACGGTCCGTAGTTTGATTCGTCTTCCGGTAGGACTGCCTACCGGAAAAACGCAAAGTCTAGATGGCCAtttggt

gcaatagacataagcggctatttaacgaccctgccctgaaccgacgacaagctgacgaccgggtctccgcaagtggcacttttcggggaaatgtgcgcggaacccct
cgttatctgtattcgccgataaattgctgggacgggacttggctgctgttcgactgctggcccagaggcgttcaccgtgaaaagcccctttacacgcgccttggggga

pJ2043 (7459 bp) (from 4816-6099 bp)

atttgtttatttttctaaatacattcaaatatgtatccgctcatgaattaattcttagaaaaactcatcgagcatcaaatgaaactgcaatttattcatatcaggat
taaacaaataaaaagatttatgtaagtttatacataggcgagtacttaattaagaatctttttgagtagctcgtagtttactttgacgttaaataagtatagtccta
KanR

tatcaataccatatttttgaaaaagccgtttctgtaatgaaggagaaaactcaccgaggcagttccataggatggcaagatcctggtatcggtctgcgattccgact
atagttatggtataaaaactttttcggcaaagacattacttcctcttttgagtggctccgtcaaggtatcctaccgttctaggaccatagccagacgctaaggctga
KanR

cgtccaacatcaatacaacctattaatttcccctcgtcaaaaataaggttatcaagtgagaaatcaccatgagtgacgactgaatccggtgagaatggcaaaagttt
gcaggttgtagttatgttggataattaaaggggagcagtttttattccaatagttcactctttagtggtactcactgctgacttaggccactcttaccgttttcaaa
KanR

atgcatttctttccagacttgttcaacaggccagccattacgctcgtcatcaaaatcactcgcatcaaccaaaccgttattcattcgtgattgcgcctgagcgagac
tacgtaaagaaaggtctgaacaagttgtccggtcggtaatgcgagcagtagttttagtgagcgtagttggtttggcaataagtaagcactaacgcggactcgctctg
KanR

gaaatacgcggtcgctgttaaaaggacaattacaaacaggaatcgaatgcaaccggcgcaggaacactgccagcgcatcaacaatattttcacctgaatcaggatat
ctttatgcgccagcgacaattttcctgttaatgtttgtccttagcttacgttggccgcgtccttgtgacggtcgcgtagttgttataaaagtggacttagtcctata
KanR

tcttctaatacctggaatgctgttttcccggggatcgcagtggtgagtaaccatgcatcatcaggagtacggataaaatgcttgatggtcggaagaggcataaattc
agaagattatggaccttacgacaaaagggcccctagcgtcaccactcattggtacgtagtagtcctcatgcctattttacgaactaccagccttctccgtatttaag
KanR

cgtcagccagtttagtctgaccatctcatctgtaacatcattggcaacgctacctttgccatgtttcagaaacaactctggcgcatcgggcttcccatacaatcgat
gcagtcggtcaaatcagactggtagagtagacattgtagtaaccgttgcgatggaaacggtacaaagtctttgttgagaccgcgtagcccgaagggtatgttagcta
KanR

agattgtcgcacctgattgcccgacattatcgcgagcccatttatacccatataaatcagcatccatgttggaatttaatcgcggcctagagcaagacgtttcccgt
tctaacagcgtggactaacgggctgtaatagcgctcgggtaaatatgggtatatttagtcgtaggtacaaccttaaattagcgccggatctcgttctgcaaagggca
KanR

tgaatatggctcatactcttcctttttcaatattattgaagcatttatcagggttattgtctcatgagcggatacatatttgaatgtatttagaaaaataaacaaat
acttataccgagtatgagaaggaaaaagttataataacttcgtaaatagtcccaataacagagtactcgcctatgtataaacttacataaatctttttatttgttta
KanR

aggcatgctagcgcagaaacgtcctagaagatgccaggaggatacttagcagagagacaataaggccggagcgaagccgtttttccataggctccgcccccctgacg
tccgtacgatcgcgtctttgcaggatcttctacggtcctcctatgaatcgtctctctgttattccggcctcgcttcggcaaaaaggtatccgaggcggggggactgc
ColA ori

aacatcacgaaatctgacgctcaaatcagtggtggcgaaacccgacaggactataaagataccaggcgtttccccctgatggctccctcttgcgctctcctgttccc
ttgtagtgctttagactgcgagtttagtcaccaccgctttgggctgtcctgatatttctatggtccgcaaagggggactaccgagggagaacgcgagaggacaaggg
ColA ori

gtcctgcggcgtccgtgttgtggtggaggctttacccaaatcaccacgtcccgttccgtgtagacagttcgctccaagctgggctgtgtgcaagaaccccccgttca
caggacgccgcaggcacaacaccacctccgaaatgggtttagtggtgcagggcaaggcacatctgtcaagcgaggttcgacccgacacacgttcttggggggcaagt
ColA ori

pJ2043 (7459 bp) (from 6100-7383 bp)

```
gcccgactgctgcgccttatccggtaactatcatcttgagtccaacccggaaagacacgacaaaacgccactggcagcagccattggtaactgagaattagtggatt
cgggctgacgacgcggaataggccattgatagtagaactcaggttgggcctttctgtgctgttttgcggtgaccgtcgtcggtaaccattgactcttaatcacctaa
ColA ori

tagatatcgagagtcttgaagtggtggcctaacagaggctacactgaaaggacagtatttggtatctgcgctccactaaagccagttaccaggttaagcagttcccc
atctatagctctcagaacttcaccaccggattgtctccgatgtgactttcctgtcataaaccatagacgcgaggtgatttcggtcaatggtccaattcgtcaagggg
ColA ori

aactgacttaaccttcgatcaaaccgcctccccaggcggttttttcgtttacagagcaggagattacgacgatcgtaaaaggatctcaagaagatcctttacggatt
ttgactgaattggaagctagtttggcggaggggtccgccaaaaaagcaaatgtctcgtcctctaatgctgctagcattttcctagagttcttctaggaaatgcctaa
ColA ori

cccgacaccatcactctagatttcagtgcaatttatctcttcaaatgtagcacctgaagtcagccccatacgatataagttgtaattctcatgttagtcatgccccg
gggctgtggtagtgagatctaaagtcacgttaaatagagaagtttacatcgtggacttcagtcggggtatgctatattcaacattaagagtacaatcagtacggggc
Co...i

cgcccaccggaaggagctgactgggttgCTCCTAgGGTCTGATTCGTTACCAATTATGACAACTTGACGGCTACATCATTCACTTTTTCTTCACAACCGGCACGGAA
gcgggtggccttcctcgactgacccaacGAGGATcCCAGACTAAGCAATGGTTAATACTGTTGAACTGCCGATGTAGTAAGTGAAAAAGAAGTGTTGGCCGTGCCTT
araC

CTCGCTCGGGCTGGCCCCGGTGCATTTTTTAAATACCCGCGAGAAATAGAGTTGATCGTCAAAACCAACATTGCGACCGACGGTGGCGATAGGCATCCGGGTGGTGC
GAGCGAGCCCGACCGGGGCCACGTAAAAAATTTATGGGCGCTCTTTATCTCAACTAGCAGTTTTGGTTGTAACGCTGGCTGCCACCGCTATCCGTAGGCCCACCACG
araC

TCAAAAGCAGCTTCGCCTGGCTGATACGTTGGTCCTCGCGCCAGCTTAAGACGCTAATCCCTAACTGCTGGCGGAAAAGATGTGACAGACGCGACGGCGACAAGCAA
AGTTTTCGTCGAAGCGGACCGACTATGCAACCAGGAGCGCGGTCGAATTCTGCGATTAGGGATTGACGACCGCCTTTTCTACACTGTCTGCGCTGCCGCTGTTCGTT
araC

ACATGCTGTGCGACGCTGGCGATATCAAAATTGCTGTCTGCCAGGTGATCGCTGATGTACTGACAAGCCTCGCGTACCCGATTATCCATCGGTGGATGGAGCGACTC
TGTACGACACGCTGCGACCGCTATAGTTTTAACGACAGACGGTCCACTAGCGACTACATGACTGTTCGGAGCGCATGGGCTAATAGGTAGCCACCTACCTCGCTGAG
araC

GTTAATCGCTTCCATGCGCCGCAGTAACAATTGCTCAAGCAGATTTATCGCCAGCAGCTCCGAATAGCGCCCTTCCCCTTGCCCGGCGTTAATGATTTGCCCAAACA
CAATTAGCGAAGGTACGCGGCGTCATTGTTAACGAGTTCGTCTAAATAGCGGTCGTCGAGGCTTATCGCGGGAAGGGGAACGGGCCGCAATTACTAAACGGGTTTGT
araC

GGTCGCTGAAATGCGGCTGGTGCGCTTCATCCGGGCGAAAGAACCCCGTATTGGCAAATATTGACGGCCAGTTAAGCCATTCATGCCAGTAGGCGCGCGGACGAAAG
CCAGCGACTTTACGCCGACCACGCGAAGTAGGCCCGCTTTCTTGGGGCATAACCGTTTATAACTGCCGGTCAATTCGGTAAGTACGGTCATCCGCGCGCCTGCTTTC
araC

TAAACCCACTGGTGATACCATTCGCGAGCCTCCGGATGACGACCGTAGTGATGAATCTCTCCTGGCGGGAACAGCAAAATATCACCCGGTCGGCAAACAAATTCTCG
ATTTGGGTGACCACTATGGTAAGCGCTCGGAGGCCTACTGCTGGCATCACTACTTAGAGAGGACCGCCCTTGTCGTTTTATAGTGGGCCAGCCGTTTGTTTAAGAGC
araC

TCCCTGATTTTTCACCACCCCCTGACCGCGAATGGTGAGATTGAGAATATAACCTTTCATTCCCAGCGGTCGGTCGATAAAAAAATCGAGATAACCGTTGGCCTCAA
AGGGACTAAAAAGTGGTGGGGGACTGGCGCTTACCACTCTAACTCTTATATTGGAAAGTAAGGGTCGCCAGCCAGCTATTTTTTTAGCTCTATTGGCAACCGGAGTT
araC
```

pJ2043 (7459 bp) (from 7384-7459 bp)

```
TCGGCGTTAAACCCGCCACCAGATGGGCATTAAACGAGTATCCCGGCAGCAGGGGATCATTTTGCGCTTCAGCCAT
AGCCGCAATTTGGGCGGTGGTCTACCCGTAATTTGCTCATAGGGCCGTCGTCCCCTAGTAAAACGCGAAGTCGGTA
```

araC

(from 1-1284 bp)

pJ2043_N2only (7295 bp)

ACTTTTCATACTCCCGCCATTCAGAGAAGAAACCAATTGTCCATATTGCATCAGACATTGCCGTCACTGCGTCTTTTACTGGCTCTTCTCGCTAACCAAACCGGTAA
TGAAAAGTATGAGGGCGGTAAGTCTCTTCTTTGGTTAACAGGTATAACGTAGTCTGTAACGGCAGTGACGCAGAAAATGACCGAGAAGAGCGATTGGTTTGGCCATT

CCCCGCTTATTAAAAGCATTCTGTAACAAAGCGGGACCAAAGCCATGACAAAAACGCGTAACAAAAGTGTCTATAATCACGGCAGAAAAGTCCACATTGATTATTTG
GGGGCGAATAATTTTCGTAAGACATTGTTTCGCCCTGGTTTCGGTACTGTTTTTGCGCATTGTTTTCACAGATATTAGTGCCGTCTTTTCAGGTGTAACTAATAAAC

EcoRI

CACGGCGTCACACTTTGCTATGCCATAGCATTTTTATCCATAAGATTAGCGGtTCCTACCTGACGCTTTTTATCGCAACTCTCTACTGTTTCTCCATACCGAATTCA
GTGCCGCAGTGTGAAACGATACGGTATCGTAAAAATAGGTATTCTAATCGCCaAGGATGGACTGCGAAAAATAGCGTTGAGAGATGACAAAGAGGTATGGCTTAAGT

P(BAD) promoter

SacI XhoI

TAGGATAGATTCTGGAAACTTTACCGTCCGAGCTCAGGCTTACCTTACTCGAGCAATAAACAGTTGATAGGGCTTCTCCGTTACAGCCTGCGGTCCGGGTTCACTGC
ATCCTATCTAAGACCTTTGAAATGGCAGGCTCGAGTCCGAATGGAATGAGCTCGTTATTTGTCAACTATCCCGAAGAGGCAATGTCGGACGCCAGGCCCAAGTGACG

Linker_14 REs..._8 Linker_0 Prefix

CGTATAGGCAGTAATTTTGTTTAACTTTAAGAAGGAGATATACATATGGTTTCGGTTATCAAACCAGAGATGAAAATGCGTTACTATATGGATGGTTCAGTAAATGG
GCATATCCGTCATTAAAACAAATTGAAATTCTTCCTCTATATGTATACCAAAGCCAATAGTTTGGTCTCTACTTTTACGCAATGATATACCTACCAAGTCATTTACC

Cs...e RBS mKO2

TCACGAATTTACTATTGAGGGCGAGGGTACGGGACGCCCATACGAGGGGCACCAGGAAATGACTTTACGCGTCACAATGGCTGAAGGCGGGCCTATGCCGTTTGCGT
AGTGCTTAAATGATAACTCCCGCTCCCATGCCCTGCGGGTATGCTCCCCGTGGTCCTTTACTGAAATGCGCAGTGTTACCGACTTCCGCCCGGATACGGCAAACGCA

mKO2

TCGATCTTGTTAGTCATGTCTTTTGTTACGGTCACCGTGTATTTACTAAATACCCCGAGGAAATTCCAGACTATTTCAAACAAGCCTTCCCGGAAGGTTTGTCTTGG
AGCTAGAACAATCAGTACAGAAAACAATGCCAGTGGCACATAAATGATTTATGGGGCTCCTTTAAGGTCTGATAAAGTTTGTTCGGAAGGGCCTTCCAAACAGAACC

mKO2

GAGCGCAGTTTAGAGTTTGAAGACGGTGGCTCGGCCAGCGTGTCAGCTCATATTAGTCTTCGCGGCAATACATTTTATCACAAGTCAAAGTTCACCGGCGTGAACTT
CTCGCGTCAAATCTCAAACTTCTGCCACCGAGCCGGTCGCACAGTCGAGTATAATCAGAAGCGCCGTTATGTAAAATAGTGTTCAGTTTCAAGTGGCCGCACTTGAA

mKO2

CCCCGCAGACGGCCCAATCATGCAGAATCAAAGTGTTGATTGGGAACCGTCCACAGAGAAGATTACAGCTTCCGATGGAGTCTTAAAGGGCGATGTAACCATGTACT
GGGGCGTCTGCCGGGTTAGTACGTCTTAGTTTCACAACTAACCCTTGGCAGGTGTCTCTTCTAATGTCGAAGGCTACCTCAGAATTTCCCGCTACATTGGTACATGA

mKO2

TAAAATTAGAAGGGGGAGGGAACCATAAATGTCAGATGAAGACTACCTATAAGGCCGCAAAAGAGATTCTTGAAATGCCCGGAGACCACTACATTGGGCATCGTTTG
ATTTTAATCTTCCCCCTCCCTTGGTATTTACAGTCTACTTCTGATGGATATTCCGGCGTTTTCTCTAAGAACTTTACGGGCCTCTGGTGATGTAACCCGTAGCAAAC

mKO2

GTCCGTAAGACAGAAGGAAATATTACTGAACAGGTCGAAGACGCTGTGGCACACAGCATGTCCCGCCGTAATACTGACGCCATCACAATCCACAGCATCCTGGATTG
CAGGCATTCTGTCTTCCTTTATAATGACTTGTCCAGCTTCTGCGACACCGTGTGTCGTACAGGGCGGCATTATGACTGCGGTAGTGTTAGGTGTCGTAGGACCTAAC

mKO2 MarA

GATTGAAGACAACTTGGAGTCGCCGTTGAGTTTAGAAAAAGTTAGTGAACGTAGTGGTTACTCAAAGTGGCACCTTCAGCGCATGTTTAAGAAGGAAACGGGTCATT
CTAACTTCTGTTGAACCTCAGCGGCAACTCAAATCTTTTTCAATCACTTGCATCACCAATGAGTTTCACCGTGGAAGTCGCGTACAAATTCTTCCTTTGCCCAGTAA

MarA

pJ2043_N2only (7295 bp) (from 1285-2461 bp)

CATTGGGTCAATATATTCGTTCTCGCAAGATGACTGAAATTGCCCAGAAATTGAAAGAGTCTAATGAACCTATTTTGTACCTGGCGGAGCGTTACGGCTTTGAAAGT
GTAACCCAGTTATATAAGCAAGAGCGTTCTACTGACTTTAACGGGTCTTTAACTTTCTCAGATTACTTGGATAAAACATGGACCGCCTCGCAATGCCGAAACTTTCA
MarA

CAGCAAACCCTTACACGTACCTTCAAGAATTACTTTGACGTTCCACCACACAAATATCGTATGACCAACATGCAGGGTGAGTCACGTTTTTTGCATCCGTTGAATCA
GTCGTTTGGGAATGTGCATGGAAGTTCTTAATGAAACTGCAAGGTGGTGTGTTTATAGCATACTGGTTGTACGTCCCACTCAGTGCAAAAAACGTAGGCAACTTAGT
MarA

NcoI
TTACAATTCCTAATAATCGCTGGGACGCCCGCCATGGTTCAGCCAAAAAACTTAAGACCGCCGGTCTTGTCCACTACCTTGCAGTAATGCGGTGGACAGGATCGGCG
AATGTTAAGGATTATTAGCGACCCTGCGGGCGGTACCAAGTCGGTTTTTTGAATTCTGGCGGCCAGAACAGGTGATGGAACGTCATTACGCCACCTGTCCTAGCCGC
MarA | Suffix | | ECK120029600 Terminator

GTTTTCTTTTCTCTTCTCAATTCTTCTGACCTGTAACGAATAATAGATAGTAAAGTAGTCTCCGATTGAGTTTTCTCTGCCGAGTCCCACCCAGTTCTGTGATTTCA
CAAAAGAAAAGAGAAGAGTTAAGAAGACTGGACATTGCTTATTATCTATCATTTCATCAGAGGCTAACTCAAAAGAGACGGCTCAGGGTGGGTCAAGACACTAAAGT
ECK1200...inator | Spacer 1

GTAAGTTGGTAATTGATACACTGTTGCGAGAACTGCTGCCTGGTAGTAGATAGGTTGTTATTGAGTAAGAAGGTAAAGTGAACGAAATCCCTGAAACTGAGACTGTA
CATTCAACCATTAACTATGTGACAACGCTCTTGACGACGGACCATCATCTATCCAACAATAACTCATTCTTCCATTTCACTTGCTTTAGGGACTTTGACTCTGACAT
Spacer 1

HindIII
GAAAATAAGCTTCAGCCTGCGGTCCGGTTGACAGCTAGCTCAGTCCTAGGTACTGTGCTAGCTCGCTGGGACGCCCGGGGACTACACTTACGAAACTATTGATTGCT
CTTTTATTCGAAGTCGGACGCCAGGCCAACTGTCGATCGAGTCAGGATCCATGACACGATCGAGCGACCCTGCGGGCCCCTGATGTGAATGCTTTGATAACTAACGA
| | Prefix | P(BBa_J23102) | Suffix | Linker_1

BamHI
CAGCCTGCGGTCCGGccaAGTACTTAGTACACACTGATTCGCTGGGACGCCCGGGATCCAAGAGATTTCTACACGATTGAGCACTGTCTCAGCCTGCGGTCCGGGTT
GTCGGACGCCAGGCCggtTCATGAATCATGTGTGACTAAGCGACCCTGCGGGCCCTAGGTTCTCTAAAGATGTGCTAACTCGTGACAGAGTCGGACGCCAGGCCCAA
Prefix | bs-2 | Suffix | | Linker_10 | Prefix |

CACTGCCGTATAGGCAGTAATTTTGTTTAACTTTAAGAAGGAGATATACATATGCGTAAAGGCGAAGAACTGTTTACCGGTGTGGTTCCGATTCTGGTGGAACTGGA
GTGACGGCATATCCGTCATTAAAACAAATTGAAATTCTTCCTCTATATGTATACGCATTTCCGCTTCTTGACAAATGGCCACACCAAGGCTAAGACCACCTTGACCT
Csy4 site | RBS | sfGFP

CGGCGATGTTAATGGTCATAAATTCAGTGTTCGCGGCGAAGGTGAAGGCGATGCGACGAACGGCAAACTGACCCTGAAATTTATCTGCACCACGGGTAAACTGCCGG
GCCGCTACAATTACCAGTATTTAAGTCACAAGCGCCGCTTCCACTTCCGCTACGCTGCTTGCCGTTTGACTGGGACTTTAAATAGACGTGGTGCCCATTTGACGGCC
sfGFP

TCCCGTGGCCGACGCTGGTGACCACGCTGACCTATGGCGTTCAATGTTTTGCGCGTTACCCGGATCACATGAAACAGCACGACTTTTTCAAATCGGCCATGCCGGAA
AGGGCACCGGCTGCGACCACTGGTGCGACTGGATACCGCAAGTTACAAAACGCGCAATGGGCCTAGTGTACTTTGTCGTGCTGAAAAAGTTTAGCCGGTACGGCCTT
sfGFP

GGCTATGTGCAGGAACGTACGATTAGCTTTAAAGACGATGGTACGTATAAAACCCGCGCGGAAGTGAAATTCGAAGGCGATACCCTGGTTAACCGTATCGAACTGAA
CCGATACACGTCCTTGCATGCTAATCGAAATTTCTGCTACCATGCATATTTTGGGCGCGCCTTCACTTTAAGCTTCCGCTATGGGACCAATTGGCATAGCTTGACTT
sfGFP

pJ2043_N2only (7295 bp) (from 2462-3638 bp)

AGGTATCGATTTCAAAGAAGACGGCAATATTCTGGGTCATAAACTGGAATATAACTTCAATTCCCACAACGTGTACATCACCGCGGATAAACAGAAAAACGGCATTA
TCCATAGCTAAAGTTTCTTCTGCCGTTATAAGACCCAGTATTTGACCTTATATTGAAGTTAAGGGTGTTGCACATGTAGTGGCGCCTATTTGTCTTTTTGCCGTAAT
sfGFP

AAGCCAATTTCAAAATCCGCCATAATGTGGAAGATGGTAGCGTTCAGCTGGCCGACCACTATCAGCAAAACACGCCGATTGGTGATGGCCCGGTCCTGCTGCCGGAC
TTCGGTTAAAGTTTTAGGCGGTATTACACCTTCTACCATCGCAAGTCGACCGGCTGGTGATAGTCGTTTTGTGCGGCTAACCACTACCGGGCCAGGACGACGGCCTG
sfGFP

AATCACTACCTGAGTACCCAGTCCGTGCTGTCAAAAGATCCGAACGAAAAACGTGACCACATGGTCCTGCTGGAATTTGTGACGGCTGCGGGTATCACCCACGGCAT
TTAGTGATGGACTCATGGGTCAGGCACGACAGTTTTCTAGGCTTGCTTTTTGCACTGGTGTACCAGGACGACCTTAAACACTGCCGACGCCCATAGTGGGTGCCGTA
sfGFP

SbfI

GGACGAACTGTATAAAATGTCCCGCCGTAATACTGACGCCATCACAATCCACAGCATCCTGGATTGGATTGAAGACTAATAATCGCTGGGACGCCCGCCTGCAGGCT
CCTGCTTGACATATTTTACAGGGCGGCATTATGACTGCGGTAGTGTTAGGTGTCGTAGGACCTAACCTAACTTCTGATTATTAGCGACCCTGCGGGCGGACGTCCGA
sfGFP | MarAn20 | Suffix

KpnI

CGGTACCAAATTCCAGAAAAGAGGCCTCCCGAAAGGGGGGCCTTTTTTCGTTTTGGTCCTAATAGATAAAGGATAGGTCTGGTAGTGTTGTTCGTTCTCGCAGGTAA
GCCATGGTTTAAGGTCTTTTCTCCGGAGGGCTTTCCCCCCGGAAAAAAGCAAAACCAGGATTATCTATTTCCTATCCAGACCATCACAACAAGCAAGAGCGTCCATT
L3S2P21 Terminator | Spacer 2.5

ATCAATAATACTCAGCAGTTCCGTAGACTTTTCAGTGGGACAGGGTAGCGATAACAGATAGATTGTAATAAGACACAGTAGGTGCTCGTAGTTGCGTGAAGAGAACC
TAGTTATTATGAGTCGTCAAGGCATCTGAAAAGTCACCCTGTCCCATCGCTATTGTCTATCTAACATTATTCTGTGTCATCCACGAGCATCAACGCACTTCTCTTGG
Spacer 2.5

SalI | AatII

GCTCAGGAAATCCAGTCAGAAGTATTGGTAATCGTTGAAAACTCAGTCGACGCACTTACTGAAGACGTCCTATTACACTCGTCGTTGGAAACTGAAGATCAGCCTGC
CGAGTCCTTTAGGTCAGTCTTCATAACCATTAGCAACTTTTGAGTCAGCTGCGTGAATGACTTCTGCAGGATAATGTGAGCAGCAACCTTTGACTTCTAGTCGGACG
Spacer 2.5 | Spa... 5 | Linker_11

GGTCCGGGTTCACTGCCGTATAGGCAGTAATTTTGTTTAACTTTAAGAAGGAGATATACATATGAATCAGTCATTCATCTCGGACATCTTATATGCCGACATCGAAT
CCAGGCCCAAGTGACGGCATATCCGTCATTAAAACAAATTGAAATTCTTCCTCTATATGTATACTTAGTCAGTAAGTAGAGCCTGTAGAATATACGGCTGTAGCTTA
Csy4 site | RBS | RepA70

CGAAGGCTAAGGAACTTACAGTCAATTCCAACAATACTGTCCAGCCGGTCGCGCTTATGCGCTTAGGAGTTTTCGTTCCCAAACCTTCCAAGAGCAAAGGAGAAAGT
GCTTCCGATTCCTTGAATGTCAGTTAAGGTTGTTATGACAGGTCGGCCAGCGCGAATACGCGAATCCTCAAAAGCAAGGGTTTGGAAGGTTCTCGTTTCCTCTTTCA
RepA70

AAGGAAATTGACGCCACCAAAGCCTTCTCTCAACTGGAGATTGCTAAAGCAGAGGGCatggttagtaaaggagaagaaaataacatggcaCTGATTAAGGAGAACAT
TTCCTTTAACTGCGGTGGTTTCGGAAGAGAGTTGACCTCTAACGATTTCGTCTCCCGtaccaatcatttcctcttcttttattgtaccgtGACTAATTCCTCTTGTA
RepA70 | mKate2

GCACATGAAGCTGTACATGGAGGGCACCGTGAACAACCACCACTTCAAGTGCACATCCGAGGGCGAAGGCAAGCCCTACGAGGGCACCCAGACCATGAGAATCAAGg
CGTGTACTTCGACATGTACCTCCCGTGGCACTTGTTGGTGGTGAAGTTCACGTGTAGGCTCCCGCTTCCGTTCGGGATGCTCCCGTGGGTCTGGTACTCTTAGTTCc
mKate2

pJ2043_N2only (7295 bp) (from 3639-4922 bp)

ccGTCGAGGGCGGCCCTCTCCCCTTCGCCTTCGACATCCTGGCTACCAGCTTCATGTACGGCAGCAAAACCTTCATCAACCACACCCAGGGCATCCCCGACTTCTTT
ggCAGCTCCCGCCGGGAGAGGGGAAGCGGAAGCTGTAGGACCGATGGTCGAAGTACATGCCGTCGTTTTGGAAGTAGTTGGTGTGGGTCCCGTAGGGGCTGAAGAAA
mKate2

AAGCAGTCCTTCCCTGAGGGCTTCACATGGGAGAGAGTCACCACATACGAAGACGGGGGCGTGCTGACCGCTACCCAGGACACCAGCCTCCAGGACGGCTGCCTCAT
TTCGTCAGGAAGGGACTCCCGAAGTGTACCCTCTCTCAGTGGTGTATGCTTCTGCCCCCGCACGACTGGCGATGGGTCCTGTGGTCGGAGGTCCTGCCGACGGAGTA
mKate2

CTACAACGTCAAGATCAGAGGGGTGAACTTCCCATCCAACGGCCCTGTGATGCAGAAGAAAACACTCGGCTGGGAGGCCTCCACCGAGaccCTGTACCCCGCTGACG
GATGTTGCAGTTCTAGTCTCCCCACTTGAAGGGTAGGTTGCCGGGACACTACGTCTTCTTTTGTGAGCCGACCCTCCGGAGGTGGCTCtggGACATGGGGCGACTGC
mKate2

GCGGCCTGGAAGGCAGAgcCGACATGGCCCTGAAGCTCGTGGGCGGGGGCCACCTGATCTGCAACTTGAAGACCACATACAGATCCAAGAAACCCGCTAAGAACCTC
CGCCGGACCTTCCGTCTcgGCTGTACCGGGACTTCGAGCACCCGCCCCCGGTGGACTAGACGTTGAACTTCTGGTGTATGTCTAGGTTCTTTGGGCGATTCTTGGAG
mKate2

AAGATGCCCGGCGTCTACTATGTGGACAGAAGACTGGAAAGAATCAAGGAGGCCGACAAAGAGACCTACGTCGAGCAGCACGAGGTGGCTGTGGCCAGATACTGCGA
TTCTACGGGCCGCAGATGATACACCTGTCTTCTGACCTTTCTTAGTTCCTCCGGCTGTTTCTCTGGATGCAGCTCGTCGTGCTCCACCGACACCGGTCTATGACGCT
mKate2

NotI
CCTCCCTAGCAAACTGGGGCACAgAgtctaATAATCGCTGGGACGCCCGGCGGCCGCggaaacacagAAAAAAGCCCGCACCTGACAGTGCGGGCTTTTTTTTTcga
GGAGGGATCGTTTGACCCCGTGTcTcagatTATTAGCGACCCTGCGGGCCGCCGGCGcctttgtgtcTTTTTTCGGGCGTGGACTGTCACGCCCGAAAAAAAAAgct
mKate2 | Suffix | | ECK120033737 Terminator

KasI | BbvCI | AscI
ccaaaggTAGCGAACGACGAGTCACTGTTGAGGATAAATACTTTCTCTACTAGGCGCCTGTTACACAGGTCCTCAGCGGCGCGCCTTTGTCGGTGAACGCTCTCCTG
ggtttccATCGCTTGCTGCTCAGTGACAACTCCTATTTATGAAAGAGATGATCCGCGGACAATGTGTCCAGGAGTCGCCGCGCGGAAACAGCCACTTGCGAGAGGAC
Linker_8 | Spa...15

AGTAGGACAAATCCGCCGGGAGCGGATTTGAACGTTGTGAAGCAACGGCCCGGAGGGTGGCGGGCAGGACGCCCGCCATAAACTGCCAGGCATCAAACTAAGCAGAA
TCATCCTGTTTAGGCGGCCCTCGCCTAAACTTGCAACACTTCGTTGCCGGGCCTCCCACCGCCCGTCCTGCGGGCGGTATTTGACGGTCCGTAGTTTGATTCGTCTT

GGCCATCCTGACGGATGGCCTTTTTGCGTTTCAGATCTACCGGTaaaccagcaatagacataagcggctatttaacgaccctgccctgaaccgacgacaagctgacg
CCGGTAGGACTGCCTACCGGAAAAACGCAAAGTCTAGATGGCCAtttggtcgttatctgtattcgccgataaattgctgggacgggacttggctgctgttcgactgc

accgggtctccgcaagtggcacttttcggggaaatgtgcgcggaacccctatttgtttatttttctaaatacattcaaatatgtatccgctcatgaattaattctta
tggcccagaggcgttcaccgtgaaaagcccctttacacgcgccttggggataaacaaataaaaagatttatgtaagtttatacataggcgagtacttaattaagaat

gaaaaactcatcgagcatcaaatgaaactgcaatttattcatatcaggattatcaataccatatttttgaaaaagccgtttctgtaatgaaggagaaaactcaccga
ctttttgagtagctcgtagtttactttgacgttaaataagtatagtcctaatagttatggtataaaaactttttcggcaaagacattacttcctcttttgagtggct
KanR

ggcagttccataggatggcaagatcctggtatcggtctgcgattccgactcgtccaacatcaatacaacctattaatttcccctcgtcaaaaataaggttatcaagt
ccgtcaaggtatcctaccgttctaggaccatagccagacgctaaggctgagcaggttgtagttatgttggataattaaaggggagcagtttttattccaatagttca
KanR

pJ2043_N2only (7295 bp) (from 4923-6206 bp)

gagaaatcaccatgagtgacgactgaatccggtgagaatggcaaaagtttatgcatttctttccagacttgttcaacaggccagccattacgctcgtcatcaaaatc
ctctttagtggtactcactgctgacttaggccactcttaccgttttcaaatacgtaaagaaaggtctgaacaagttgtccggtcggtaatgcgagcagtagttttag
KanR

actcgcatcaaccaaaccgttattcattcgtgattgcgcctgagcgagacgaaatacgcggtcgctgttaaaaggacaattacaaacaggaatcgaatgcaaccggc
tgagcgtagttggtttggcaataagtaagcactaacgcggactcgctctgctttatgcgccagcgacaattttcctgttaatgtttgtccttagcttacgttggccg
KanR

gcaggaacactgccagcgcatcaacaatattttcacctgaatcaggatattcttctaatacctggaatgctgttttcccggggatcgcagtggtgagtaaccatgca
cgtccttgtgacggtcgcgtagttgttataaaagtggacttagtcctataagaagattatggaccttacgacaaaagggcccctagcgtcaccactcattggtacgt
KanR

tcatcaggagtacggataaaatgcttgatggtcggaagaggcataaattccgtcagccagtttagtctgaccatctcatctgtaacatcattggcaacgctaccttt
agtagtcctcatgcctattttacgaactaccagccttctccgtatttaaggcagtcggtcaaatcagactggtagagtagacattgtagtaaccgttgcgatggaaa
KanR

gccatgtttcagaaacaactctggcgcatcgggcttcccatacaatcgatagattgtcgcacctgattgcccgacattatcgcgagcccatttatacccatataaat
cggtacaaagtctttgttgagaccgcgtagcccgaagggtatgttagctatctaacagcgtggactaacgggctgtaatagcgctcgggtaaatatgggtatattta
KanR

cagcatccatgttggaatttaatcgcggcctagagcaagacgtttcccgttgaatatggctcatactcttcctttttcaatattattgaagcatttatcagggttat
gtcgtaggtacaaccttaaattagcgccggatctcgttctgcaaagggcaacttataccgagtatgagaaggaaaaagttataataacttcgtaaatagtcccaata
KanR

tgtctcatgagcggatacatatttgaatgtatttagaaaaataaacaaataggcatgctagcgcagaaacgtcctagaagatgccaggaggatacttagcagagaga
acagagtactcgcctatgtataaacttacataaatctttttatttgtttatccgtacgatcgcgtctttgcaggatcttctacggtcctcctatgaatcgtctctct
ColA ori

caataaggccggagcgaagccgtttttccataggctccgcccccctgacgaacatcacgaaatctgacgctcaaatcagtggtggcgaaacccgacaggactataaa
gttattccggcctcgcttcggcaaaaaggtatccgaggcggggggactgcttgtagtgctttagactgcgagtttagtcaccaccgctttgggctgtcctgatattt
ColA ori

gataccaggcgtttccccctgatggctccctcttgcgctctcctgttcccgtcctgcggcgtccgtgttgtggtggaggctttacccaaatcaccacgtcccgttcc
ctatggtccgcaaaggggggactaccgagggagaacgcgagaggacaagggcaggacgccgcaggcacaacaccacctccgaaatgggtttagtggtgcagggcaagg
ColA ori

gtgtagacagttcgctccaagctgggctgtgtgcaagaacccccc gttcagcccgactgctgcgccttatccggtaactatcatcttgagtccaacccggaaagaca
cacatctgtcaagcgaggttcgacccgacacacgttcttggggggcaagtcgggctgacgacgcggaataggccattgatagtagaactcaggttgggcctttctgt
ColA ori

cgacaaaacgccactggcagcagccattggtaactgagaattagtggatttagatatcgagagtcttgaagtggtggcctaacagaggctacactgaaaggacagta
gctgttttgcggtgaccgtcgtcggtaaccattgactcttaatcacctaaatctatagctctcagaacttcaccaccggattgtctccgatgtgactttcctgtcat
ColA ori

tttggtatctgcgctccactaaagccagttaccaggttaagcagttccccaactgacttaaccttcgatcaaaccgcctccccaggcggttttttcgtttacagagc
aaaccatagacgcgaggtgatttcggtcaatggtccaattcgtcaaggggttgactgaattggaagctagtttggcggaggggtccgccaaaaaagcaaatgtctcg
ColA ori

pJ2043_N2only (7295 bp) (from 6207-7295 bp)

aggagattacgacgatcgtaaaaggatctcaagaagatcctttacggattcccgacaccatcactctagatttcagtgcaatttatctcttcaaatgtagcacctga
tcctctaatgctgctagcattttcctagagttcttctaggaaatgcctaagggctgtggtagtgagatctaaagtcacgttaaatagagaagtttacatcgtggact
ColA ori

agtcagccccatacgatataagttgtaattctcatgttagtcatgccccgcgcccaccggaaggagctgactgggttgCTCCTAgGGTCTGATTCGTTACCAATTAT
tcagtcggggtatgctatattcaacattaagagtacaatcagtacggggcgcgggtggccttcctcgactgacccaacGAGGATcCCAGACTAAGCAATGGTTAATA

GACAACTTGACGGCTACATCATTCACTTTTTCTTCACAACCGGCACGGAACTCGCTCGGGCTGGCCCCGGTGCATTTTTTAAATACCCGCGAGAAATAGAGTTGATC
CTGTTGAACTGCCGATGTAGTAAGTGAAAAAGAAGTGTTGGCCGTGCCTTGAGCGAGCCCGACCGGGGCCACGTAAAAAATTTATGGGCGCTCTTTATCTCAACTAG
araC

GTCAAAACCAACATTGCGACCGACGGTGGCGATAGGCATCCGGGTGGTGCTCAAAAGCAGCTTCGCCTGGCTGATACGTTGGTCCTCGCGCCAGCTTAAGACGCTAA
CAGTTTTGGTTGTAACGCTGGCTGCCACCGCTATCCGTAGGCCCACCACGAGTTTTCGTCGAAGCGGACCGACTATGCAACCAGGAGCGCGGTCGAATTCTGCGATT
araC

TCCCTAACTGCTGGCGGAAAAGATGTGACAGACGCGACGGCGACAAGCAAACATGCTGTGCGACGCTGGCGATATCAAAATTGCTGTCTGCCAGGTGATCGCTGATG
AGGGATTGACGACCGCCTTTTCTACACTGTCTGCGCTGCCGCTGTTCGTTTGTACGACACGCTGCGACCGCTATAGTTTTAACGACAGACGGTCCACTAGCGACTAC
araC

TACTGACAAGCCTCGCGTACCCGATTATCCATCGGTGGATGGAGCGACTCGTTAATCGCTTCCATGCGCCGCAGTAACAATTGCTCAAGCAGATTTATCGCCAGCAG
ATGACTGTTCGGAGCGCATGGGCTAATAGGTAGCCACCTACCTCGCTGAGCAATTAGCGAAGGTACGCGGCGTCATTGTTAACGAGTTCGTCTAAATAGCGGTCGTC
araC

CTCCGAATAGCGCCCTTCCCCTTGCCCGGCGTTAATGATTTGCCCAAACAGGTCGCTGAAATGCGGCTGGTGCGCTTCATCCGGGCGAAAGAACCCCGTATTGGCAA
GAGGCTTATCGCGGGAAGGGGAACGGGCCGCAATTACTAAACGGGTTTGTCCAGCGACTTTACGCCGACCACGCGAAGTAGGCCCGCTTTCTTGGGGCATAACCGTT
araC

ATATTGACGGCCAGTTAAGCCATTCATGCCAGTAGGCGCGCGGACGAAAGTAAACCCACTGGTGATACCATTCGCGAGCCTCCGGATGACGACCGTAGTGATGAATC
TATAACTGCCGGTCAATTCGGTAAGTACGGTCATCCGCGCGCCTGCTTTCATTTGGGTGACCACTATGGTAAGCGCTCGGAGGCCTACTGCTGGCATCACTACTTAG
araC

TCTCCTGGCGGGAACAGCAAAATATCACCCGGTCGGCAAACAAATTCTCGTCCCTGATTTTTCACCACCCCCTGACCGCGAATGGTGAGATTGAGAATATAACCTTT
AGAGGACCGCCCTTGTCGTTTTATAGTGGGCCAGCCGTTTGTTTAAGAGCAGGGACTAAAAAGTGGTGGGGGACTGGCGCTTACCACTCTAACTCTTATATTGGAAA
araC

CATTCCCAGCGGTCGGTCGATAAAAAAATCGAGATAACCGTTGGCCTCAATCGGCGTTAAACCCGCCACCAGATGGGCATTAAACGAGTATCCCGGCAGCAGGGGAT
GTAAGGGTCGCCAGCCAGCTATTTTTTTAGCTCTATTGGCAACCGGAGTTAGCCGCAATTTGGGCGGTGGTCTACCCGTAATTTGCTCATAGGGCCGTCGTCCCCTA
araC

CATTTTGCGCTTCAGCCAT
GTAAAACGCGAAGTCGGTA
araC

(from 1-1177 bp)

pJ2044 (7459 bp)

ACTTTTCATACTCCCGCCATTCAGAGAAGAAACCAATTGTCCATATTGCATCAGACATTGCCGTCACTGCGTCTTTTACTGGCTCTTCTCGCTAACCAAACCGGTAA
TGAAAAGTATGAGGGCGGTAAGTCTCTTCTTTGGTTAACAGGTATAACGTAGTCTGTAACGGCAGTGACGCAGAAAATGACCGAGAAGAGCGATTGGTTTGGCCATT

CCCCGCTTATTAAAAGCATTCTGTAACAAAGCGGGACCAAAGCCATGACAAAAACGCGTAACAAAAGTGTCTATAATCACGGCAGAAAAGTCCACATTGATTATTTG
GGGGCGAATAATTTTCGTAAGACATTGTTTCGCCCTGGTTTCGGTACTGTTTTTGCGCATTGTTTTCACAGATATTAGTGCCGTCTTTTCAGGTGTAACTAATAAAC

EcoRI
CACGGCGTCACACTTTGCTATGCCATAGCATTTTTATCCATAAGATTAGCGGtTCCTACCTGACGCTTTTTATCGCAACTCTCTACTGTTTCTCCATACCGAATTCA
GTGCCGCAGTGTGAAACGATACGGTATCGTAAAAATAGGTATTCTAATCGCCaAGGATGGACTGCGAAAAATAGCGTTGAGAGATGACAAAGAGGTATGGCTTAAGT
P(BAD) promoter

SacI
TAGGATAGATTCTGGAAACTTTACCGTCCGAGCTCCAGCCTGCGGTCCGGGTTCACTGCCGTATAGGCAGTCTCAAGCTAGACTCTAGTGGTTTcAGAGCTATGCTG
ATCCTATCTAAGACCTTTGAAATGGCAGGCTCGAGGTCGGACGCCAGGCCCAAGTGACGGCATATCCGTCAGAGTTCGATCTGAGATCACCAAAgTCTCGATACGAC
Linker_14 Prefix Csy4 site sgRNA-1

XhoI
GAAACAGCATAGCAAGTTgAAATAAGGCTAGTCCGTTATCAACTTGAAAAAGTGGCACCGAGTCGGTGCGTTCACTGCCGTATAGGCAGTCGCTGGGACGCCCGCTC
CTTTGTCGTATCGTTCAAcTTTATTCCGATCAGGCAATAGTTGAACTTTTTCACCGTGGCTCAGCCACGCAAGTGACGGCATATCCGTCAGCGACCCTGCGGGCGAG
sgRNA-1 Csy4 site Suffix

GAGCAATAAACAGTTGATAGGGCTTCTCCGTTACAGCCTGCGGTCCGGGTTCACTGCCGTATAGGCAGTAATTTTGTTTAACTTTAAGAAGGAGATATACATATGGT
CTCGTTATTTGTCAACTATCCCGAAGAGGCAATGTCGGACGCCAGGCCCAAGTGACGGCATATCCGTCATTAAAACAAATTGAAATTCTTCCTCTATATGTATACCA
Linker_0 Prefix Csy4 site RBS

TTCGGTTATCAAACCAGAGATGAAAATGCGTTACTATATGGATGGTTCAGTAAATGGTCACGAATTTACTATTGAGGGCGAGGGTACGGGACGCCCATACGAGGGGC
AAGCCAATAGTTTGGTCTCTACTTTTACGCAATGATATACCTACCAAGTCATTTACCAGTGCTTAAATGATAACTCCCGCTCCCATGCCCTGCGGGTATGCTCCCCG
mKO2

ACCAGGAAATGACTTTACGCGTCACAATGGCTGAAGGCGGGCCTATGCCGTTTGCGTTCGATCTTGTTAGTCATGTCTTTTGTTACGGTCACCGTGTATTTACTAAA
TGGTCCTTTACTGAAATGCGCAGTGTTACCGACTTCCGCCCGGATACGGCAAACGCAAGCTAGAACAATCAGTACAGAAAACAATGCCAGTGGCACATAAATGATTT
mKO2

TACCCCGAGGAAATTCCAGACTATTTCAAACAAGCCTTCCCGGAAGGTTTGTCTTGGGAGCGCAGTTTAGAGTTTGAAGACGGTGGCTCGGCCAGCGTGTCAGCTCA
ATGGGGCTCCTTTAAGGTCTGATAAAGTTTGTTCGGAAGGGCCTTCCAAACAGAACCCTCGCGTCAAATCTCAAACTTCTGCCACCGAGCCGGTCGCACAGTCGAGT
mKO2

TATTAGTCTTCGCGGCAATACATTTTATCACAAGTCAAAGTTCACCGGCGTGAACTTCCCCGCAGACGGCCCAATCATGCAGAATCAAAGTGTTGATTGGGAACCGT
ATAATCAGAAGCGCCGTTATGTAAAATAGTGTTCAGTTTCAAGTGGCCGCACTTGAAGGGGCGTCTGCCGGGTTAGTACGTCTTAGTTTCACAACTAACCCTTGGCA
mKO2

CCACAGAGAAGATTACAGCTTCCGATGGAGTCTTAAAGGGCGATGTAACCATGTACTTAAAATTAGAAGGGGGAGGGAACCATAAATGTCAGATGAAGACTACCTAT
GGTGTCTCTTCTAATGTCGAAGGCTACCTCAGAATTTCCCGCTACATTGGTACATGAATTTTAATCTTCCCCCTCCCTTGGTATTTACAGTCTACTTCTGATGGATA
mKO2

pJ2044 (7459 bp) (from 1178-2354 bp)

AAGGCCGCAAAAGAGATTCTTGAAATGCCCGGAGACCACTACATTGGGCATCGTTTGGTCCGTAAGACAGAAGGAAATATTACTGAACAGGTCGAAGACGCTGTGGC
TTCCGGCGTTTTCTCTAAGAACTTTACGGGCCTCTGGTGATGTAACCCGTAGCAAACCAGGCATTCTGTCTTCCTTTATAATGACTTGTCCAGCTTCTGCGACACCG
mKO2

ACACAGCATGTCCCGCCGTAATACTGACGCCATCACAATCCACAGCATCCTGGATTGGATTGAAGACAACTTGGAGTCGCCGTTGAGTTTAGAAAAAGTTAGTGAAC
TGTGTCGTACAGGGCGGCATTATGACTGCGGTAGTGTTAGGTGTCGTAGGACCTAACCTAACTTCTGTTGAACCTCAGCGGCAACTCAAATCTTTTTCAATCACTTG
MarA

GTAGTGGTTACTCAAAGTGGCACCTTCAGCGCATGTTTAAGAAGGAAACGGGTCATTCATTGGGTCAATATATTCGTTCTCGCAAGATGACTGAAATTGCCCAGAAA
CATCACCAATGAGTTTCACCGTGGAAGTCGCGTACAAATTCTTCCTTTGCCCAGTAAGTAACCCAGTTATATAAGCAAGAGCGTTCTACTGACTTTAACGGGTCTTT
MarA

TTGAAAGAGTCTAATGAACCTATTTTGTACCTGGCGGAGCGTTACGGCTTTGAAAGTCAGCAAACCCTTACACGTACCTTCAAGAATTACTTTGACGTTCCACCACA
AACTTTCTCAGATTACTTGGATAAAACATGGACCGCCTCGCAATGCCGAAACTTTCAGTCGTTTGGGAATGTGCATGGAAGTTCTTAATGAAACTGCAAGGTGGTGT
MarA

NcoI
CAAATATCGTATGACCAACATGCAGGGTGAGTCACGTTTTTTGCATCCGTTGAATCATTACAATTCCTAATAATCGCTGGGACGCCCGCCATGGTTCAGCCAAAAAA
GTTTATAGCATACTGGTTGTACGTCCCACTCAGTGCAAAAAACGTAGGCAACTTAGTAATGTTAAGGATTATTAGCGACCCTGCGGGCGGTACCAAGTCGGTTTTTT
MarA | Suffix | ECK...or

CTTAAGACCGCCGGTCTTGTCCACTACCTTGCAGTAATGCGGTGGACAGGATCGGCGGTTTTCTTTTCTCTTCTCAATTCTTCTGACCTGTAACGAATAATAGATAG
GAATTCTGGCGGCCAGAACAGGTGATGGAACGTCATTACGCCACCTGTCCTAGCCGCCAAAAGAAAAGAGAAGAGTTAAGAAGACTGGACATTGCTTATTATCTATC
ECK120029600 Terminator | Spacer 1

TAAAGTAGTCTCCGATTGAGTTTTCTCTGCCGAGTCCCACCCAGTTCTGTGATTTCAGTAAGTTGGTAATTGATACACTGTTGCGAGAACTGCTGCCTGGTAGTAGA
ATTTCATCAGAGGCTAACTCAAAAGAGACGGCTCAGGGTGGGTCAAGACACTAAAGTCATTCAACCATTAACTATGTGACAACGCTCTTGACGACGGACCATCATCT
Spacer 1

HindIII
TAGGTTGTTATTGAGTAAGAAGGTAAAGTGAACGAAATCCCTGAAACTGAGACTGTAGAAAATAAGCTTCAGCCTGCGGTCCGGTTGACAGCTAGCTCAGTCCTAGG
ATCCAACAATAACTCATTCTTCCATTTCACTTGCTTTAGGGACTTTGACTCTGACATCTTTTATTCGAAGTCGGACGCCAGGCCAACTGTCGATCGAGTCAGGATCC
Spacer 1 | Prefix | P(BBa_J23102)

TACTGTGCTAGCTCGCTGGGACGCCCGGGGACTACACTTACGAAACTATTGATTGCTCAGCCTGCGGTCCGGccaCACTAGAGTCTAGCTTGAGATCGCTGGGACGC
ATGACACGATCGAGCGACCCTGCGGGCCCCTGATGTGAATGCTTTGATAACTAACGAGTCGGACGCCAGGCCggtGTGATCTCAGATCGAACTCTAGCGACCCTGCG
P(B...2) | Suffix | Linker_1 | Prefix | bs-1 | Suffix

BamHI
CCGGGATCCAAGAGATTTCTACACGATTGAGCACTGTCTCAGCCTGCGGTCCGGGTTCACTGCCGTATAGGCAGTAATTTTGTTTAACTTTAAGAAGGAGATATACA
GGCCCTAGGTTCTCTAAAGATGTGCTAACTCGTGACAGAGTCGGACGCCAGGCCCAAGTGACGGCATATCCGTCATTAAAACAAATTGAAATTCTTCCTCTATATGT
Linker_10 | Prefix | Csy4 site | RBS

TATGCGTAAAGGCGAAGAACTGTTTACCGGTGTGGTTCCGATTCTGGTGGAACTGGACGGCGATGTTAATGGTCATAAATTCAGTGTTCGCGGCGAAGGTGAAGGCG
ATACGCATTTCCGCTTCTTGACAAATGGCCACACCAAGGCTAAGACCACCTTGACCTGCCGCTACAATTACCAGTATTTAAGTCACAAGCGCCGCTTCCACTTCCGC
sfGFP

pJ2044 (7459 bp) (from 2355-3531 bp)

ATGCGACGAACGGCAAACTGACCCTGAAATTTATCTGCACCACGGGTAAACTGCCGGTCCCGTGGCCGACGCTGGTGACCACGCTGACCTATGGCGTTCAATGTTTT
TACGCTGCTTGCCGTTTGACTGGGACTTTAAATAGACGTGGTGCCCATTTGACGGCCAGGGCACCGGCTGCGACCACTGGTGCGACTGGATACCGCAAGTTACAAAA
sfGFP

GCGCGTTACCCGGATCACATGAAACAGCACGACTTTTTCAAATCGGCCATGCCGGAAGGCTATGTGCAGGAACGTACGATTAGCTTTAAAGACGATGGTACGTATAA
CGCGCAATGGGCCTAGTGTACTTTGTCGTGCTGAAAAAGTTTAGCCGGTACGGCCTTCCGATACACGTCCTTGCATGCTAATCGAAATTTCTGCTACCATGCATATT
sfGFP

AACCCGCGCGGAAGTGAAATTCGAAGGCGATACCCTGGTTAACCGTATCGAACTGAAAGGTATCGATTTCAAAGAAGACGGCAATATTCTGGGTCATAAACTGGAAT
TTGGGCGCGCCTTCACTTTAAGCTTCCGCTATGGGACCAATTGGCATAGCTTGACTTTCCATAGCTAAAGTTTCTTCTGCCGTTATAAGACCCAGTATTTGACCTTA
sfGFP

ATAACTTCAATTCCCACAACGTGTACATCACCGCGGATAAACAGAAAAACGGCATTAAAGCCAATTTCAAAATCCGCCATAATGTGGAAGATGGTAGCGTTCAGCTG
TATTGAAGTTAAGGGTGTTGCACATGTAGTGGCGCCTATTTGTCTTTTTGCCGTAATTTCGGTTAAAGTTTTAGGCGGTATTACACCTTCTACCATCGCAAGTCGAC
sfGFP

GCCGACCACTATCAGCAAAACACGCCGATTGGTGATGGCCCGGTCCTGCTGCCGGACAATCACTACCTGAGTACCCAGTCCGTGCTGTCAAAAGATCCGAACGAAAA
CGGCTGGTGATAGTCGTTTTGTGCGGCTAACCACTACCGGGCCAGGACGACGGCCTGTTAGTGATGGACTCATGGGTCAGGCACGACAGTTTTCTAGGCTTGCTTTT
sfGFP

ACGTGACCACATGGTCCTGCTGGAATTTGTGACGGCTGCGGGTATCACCCACGGCATGGACGAACTGTATAAAATGTCCCGCCGTAATACTGACGCCATCACAATCC
TGCACTGGTGTACCAGGACGACCTTAAACACTGCCGACGCCCATAGTGGGTGCCGTACCTGCTTGACATATTTTACAGGGCGGCATTATGACTGCGGTAGTGTTAGG
sfGFP | MarAn20

SbfI KpnI
ACAGCATCCTGGATTGGATTGAAGACTAATAATCGCTGGGACGCCCGCCTGCAGGCTCGGTACCAAATTCCAGAAAAGAGGCCTCCCGAAAGGGGGGCCTTTTTTCG
TGTCGTAGGACCTAACCTAACTTCTGATTATTAGCGACCCTGCGGGCGGACGTCCGAGCCATGGTTTAAGGTCTTTTCTCCGGAGGGCTTTCCCCCCGGAAAAAAGC
MarAn20 | Suffix | | L3S2P21 Terminator

TTTTGGTCCTAATAGATAAAGGATAGGTCTGGTAGTGTTGTTCGTTCTCGCAGGTAAATCAATAATACTCAGCAGTTCCGTAGACTTTTCAGTGGGACAGGGTAGCG
AAAACCAGGATTATCTATTTCCTATCCAGACCATCACAACAAGCAAGAGCGTCCATTTAGTTATTATGAGTCGTCAAGGCATCTGAAAAGTCACCCTGTCCCATCGC
| Spacer 2.5

SalI
ATAACAGATAGATTGTAATAAGACACAGTAGGTGCTCGTAGTTGCGTGAAGAGAACCGCTCAGGAAATCCAGTCAGAAGTATTGGTAATCGTTGAAAACTCAGTCGA
TATTGTCTATCTAACATTATTCTGTGTCATCCACGAGCATCAACGCACTTCTCTTGGCGAGTCCTTTAGGTCAGTCTTCATAACCATTAGCAACTTTTGAGTCAGCT
Spacer 2.5 |

AatII
CGCACTTACTGAAGACGTCCTATTACACTCGTCGTTGGAAACTGAAGATCAGCCTGCGGTCCGGGTTCACTGCCGTATAGGCAGTAATTTTGTTTAACTTTAAGAAG
GCGTGAATGACTTCTGCAGGATAATGTGAGCAGCAACCTTTGACTTCTAGTCGGACGCCAGGCCCAAGTGACGGCATATCCGTCATTAAAACAAATTGAAATTCTTC
| Spa... 5 | | Linker_11 | Prefix | Csy4 site | RBS

GAGATATACATATGAATCAGTCATTCATCTCGGACATCTTATATGCCGACATCGAATCGAAGGCTAAGGAACTTACAGTCAATTCCAACAATACTGTCCAGCCGGTC
CTCTATATGTATACTTAGTCAGTAAGTAGAGCCTGTAGAATATACGGCTGTAGCTTAGCTTCCGATTCCTTGAATGTCAGTTAAGGTTGTTATGACAGGTCGGCCAG
RBS | RepA70

pJ2044 (7459 bp) (from 3532-4815 bp)

GCGCTTATGCGCTTAGGAGTTTTCGTTCCCAAACCTTCCAAGAGCAAAGGAGAAAGTAAGGAAATTGACGCCACCAAAGCCTTCTCTCAACTGGAGATTGCTAAAGC
CGCGAATACGCGAATCCTCAAAAGCAAGGGTTTGGAAGGTTCTCGTTTCCTCTTTCATTCCTTTAACTGCGGTGGTTTCGGAAGAGAGTTGACCTCTAACGATTTCG
RepA70

AGAGGGCatggttagtaaaggagaagaaaataacatggcaCTGATTAAGGAGAACATGCACATGAAGCTGTACATGGAGGGCACCGTGAACAACCACCACTTCAAGT
TCTCCCGtaccaatcatttcctcttcttttattgtaccgtGACTAATTCCTCTTGTACGTGTACTTCGACATGTACCTCCCGTGGCACTTGTTGGTGGTGAAGTTCA
mKate2

GCACATCCGAGGGCGAAGGCAAGCCCTACGAGGGCACCCAGACCATGAGAATCAAGgccGTCGAGGGCGGCCCTCTCCCCTTCGCCTTCGACATCCTGGCTACCAGC
CGTGTAGGCTCCCGCTTCCGTTCGGGATGCTCCCGTGGGTCTGGTACTCTTAGTTCcggCAGCTCCCGCCGGGAGAGGGGAAGCGGAAGCTGTAGGACCGATGGTCG
mKate2

TTCATGTACGGCAGCAAAACCTTCATCAACCACACCCAGGGCATCCCCGACTTCTTTAAGCAGTCCTTCCCTGAGGGCTTCACATGGGAGAGAGTCACCACATACGA
AAGTACATGCCGTCGTTTTGGAAGTAGTTGGTGTGGGTCCCGTAGGGGCTGAAGAAATTCGTCAGGAAGGGACTCCCGAAGTGTACCCTCTCTCAGTGGTGTATGCT
mKate2

AGACGGGGGCGTGCTGACCGCTACCCAGGACACCAGCCTCCAGGACGGCTGCCTCATCTACAACGTCAAGATCAGAGGGGTGAACTTCCCATCCAACGGCCCTGTGA
TCTGCCCCCGCACGACTGGCGATGGGTCCTGTGGTCGGAGGTCCTGCCGACGGAGTAGATGTTGCAGTTCTAGTCTCCCCACTTGAAGGGTAGGTTGCCGGGACACT
mKate2

TGCAGAAGAAAACACTCGGCTGGGAGGCCTCCACCGAGaccCTGTACCCCGCTGACGGCGGCCTGGAAGGCAGAgcCGACATGGCCCTGAAGCTCGTGGGCGGGGGC
ACGTCTTCTTTTGTGAGCCGACCCTCCGGAGGTGGCTCtggGACATGGGGCGACTGCCGCCGGACCTTCCGTCTcgGCTGTACCGGGACTTCGAGCACCCGCCCCCG
mKate2

CACCTGATCTGCAACTTGAAGACCACATACAGATCCAAGAAACCCGCTAAGAACCTCAAGATGCCCGGCGTCTACTATGTGGACAGAAGACTGGAAAGAATCAAGGA
GTGGACTAGACGTTGAACTTCTGGTGTATGTCTAGGTTCTTTGGGCGATTCTTGGAGTTCTACGGGCCGCAGATGATACACCTGTCTTCTGACCTTTCTTAGTTCCT
mKate2

GGCCGACAAAGAGACCTACGTCGAGCAGCACGAGGTGGCTGTGGCCAGATACTGCGACCTCCCTAGCAAACTGGGGCACAgAgtctaATAATCGCTGGGACGCCCGG
CCGGCTGTTTCTCTGGATGCAGCTCGTCGTGCTCCACCGACACCGGTCTATGACGCTGGAGGGATCGTTTGACCCCGTGTcTcagatTATTAGCGACCCTGCGGGCC
mKate2 Suffix

NotI

CGGCCGCggaaacacagAAAAAAGCCCGCACCTGACAGTGCGGGCTTTTTTTTTcgaccaaaggTAGCGAACGACGAGTCACTGTTGAGGATAAATACTTTCTCTAC
GCCGGCGcctttgtgtcTTTTTTCGGGCGTGGACTGTCACGCCCGAAAAAAAAAgctggtttccATCGCTTGCTGCTCAGTGACAACTCCTATTTATGAAAGAGATG
ECK120033737 Terminator Linker_8

KasI BbvCI AscI

TAGGCGCCTGTTACACAGGTCCTCAGCGGCGCGCCTTTGTCGGTGAACGCTCTCCTGAGTAGGACAAATCCGCCGGGAGCGGATTTGAACGTTGTGAAGCAACGGCC
ATCCGCGGACAATGTGTCCAGGAGTCGCCGCGCGGAAACAGCCACTTGCGAGAGGACTCATCCTGTTTAGGCGGCCCTCGCCTAAACTTGCAACACTTCGTTGCCGG
Spa...15

CGGAGGGTGGCGGGCAGGACGCCCGCCATAAACTGCCAGGCATCAAACTAAGCAGAAGGCCATCCTGACGGATGGCCTTTTTGCGTTTCAGATCTACCGGTaaacca
GCCTCCCACCGCCCGTCCTGCGGGCGGTATTTGACGGTCCGTAGTTTGATTCGTCTTCCGGTAGGACTGCCTACCGGAAAAACGCAAAGTCTAGATGGCCAtttggt

gcaatagacataagcggctatttaacgaccctgccctgaaccgacgacaagctgacgaccgggtctccgcaagtggcacttttcggggaaatgtgcgcggaacccct
cgttatctgtattcgccgataaattgctgggacgggacttggctgctgttcgactgctggcccagaggcgttcaccgtgaaaagcccctttacacgcgccttgggga

pJ2044 (7459 bp) (from 4816-6099 bp)

atttgtttatttttctaaatacattcaaatatgtatccgctcatgaattaattcttagaaaaactcatcgagcatcaaatgaaactgcaatttattcatatcaggat
taaacaaataaaaagatttatgtaagtttatacataggcgagtacttaattaagaatctttttgagtagctcgtagtttactttgacgttaaataagtatagtccta
KanR

tatcaataccatatttttgaaaaagccgtttctgtaatgaaggagaaaactcaccgaggcagttccataggatggcaagatcctggtatcggtctgcgattccgact
atagttatggtataaaaactttttcggcaaagacattacttcctcttttgagtggctccgtcaaggtatcctaccgttctaggaccatagccagacgctaaggctga
KanR

cgtccaacatcaatacaacctattaatttcccctcgtcaaaaataaggttatcaagtgagaaatcaccatgagtgacgactgaatccggtgagaatggcaaaagttt
gcaggttgtagttatgttggataattaaaggggagcagtttttattccaatagttcactctttagtggtactcactgctgacttaggccactcttaccgttttcaaa
KanR

atgcatttctttccagacttgttcaacaggccagccattacgctcgtcatcaaaatcactcgcatcaaccaaaccgttattcattcgtgattgcgcctgagcgagac
tacgtaaagaaaggtctgaacaagttgtccggtcggtaatgcgagcagtagttttagtgagcgtagttggtttggcaataagtaagcactaacgcggactcgctctg
KanR

gaaatacgcggtcgctgttaaaaggacaattacaaacaggaatcgaatgcaaccggcgcaggaacactgccagcgcatcaacaatattttcacctgaatcaggatat
ctttatgcgccagcgacaattttcctgttaatgtttgtccttagcttacgttggccgcgtccttgtgacggtcgcgtagttgttataaaagtggacttagtcctata
KanR

tcttctaatacctggaatgctgttttcccggggatcgcagtggtgagtaaccatgcatcatcaggagtacggataaaatgcttgatggtcggaagaggcataaattc
agaagattatggaccttacgacaaaagggcccctagcgtcaccactcattggtacgtagtagtcctcatgcctattttacgaactaccagccttctccgtatttaag
KanR

cgtcagccagtttagtctgaccatctcatctgtaacatcattggcaacgctacctttgccatgtttcagaaacaactctggcgcatcgggcttcccatacaatcgat
gcagtcggtcaaatcagactggtagagtagacattgtagtaaccgttgcgatggaaacggtacaaagtctttgttgagaccgcgtagcccgaagggtatgttagcta
KanR

agattgtcgcacctgattgcccgacattatcgcgagcccatttatacccatataaatcagcatccatgttggaatttaatcgcggcctagagcaagacgtttcccgt
tctaacagcgtggactaacgggctgtaatagcgctcgggtaaatatgggtatatttagtcgtaggtacaaccttaaattagcgccggatctcgttctgcaaagggca
KanR

tgaatatggctcatactcttcctttttcaatattattgaagcatttatcagggttattgtctcatgagcggatacatatttgaatgtatttagaaaaataaacaaat
acttataccgagtatgagaaggaaaaagttataataacttcgtaaatagtcccaataacagagtactcgcctatgtataaacttacataaatctttttatttgttta
KanR

aggcatgctagcgcagaaacgtcctagaagatgccaggaggatacttagcagagagacaataaggccggagcgaagccgtttttccataggctccgcccccctgacg
tccgtacgatcgcgtctttgcaggatcttctacggtcctcctatgaatcgtctctctgttattccggcctcgcttcggcaaaaaggtatccgaggcggggggactgc
ColA ori

aacatcacgaaatctgacgctcaaatcagtggtggcgaaacccgacaggactataaagataccaggcgtttccccctgatggctccctcttgcgctctcctgttccc
ttgtagtgctttagactgcgagtttagtcaccaccgctttgggctgtcctgatatttctatggtccgcaaagggggactaccgagggagaacgcgagaggacaaggg
ColA ori

gtcctgcggcgtccgtgttgtggtggaggctttacccaaatcaccacgtcccgttccgtgtagacagttcgctccaagctgggctgtgtgcaagaaccccccgttca
caggacgccgcaggcacaacaccacctccgaaatgggtttagtggtgcagggcaaggcacatctgtcaagcgaggttcgacccgacacacgttcttggggggcaagt
ColA ori

gcccgactgctgcgccttatccggtaactatcatcttgagtccaacccggaaagacacgacaaaacgccactggcagcagccattggtaactgagaattagtggatt
cgggctgacgacgcggaataggccattgatagtagaactcaggttgggcctttctgtgctgttttgcggtgaccgtcgtcggtaaccattgactcttaatcacctaa
ColA ori

tagatatcgagagtcttgaagtggtggcctaacagaggctacactgaaaggacagtatttggtatctgcgctccactaaagccagttaccaggttaagcagttcccc
atctatagctctcagaacttcaccaccggattgtctccgatgtgactttcctgtcataaaccatagacgcgaggtgatttcggtcaatggtccaattcgtcaagggg
ColA ori

aactgacttaaccttcgatcaaaccgcctccccaggcggttttttcgtttacagagcaggagattacgacgatcgtaaaaggatctcaagaagatcctttacggatt
ttgactgaattggaagctagtttggcggaggggtccgccaaaaaagcaaatgtctcgtcctctaatgctgctagcattttcctagagttcttctaggaaatgcctaa
ColA ori

cccgacaccatcactctagatttcagtgcaatttatctcttcaaatgtagcacctgaagtcagccccatacgatataagttgtaattctcatgttagtcatgccccg
gggctgtggtagtgagatctaaagtcacgttaaatagagaagtttacatcgtggacttcagtcggggtatgctatattcaacattaagagtacaatcagtacggggc
Co...i

cgcccaccggaaggagctgactgggttgCTCCTAgGGTCTGATTCGTTACCAATTATGACAACTTGACGGCTACATCATTCACTTTTTCTTCACAACCGGCACGGAA
gcgggtggccttcctcgactgacccaacGAGGATcCCAGACTAAGCAATGGTTAATACTGTTGAACTGCCGATGTAGTAAGTGAAAAAGAAGTGTTGGCCGTGCCTT
araC

CTCGCTCGGGCTGGCCCCGGTGCATTTTTTAAATACCCGCGAGAAATAGAGTTGATCGTCAAAACCAACATTGCGACCGACGGTGGCGATAGGCATCCGGGTGGTGC
GAGCGAGCCCGACCGGGGCCACGTAAAAAATTTATGGGCGCTCTTTATCTCAACTAGCAGTTTTGGTTGTAACGCTGGCTGCCACCGCTATCCGTAGGCCCACCACG
araC

TCAAAAGCAGCTTCGCCTGGCTGATACGTTGGTCCTCGCGCCAGCTTAAGACGCTAATCCCTAACTGCTGGCGGAAAAGATGTGACAGACGCGACGGCGACAAGCAA
AGTTTTCGTCGAAGCGGACCGACTATGCAACCAGGAGCGCGGTCGAATTCTGCGATTAGGGATTGACGACCGCCTTTTCTACACTGTCTGCGCTGCCGCTGTTCGTT
araC

ACATGCTGTGCGACGCTGGCGATATCAAAATTGCTGTCTGCCAGGTGATCGCTGATGTACTGACAAGCCTCGCGTACCCGATTATCCATCGGTGGATGGAGCGACTC
TGTACGACACGCTGCGACCGCTATAGTTTTAACGACAGACGGTCCACTAGCGACTACATGACTGTTCGGAGCGCATGGGCTAATAGGTAGCCACCTACCTCGCTGAG
araC

GTTAATCGCTTCCATGCGCCGCAGTAACAATTGCTCAAGCAGATTTATCGCCAGCAGCTCCGAATAGCGCCCTTCCCCTTGCCCGGCGTTAATGATTTGCCCAAACA
CAATTAGCGAAGGTACGCGGCGTCATTGTTAACGAGTTCGTCTAAATAGCGGTCGTCGAGGCTTATCGCGGGAAGGGGAACGGGCCGCAATTACTAAACGGGTTTGT
araC

GGTCGCTGAAATGCGGCTGGTGCGCTTCATCCGGGCGAAAGAACCCCGTATTGGCAAATATTGACGGCCAGTTAAGCCATTCATGCCAGTAGGCGCGCGGACGAAAG
CCAGCGACTTTACGCCGACCACGCGAAGTAGGCCCGCTTTCTTGGGGCATAACCGTTTATAACTGCCGGTCAATTCGGTAAGTACGGTCATCCGCGCGCCTGCTTTC
araC

TAAACCCACTGGTGATACCATTCGCGAGCCTCCGGATGACGACCGTAGTGATGAATCTCTCCTGGCGGGAACAGCAAAATATCACCCGGTCGGCAAACAAATTCTCG
ATTTGGGTGACCACTATGGTAAGCGCTCGGAGGCCTACTGCTGGCATCACTACTTAGAGAGGACCGCCCTTGTCGTTTTATAGTGGGCCAGCCGTTTGTTTAAGAGC
araC

TCCCTGATTTTTCACCACCCCCTGACCGCGAATGGTGAGATTGAGAATATAACCTTTCATTCCCAGCGGTCGGTCGATAAAAAAATCGAGATAACCGTTGGCCTCAA
AGGGACTAAAAAGTGGTGGGGGACTGGCGCTTACCACTCTAACTCTTATATTGGAAAGTAAGGGTCGCCAGCCAGCTATTTTTTTAGCTCTATTGGCAACCGGAGTT
araC

```
TCGGCGTTAAACCCGCCACCAGATGGGCATTAAACGAGTATCCCGGCAGCAGGGGATCATTTTGCGCTTCAGCCAT
AGCCGCAATTTGGGCGGTGGTCTACCCGTAATTTGCTCATAGGGCCGTCGTCCCCTAGTAAAACGCGAAGTCGGTA
```

araC

(from 1-1284 bp)

pJ2044_N2only (7295 bp)

ACTTTTCATACTCCCGCCATTCAGAGAAGAAACCAATTGTCCATATTGCATCAGACATTGCCGTCACTGCGTCTTTTACTGGCTCTTCTCGCTAACCAAACCGGTAA
TGAAAAGTATGAGGGCGGTAAGTCTCTTCTTTGGTTAACAGGTATAACGTAGTCTGTAACGGCAGTGACGCAGAAAATGACCGAGAAGAGCGATTGGTTTGGCCATT

CCCCGCTTATTAAAAGCATTCTGTAACAAAGCGGGACCAAAGCCATGACAAAAACGCGTAACAAAAGTGTCTATAATCACGGCAGAAAAGTCCACATTGATTATTTG
GGGGCGAATAATTTTCGTAAGACATTGTTTCGCCCTGGTTTCGGTACTGTTTTTGCGCATTGTTTTCACAGATATTAGTGCCGTCTTTTCAGGTGTAACTAATAAAC

EcoRI

CACGGCGTCACACTTTGCTATGCCATAGCATTTTTATCCATAAGATTAGCGGtTCCTACCTGACGCTTTTTATCGCAACTCTCTACTGTTTCTCCATACCGAATTCA
GTGCCGCAGTGTGAAACGATACGGTATCGTAAAAATAGGTATTCTAATCGCCaAGGATGGACTGCGAAAAATAGCGTTGAGAGATGACAAAGAGGTATGGCTTAAGT

P(BAD) promoter

SacI XhoI

TAGGATAGATTCTGGAAACTTTACCGTCCGAGCTCAGGCTTACCTTACTCGAGCAATAAACAGTTGATAGGGCTTCTCCGTTACAGCCTGCGGTCCGGGTTCACTGC
ATCCTATCTAAGACCTTTGAAATGGCAGGCTCGAGTCCGAATGGAATGAGCTCGTTATTTGTCAACTATCCCGAAGAGGCAATGTCGGACGCCAGGCCCAAGTGACG

Linker_14 REs..._8 Linker_0 Prefix

CGTATAGGCAGTAATTTTGTTTAACTTTAAGAAGGAGATATACATATGGTTTCGGTTATCAAACCAGAGATGAAAATGCGTTACTATATGGATGGTTCAGTAAATGG
GCATATCCGTCATTAAAACAAATTGAAATTCTTCCTCTATATGTATACCAAAGCCAATAGTTTGGTCTCTACTTTTACGCAATGATATACCTACCAAGTCATTTACC

Cs...e RBS mKO2

TCACGAATTTACTATTGAGGGCGAGGGTACGGGACGCCCATACGAGGGGCACCAGGAAATGACTTTACGCGTCACAATGGCTGAAGGCGGGCCTATGCCGTTTGCGT
AGTGCTTAAATGATAACTCCCGCTCCCATGCCCTGCGGGTATGCTCCCCGTGGTCCTTTACTGAAATGCGCAGTGTTACCGACTTCCGCCCGGATACGGCAAACGCA

mKO2

TCGATCTTGTTAGTCATGTCTTTTGTTACGGTCACCGTGTATTTACTAAATACCCCGAGGAAATTCCAGACTATTTCAAACAAGCCTTCCCGGAAGGTTTGTCTTGG
AGCTAGAACAATCAGTACAGAAAACAATGCCAGTGGCACATAAATGATTTATGGGGCTCCTTTAAGGTCTGATAAAGTTTGTTCGGAAGGGCCTTCCAAACAGAACC

mKO2

GAGCGCAGTTTAGAGTTTGAAGACGGTGGCTCGGCCAGCGTGTCAGCTCATATTAGTCTTCGCGGCAATACATTTTATCACAAGTCAAAGTTCACCGGCGTGAACTT
CTCGCGTCAAATCTCAAACTTCTGCCACCGAGCCGGTCGCACAGTCGAGTATAATCAGAAGCGCCGTTATGTAAAATAGTGTTCAGTTTCAAGTGGCCGCACTTGAA

mKO2

CCCCGCAGACGGCCCAATCATGCAGAATCAAAGTGTTGATTGGGAACCGTCCACAGAGAAGATTACAGCTTCCGATGGAGTCTTAAAGGGCGATGTAACCATGTACT
GGGGCGTCTGCCGGGTTAGTACGTCTTAGTTTCACAACTAACCCTTGGCAGGTGTCTCTTCTAATGTCGAAGGCTACCTCAGAATTTCCCGCTACATTGGTACATGA

mKO2

TAAAATTAGAAGGGGGAGGGAACCATAAATGTCAGATGAAGACTACCTATAAGGCCGCAAAAGAGATTCTTGAAATGCCCGGAGACCACTACATTGGGCATCGTTTG
ATTTTAATCTTCCCCCTCCCTTGGTATTTACAGTCTACTTCTGATGGATATTCCGGCGTTTTCTCTAAGAACTTTACGGGCCTCTGGTGATGTAACCCGTAGCAAAC

mKO2

GTCCGTAAGACAGAAGGAAATATTACTGAACAGGTCGAAGACGCTGTGGCACACAGCATGTCCCGCCGTAATACTGACGCCATCACAATCCACAGCATCCTGGATTG
CAGGCATTCTGTCTTCCTTTATAATGACTTGTCCAGCTTCTGCGACACCGTGTGTCGTACAGGGCGGCATTATGACTGCGGTAGTGTTAGGTGTCGTAGGACCTAAC

mKO2 MarA

GATTGAAGACAACTTGGAGTCGCCGTTGAGTTTAGAAAAAGTTAGTGAACGTAGTGGTTACTCAAAGTGGCACCTTCAGCGCATGTTTAAGAAGGAAACGGGTCATT
CTAACTTCTGTTGAACCTCAGCGGCAACTCAAATCTTTTTCAATCACTTGCATCACCAATGAGTTTCACCGTGGAAGTCGCGTACAAATTCTTCCTTTGCCCAGTAA

MarA

pJ2044_N2only (7295 bp) (from 1285-2461 bp)

CATTGGGTCAATATATTCGTTCTCGCAAGATGACTGAAATTGCCCAGAAATTGAAAGAGTCTAATGAACCTATTTTGTACCTGGCGGAGCGTTACGGCTTTGAAAGT
GTAACCCAGTTATATAAGCAAGAGCGTTCTACTGACTTTAACGGGTCTTTAACTTTCTCAGATTACTTGGATAAAACATGGACCGCCTCGCAATGCCGAAACTTTCA
MarA

CAGCAAACCCTTACACGTACCTTCAAGAATTACTTTGACGTTCCACCACACAAATATCGTATGACCAACATGCAGGGTGAGTCACGTTTTTTGCATCCGTTGAATCA
GTCGTTTGGGAATGTGCATGGAAGTTCTTAATGAAACTGCAAGGTGGTGTGTTTATAGCATACTGGTTGTACGTCCCACTCAGTGCAAAAAACGTAGGCAACTTAGT
MarA

NcoI
TTACAATTCCTAATAATCGCTGGGACGCCCGCCATGGTTCAGCCAAAAAACTTAAGACCGCCGGTCTTGTCCACTACCTTGCAGTAATGCGGTGGACAGGATCGGCG
AATGTTAAGGATTATTAGCGACCCTGCGGGCGGTACCAAGTCGGTTTTTTGAATTCTGGCGGCCAGAACAGGTGATGGAACGTCATTACGCCACCTGTCCTAGCCGC
MarA | Suffix | | ECK120029600 Terminator

GTTTTCTTTTCTCTTCTCAATTCTTCTGACCTGTAACGAATAATAGATAGTAAAGTAGTCTCCGATTGAGTTTTCTCTGCCGAGTCCCACCCAGTTCTGTGATTTCA
CAAAAGAAAAGAGAAGAGTTAAGAAGACTGGACATTGCTTATTATCTATCATTTCATCAGAGGCTAACTCAAAAGAGACGGCTCAGGGTGGGTCAAGACACTAAAGT
ECK1200...inator | Spacer 1

GTAAGTTGGTAATTGATACACTGTTGCGAGAACTGCTGCCTGGTAGTAGATAGGTTGTTATTGAGTAAGAAGGTAAAGTGAACGAAATCCCTGAAACTGAGACTGTA
CATTCAACCATTAACTATGTGACAACGCTCTTGACGACGGACCATCATCTATCCAACAATAACTCATTCTTCCATTTCACTTGCTTTAGGGACTTTGACTCTGACAT
Spacer 1

HindIII
GAAAATAAGCTTCAGCCTGCGGTCCGGTTGACAGCTAGCTCAGTCCTAGGTACTGTGCTAGCTCGCTGGGACGCCCGGGGACTACACTTACGAAACTATTGATTGCT
CTTTTATTCGAAGTCGGACGCCAGGCCAACTGTCGATCGAGTCAGGATCCATGACACGATCGAGCGACCCTGCGGGCCCCTGATGTGAATGCTTTGATAACTAACGA
| | Prefix | P(BBa_J23102) | Suffix | Linker_1

BamHI
CAGCCTGCGGTCCGGccaCACTAGAGTCTAGCTTGAGATCGCTGGGACGCCCGGGATCCAAGAGATTTCTACACGATTGAGCACTGTCTCAGCCTGCGGTCCGGGTT
GTCGGACGCCAGGCCggtGTGATCTCAGATCGAACTCTAGCGACCCTGCGGGCCCTAGGTTCTCTAAAGATGTGCTAACTCGTGACAGAGTCGGACGCCAGGCCCAA
Prefix | bs-1 | Suffix | | Linker_10 | Prefix |

CACTGCCGTATAGGCAGTAATTTTGTTTAACTTTAAGAAGGAGATATACATATGCGTAAAGGCGAAGAACTGTTTACCGGTGTGGTTCCGATTCTGGTGGAACTGGA
GTGACGGCATATCCGTCATTAAAACAAATTGAAATTCTTCCTCTATATGTATACGCATTTCCGCTTCTTGACAAATGGCCACACCAAGGCTAAGACCACCTTGACCT
Csy4 site | RBS | sfGFP

CGGCGATGTTAATGGTCATAAATTCAGTGTTCGCGGCGAAGGTGAAGGCGATGCGACGAACGGCAAACTGACCCTGAAATTTATCTGCACCACGGGTAAACTGCCGG
GCCGCTACAATTACCAGTATTTAAGTCACAAGCGCCGCTTCCACTTCCGCTACGCTGCTTGCCGTTTGACTGGGACTTTAAATAGACGTGGTGCCCATTTGACGGCC
sfGFP

TCCCGTGGCCGACGCTGGTGACCACGCTGACCTATGGCGTTCAATGTTTTGCGCGTTACCCGGATCACATGAAACAGCACGACTTTTTCAAATCGGCCATGCCGGAA
AGGGCACCGGCTGCGACCACTGGTGCGACTGGATACCGCAAGTTACAAAACGCGCAATGGGCCTAGTGTACTTTGTCGTGCTGAAAAAGTTTAGCCGGTACGGCCTT
sfGFP

GGCTATGTGCAGGAACGTACGATTAGCTTTAAAGACGATGGTACGTATAAAACCCGCGCGGAAGTGAAATTCGAAGGCGATACCCTGGTTAACCGTATCGAACTGAA
CCGATACACGTCCTTGCATGCTAATCGAAATTTCTGCTACCATGCATATTTTGGGCGCGCCTTCACTTTAAGCTTCCGCTATGGGACCAATTGGCATAGCTTGACTT
sfGFP

pJ2044_N2only (7295 bp) (from 2462-3638 bp)

AGGTATCGATTTCAAAGAAGACGGCAATATTCTGGGTCATAAACTGGAATATAACTTCAATTCCCACAACGTGTACATCACCGCGGATAAACAGAAAAACGGCATTA
TCCATAGCTAAAGTTTCTTCTGCCGTTATAAGACCCAGTATTTGACCTTATATTGAAGTTAAGGGTGTTGCACATGTAGTGGCGCCTATTTGTCTTTTTGCCGTAAT
sfGFP

AAGCCAATTTCAAAATCCGCCATAATGTGGAAGATGGTAGCGTTCAGCTGGCCGACCACTATCAGCAAAACACGCCGATTGGTGATGGCCCGGTCCTGCTGCCGGAC
TTCGGTTAAAGTTTTAGGCGGTATTACACCTTCTACCATCGCAAGTCGACCGGCTGGTGATAGTCGTTTTGTGCGGCTAACCACTACCGGGCCAGGACGACGGCCTG
sfGFP

AATCACTACCTGAGTACCCAGTCCGTGCTGTCAAAAGATCCGAACGAAAAACGTGACCACATGGTCCTGCTGGAATTTGTGACGGCTGCGGGTATCACCCACGGCAT
TTAGTGATGGACTCATGGGTCAGGCACGACAGTTTTCTAGGCTTGCTTTTTGCACTGGTGTACCAGGACGACCTTAAACACTGCCGACGCCCATAGTGGGTGCCGTA
sfGFP

SbfI
GGACGAACTGTATAAAATGTCCCGCCGTAATACTGACGCCATCACAATCCACAGCATCCTGGATTGGATTGAAGACTAATAATCGCTGGGACGCCCGCCTGCAGGCT
CCTGCTTGACATATTTTACAGGGCGGCATTATGACTGCGGTAGTGTTAGGTGTCGTAGGACCTAACCTAACTTCTGATTATTAGCGACCCTGCGGGCGGACGTCCGA
sfGFP | MarAn20 | Suffix

KpnI
CGGTACCAAATTCCAGAAAAGAGGCCTCCCGAAAGGGGGGCCTTTTTTCGTTTTGGTCCTAATAGATAAAGGATAGGTCTGGTAGTGTTGTTCGTTCTCGCAGGTAA
GCCATGGTTTAAGGTCTTTTCTCCGGAGGGCTTTCCCCCCGGAAAAAAGCAAAACCAGGATTATCTATTTCCTATCCAGACCATCACAACAAGCAAGAGCGTCCATT
L3S2P21 Terminator | Spacer 2.5

ATCAATAATACTCAGCAGTTCCGTAGACTTTTCAGTGGGACAGGGTAGCGATAACAGATAGATTGTAATAAGACACAGTAGGTGCTCGTAGTTGCGTGAAGAGAACC
TAGTTATTATGAGTCGTCAAGGCATCTGAAAAGTCACCCTGTCCCATCGCTATTGTCTATCTAACATTATTCTGTGTCATCCACGAGCATCAACGCACTTCTCTTGG
Spacer 2.5

SalI AatII
GCTCAGGAAATCCAGTCAGAAGTATTGGTAATCGTTGAAAACTCAGTCGACGCACTTACTGAAGACGTCCTATTACACTCGTCGTTGGAAACTGAAGATCAGCCTGC
CGAGTCCTTTAGGTCAGTCTTCATAACCATTAGCAACTTTTGAGTCAGCTGCGTGAATGACTTCTGCAGGATAATGTGAGCAGCAACCTTTGACTTCTAGTCGGACG
Spacer 2.5 | Spa... 5 | Linker_11

GGTCCGGGTTCACTGCCGTATAGGCAGTAATTTTGTTTAACTTTAAGAAGGAGATATACATATGAATCAGTCATTCATCTCGGACATCTTATATGCCGACATCGAAT
CCAGGCCCAAGTGACGGCATATCCGTCATTAAAACAAATTGAAATTCTTCCTCTATATGTATACTTAGTCAGTAAGTAGAGCCTGTAGAATATACGGCTGTAGCTTA
Csy4 site | RBS | RepA70

CGAAGGCTAAGGAACTTACAGTCAATTCCAACAATACTGTCCAGCCGGTCGCGCTTATGCGCTTAGGAGTTTTCGTTCCCAAACCTTCCAAGAGCAAAGGAGAAAGT
GCTTCCGATTCCTTGAATGTCAGTTAAGGTTGTTATGACAGGTCGGCCAGCGCGAATACGCGAATCCTCAAAAGCAAGGGTTTGGAAGGTTCTCGTTTCCTCTTTCA
RepA70

AAGGAAATTGACGCCACCAAAGCCTTCTCTCAACTGGAGATTGCTAAAGCAGAGGGCatggttagtaaaggagaagaaaataacatggcaCTGATTAAGGAGAACAT
TTCCTTTAACTGCGGTGGTTTCGGAAGAGAGTTGACCTCTAACGATTTCGTCTCCCGtaccaatcatttcctcttcttttattgtaccgtGACTAATTCCTCTTGTA
RepA70 | mKate2

GCACATGAAGCTGTACATGGAGGGCACCGTGAACAACCACCACTTCAAGTGCACATCCGAGGGCGAAGGCAAGCCCTACGAGGGCACCCAGACCATGAGAATCAAGg
CGTGTACTTCGACATGTACCTCCCGTGGCACTTGTTGGTGGTGAAGTTCACGTGTAGGCTCCCGCTTCCGTTCGGGATGCTCCCGTGGGTCTGGTACTCTTAGTTCc
mKate2

pJ2044_N2only (7295 bp) (from 3639-4922 bp)

ccGTCGAGGGCGGCCCTCTCCCCTTCGCCTTCGACATCCTGGCTACCAGCTTCATGTACGGCAGCAAAACCTTCATCAACCACACCCAGGGCATCCCCGACTTCTTT
ggCAGCTCCCGCCGGGAGAGGGGAAGCGGAAGCTGTAGGACCGATGGTCGAAGTACATGCCGTCGTTTTGGAAGTAGTTGGTGTGGGTCCCGTAGGGGCTGAAGAAA
mKate2

AAGCAGTCCTTCCCTGAGGGCTTCACATGGGAGAGAGTCACCACATACGAAGACGGGGGCGTGCTGACCGCTACCCAGGACACCAGCCTCCAGGACGGCTGCCTCAT
TTCGTCAGGAAGGGACTCCCGAAGTGTACCCTCTCTCAGTGGTGTATGCTTCTGCCCCCGCACGACTGGCGATGGGTCCTGTGGTCGGAGGTCCTGCCGACGGAGTA
mKate2

CTACAACGTCAAGATCAGAGGGGTGAACTTCCCATCCAACGGCCCTGTGATGCAGAAGAAAACACTCGGCTGGGAGGCCTCCACCGAGaccCTGTACCCCGCTGACG
GATGTTGCAGTTCTAGTCTCCCCACTTGAAGGGTAGGTTGCCGGGACACTACGTCTTCTTTTGTGAGCCGACCCTCCGGAGGTGGCTCtggGACATGGGGCGACTGC
mKate2

GCGGCCTGGAAGGCAGAgcCGACATGGCCCTGAAGCTCGTGGGCGGGGGCCACCTGATCTGCAACTTGAAGACCACATACAGATCCAAGAAACCCGCTAAGAACCTC
CGCCGGACCTTCCGTCTcgGCTGTACCGGGACTTCGAGCACCCGCCCCCGGTGGACTAGACGTTGAACTTCTGGTGTATGTCTAGGTTCTTTGGGCGATTCTTGGAG
mKate2

AAGATGCCCGGCGTCTACTATGTGGACAGAAGACTGGAAAGAATCAAGGAGGCCGACAAAGAGACCTACGTCGAGCAGCACGAGGTGGCTGTGGCCAGATACTGCGA
TTCTACGGGCCGCAGATGATACACCTGTCTTCTGACCTTTCTTAGTTCCTCCGGCTGTTTCTCTGGATGCAGCTCGTCGTGCTCCACCGACACCGGTCTATGACGCT
mKate2

NotI
CCTCCCTAGCAAACTGGGGCACAgAgtctaATAATCGCTGGGACGCCCGGCGGCCGCggaaacacagAAAAAAGCCCGCACCTGACAGTGCGGGCTTTTTTTTTcga
GGAGGGATCGTTTGACCCCGTGTcTcagatTATTAGCGACCCTGCGGGCCGCCGGCGcctttgtgtcTTTTTTCGGGCGTGGACTGTCACGCCCGAAAAAAAAAgct
mKate2 Suffix ECK120033737 Terminator

KasI BbvCI AscI
ccaaaggTAGCGAACGACGAGTCACTGTTGAGGATAAATACTTTCTCTACTAGGCGCCTGTTACACAGGTCCTCAGCGGCGCGCCTTTGTCGGTGAACGCTCTCCTG
ggtttccATCGCTTGCTGCTCAGTGACAACTCCTATTTATGAAAGAGATGATCCGCGGACAATGTGTCCAGGAGTCGCCGCGCGGAAACAGCCACTTGCGAGAGGAC
Linker_8 Spa...15

AGTAGGACAAATCCGCCGGGAGCGGATTTGAACGTTGTGAAGCAACGGCCCGGAGGGTGGCGGGCAGGACGCCCGCCATAAACTGCCAGGCATCAAACTAAGCAGAA
TCATCCTGTTTAGGCGGCCCTCGCCTAAACTTGCAACACTTCGTTGCCGGGCCTCCCACCGCCCGTCCTGCGGGCGGTATTTGACGGTCCGTAGTTTGATTCGTCTT

GGCCATCCTGACGGATGGCCTTTTTGCGTTTCAGATCTACCGGTaaaccagcaatagacataagcggctatttaacgaccctgccctgaaccgacgacaagctgacg
CCGGTAGGACTGCCTACCGGAAAAACGCAAAGTCTAGATGGCCAtttggtcgttatctgtattcgccgataaattgctgggacgggacttggctgctgttcgactgc

accgggtctccgcaagtggcacttttcggggaaatgtgcgcggaacccctatttgtttatttttctaaatacattcaaatatgtatccgctcatgaattaattctta
tggcccagaggcgttcaccgtgaaaagcccctttacacgcgccttggggataaacaaataaaaagatttatgtaagtttatacataggcgagtacttaattaagaat

gaaaaactcatcgagcatcaaatgaaactgcaatttattcatatcaggattatcaataccatatttttgaaaaagccgtttctgtaatgaaggagaaaactcaccga
ctttttgagtagctcgtagtttactttgacgttaaataagtatagtcctaatagttatggtataaaaactttttcggcaaagacattacttcctcttttgagtggct
KanR

ggcagttccataggatggcaagatcctggtatcggtctgcgattccgactcgtccaacatcaatacaacctattaatttcccctcgtcaaaaataaggttatcaagt
ccgtcaaggtatcctaccgttctaggaccatagccagacgctaaggctgagcaggttgtagttatgttggataattaaaggggagcagtttttattccaatagttca
KanR

pJ2044_N2only (7295 bp) (from 4923-6206 bp)

gagaaatcaccatgagtgacgactgaatccggtgagaatggcaaaagtttatgcatttctttccagacttgttcaacaggccagccattacgctcgtcatcaaaatc
ctctttagtggtactcactgctgacttaggccactcttaccgttttcaaatacgtaaagaaaggtctgaacaagttgtccggtcggtaatgcgagcagtagttttag
KanR

actcgcatcaaccaaaccgttattcattcgtgattgcgcctgagcgagacgaaatacgcggtcgctgttaaaaggacaattacaaacaggaatcgaatgcaaccggc
tgagcgtagttggtttggcaataagtaagcactaacgcggactcgctctgctttatgcgccagcgacaattttcctgttaatgtttgtccttagcttacgttggccg
KanR

gcaggaacactgccagcgcatcaacaatattttcacctgaatcaggatattcttctaatacctggaatgctgttttcccggggatcgcagtggtgagtaaccatgca
cgtccttgtgacggtcgcgtagttgttataaaagtggacttagtcctataagaagattatggaccttacgacaaaagggcccctagcgtcaccactcattggtacgt
KanR

tcatcaggagtacggataaaatgcttgatggtcggaagaggcataaattccgtcagccagtttagtctgaccatctcatctgtaacatcattggcaacgctaccttt
agtagtcctcatgcctattttacgaactaccagccttctccgtatttaaggcagtcggtcaaatcagactggtagagtagacattgtagtaaccgttgcgatggaaa
KanR

gccatgtttcagaaacaactctggcgcatcgggcttcccatacaatcgatagattgtcgcacctgattgcccgacattatcgcgagcccatttatacccatataaat
cggtacaaagtctttgttgagaccgcgtagcccgaagggtatgttagctatctaacagcgtggactaacgggctgtaatagcgctcgggtaaatatgggtatattta
KanR

cagcatccatgttggaatttaatcgcggcctagagcaagacgtttcccgttgaatatggctcatactcttcctttttcaatattattgaagcatttatcagggttat
gtcgtaggtacaaccttaaattagcgccggatctcgttctgcaaagggcaacttataccgagtatgagaaggaaaaagttataataacttcgtaaatagtcccaata
KanR

tgtctcatgagcggatacatatttgaatgtatttagaaaaataaacaaataggcatgctagcgcagaaacgtcctagaagatgccaggaggatacttagcagagaga
acagagtactcgcctatgtataaacttacataaatctttttatttgtttatccgtacgatcgcgtctttgcaggatcttctacggtcctcctatgaatcgtctctct
ColA ori

caataaggccggagcgaagccgtttttccataggctccgcccccctgacgaacatcacgaaatctgacgctcaaatcagtggtggcgaaacccgacaggactataaa
gttattccggcctcgcttcggcaaaaaggtatccgaggcggggggactgcttgtagtgctttagactgcgagtttagtcaccaccgctttgggctgtcctgatattt
ColA ori

gataccaggcgtttccccctgatggctccctcttgcgctctcctgttcccgtcctgcggcgtccgtgttgtggtggaggctttacccaaatcaccacgtcccgttcc
ctatggtccgcaaaggggggactaccgagggagaacgcgagaggacaagggcaggacgccgcaggcacaacaccacctccgaaatgggtttagtggtgcagggcaagg
ColA ori

gtgtagacagttcgctccaagctgggctgtgtgcaagaaccccccgttcagcccgactgctgcgccttatccggtaactatcatcttgagtccaacccggaaagaca
cacatctgtcaagcgaggttcgacccgacacacgttcttggggggcaagtcgggctgacgacgcggaataggccattgatagtagaactcaggttgggcctttctgt
ColA ori

cgacaaaacgccactggcagcagccattggtaactgagaattagtggatttagatatcgagagtcttgaagtggtggcctaacagaggctacactgaaaggacagta
gctgttttgcggtgaccgtcgtcggtaaccattgactcttaatcacctaaatctatagctctcagaacttcaccaccggattgtctccgatgtgactttcctgtcat
ColA ori

tttggtatctgcgctccactaaagccagttaccaggttaagcagttccccaactgacttaaccttcgatcaaaccgcctccccaggcggttttttcgtttacagagc
aaaccatagacgcgaggtgatttcggtcaatggtccaattcgtcaaggggttgactgaattggaagctagtttggcggaggggtccgccaaaaaagcaaatgtctcg
ColA ori

pJ2044_N2only (7295 bp) (from 6207-7295 bp)

aggagattacgacgatcgtaaaaggatctcaagaagatcctttacggattcccgacaccatcactctagatttcagtgcaatttatctcttcaaatgtagcacctga
tcctctaatgctgctagcattttcctagagttcttctaggaaatgcctaagggctgtggtagtgagatctaaagtcacgttaaatagagaagtttacatcgtggact
ColA ori

agtcagccccatacgatataagttgtaattctcatgttagtcatgccccgcgcccaccggaaggagctgactgggttgCTCCTAgGGTCTGATTCGTTACCAATTAT
tcagtcggggtatgctatattcaacattaagagtacaatcagtacggggcgcgggtggccttcctcgactgacccaacGAGGATcCCAGACTAAGCAATGGTTAATA

GACAACTTGACGGCTACATCATTCACTTTTTCTTCACAACCGGCACGGAACTCGCTCGGGCTGGCCCCGGTGCATTTTTTAAATACCCGCGAGAAATAGAGTTGATC
CTGTTGAACTGCCGATGTAGTAAGTGAAAAAGAAGTGTTGGCCGTGCCTTGAGCGAGCCCGACCGGGGCCACGTAAAAAATTTATGGGCGCTCTTTATCTCAACTAG
araC

GTCAAAACCAACATTGCGACCGACGGTGGCGATAGGCATCCGGGTGGTGCTCAAAAGCAGCTTCGCCTGGCTGATACGTTGGTCCTCGCGCCAGCTTAAGACGCTAA
CAGTTTTGGTTGTAACGCTGGCTGCCACCGCTATCCGTAGGCCCACCACGAGTTTTCGTCGAAGCGGACCGACTATGCAACCAGGAGCGCGGTCGAATTCTGCGATT
araC

TCCCTAACTGCTGGCGGAAAAGATGTGACAGACGCGACGGCGACAAGCAAACATGCTGTGCGACGCTGGCGATATCAAAATTGCTGTCTGCCAGGTGATCGCTGATG
AGGGATTGACGACCGCCTTTTCTACACTGTCTGCGCTGCCGCTGTTCGTTTGTACGACACGCTGCGACCGCTATAGTTTTAACGACAGACGGTCCACTAGCGACTAC
araC

TACTGACAAGCCTCGCGTACCCGATTATCCATCGGTGGATGGAGCGACTCGTTAATCGCTTCCATGCGCCGCAGTAACAATTGCTCAAGCAGATTTATCGCCAGCAG
ATGACTGTTCGGAGCGCATGGGCTAATAGGTAGCCACCTACCTCGCTGAGCAATTAGCGAAGGTACGCGGCGTCATTGTTAACGAGTTCGTCTAAATAGCGGTCGTC
araC

CTCCGAATAGCGCCCTTCCCCTTGCCCGGCGTTAATGATTTGCCCAAACAGGTCGCTGAAATGCGGCTGGTGCGCTTCATCCGGGCGAAAGAACCCCGTATTGGCAA
GAGGCTTATCGCGGGAAGGGGAACGGGCCGCAATTACTAAACGGGTTTGTCCAGCGACTTTACGCCGACCACGCGAAGTAGGCCCGCTTTCTTGGGGCATAACCGTT
araC

ATATTGACGGCCAGTTAAGCCATTCATGCCAGTAGGCGCGCGGACGAAAGTAAACCCACTGGTGATACCATTCGCGAGCCTCCGGATGACGACCGTAGTGATGAATC
TATAACTGCCGGTCAATTCGGTAAGTACGGTCATCCGCGCGCCTGCTTTCATTTGGGTGACCACTATGGTAAGCGCTCGGAGGCCTACTGCTGGCATCACTACTTAG
araC

TCTCCTGGCGGGAACAGCAAAATATCACCCGGTCGGCAAACAAATTCTCGTCCCTGATTTTTCACCACCCCCTGACCGCGAATGGTGAGATTGAGAATATAACCTTT
AGAGGACCGCCCTTGTCGTTTTATAGTGGGCCAGCCGTTTGTTTAAGAGCAGGGACTAAAAAGTGGTGGGGGACTGGCGCTTACCACTCTAACTCTTATATTGGAAA
araC

CATTCCCAGCGGTCGGTCGATAAAAAAATCGAGATAACCGTTGGCCTCAATCGGCGTTAAACCCGCCACCAGATGGGCATTAAACGAGTATCCCGGCAGCAGGGGAT
GTAAGGGTCGCCAGCCAGCTATTTTTTTAGCTCTATTGGCAACCGGAGTTAGCCGCAATTTGGGCGGTGGTCTACCCGTAATTTGCTCATAGGGCCGTCGTCCCCTA
araC

CATTTTGCGCTTCAGCCAT
GTAAAACGCGAAGTCGGTA
araC

(from 1-1177 bp)

pJ2044_t4 (7455 bp)

ACTTTTCATACTCCCGCCATTCAGAGAAGAAACCAATTGTCCATATTGCATCAGACATTGCCGTCACTGCGTCTTTTACTGGCTCTTCTCGCTAACCAAACCGGTAA
TGAAAAGTATGAGGGCGGTAAGTCTCTTCTTTGGTTAACAGGTATAACGTAGTCTGTAACGGCAGTGACGCAGAAAATGACCGAGAAGAGCGATTGGTTTGGCCATT

CCCCGCTTATTAAAAGCATTCTGTAACAAAGCGGGACCAAAGCCATGACAAAAACGCGTAACAAAAGTGTCTATAATCACGGCAGAAAAGTCCACATTGATTATTTG
GGGGCGAATAATTTTCGTAAGACATTGTTTCGCCCTGGTTTCGGTACTGTTTTTGCGCATTGTTTTCACAGATATTAGTGCCGTCTTTTCAGGTGTAACTAATAAAC

EcoRI
CACGGCGTCACACTTTGCTATGCCATAGCATTTTTATCCATAAGATTAGCGGtTCCTACCTGACGCTTTTTATCGCAACTCTCTACTGTTTCTCCATACCGAATTCA
GTGCCGCAGTGTGAAACGATACGGTATCGTAAAAATAGGTATTCTAATCGCCaAGGATGGACTGCGAAAAATAGCGTTGAGAGATGACAAAGAGGTATGGCTTAAGT
P(BAD) promoter

SacI
TAGGATAGATTCTGGAAACTTTACCGTCCGAGCTCCAGCCTGCGGTCCGGGTTCACTGCCGTATAGGCAGAAGCTAGACTCTAGTGGTTTcAGAGCTATGCTGGAAA
ATCCTATCTAAGACCTTTGAAATGGCAGGCTCGAGGTCGGACGCCAGGCCCAAGTGACGGCATATCCGTCTTCGATCTGAGATCACCAAAgTCTCGATACGACCTTT
Linker_14 | Prefix | Csy4 site | sgRNA-1t4

XhoI
CAGCATAGCAAGTTgAAATAAGGCTAGTCCGTTATCAACTTGAAAAAGTGGCACCGAGTCGGTGCGTTCACTGCCGTATAGGCAGTCGCTGGGACGCCCGCTCGAGC
GTCGTATCGTTCAAcTTTATTCCGATCAGGCAATAGTTGAACTTTTTCACCGTGGCTCAGCCACGCAAGTGACGGCATATCCGTCAGCGACCCTGCGGGCGAGCTCG
sgRNA-1t4 | Csy4 site | Suffix

AATAAACAGTTGATAGGGCTTCTCCGTTACAGCCTGCGGTCCGGGTTCACTGCCGTATAGGCAGTAATTTTGTTTAACTTTAAGAAGGAGATATACATATGGTTTCG
TTATTTGTCAACTATCCCGAAGAGGCAATGTCGGACGCCAGGCCCAAGTGACGGCATATCCGTCATTAAAACAAATTGAAATTCTTCCTCTATATGTATACCAAAGC
Linker_0 | Prefix | Csy4 site | RBS | mKO2

GTTATCAAACCAGAGATGAAAATGCGTTACTATATGGATGGTTCAGTAAATGGTCACGAATTTACTATTGAGGGCGAGGGTACGGGACGCCCATACGAGGGGCACCA
CAATAGTTTGGTCTCTACTTTTACGCAATGATATACCTACCAAGTCATTTACCAGTGCTTAAATGATAACTCCCGCTCCCATGCCCTGCGGGTATGCTCCCCGTGGT
mKO2

GGAAATGACTTTACGCGTCACAATGGCTGAAGGCGGGCCTATGCCGTTTGCGTTCGATCTTGTTAGTCATGTCTTTTGTTACGGTCACCGTGTATTTACTAAATACC
CCTTTACTGAAATGCGCAGTGTTACCGACTTCCGCCCGGATACGGCAAACGCAAGCTAGAACAATCAGTACAGAAAACAATGCCAGTGGCACATAAATGATTTATGG
mKO2

CCGAGGAAATTCCAGACTATTTCAAACAAGCCTTCCCGGAAGGTTTGTCTTGGGAGCGCAGTTTAGAGTTTGAAGACGGTGGCTCGGCCAGCGTGTCAGCTCATATT
GGCTCCTTTAAGGTCTGATAAAGTTTGTTCGGAAGGGCCTTCCAAACAGAACCCTCGCGTCAAATCTCAAACTTCTGCCACCGAGCCGGTCGCACAGTCGAGTATAA
mKO2

AGTCTTCGCGGCAATACATTTTATCACAAGTCAAAGTTCACCGGCGTGAACTTCCCCGCAGACGGCCCAATCATGCAGAATCAAAGTGTTGATTGGGAACCGTCCAC
TCAGAAGCGCCGTTATGTAAAATAGTGTTCAGTTTCAAGTGGCCGCACTTGAAGGGGCGTCTGCCGGGTTAGTACGTCTTAGTTTCACAACTAACCCTTGGCAGGTG
mKO2

AGAGAAGATTACAGCTTCCGATGGAGTCTTAAAGGGCGATGTAACCATGTACTTAAAATTAGAAGGGGGAGGGAACCATAAATGTCAGATGAAGACTACCTATAAGG
TCTCTTCTAATGTCGAAGGCTACCTCAGAATTTCCCGCTACATTGGTACATGAATTTTAATCTTCCCCCTCCCTTGGTATTTACAGTCTACTTCTGATGGATATTCC
mKO2

pJ2044_t4 (7455 bp) (from 1178-2354 bp)

CCGCAAAAGAGATTCTTGAAATGCCCGGAGACCACTACATTGGGCATCGTTTGGTCCGTAAGACAGAAGGAAATATTACTGAACAGGTCGAAGACGCTGTGGCACAC
GGCGTTTTCTCTAAGAACTTTACGGGCCTCTGGTGATGTAACCCGTAGCAAACCAGGCATTCTGTCTTCCTTTATAATGACTTGTCCAGCTTCTGCGACACCGTGTG
mKO2

AGCATGTCCCGCCGTAATACTGACGCCATCACAATCCACAGCATCCTGGATTGGATTGAAGACAACTTGGAGTCGCCGTTGAGTTTAGAAAAAGTTAGTGAACGTAG
TCGTACAGGGCGGCATTATGACTGCGGTAGTGTTAGGTGTCGTAGGACCTAACCTAACTTCTGTTGAACCTCAGCGGCAACTCAAATCTTTTTCAATCACTTGCATC
MarA

TGGTTACTCAAAGTGGCACCTTCAGCGCATGTTTAAGAAGGAAACGGGTCATTCATTGGGTCAATATATTCGTTCTCGCAAGATGACTGAAATTGCCCAGAAATTGA
ACCAATGAGTTTCACCGTGGAAGTCGCGTACAAATTCTTCCTTTGCCCAGTAAGTAACCCAGTTATATAAGCAAGAGCGTTCTACTGACTTTAACGGGTCTTTAACT
MarA

AAGAGTCTAATGAACCTATTTTGTACCTGGCGGAGCGTTACGGCTTTGAAAGTCAGCAAACCCTTACACGTACCTTCAAGAATTACTTTGACGTTCCACCACACAAA
TTCTCAGATTACTTGGATAAAACATGGACCGCCTCGCAATGCCGAAACTTTCAGTCGTTTGGGAATGTGCATGGAAGTTCTTAATGAAACTGCAAGGTGGTGTGTTT
MarA

NcoI
TATCGTATGACCAACATGCAGGGTGAGTCACGTTTTTTGCATCCGTTGAATCATTACAATTCCTAATAATCGCTGGGACGCCCGCCATGGTTCAGCCAAAAAACTTA
ATAGCATACTGGTTGTACGTCCCACTCAGTGCAAAAAACGTAGGCAACTTAGTAATGTTAAGGATTATTAGCGACCCTGCGGGCGGTACCAAGTCGGTTTTTTGAAT
MarA | Suffix | ECK12...ator

AGACCGCCGGTCTTGTCCACTACCTTGCAGTAATGCGGTGGACAGGATCGGCGGTTTTCTTTTCTCTTCTCAATTCTTCTGACCTGTAACGAATAATAGATAGTAAA
TCTGGCGGCCAGAACAGGTGATGGAACGTCATTACGCCACCTGTCCTAGCCGCCAAAAGAAAAGAGAAGAGTTAAGAAGACTGGACATTGCTTATTATCTATCATTT
ECK120029600 Terminator | Spacer 1

GTAGTCTCCGATTGAGTTTTCTCTGCCGAGTCCCACCCAGTTCTGTGATTTCAGTAAGTTGGTAATTGATACACTGTTGCGAGAACTGCTGCCTGGTAGTAGATAGG
CATCAGAGGCTAACTCAAAAGAGACGGCTCAGGGTGGGTCAAGACACTAAAGTCATTCAACCATTAACTATGTGACAACGCTCTTGACGACGGACCATCATCTATCC
Spacer 1

HindIII
TTGTTATTGAGTAAGAAGGTAAAGTGAACGAAATCCCTGAAACTGAGACTGTAGAAAATAAGCTTCAGCCTGCGGTCCGGTTGACAGCTAGCTCAGTCCTAGGTACT
AACAATAACTCATTCTTCCATTTCACTTGCTTTAGGGACTTTGACTCTGACATCTTTTATTCGAAGTCGGACGCCAGGCCAACTGTCGATCGAGTCAGGATCCATGA
Spacer 1 | Prefix | P(BBa_J23102)

GTGCTAGCTCGCTGGGACGCCCGGGGACTACACTTACGAAACTATTGATTGCTCAGCCTGCGGTCCGGccaCACTAGAGTCTAGCTTGAGATCGCTGGGACGCCCGG
CACGATCGAGCGACCCTGCGGGCCCCTGATGTGAATGCTTTGATAACTAACGAGTCGGACGCCAGGCCggtGTGATCTCAGATCGAACTCTAGCGACCCTGCGGGCC
Suffix | Linker_1 | Prefix | bs-1 | Suffix

BamHI
GATCCAAGAGATTTCTACACGATTGAGCACTGTCTCAGCCTGCGGTCCGGGTTCACTGCCGTATAGGCAGTAATTTTGTTTAACTTTAAGAAGGAGATATACATATG
CTAGGTTCTCTAAAGATGTGCTAACTCGTGACAGAGTCGGACGCCAGGCCCAAGTGACGGCATATCCGTCATTAAAACAAATTGAAATTCTTCCTCTATATGTATAC
Linker_10 | Prefix | Csy4 site | RBS

CGTAAAGGCGAAGAACTGTTTACCGGTGTGGTTCCGATTCTGGTGGAACTGGACGGCGATGTTAATGGTCATAAATTCAGTGTTCGCGGCGAAGGTGAAGGCGATGC
GCATTTCCGCTTCTTGACAAATGGCCACACCAAGGCTAAGACCACCTTGACCTGCCGCTACAATTACCAGTATTTAAGTCACAAGCGCCGCTTCCACTTCCGCTACG
sfGFP

pJ2044_t4 (7455 bp) (from 2355-3531 bp)

GACGAACGGCAAACTGACCCTGAAATTTATCTGCACCACGGGTAAACTGCCGGTCCCGTGGCCGACGCTGGTGACCACGCTGACCTATGGCGTTCAATGTTTTGCGC
CTGCTTGCCGTTTGACTGGGACTTTAAATAGACGTGGTGCCCATTTGACGGCCAGGGCACCGGCTGCGACCACTGGTGCGACTGGATACCGCAAGTTACAAAACGCG
sfGFP

GTTACCCGGATCACATGAAACAGCACGACTTTTTCAAATCGGCCATGCCGGAAGGCTATGTGCAGGAACGTACGATTAGCTTTAAAGACGATGGTACGTATAAAACC
CAATGGGCCTAGTGTACTTTGTCGTGCTGAAAAAGTTTAGCCGGTACGGCCTTCCGATACACGTCCTTGCATGCTAATCGAAATTTCTGCTACCATGCATATTTTGG
sfGFP

CGCGCGGAAGTGAAATTCGAAGGCGATACCCTGGTTAACCGTATCGAACTGAAAGGTATCGATTTCAAAGAAGACGGCAATATTCTGGGTCATAAACTGGAATATAA
GCGCGCCTTCACTTTAAGCTTCCGCTATGGGACCAATTGGCATAGCTTGACTTTCCATAGCTAAAGTTTCTTCTGCCGTTATAAGACCCAGTATTTGACCTTATATT
sfGFP

CTTCAATTCCCACAACGTGTACATCACCGCGGATAAACAGAAAAACGGCATTAAAGCCAATTTCAAAATCCGCCATAATGTGGAAGATGGTAGCGTTCAGCTGGCCG
GAAGTTAAGGGTGTTGCACATGTAGTGGCGCCTATTTGTCTTTTTGCCGTAATTTCGGTTAAAGTTTTAGGCGGTATTACACCTTCTACCATCGCAAGTCGACCGGC
sfGFP

ACCACTATCAGCAAAACACGCCGATTGGTGATGGCCCGGTCCTGCTGCCGGACAATCACTACCTGAGTACCCAGTCCGTGCTGTCAAAAGATCCGAACGAAAAACGT
TGGTGATAGTCGTTTTGTGCGGCTAACCACTACCGGGCCAGGACGACGGCCTGTTAGTGATGGACTCATGGGTCAGGCACGACAGTTTTCTAGGCTTGCTTTTTGCA
sfGFP

GACCACATGGTCCTGCTGGAATTTGTGACGGCTGCGGGTATCACCCACGGCATGGACGAACTGTATAAAATGTCCCGCCGTAATACTGACGCCATCACAATCCACAG
CTGGTGTACCAGGACGACCTTAAACACTGCCGACGCCCATAGTGGGTGCCGTACCTGCTTGACATATTTTACAGGGCGGCATTATGACTGCGGTAGTGTTAGGTGTC
sfGFP MarAn20

SbfI KpnI
CATCCTGGATTGGATTGAAGACTAATAATCGCTGGGACGCCCGCCTGCAGGCTCGGTACCAAATTCCAGAAAAGAGGCCTCCCGAAAGGGGGGCCTTTTTTCGTTTT
GTAGGACCTAACCTAACTTCTGATTATTAGCGACCCTGCGGGCGGACGTCCGAGCCATGGTTTAAGGTCTTTTCTCCGGAGGGCTTTCCCCCCGGAAAAAAGCAAAA
MarAn20 Suffix L3S2P21 Terminator

GGTCCTAATAGATAAAGGATAGGTCTGGTAGTGTTGTTCGTTCTCGCAGGTAAATCAATAATACTCAGCAGTTCCGTAGACTTTTCAGTGGGACAGGGTAGCGATAA
CCAGGATTATCTATTTCCTATCCAGACCATCACAACAAGCAAGAGCGTCCATTTAGTTATTATGAGTCGTCAAGGCATCTGAAAAGTCACCCTGTCCCATCGCTATT
Spacer 2.5

SalI
CAGATAGATTGTAATAAGACACAGTAGGTGCTCGTAGTTGCGTGAAGAGAACCGCTCAGGAAATCCAGTCAGAAGTATTGGTAATCGTTGAAAACTCAGTCGACGCA
GTCTATCTAACATTATTCTGTGTCATCCACGAGCATCAACGCACTTCTCTTGGCGAGTCCTTTAGGTCAGTCTTCATAACCATTAGCAACTTTTGAGTCAGCTGCGT
Spacer 2.5

AatII
CTTACTGAAGACGTCCTATTACACTCGTCGTTGGAAACTGAAGATCAGCCTGCGGTCCGGGTTCACTGCCGTATAGGCAGTAATTTTGTTTAACTTTAAGAAGGAGA
GAATGACTTCTGCAGGATAATGTGAGCAGCAACCTTTGACTTCTAGTCGGACGCCAGGCCCAAGTGACGGCATATCCGTCATTAAAACAAATTGAAATTCTTCCTCT
Linker_11 Prefix Csy4 site RBS

TATACATATGAATCAGTCATTCATCTCGGACATCTTATATGCCGACATCGAATCGAAGGCTAAGGAACTTACAGTCAATTCCAACAATACTGTCCAGCCGGTCGCGC
ATATGTATACTTAGTCAGTAAGTAGAGCCTGTAGAATATACGGCTGTAGCTTAGCTTCCGATTCCTTGAATGTCAGTTAAGGTTGTTATGACAGGTCGGCCAGCGCG
RepA70

pJ2044_t4 (7455 bp) (from 3532-4815 bp)

TTATGCGCTTAGGAGTTTTCGTTCCCAAACCTTCCAAGAGCAAAGGAGAAAGTAAGGAAATTGACGCCACCAAAGCCTTCTCTCAACTGGAGATTGCTAAAGCAGAG
AATACGCGAATCCTCAAAAGCAAGGGTTTGGAAGGTTCTCGTTTCCTCTTTCATTCCTTTAACTGCGGTGGTTTCGGAAGAGAGTTGACCTCTAACGATTTCGTCTC
RepA70

GGCatggttagtaaaggagaagaaaataacatggcaCTGATTAAGGAGAACATGCACATGAAGCTGTACATGGAGGGCACCGTGAACAACCACCACTTCAAGTGCAC
CCGtaccaatcatttcctcttcttttattgtaccgtGACTAATTCCTCTTGTACGTGTACTTCGACATGTACCTCCCGTGGCACTTGTTGGTGGTGAAGTTCACGTG
mKate2

ATCCGAGGGCGAAGGCAAGCCCTACGAGGGCACCCAGACCATGAGAATCAAGgccGTCGAGGGCGGCCCTCTCCCCTTCGCCTTCGACATCCTGGCTACCAGCTTCA
TAGGCTCCCGCTTCCGTTCGGGATGCTCCCGTGGGTCTGGTACTCTTAGTTCcggCAGCTCCCGCCGGGAGAGGGGAAGCGGAAGCTGTAGGACCGATGGTCGAAGT
mKate2

TGTACGGCAGCAAAACCTTCATCAACCACACCCAGGGCATCCCCGACTTCTTTAAGCAGTCCTTCCCTGAGGGCTTCACATGGGAGAGAGTCACCACATACGAAGAC
ACATGCCGTCGTTTTGGAAGTAGTTGGTGTGGGTCCCGTAGGGGCTGAAGAAATTCGTCAGGAAGGGACTCCCGAAGTGTACCCTCTCTCAGTGGTGTATGCTTCTG
mKate2

GGGGGCGTGCTGACCGCTACCCAGGACACCAGCCTCCAGGACGGCTGCCTCATCTACAACGTCAAGATCAGAGGGGTGAACTTCCCATCCAACGGCCCTGTGATGCA
CCCCCGCACGACTGGCGATGGGTCCTGTGGTCGGAGGTCCTGCCGACGGAGTAGATGTTGCAGTTCTAGTCTCCCCACTTGAAGGGTAGGTTGCCGGGACACTACGT
mKate2

GAAGAAAACACTCGGCTGGGAGGCCTCCACCGAGaccCTGTACCCCGCTGACGGCGGCCTGGAAGGCAGAgcCGACATGGCCCTGAAGCTCGTGGGCGGGGGCCACC
CTTCTTTTGTGAGCCGACCCTCCGGAGGTGGCTCtggGACATGGGGCGACTGCCGCCGGACCTTCCGTCTcgGCTGTACCGGGACTTCGAGCACCCGCCCCCGGTGG
mKate2

TGATCTGCAACTTGAAGACCACATACAGATCCAAGAAACCCGCTAAGAACCTCAAGATGCCCGGCGTCTACTATGTGGACAGAAGACTGGAAAGAATCAAGGAGGCC
ACTAGACGTTGAACTTCTGGTGTATGTCTAGGTTCTTTGGGCGATTCTTGGAGTTCTACGGGCCGCAGATGATACACCTGTCTTCTGACCTTTCTTAGTTCCTCCGG
mKate2

NotI

GACAAAGAGACCTACGTCGAGCAGCACGAGGTGGCTGTGGCCAGATACTGCGACCTCCCTAGCAAACTGGGGCACAgAgtctaAATAATCGCTGGGACGCCCGGCGGC
CTGTTTCTCTGGATGCAGCTCGTCGTGCTCCACCGACACCGGTCTATGACGCTGGAGGGATCGTTTGACCCCGTGTcTcagatTATTAGCGACCCTGCGGGCCGCCG
mKate2 Suffix

KasI

CGCggaaacacagAAAAAAGCCCGCACCTGACAGTGCGGGCTTTTTTTTTcgaccaaaggTAGCGAACGACGAGTCACTGTTGAGGATAAATACTTTCTCTACTAGG
GCGcctttgtgtcTTTTTTCGGGCGTGGACTGTCACGCCCGAAAAAAAAAgctggtttccATCGCTTGCTGCTCAGTGACAACTCCTATTTATGAAAGAGATGATCC
ECK120033737 Terminator Linker_8

BbvCI AscI

CGCCTGTTACACAGGTCCTCAGCGGCGCGCCTTTGTCGGTGAACGCTCTCCTGAGTAGGACAAATCCGCCGGGAGCGGATTTGAACGTTGTGAAGCAACGGCCCGGA
GCGGACAATGTGTCCAGGAGTCGCCGCGCGGAAACAGCCACTTGCGAGAGGACTCATCCTGTTTAGGCGGCCCTCGCCTAAACTTGCAACACTTCGTTGCCGGGCCT
Spa...15

GGGTGGCGGGCAGGACGCCCGCCATAAACTGCCAGGCATCAAACTAAGCAGAAGGCCATCCTGACGGATGGCCTTTTTGCGTTTCAGATCTACCGGTaaaccagcaa
CCCACCGCCCGTCCTGCGGGCGGTATTTGACGGTCCGTAGTTTGATTCGTCTTCCGGTAGGACTGCCTACCGGAAAAACGCAAAGTCTAGATGGCCAtttggtcgtt

tagacataagcggctatttaacgaccctgccctgaaccgacgacaagctgacgaccgggtctccgcaagtggcacttttcggggaaatgtgcgcggaacccctattt
atctgtattcgccgataaattgctgggacgggacttggctgctgttcgactgctggcccagaggcgttcaccgtgaaaagcccctttacacgcgccttggggataaa

pJ2044_t4 (7455 bp) (from 4816-6099 bp)

gtttatttttctaaatacattcaaatatgtatccgctcatgaattaattcttagaaaaactcatcgagcatcaaatgaaactgcaatttattcatatcaggattatc
caaataaaaagatttatgtaagtttatacataggcgagtacttaattaagaatctttttgagtagctcgtagtttactttgacgttaaataagtatagtcctaatag
KanR

aataccatatttttgaaaaagccgtttctgtaatgaaggagaaaactcaccgaggcagttccataggatggcaagatcctggtatcggtctgcgattccgactcgtc
ttatggtataaaaactttttcggcaaagacattacttcctcttttgagtggctccgtcaaggtatcctaccgttctaggaccatagccagacgctaaggctgagcag
KanR

caacatcaatacaacctattaatttcccctcgtcaaaaataaggttatcaagtgagaaatcaccatgagtgacgactgaatccggtgagaatggcaaaagtttatgc
gttgtagttatgttggataattaaaggggagcagtttttattccaatagttcactctttagtggtactcactgctgacttaggccactcttaccgttttcaaatacg
KanR

atttctttccagacttgttcaacaggccagccattacgctcgtcatcaaaatcactcgcatcaaccaaaccgttattcattcgtgattgcgcctgagcgagacgaaa
taaagaaaggtctgaacaagttgtccggtcggtaatgcgagcagtagttttagtgagcgtagttggtttggcaataagtaagcactaacgcggactcgctctgcttt
KanR

tacgcggtcgctgttaaaaggacaattacaaacaggaatcgaatgcaaccggcgcaggaacactgccagcgcatcaacaatattttcacctgaatcaggatattctt
atgcgccagcgacaattttcctgttaatgtttgtccttagcttacgttggccgcgtccttgtgacggtcgcgtagttgttataaaagtggacttagtcctataagaa
KanR

ctaatacctggaatgctgttttcccggggatcgcagtggtgagtaaccatgcatcatcaggagtacggataaaatgcttgatggtcggaagaggcataaattccgtc
gattatggaccttacgacaaaagggcccctagcgtcaccactcattggtacgtagtagtcctcatgcctattttacgaactaccagccttctccgtatttaaggcag
KanR

agccagtttagtctgaccatctcatctgtaacatcattggcaacgctacctttgccatgtttcagaaacaactctggcgcatcgggcttcccatacaatcgatagat
tcggtcaaatcagactggtagagtagacattgtagtaaccgttgcgatggaaacggtacaaagtctttgttgagaccgcgtagcccgaagggtatgttagctatcta
KanR

tgtcgcacctgattgcccgacattatcgcgagcccatttatacccatataaatcagcatccatgttggaatttaatcgcggcctagagcaagacgtttcccgttgaa
acagcgtggactaacgggctgtaatagcgctcgggtaaatatgggtatatttagtcgtaggtacaaccttaaattagcgccggatctcgttctgcaaagggcaactt
KanR

tatggctcatactcttcctttttcaatattattgaagcatttatcagggttattgtctcatgagcggatacatatttgaatgtatttagaaaaataaacaaataggc
ataccgagtatgagaaggaaaaagttataataacttcgtaaatagtcccaataacagagtactcgcctatgtataaacttacataaatctttttatttgtttatccg
KanR

atgctagcgcagaaacgtcctagaagatgccaggaggatacttagcagagagacaataaggccggagcgaagccgtttttccataggctccgcccccctgacgaaca
tacgatcgcgtctttgcaggatcttctacggtcctcctatgaatcgtctctctgttattccggcctcgcttcggcaaaaaggtatccgaggcggggggactgcttgt
ColA ori

tcacgaaatctgacgctcaaatcagtggtggcgaaacccgacaggactataaagataccaggcgtttccccctgatggctccctcttgcgctctcctgttcccgtcc
agtgctttagactgcgagtttagtcaccaccgctttgggctgtcctgatatttctatggtccgcaaagggggactaccgagggagaacgcgagaggacaagggcagg
ColA ori

tgcggcgtccgtgttgtggtggaggctttacccaaatcaccacgtcccgttccgtgtagacagttcgctccaagctgggctgtgtgcaagaaccccccgttcagccc
acgccgcaggcacaacaccacctccgaaatgggtttagtggtgcagggcaaggcacatctgtcaagcgaggttcgacccgacacacgttcttggggggcaagtcggg
ColA ori

pJ2044_t4 (7455 bp) (from 6100-7383 bp)

```
gactgctgcgccttatccggtaactatcatcttgagtccaacccggaaagacacgacaaaacgccactggcagcagccattggtaactgagaattagtggatttaga
ctgacgacgcggaataggccattgatagtagaactcaggttgggcctttctgtgctgttttgcggtgaccgtcgtcggtaaccattgactcttaatcacctaaatct
                                                 ColA ori

tatcgagagtcttgaagtggtggcctaacagaggctacactgaaggacagtatttggtatctgcgctccactaaagccagttaccaggttaagcagttccccaact
atagctctcagaacttcaccaccggattgtctccgatgtgactttcctgtcataaaccatagacgcgaggtgatttcggtcaatggtccaattcgtcaaggggttga
                                                 ColA ori

gacttaaccttcgatcaaaccgcctccccaggcggtttttttcgtttacagagcaggagattacgacgatcgtaaaaggatctcaagaagatcctttacggattcccg
ctgaattggaagctagtttggcggaggggtccgccaaaaaagcaaatgtctcgtcctctaatgctgctagcattttcctagagttcttctaggaaatgcctaagggc
                                                 ColA ori

acaccatcactctagatttcagtgcaatttatctcttcaaatgtagcacctgaagtcagccccatacgatataagttgtaattctcatgttagtcatgccccgcgcc
tgtggtagtgagatctaaagtcacgttaaatagagaagtttacatcgtggacttcagtcggggtatgctatattcaacattaagagtacaatcagtacggggcgcgg

caccggaaggagctgactgggttgCTCCTAgGGTCTGATTCGTTACCAATTATGACAACTTGACGGCTACATCATTCACTTTTTCTTCACAACCGGCACGGAACTCG
gtggccttcctcgactgacccaacGAGGATcCCAGACTAAGCAATGGTTAATACTGTTGAACTGCCGATGTAGTAAGTGAAAAAGAAGTGTTGGCCGTGCCTTGAGC
                                                                             araC

CTCGGGCTGGCCCCGGTGCATTTTTTAAATACCCGCGAGAAATAGAGTTGATCGTCAAAACCAACATTGCGACCGACGGTGGCGATAGGCATCCGGGTGGTGCTCAA
GAGCCCGACCGGGGCCACGTAAAAAATTTATGGGCGCTCTTTATCTCAACTAGCAGTTTTGGTTGTAACGCTGGCTGCCACCGCTATCCGTAGGCCCACCACGAGTT
                                                    araC

AAGCAGCTTCGCCTGGCTGATACGTTGGTCCTCGCGCCAGCTTAAGACGCTAATCCCTAACTGCTGGCGGAAAAGATGTGACAGACGCGACGGCGACAAGCAAACAT
TTCGTCGAAGCGGACCGACTATGCAACCAGGAGCGCGGTCGAATTCTGCGATTAGGGATTGACGACCGCCTTTTCTACACTGTCTGCGCTGCCGCTGTTCGTTTGTA
                                                    araC

GCTGTGCGACGCTGGCGATATCAAAATTGCTGTCTGCCAGGTGATCGCTGATGTACTGACAAGCCTCGCGTACCCGATTATCCATCGGTGGATGGAGCGACTCGTTA
CGACACGCTGCGACCGCTATAGTTTTAACGACAGACGGTCCACTAGCGACTACATGACTGTTCGGAGCGCATGGGCTAATAGGTAGCCACCTACCTCGCTGAGCAAT
                                                    araC

ATCGCTTCCATGCGCCGCAGTAACAATTGCTCAAGCAGATTTATCGCCAGCAGCTCCGAATAGCGCCCTTCCCCTTGCCCGGCGTTAATGATTTGCCCAAACAGGTC
TAGCGAAGGTACGCGGCGTCATTGTTAACGAGTTCGTCTAAATAGCGGTCGTCGAGGCTTATCGCGGGAAGGGGAACGGGCCGCAATTACTAAACGGGTTTGTCCAG
                                                    araC

GCTGAAATGCGGCTGGTGCGCTTCATCCGGGCGAAAGAACCCCGTATTGGCAAATATTGACGGCCAGTTAAGCCATTCATGCCAGTAGGCGCGCGGACGAAAGTAAA
CGACTTTACGCCGACCACGCGAAGTAGGCCCGCTTTCTTGGGGCATAACCGTTTATAACTGCCGGTCAATTCGGTAAGTACGGTCATCCGCGCGCCTGCTTTCATTT
                                                    araC

CCCACTGGTGATACCATTCGCGAGCCTCCGGATGACGACCGTAGTGATGAATCTCTCCTGGCGGGAACAGCAAAATATCACCCGGTCGGCAAACAAATTCTCGTCCC
GGGTGACCACTATGGTAAGCGCTCGGAGGCCTACTGCTGGCATCACTACTTAGAGAGGACCGCCCTTGTCGTTTTATAGTGGGCCAGCCGTTTGTTTAAGAGCAGGG
                                                    araC

TGATTTTTCACCACCCCCTGACCGCGAATGGTGAGATTGAGAATATAACCTTTCATTCCCAGCGGTCGGTCGATAAAAAAATCGAGATAACCGTTGGCCTCAATCGG
ACTAAAAAGTGGTGGGGGACTGGCGCTTACCACTCTAACTCTTATATTGGAAAGTAAGGGTCGCCAGCCAGCTATTTTTTTAGCTCTATTGGCAACCGGAGTTAGCC
                                                    araC
```

pJ2044_t4 (7455 bp) (from 7384-7455 bp)

CGTTAAACCCGCCACCAGATGGGCATTAAACGAGTATCCCGGCAGCAGGGGATCATTTTGCGCTTCAGCCAT
GCAATTTGGGCGGTGGTCTACCCGTAATTTGCTCATAGGGCCGTCGTCCCCTAGTAAAACGCGAAGTCGGTA

araC

(from 1-1177 bp)

pJ2048.2 (8003 bp)

ACTTTTCATACTCCCGCCATTCAGAGAAGAAACCAATTGTCCATATTGCATCAGACATTGCCGTCACTGCGTCTTTTACTGGCTCTTCTCGCTAACCAAACCGGTAA
TGAAAAGTATGAGGGCGGTAAGTCTCTTCTTTGGTTAACAGGTATAACGTAGTCTGTAACGGCAGTGACGCAGAAAATGACCGAGAAGAGCGATTGGTTTGGCCATT

CCCCGCTTATTAAAAGCATTCTGTAACAAAGCGGGACCAAAGCCATGACAAAAACGCGTAACAAAAGTGTCTATAATCACGGCAGAAAAGTCCACATTGATTATTTG
GGGGCGAATAATTTTCGTAAGACATTGTTTCGCCCTGGTTTCGGTACTGTTTTTGCGCATTGTTTTCACAGATATTAGTGCCGTCTTTTCAGGTGTAACTAATAAAC

EcoRI

CACGGCGTCACACTTTGCTATGCCATAGCATTTTTATCCATAAGATTAGCGGtTCCTACCTGACGCTTTTTATCGCAACTCTCTACTGTTTCTCCATACCGAATTCA
GTGCCGCAGTGTGAAACGATACGGTATCGTAAAAATAGGTATTCTAATCGCCaAGGATGGACTGCGAAAAATAGCGTTGAGAGATGACAAAGAGGTATGGCTTAAGT

P(BAD) promoter

SacI

TAGGATAGATTCTGGAAACTTTACCGTCCGAGCTCCAGCCTGCGGTCCGGGTTCACTGCCGTATAGGCAGTGACTGAGCTAGTGTACTCTGTTTcAGAGCTATGCTG
ATCCTATCTAAGACCTTTGAAATGGCAGGCTCGAGGTCGGACGCCAGGCCCAAGTGACGGCATATCCGTCACTGACTCGATCACATGAGACAAAgTCTCGATACGAC

Linker_14 Prefix Csy4 site sgRNA-3

GAAACAGCATAGCAAGTTgAAATAAGGCTAGTCCGTTATCAACTTGAAAAAGTGGCACCGAGTCGGTGCGTTCACTGCCGTATAGGCAGTCGCTGGGACGCCCGGGG
CTTTGTCGTATCGTTCAAcTTTATTCCGATCAGGCAATAGTTGAACTTTTTCACCGTGGCTCAGCCACGCAAGTGACGGCATATCCGTCAGCGACCCTGCGGGCCCC

sgRNA-3 Csy4 site Suffix

ACTACACTTACGAAACTATTGATTGCTCAGCCTGCGGTCCGGGTTCACTGCCGTATAGGCAGCATCTTAGAGTATGTAGTTTcAGAGCTATGCTGGAAACAGCATAG
TGATGTGAATGCTTTGATAACTAACGAGTCGGACGCCAGGCCCAAGTGACGGCATATCCGTCGTAGAATCTCATACATCAAAgTCTCGATACGACCTTTGTCGTATC

Linker_1 Prefix Csy4 site sgRNA-4t4

XhoI

CAAGTTgAAATAAGGCTAGTCCGTTATCAACTTGAAAAAGTGGCACCGAGTCGGTGCGTTCACTGCCGTATAGGCAGTCGCTGGGACGCCCGCTCGAGCAATAAACA
GTTCAAcTTTATTCCGATCAGGCAATAGTTGAACTTTTTCACCGTGGCTCAGCCACGCAAGTGACGGCATATCCGTCAGCGACCCTGCGGGCGAGCTCGTTATTTGT

sgRNA-4t4 Csy4 site Suffix

GTTGATAGGGCTTCTCCGTTACAGCCTGCGGTCCGGGTTCACTGCCGTATAGGCAGTAATTTTGTTTAACTTTAAGAAGGAGATATACATATGGTTTCGGTTATCAA
CAACTATCCCGAAGAGGCAATGTCGGACGCCAGGCCCAAGTGACGGCATATCCGTCATTAAAACAAATTGAAATTCTTCCTCTATATGTATACCAAAGCCAATAGTT

Linker_0 Prefix Csy4 site RBS mKO2

ACCAGAGATGAAAATGCGTTACTATATGGATGGTTCAGTAAATGGTCACGAATTTACTATTGAGGGCGAGGGTACGGGACGCCCATACGAGGGGCACCAGGAAATGA
TGGTCTCTACTTTTACGCAATGATATACCTACCAAGTCATTTACCAGTGCTTAAATGATAACTCCCGCTCCCATGCCCTGCGGGTATGCTCCCCGTGGTCCTTTACT

mKO2

CTTTACGCGTCACAATGGCTGAAGGCGGGCCTATGCCGTTTGCGTTCGATCTTGTTAGTCATGTCTTTTGTTACGGTCACCGTGTATTTACTAAATACCCCGAGGAA
GAAATGCGCAGTGTTACCGACTTCCGCCCGGATACGGCAAACGCAAGCTAGAACAATCAGTACAGAAAACAATGCCAGTGGCACATAAATGATTTATGGGGCTCCTT

mKO2

ATTCCAGACTATTTCAAACAAGCCTTCCCGGAAGGTTTGTCTTGGGAGCGCAGTTTAGAGTTTGAAGACGGTGGCTCGGCCAGCGTGTCAGCTCATATTAGTCTTCG
TAAGGTCTGATAAAGTTTGTTCGGAAGGGCCTTCCAAACAGAACCCTCGCGTCAAATCTCAAACTTCTGCCACCGAGCCGGTCGCACAGTCGAGTATAATCAGAAGC

mKO2

pJ2048.2 (8003 bp) (from 1178-2354 bp)

```
CGGCAATACATTTTATCACAAGTCAAAGTTCACCGGCGTGAACTTCCCCGCAGACGGCCCAATCATGCAGAATCAAAGTGTTGATTGGGAACCGTCCACAGAGAAGA
GCCGTTATGTAAAATAGTGTTCAGTTTCAAGTGGCCGCACTTGAAGGGGCGTCTGCCGGGTTAGTACGTCTTAGTTTCACAACTAACCCTTGGCAGGTGTCTCTTCT
```
mKO2

```
TTACAGCTTCCGATGGAGTCTTAAAGGGCGATGTAACCATGTACTTAAAATTAGAAGGGGGAGGGAACCATAAATGTCAGATGAAGACTACCTATAAGGCCGCAAAA
AATGTCGAAGGCTACCTCAGAATTTCCCGCTACATTGGTACATGAATTTTAATCTTCCCCCTCCCTTGGTATTTACAGTCTACTTCTGATGGATATTCCGGCGTTTT
```
mKO2

```
GAGATTCTTGAAATGCCCGGAGACCACTACATTGGGCATCGTTTGGTCCGTAAGACAGAAGGAAATATTACTGAACAGGTCGAAGACGCTGTGGCACACAGCATGTC
CTCTAAGAACTTTACGGGCCTCTGGTGATGTAACCCGTAGCAAACCAGGCATTCTGTCTTCCTTTATAATGACTTGTCCAGCTTCTGCGACACCGTGTGTCGTACAG
```
mKO2

```
CCGCCGTAATACTGACGCCATCACAATCCACAGCATCCTGGATTGGATTGAAGACAACTTGGAGTCGCCGTTGAGTTTAGAAAAAGTTAGTGAACGTAGTGGTTACT
GGCGGCATTATGACTGCGGTAGTGTTAGGTGTCGTAGGACCTAACCTAACTTCTGTTGAACCTCAGCGGCAACTCAAATCTTTTTCAATCACTTGCATCACCAATGA
```
MarA

```
CAAAGTGGCACCTTCAGCGCATGTTTAAGAAGGAAACGGGTCATTCATTGGGTCAATATATTCGTTCTCGCAAGATGACTGAAATTGCCCAGAAATTGAAAGAGTCT
GTTTCACCGTGGAAGTCGCGTACAAATTCTTCCTTTGCCCAGTAAGTAACCCAGTTATATAAGCAAGAGCGTTCTACTGACTTTAACGGGTCTTTAACTTTCTCAGA
```
MarA

```
AATGAACCTATTTTGTACCTGGCGGAGCGTTACGGCTTTGAAAGTCAGCAAACCCTTACACGTACCTTCAAGAATTACTTTGACGTTCCACCACACAAATATCGTAT
TTACTTGGATAAAACATGGACCGCCTCGCAATGCCGAAACTTTCAGTCGTTTGGGAATGTGCATGGAAGTTCTTAATGAAACTGCAAGGTGGTGTGTTTATAGCATA
```
MarA

NcoI

```
GACCAACATGCAGGGTGAGTCACGTTTTTTGCATCCGTTGAATCATTACAATTCCTAATAATCGCTGGGACGCCCGCCATGGTTCAGCCAAAAAACTTAAGACCGCC
CTGGTTGTACGTCCCACTCAGTGCAAAAAACGTAGGCAACTTAGTAATGTTAAGGATTATTAGCGACCCTGCGGGCGGTACCAAGTCGGTTTTTTGAATTCTGGCGG
```
MarA | Suffix | ECK120029...rminator

```
GGTCTTGTCCACTACCTTGCAGTAATGCGGTGGACAGGATCGGCGGTTTTCTTTTCTCTTCTCAATTCTTCTGACCTGTAACGAATAATAGATAGTAAAGTAGTCTC
CCAGAACAGGTGATGGAACGTCATTACGCCACCTGTCCTAGCCGCCAAAAGAAAAGAGAAGAGTTAAGAAGACTGGACATTGCTTATTATCTATCATTTCATCAGAG
```
ECK120029600 Terminator | Spacer 1

```
CGATTGAGTTTTCTCTGCCGAGTCCCACCCAGTTCTGTGATTTCAGTAAGTTGGTAATTGATACACTGTTGCGAGAACTGCTGCCTGGTAGTAGATAGGTTGTTATT
GCTAACTCAAAAGAGACGGCTCAGGGTGGGTCAAGACACTAAAGTCATTCAACCATTAACTATGTGACAACGCTCTTGACGACGGACCATCATCTATCCAACAATAA
```
Spacer 1

HindIII

```
GAGTAAGAAGGTAAAGTGAACGAAATCCCTGAAACTGAGACTGTAGAAAATAAGCTTCAGCCTGCGGTCCGGttgacggctagctcagtcctaggtacagtgctagc
CTCATTCTTCCATTTCACTTGCTTTAGGGACTTTGACTCTGACATCTTTTATTCGAAGTCGGACGCCAGGCCaactgccgatcgagtcaggatccatgtcacgatcg
```
Spacer 1 | Prefix | P(BBa_J23100)

BamHI

```
TCGCTGGGACGCCCGAGATAGCCGTTACACAGGTGACACTTATTTCAGCCTGCGGTCCGGccaTACATACTCTAAGATGTGTCTCGCTGGGACGCCCGGGATCCAAG
AGCGACCCTGCGGGCTCTATCGGCAATGTGTCCACTGTGAATAAAGTCGGACGCCAGGCCggtATGTATGAGATTCTACACAGAGCGACCCTGCGGGCCCTAGGTTC
```
Suffix | Linker_24 | Prefix | bs-4 | Suffix

pJ2048.2 (8003 bp) (from 2355-3531 bp)

```
AGATTTCTACACGATTGAGCACTGTCTCAGCCTGCGGTCCGGGTTCACTGCCGTATAGGCAGTAATTTTGTTTAACTTTAAGAAGGAGATATACATATGCGTAAAGG
TCTAAAGATGTGCTAACTCGTGACAGAGTCGGACGCCAGGCCCAAGTGACGGCATATCCGTCATTAAAACAAATTGAAATTCTTCCTCTATATGTATACGCATTTCC
```
Linker_10 | Prefix | Csy4 site | RBS | sfGFP

```
CGAAGAACTGTTTACCGGTGTGGTTCCGATTCTGGTGGAACTGGACGGCGATGTTAATGGTCATAAATTCAGTGTTCGCGGCGAAGGTGAAGGCGATGCGACGAACG
GCTTCTTGACAAATGGCCACACCAAGGCTAAGACCACCTTGACCTGCCGCTACAATTACCAGTATTTAAGTCACAAGCGCCGCTTCCACTTCCGCTACGCTGCTTGC
```
sfGFP

```
GCAAACTGACCCTGAAATTTATCTGCACCACGGGTAAACTGCCGGTCCCGTGGCCGACGCTGGTGACCACGCTGACCTATGGCGTTCAATGTTTTGCGCGTTACCCG
CGTTTGACTGGGACTTTAAATAGACGTGGTGCCCATTTGACGGCCAGGGCACCGGCTGCGACCACTGGTGCGACTGGATACCGCAAGTTACAAAACGCGCAATGGGC
```
sfGFP

```
GATCACATGAAACAGCACGACTTTTTCAAATCGGCCATGCCGGAAGGCTATGTGCAGGAACGTACGATTAGCTTTAAAGACGATGGTACGTATAAAACCCGCGCGGA
CTAGTGTACTTTGTCGTGCTGAAAAAGTTTAGCCGGTACGGCCTTCCGATACACGTCCTTGCATGCTAATCGAAATTTCTGCTACCATGCATATTTTGGGCGCGCCT
```
sfGFP

```
AGTGAAATTCGAAGGCGATACCCTGGTTAACCGTATCGAACTGAAAGGTATCGATTTCAAAGAAGACGGCAATATTCTGGGTCATAAACTGGAATATAACTTCAATT
TCACTTTAAGCTTCCGCTATGGGACCAATTGGCATAGCTTGACTTTCCATAGCTAAAGTTTCTTCTGCCGTTATAAGACCCAGTATTTGACCTTATATTGAAGTTAA
```
sfGFP

```
CCCACAACGTGTACATCACCGCGGATAAACAGAAAAACGGCATTAAAGCCAATTTCAAAATCCGCCATAATGTGGAAGATGGTAGCGTTCAGCTGGCCGACCACTAT
GGGTGTTGCACATGTAGTGGCGCCTATTTGTCTTTTTGCCGTAATTTCGGTTAAAGTTTTAGGCGGTATTACACCTTCTACCATCGCAAGTCGACCGGCTGGTGATA
```
sfGFP

```
CAGCAAAACACGCCGATTGGTGATGGCCCGGTCCTGCTGCCGGACAATCACTACCTGAGTACCCAGTCCGTGCTGTCAAAAGATCCGAACGAAAAACGTGACCACAT
GTCGTTTTGTGCGGCTAACCACTACCGGGCCAGGACGACGGCCTGTTAGTGATGGACTCATGGGTCAGGCACGACAGTTTTCTAGGCTTGCTTTTTGCACTGGTGTA
```
sfGFP

```
GGTCCTGCTGGAATTTGTGACGGCTGCGGGTATCACCCACGGCATGGACGAACTGTATAAAATGTCCCGCCGTAATACTGACGCCATCACAATCCACAGCATCCTGG
CCAGGACGACCTTAAACACTGCCGACGCCCATAGTGGGTGCCGTACCTGCTTGACATATTTTACAGGGCGGCATTATGACTGCGGTAGTGTTAGGTGTCGTAGGACC
```
sfGFP | MarAn20

SbfI | KpnI

```
ATTGGATTGAAGACTAATAATCGCTGGGACGCCCGCCTGCAGGCTCGGTACCAAATTCCAGAAAAGAGGCCTCCCGAAAGGGGGGCCTTTTTTCGTTTTGGTCCTAA
TAACCTAACTTCTGATTATTAGCGACCCTGCGGGCGGACGTCCGAGCCATGGTTTAAGGTCTTTTCTCCGGAGGGCTTTCCCCCCGGAAAAAAGCAAAACCAGGATT
```
MarAn20 | Suffix | L3S2P21 Terminator

```
TAGATAAAGGATAGGTCTGGTAGTGTTGTTCGTTCTCGCAGGTAAATCAATAATACTCAGCAGTTCCGTAGACTTTTCAGTGGGACAGGGTAGCGATAACAGATAGA
ATCTATTTCCTATCCAGACCATCACAACAAGCAAGAGCGTCCATTTAGTTATTATGAGTCGTCAAGGCATCTGAAAAGTCACCCTGTCCCATCGCTATTGTCTATCT
```
Spacer 2.5

SalI

```
TTGTAATAAGACACAGTAGGTGCTCGTAGTTGCGTGAAGAGAACCGCTCAGGAAATCCAGTCAGAAGTATTGGTAATCGTTGAAAACTCAGTCGACCAGCCTGCGGT
AACATTATTCTGTGTCATCCACGAGCATCAACGCACTTCTCTTGGCGAGTCCTTTAGGTCAGTCTTCATAACCATTAGCAACTTTTGAGTCAGCTGGTCGGACGCCA
```
Spacer 2.5 | Prefix

pJ2048.2 (8003 bp) (from 3532-4815 bp)

CCGGTTTACGGCTAGCTCAGTCCTAGGTATTATGCTAGCTCGCTGGGACGCCCGAGTGACGACTGCGAAGTAACCTCTATTTATCAGCCTGCGGTCCGGccaAGAGT
GGCCAAATGCCGATCGAGTCAGGATCCATAATACGATCGAGCGACCCTGCGGGCTCACTGCTGACGCTTCATTGGAGATAAATAGTCGGACGCCAGGCCggtTCTCA
P(BBa_J23150) | Suffix | Linker_25 | Prefix

ACACTAGCTCAGTCATCGCTGGGACGCCCGGTAGTGCTTATCAGACCCAATACTGTTGAACAGCCTGCGGTCCGGGTTCACTGCCGTATAGGCAGGACACATCTTAG
TGTGATCGAGTCAGTAGCGACCCTGCGGGCCATCACGAATAGTCTGGGTTATGACAACTTGTCGGACGCCAGGCCCAAGTGACGGCATATCCGTCCTGTGTAGAATC
bs-3 | Suffix | Linker_17 | Prefix | Csy4 site | sgRNA-4

AGTATGTAGTTTcAGAGCTATGCTGGAAACAGCATAGCAAGTTgAAATAAGGCTAGTCCGTTATCAACTTGAAAAAGTGGCACCGAGTCGGTGCGTTCACTGCCGTA
TCATACATCAAAgTCTCGATACGACCTTTGTCGTATCGTTCAAcTTTATTCCGATCAGGCAATAGTTGAACTTTTTCACCGTGGCTCAGCCACGCAAGTGACGGCAT
sgRNA-4 | Csy...te

AatII

TAGGCAGTCGCTGGGACGCCCGGACGTCCTATTACACTCGTCGTTGGAAACTGAAGATCAGCCTGCGGTCCGGGTTCACTGCCGTATAGGCAGTAATTTTGTTTAAC
ATCCGTCAGCGACCCTGCGGGCCTGCAGGATAATGTGAGCAGCAACCTTTGACTTCTAGTCGGACGCCAGGCCCAAGTGACGGCATATCCGTCATTAAAACAAATTG
Suffix | Linker_11 | Prefix | Csy4 site | RBS

TTTAAGAAGGAGATATACATATGAATCAGTCATTCATCTCGGACATCTTATATGCCGACATCGAATCGAAGGCTAAGGAACTTACAGTCAATTCCAACAATACTGTC
AAATTCTTCCTCTATATGTATACTTAGTCAGTAAGTAGAGCCTGTAGAATATACGGCTGTAGCTTAGCTTCCGATTCCTTGAATGTCAGTTAAGGTTGTTATGACAG
RBS | RepA70

CAGCCGGTCGCGCTTATGCGCTTAGGAGTTTTCGTTCCCAAACCTTCCAAGAGCAAAGGAGAAAGTAAGGAAATTGACGCCACCAAAGCCTTCTCTCAACTGGAGAT
GTCGGCCAGCGCGAATACGCGAATCCTCAAAAGCAAGGGTTTGGAAGGTTCTCGTTTCCTCTTTCATTCCTTTAACTGCGGTGGTTTCGGAAGAGAGTTGACCTCTA
RepA70

TGCTAAAGCAGAGGGCatggttagtaaaggagaagaaaataacatggcaCTGATTAAGGAGAACATGCACATGAAGCTGTACATGGAGGGCACCGTGAACAACCACC
ACGATTTCGTCTCCCGtaccaatcatttcctcttcttttattgtaccgtGACTAATTCCTCTTGTACGTGTACTTCGACATGTACCTCCCGTGGCACTTGTTGGTGG
RepA70 | mKate2

ACTTCAAGTGCACATCCGAGGGCGAAGGCAAGCCCTACGAGGGCACCCAGACCATGAGAATCAAGgccGTCGAGGGCGGCCCTCTCCCCTTCGCCTTCGACATCCTG
TGAAGTTCACGTGTAGGCTCCCGCTTCCGTTCGGGATGCTCCCGTGGGTCTGGTACTCTTAGTTCcggCAGCTCCCGCCGGGAGAGGGGAAGCGGAAGCTGTAGGAC
mKate2

GCTACCAGCTTCATGTACGGCAGCAAAACCTTCATCAACCACACCCAGGGCATCCCCGACTTCTTTAAGCAGTCCTTCCCTGAGGGCTTCACATGGGAGAGAGTCAC
CGATGGTCGAAGTACATGCCGTCGTTTTGGAAGTAGTTGGTGTGGGTCCCGTAGGGGCTGAAGAAATTCGTCAGGAAGGGACTCCCGAAGTGTACCCTCTCTCAGTG
mKate2

CACATACGAAGACGGGGGCGTGCTGACCGCTACCCAGGACACCAGCCTCCAGGACGGCTGCCTCATCTACAACGTCAAGATCAGAGGGGTGAACTTCCCATCCAACG
GTGTATGCTTCTGCCCCCGCACGACTGGCGATGGGTCCTGTGGTCGGAGGTCCTGCCGACGGAGTAGATGTTGCAGTTCTAGTCTCCCCACTTGAAGGGTAGGTTGC
mKate2

GCCCTGTGATGCAGAAGAAAACACTCGGCTGGGAGGCCTCCACCGAGaccCTGTACCCCGCTGACGGCGGCCTGGAAGGCAGAgcCGACATGGCCCTGAAGCTCGTG
CGGGACACTACGTCTTCTTTTGTGAGCCGACCCTCCGGAGGTGGCTCtggGACATGGGGCGACTGCCGCCGGACCTTCCGTCTcgGCTGTACCGGGACTTCGAGCAC
mKate2

GGCGGGGGCCACCTGATCTGCAACTTGAAGACCACATACAGATCCAAGAAACCCGCTAAGAACCTCAAGATGCCCGGCGTCTACTATGTGGACAGAAGACTGGAAAG
CCGCCCCCGGTGGACTAGACGTTGAACTTCTGGTGTATGTCTAGGTTCTTTGGGCGATTCTTGGAGTTCTACGGGCCGCAGATGATACACCTGTCTTCTGACCTTTC
mKate2

pJ2048.2 (8003 bp) (from 4816-6099 bp)

AATCAAGGAGGCCGACAAAGAGACCTACGTCGAGCAGCACGAGGTGGCTGTGGCCAGATACTGCGACCTCCCTAGCAAACTGGGGCACAgAgtctaATAATCGCTGG
TTAGTTCCTCCGGCTGTTTCTCTGGATGCAGCTCGTCGTGCTCCACCGACACCGGTCTATGACGCTGGAGGGATCGTTTGACCCCGTGTcTcagatTATTAGCGACC
mKate2

NotI
GACGCCCGGCGGCCGCggaaacacagAAAAAAGCCCGCACCTGACAGTGCGGGCTTTTTTTTTcgaccaaaggTAGCGAACGACGAGTCACTGTTGAGGATAAATAC
CTGCGGGCCGCCGGCGcctttgtgtcTTTTTTCGGGCGTGGACTGTCACGCCCGAAAAAAAAAgctggtttccATCGCTTGCTGCTCAGTGACAACTCCTATTTATG
ECK120033737 Terminator
Linker_8

KasI BbvCI AscI
TTTCTCTACTAGGCGCCTGTTACACAGGTCCTCAGCGGCGCGCCTTTGTCGGTGAACGCTCTCCTGAGTAGGACAAATCCGCCGGGAGCGGATTTGAACGTTGTGAA
AAAGAGATGATCCGCGGACAATGTGTCCAGGAGTCGCCGCGCGGAAACAGCCACTTGCGAGAGGACTCATCCTGTTTAGGCGGCCCTCGCCTAAACTTGCAACACTT
Li...8
Spa...15

GCAACGGCCCGGAGGGTGGCGGGCAGGACGCCCGCCATAAACTGCCAGGCATCAAACTAAGCAGAAGGCCATCCTGACGGATGGCCTTTTTGCGTTTCAGATCTACC
CGTTGCCGGGCCTCCCACCGCCCGTCCTGCGGGCGGTATTTGACGGTCCGTAGTTTGATTCGTCTTCCGGTAGGACTGCCTACCGGAAAAACGCAAAGTCTAGATGG

GGTaaaccagcaatagacataagcggctatttaacgaccctgccctgaaccgacgacaagctgacgaccgggtctccgcaagtggcacttttcggggaaatgtgcgc
CCAtttggtcgttatctgtattcgccgataaattgctgggacgggacttggctgctgttcgactgctggcccagaggcgttcaccgtgaaaagcccctttacacgcg

ggaacccctatttgtttatttttctaaatacattcaaatatgtatccgctcatgaattaattcttagaaaaactcatcgagcatcaaatgaaactgcaatttattca
ccttggggataaacaaataaaaagatttatgtaagtttatacataggcgagtacttaattaagaatctttttgagtagctcgtagtttactttgacgttaaataagt
KanR

tatcaggattatcaataccatatttttgaaaaagccgtttctgtaatgaaggagaaaactcaccgaggcagttccataggatggcaagatcctggtatcggtctgcg
atagtcctaatagttatggtataaaaactttttcggcaaagacattacttcctcttttgagtggctccgtcaaggtatcctaccgttctaggaccatagccagacgc
KanR

attccgactcgtccaacatcaatacaacctattaatttcccctcgtcaaaaataaggttatcaagtgagaaatcaccatgagtgacgactgaatccggtgagaatgg
taaggctgagcaggttgtagttatgttggataattaaaggggagcagtttttattccaatagttcactctttagtggtactcactgctgacttaggccactcttacc
KanR

caaaagtttatgcatttctttccagacttgttcaacaggccagccattacgctcgtcatcaaaatcactcgcatcaaccaaaccgttattcattcgtgattgcgcct
gttttcaaatacgtaaagaaaggtctgaacaagttgtccggtcggtaatgcgagcagtagttttagtgagcgtagttggtttggcaataagtaagcactaacgcgga
KanR

gagcgagacgaaatacgcggtcgctgttaaaaggacaattacaaacaggaatcgaatgcaaccggcgcaggaacactgccagcgcatcaacaatattttcacctgaa
ctcgctctgctttatgcgccagcgacaattttcctgttaatgtttgtccttagcttacgttggccgcgtccttgtgacggtcgcgtagttgttataaaagtggactt
KanR

tcaggatattcttctaatacctggaatgctgttttcccggggatcgcagtggtgagtaaccatgcatcatcaggagtacggataaaatgcttgatggtcggaagagg
agtcctataagaagattatggaccttacgacaaaagggcccctagcgtcaccactcattggtacgtagtagtcctcatgcctattttacgaactaccagccttctcc
KanR

cataaattccgtcagccagtttagtctgaccatctcatctgtaacatcattggcaacgctacctttgccatgtttcagaaacaactctggcgcatcgggcttcccat
gtatttaaggcagtcggtcaaatcagactggtagagtagacattgtagtaaccgttgcgatggaaacggtacaaagtctttgttgagaccgcgtagcccgaagggta
KanR

pJ2048.2 (8003 bp) (from 6100-7383 bp)

acaatcgatagattgtcgcacctgattgcccgacattatcgcgagcccatttatacccatataaatcagcatccatgttggaatttaatcgcggcctagagcaagac
tgttagctatctaacagcgtggactaacgggctgtaatagcgctcgggtaaatatgggtatatttagtcgtaggtacaaccttaaattagcgccggatctcgttctg
KanR

gtttcccgttgaatatggctcatactcttcctttttcaatattattgaagcatttatcagggttattgtctcatgagcggatacatatttgaatgtatttagaaaaa
caaagggcaacttataccgagtatgagaaggaaaaagttataataacttcgtaaatagtcccaataacagagtactcgcctatgtataaacttacataaatcttttt
KanR

taaacaaataggcatgctagcgcagaaacgtcctagaagatgccaggaggatacttagcagagagacaataaggccggagcgaagccgtttttccataggctccgcc
atttgtttatccgtacgatcgcgtctttgcaggatcttctacggtcctcctatgaatcgtctctctgttattccggcctcgcttcggcaaaaaggtatccgaggcgg
ColA ori

cccctgacgaacatcacgaaatctgacgctcaaatcagtggtggcgaaacccgacaggactataaagataccaggcgtttccccctgatggctccctcttgcgctct
ggggactgcttgtagtgctttagactgcgagtttagtcaccaccgctttgggctgtcctgatatttctatggtccgcaaagggggactaccgagggagaacgcgaga
ColA ori

cctgttcccgtcctgcggcgtccgtgttgtggtggaggctttacccaaatcaccacgtcccgttccgtgtagacagttcgctccaagctgggctgtgtgcaagaacc
ggacaagggcaggacgccgcaggcacaacaccacctccgaaatgggtttagtggtgcagggcaaggcacatctgtcaagcgaggttcgacccgacacacgttcttgg
ColA ori

ccccgttcagcccgactgctgcgccttatccggtaactatcatcttgagtccaacccggaaagacacgacaaaacgccactggcagcagccattggtaactgagaat
ggggcaagtcgggctgacgacgcggaataggccattgatagtagaactcaggttgggcctttctgtgctgttttgcggtgaccgtcgtcggtaaccattgactctta
ColA ori

tagtggatttagatatcgagagtcttgaagtggtggcctaacagaggctacactgaaaggacagtatttggtatctgcgctccactaaagccagttaccaggttaag
atcacctaaatctatagctctcagaacttcaccaccggattgtctccgatgtgactttcctgtcataaaccatagacgcgaggtgatttcggtcaatggtccaattc
ColA ori

cagttccccaactgacttaaccttcgatcaaaccgcctccccaggcggtttttcgtttacagagcaggagattacgacgatcgtaaaaggatctcaagaagatcct
gtcaaggggttgactgaattggaagctagtttggcggaggggtccgccaaaaagcaaatgtctcgtcctctaatgctgctagcattttcctagagttcttctagga
ColA ori

ttacggattcccgacaccatcactctagatttcagtgcaatttatctcttcaaatgtagcacctgaagtcagccccatacgatataagttgtaattctcatgttagt
aatgcctaagggctgtggtagtgagatctaaagtcacgttaaatagagaagtttacatcgtggacttcagtcggggtatgctatattcaacattaagagtacaatca
ColA ori

catgccccgcgcccaccggaaggagctgactgggttgCTCCTAgGGTCTGATTCGTTACCAATTATGACAACTTGACGGCTACATCATTCACTTTTTCTTCACAACC
gtacggggcgcgggtggccttcctcgactgacccaacGAGGATcCCAGACTAAGCAATGGTTAATACTGTTGAACTGCCGATGTAGTAAGTGAAAAAGAAGTGTTGG
araC

GGCACGGAACTCGCTCGGGCTGGCCCCGGTGCATTTTTTAAATACCCGCGAGAAATAGAGTTGATCGTCAAAACCAACATTGCGACCGACGGTGGCGATAGGCATCC
CCGTGCCTTGAGCGAGCCCGACCGGGGCCACGTAAAAAATTTATGGGCGCTCTTTATCTCAACTAGCAGTTTTGGTTGTAACGCTGGCTGCCACCGCTATCCGTAGG
araC

GGGTGGTGCTCAAAAGCAGCTTCGCCTGGCTGATACGTTGGTCCTCGCGCCAGCTTAAGACGCTAATCCCTAACTGCTGGCGGAAAAGATGTGACAGACGCGACGGC
CCCACCACGAGTTTTCGTCGAAGCGGACCGACTATGCAACCAGGAGCGCGGTCGAATTCTGCGATTAGGGATTGACGACCGCCTTTTCTACACTGTCTGCGCTGCCG
araC

GACAAGCAAACATGCTGTGCGACGCTGGCGATATCAAAATTGCTGTCTGCCAGGTGATCGCTGATGTACTGACAAGCCTCGCGTACCCGATTATCCATCGGTGGATG
CTGTTCGTTTGTACGACACGCTGCGACCGCTATAGTTTTAACGACAGACGGTCCACTAGCGACTACATGACTGTTCGGAGCGCATGGGCTAATAGGTAGCCACCTAC
araC

GAGCGACTCGTTAATCGCTTCCATGCGCCGCAGTAACAATTGCTCAAGCAGATTTATCGCCAGCAGCTCCGAATAGCGCCCTTCCCCTTGCCCGGCGTTAATGATTT
CTCGCTGAGCAATTAGCGAAGGTACGCGGCGTCATTGTTAACGAGTTCGTCTAAATAGCGGTCGTCGAGGCTTATCGCGGGAAGGGGAACGGGCCGCAATTACTAAA
araC

GCCCAAACAGGTCGCTGAAATGCGGCTGGTGCGCTTCATCCGGGCGAAAGAACCCCGTATTGGCAAATATTGACGGCCAGTTAAGCCATTCATGCCAGTAGGCGCGC
CGGGTTTGTCCAGCGACTTTACGCCGACCACGCGAAGTAGGCCCGCTTTCTTGGGGCATAACCGTTTATAACTGCCGGTCAATTCGGTAAGTACGGTCATCCGCGCG
araC

GGACGAAAGTAAACCCACTGGTGATACCATTCGCGAGCCTCCGGATGACGACCGTAGTGATGAATCTCTCCTGGCGGGAACAGCAAAATATCACCCGGTCGGCAAAC
CCTGCTTTCATTTGGGTGACCACTATGGTAAGCGCTCGGAGGCCTACTGCTGGCATCACTACTTAGAGAGGACCGCCCTTGTCGTTTTATAGTGGGCCAGCCGTTTG
araC

AAATTCTCGTCCCTGATTTTTCACCACCCCCTGACCGCGAATGGTGAGATTGAGAATATAACCTTTCATTCCCAGCGGTCGGTCGATAAAAAAATCGAGATAACCGT
TTTAAGAGCAGGGACTAAAAAGTGGTGGGGGACTGGCGCTTACCACTCTAACTCTTATATTGGAAAGTAAGGGTCGCCAGCCAGCTATTTTTTTAGCTCTATTGGCA
araC

TGGCCTCAATCGGCGTTAAACCCGCCACCAGATGGGCATTAAACGAGTATCCCGGCAGCAGGGGATCATTTTGCGCTTCAGCCAT
ACCGGAGTTAGCCGCAATTTGGGCGGTGGTCTACCCGTAATTTGCTCATAGGGCCGTCGTCCCCTAGTAAAACGCGAAGTCGGTA
araC

(from 1-1177 bp)

pJ2048.2_Gs (5853 bp)

ACTTTTCATACTCCCGCCATTCAGAGAAGAAACCAATTGTCCATATTGCATCAGACATTGCCGTCACTGCGTCTTTTACTGGCTCTTCTCGCTAACCAAACCGGTAA
TGAAAAGTATGAGGGCGGTAAGTCTCTTCTTTGGTTAACAGGTATAACGTAGTCTGTAACGGCAGTGACGCAGAAAATGACCGAGAAGAGCGATTGGTTTGGCCATT

CCCCGCTTATTAAAAGCATTCTGTAACAAAGCGGGACCAAAGCCATGACAAAAACGCGTAACAAAAGTGTCTATAATCACGGCAGAAAAGTCCACATTGATTATTTG
GGGGCGAATAATTTTCGTAAGACATTGTTTCGCCCTGGTTTCGGTACTGTTTTTGCGCATTGTTTTCACAGATATTAGTGCCGTCTTTTCAGGTGTAACTAATAAAC

EcoRI

CACGGCGTCACACTTTGCTATGCCATAGCATTTTTATCCATAAGATTAGCGGtTCCTACCTGACGCTTTTTATCGCAACTCTCTACTGTTTCTCCATACCGAATTCA
GTGCCGCAGTGTGAAACGATACGGTATCGTAAAAATAGGTATTCTAATCGCCaAGGATGGACTGCGAAAAATAGCGTTGAGAGATGACAAAGAGGTATGGCTTAAGT

P(BAD) promoter

SacI

TAGGATAGATTCTGGAAACTTTACCGTCCGAGCTCCAGCCTGCGGTCCGGGTTCACTGCCGTATAGGCAGTGACTGAGCTAGTGTACTCTGTTTcAGAGCTATGCTG
ATCCTATCTAAGACCTTTGAAATGGCAGGCTCGAGGTCGGACGCCAGGCCCAAGTGACGGCATATCCGTCACTGACTCGATCACATGAGACAAAgTCTCGATACGAC

Linker_14 | Prefix | Csy4 site | sgRNA-3

GAAACAGCATAGCAAGTTgAAATAAGGCTAGTCCGTTATCAACTTGAAAAAGTGGCACCGAGTCGGTGCGTTCACTGCCGTATAGGCAGTCGCTGGGACGCCCGGGG
CTTTGTCGTATCGTTCAAcTTTATTCCGATCAGGCAATAGTTGAACTTTTTCACCGTGGCTCAGCCACGCAAGTGACGGCATATCCGTCAGCGACCCTGCGGGCCCC

sgRNA-3 | Csy4 site | Suffix

ACTACACTTACGAAACTATTGATTGCTCAGCCTGCGGTCCGGGTTCACTGCCGTATAGGCAGCATCTTAGAGTATGTAGTTTcAGAGCTATGCTGGAAACAGCATAG
TGATGTGAATGCTTTGATAACTAACGAGTCGGACGCCAGGCCCAAGTGACGGCATATCCGTCGTAGAATCTCATACATCAAAgTCTCGATACGACCTTTGTCGTATC

Linker_1 | Prefix | Csy4 site | sgRNA-4t4

XhoI

CAAGTTgAAATAAGGCTAGTCCGTTATCAACTTGAAAAAGTGGCACCGAGTCGGTGCGTTCACTGCCGTATAGGCAGTCGCTGGGACGCCCGCTCGAGCAATAAACA
GTTCAAcTTTATTCCGATCAGGCAATAGTTGAACTTTTTCACCGTGGCTCAGCCACGCAAGTGACGGCATATCCGTCAGCGACCCTGCGGGCGAGCTCGTTATTTGT

sgRNA-4t4 | Csy4 site | Suffix

NcoI

GTTGATAGGGCTTCTCCGTTACCATGGTTCAGCCAAAAAACTTAAGACCGCCGGTCTTGTCCACTACCTTGCAGTAATGCGGTGGACAGGATCGGCGGTTTTCTTTT
CAACTATCCCGAAGAGGCAATGGTACCAAGTCGGTTTTTTGAATTCTGGCGGCCAGAACAGGTGATGGAACGTCATTACGCCACCTGTCCTAGCCGCCAAAAGAAAA

Linker_0 | ECK120029600 Terminator

CTCTTCTCAATTCTTCTGACCTGTAACGAATAATAGATAGTAAAGTAGTCTCCGATTGAGTTTTCTCTGCCGAGTCCCACCCAGTTCTGTGATTTCAGTAAGTTGGT
GAGAAGAGTTAAGAAGACTGGACATTGCTTATTATCTATCATTTCATCAGAGGCTAACTCAAAAGAGACGGCTCAGGGTGGGTCAAGACACTAAAGTCATTCAACCA

EC...r | Spacer 1

HindIII

AATTGATACACTGTTGCGAGAACTGCTGCCTGGTAGTAGATAGGTTGTTATTGAGTAAGAAGGTAAAGTGAACGAAATCCCTGAAACTGAGACTGTAGAAAATAAGC
TTAACTATGTGACAACGCTCTTGACGACGGACCATCATCTATCCAACAATAACTCATTCTTCCATTTCACTTGCTTTAGGGACTTTGACTCTGACATCTTTTATTCG

Spacer 1

TTCAGCCTGCGGTCCGGttgacggctagctcagtcctaggtacagtgctagcTCGCTGGGACGCCCGAGATAGCCGTTACACAGGTGACACTTATTTCAGCCTGCGG
AAGTCGGACGCCAGGCCaactgccgatcgagtcaggatccatgtcacgatcgAGCGACCCTGCGGGCTCTATCGGCAATGTGTCCACTGTGAATAAAGTCGGACGCC

Prefix | P(BBa_J23100) | Suffix | Linker_24 | Pr...x

pJ2048.2_Gs (5853 bp) (from 1178-2354 bp)

```
BamHI
TCCGGccaTACATACTCTAAGATGTGTCTCGCTGGGACGCCCGGGATCCAAGAGATTTCTACACGATTGAGCACTGTCTCAGCCTGCGGTCCGGGTTCACTGCCGTA
AGGCCggtATGTATGAGATTCTACACAGAGCGACCCTGCGGGCCCTAGGTTCTCTAAAGATGTGCTAACTCGTGACAGAGTCGGACGCCAGGCCCAAGTGACGGCAT
bs-4  Suffix  Linker_10  Prefix  Csy...te

TAGGCAGTAATTTTGTTTAACTTTAAGAAGGAGATATACATATGCGTAAAGGCGAAGAACTGTTTACCGGTGTGGTTCCGATTCTGGTGGAACTGGACGGCGATGTT
ATCCGTCATTAAAACAAATTGAAATTCTTCCTCTATATGTATACGCATTTCCGCTTCTTGACAAATGGCCACACCAAGGCTAAGACCACCTTGACCTGCCGCTACAA
RBS  sfGFP

AATGGTCATAAATTCAGTGTTCGCGGCGAAGGTGAAGGCGATGCGACGAACGGCAAACTGACCCTGAAATTTATCTGCACCACGGGTAAACTGCCGGTCCCGTGGCC
TTACCAGTATTTAAGTCACAAGCGCCGCTTCCACTTCCGCTACGCTGCTTGCCGTTTGACTGGGACTTTAAATAGACGTGGTGCCCATTTGACGGCCAGGGCACCGG
sfGFP

GACGCTGGTGACCACGCTGACCTATGGCGTTCAATGTTTTGCGCGTTACCCGGATCACATGAAACAGCACGACTTTTTCAAATCGGCCATGCCGGAAGGCTATGTGC
CTGCGACCACTGGTGCGACTGGATACCGCAAGTTACAAAACGCGCAATGGGCCTAGTGTACTTTGTCGTGCTGAAAAAGTTTAGCCGGTACGGCCTTCCGATACACG
sfGFP

AGGAACGTACGATTAGCTTTAAAGACGATGGTACGTATAAAACCCGCGCGGAAGTGAAATTCGAAGGCGATACCCTGGTTAACCGTATCGAACTGAAAGGTATCGAT
TCCTTGCATGCTAATCGAAATTTCTGCTACCATGCATATTTTGGGCGCGCCTTCACTTTAAGCTTCCGCTATGGGACCAATTGGCATAGCTTGACTTTCCATAGCTA
sfGFP

TTCAAAGAAGACGGCAATATTCTGGGTCATAAACTGGAATATAACTTCAATTCCCACAACGTGTACATCACCGCGGATAAACAGAAAAACGGCATTAAAGCCAATTT
AAGTTTCTTCTGCCGTTATAAGACCCAGTATTTGACCTTATATTGAAGTTAAGGGTGTTGCACATGTAGTGGCGCCTATTTGTCTTTTTGCCGTAATTTCGGTTAAA
sfGFP

CAAAATCCGCCATAATGTGGAAGATGGTAGCGTTCAGCTGGCCGACCACTATCAGCAAAACACGCCGATTGGTGATGGCCCGGTCCTGCTGCCGGACAATCACTACC
GTTTTAGGCGGTATTACACCTTCTACCATCGCAAGTCGACCGGCTGGTGATAGTCGTTTTGTGCGGCTAACCACTACCGGGCCAGGACGACGGCCTGTTAGTGATGG
sfGFP

TGAGTACCCAGTCCGTGCTGTCAAAAGATCCGAACGAAAAACGTGACCACATGGTCCTGCTGGAATTTGTGACGGCTGCGGGTATCACCCACGGCATGGACGAACTG
ACTCATGGGTCAGGCACGACAGTTTTCTAGGCTTGCTTTTTGCACTGGTGTACCAGGACGACCTTAAACACTGCCGACGCCCATAGTGGGTGCCGTACCTGCTTGAC
sfGFP

SbfI  KpnI
TATAAAATGTCCCGCCGTAATACTGACGCCATCACAATCCACAGCATCCTGGATTGGATTGAAGACTAATAATCGCTGGGACGCCCGCCTGCAGGCTCGGTACCAAA
ATATTTTACAGGGCGGCATTATGACTGCGGTAGTGTTAGGTGTCGTAGGACCTAACCTAACTTCTGATTATTAGCGACCCTGCGGGCGGACGTCCGAGCCATGGTTT
MarAn20  Suffix  L3S...or

TTCCAGAAAAGAGGCCTCCCGAAAGGGGGGCCTTTTTTCGTTTTGGTCCTAATAGATAAAGGATAGGTCTGGTAGTGTTGTTCGTTCTCGCAGGTAAATCAATAATA
AAGGTCTTTTCTCCGGAGGGCTTTCCCCCCGGAAAAAAGCAAAACCAGGATTATCTATTTCCTATCCAGACCATCACAACAAGCAAGAGCGTCCATTTAGTTATTAT
L3S2P21 Terminator  Spacer 2.5

CTCAGCAGTTCCGTAGACTTTTCAGTGGGACAGGGTAGCGATAACAGATAGATTGTAATAAGACACAGTAGGTGCTCGTAGTTGCGTGAAGAGAACCGCTCAGGAAA
GAGTCGTCAAGGCATCTGAAAAGTCACCCTGTCCCATCGCTATTGTCTATCTAACATTATTCTGTGTCATCCACGAGCATCAACGCACTTCTCTTGGCGAGTCCTTT
Spacer 2.5
```

pJ2048.2_Gs (5853 bp) (from 2355-3531 bp)

SalI
TCCAGTCAGAAGTATTGGTAATCGTTGAAAACTCAGTCGACCAGCCTGCGGTCCGGTTTACGGCTAGCTCAGTCCTAGGTATTATGCTAGCTCGCTGGGACGCCCGA
AGGTCAGTCTTCATAACCATTAGCAACTTTTGAGTCAGCTGGTCGGACGCCAGGCCAAATGCCGATCGAGTCAGGATCCATAATACGATCGAGCGACCCTGCGGGCT
Spacer 2.5 | Prefix | P(BBa_J23150) | Suffix

GTGACGACTGCGAAGTAACCTCTATTTATCAGCCTGCGGTCCGGccaAGAGTACACTAGCTCAGTCATCGCTGGGACGCCCGGTAGTGCTTATCAGACCCAATACTG
CACTGCTGACGCTTCATTGGAGATAAATAGTCGGACGCCAGGCCggtTCTCATGTGATCGAGTCAGTAGCGACCCTGCGGGCCATCACGAATAGTCTGGGTTATGAC
Linker_25 | Prefix | bs-3 | Suffix | Linker_17

TTGAACAGCCTGCGGTCCGGGTTCACTGCCGTATAGGCAGGACACATCTTAGAGTATGTAGTTTcAGAGCTATGCTGGAAACAGCATAGCAAGTTgAAATAAGGCTA
AACTTGTCGGACGCCAGGCCCAAGTGACGGCATATCCGTCCTGTGTAGAATCTCATACATCAAAgTCTCGATACGACCTTTGTCGTATCGTTCAACTTTATTCCGAT
Prefix | Csy4 site | sgRNA-4

AatII
GTCCGTTATCAACTTGAAAAAGTGGCACCGAGTCGGTGCGTTCACTGCCGTATAGGCAGTCGCTGGGACGCCCGGACGTCCTATTACACTCGTCGTTGGAAACTGAA
CAGGCAATAGTTGAACTTTTTCACCGTGGCTCAGCCACGCAAGTGACGGCATATCCGTCAGCGACCCTGCGGGCCTGCAGGATAATGTGAGCAGCAACCTTTGACTT
sgRNA-4 | Csy4 site | Suffix | Linker_11

NotI
GATGCGGCCGCggaaacacagAAAAAAGCCCGCACCTGACAGTGCGGGCTTTTTTTTTcgaccaaaggTAGCGAACGACGAGTCACTGTTGAGGATAAATACTTTCT
CTACGCCGGCGcctttgtgtcTTTTTTCGGGCGTGGACTGTCACGCCCGAAAAAAAAAgctggtttccATCGCTTGCTGCTCAGTGACAACTCCTATTTATGAAAGA
ECK120033737 Terminator | Linker_8

KasI | BbvCI | AscI
CTACTAGGCGCCTGTTACACAGGTCCTCAGCGGCGCGCCTTTGTCGGTGAACGCTCTCCTGAGTAGGACAAATCCGCCGGGAGCGGATTTGAACGTTGTGAAGCAAC
GATGATCCGCGGACAATGTGTCCAGGAGTCGCCGCGCGGAAACAGCCACTTGCGAGAGGACTCATCCTGTTTAGGCGGCCCTCGCCTAAACTTGCAACACTTCGTTG
Spa...15

GGCCCGGAGGGTGGCGGGCAGGACGCCCGCCATAAACTGCCAGGCATCAAACTAAGCAGAAGGCCATCCTGACGGATGGCCTTTTTGCGTTTCAGATCTgggatttt
CCGGGCCTCCCACCGCCCGTCCTGCGGGCGGTATTTGACGGTCCGTAGTTTGATTCGTCTTCCGGTAGGACTGCCTACCGGAAAAACGCAAAGTCTAGAccctaaaa

ggtcatgagattatcaaaaaggatcttcacctagatccttttaaattaaaaatgaagttttaaatcaatctaaagtatatatgagtaaacttggtctgacagttacc
ccagtactctaatagtttttcctagaagtggatctaggaaaatttaatttttacttcaaaatttagttagatttcatatatactcatttgaaccagactgtcaatgg

aatgcttaatcagtgaggcacctatctcagcgatctgtctatttcgttcatccatagttgcctgactccccgtcgtgtagataactacgatacgggagggcttacca
ttacgaattagtcactccgtggatagagtcgctagacagataaagcaagtaggtatcaacggactgaggggcagcacatctattgatgctatgccctcccgaatggt
AmpR

tctggccccagtgctgcaatgataccgcgagacccacgctcaccggctccagatttatcagcaataaaccagccagccggaagggccgagcgcagaagtggtcctgc
agaccggggtcacgacgttactatggcgctctgggtgcgagtggccgaggtctaaatagtcgttatttggtcggtcggccttcccggctcgcgtcttcaccaggacg
AmpR

aactttatccgcctccatccagtctattaattgttgccgggaagctagagtaagtagttcgccagttaatagtttgcgcaacgttgttgccattgctgcaggcatcg
ttgaaataggcggaggtaggtcagataattaacaacggcccttcgatctcattcatcaagcggtcaattatcaaacgcgttgcaacaacggtaacgacgtccgtagc
AmpR

pJ2048.2_Gs (5853 bp) (from 3532-4815 bp)

```
tggtgtcacgctcgtcgtttggtatggcttcattcagctccggttcccaacgatcaaggcgagttacatgatcccccatgttgtgcaaaaaagcggttagctccttc
accacagtgcgagcagcaaaccataccgaagtaagtcgaggccaagggttgctagttccgctcaatgtactagggggtacaacacgttttttcgccaatcgaggaag
                                         AmpR

ggtcctccgatcgttgtcagaagtaagttggccgcagtgttatcactcatggttatggcagcactgcataattctcttactgtcatgccatccgtaagatgcttttc
ccaggaggctagcaacagtcttcattcaaccggcgtcacaatagtgagtaccaataccgtcgtgacgtattaagagaatgacagtacggtaggcattctacgaaaag
                                         AmpR

tgtgactggtgagtactcaaccaagtcattctgagaatagtgtatgcggcgaccgagttgctcttgcccggcgtcaacacgggataataccgcgccacatagcagaa
acactgaccactcatgagttggttcagtaagactcttatcacatacgccgctggctcaacgagaacgggccgcagttgtgccctattatggcgcggtgtatcgtctt
                                         AmpR

ctttaaaagtgctcatcattggaaaacgttcttcggggcgaaaactctcaaggatcttaccgctgttgagatccagttcgatgtaacccactcgtgcacccaactga
gaaattttcacgagtagtaaccttttgcaagaagccccgcttttgagagttcctagaatggcgacaactctaggtcaagctacattgggtgagcacgtgggttgact
                                         AmpR

tcttcagcatcttttactttcaccagcgtttctgggtgagcaaaaacaggaaggcaaaatgccgcaaaaaagggaataagggcgacacggaaatgttgaatactcat
agaagtcgtagaaaatgaaagtggtcgcaaagacccactcgtttttgtccttccgttttacggcgttttttcccttattcccgctgtgcctttacaacttatgagta
                                         AmpR

actcttcctttttcaatattattgaagcatttatcagggttattgtctcatgagcggatacatatttgaatgtatttagaaaaataaacaaataggggttccgcgca
tgagaaggaaaaagttataataacttcgtaaatagtcccaataacagagtactcgcctatgtataaacttacataaatctttttatttgtttatccccaaggcgcgt

gcatgctagcGgcagaaacgtcctagaagatgccaggaggatacttagcagagagacaataaggccggagcgaagccgtttttccataggctccgcccccctgacga
cgtacgatcgCcgtctttgcaggatcttctacggtcctcctatgaatcgtctctctgttattccggcctcgcttcggcaaaaaggtatccgaggcggggggactgct
                                              ColA ori

acatcacgaaatctgacgctcaaatcagtggtggcgaaacccgacaggactataaagataccaggcgtttccccctgatggctccctcttgcgctctcctgttcccg
tgtagtgctttagactgcgagtttagtcaccaccgctttgggctgtcctgatatttctatggtccgcaaagggggactaccgagggagaacgcgagaggacaagggc
                                         ColA ori

tcctgcggcgtccgtgttgtggtggaggctttacccaaatcaccacgtcccgttccgtgtagacagttcgctccaagctgggctgtgtgcaagaaccccccgttcag
aggacgccgcaggcacaacaccacctccgaaatgggtttagtggtgcagggcaaggcacatctgtcaagcgaggttcgacccgacacacgttcttggggggcaagtc
                                         ColA ori

cccgactgctgcgccttatccggtaactatcatcttgagtccaacccggaaagacacgacaaaacgccactggcagcagccattggtaactgagaattagtggattt
gggctgacgacgcggaataggccattgatagtagaactcaggttgggcctttctgtgctgttttgcggtgaccgtcgtcggtaaccattgactcttaatcacctaaa
                                         ColA ori

agatatcgagagtcttgaagtggtggcctaacagaggctacactgaaaggacagtatttggtatctgcgctccactaaagccagttaccaggttaagcagttcccca
tctatagctctcagaacttcaccaccggattgtctccgatgtgactttcctgtcataaaccatagacgcgaggtgatttcggtcaatggtccaattcgtcaaggggt
                                         ColA ori

actgacttaaccttcgatcaaaccgcctccccaggcggtttttttcgtttacagagcaggagattacgacgatcgtaaaaggatctcaagaagatcctttacggattc
tgactgaattggaagctagtttggcggaggggtccgccaaaaaaagcaaatgtctcgtcctctaatgctgctagcattttcctagagttcttctaggaaatgcctaag
                                         ColA ori
```

pJ2048.2_Gs (5853 bp) (from 4816-5853 bp)

ccgacaccatcactctagatttcagtgcaatttatctcttcaaatgtagcacctgaagtcagccccatacgatataagttgtaattctcatgttagtcatgccccgc
ggctgtggtagtgagatctaaagtcacgttaaatagagaagtttacatcgtggacttcagtcggggtatgctatattcaacattaagagtacaatcagtacggggcg

gcccaccggaaggagctgactgggttgCTCCTAgGGTCTGATTCGTTACCAATTATGACAACTTGACGGCTACATCATTCACTTTTTCTTCACAACCGGCACGGAAC
cgggtggccttcctcgactgacccaacGAGGATcCCAGACTAAGCAATGGTTAATACTGTTGAACTGCCGATGTAGTAAGTGAAAAAGAAGTGTTGGCCGTGCCTTG
araC

TCGCTCGGGCTGGCCCCGGTGCATTTTTTAAATACCCGCGAGAAATAGAGTTGATCGTCAAAACCAACATTGCGACCGACGGTGGCGATAGGCATCCGGGTGGTGCT
AGCGAGCCCGACCGGGGCCACGTAAAAAATTTATGGGCGCTCTTTATCTCAACTAGCAGTTTTGGTTGTAACGCTGGCTGCCACCGCTATCCGTAGGCCCACCACGA
araC

CAAAAGCAGCTTCGCCTGGCTGATACGTTGGTCCTCGCGCCAGCTTAAGACGCTAATCCCTAACTGCTGGCGGAAAAGATGTGACAGACGCGACGGCGACAAGCAAA
GTTTTCGTCGAAGCGGACCGACTATGCAACCAGGAGCGCGGTCGAATTCTGCGATTAGGGATTGACGACCGCCTTTTCTACACTGTCTGCGCTGCCGCTGTTCGTTT
araC

CATGCTGTGCGACGCTGGCGATATCAAAATTGCTGTCTGCCAGGTGATCGCTGATGTACTGACAAGCCTCGCGTACCCGATTATCCATCGGTGGATGGAGCGACTCG
GTACGACACGCTGCGACCGCTATAGTTTTAACGACAGACGGTCCACTAGCGACTACATGACTGTTCGGAGCGCATGGGCTAATAGGTAGCCACCTACCTCGCTGAGC
araC

TTAATCGCTTCCATGCGCCGCAGTAACAATTGCTCAAGCAGATTTATCGCCAGCAGCTCCGAATAGCGCCCTTCCCCTTGCCCGGCGTTAATGATTTGCCCAAACAG
AATTAGCGAAGGTACGCGGCGTCATTGTTAACGAGTTCGTCTAAATAGCGGTCGTCGAGGCTTATCGCGGGAAGGGGAACGGGCCGCAATTACTAAACGGGTTTGTC
araC

GTCGCTGAAATGCGGCTGGTGCGCTTCATCCGGGCGAAAGAACCCCGTATTGGCAAATATTGACGGCCAGTTAAGCCATTCATGCCAGTAGGCGCGCGGACGAAAGT
CAGCGACTTTACGCCGACCACGCGAAGTAGGCCCGCTTTCTTGGGGCATAACCGTTTATAACTGCCGGTCAATTCGGTAAGTACGGTCATCCGCGCGCCTGCTTTCA
araC

AAACCCACTGGTGATACCATTCGCGAGCCTCCGGATGACGACCGTAGTGATGAATCTCTCCTGGCGGGAACAGCAAAATATCACCCGGTCGGCAAACAAATTCTCGT
TTTGGGTGACCACTATGGTAAGCGCTCGGAGGCCTACTGCTGGCATCACTACTTAGAGAGGACCGCCCTTGTCGTTTTATAGTGGGCCAGCCGTTTGTTTAAGAGCA
araC

CCCTGATTTTTCACCACCCCCTGACCGCGAATGGTGAGATTGAGAATATAACCTTTCATTCCCAGCGGTCGGTCGATAAAAAAATCGAGATAACCGTTGGCCTCAAT
GGGACTAAAAAGTGGTGGGGGACTGGCGCTTACCACTCTAACTCTTATATTGGAAAGTAAGGGTCGCCAGCCAGCTATTTTTTTAGCTCTATTGGCAACCGGAGTTA
araC

CGGCGTTAAACCCGCCACCAGATGGGCATTAAACGAGTATCCCGGCAGCAGGGGATCATTTTGCGCTTCAGCCAT
GCCGCAATTTGGGCGGTGGTCTACCCGTAATTTGCTCATAGGGCCGTCGTCCCCTAGTAAAACGCGAAGTCGGTA
araC

(from 1-1070 bp)

pJ2048_2xNOT (5651 bp)

ACTTTTCATACTCCCGCCATTCAGAGAAGAAACCAATTGTCCATATTGCATCAGACATTGCCGTCACTGCGTCTTTTACTGGCTCTTCTCGCTAACCAAACCGGTAA
TGAAAAGTATGAGGGCGGTAAGTCTCTTCTTTGGTTAACAGGTATAACGTAGTCTGTAACGGCAGTGACGCAGAAAATGACCGAGAAGAGCGATTGGTTTGGCCATT

CCCCGCTTATTAAAAGCATTCTGTAACAAAGCGGGACCAAAGCCATGACAAAAACGCGTAACAAAAGTGTCTATAATCACGGCAGAAAAGTCCACATTGATTATTTG
GGGGCGAATAATTTTCGTAAGACATTGTTTCGCCCTGGTTTCGGTACTGTTTTTGCGCATTGTTTTCACAGATATTAGTGCCGTCTTTTCAGGTGTAACTAATAAAC

EcoRI
CACGGCGTCACACTTTGCTATGCCATAGCATTTTTATCCATAAGATTAGCGGtTCCTACCTGACGCTTTTTATCGCAACTCTCTACTGTTTCTCCATACCGAATTCA
GTGCCGCAGTGTGAAACGATACGGTATCGTAAAAATAGGTATTCTAATCGCCaAGGATGGACTGCGAAAAATAGCGTTGAGAGATGACAAAGAGGTATGGCTTAAGT
P(BAD) promoter

SacI
TAGGATAGATTCTGGAAACTTTACCGTCCGAGCTCCAGCCTGCGGTCCGGGTTCACTGCCGTATAGGCAGTGACTGAGCTAGTGTACTCTGTTTcAGAGCTATGCTG
ATCCTATCTAAGACCTTTGAAATGGCAGGCTCGAGGTCGGACGCCAGGCCCAAGTGACGGCATATCCGTCACTGACTCGATCACATGAGACAAAgTCTCGATACGAC
Linker_14 Prefix Csy4 site sgRNA-3

XhoI
GAAACAGCATAGCAAGTTgAAATAAGGCTAGTCCGTTATCAACTTGAAAAAGTGGCACCGAGTCGGTGCGTTCACTGCCGTATAGGCAGTCGCTGGGACGCCCGCTC
CTTTGTCGTATCGTTCAAcTTTATTCCGATCAGGCAATAGTTGAACTTTTTCACCGTGGCTCAGCCACGCAAGTGACGGCATATCCGTCAGCGACCCTGCGGGCGAG
sgRNA-3 Csy4 site Suffix

NcoI
GAGCAATAAACAGTTGATAGGGCTTCTCCGTTACCATGGTTCAGCCAAAAAACTTAAGACCGCCGGTCTTGTCCACTACCTTGCAGTAATGCGGTGGACAGGATCGG
CTCGTTATTTGTCAACTATCCCGAAGAGGCAATGGTACCAAGTCGGTTTTTTGAATTCTGGCGGCCAGAACAGGTGATGGAACGTCATTACGCCACCTGTCCTAGCC
Linker_0 ECK120029600 Terminator

CGGTTTTCTTTTCTCTTCTCAATTCTTCTGACCTGTAACGAATAATAGATAGTAAAGTAGTCTCCGATTGAGTTTTCTCTGCCGAGTCCCACCCAGTTCTGTGATTT
GCCAAAAGAAAAGAGAAGAGTTAAGAAGACTGGACATTGCTTATTATCTATCATTTCATCAGAGGCTAACTCAAAAGAGACGGCTCAGGGTGGGTCAAGACACTAAA
ECK12002...minator Spacer 1

CAGTAAGTTGGTAATTGATACACTGTTGCGAGAACTGCTGCCTGGTAGTAGATAGGTTGTTATTGAGTAAGAAGGTAAAGTGAACGAAATCCCTGAAACTGAGACTG
GTCATTCAACCATTAACTATGTGACAACGCTCTTGACGACGGACCATCATCTATCCAACAATAACTCATTCTTCCATTTCACTTGCTTTAGGGACTTTGACTCTGAC
Spacer 1

HindIII
TAGAAAATAAGCTTCAGCCTGCGGTCCGGttgacggctagctcagtcctaggtacagtgctagcTCGCTGGGACGCCCGAGATAGCCGTTACACAGGTGACACTTAT
ATCTTTTATTCGAAGTCGGACGCCAGGCCaactgccgatcgagtcaggatccatgtcacgatcgAGCGACCCTGCGGGCTCTATCGGCAATGTGTCCACTGTGAATA
Prefix P(BBa_J23100) Suffix Linker_24

BamHI
TTCAGCCTGCGGTCCGGccaTACATACTCTAAGATGTGTCTCGCTGGGACGCCCGGGATCCAAGAGATTTCTACACGATTGAGCACTGTCTCAGCCTGCGGTCCGGG
AAGTCGGACGCCAGGCCggtATGTATGAGATTCTACACAGAGCGACCCTGCGGGCCCTAGGTTCTCTAAAGATGTGCTAACTCGTGACAGAGTCGGACGCCAGGCCC
Prefix bs-4 Suffix Linker_10 Prefix

pJ2048_2xNOT (5651 bp) (from 1071-2247 bp)

TTCACTGCCGTATAGGCAGTAATTTTGTTTAACTTTAAGAAGGAGATATACATATGCGTAAAGGCGAAGAACTGTTTACCGGTGTGGTTCCGATTCTGGTGGAACTG
AAGTGACGGCATATCCGTCATTAAAACAAATTGAAATTCTTCCTCTATATGTATACGCATTTCCGCTTCTTGACAAATGGCCACACCAAGGCTAAGACCACCTTGAC
Csy4 site | RBS | sfGFP

GACGGCGATGTTAATGGTCATAAATTCAGTGTTCGCGGCGAAGGTGAAGGCGATGCGACGAACGGCAAACTGACCCTGAAATTTATCTGCACCACGGGTAAACTGCC
CTGCCGCTACAATTACCAGTATTTAAGTCACAAGCGCCGCTTCCACTTCCGCTACGCTGCTTGCCGTTTGACTGGGACTTTAAATAGACGTGGTGCCCATTTGACGG
sfGFP

GGTCCCGTGGCCGACGCTGGTGACCACGCTGACCTATGGCGTTCAATGTTTTGCGCGTTACCCGGATCACATGAAACAGCACGACTTTTTCAAATCGGCCATGCCGG
CCAGGGCACCGGCTGCGACCACTGGTGCGACTGGATACCGCAAGTTACAAAACGCGCAATGGGCCTAGTGTACTTTGTCGTGCTGAAAAAGTTTAGCCGGTACGGCC
sfGFP

AAGGCTATGTGCAGGAACGTACGATTAGCTTTAAAGACGATGGTACGTATAAAACCCGCGCGGAAGTGAAATTCGAAGGCGATACCCTGGTTAACCGTATCGAACTG
TTCCGATACACGTCCTTGCATGCTAATCGAAATTTCTGCTACCATGCATATTTTGGGCGCGCCTTCACTTTAAGCTTCCGCTATGGGACCAATTGGCATAGCTTGAC
sfGFP

AAAGGTATCGATTTCAAAGAAGACGGCAATATTCTGGGTCATAAACTGGAATATAACTTCAATTCCCACAACGTGTACATCACCGCGGATAAACAGAAAAACGGCAT
TTTCCATAGCTAAAGTTTCTTCTGCCGTTATAAGACCCAGTATTTGACCTTATATTGAAGTTAAGGGTGTTGCACATGTAGTGGCGCCTATTTGTCTTTTTGCCGTA
sfGFP

TAAAGCCAATTTCAAAATCCGCCATAATGTGGAAGATGGTAGCGTTCAGCTGGCCGACCACTATCAGCAAAACACGCCGATTGGTGATGGCCCGGTCCTGCTGCCGG
ATTTCGGTTAAAGTTTTAGGCGGTATTACACCTTCTACCATCGCAAGTCGACCGGCTGGTGATAGTCGTTTTGTGCGGCTAACCACTACCGGGCCAGGACGACGGCC
sfGFP

ACAATCACTACCTGAGTACCCAGTCCGTGCTGTCAAAAGATCCGAACGAAAAACGTGACCACATGGTCCTGCTGGAATTTGTGACGGCTGCGGGTATCACCCACGGC
TGTTAGTGATGGACTCATGGGTCAGGCACGACAGTTTTCTAGGCTTGCTTTTTGCACTGGTGTACCAGGACGACCTTAAACACTGCCGACGCCCATAGTGGGTGCCG
sfGFP

SbfI
ATGGACGAACTGTATAAAATGTCCCGCCGTAATACTGACGCCATCACAATCCACAGCATCCTGGATTGGATTGAAGACTAATAATCGCTGGGACGCCCGCCTGCAGG
TACCTGCTTGACATATTTTACAGGGCGGCATTATGACTGCGGTAGTGTTAGGTGTCGTAGGACCTAACCTAACTTCTGATTATTAGCGACCCTGCGGGCGGACGTCC
sfGFP | MarAn20 | Suffix

KpnI
CTCGGTACCAAATTCCAGAAAAGAGGCCTCCCGAAAGGGGGGCCTTTTTTCGTTTTGGTCCTAATAGATAAAGGATAGGTCTGGTAGTGTTGTTCGTTCTCGCAGGT
GAGCCATGGTTTAAGGTCTTTTCTCCGGAGGGCTTTCCCCCCGGAAAAAAGCAAAACCAGGATTATCTATTTCCTATCCAGACCATCACAACAAGCAAGAGCGTCCA
L3S2P21 Terminator | Spacer 2.5

AAATCAATAATACTCAGCAGTTCCGTAGACTTTTCAGTGGGACAGGGTAGCGATAACAGATAGATTGTAATAAGACACAGTAGGTGCTCGTAGTTGCGTGAAGAGAA
TTTAGTTATTATGAGTCGTCAAGGCATCTGAAAAGTCACCCTGTCCCATCGCTATTGTCTATCTAACATTATTCTGTGTCATCCACGAGCATCAACGCACTTCTCTT
Spacer 2.5

SalI
CCGCTCAGGAAATCCAGTCAGAAGTATTGGTAATCGTTGAAAACTCAGTCGACCAGCCTGCGGTCCGGTTTACGGCTAGCTCAGTCCTAGGTATTATGCTAGCTCGC
GGCGAGTCCTTTAGGTCAGTCTTCATAACCATTAGCAACTTTTGAGTCAGCTGGTCGGACGCCAGGCCAAATGCCGATCGAGTCAGGATCCATAATACGATCGAGCG
Spacer 2.5 | Prefix | P(BBa_J23150)

pJ2048_2xNOT (5651 bp) (from 2248-3531 bp)

TGGGACGCCCGAGTGACGACTGCGAAGTAACCTCTATTTATCAGCCTGCGGTCCGGccaAGAGTACACTAGCTCAGTCATCGCTGGGACGCCCGGTAGTGCTTATCA
ACCCTGCGGGCTCACTGCTGACGCTTCATTGGAGATAAATAGTCGGACGCCAGGCCggtTCTCATGTGATCGAGTCAGTAGCGACCCTGCGGGCCATCACGAATAGT
Suffix Linker_25 Prefix bs-3 Suffix Lin...17

GACCCAATACTGTTGAACAGCCTGCGGTCCGGGTTCACTGCCGTATAGGCAGGACACATCTTAGAGTATGTAGTTTcAGAGCTATGCTGGAAACAGCATAGCAAGTT
CTGGGTTATGACAACTTGTCGGACGCCAGGCCCAAGTGACGGCATATCCGTCCTGTGTAGAATCTCATACATCAAAgTCTCGATACGACCTTTGTCGTATCGTTCAA
Linker_17 Prefix Csy4 site sgRNA-4

AatII
gAAATAAGGCTAGTCCGTTATCAACTTGAAAAAGTGGCACCGAGTCGGTGCGTTCACTGCCGTATAGGCAGTCGCTGGGACGCCCGGACGTCCTATTACACTCGTCG
cTTTATTCCGATCAGGCAATAGTTGAACTTTTTCACCGTGGCTCAGCCACGCAAGTGACGGCATATCCGTCAGCGACCCTGCGGGCCTGCAGGATAATGTGAGCAGC
sgRNA-4 Csy4 site Suffix Linker_11

NotI
TTGGAAACTGAAGATGCGGCCGCggaaacacagAAAAAAGCCCGCACCTGACAGTGCGGGCTTTTTTTTTcgaccaaaggTAGCGAACGACGAGTCACTGTTGAGGA
AACCTTTGACTTCTACGCCGGCGcctttgtgtcTTTTTTCGGGCGTGGACTGTCACGCCCGAAAAAAAAAgctggtttccATCGCTTGCTGCTCAGTGACAACTCCT
Linker_11 ECK120033737 Terminator Linker_8

KasI BbvCI AscI
TAAATACTTTCTCTACTAGGCGCCTGTTACACAGGTCCTCAGCGGCGCGCCTTTGTCGGTGAACGCTCTCCTGAGTAGGACAAATCCGCCGGGAGCGGATTTGAACG
ATTTATGAAAGAGATGATCCGCGGACAATGTGTCCAGGAGTCGCCGCGCGGAAACAGCCACTTGCGAGAGGACTCATCCTGTTTAGGCGGCCCTCGCCTAAACTTGC
Linker_8 Spa...15

TTGTGAAGCAACGGCCCGGAGGGTGGCGGGCAGGACGCCCGCCATAAACTGCCAGGCATCAAACTAAGCAGAAGGCCATCCTGACGGATGGCCTTTTTGCGTTTCAG
AACACTTCGTTGCCGGGCCTCCCACCGCCCGTCCTGCGGGCGGTATTTGACGGTCCGTAGTTTGATTCGTCTTCCGGTAGGACTGCCTACCGGAAAAACGCAAAGTC

ATCTgggattttggtcatgagattatcaaaaaggatcttcacctagatccttttaaattaaaaatgaagttttaaatcaatctaaagtatatatgagtaaacttggt
TAGAccctaaaaccagtactctaatagtttttcctagaagtggatctaggaaaatttaatttttacttcaaaatttagttagatttcatatatactcatttgaacca

ctgacagttaccaatgcttaatcagtgaggcacctatctcagcgatctgtctatttcgttcatccatagttgcctgactccccgtcgtgtagataactacgatacgg
gactgtcaatggttacgaattagtcactccgtggatagagtcgctagacagataaagcaagtaggtatcaacggactgaggggcagcacatctattgatgctatgcc
AmpR

gagggcttaccatctggccccagtgctgcaatgataccgcgagacccacgctcaccggctccagatttatcagcaataaaccagccagccggaagggccgagcgcag
ctcccgaatggtagaccggggtcacgacgttactatggcgctctgggtgcgagtggccgaggtctaaatagtcgttatttggtcggtcggccttcccggctcgcgtc
AmpR

aagtggtcctgcaactttatccgcctccatccagtctattaattgttgccgggaagctagagtaagtagttcgccagttaatagtttgcgcaacgttgttgccattg
ttcaccaggacgttgaaataggcggaggtaggtcagataattaacaacggcccttcgatctcattcatcaagcggtcaattatcaaacgcgttgcaacaacggtaac
AmpR

ctgcaggcatcgtggtgtcacgctcgtcgtttggtatggcttcattcagctccggttcccaacgatcaaggcgagttacatgatcccccatgttgtgcaaaaaagcg
gacgtccgtagcaccacagtgcgagcagcaaaccataccgaagtaagtcgaggccaagggttgctagttccgctcaatgtactagggggtacaacacgttttttcgc
AmpR

gttagctccttcggtcctccgatcgttgtcagaagtaagttggccgcagtgttatcactcatggttatggcagcactgcataattctcttactgtcatgccatccgt
caatcgaggaagccaggaggctagcaacagtcttcattcaaccggcgtcacaatagtgagtaccaataccgtcgtgacgtattaagagaatgacagtacggtaggca
AmpR

pJ2048_2xNOT (5651 bp) (from 3532-4815 bp)

aagatgcttttctgtgactggtgagtactcaaccaagtcattctgagaatagtgtatgcggcgaccgagttgctcttgcccggcgtcaacacgggataataccgcgc
ttctacgaaaagacactgaccactcatgagttggttcagtaagactcttatcacatacgccgctggctcaacgagaacgggccgcagttgtgccctattatggcgcg
AmpR

cacatagcagaactttaaaagtgctcatcattggaaaacgttcttcggggcgaaaactctcaaggatcttaccgctgttgagatccagttcgatgtaacccactcgt
gtgtatcgtcttgaaattttcacgagtagtaaccttttgcaagaagccccgcttttgagagttcctagaatggcgacaactctaggtcaagctacattgggtgagca
AmpR

gcacccaactgatcttcagcatcttttactttcaccagcgtttctgggtgagcaaaaacaggaaggcaaaatgccgcaaaaaagggaataagggcgacacggaaatg
cgtgggttgactagaagtcgtagaaaatgaaagtggtcgcaaagacccactcgtttttgtccttccgttttacggcgttttttcccttattcccgctgtgcctttac
AmpR

ttgaatactcatactcttcctttttcaatattattgaagcatttatcagggttattgtctcatgagcggatacatatttgaatgtatttagaaaaataaacaaatag
aacttatgagtatgagaaggaaaaagttataataacttcgtaaatagtcccaataacagagtactcgcctatgtataaacttacataaatctttttatttgtttatc
AmpR

gggttccgcgcagcatgctagcGgcagaaacgtcctagaagatgccaggaggatacttagcagagagacaataaggccggagcgaagccgtttttccataggctccg
cccaaggcgcgtcgtacgatcgCcgtctttgcaggatcttctacggtcctcctatgaatcgtctctctgttattccggcctcgcttcggcaaaaaggtatccgaggc
ColA ori

cccccctgacgaacatcacgaaatctgacgctcaaatcagtggtggcgaaacccgacaggactataaagataccaggcgtttccccctgatggctccctcttgcgct
ggggggactgcttgtagtgctttagactgcgagtttagtcaccaccgctttgggctgtcctgatatttctatggtccgcaaagggggactaccgagggagaacgcga
ColA ori

ctcctgttcccgtcctgcggcgtccgtgttgtggtggaggctttacccaaatcaccacgtcccgttccgtgtagacagttcgctccaagctgggctgtgtgcaagaa
gaggacaagggcaggacgccgcaggcacaacaccacctccgaaatgggtttagtggtgcagggcaaggcacatctgtcaagcgaggttcgacccgacacacgttctt
ColA ori

ccccccgttcagcccgactgctgcgccttatccggtaactatcatcttgagtccaacccggaaagacacgacaaaacgccactggcagcagccattggtaactgaga
ggggggcaagtcgggctgacgacgcggaataggccattgatagtagaactcaggttgggcctttctgtgctgttttgcggtgaccgtcgtcggtaaccattgactct
ColA ori

attagtggatttagatatcgagagtcttgaagtggtggcctaacagaggctacactgaaaggacagtatttggtatctgcgctccactaaagccagttaccaggtta
taatcacctaaatctatagctctcagaacttcaccaccggattgtctccgatgtgactttcctgtcataaaccatagacgcgaggtgatttcggtcaatggtccaat
ColA ori

agcagttccccaactgacttaaccttcgatcaaaccgcctccccaggcggttttttcgtttacagagcaggagattacgacgatcgtaaaaggatctcaagaagatc
tcgtcaaggggttgactgaattggaagctagtttggcggaggggtccgccaaaaaagcaaatgtctcgtcctctaatgctgctagcattttcctagagttcttctag
ColA ori

ctttacggattcccgacaccatcactctagatttcagtgcaatttatctcttcaaatgtagcacctgaagtcagccccatacgatataagttgtaattctcatgtta
gaaatgcctaagggctgtggtagtgagatctaaagtcacgttaaatagagaagtttacatcgtggacttcagtcggggtatgctatattcaacattaagagtacaat
ColA ori

gtcatgccccgcgcccaccggaaggagctgactgggttgCTCCTAgGGTCTGATTCGTTACCAATTATGACAACTTGACGGCTACATCATTCACTTTTTCTTCACAA
cagtacggggcgcgggtggccttcctcgactgacccaacGAGGATcCCAGACTAAGCAATGGTTAATACTGTTGAACTGCCGATGTAGTAAGTGAAAAAGAAGTGTT
araC

pJ2048_2xNOT (5651 bp) (from 4816-5651 bp)

CCGGCACGGAACTCGCTCGGGCTGGCCCCGGTGCATTTTTTAAATACCCGCGAGAAATAGAGTTGATCGTCAAAACCAACATTGCGACCGACGGTGGCGATAGGCAT
GGCCGTGCCTTGAGCGAGCCCGACCGGGGCCACGTAAAAAATTTATGGGCGCTCTTTATCTCAACTAGCAGTTTTGGTTGTAACGCTGGCTGCCACCGCTATCCGTA
araC

CCGGGTGGTGCTCAAAAGCAGCTTCGCCTGGCTGATACGTTGGTCCTCGCGCCAGCTTAAGACGCTAATCCCTAACTGCTGGCGGAAAAGATGTGACAGACGCGACG
GGCCCACCACGAGTTTTCGTCGAAGCGGACCGACTATGCAACCAGGAGCGCGGTCGAATTCTGCGATTAGGGATTGACGACCGCCTTTTCTACACTGTCTGCGCTGC
araC

GCGACAAGCAAACATGCTGTGCGACGCTGGCGATATCAAAATTGCTGTCTGCCAGGTGATCGCTGATGTACTGACAAGCCTCGCGTACCCGATTATCCATCGGTGGA
CGCTGTTCGTTTGTACGACACGCTGCGACCGCTATAGTTTTAACGACAGACGGTCCACTAGCGACTACATGACTGTTCGGAGCGCATGGGCTAATAGGTAGCCACCT
araC

TGGAGCGACTCGTTAATCGCTTCCATGCGCCGCAGTAACAATTGCTCAAGCAGATTTATCGCCAGCAGCTCCGAATAGCGCCCTTCCCCTTGCCCGGCGTTAATGAT
ACCTCGCTGAGCAATTAGCGAAGGTACGCGGCGTCATTGTTAACGAGTTCGTCTAAATAGCGGTCGTCGAGGCTTATCGCGGGAAGGGGAACGGGCCGCAATTACTA
araC

TTGCCCAAACAGGTCGCTGAAATGCGGCTGGTGCGCTTCATCCGGGCGAAAGAACCCCGTATTGGCAAATATTGACGGCCAGTTAAGCCATTCATGCCAGTAGGCGC
AACGGGTTTGTCCAGCGACTTTACGCCGACCACGCGAAGTAGGCCCGCTTTCTTGGGGCATAACCGTTTATAACTGCCGGTCAATTCGGTAAGTACGGTCATCCGCG
araC

GCGGACGAAAGTAAACCCACTGGTGATACCATTCGCGAGCCTCCGGATGACGACCGTAGTGATGAATCTCTCCTGGCGGGAACAGCAAAATATCACCCGGTCGGCAA
CGCCTGCTTTCATTTGGGTGACCACTATGGTAAGCGCTCGGAGGCCTACTGCTGGCATCACTACTTAGAGAGGACCGCCCTTGTCGTTTTATAGTGGGCCAGCCGTT
araC

ACAAATTCTCGTCCCTGATTTTTCACCACCCCCTGACCGCGAATGGTGAGATTGAGAATATAACCTTTCATTCCCAGCGGTCGGTCGATAAAAAAATCGAGATAACC
TGTTTAAGAGCAGGGACTAAAAAGTGGTGGGGGACTGGCGCTTACCACTCTAACTCTTATATTGGAAAGTAAGGGTCGCCAGCCAGCTATTTTTTTAGCTCTATTGG
araC

GTTGGCCTCAATCGGCGTTAAACCCGCCACCAGATGGGCATTAAACGAGTATCCCGGCAGCAGGGGATCATTTTGCGCTTCAGCCAT
CAACCGGAGTTAGCCGCAATTTGGGCGGTGGTCTACCCGTAATTTGCTCATAGGGCCGTCGTCCCCTAGTAAAACGCGAAGTCGGTA
araC

(from 1-1177 bp)

pJ2072.2_c1 (6900 bp)

EcoRI SacI

GAATTCATAGGATAGATTCTGGAAACTTTACCGTCCGAGCTCCAGCCTGCGGTCCGGttgacggctagctcagtcctaggtacagtgctagcTCGCTGGGACGCCCG
CTTAAGTATCCTATCTAAGACCTTTGAAATGGCAGGCTCGAGGTCGGACGCCAGGCCaactgccgatcgagtcaggatccatgtcacgatcgAGCGACCCTGCGGGC

Linker_14 Prefix P(BBa_J23100) Suffix

AGAGCCGAATCGCACTTATTTACAGTAGTTCAGCCTGCGGTCCGGccaTACATACTCTAAGATGTGTCTCGCTGGGACGCCCGGGGACTACACTTACGAAACTATTG
TCTCGGCTTAGCGTGAATAAATGTCATCAAGTCGGACGCCAGGCCggtATGTATGAGATTCTACACAGAGCGACCCTGCGGGCCCCTGATGTGAATGCTTTGATAAC

Linker_21 Prefix bs-4 Suffix Linker_1

ATTGCTCAGCCTGCGGTCCGGGTTCACTGCCGTATAGGCAGATCAGTGTGTACTAAGTACTGTTTcAGAGCTATGCTGGAAACAGCATAGCAAGTTgAAATAAGGCT
TAACGAGTCGGACGCCAGGCCCAAGTGACGGCATATCCGTCTAGTCACACATGATTCATGACAAAgTCTCGATACGACCTTTGTCGTATCGTTCAAcTTTATTCCGA

Prefix Csy4 site sgRNA-2

XhoI

AGTCCGTTATCAACTTGAAAAAGTGGCACCGAGTCGGTGCGTTCACTGCCGTATAGGCAGTCGCTGGGACGCCCGCTCGAGCAATAAACAGTTGATAGGGCTTCTCC
TCAGGCAATAGTTGAACTTTTTCACCGTGGCTCAGCCACGCAAGTGACGGCATATCCGTCAGCGACCCTGCGGGCGAGCTCGTTATTTGTCAACTATCCCGAAGAGG

sgRNA-2 Csy4 site Suffix Linker_0

GTTACAGCCTGCGGTCCGGGTTCACTGCCGTATAGGCAGTAATTTTGTTTAACTTTAAGAAGGAGATATACATATGGTGAGCAAGGGCGAGGAGGATAACATGGCCA
CAATGTCGGACGCCAGGCCCAAGTGACGGCATATCCGTCATTAAAACAAATTGAAATTCTTCCTCTATATGTATACCACTCGTTCCCGCTCCTCCTATTGTACCGGT

Prefix Csy4 site RBS mCherry

TCATCAAGGAGTTCATGCGCTTCAAGGTGCACATGGAGGGCTCCGTGAACGGCCACGAGTTCGAGATCGAGGGCGAGGGCGAGGGCCGCCCCTACGAGGGCACCCAG
AGTAGTTCCTCAAGTACGCGAAGTTCCACGTGTACCTCCCGAGGCACTTGCCGGTGCTCAAGCTCTAGCTCCCGCTCCCGCTCCCGGCGGGGATGCTCCCGTGGGTC

mCherry

ACCGCCAAGCTGAAGGTGACCAAGGGTGGCCCCCTGCCCTTCGCCTGGGACATCCTGTCCCCTCAGTTCATGTACGGCTCCAAGGCCTACGTGAAGCACCCCGCCGA
TGGCGGTTCGACTTCCACTGGTTCCCACCGGGGGACGGGAAGCGGACCCTGTAGGACAGGGGAGTCAAGTACATGCCGAGGTTCCGGATGCACTTCGTGGGGCGGCT

mCherry

CATCCCCGACTACTTGAAGCTGTCCTTCCCCGAGGGCTTCAAGTGGGAGCGCGTGATGAACTTCGAGGACGGCGGCGTGGTGACCGTGACCCAGGACTCCTCtCTGC
GTAGGGGCTGATGAACTTCGACAGGAAGGGGCTCCCGAAGTTCACCCTCGCGCACTACTTGAAGCTCCTGCCGCCGCACCACTGGCACTGGGTCCTGAGGAGaGACG

mCherry

AGGACGGCGAGTTCATCTACAAGGTGAAGCTGCGCGGCACCAACTTCCCCTCCGACGGCCCCGTAATGCAGAAGAAGACtATGGGCTGGGAGGCCTCCTCCGAGCGG
TCCTGCCGCTCAAGTAGATGTTCCACTTCGACGCGCCGTGGTTGAAGGGGAGGCTGCCGGGGCATTACGTCTTCTTCTGaTACCCGACCCTCCGGAGGAGGCTCGCC

mCherry

ATGTACCCCGAGGACGGCGCgCTGAAGGGCGAGATCAAGCAGAGGCTGAAGCTGAAGGACGGCGGCCACTACGACGCTGAaGTCAAGACCACCTACAAGGCCAAGAA
TACATGGGGCTCCTGCCGCGcGACTTCCCGCTCTAGTTCGTCTCCGACTTCGACTTCCTGCCGCCGGTGATGCTGCGACTtCAGTTCTGGTGGATGTTCCGGTTCTT

mCherry

GCCCGTGCAGCTGCCCGGCGCgTACAACGTCAACATCAAGTTGGACATCACCTCCCACAACGAGGACTACACCATCGTGGAACAGTACGAACGCGCCGAGGGCCGCC
CGGGCACGTCGACGGGCCGCGcATGTTGCAGTTGTAGTTCAACCTGTAGTGGAGGGTGTTGCTCCTGATGTGGTAGCACCTTGTCATGCTTGCGCGGCTCCCGGCGG

mCherry

pJ2072.2_c1 (6900 bp) (from 1178-2354 bp)

ACTCCACCGGCGGCATGGACGAGCTGTACAAGATGTCCCGCCGTAATACTGACGCCATCACAATCCACAGCATCCTGGATTGGATTGAAGACAACTTGGAGTCGCCG
TGAGGTGGCCGCCGTACCTGCTCGACATGTTCTACAGGGCGGCATTATGACTGCGGTAGTGTTAGGTGTCGTAGGACCTAACCTAACTTCTGTTGAACCTCAGCGGC
mCherry MarA

TTGAGTTTAGAAAAAGTTAGTGAACGTAGTGGTTACTCAAAGTGGCACCTTCAGCGCATGTTTAAGAAGGAAACGGGTCATTCATTGGGTCAATATATTCGTTCTCG
AACTCAAATCTTTTTCAATCACTTGCATCACCAATGAGTTTCACCGTGGAAGTCGCGTACAAATTCTTCCTTTGCCCAGTAAGTAACCCAGTTATATAAGCAAGAGC
MarA

CAAGATGACTGAAATTGCCCAGAAATTGAAAGAGTCTAATGAACCTATTTTGTACCTGGCGGAGCGTTACGGCTTTGAAAGTCAGCAAACCCTTACACGTACCTTCA
GTTCTACTGACTTTAACGGGTCTTTAACTTTCTCAGATTACTTGGATAAAACATGGACCGCCTCGCAATGCCGAAACTTTCAGTCGTTTGGGAATGTGCATGGAAGT
MarA

AGAATTACTTTGACGTTCCACCACACAAATATCGTATGACCAACATGCAGGGTGAGTCACGTTTTTTGCATCCGTTGAATCATTACAATTCCTAATAATCGCTGGGA
TCTTAATGAAACTGCAAGGTGGTGTGTTTATAGCATACTGGTTGTACGTCCCACTCAGTGCAAAAAACGTAGGCAACTTAGTAATGTTAAGGATTATTAGCGACCCT
MarA

NcoI
CGCCCGCCATGGTTCAGCCAAAAAACTTAAGACCGCCGGTCTTGTCCACTACCTTGCAGTAATGCGGTGGACAGGATCGGCGGTTTTCTTTTCTCTTCTCAATTCTT
GCGGGCGGTACCAAGTCGGTTTTTTGAATTCTGGCGGCCAGAACAGGTGATGGAACGTCATTACGCCACCTGTCCTAGCCGCCAAAAGAAAAGAGAAGAGTTAAGAA
ECK120029600 Terminator

CTGACCTGTAACGAATAATAGATAGTAAAGTAGTCTCCGATTGAGTTTTCTCTGCCGAGTCCCACCCAGTTCTGTGATTTCAGTAAGTTGGTAATTGATACACTGTT
GACTGGACATTGCTTATTATCTATCATTTCATCAGAGGCTAACTCAAAAGAGACGGCTCAGGGTGGGTCAAGACACTAAAGTCATTCAACCATTAACTATGTGACAA
Spacer 1

HindIII
GCGAGAACTGCTGCCTGGTAGTAGATAGGTTGTTATTGAGTAAGAAGGTAAAGTGAACGAAATCCCTGAAACTGAGACTGTAGAAAATAAGCTTCAGCCTGCGGTCC
CGCTCTTGACGACGGACCATCATCTATCCAACAATAACTCATTCTTCCATTTCACTTGCTTTAGGGACTTTGACTCTGACATCTTTTATTCGAAGTCGGACGCCAGG
Spacer 1 Prefix

GGttgacggctagctcagtcctaggtacagtgctagcTCGCTGGGACGCCCGAGATAGCCGTTACACAGGTGACACTTATTTCAGCCTGCGGTCCGGccaCACTAGA
CCaactgccgatcgagtcaggatccatgtcacgatcgAGCGACCCTGCGGGCTCTATCGGCAATGTGTCCACTGTGAATAAAGTCGGACGCCAGGCCggtGTGATCT
P(BBa_J23100) Suffix Linker_24 Prefix bs-1

GTCTAGCTTGAGATCGCTGGGACGCCCGTGAACAGTTGCTCTGATTGAAACCACGATTCAGCCTGCGGTCCGGGTTCACTGCCGTATAGGCAGTGACTGAGCTAGTG
CAGATCGAACTCTAGCGACCCTGCGGGCACTTGTCAACGAGACTAACTTTGGTGCTAAGTCGGACGCCAGGCCCAAGTGACGGCATATCCGTCACTGACTCGATCAC
bs-1 Suffix Linker_19 Prefix Csy4 site sgRNA-3

TACTCTGTTTcAGAGCTATGCTGGAAACAGCATAGCAAGTTgAAATAAGGCTAGTCCGTTATCAACTTGAAAAAGTGGCACCGAGTCGGTGCGTTCACTGCCGTATA
ATGAGACAAAgTCTCGATACGACCTTTGTCGTATCGTTCAAcTTTATTCCGATCAGGCAATAGTTGAACTTTTTCACCGTGGCTCAGCCACGCAAGTGACGGCATAT
sgRNA-3 Csy4 site

BamHI
GGCAGTCGCTGGGACGCCCGGGATCCAAGAGATTTCTACACGATTGAGCACTGTCTCAGCCTGCGGTCCGGGTTCACTGCCGTATAGGCAGTAATTTTGTTTAACTT
CCGTCAGCGACCCTGCGGGCCCTAGGTTCTCTAAAGATGTGCTAACTCGTGACAGAGTCGGACGCCAGGCCCAAGTGACGGCATATCCGTCATTAAAACAAATTGAA
Suffix Linker_10 Prefix Csy4 site RBS

pJ2072.2_c1 (6900 bp) (from 2355-3531 bp)

TAAGAAGGAGATATACATATGTCTAAAGGTGAAGAATTATTCACTGGTGTTGTCCCAATTTTGGTTGAATTAGATGGTGATGTTAATGGTCACAAATTTTCTGTCTC
ATTCTTCCTCTATATGTATACAGATTTCCACTTCTTAATAAGTGACCACAACAGGGTTAAAACCAACTTAATCTACCACTACAATTACCAGTGTTTAAAAGACAGAG
RBS mCitrine

CGGTGAAGGTGAAGGTGATGCTACTTACGGTAAATTGACCTTAAAATTTATTTGTACTACTGGTAAATTGCCAGTTCCgTGGCCAACCTTAGTCACTACTTTAGGTT
GCCACTTCCACTTCCACTACGATGAATGCCATTTAACTGGAATTTTAAATAAACATGATGACCATTTAACGGTCAAGGcACCGGTTGGAATCAGTGATGAAATCCAA
mCitrine

ATGGTTTGATGTGTTTTGCTAGATACCCAGATCATATGAAACAACATGACTTTTTCAAGTCTGCCATGCCAGAAGGTTATGTTCAAGAAAGAACTATTTTTTTCAAA
TACCAAACTACACAAAACGATCTATGGGTCTAGTATACTTTGTTGTACTGAAAAAGTTCAGACGGTACGGTCTTCCAATACAAGTTCTTTCTTGATAAAAAAAGTTT
mCitrine

GATGACGGTAACTACAAGACCAGAGCTGAAGTCAAGTTTGAAGGTGATACCTTAGTTAATAGAATCGAATTAAAAGGTATTGATTTTAAAGAAGATGGTAACATTTT
CTACTGCCATTGATGTTCTGGTCTCGACTTCAGTTCAAACTTCCACTATGGAATCAATTATCTTAGCTTAATTTTCCATAACTAAAATTTCTTCTACCATTGTAAAA
mCitrine

AGGTCACAAATTGGAATACAACTATAACTCTCACAATGTTTACATCATGGCTGACAAACAAAAGAATGGTATCAAAGTTAACTTCAAAATTAGACACAACATTGAAG
TCCAGTGTTTAACCTTATGTTGATATTGAGAGTGTTACAAATGTAGTACCGACTGTTTGTTTTCTTACCATAGTTTCAATTGAAGTTTTAATCTGTGTTGTAACTTC
mCitrine

ATGGTTCTGTTCAATTAGCTGACCATTATCAACAAAATACTCCAATTGGTGATGGTCCAGTCTTGTTACCAGACAACCATTACTTATCCTATCAATCTaaaTTATCC
TACCAAGACAAGTTAATCGACTGGTAATAGTTGTTTTATGAGGTTAACCACTACCAGGTCAGAACAATGGTCTGTTGGTAATGAATAGGATAGTTAGAtttAATAGG
mCitrine

AAAGATCCAAACGAAAAGAGAGACCACATGGTCTTGTTAGAATTTGTTACTGCTGCTGGTATTACCCAcGGTATGGATGAATTGTACAAAATGTCCCGCCGTAATAC
TTTCTAGGTTTGCTTTTCTCTCTGGTGTACCAGAACAATCTTAAACAATGACGACGACCATAATGGGTgCCATACCTACTTAACATGTTTTACAGGGCGGCATTATG
mCitrine MarAn20

SbfI KpnI

TGACGCCATCACAATCCACAGCATCCTGGATTGGATTGAAGACTAATAATCGCTGGGACGCCCGCCTGCAGGCTCGGTACCAAATTCCAGAAAAGAGGCCTCCCGAA
ACTGCGGTAGTGTTAGGTGTCGTAGGACCTAACCTAACTTCTGATTATTAGCGACCCTGCGGGCGGACGTCCGAGCCATGGTTTAAGGTCTTTTCTCCGGAGGGCTT
MarAn20 Suffix L3S2P21 Terminator

AGGGGGGCCTTTTTTCGTTTTGGTCCTAATAGATAAAGGATAGGTCTGGTAGTGTTGTTCGTTCTCGCAGGTAAATCAATAATACTCAGCAGTTCCGTAGACTTTTC
TCCCCCCGGAAAAAAGCAAAACCAGGATTATCTATTTCCTATCCAGACCATCACAACAAGCAAGAGCGTCCATTTAGTTATTATGAGTCGTCAAGGCATCTGAAAAG
L3S2P21 Terminator Spacer 2.5

AGTGGGACAGGGTAGCGATAACAGATAGATTGTAATAAGACACAGTAGGTGCTCGTAGTTGCGTGAAGAGAACCGCTCAGGAAATCCAGTCAGAAGTATTGGTAATC
TCACCCTGTCCCATCGCTATTGTCTATCTAACATTATTCTGTGTCATCCACGAGCATCAACGCACTTCTCTTGGCGAGTCCTTTAGGTCAGTCTTCATAACCATTAG
Spacer 2.5

SalI

GTTGAAAACTCAGTCGACCAGCCTGCGGTCCGGttgacggctagctcagtcctaggtacagtgctagcTCGCTGGGACGCCCGAGTGACGACTGCGAAGTAACCTCT
CAACTTTTGAGTCAGCTGGTCGGACGCCAGGCCaactgccgatcgagtcaggatccatgtcacgatcgAGCGACCCTGCGGGCTCACTGCTGACGCTTCATTGGAGA
Spa....5 Prefix P(BBa_J23100) Suffix Linker_25

pJ2072.2_c1 (6900 bp) (from 3532-4815 bp)

ATTTATCAGCCTGCGGTCCGGccaAGTACTTAGTACACACTGATTCGCTGGGACGCCCGGTAGTGCTTATCAGACCCAATACTGTTGAACAGCCTGCGGTCCGGGTT
TAAATAGTCGGACGCCAGGCCggtTCATGAATCATGTGTGACTAAGCGACCCTGCGGGCCATCACGAATAGTCTGGGTTATGACAACTTGTCGGACGCCAGGCCCAA
Prefix bs-2 Suffix Linker_17 Prefix

CACTGCCGTATAGGCAGTCTCAAGCTAGACTCTAGTGGTTTcAGAGCTATGCTGGAAACAGCATAGCAAGTTgAAATAAGGCTAGTCCGTTATCAACTTGAAAAAGT
GTGACGGCATATCCGTCAGAGTTCGATCTGAGATCACCAAAgTCTCGATACGACCTTTGTCGTATCGTTCAAcTTTATTCCGATCAGGCAATAGTTGAACTTTTTCA
Csy4 site sgRNA-1

AatII

GGCACCGAGTCGGTGCGTTCACTGCCGTATAGGCAGTCGCTGGGACGCCCGGACGTCCTATTACACTCGTCGTTGGAAACTGAAGATCAGCCTGCGGTCCGGGTTCA
CCGTGGCTCAGCCACGCAAGTGACGGCATATCCGTCAGCGACCCTGCGGGCCTGCAGGATAATGTGAGCAGCAACCTTTGACTTCTAGTCGGACGCCAGGCCCAAGT
sgRNA-1 Csy4 site Suffix Linker_11 Prefix

CTGCCGTATAGGCAGTAATTTTGTTTAACTTTAAGAAGGAGATATACATATGAATCAGTCATTCATCTCGGACATCTTATATGCCGACATCGAATCGAAGGCTAAGG
GACGGCATATCCGTCATTAAAACAAATTGAAATTCTTCCTCTATATGTATACTTAGTCAGTAAGTAGAGCCTGTAGAATATACGGCTGTAGCTTAGCTTCCGATTCC
Csy4 site RBS RepA70

AACTTACAGTCAATTCCAACAATACTGTCCAGCCGGTCGCGCTTATGCGCTTAGGAGTTTTCGTTCCCAAACCTTCCAAGAGCAAAGGAGAAAGTAAGGAAATTGAC
TTGAATGTCAGTTAAGGTTGTTATGACAGGTCGGCCAGCGCGAATACGCGAATCCTCAAAAGCAAGGGTTTGGAAGGTTCTCGTTTCCTCTTTCATTCCTTTAACTG
RepA70

GCCACCAAAGCCTTCTCTCAACTGGAGATTGCTAAAGCAGAGGGCATGAGTAAAGGAGAAGAACTTTTCACTGGAGTTGTCCCAATTCTTGTTGAATTAGATGGTGA
CGGTGGTTTCGGAAGAGAGTTGACCTCTAACGATTTCGTCTCCCGTACTCATTTCCTCTTCTTGAAAAGTGACCTCAACAGGGTTAAGAACAACTTAATCTACCACT
RepA70 Cerulean

TGTTAATGGGCACAAATTTTCTGTCAGTGGAGAGGGTGAAGGTGATGCAACATACGGAAAACTTACCCTTAAATTTATTTGCACTACTGGAAAACTACCTGTTCCgT
ACAATTACCCGTGTTTAAAAGACAGTCACCTCTCCCACTTCCACTACGTTGTATGCCTTTTGAATGGGAATTTAAATAAACGTGATGACCTTTTGATGGACAAGGcA
Cerulean

GGCCAACACTTGTCACTACTTTGACTTGGGGTGTTCAATGCTTTGCTAGATACCCAGATCATATGAAACAGCATGACTTTTTCAAGAGTGCCATGCCCGAAGGTTAT
CCGGTTGTGAACAGTGATGAAACTGAACCCCACAAGTTACGAAACGATCTATGGGTCTAGTATACTTTGTCGTACTGAAAAAGTTCTCACGGTACGGGCTTCCAATA
Cerulean

GTACAGGAAAGAACTATATTTTTCAAAGATGACGGGAACTACAAGACACGTGCTGAAGTCAAGTTTGAAGGTGATACCCTTGTTAATAGAATCGAGTTAAAAGGTAT
CATGTCCTTTCTTGATATAAAAAGTTTCTACTGCCCTTGATGTTCTGTGCACGACTTCAGTTCAAACTTCCACTATGGGAACAATTATCTTAGCTCAATTTTCCATA
Cerulean

TGATTTTAAAGAAGATGGAAACATTCTTGGACACAAATTGGAATACAACGCTATTTCAGATAATGTATACATCACTGCAGACAAACAAAAGAATGGAATCAAAGCTA
ACTAAAATTTCTTCTACCTTTGTAAGAACCTGTGTTTAACCTTATGTTGCGATAAAGTCTATTACATATGTAGTGACGTCTGTTTGTTTTCTTACCTTAGTTTCGAT
Cerulean

ATTTCAAAATTAGACACAACATTGAAGATGGAAGCGTTCAACTAGCAGACCATTATCAACAAAATACTCCAATTGGCGATGGCCCTGTCCTTTTACCAGACAACCAT
TAAAGTTTTAATCTGTGTTGTAACTTCTACCTTCGCAAGTTGATCGTCTGGTAATAGTTGTTTTATGAGGTTAACCGCTACCGGGACAGGAAAATGGTCTGTTGGTA
Cerulean

TACCTGTCCACACAATCTaaaCTTTCGAAAGATCCCAACGAAAAGAGAGACCACATGGTCCTTCTTGAGTTTGTAACAGCTGCTGGGATTACACTAGGCATGGATGA
ATGGACAGGTGTGTTAGAtttGAAAGCTTTCTAGGGTTGCTTTTCTCTCTGGTGTACCAGGAAGAACTCAAACATTGTCGACGACCCTAATGTGATCCGTACCTACT
Cerulean

pJ2072.2_c1 (6900 bp) (from 4816-6099 bp)

NotI

ACTATACAAATAATAATCGCTGGGACGCCCGGCGGCCGCggaaacacagAAAAAAGCCCGCACCTGACAGTGCGGGCTTTTTTTTTcgaccaaaggTAGCGAACGAC
TGATATGTTTATTATTAGCGACCCTGCGGGCCGCCGGCGcctttgtgtcTTTTTTCGGGCGTGGACTGTCACGCCCGAAAAAAAAAgctggtttccATCGCTTGCTG

Cerulean | Suffix | ECK120033737 Terminator | Li...8

KasI BbvCI AscI

GAGTCACTGTTGAGGATAAATACTTTCTCTACTAGGCGCCTGTTACACAGGTCCTCAGCGGCGCGCCTTTGTCGGTGAACGCTCTCCTGAGTAGGACAAATCCGCCG
CTCAGTGACAACTCCTATTTATGAAAGAGATGATCCGCGGACAATGTGTCCAGGAGTCGCCGCGCGGAAACAGCCACTTGCGAGAGGACTCATCCTGTTTAGGCGGC

Linker_8 | Spa...15

GGAGCGGATTTGAACGTTGTGAAGCAACGGCCCGGAGGGTGGCGGGCAGGACGCCCGCCATAAACTGCCAGGCATCAAACTAAGCAGAAGGCCATCCTGACGGATGG
CCTCGCCTAAACTTGCAACACTTCGTTGCCGGGCCTCCCACCGCCCGTCCTGCGGGCGGTATTTGACGGTCCGTAGTTTGATTCGTCTTCCGGTAGGACTGCCTACC

CCTTTTTGCGTTTCAGATCTACCGGTaaaccagcaatagacataagcggctatttaacgaccctgccctgaaccgacgacaagctgacgaccgggtctccgcaagtg
GGAAAAACGCAAAGTCTAGATGGCCAtttggtcgttatctgtattcgccgataaattgctgggacgggacttggctgctgttcgactgctggcccagaggcgttcac

gcacttttcggggaaatgtgcgcggaacccctatttgtttatttttctaaatacattcaaatatgtatccgctcatgaattaattcttagaaaaactcatcgagcat
cgtgaaaagcccctttacacgcgccttggggataaacaaataaaaagatttatgtaagtttatacataggcgagtacttaattaagaatctttttgagtagctcgta

KanR

caaatgaaactgcaatttattcatatcaggattatcaataccatatttttgaaaaagccgtttctgtaatgaaggagaaaactcaccgaggcagttccataggatgg
gtttactttgacgttaaataagtatagtcctaatagttatggtataaaaactttttcggcaaagacattacttcctcttttgagtggctccgtcaaggtatcctacc

KanR

caagatcctggtatcggtctgcgattccgactcgtccaacatcaatacaacctattaatttcccctcgtcaaaaataaggttatcaagtgagaaatcaccatgagtg
gttctaggaccatagccagacgctaaggctgagcaggttgtagttatgttggataattaaaggggagcagtttttattccaatagttcactctttagtggtactcac

KanR

acgactgaatccggtgagaatggcaaaagtttatgcatttctttccagacttgttcaacaggccagccattacgctcgtcatcaaaatcactcgcatcaaccaaacc
tgctgacttaggccactcttaccgttttcaaatacgtaaagaaaggtctgaacaagttgtccggtcggtaatgcgagcagtagttttagtgagcgtagttggtttgg

KanR

gttattcattcgtgattgcgcctgagcgagacgaaatacgcggtcgctgttaaaaggacaattacaaacaggaatcgaatgcaaccggcgcaggaacactgccagcg
caataagtaagcactaacgcggactcgctctgctttatgcgccagcgacaattttcctgttaatgtttgtccttagcttacgttggccgcgtccttgtgacggtcgc

KanR

catcaacaatattttcacctgaatcaggatattcttctaatacctggaatgctgttttcccggggatcgcagtggtgagtaaccatgcatcatcaggagtacggata
gtagttgttataaaagtggacttagtcctataagaagattatggaccttacgacaaaagggcccctagcgtcaccactcattggtacgtagtagtcctcatgcctat

KanR

aaatgcttgatggtcggaagaggcataaattccgtcagccagtttagtctgaccatctcatctgtaacatcattggcaacgctacctttgccatgtttcagaaacaa
tttacgaactaccagccttctccgtatttaaggcagtcggtcaaatcagactggtagagtagacattgtagtaaccgttgcgatggaaacggtacaaagtctttgtt

KanR

ctctggcgcatcgggcttcccatacaatcgatagattgtcgcacctgattgcccgacattatcgcgagcccatttatacccatataaatcagcatccatgttggaat
gagaccgcgtagcccgaagggtatgttagctatctaacagcgtggactaacgggctgtaatagcgctcgggtaaatatgggtatatttagtcgtaggtacaacctta

KanR

pJ2072.2_c1 (6900 bp) (from 6100-6900 bp)

ttaatcgcggcctagagcaagacgtttcccgttgaatatggctcatactcttcctttttcaatattattgaagcatttatcagggttattgtctcatgagcggatac
aattagcgccggatctcgttctgcaaagggcaacttataccgagtatgagaaggaaaaagttataataacttcgtaaatagtcccaataacagagtactcgcctatg
KanR

atatttgaatgtatttagaaaaataaacaaataggcatgctagcgcagaaacgtcctagaagatgccaggaggatacttagcagagagacaataaggccggagcgaa
tataaacttacataaatctttttatttgtttatccgtacgatcgcgtctttgcaggatcttctacggtcctcctatgaatcgtctctctgttattccggcctcgctt
ColA ori

gccgtttttccataggctccgcccccctgacgaacatcacgaaatctgacgctcaaatcagtggtggcgaaacccgacaggactataaagataccaggcgtttcccc
cggcaaaaaggtatccgaggcggggggactgcttgtagtgctttagactgcgagtttagtcaccaccgctttgggctgtcctgatatttctatggtccgcaaagggg
ColA ori

ctgatggctccctcttgcgctctcctgttcccgtcctgcggcgtccgtgttgtggtggaggctttacccaaatcaccacgtcccgttccgtgtagacagttcgctcc
gactaccgagggagaacgcgagaggacaagggcaggacgccgcaggcacaacaccacctccgaaatgggtttagtggtgcagggcaaggcacatctgtcaagcgagg
ColA ori

aagctgggctgtgtgcaagaaccccccgttcagcccgactgctgcgccttatccggtaactatcatcttgagtccaacccggaaagacacgacaaaacgccactggc
ttcgacccgacacacgttcttggggggcaagtcgggctgacgacgcggaataggccattgatagtagaactcaggttgggcctttctgtgctgttttgcggtgaccg
ColA ori

agcagccattggtaactgagaattagtggatttagatatcgagagtcttgaagtggtggcctaacagaggctacactgaaaggacagtatttggtatctgcgctcca
tcgtcggtaaccattgactcttaatcacctaaatctatagctctcagaacttcaccaccggattgtctccgatgtgactttcctgtcataaaccatagacgcgaggt
ColA ori

ctaaagccagttaccaggttaagcagttccccaactgacttaaccttcgatcaaaccgcctccccaggcggttttttcgtttacagagcaggagattacgacgatcg
gatttcggtcaatggtccaattcgtcaaggggttgactgaattggaagctagtttggcggaggggtccgccaaaaaagcaaatgtctcgtcctctaatgctgctagc
ColA ori

taaaaggatctcaagaagatcctttacggattcccgacaccatcactctaga
attttcctagagttcttctaggaaatgcctaagggctgtggtagtgagatct
ColA ori

(from 1-1177 bp)

pJ2072.2_CRISPRlator (6900 bp)

EcoRI SacI

GAATTCATAGGATAGATTCTGGAAACTTTACCGTCCGAGCTCCAGCCTGCGGTCCGGttgacggctagctcagtcctaggtacagtgctagcTCGCTGGGACGCCCG
CTTAAGTATCCTATCTAAGACCTTTGAAATGGCAGGCTCGAGGTCGGACGCCAGGCCaactgccgatcgagtcaggatccatgtcacgatcgAGCGACCCTGCGGGC

Linker_14 Prefix P(BBa_J23100) Suffix

AGAGCCGAATCGCACTTATTTACAGTAGTTCAGCCTGCGGTCCGGccaTACATACTCTAAGATGTGTCTCGCTGGGACGCCCGGGGACTACACTTACGAAACTATTG
TCTCGGCTTAGCGTGAATAAATGTCATCAAGTCGGACGCCAGGCCggtATGTATGAGATTCTACACAGAGCGACCCTGCGGGCCCCTGATGTGAATGCTTTGATAAC

Linker_21 Prefix bs-4 Suffix Linker_1

ATTGCTCAGCCTGCGGTCCGGGTTCACTGCCGTATAGGCAGATCAGTGTGTACTAAGTACTGTTTcAGAGCTATGCTGGAAACAGCATAGCAAGTTgAAATAAGGCT
TAACGAGTCGGACGCCAGGCCCAAGTGACGGCATATCCGTCTAGTCACACATGATTCATGACAAAgTCTCGATACGACCTTTGTCGTATCGTTCAAcTTTATTCCGA

Prefix Csy4 site sgRNA-2

XhoI

AGTCCGTTATCAACTTGAAAAAGTGGCACCGAGTCGGTGCGTTCACTGCCGTATAGGCAGTCGCTGGGACGCCCGCTCGAGCAATAAACAGTTGATAGGGCTTCTCC
TCAGGCAATAGTTGAACTTTTTCACCGTGGCTCAGCCACGCAAGTGACGGCATATCCGTCAGCGACCCTGCGGGCGAGCTCGTTATTTGTCAACTATCCCGAAGAGG

sgRNA-2 Csy4 site Suffix Linker_0

GTTACAGCCTGCGGTCCGGGTTCACTGCCGTATAGGCAGTAATTTTGTTTAACTTTAAGAAGGAGATATACATATGGTGAGCAAGGGCGAGGAGGATAACATGGCCA
CAATGTCGGACGCCAGGCCCAAGTGACGGCATATCCGTCATTAAAACAAATTGAAATTCTTCCTCTATATGTATACCACTCGTTCCCGCTCCTCCTATTGTACCGGT

Prefix Csy4 site RBS mCherry

TCATCAAGGAGTTCATGCGCTTCAAGGTGCACATGGAGGGCTCCGTGAACGGCCACGAGTTCGAGATCGAGGGCGAGGGCGAGGGCCGCCCCTACGAGGGCACCCAG
AGTAGTTCCTCAAGTACGCGAAGTTCCACGTGTACCTCCCGAGGCACTTGCCGGTGCTCAAGCTCTAGCTCCCGCTCCCGCTCCCGGCGGGGATGCTCCCGTGGGTC

mCherry

ACCGCCAAGCTGAAGGTGACCAAGGGTGGCCCCCTGCCCTTCGCCTGGGACATCCTGTCCCCTCAGTTCATGTACGGCTCCAAGGCCTACGTGAAGCACCCCGCCGA
TGGCGGTTCGACTTCCACTGGTTCCCACCGGGGGACGGGAAGCGGACCCTGTAGGACAGGGGAGTCAAGTACATGCCGAGGTTCCGGATGCACTTCGTGGGGCGGCT

mCherry

CATCCCCGACTACTTGAAGCTGTCCTTCCCCGAGGGCTTCAAGTGGGAGCGCGTGATGAACTTCGAGGACGGCGGCGTGGTGACCGTGACCCAGGACTCCTCtCTGC
GTAGGGGCTGATGAACTTCGACAGGAAGGGGCTCCCGAAGTTCACCCTCGCGCACTACTTGAAGCTCCTGCCGCCGCACCACTGGCACTGGGTCCTGAGGAGaGACG

mCherry

AGGACGGCGAGTTCATCTACAAGGTGAAGCTGCGCGGCACCAACTTCCCCTCCGACGGCCCCGTAATGCAGAAGAAGACtATGGGCTGGGAGGCCTCCTCCGAGCGG
TCCTGCCGCTCAAGTAGATGTTCCACTTCGACGCGCCGTGGTTGAAGGGGAGGCTGCCGGGGCATTACGTCTTCTTCTGaTACCCGACCCTCCGGAGGAGGCTCGCC

mCherry

ATGTACCCCGAGGACGGCGCgCTGAAGGGCGAGATCAAGCAGAGGCTGAAGCTGAAGGACGGCGGCCACTACGACGCTGAaGTCAAGACCACCTACAAGGCCAAGAA
TACATGGGGCTCCTGCCGCGcGACTTCCCGCTCTAGTTCGTCTCCGACTTCGACTTCCTGCCGCCGGTGATGCTGCGACTtCAGTTCTGGTGGATGTTCCGGTTCTT

mCherry

GCCCGTGCAGCTGCCCGGCGCgTACAACGTCAACATCAAGTTGGACATCACCTCCCACAACGAGGACTACACCATCGTGGAACAGTACGAACGCGCCGAGGGCCGCC
CGGGCACGTCGACGGGCCGCGcATGTTGCAGTTGTAGTTCAACCTGTAGTGGAGGGTGTTGCTCCTGATGTGGTAGCACCTTGTCATGCTTGCGCGGCTCCCGGCGG

mCherry

pJ2072.2_CRISPRlator (6900 bp) (from 1178-2354 bp)

ACTCCACCGGCGGCATGGACGAGCTGTACAAGATGTCCCGCCGTAATACTGACGCCATCACAATCCACAGCATCCTGGATTGGATTGAAGACAACTTGGAGTCGCCG
TGAGGTGGCCGCCGTACCTGCTCGACATGTTCTACAGGGCGGCATTATGACTGCGGTAGTGTTAGGTGTCGTAGGACCTAACCTAACTTCTGTTGAACCTCAGCGGC
mCherry MarA

TTGAGTTTAGAAAAAGTTAGTGAACGTAGTGGTTACTCAAAGTGGCACCTTCAGCGCATGTTTAAGAAGGAAACGGGTCATTCATTGGGTCAATATATTCGTTCTCG
AACTCAAATCTTTTTCAATCACTTGCATCACCAATGAGTTTCACCGTGGAAGTCGCGTACAAATTCTTCCTTTGCCCAGTAAGTAACCCAGTTATATAAGCAAGAGC
MarA

CAAGATGACTGAAATTGCCCAGAAATTGAAAGAGTCTAATGAACCTATTTTGTACCTGGCGGAGCGTTACGGCTTTGAAAGTCAGCAAACCCTTACACGTACCTTCA
GTTCTACTGACTTTAACGGGTCTTTAACTTTCTCAGATTACTTGGATAAAACATGGACCGCCTCGCAATGCCGAAACTTTCAGTCGTTTGGGAATGTGCATGGAAGT
MarA

AGAATTACTTTGACGTTCCACCACACAAATATCGTATGACCAACATGCAGGGTGAGTCACGTTTTTTGCATCCGTTGAATCATTACAATTCCTAATAATCGCTGGGA
TCTTAATGAAACTGCAAGGTGGTGTGTTTATAGCATACTGGTTGTACGTCCCACTCAGTGCAAAAAACGTAGGCAACTTAGTAATGTTAAGGATTATTAGCGACCCT
MarA

NcoI
CGCCCGCCATGGTTCAGCCAAAAAACTTAAGACCGCCGGTCTTGTCCACTACCTTGCAGTAATGCGGTGGACAGGATCGGCGGTTTTCTTTTCTCTTCTCAATTCTT
GCGGGCGGTACCAAGTCGGTTTTTTGAATTCTGGCGGCCAGAACAGGTGATGGAACGTCATTACGCCACCTGTCCTAGCCGCCAAAAGAAAAGAGAAGAGTTAAGAA
ECK120029600 Terminator

CTGACCTGTAACGAATAATAGATAGTAAAGTAGTCTCCGATTGAGTTTTCTCTGCCGAGTCCCACCCAGTTCTGTGATTTCAGTAAGTTGGTAATTGATACACTGTT
GACTGGACATTGCTTATTATCTATCATTTCATCAGAGGCTAACTCAAAAGAGACGGCTCAGGGTGGGTCAAGACACTAAAGTCATTCAACCATTAACTATGTGACAA
Spacer 1

HindIII
GCGAGAACTGCTGCCTGGTAGTAGATAGGTTGTTATTGAGTAAGAAGGTAAAGTGAACGAAATCCCTGAAACTGAGACTGTAGAAAATAAGCTTCAGCCTGCGGTCC
CGCTCTTGACGACGGACCATCATCTATCCAACAATAACTCATTCTTCCATTTCACTTGCTTTAGGGACTTTGACTCTGACATCTTTTATTCGAAGTCGGACGCCAGG
Spacer 1 Prefix

GGttgacggctagctcagtcctaggtacagtgctagcTCGCTGGGACGCCCGAGATAGCCGTTACACAGGTGACACTTATTTCAGCCTGCGGTCCGGccaCACTAGA
CCaactgccgatcgagtcaggatccatgtcacgatcgAGCGACCCTGCGGGCTCTATCGGCAATGTGTCCACTGTGAATAAAGTCGGACGCCAGGCCggtGTGATCT
P(BBa_J23100) Suffix Linker_24 Prefix bs-1

GTCTAGCTTGAGATCGCTGGGACGCCCGTGAACAGTTGCTCTGATTGAAACCACGATTCAGCCTGCGGTCCGGGTTCACTGCCGTATAGGCAGGACACATCTTAGAG
CAGATCGAACTCTAGCGACCCTGCGGGCACTTGTCAACGAGACTAACTTTGGTGCTAAGTCGGACGCCAGGCCCAAGTGACGGCATATCCGTCCTGTGTAGAATCTC
bs-1 Suffix Linker_19 Prefix Csy4 site sgRNA-4

TATGTAGTTTcAGAGCTATGCTGGAAACAGCATAGCAAGTTgAAATAAGGCTAGTCCGTTATCAACTTGAAAAAGTGGCACCGAGTCGGTGCGTTCACTGCCGTATA
ATACATCAAAgTCTCGATACGACCTTTGTCGTATCGTTCAAcTTTATTCCGATCAGGCAATAGTTGAACTTTTTCACCGTGGCTCAGCCACGCAAGTGACGGCATAT
sgRNA-4 Csy4 site

BamHI
GGCAGTCGCTGGGACGCCCGGGATCCAAGAGATTTCTACACGATTGAGCACTGTCTCAGCCTGCGGTCCGGGTTCACTGCCGTATAGGCAGTAATTTTGTTTAACTT
CCGTCAGCGACCCTGCGGGCCCTAGGTTCTCTAAAGATGTGCTAACTCGTGACAGAGTCGGACGCCAGGCCCAAGTGACGGCATATCCGTCATTAAAACAAATTGAA
Suffix Linker_10 Prefix Csy4 site RBS

pJ2072.2_CRISPRlator (6900 bp) (from 2355-3531 bp)

TAAGAAGGAGATATACATATGTCTAAAGGTGAAGAATTATTCACTGGTGTTGTCCCAATTTTGGTTGAATTAGATGGTGATGTTAATGGTCACAAATTTTCTGTCTC
ATTCTTCCTCTATATGTATACAGATTTCCACTTCTTAATAAGTGACCACAACAGGGTTAAAACCAACTTAATCTACCACTACAATTACCAGTGTTTAAAAGACAGAG
RBS | mCitrine

CGGTGAAGGTGAAGGTGATGCTACTTACGGTAAATTGACCTTAAAATTTATTTGTACTACTGGTAAATTGCCAGTTCCgTGGCCAACCTTAGTCACTACTTTAGGTT
GCCACTTCCACTTCCACTACGATGAATGCCATTTAACTGGAATTTTAAATAAACATGATGACCATTTAACGGTCAAGGcACCGGTTGGAATCAGTGATGAAATCCAA
mCitrine

ATGGTTTGATGTGTTTTGCTAGATACCCAGATCATATGAAACAACATGACTTTTTCAAGTCTGCCATGCCAGAAGGTTATGTTCAAGAAAGAACTATTTTTTTCAAA
TACCAAACTACACAAAACGATCTATGGGTCTAGTATACTTTGTTGTACTGAAAAAGTTCAGACGGTACGGTCTTCCAATACAAGTTCTTTCTTGATAAAAAAAGTTT
mCitrine

GATGACGGTAACTACAAGACCAGAGCTGAAGTCAAGTTTGAAGGTGATACCTTAGTTAATAGAATCGAATTAAAAGGTATTGATTTTAAAGAAGATGGTAACATTTT
CTACTGCCATTGATGTTCTGGTCTCGACTTCAGTTCAAACTTCCACTATGGAATCAATTATCTTAGCTTAATTTTCCATAACTAAAATTTCTTCTACCATTGTAAAA
mCitrine

AGGTCACAAATTGGAATACAACTATAACTCTCACAATGTTTACATCATGGCTGACAAACAAAAGAATGGTATCAAAGTTAACTTCAAAATTAGACACAACATTGAAG
TCCAGTGTTTAACCTTATGTTGATATTGAGAGTGTTACAAATGTAGTACCGACTGTTTGTTTTCTTACCATAGTTTCAATTGAAGTTTTAATCTGTGTTGTAACTTC
mCitrine

ATGGTTCTGTTCAATTAGCTGACCATTATCAACAAAATACTCCAATTGGTGATGGTCCAGTCTTGTTACCAGACAACCATTACTTATCCTATCAATCTaaaTTATCC
TACCAAGACAAGTTAATCGACTGGTAATAGTTGTTTTATGAGGTTAACCACTACCAGGTCAGAACAATGGTCTGTTGGTAATGAATAGGATAGTTAGAtttAATAGG
mCitrine

AAAGATCCAAACGAAAAGAGAGACCACATGGTCTTGTTAGAATTTGTTACTGCTGCTGGTATTACCCAcGGTATGGATGAATTGTACAAAATGTCCCGCCGTAATAC
TTTCTAGGTTTGCTTTTCTCTCTGGTGTACCAGAACAATCTTAAACAATGACGACGACCATAATGGGTgCCATACCTACTTAACATGTTTTACAGGGCGGCATTATG
mCitrine | MarAn20

SbfI KpnI

TGACGCCATCACAATCCACAGCATCCTGGATTGGATTGAAGACTAATAATCGCTGGGACGCCCGCCTGCAGGCTCGGTACCAAATTCCAGAAAAGAGGCCTCCCGAA
ACTGCGGTAGTGTTAGGTGTCGTAGGACCTAACCTAACTTCTGATTATTAGCGACCCTGCGGGCGGACGTCCGAGCCATGGTTTAAGGTCTTTTCTCCGGAGGGCTT
MarAn20 | Suffix | | L3S2P21 Terminator

AGGGGGGCCTTTTTTCGTTTTGGTCCTAATAGATAAAGGATAGGTCTGGTAGTGTTGTTCGTTCTCGCAGGTAAATCAATAATACTCAGCAGTTCCGTAGACTTTTC
TCCCCCCGGAAAAAAGCAAAACCAGGATTATCTATTTCCTATCCAGACCATCACAACAAGCAAGAGCGTCCATTTAGTTATTATGAGTCGTCAAGGCATCTGAAAAG
L3S2P21 Terminator | Spacer 2.5

AGTGGGACAGGGTAGCGATAACAGATAGATTGTAATAAGACACAGTAGGTGCTCGTAGTTGCGTGAAGAGAACCGCTCAGGAAATCCAGTCAGAAGTATTGGTAATC
TCACCCTGTCCCATCGCTATTGTCTATCTAACATTATTCTGTGTCATCCACGAGCATCAACGCACTTCTCTTGGCGAGTCCTTTAGGTCAGTCTTCATAACCATTAG
Spacer 2.5

SalI

GTTGAAAACTCAGTCGACCAGCCTGCGGTCCGGttgacggctagctcagtcctaggtacagtgctagcTCGCTGGGACGCCCGAGTGACGACTGCGAAGTAACCTCT
CAACTTTTGAGTCAGCTGGTCGGACGCCAGGCCaactgccgatcgagtcaggatccatgtcacgatcgAGCGACCCTGCGGGCTCACTGCTGACGCTTCATTGGAGA
Spa....5 | | Prefix | P(BBa_J23100) | Suffix | Linker_25

pJ2072.2_CRISPRlator (6900 bp) (from 3532-4815 bp)

ATTTATCAGCCTGCGGTCCGGccaAGTACTTAGTACACACTGATTCGCTGGGACGCCCGGTAGTGCTTATCAGACCCAATACTGTTGAACAGCCTGCGGTCCGGGTT
TAAATAGTCGGACGCCAGGCCggtTCATGAATCATGTGTGACTAAGCGACCCTGCGGGCCATCACGAATAGTCTGGGTTATGACAACTTGTCGGACGCCAGGCCCAA

Prefix bs-2 Suffix Linker_17 Prefix

CACTGCCGTATAGGCAGTCTCAAGCTAGACTCTAGTGGTTTcAGAGCTATGCTGGAAACAGCATAGCAAGTTgAAATAAGGCTAGTCCGTTATCAACTTGAAAAAGT
GTGACGGCATATCCGTCAGAGTTCGATCTGAGATCACCAAAgTCTCGATACGACCTTTGTCGTATCGTTCAAcTTTATTCCGATCAGGCAATAGTTGAACTTTTTCA

Csy4 site sgRNA-1

AatII

GGCACCGAGTCGGTGCGTTCACTGCCGTATAGGCAGTCGCTGGGACGCCCGGACGTCCTATTACACTCGTCGTTGGAAACTGAAGATCAGCCTGCGGTCCGGGTTCA
CCGTGGCTCAGCCACGCAAGTGACGGCATATCCGTCAGCGACCCTGCGGGCCTGCAGGATAATGTGAGCAGCAACCTTTGACTTCTAGTCGGACGCCAGGCCCAAGT

sgRNA-1 Csy4 site Suffix Linker_11 Prefix

CTGCCGTATAGGCAGTAATTTTGTTTAACTTTAAGAAGGAGATATACATATGAATCAGTCATTCATCTCGGACATCTTATATGCCGACATCGAATCGAAGGCTAAGG
GACGGCATATCCGTCATTAAAACAAATTGAAATTCTTCCTCTATATGTATACTTAGTCAGTAAGTAGAGCCTGTAGAATATACGGCTGTAGCTTAGCTTCCGATTCC

Csy4 site RBS RepA70

AACTTACAGTCAATTCCAACAATACTGTCCAGCCGGTCGCGCTTATGCGCTTAGGAGTTTTCGTTCCCAAACCTTCCAAGAGCAAAGGAGAAAGTAAGGAAATTGAC
TTGAATGTCAGTTAAGGTTGTTATGACAGGTCGGCCAGCGCGAATACGCGAATCCTCAAAAGCAAGGGTTTGGAAGGTTCTCGTTTCCTCTTTCATTCCTTTAACTG

RepA70

GCCACCAAAGCCTTCTCTCAACTGGAGATTGCTAAAGCAGAGGGCATGAGTAAAGGAGAAGAACTTTTCACTGGAGTTGTCCCAATTCTTGTTGAATTAGATGGTGA
CGGTGGTTTCGGAAGAGAGTTGACCTCTAACGATTTCGTCTCCCGTACTCATTTCCTCTTCTTGAAAAGTGACCTCAACAGGGTTAAGAACAACTTAATCTACCACT

RepA70 Cerulean

TGTTAATGGGCACAAATTTTCTGTCAGTGGAGAGGGTGAAGGTGATGCAACATACGGAAAACTTACCCTTAAATTTATTTGCACTACTGGAAAACTACCTGTTCCgT
ACAATTACCCGTGTTTAAAAGACAGTCACCTCTCCCACTTCCACTACGTTGTATGCCTTTTGAATGGGAATTTAAATAAACGTGATGACCTTTTGATGGACAAGGcA

Cerulean

GGCCAACACTTGTCACTACTTTGACTTGGGGTGTTCAATGCTTTGCTAGATACCCAGATCATATGAAACAGCATGACTTTTTCAAGAGTGCCATGCCCGAAGGTTAT
CCGGTTGTGAACAGTGATGAAACTGAACCCCACAAGTTACGAAACGATCTATGGGTCTAGTATACTTTGTCGTACTGAAAAAGTTCTCACGGTACGGGCTTCCAATA

Cerulean

GTACAGGAAAGAACTATATTTTTCAAAGATGACGGGAACTACAAGACACGTGCTGAAGTCAAGTTTGAAGGTGATACCCTTGTTAATAGAATCGAGTTAAAAGGTAT
CATGTCCTTTCTTGATATAAAAAGTTTCTACTGCCCTTGATGTTCTGTGCACGACTTCAGTTCAAACTTCCACTATGGGAACAATTATCTTAGCTCAATTTTCCATA

Cerulean

TGATTTTAAAGAAGATGGAAACATTCTTGGACACAAATTGGAATACAACGCTATTTCAGATAATGTATACATCACTGCAGACAAACAAAAGAATGGAATCAAAGCTA
ACTAAAATTTCTTCTACCTTTGTAAGAACCTGTGTTTAACCTTATGTTGCGATAAAGTCTATTACATATGTAGTGACGTCTGTTTGTTTTCTTACCTTAGTTTCGAT

Cerulean

ATTTCAAAATTAGACACAACATTGAAGATGGAAGCGTTCAACTAGCAGACCATTATCAACAAAATACTCCAATTGGCGATGGCCCTGTCCTTTTACCAGACAACCAT
TAAAGTTTTAATCTGTGTTGTAACTTCTACCTTCGCAAGTTGATCGTCTGGTAATAGTTGTTTTATGAGGTTAACCGCTACCGGGACAGGAAAATGGTCTGTTGGTA

Cerulean

TACCTGTCCACACAATCTaaaCTTTCGAAAGATCCCAACGAAAAGAGAGACCACATGGTCCTTCTTGAGTTTGTAACAGCTGCTGGGATTACACTAGGCATGGATGA
ATGGACAGGTGTGTTAGAtttGAAAGCTTTCTAGGGTTGCTTTTCTCTCTGGTGTACCAGGAAGAACTCAAACATTGTCGACGACCCTAATGTGATCCGTACCTACT

Cerulean

pJ2072.2_CRISPRlator (6900 bp) (from 4816-6099 bp)

NotI

ACTATACAAATAATAATCGCTGGGACGCCCGGCGGCCGCggaaacacagAAAAAAGCCCGCACCTGACAGTGCGGGCTTTTTTTTTcgaccaaaggTAGCGAACGAC
TGATATGTTTATTATTAGCGACCCTGCGGGCCGCCGGCGcctttgtgtcTTTTTTCGGGCGTGGACTGTCACGCCCGAAAAAAAAAgctggtttccATCGCTTGCTG

Cerulean Suffix ECK120033737 Terminator Li...8

KasI BbvCI AscI

GAGTCACTGTTGAGGATAAATACTTTCTCTACTAGGCGCCTGTTACACAGGTCCTCAGCGGCGCGCCTTTGTCGGTGAACGCTCTCCTGAGTAGGACAAATCCGCCG
CTCAGTGACAACTCCTATTTATGAAAGAGATGATCCGCGGACAATGTGTCCAGGAGTCGCCGCGCGGAAACAGCCACTTGCGAGAGGACTCATCCTGTTTAGGCGGC

Linker_8 Spa...15

GGAGCGGATTTGAACGTTGTGAAGCAACGGCCCGGAGGGTGGCGGGCAGGACGCCCGCCATAAACTGCCAGGCATCAAACTAAGCAGAAGGCCATCCTGACGGATGG
CCTCGCCTAAACTTGCAACACTTCGTTGCCGGGCCTCCCACCGCCCGTCCTGCGGGCGGTATTTGACGGTCCGTAGTTTGATTCGTCTTCCGGTAGGACTGCCTACC

CCTTTTTGCGTTTCAGATCTACCGGTaaaccagcaatagacataagcggctatttaacgaccctgccctgaaccgacgacaagctgacgaccgggtctccgcaagtg
GGAAAAACGCAAAGTCTAGATGGCCAtttggtcgttatctgtattcgccgataaattgctgggacgggacttggctgctgttcgactgctggcccagaggcgttcac

gcacttttcggggaaatgtgcgcggaacccctatttgtttatttttctaaatacattcaaatatgtatccgctcatgaattaattcttagaaaaactcatcgagcat
cgtgaaaagcccctttacacgcgccttggggataaacaaataaaaagatttatgtaagtttatacatagcgagtacttaattaagaatctttttgagtagctcgta

KanR

caaatgaaactgcaatttattcatatcaggattatcaataccatatttttgaaaaagccgtttctgtaatgaaggagaaaactcaccgaggcagttccataggatgg
gtttactttgacgttaaataagtatagtcctaatagttatggtataaaaactttttcggcaaagacattacttcctcttttgagtggctccgtcaaggtatcctacc

KanR

caagatcctggtatcggtctgcgattccgactcgtccaacatcaatacaacctattaatttcccctcgtcaaaaataaggttatcaagtgagaaatcaccatgagtg
gttctaggaccatagccagacgctaaggctgagcaggttgtagttatgttggataattaaaggggagcagtttttattccaatagttcactctttagtggtactcac

KanR

acgactgaatccggtgagaatggcaaaagtttatgcatttctttccagacttgttcaacaggccagccattacgctcgtcatcaaaatcactcgcatcaaccaaacc
tgctgacttaggccactcttaccgttttcaaatacgtaaagaaaggtctgaacaagttgtccggtcggtaatgcgagcagtagttttagtgagcgtagttggtttgg

KanR

gttattcattcgtgattgcgcctgagcgagacgaaatacgcggtcgctgttaaaaggacaattacaaacaggaatcgaatgcaaccggcgcaggaacactgccagcg
caataagtaagcactaacgcggactcgctctgctttatgcgccagcgacaattttcctgttaatgtttgtccttagcttacgttggccgcgtccttgtgacggtcgc

KanR

catcaacaatattttcacctgaatcaggatattcttctaatacctggaatgctgttttcccggggatcgcagtggtgagtaaccatgcatcatcaggagtacggata
gtagttgttataaaagtggacttagtcctataagaagattatggaccttacgacaaaagggcccctagcgtcaccactcattggtacgtagtagtcctcatgcctat

KanR

aaatgcttgatggtcggaagaggcataaattccgtcagccagtttagtctgaccatctcatctgtaacatcattggcaacgctacctttgccatgtttcagaaacaa
tttacgaactaccagccttctccgtatttaaggcagtcggtcaaatcagactggtagagtagacattgtagtaaccgttgcgatggaaacggtacaaagtctttgtt

KanR

ctctggcgcatcgggcttcccatacaatcgatagattgtcgcacctgattgcccgacattatcgcgagcccatttatacccatataaatcagcatccatgttggaat
gagaccgcgtagcccgaagggtatgttagctatctaacagcgtggactaacgggctgtaatagcgctcgggtaaatatgggtatatttagtcgtaggtacaacctta

KanR

pJ2072.2_CRISPRlator (6900 bp) (from 6100-6900 bp)

ttaatcgcggcctagagcaagacgtttcccgttgaatatggctcatactcttcctttttcaatattattgaagcatttatcagggttattgtctcatgagcggatac
aattagcgccggatctcgttctgcaaagggcaacttataccgagtatgagaaggaaaaagttataataacttcgtaaatagtcccaataacagagtactcgcctatg
KanR

atatttgaatgtatttagaaaaataaacaaataggcatgctagcgcagaaacgtcctagaagatgccaggaggatacttagcagagagacaataaggccggagcgaa
tataaacttacataaatctttttatttgtttatccgtacgatcgcgtctttgcaggatcttctacggtcctcctatgaatcgtctctctgttattccggcctcgctt
ColA ori

gccgtttttccataggctccgcccccctgacgaacatcacgaaatctgacgctcaaatcagtggtggcgaaacccgacaggactataaagataccaggcgtttcccc
cggcaaaaaggtatccgaggcggggggactgcttgtagtgctttagactgcgagtttagtcaccaccgctttgggctgtcctgatatttctatggtccgcaaagggg
ColA ori

ctgatggctccctcttgcgctctcctgttcccgtcctgcggcgtccgtgttgtggtggaggctttacccaaatcaccacgtcccgttccgtgtagacagttcgctcc
gactaccgagggagaacgcgagaggacaagggcaggacgccgcaggcacaacaccacctccgaaatgggtttagtggtgcagggcaaggcacatctgtcaagcgagg
ColA ori

aagctgggctgtgtgcaagaaccccccgttcagcccgactgctgcgccttatccggtaactatcatcttgagtccaacccggaaagacacgacaaaacgccactggc
ttcgacccgacacacgttcttggggggcaagtcgggctgacgacgcggaataggccattgatagtagaactcaggttgggcctttctgtgctgttttgcggtgaccg
ColA ori

agcagccattggtaactgagaattagtggatttagatatcgagagtcttgaagtggtggcctaacagaggctacactgaaaggacagtatttggtatctgcgctcca
tcgtcggtaaccattgactcttaatcacctaaatctatagctctcagaacttcaccaccggattgtctccgatgtgactttcctgtcataaaccatagacgcgaggt
ColA ori

ctaaagccagttaccaggttaagcagttccccaactgacttaaccttcgatcaaaccgcctccccaggcggttttttcgtttacagagcaggagattacgacgatcg
gatttcggtcaatggtccaattcgtcaaggggttgactgaattggaagctagtttggcggaggggtccgccaaaaaagcaaatgtctcgtcctctaatgctgctagc
ColA ori

taaaaggatctcaagaagatcctttacggattcccgacaccatcactctaga
attttcctagagttcttctaggaaatgcctaagggctgtggtagtgagatct
ColA ori

(from 1-1070 bp)

pJ2076.2_cL (4415 bp)

EcoRI SacI

GAATTCATAGGATAGATTCTGGAAACTTTACCGTCCGAGCTCCAGCCTGCGGTCCGGttgacggctagctcagtcctaggtacagtgctagcTCGCTGGGACGCCCG
CTTAAGTATCCTATCTAAGACCTTTGAAATGGCAGGCTCGAGGTCGGACGCCAGGCCaactgccgatcgagtcaggatccatgtcacgatcgAGCGACCCTGCGGGC

Linker_14 | Prefix | P(BBa_J23100) | Suffix

AGAGCCGAATCGCACTTATTTACAGTAGTTCAGCCTGCGGTCCGGccaTACATACTCTAAGATGTGTCTCGCTGGGACGCCCGGGGACTACACTTACGAAACTATTG
TCTCGGCTTAGCGTGAATAAATGTCATCAAGTCGGACGCCAGGCCggtATGTATGAGATTCTACACAGAGCGACCCTGCGGGCCCCTGATGTGAATGCTTTGATAAC

Linker_21 | Prefix | bs-4 | Suffix | Linker_1

ATTGCTCAGCCTGCGGTCCGGTGTAAGAGAAATCCACGAAGGAAAAGGTGAAAGCAGAACAGATAATCAGTAATAGTAGCAACTCAACCACAGGACACTTCCCTCAC
TAACGAGTCGGACGCCAGGCCACATTCTCTTTAGGTGCTTCCTTTTCCACTTTCGTCTTGTCTATTAGTCATTATCATCGTTGAGTTGGTGTCCTGTGAAGGGAGTG

Prefix | Spacer 2.2

TCCGAACGAATCACAATACGCTCCAATCTTCAACTACACGATAAAACTGGTAATCCCAACAACCTGACTCACACTCTACTTTCTCAACGAGGAACTTCTTATTCTGA
AGGCTTGCTTAGTGTTATGCGAGGTTAGAAGTTGATGTGCTATTTTGACCATTAGGGTTGTTGGACTGAGTGTGAGATGAAAGAGTTGCTCCTTGAAGAATAAGACT

Spacer 2.2

XhoI NcoI

TACAATCTCGCTGGGACGCCCGCTCGAGCAATAAACAGTTGATAGGGCTTCTCCGTTACCATGGTTCAGCCAAAAAACTTAAGACCGCCGGTCTTGTCCACTACCTT
ATGTTAGAGCGACCCTGCGGGCGAGCTCGTTATTTGTCAACTATCCCGAAGAGGCAATGGTACCAAGTCGGTTTTTTGAATTCTGGCGGCCAGAACAGGTGATGGAA

Suffix | Linker_0 | ECK120029600 Terminator

GCAGTAATGCGGTGGACAGGATCGGCGGTTTTCTTTTCTCTTCTCAATTCTTCTGACCTGTAACGAATAATAGATAGTAAAGTAGTCTCCGATTGAGTTTTCTCTGC
CGTCATTACGCCACCTGTCCTAGCCGCCAAAAGAAAAGAGAAGAGTTAAGAAGACTGGACATTGCTTATTATCTATCATTTCATCAGAGGCTAACTCAAAAGAGACG

ECK120029600 Terminator | Spacer 1

CGAGTCCCACCCAGTTCTGTGATTTCAGTAAGTTGGTAATTGATACACTGTTGCGAGAACTGCTGCCTGGTAGTAGATAGGTTGTTATTGAGTAAGAAGGTAAAGTG
GCTCAGGGTGGGTCAAGACACTAAAGTCATTCAACCATTAACTATGTGACAACGCTCTTGACGACGGACCATCATCTATCCAACAATAACTCATTCTTCCATTTCAC

Spacer 1

HindIII

AACGAAATCCCTGAAACTGAGACTGTAGAAAATAAGCTTCAGCCTGCGGTCCGGTTGACAGCTAGCTCAGTCCTAGGTACTGTGCTAGCTCGCTGGGACGCCCGAGA
TTGCTTTAGGGACTTTGACTCTGACATCTTTTATTCGAAGTCGGACGCCAGGCCAACTGTCGATCGAGTCAGGATCCATGACACGATCGAGCGACCCTGCGGGCTCT

Spacer 1 | Prefix | P(BBa_J23102) | Suffix

TAGCCGTTACACAGGTGACACTTATTTCAGCCTGCGGTCCGGccaCACTAGAGTCTAGCTTGAGATCGCTGGGACGCCCGTGAACAGTTGCTCTGATTGAAACCACG
ATCGGCAATGTGTCCACTGTGAATAAAGTCGGACGCCAGGCCggtGTGATCTCAGATCGAACTCTAGCGACCCTGCGGGCACTTGTCAACGAGACTAACTTTGGTGC

Linker_24 | Prefix | bs-1 | Suffix | Linker_19

ATTCAGCCTGCGGTCCGGGTTCACTGCCGTATAGGCAGCATCTTAGAGTATGTAGTTTcAGAGCTATGCTGGAAACAGCATAGCAAGTTgAAATAAGGCTAGTCCGT
TAAGTCGGACGCCAGGCCCAAGTGACGGCATATCCGTCGTAGAATCTCATACATCAAAgTCTCGATACGACCTTTGTCGTATCGTTCAAcTTTATTCCGATCAGGCA

Prefix | Csy4 site | sgRNA-4 t4

pJ2076.2_cL (4415 bp) (from 1071-2247 bp)

BamHI

TATCAACTTGAAAAAGTGGCACCGAGTCGGTGCGTTCACTGCCGTATAGGCAGTCGCTGGGACGCCCGGGATCCAAGAGATTTCTACACGATTGAGCACTGTCTCAG
ATAGTTGAACTTTTTCACCGTGGCTCAGCCACGCAAGTGACGGCATATCCGTCAGCGACCCTGCGGGCCCTAGGTTCTCTAAAGATGTGCTAACTCGTGACAGAGTC

sgRNA-4 t4 | Csy4 site | Suffix | | Linker_10 |

CCTGCGGTCCGGGTTCACTGCCGTATAGGCAGTAATTTTGTTTAACTTTAAGAAGGAGATATACATATGCGTAAAGGCGAAGAACTGTTTACCGGTGTGGTTCCGAT
GGACGCCAGGCCCAAGTGACGGCATATCCGTCATTAAAACAAATTGAAATTCTTCCTCTATATGTATACGCATTTCCGCTTCTTGACAAATGGCCACACCAAGGCTA

Prefix | Csy4 site | RBS | sfGFP

TCTGGTGGAACTGGACGGCGATGTTAATGGTCATAAATTCAGTGTTCGCGGCGAAGGTGAAGGCGATGCGACGAACGGCAAACTGACCCTGAAATTTATCTGCACCA
AGACCACCTTGACCTGCCGCTACAATTACCAGTATTTAAGTCACAAGCGCCGCTTCCACTTCCGCTACGCTGCTTGCCGTTTGACTGGGACTTTAAATAGACGTGGT

sfGFP

CGGGTAAACTGCCGGTCCCGTGGCCGACGCTGGTGACCACGCTGACCTATGGCGTTCAATGTTTTGCGCGTTACCCGGATCACATGAAACAGCACGACTTTTTCAAA
GCCCATTTGACGGCCAGGGCACCGGCTGCGACCACTGGTGCGACTGGATACCGCAAGTTACAAAACGCGCAATGGGCCTAGTGTACTTTGTCGTGCTGAAAAAGTTT

sfGFP

TCGGCCATGCCGGAAGGCTATGTGCAGGAACGTACGATTAGCTTTAAAGACGATGGTACGTATAAAACCCGCGCGGAAGTGAAATTCGAAGGCGATACCCTGGTTAA
AGCCGGTACGGCCTTCCGATACACGTCCTTGCATGCTAATCGAAATTTCTGCTACCATGCATATTTTGGGCGCGCCTTCACTTTAAGCTTCCGCTATGGGACCAATT

sfGFP

CCGTATCGAACTGAAAGGTATCGATTTCAAAGAAGACGGCAATATTCTGGGTCATAAACTGGAATATAACTTCAATTCCCACAACGTGTACATCACCGCGGATAAAC
GGCATAGCTTGACTTTCCATAGCTAAAGTTTCTTCTGCCGTTATAAGACCCAGTATTTGACCTTATATTGAAGTTAAGGGTGTTGCACATGTAGTGGCGCCTATTTG

sfGFP

AGAAAAACGGCATTAAAGCCAATTTCAAAATCCGCCATAATGTGGAAGATGGTAGCGTTCAGCTGGCCGACCACTATCAGCAAAACACGCCGATTGGTGATGGCCCG
TCTTTTTGCCGTAATTTCGGTTAAAGTTTTAGGCGGTATTACACCTTCTACCATCGCAAGTCGACCGGCTGGTGATAGTCGTTTTGTGCGGCTAACCACTACCGGGC

sfGFP

GTCCTGCTGCCGGACAATCACTACCTGAGTACCCAGTCCGTGCTGTCAAAAGATCCGAACGAAAAACGTGACCACATGGTCCTGCTGGAATTTGTGACGGCTGCGGG
CAGGACGACGGCCTGTTAGTGATGGACTCATGGGTCAGGCACGACAGTTTTCTAGGCTTGCTTTTTGCACTGGTGTACCAGGACGACCTTAAACACTGCCGACGCCC

sfGFP

TATCACCCACGGCATGGACGAACTGTATAAAATGTCCCGCCGTAATACTGACGCCATCACAATCCACAGCATCCTGGATTGGATTGAAGACTAATAATCGCTGGGAC
ATAGTGGGTGCCGTACCTGCTTGACATATTTTACAGGGCGGCATTATGACTGCGGTAGTGTTAGGTGTCGTAGGACCTAACCTAACTTCTGATTATTAGCGACCCTG

sfGFP | MarAn20 | Su...x

SbfI KpnI

GCCCGCCTGCAGGCTCGGTACCAAATTCCAGAAAAGAGGCCTCCCGAAAGGGGGGCCTTTTTTCGTTTTGGTCCTAATAGATAAAGGATAGGTCTGGTAGTGTTGTT
CGGGCGGACGTCCGAGCCATGGTTTAAGGTCTTTTCTCCGGAGGGCTTTCCCCCCGGAAAAAAGCAAAACCAGGATTATCTATTTCCTATCCAGACCATCACAACAA

| | L3S2P21 Terminator | Spacer 2.5

CGTTCTCGCAGGTAAATCAATAATACTCAGCAGTTCCGTAGACTTTTCAGTGGGACAGGGTAGCGATAACAGATAGATTGTAATAAGACACAGTAGGTGCTCGTAGT
GCAAGAGCGTCCATTTAGTTATTATGAGTCGTCAAGGCATCTGAAAAGTCACCCTGTCCCATCGCTATTGTCTATCTAACATTATTCTGTGTCATCCACGAGCATCA

Spacer 2.5

pJ2076.2_cL (4415 bp) (from 2248-3531 bp)

SalI AatII

TGCGTGAAGAGAACCGCTCAGGAAATCCAGTCAGAAGTATTGGTAATCGTTGAAAACTCAGTCGACGCACTTACTGAAGACGTCCTATTACACTCGTCGTTGGAAAC
ACGCACTTCTCTTGGCGAGTCCTTTAGGTCAGTCTTCATAACCATTAGCAACTTTTGAGTCAGCTGCGTGAATGACTTCTGCAGGATAATGTGAGCAGCAACCTTTG

Spacer 2.5 Spa... 5 Linker_11

NotI

TGAAGATGCGGCCGCggaaacacagAAAAAAGCCCGCACCTGACAGTGCGGGCTTTTTTTTTcgaccaaaggTAGCGAACGACGAGTCACTGTTGAGGATAAATACT
ACTTCTACGCCGGCGcctttgtgtcTTTTTTCGGGCGTGGACTGTCACGCCCGAAAAAAAAAgctggtttccATCGCTTGCTGCTCAGTGACAACTCCTATTTATGA

ECK120033737 Terminator Linker_8

KasI BbvCI AscI

TTCTCTACTAGGCGCCTGTTACACAGGTCCTCAGCGGCGCGCCTTTGTCGGTGAACGCTCTCCTGAGTAGGACAAATCCGCCGGGAGCGGATTTGAACGTTGTGAAG
AAGAGATGATCCGCGGACAATGTGTCCAGGAGTCGCCGCGCGGAAACAGCCACTTGCGAGAGGACTCATCCTGTTTAGGCGGCCCTCGCCTAAACTTGCAACACTTC

Li...8 Spa...15

CAACGGCCCGGAGGGTGGCGGGCAGGACGCCCGCCATAAACTGCCAGGCATCAAACTAAGCAGAAGGCCATCCTGACGGATGGCCTTTTTGCGTTTCAGATCTACCG
GTTGCCGGGCCTCCCACCGCCCGTCCTGCGGGCGGTATTTGACGGTCCGTAGTTTGATTCGTCTTCCGGTAGGACTGCCTACCGGAAAAACGCAAAGTCTAGATGGC

GTaaaccagcaatagacataagcggctatttaacgaccctgccctgaaccgacgacaagctgacgaccgggtctccgcaagtggcacttttcggggaaatgtgcgcg
CAtttggtcgttatctgtattcgccgataaattgctgggacgggacttggctgctgttcgactgctggcccagaggcgttcaccgtgaaaagcccctttacacgcgc

gaacccctatttgtttatttttctaaatacattcaaatatgtatccgctcatgaattaattcttagaaaaactcatcgagcatcaaatgaaactgcaatttattcat
cttggggataaacaaataaaaagatttatgtaagtttatacataggcgagtacttaattaagaatctttttgagtagctcgtagtttactttgacgttaaataagta

KanR

atcaggattatcaataccatatttttgaaaaagccgtttctgtaatgaaggagaaaactcaccgaggcagttccataggatggcaagatcctggtatcggtctgcga
tagtcctaatagttatggtataaaaactttttcggcaaagacattacttcctcttttgagtggctccgtcaaggtatcctaccgttctaggaccatagccagacgct

KanR

ttccgactcgtccaacatcaatacaacctattaatttcccctcgtcaaaaataaggttatcaagtgagaaatcaccatgagtgacgactgaatccggtgagaatggc
aaggctgagcaggttgtagttatgttggataattaaaggggagcagtttttattccaatagttcactctttagtggtactcactgctgacttaggccactcttaccg

KanR

aaaagtttatgcatttctttccagacttgttcaacaggccagccattacgctcgtcatcaaaatcactcgcatcaaccaaaccgttattcattcgtgattgcgcctg
ttttcaaatacgtaaagaaaggtctgaacaagttgtccggtcggtaatgcgagcagtagttttagtgagcgtagttggtttggcaataagtaagcactaacgcggac

KanR

agcgagacgaaatacgcggtcgctgttaaaaggacaattacaaacaggaatcgaatgcaaccggcgcaggaacactgccagcgcatcaacaatattttcacctgaat
tcgctctgctttatgcgccagcgacaattttcctgttaatgtttgtccttagcttacgttggccgcgtccttgtgacggtcgcgtagttgttataaaagtggactta

KanR

caggatattcttctaatacctggaatgctgttttcccggggatcgcagtggtgagtaaccatgcatcatcaggagtacggataaaatgcttgatggtcggaagaggc
gtcctataagaagattatggaccttacgacaaaagggcccctagcgtcaccactcattggtacgtagtagtcctcatgcctattttacgaactaccagccttctccg

KanR

ataaattccgtcagccagtttagtctgaccatctcatctgtaacatcattggcaacgctacctttgccatgtttcagaaacaactctggcgcatcgggcttcccata
tatttaaggcagtcggtcaaatcagactggtagagtagacattgtagtaaccgttgcgatggaaacggtacaaagtctttgttgagaccgcgtagcccgaagggtat

KanR

pJ2076.2_cL (4415 bp) (from 3532-4415 bp)

caatcgatagattgtcgcacctgattgcccgacattatcgcgagcccatttatacccatataaatcagcatccatgttggaatttaatcgcggcctagagcaagacg
gttagctatctaacagcgtggactaacgggctgtaatagcgctcgggtaaatatgggtatatttagtcgtaggtacaaccttaaattagcgccggatctcgttctgc
KanR

tttcccgttgaatatggctcatactcttcctttttcaatattattgaagcatttatcagggttattgtctcatgagcggatacatatttgaatgtatttagaaaaat
aaagggcaacttataccgagtatgagaaggaaaaagttataataacttcgtaaatagtcccaataacagagtactcgcctatgtataaacttacataaatcttttta
KanR

aaacaaataggcatgctagcgcagaaacgtcctagaagatgccaggaggatacttagcagagagacaataaggccggagcgaagccgtttttccataggctccgccc
tttgtttatccgtacgatcgcgtctttgcaggatcttctacggtcctcctatgaatcgtctctctgttattccggcctcgcttcggcaaaaaggtatccgaggcggg
ColA ori

ccctgacgaacatcacgaaatctgacgctcaaatcagtggtggcgaaacccgacaggactataaagataccaggcgtttccccctgatggctccctcttgcgctctc
gggactgcttgtagtgctttagactgcgagtttagtcaccaccgctttgggctgtcctgatatttctatggtccgcaaagggggactaccgagggagaacgcgagag
ColA ori

ctgttcccgtcctgcggcgtccgtgttgtggtggaggctttacccaaatcaccacgtcccgttccgtgtagacagttcgctccaagctgggctgtgtgcaagaaccc
gacaagggcaggacgccgcaggcacaacaccacctccgaaatgggtttagtggtgcagggcaaggcacatctgtcaagcgaggttcgacccgacacacgttcttggg
ColA ori

cccgttcagcccgactgctgcgccttatccggtaactatcatcttgagtccaacccggaaagacacgacaaaacgccactggcagcagccattggtaactgagaatt
gggcaagtcgggctgacgacgcggaataggccattgatagtagaactcaggttgggcctttctgtgctgttttgcggtgaccgtcgtcggtaaccattgactcttaa
ColA ori

agtggatttagatatcgagagtcttgaagtggtggcctaacagaggctacactgaaaggacagtatttggtatctgcgctccactaaagccagttaccaggttaagc
tcacctaaatctatagctctcagaacttcaccaccggattgtctccgatgtgactttcctgtcataaaccatagacgcgaggtgatttcggtcaatggtccaattcg
ColA ori

agttccccaactgacttaaccttcgatcaaaccgcctccccaggcggtttttcgtttacagagcaggagattacgacgatcgtaaaaggatctcaagaagatcctt
tcaaggggttgactgaattggaagctagtttggcggaggggtccgccaaaaagcaaatgtctcgtcctctaatgctgctagcattttcctagagttcttctaggaa
ColA ori

tacggattcccgacaccatcactctaga
atgcctaagggctgtggtagtgagatct
ColA ori

(from 1-1070 bp)

pJ2076.2_cR (4419 bp)

EcoRI SacI

GAATTCATAGGATAGATTCTGGAAACTTTACCGTCCGAGCTCCAGCCTGCGGTCCGGttgacggctagctcagtcctaggtacagtgctagcTCGCTGGGACGCCCG
CTTAAGTATCCTATCTAAGACCTTTGAAATGGCAGGCTCGAGGTCGGACGCCAGGCCaactgccgatcgagtcaggatccatgtcacgatcgAGCGACCCTGCGGGC

Linker_14 Prefix P(BBa_J23100) Suffix

AGAGCCGAATCGCACTTATTTACAGTAGTTCAGCCTGCGGTCCGGccaTACATACTCTAAGATGTGTCTCGCTGGGACGCCCGGGGACTACACTTACGAAACTATTG
TCTCGGCTTAGCGTGAATAAATGTCATCAAGTCGGACGCCAGGCCggtATGTATGAGATTCTACACAGAGCGACCCTGCGGGCCCCTGATGTGAATGCTTTGATAAC

Linker_21 Prefix bs-4 Suffix Linker_1

ATTGCTCAGCCTGCGGTCCGGGTTCACTGCCGTATAGGCAGTCTCAAGCTAGACTCTAGTGGTTTcAGAGCTATGCTGGAAACAGCATAGCAAGTTgAAATAAGGCT
TAACGAGTCGGACGCCAGGCCCAAGTGACGGCATATCCGTCAGAGTTCGATCTGAGATCACCAAAgTCTCGATACGACCTTTGTCGTATCGTTCAAcTTTATTCCGA

Prefix Csy4 site sgRNA-1

XhoI

AGTCCGTTATCAACTTGAAAAAGTGGCACCGAGTCGGTGCGTTCACTGCCGTATAGGCAGTCGCTGGGACGCCCGCTCGAGCAATAAACAGTTGATAGGGCTTCTCC
TCAGGCAATAGTTGAACTTTTTCACCGTGGCTCAGCCACGCAAGTGACGGCATATCCGTCAGCGACCCTGCGGGCGAGCTCGTTATTTGTCAACTATCCCGAAGAGG

sgRNA-1 Csy4 site Suffix Linker_0

NcoI

GTTACCATGGTTCAGCCAAAAAACTTAAGACCGCCGGTCTTGTCCACTACCTTGCAGTAATGCGGTGGACAGGATCGGCGGTTTTCTTTTCTCTTCTCAATTCTTCT
CAATGGTACCAAGTCGGTTTTTTGAATTCTGGCGGCCAGAACAGGTGATGGAACGTCATTACGCCACCTGTCCTAGCCGCCAAAAGAAAAGAGAAGAGTTAAGAAGA

ECK120029600 Terminator

GACCTGTAACGAATAATAGATAGTAAAGTAGTCTCCGATTGAGTTTTCTCTGCCGAGTCCCACCCAGTTCTGTGATTTCAGTAAGTTGGTAATTGATACACTGTTGC
CTGGACATTGCTTATTATCTATCATTTCATCAGAGGCTAACTCAAAAGAGACGGCTCAGGGTGGGTCAAGACACTAAAGTCATTCAACCATTAACTATGTGACAACG

Spacer 1

HindIII

GAGAACTGCTGCCTGGTAGTAGATAGGTTGTTATTGAGTAAGAAGGTAAAGTGAACGAAATCCCTGAAACTGAGACTGTAGAAAATAAGCTTCAGCCTGCGGTCCGG
CTCTTGACGACGGACCATCATCTATCCAACAATAACTCATTCTTCCATTTCACTTGCTTTAGGGACTTTGACTCTGACATCTTTTATTCGAAGTCGGACGCCAGGCC

Spacer 1 Prefix

TTGACAGCTAGCTCAGTCCTAGGTACTGTGCTAGCTCGCTGGGACGCCCGAGATAGCCGTTACACAGGTGACACTTATTTCAGCCTGCGGTCCGGccaCACTAGAGT
AACTGTCGATCGAGTCAGGATCCATGACACGATCGAGCGACCCTGCGGGCTCTATCGGCAATGTGTCCACTGTGAATAAAGTCGGACGCCAGGCCggtGTGATCTCA

P(BBa_J23102) Suffix Linker_24 Prefix bs-1

CTAGCTTGAGATCGCTGGGACGCCCGTGAACAGTTGCTCTGATTGAAACCACGATTCAGCCTGCGGTCCGGTGTAAGAGAAATCCACGAAGGAAAAGGTGAAAGCAG
GATCGAACTCTAGCGACCCTGCGGGCACTTGTCAACGAGACTAACTTTGGTGCTAAGTCGGACGCCAGGCCACATTCTCTTTAGGTGCTTCCTTTTCCACTTTCGTC

bs-1 Suffix Linker_19 Prefix Spacer 2.2

AACAGATAATCAGTAATAGTAGCAACTCAACCACAGGACACTTCCCTCACTCCGAACGAATCACAATACGCTCCAATCTTCAACTACACGATAAAACTGGTAATCCC
TTGTCTATTAGTCATTATCATCGTTGAGTTGGTGTCCTGTGAAGGGAGTGAGGCTTGCTTAGTGTTATGCGAGGTTAGAAGTTGATGTGCTATTTTGACCATTAGGG

Spacer 2.2

pJ2076.2_cR (4419 bp) (from 1071-2247 bp)

BamHI

AACAACCTGACTCACACTCTACTTTCTCAACGAGGAACTTCTTATTCTGATACAATCTCGCTGGGACGCCCGGGATCCAAGAGATTTCTACACGATTGAGCACTGTC
TTGTTGGACTGAGTGTGAGATGAAAGAGTTGCTCCTTGAAGAATAAGACTATGTTAGAGCGACCCTGCGGGCCCTAGGTTCTCTAAAGATGTGCTAACTCGTGACAG

Spacer 2.2 | Suffix | Linker_10

TCAGCCTGCGGTCCGGGTTCACTGCCGTATAGGCAGTAATTTTGTTTAACTTTAAGAAGGAGATATACATATGCGTAAAGGCGAAGAACTGTTTACCGGTGTGGTTC
AGTCGGACGCCAGGCCCAAGTGACGGCATATCCGTCATTAAAACAAATTGAAATTCTTCCTCTATATGTATACGCATTTCCGCTTCTTGACAAATGGCCACACCAAG

Prefix | Csy4 site | RBS | sfGFP

CGATTCTGGTGGAACTGGACGGCGATGTTAATGGTCATAAATTCAGTGTTCGCGGCGAAGGTGAAGGCGATGCGACGAACGGCAAACTGACCCTGAAATTTATCTGC
GCTAAGACCACCTTGACCTGCCGCTACAATTACCAGTATTTAAGTCACAAGCGCCGCTTCCACTTCCGCTACGCTGCTTGCCGTTTGACTGGGACTTTAAATAGACG

sfGFP

ACCACGGGTAAACTGCCGGTCCCGTGGCCGACGCTGGTGACCACGCTGACCTATGGCGTTCAATGTTTTGCGCGTTACCCGGATCACATGAAACAGCACGACTTTTT
TGGTGCCCATTTGACGGCCAGGGCACCGGCTGCGACCACTGGTGCGACTGGATACCGCAAGTTACAAAACGCGCAATGGGCCTAGTGTACTTTGTCGTGCTGAAAAA

sfGFP

CAAATCGGCCATGCCGGAAGGCTATGTGCAGGAACGTACGATTAGCTTTAAAGACGATGGTACGTATAAAACCCGCGCGGAAGTGAAATTCGAAGGCGATACCCTGG
GTTTAGCCGGTACGGCCTTCCGATACACGTCCTTGCATGCTAATCGAAATTTCTGCTACCATGCATATTTTGGGCGCGCCTTCACTTTAAGCTTCCGCTATGGGACC

sfGFP

TTAACCGTATCGAACTGAAAGGTATCGATTTCAAAGAAGACGGCAATATTCTGGGTCATAAACTGGAATATAACTTCAATTCCCACAACGTGTACATCACCGCGGAT
AATTGGCATAGCTTGACTTTCCATAGCTAAAGTTTCTTCTGCCGTTATAAGACCCAGTATTTGACCTTATATTGAAGTTAAGGGTGTTGCACATGTAGTGGCGCCTA

sfGFP

AAACAGAAAAACGGCATTAAAGCCAATTTCAAAATCCGCCATAATGTGGAAGATGGTAGCGTTCAGCTGGCCGACCACTATCAGCAAAACACGCCGATTGGTGATGG
TTTGTCTTTTTGCCGTAATTTCGGTTAAAGTTTTAGGCGGTATTACACCTTCTACCATCGCAAGTCGACCGGCTGGTGATAGTCGTTTTGTGCGGCTAACCACTACC

sfGFP

CCCGGTCCTGCTGCCGGACAATCACTACCTGAGTACCCAGTCCGTGCTGTCAAAAGATCCGAACGAAAAACGTGACCACATGGTCCTGCTGGAATTTGTGACGGCTG
GGGCCAGGACGACGGCCTGTTAGTGATGGACTCATGGGTCAGGCACGACAGTTTTCTAGGCTTGCTTTTTGCACTGGTGTACCAGGACGACCTTAAACACTGCCGAC

sfGFP

CGGGTATCACCCACGGCATGGACGAACTGTATAAAATGTCCCGCCGTAATACTGACGCCATCACAATCCACAGCATCCTGGATTGGATTGAAGACTAATAATCGCTG
GCCCATAGTGGGTGCCGTACCTGCTTGACATATTTTACAGGGCGGCATTATGACTGCGGTAGTGTTAGGTGTCGTAGGACCTAACCTAACTTCTGATTATTAGCGAC

sfGFP | MarAn20

SbfI | KpnI

GGACGCCCGCCTGCAGGCTCGGTACCAAATTCCAGAAAAGAGGCCTCCCGAAAGGGGGGCCTTTTTTCGTTTTGGTCCTAATAGATAAAGGATAGGTCTGGTAGTGT
CCTGCGGGCGGACGTCCGAGCCATGGTTTAAGGTCTTTTCTCCGGAGGGCTTTCCCCCCGGAAAAAAGCAAAACCAGGATTATCTATTTCCTATCCAGACCATCACA

L3S2P21 Terminator | Spacer 2.5

TGTTCGTTCTCGCAGGTAAATCAATAATACTCAGCAGTTCCGTAGACTTTTCAGTGGGACAGGGTAGCGATAACAGATAGATTGTAATAAGACACAGTAGGTGCTCG
ACAAGCAAGAGCGTCCATTTAGTTATTATGAGTCGTCAAGGCATCTGAAAAGTCACCCTGTCCCATCGCTATTGTCTATCTAACATTATTCTGTGTCATCCACGAGC

Spacer 2.5

pJ2076.2_cR (4419 bp) (from 2248-3531 bp)

SalI AatII

TAGTTGCGTGAAGAGAACCGCTCAGGAAATCCAGTCAGAAGTATTGGTAATCGTTGAAAACTCAGTCGACGCACTTACTGAAGACGTCCTATTACACTCGTCGTTGG
ATCAACGCACTTCTCTTGGCGAGTCCTTTAGGTCAGTCTTCATAACCATTAGCAACTTTTGAGTCAGCTGCGTGAATGACTTCTGCAGGATAATGTGAGCAGCAACC

Spacer 2.5 | Spa... 5 | Linker_11

NotI

AAACTGAAGATGCGGCCGCggaaacacagAAAAAAGCCCGCACCTGACAGTGCGGGCTTTTTTTTTcgaccaaaggTAGCGAACGACGAGTCACTGTTGAGGATAAA
TTTGACTTCTACGCCGGCGcctttgtgtcTTTTTTCGGGCGTGGACTGTCACGCCCGAAAAAAAAAgctggtttccATCGCTTGCTGCTCAGTGACAACTCCTATTT

Li...1 | ECK120033737 Terminator | Linker_8

KasI BbvCI AscI

TACTTTCTCTACTAGGCGCCTGTTACACAGGTCCTCAGCGGCGCGCCTTTGTCGGTGAACGCTCTCCTGAGTAGGACAAATCCGCCGGGAGCGGATTTGAACGTTGT
ATGAAAGAGATGATCCGCGGACAATGTGTCCAGGAGTCGCCGCGCGGAAACAGCCACTTGCGAGAGGACTCATCCTGTTTAGGCGGCCCTCGCCTAAACTTGCAACA

Linker_8 | Spa...15

GAAGCAACGGCCCGGAGGGTGGCGGGCAGGACGCCCGCCATAAACTGCCAGGCATCAAACTAAGCAGAAGGCCATCCTGACGGATGGCCTTTTTGCGTTTCAGATCT
CTTCGTTGCCGGGCCTCCCACCGCCCGTCCTGCGGGCGGTATTTGACGGTCCGTAGTTTGATTCGTCTTCCGGTAGGACTGCCTACCGGAAAAACGCAAAGTCTAGA

ACCGGTaaaccagcaatagacataagcggctatttaacgaccctgccctgaaccgacgacaagctgacgaccgggtctccgcaagtggcacttttcggggaaatgtg
TGGCCAtttggtcgttatctgtattcgccgataaattgctgggacgggacttggctgctgttcgactgctggcccagaggcgttcaccgtgaaaagcccctttacac

cgcggaacccctatttgtttatttttctaaatacattcaaatatgtatccgctcatgaattaattcttagaaaaactcatcgagcatcaaatgaaactgcaatttat
gcgccttggggataaacaaataaaaagatttatgtaagtttatacataggcgagtacttaattaagaatctttttgagtagctcgtagtttactttgacgttaaata

KanR

tcatatcaggattatcaataccatatttttgaaaaagccgtttctgtaatgaaggagaaaactcaccgaggcagttccataggatggcaagatcctggtatcggtct
agtatagtcctaatagttatggtataaaaactttttcggcaaagacattacttcctcttttgagtggctccgtcaaggtatcctaccgttctaggaccatagccaga

KanR

gcgattccgactcgtccaacatcaatacaacctattaatttcccctcgtcaaaaataaggttatcaagtgagaaatcaccatgagtgacgactgaatccggtgagaa
cgctaaggctgagcaggttgtagttatgttggataattaaaggggagcagtttttattccaatagttcactctttagtggtactcactgctgacttaggccactctt

KanR

tggcaaaagtttatgcatttctttccagacttgttcaacaggccagccattacgctcgtcatcaaaatcactcgcatcaaccaaaccgttattcattcgtgattgcg
accgttttcaaatacgtaaagaaaggtctgaacaagttgtccggtcggtaatgcgagcagtagttttagtgagcgtagttggtttggcaataagtaagcactaacgc

KanR

cctgagcgagacgaaatacgcggtcgctgttaaaaggacaattacaaacaggaatcgaatgcaaccggcgcaggaacactgccagcgcatcaacaatattttcacct
ggactcgctctgctttatgcgccagcgacaattttcctgttaatgtttgtccttagcttacgttggccgcgtccttgtgacggtcgcgtagttgttataaaagtgga

KanR

gaatcaggatattcttctaatacctggaatgctgttttcccggggatcgcagtggtgagtaaccatgcatcatcaggagtacggataaaatgcttgatggtcggaag
cttagtcctataagaagattatggaccttacgacaaaagggcccctagcgtcaccactcattggtacgtagtagtcctcatgcctattttacgaactaccagccttc

KanR

aggcataaattccgtcagccagtttagtctgaccatctcatctgtaacatcattggcaacgctacctttgccatgtttcagaaacaactctggcgcatcgggcttcc
tccgtatttaaggcagtcggtcaaatcagactggtagagtagacattgtagtaaccgttgcgatggaaacggtacaaagtctttgttgagaccgcgtagcccgaagg

KanR

pJ2076.2_cR (4419 bp) (from 3532-4419 bp)

catacaatcgatagattgtcgcacctgattgcccgacattatcgcgagcccatttatacccatataaatcagcatccatgttggaatttaatcgcggcctagagcaa
gtatgttagctatctaacagcgtggactaacgggctgtaatagcgctcgggtaaatatgggtatatttagtcgtaggtacaaccttaaattagcgccggatctcgtt
KanR

gacgtttcccgttgaatatggctcatactcttcctttttcaatattattgaagcatttatcagggttattgtctcatgagcggatacatatttgaatgtatttagaa
ctgcaaagggcaacttataccgagtatgagaaggaaaaagttataataacttcgtaaatagtcccaataacagagtactcgcctatgtataaacttacataaatctt
KanR

aaataaacaaataggcatgctagcgcagaaacgtcctagaagatgccaggaggatacttagcagagagacaataaggccggagcgaagccgtttttccataggctcc
tttatttgtttatccgtacgatcgcgtctttgcaggatcttctacggtcctcctatgaatcgtctctctgttattccggcctcgcttcggcaaaaaggtatccgagg
ColA ori

gcccccctgacgaacatcacgaaatctgacgctcaaatcagtggtggcgaaacccgacaggactataaagataccaggcgtttccccctgatggctccctcttgcgc
cggggggactgcttgtagtgctttagactgcgagtttagtcaccaccgctttgggctgtcctgatatttctatggtccgcaaaggggggactaccgagggagaacgcg
ColA ori

tctcctgttcccgtcctgcggcgtccgtgttgtggtggaggctttacccaaatcaccacgtcccgttccgtgtagacagttcgctccaagctgggctgtgtgcaaga
agaggacaagggcaggacgccgcaggcacaacaccacctccgaaatgggtttagtggtgcagggcaaggcacatctgtcaagcgaggttcgacccgacacacgttct
ColA ori

accccccgttcagcccgactgctgcgccttatccggtaactatcatcttgagtccaacccggaaagacacgacaaaacgccactggcagcagccattggtaactgag
tggggggcaagtcgggctgacgacgcggaataggccattgatagtagaactcaggttgggcctttctgtgctgttttgcggtgaccgtcgtcggtaaccattgactc
ColA ori

aattagtggatttagatatcgagagtcttgaagtggtggcctaacagaggctacactgaaaggacagtatttggtatctgcgctccactaaagccagttaccaggtt
ttaatcacctaaatctatagctctcagaacttcaccaccggattgtctccgatgtgactttcctgtcataaaccatagacgcgaggtgatttcggtcaatggtccaa
ColA ori

aagcagttccccaactgacttaaccttcgatcaaaccgcctccccaggcggtttttttcgtttacagagcaggagattacgacgatcgtaaaaggatctcaagaagat
ttcgtcaaggggttgactgaattggaagctagtttggcggaggggtccgccaaaaaaagcaaatgtctcgtcctctaatgctgctagcattttcctagagttcttcta
ColA ori

cctttacggattcccgacaccatcactctaga
ggaaatgcctaagggctgtggtagtgagatct
ColA ori

(from 1-1070 bp)

pJ2076.2_TS (4361 bp)

EcoRI SacI

GAATTCATAGGATAGATTCTGGAAACTTTACCGTCCGAGCTCCAGCCTGCGGTCCGGttgacggctagctcagtcctaggtacagtgctagcTCGCTGGGACGCCCG
CTTAAGTATCCTATCTAAGACCTTTGAAATGGCAGGCTCGAGGTCGGACGCCAGGCCaactgccgatcgagtcaggatccatgtcacgatcgAGCGACCCTGCGGGC

Linker_14 Prefix P(BBa_J23100) Suffix

AGAGCCGAATCGCACTTATTTACAGTAGTTCAGCCTGCGGTCCGGccaTACATACTCTAAGATGTGTCTCGCTGGGACGCCCGGGGACTACACTTACGAAACTATTG
TCTCGGCTTAGCGTGAATAAATGTCATCAAGTCGGACGCCAGGCCggtATGTATGAGATTCTACACAGAGCGACCCTGCGGGCCCCTGATGTGAATGCTTTGATAAC

Linker_21 Prefix bs-4 Suffix Linker_1

ATTGCTCAGCCTGCGGTCCGGGTTCACTGCCGTATAGGCAGTCTCAAGCTAGACTCTAGTGGTTTcAGAGCTATGCTGGAAACAGCATAGCAAGTTgAAATAAGGCT
TAACGAGTCGGACGCCAGGCCCAAGTGACGGCATATCCGTCAGAGTTCGATCTGAGATCACCAAAgTCTCGATACGACCTTTGTCGTATCGTTCAAcTTTATTCCGA

Prefix Csy4 site sgRNA-1

XhoI

AGTCCGTTATCAACTTGAAAAAGTGGCACCGAGTCGGTGCGTTCACTGCCGTATAGGCAGTCGCTGGGACGCCCGCTCGAGCAATAAACAGTTGATAGGGCTTCTCC
TCAGGCAATAGTTGAACTTTTTCACCGTGGCTCAGCCACGCAAGTGACGGCATATCCGTCAGCGACCCTGCGGGCGAGCTCGTTATTTGTCAACTATCCCGAAGAGG

sgRNA-1 Csy4 site Suffix Linker_0

NcoI

GTTACCATGGTTCAGCCAAAAAACTTAAGACCGCCGGTCTTGTCCACTACCTTGCAGTAATGCGGTGGACAGGATCGGCGGTTTTCTTTTCTCTTCTCAATTCTTCT
CAATGGTACCAAGTCGGTTTTTTGAATTCTGGCGGCCAGAACAGGTGATGGAACGTCATTACGCCACCTGTCCTAGCCGCCAAAAGAAAAGAGAAGAGTTAAGAAGA

ECK120029600 Terminator

GACCTGTAACGAATAATAGATAGTAAAGTAGTCTCCGATTGAGTTTTCTCTGCCGAGTCCCACCCAGTTCTGTGATTTCAGTAAGTTGGTAATTGATACACTGTTGC
CTGGACATTGCTTATTATCTATCATTTCATCAGAGGCTAACTCAAAAGAGACGGCTCAGGGTGGGTCAAGACACTAAAGTCATTCAACCATTAACTATGTGACAACG

Spacer 1

HindIII

GAGAACTGCTGCCTGGTAGTAGATAGGTTGTTATTGAGTAAGAAGGTAAAGTGAACGAAATCCCTGAAACTGAGACTGTAGAAAATAAGCTTCAGCCTGCGGTCCGG
CTCTTGACGACGGACCATCATCTATCCAACAATAACTCATTCTTCCATTTCACTTGCTTTAGGGACTTTGACTCTGACATCTTTTATTCGAAGTCGGACGCCAGGCC

Spacer 1 Prefix

TTGACAGCTAGCTCAGTCCTAGGTACTGTGCTAGCTCGCTGGGACGCCCGAGATAGCCGTTACACAGGTGACACTTATTTCAGCCTGCGGTCCGGccaCACTAGAGT
AACTGTCGATCGAGTCAGGATCCATGACACGATCGAGCGACCCTGCGGGCTCTATCGGCAATGTGTCCACTGTGAATAAAGTCGGACGCCAGGCCggtGTGATCTCA

P(BBa_J23102) Suffix Linker_24 Prefix bs-1

CTAGCTTGAGATCGCTGGGACGCCCGTGAACAGTTGCTCTGATTGAAACCACGATTCAGCCTGCGGTCCGGGTTCACTGCCGTATAGGCAGCATCTTAGAGTATGTA
GATCGAACTCTAGCGACCCTGCGGGCACTTGTCAACGAGACTAACTTTGGTGCTAAGTCGGACGCCAGGCCCAAGTGACGGCATATCCGTCGTAGAATCTCATACAT

bs-1 Suffix Linker_19 Prefix Csy4 site sgRNA-4 t4

GTTTcAGAGCTATGCTGGAAACAGCATAGCAAGTTgAAATAAGGCTAGTCCGTTATCAACTTGAAAAAGTGGCACCGAGTCGGTGCGTTCACTGCCGTATAGGCAGT
CAAAgTCTCGATACGACCTTTGTCGTATCGTTCAAcTTTATTCCGATCAGGCAATAGTTGAACTTTTTCACCGTGGCTCAGCCACGCAAGTGACGGCATATCCGTCA

sgRNA-4 t4 Csy4 site

pJ2076.2_TS (4361 bp) (from 1071-2247 bp)

BamHI

CGCTGGGACGCCCGGGATCCAAGAGATTTCTACACGATTGAGCACTGTCTCAGCCTGCGGTCCGGGTTCACTGCCGTATAGGCAGTAATTTTGTTTAACTTTAAGAA
GCGACCCTGCGGGCCCTAGGTTCTCTAAAGATGTGCTAACTCGTGACAGAGTCGGACGCCAGGCCCAAGTGACGGCATATCCGTCATTAAAACAAATTGAAATTCTT

Suffix | Linker_10 | Prefix | Csy4 site | RBS

GGAGATATACATATGCGTAAAGGCGAAGAACTGTTTACCGGTGTGGTTCCGATTCTGGTGGAACTGGACGGCGATGTTAATGGTCATAAATTCAGTGTTCGCGGCGA
CCTCTATATGTATACGCATTTCCGCTTCTTGACAAATGGCCACACCAAGGCTAAGACCACCTTGACCTGCCGCTACAATTACCAGTATTTAAGTCACAAGCGCCGCT

RBS | sfGFP

AGGTGAAGGCGATGCGACGAACGGCAAACTGACCCTGAAATTTATCTGCACCACGGGTAAACTGCCGGTCCCGTGGCCGACGCTGGTGACCACGCTGACCTATGGCG
TCCACTTCCGCTACGCTGCTTGCCGTTTGACTGGGACTTTAAATAGACGTGGTGCCCATTTGACGGCCAGGGCACCGGCTGCGACCACTGGTGCGACTGGATACCGC

sfGFP

TTCAATGTTTTGCGCGTTACCCGGATCACATGAAACAGCACGACTTTTTCAAATCGGCCATGCCGGAAGGCTATGTGCAGGAACGTACGATTAGCTTTAAAGACGAT
AAGTTACAAAACGCGCAATGGGCCTAGTGTACTTTGTCGTGCTGAAAAAGTTTAGCCGGTACGGCCTTCCGATACACGTCCTTGCATGCTAATCGAAATTTCTGCTA

sfGFP

GGTACGTATAAAACCCGCGCGGAAGTGAAATTCGAAGGCGATACCCTGGTTAACCGTATCGAACTGAAAGGTATCGATTTCAAAGAAGACGGCAATATTCTGGGTCA
CCATGCATATTTTGGGCGCGCCTTCACTTTAAGCTTCCGCTATGGGACCAATTGGCATAGCTTGACTTTCCATAGCTAAAGTTTCTTCTGCCGTTATAAGACCCAGT

sfGFP

TAAACTGGAATATAACTTCAATTCCCACAACGTGTACATCACCGCGGATAAACAGAAAAACGGCATTAAAGCCAATTTCAAAATCCGCCATAATGTGGAAGATGGTA
ATTTGACCTTATATTGAAGTTAAGGGTGTTGCACATGTAGTGGCGCCTATTTGTCTTTTTGCCGTAATTTCGGTTAAAGTTTTAGGCGGTATTACACCTTCTACCAT

sfGFP

GCGTTCAGCTGGCCGACCACTATCAGCAAAACACGCCGATTGGTGATGGCCCGGTCCTGCTGCCGGACAATCACTACCTGAGTACCCAGTCCGTGCTGTCAAAAGAT
CGCAAGTCGACCGGCTGGTGATAGTCGTTTTGTGCGGCTAACCACTACCGGGCCAGGACGACGGCCTGTTAGTGATGGACTCATGGGTCAGGCACGACAGTTTTCTA

sfGFP

CCGAACGAAAAACGTGACCACATGGTCCTGCTGGAATTTGTGACGGCTGCGGGTATCACCCACGGCATGGACGAACTGTATAAAATGTCCCGCCGTAATACTGACGC
GGCTTGCTTTTTGCACTGGTGTACCAGGACGACCTTAAACACTGCCGACGCCCATAGTGGGTGCCGTACCTGCTTGACATATTTTACAGGGCGGCATTATGACTGCG

sfGFP | MarAn20

SbfI KpnI

CATCACAATCCACAGCATCCTGGATTGGATTGAAGACTAATAATCGCTGGGACGCCCGCCTGCAGGCTCGGTACCAAATTCCAGAAAAGAGGCCTCCCGAAAGGGGG
GTAGTGTTAGGTGTCGTAGGACCTAACCTAACTTCTGATTATTAGCGACCCTGCGGGCGGACGTCCGAGCCATGGTTTAAGGTCTTTTCTCCGGAGGGCTTTCCCCC

MarAn20 | Suffix | L3S2P21 Terminator

GCCTTTTTTCGTTTTGGTCCTAATAGATAAAGGATAGGTCTGGTAGTGTTGTTCGTTCTCGCAGGTAAATCAATAATACTCAGCAGTTCCGTAGACTTTTCAGTGGG
CGGAAAAAAGCAAAACCAGGATTATCTATTTCCTATCCAGACCATCACAACAAGCAAGAGCGTCCATTTAGTTATTATGAGTCGTCAAGGCATCTGAAAAGTCACCC

L3S2P21...inator | Spacer 2.5

ACAGGGTAGCGATAACAGATAGATTGTAATAAGACACAGTAGGTGCTCGTAGTTGCGTGAAGAGAACCGCTCAGGAAATCCAGTCAGAAGTATTGGTAATCGTTGAA
TGTCCCATCGCTATTGTCTATCTAACATTATTCTGTGTCATCCACGAGCATCAACGCACTTCTCTTGGCGAGTCCTTTAGGTCAGTCTTCATAACCATTAGCAACTT

Spacer 2.5

pJ2076.2_TS (4361 bp) (from 2248-3531 bp)

SalI AatII NotI

AACTCAGTCGACGCACTTACTGAAGACGTCCTATTACACTCGTCGTTGGAAACTGAAGATGCGGCCGCggaaacacagAAAAAAGCCCGCACCTGACAGTGCGGGCT
TTGAGTCAGCTGCGTGAATGACTTCTGCAGGATAATGTGAGCAGCAACCTTTGACTTCTACGCCGGCGcctttgtgtcTTTTTTCGGGCGTGGACTGTCACGCCCGA

Spa... 5 Linker_11 ECK120033737 Terminator

KasI BbvCI AscI

TTTTTTTTcgaccaaaggTAGCGAACGACGAGTCACTGTTGAGGATAAATACTTTCTCTACTAGGCGCCTGTTACACAGGTCCTCAGCGGCGCGCCTTTGTCGGTGA
AAAAAAAAgctggtttccATCGCTTGCTGCTCAGTGACAACTCCTATTTATGAAAGAGATGATCCGCGGACAATGTGTCCAGGAGTCGCCGCGCGGAAACAGCCACT

ECK120...nator Linker_8 Spa...15

ACGCTCTCCTGAGTAGGACAAATCCGCCGGGAGCGGATTTGAACGTTGTGAAGCAACGGCCCGGAGGGTGGCGGGCAGGACGCCCGCCATAAACTGCCAGGCATCAA
TGCGAGAGGACTCATCCTGTTTAGGCGGCCCTCGCCTAAACTTGCAACACTTCGTTGCCGGGCCTCCCACCGCCCGTCCTGCGGGCGGTATTTGACGGTCCGTAGTT

ACTAAGCAGAAGGCCATCCTGACGGATGGCCTTTTTGCGTTTCAGATCTACCGGTaaaccagcaatagacataagcggctatttaacgaccctgccctgaaccgacg
TGATTCGTCTTCCGGTAGGACTGCCTACCGGAAAAACGCAAAGTCTAGATGGCCAtttggtcgttatctgtattcgccgataaattgctgggacgggacttggctgc

acaagctgacgaccgggtctccgcaagtggcacttttcggggaaatgtgcgcggaacccctatttgtttatttttctaaatacattcaaatatgtatccgctcatga
tgttcgactgctggcccagaggcgttcaccgtgaaaagcccctttacacgcgccttggggataaacaaataaaaagatttatgtaagtttatacataggcgagtact

attaattcttagaaaaactcatcgagcatcaaatgaaactgcaatttattcatatcaggattatcaataccatatttttgaaaaagccgtttctgtaatgaaggaga
taattaagaatctttttgagtagctcgtagtttactttgacgttaaataagtatagtcctaatagttatggtataaaaactttttcggcaaagacattacttcctct

KanR

aaactcaccgaggcagttccataggatggcaagatcctggtatcggtctgcgattccgactcgtccaacatcaatacaacctattaatttcccctcgtcaaaaataa
tttgagtggctccgtcaaggtatcctaccgttctaggaccatagccagacgctaaggctgagcaggttgtagttatgttggataattaaaggggagcagtttttatt

KanR

ggttatcaagtgagaaatcaccatgagtgacgactgaatccggtgagaatggcaaaagtttatgcatttctttccagacttgttcaacaggccagccattacgctcg
ccaatagttcactctttagtggtactcactgctgacttaggccactcttaccgttttcaaatacgtaaagaaaggtctgaacaagttgtccggtcggtaatgcgagc

KanR

tcatcaaaatcactcgcatcaaccaaaccgttattcattcgtgattgcgcctgagcgagacgaaatacgcggtcgctgttaaaaggacaattacaaacaggaatcga
agtagttttagtgagcgtagttggtttggcaataagtaagcactaacgcggactcgctctgctttatgcgccagcgacaattttcctgttaatgtttgtccttagct

KanR

atgcaaccggcgcaggaacactgccagcgcatcaacaatattttcacctgaatcaggatattcttctaatacctggaatgctgttttcccggggatcgcagtggtga
tacgttggccgcgtccttgtgacggtcgcgtagttgttataaaagtggacttagtcctataagaagattatggaccttacgacaaaagggcccctagcgtcaccact

KanR

gtaaccatgcatcatcaggagtacggataaaatgcttgatggtcggaagaggcataaattccgtcagccagtttagtctgaccatctcatctgtaacatcattggca
cattggtacgtagtagtcctcatgcctattttacgaactaccagccttctccgtatttaaggcagtcggtcaaatcagactggtagagtagacattgtagtaaccgt

KanR

acgctacctttgccatgtttcagaaacaactctggcgcatcgggcttcccatacaatcgatagattgtcgcacctgattgcccgacattatcgcgagcccatttata
tgcgatggaaacggtacaaagtctttgttgagaccgcgtagcccgaagggtatgttagctatctaacagcgtggactaacgggctgtaatagcgctcgggtaaatat

KanR

pJ2076.2_TS (4361 bp) (from 3532-4361 bp)

cccatataaatcagcatccatgttggaatttaatcgcggcctagagcaagacgtttcccgttgaatatggctcatactcttcctttttcaatattattgaagcattt
gggtatatttagtcgtaggtacaaccttaaattagcgccggatctcgttctgcaaagggcaacttataccgagtatgagaaggaaaaagttataataacttcgtaaa
KanR

atcagggttattgtctcatgagcggatacatatttgaatgtatttagaaaaataaacaaataggcatgctagcgcagaaacgtcctagaagatgccaggaggatact
tagtcccaataacagagtactcgcctatgtataaacttacataaatctttttatttgtttatccgtacgatcgcgtctttgcaggatcttctacggtcctcctatga
ColA ori

tagcagagagacaataaggccggagcgaagccgtttttccataggctccgcccccctgacgaacatcacgaaatctgacgctcaaatcagtggtggcgaaacccgac
atcgtctctctgttattccggcctcgcttcggcaaaaaggtatccgaggcgggggggactgcttgtagtgctttagactgcgagtttagtcaccaccgctttgggctg
ColA ori

aggactataaagataccaggcgtttccccctgatggctccctcttgcgctctcctgttcccgtcctgcggcgtccgtgttgtggtggaggctttacccaaatcacca
tcctgatatttctatggtccgcaaagggggactaccgagggagaacgcgagaggacaagggcaggacgccgcaggcacaacaccacctccgaaatgggtttagtggt
ColA ori

cgtcccgttccgtgtagacagttcgctccaagctgggctgtgtgcaagaacccccgttcagcccgactgctgcgccttatccggtaactatcatcttgagtccaac
gcagggcaaggcacatctgtcaagcgaggttcgacccgacacacgttcttgggggcaagtcgggctgacgacgcggaataggccattgatagtagaactcaggttg
ColA ori

ccggaaagacacgacaaaacgccactggcagcagccattggtaactgagaattagtggatttagatatcgagagtcttgaagtggtggcctaacagaggctacactg
ggcctttctgtgctgttttgcggtgaccgtcgtcggtaaccattgactcttaatcacctaaatctatagctctcagaacttcaccaccggattgtctccgatgtgac
ColA ori

aaaggacagtatttggtatctgcgctccactaaagccagttaccaggttaagcagttccccaactgacttaaccttcgatcaaaccgcctccccaggcggtttttttc
tttcctgtcataaaccatagacgcgaggtgatttcggtcaatggtccaattcgtcaaggggttgactgaattggaagctagtttggcggaggggtccgccaaaaaag
ColA ori

gtttacagagcaggagattacgacgatcgtaaaaggatctcaagaagatcctttacggattcccgacaccatcactctaga
caaatgtctcgtcctctaatgctgctagcattttcctagagttcttctaggaaatgcctaagggctgtggtagtgagatct
ColA ori

(from 1-1070 bp)

pJ2077.2 (6061 bp)

ACTTTTCATACTCCCGCCATTCAGAGAAGAAACCAATTGTCCATATTGCATCAGACATTGCCGTCACTGCGTCTTTTACTGGCTCTTCTCGCTAACCAAACCGGTAA
TGAAAAGTATGAGGGCGGTAAGTCTCTTCTTTGGTTAACAGGTATAACGTAGTCTGTAACGGCAGTGACGCAGAAAATGACCGAGAAGAGCGATTGGTTTGGCCATT

CCCCGCTTATTAAAAGCATTCTGTAACAAAGCGGGACCAAAGCCATGACAAAAACGCGTAACAAAAGTGTCTATAATCACGGCAGAAAAGTCCACATTGATTATTTG
GGGGCGAATAATTTTCGTAAGACATTGTTTCGCCCTGGTTTCGGTACTGTTTTTGCGCATTGTTTTCACAGATATTAGTGCCGTCTTTTCAGGTGTAACTAATAAAC

EcoRI

CACGGCGTCACACTTTGCTATGCCATAGCATTTTTATCCATAAGATTAGCGGtTCCTACCTGACGCTTTTTATCGCAACTCTCTACTGTTTCTCCATACCGAATTCA
GTGCCGCAGTGTGAAACGATACGGTATCGTAAAAATAGGTATTCTAATCGCCaAGGATGGACTGCGAAAAATAGCGTTGAGAGATGACAAAGAGGTATGGCTTAAGT

P(BAD) promoter

SacI

TAGGATAGATTCTGGAAACTTTACCGTCCGAGCTCCAGCCTGCGGTCCGGGTTCACTGCCGTATAGGCAGTCTCAAGCTAGACTCTAGTGGTTTcAGAGCTATGCTG
ATCCTATCTAAGACCTTTGAAATGGCAGGCTCGAGGTCGGACGCCAGGCCCAAGTGACGGCATATCCGTCAGAGTTCGATCTGAGATCACCAAAgTCTCGATACGAC

Linker_14 | Prefix | Csy4 site | sgRNA-1

XhoI

GAAACAGCATAGCAAGTTgAAATAAGGCTAGTCCGTTATCAACTTGAAAAAGTGGCACCGAGTCGGTGCGTTCACTGCCGTATAGGCAGTCGCTGGGACGCCCGCTC
CTTTGTCGTATCGTTCAAcTTTATTCCGATCAGGCAATAGTTGAACTTTTTCACCGTGGCTCAGCCACGCAAGTGACGGCATATCCGTCAGCGACCCTGCGGGCGAG

sgRNA-1 | Csy4 site | Suffix

NcoI

GAGCAATAAACAGTTGATAGGGCTTCTCCGTTACCATGGTTCAGCCAAAAAACTTAAGACCGCCGGTCTTGTCCACTACCTTGCAGTAATGCGGTGGACAGGATCGG
CTCGTTATTTGTCAACTATCCCGAAGAGGCAATGGTACCAAGTCGGTTTTTTGAATTCTGGCGGCCAGAACAGGTGATGGAACGTCATTACGCCACCTGTCCTAGCC

Linker_0 | ECK120029600 Terminator

CGGTTTTCTTTTCTCTTCTCAATTCTTCTGACCTGTAACGAATAATAGATAGTAAAGTAGTCTCCGATTGAGTTTTCTCTGCCGAGTCCCACCCAGTTCTGTGATTT
GCCAAAAGAAAAGAGAAGAGTTAAGAAGACTGGACATTGCTTATTATCTATCATTTCATCAGAGGCTAACTCAAAAGAGACGGCTCAGGGTGGGTCAAGACACTAAA

ECK12002...minator | Spacer 1

CAGTAAGTTGGTAATTGATACACTGTTGCGAGAACTGCTGCCTGGTAGTAGATAGGTTGTTATTGAGTAAGAAGGTAAAGTGAACGAAATCCCTGAAACTGAGACTG
GTCATTCAACCATTAACTATGTGACAACGCTCTTGACGACGGACCATCATCTATCCAACAATAACTCATTCTTCCATTTCACTTGCTTTAGGGACTTTGACTCTGAC

Spacer 1

HindIII

TAGAAAATAAGCTTCAGCCTGCGGTCCGGacctgtaggatcgtacaTgCTGACAttgtgagcggataacaaTACTGTttcttcaaTCGCTGGGACGCCCGGTAGTGC
ATCTTTTATTCGAAGTCGGACGCCAGGCCtggacatcctagcatgtAcGACTGTaacactcgcctattgttATGACAaagaagttAGCGACCCTGCGGGCCATCACG

Prefix | P(LUX) promoter | Suffix

TTATCAGACCCAATACTGTTGAACAGCCTGCGGTCCGGGTTCACTGCCGTATAGGCAGGACACATCTTAGAGTATGTAGTTTcAGAGCTATGCTGGAAACAGCATAG
AATAGTCTGGGTTATGACAACTTGTCGGACGCCAGGCCCAAGTGACGGCATATCCGTCCTGTGTAGAATCTCATACATCAAAgTCTCGATACGACCTTTGTCGTATC

Linker_17 | Prefix | Csy4 site | sgRNA-4

pJ2077.2 (6061 bp) (from 1071-2247 bp)

BamHI
CAAGTTgAAATAAGGCTAGTCCGTTATCAACTTGAAAAAGTGGCACCGAGTCGGTGCGTTCACTGCCGTATAGGCAGTCGCTGGGACGCCCGGGATCCAAGAGATTT
GTTCAAcTTTATTCCGATCAGGCAATAGTTGAACTTTTTCACCGTGGCTCAGCCACGCAAGTGACGGCATATCCGTCAGCGACCCTGCGGGCCCTAGGTTCTCTAAA
sgRNA-4 | Csy4 site | Suffix

SbfI KpnI
CTACACGATTGAGCACTGTCTCCTGCAGGCTCGGTACCAAATTCCAGAAAAGAGGCCTCCCGAAAGGGGGGCCTTTTTTCGTTTTGGTCCTAATAGATAAAGGATAG
GATGTGCTAACTCGTGACAGAGGACGTCCGAGCCATGGTTTAAGGTCTTTTCTCCGGAGGGCTTTCCCCCCGGAAAAAAGCAAAACCAGGATTATCTATTTCCTATC
Linker_10 | L3S2P21 Terminator | Spacer 2.5

GTCTGGTAGTGTTGTTCGTTCTCGCAGGTAAATCAATAATACTCAGCAGTTCCGTAGACTTTTCAGTGGGACAGGGTAGCGATAACAGATAGATTGTAATAAGACAC
CAGACCATCACAACAAGCAAGAGCGTCCATTTAGTTATTATGAGTCGTCAAGGCATCTGAAAAGTCACCCTGTCCCATCGCTATTGTCTATCTAACATTATTCTGTG
Spacer 2.5

SalI AatII
AGTAGGTGCTCGTAGTTGCGTGAAGAGAACCGCTCAGGAAATCCAGTCAGAAGTATTGGTAATCGTTGAAAACTCAGTCGACGCACTTACTGAAGACGTCCTATTAC
TCATCCACGAGCATCAACGCACTTCTCTTGGCGAGTCCTTTAGGTCAGTCTTCATAACCATTAGCAACTTTTGAGTCAGCTGCGTGAATGACTTCTGCAGGATAATG
Spacer 2.5 | Spa... 5

NotI
ACTCGTCGTTGGAAACTGAAGATGCGGCCGCggaaacacagAAAAAAGCCCGCACCTGACAGTGCGGGCTTTTTTTTTcgaccaaaggTAGCGAACGACGAGTCACT
TGAGCAGCAACCTTTGACTTCTACGCCGGCGcctttgtgtcTTTTTTCGGGCGTGGACTGTCACGCCCGAAAAAAAAAgctggtttccATCGCTTGCTGCTCAGTGA
Linker_11 | ECK120033737 Terminator | Linker_8

KasI BbvCI AscI
GTTGAGGATAAATACTTTCTCTACTAGGCGCCTGTTACACAGGTCCTCAGCGGCGCGCCTGCTGCCACCGCTGAGCAATAACTAGCgtacgggttttgctgcccgca
CAACTCCTATTTATGAAAGAGATGATCCGCGGACAATGTGTCCAGGAGTCGCCGCGCGGACGACGGTGGCGACTCGTTATTGATCGcatgcccaaaacgacgggcgt
Linker_8 | Spa...15

aacgggctgttctggtgttgctagtttgttatcagaatcgcagatccggcttcaGCCggtttgccggctgaaagcgctatttcttccagaattgccatgatttttc
ttgcccgacaagaccacaacgatcaaacaatagtcttagcgtctaggccgaagtCGGccaaacggccgactttcgcgataaagaaggtcttaacggtactaaaaaag

cccacgggaggcgtcactggctcccgtgttgtcggcagctttgattcgataagcagcatcgcctgtttcaggctgtctatgtgtgactgttgagctgtaacaagttg
gggtgccctccgcagtgaccgagggcacaacagccgtcgaaactaagctattcgtcgtagcggacaaagtccgacagatacacactgacaactcgacattgttcaac

tctcaggtgttcaatttcatgttctagttgctttgttttactggtttcacctgttctattaggtgttacatgctgttcatctgttacattgtcgatctgttcatggt
agagtccacaagttaaagtacaagatcaacgaaacaaaatgaccaaagtggacaagataatccacaatgtacgacaagtagacaatgtaacagctagacaagtacca

gaacagctttGaatgcaccaaaaactcgtaaaagctctgatgtatctatcttttttacaccgttttcatctgtgcatatggacagttttccctttgatatGtaacgg
cttgtcgaaaCttacgtggtttttgagcattttcgagactacatagatagaaaaaatgtggcaaaagtagacacgtatacctgtcaaaagggaaactataCattgcc

tgaacagttgttctacttttgtttgttagtcttgatgcttcactgatagatacaagagccataagaacctcagatccttccgtatttagccagtatgttctctagtg
acttgtcaacaagatgaaaacaaacaatcagaactacgaagtgactatctatgttctcggtattcttggagtctaggaaggcataaatcggtcatacaagagatcac
pSC101 ori

pJ2077.2 (6061 bp) (from 2248-3531 bp)

```
tggttcgttgtttttgcgtgagccatgagaacgaaccattgagatcatActtactttgcatgtcactcaaaaattttgcctcaaaactggtgagctgaatttttgca
accaagcaacaaaaacgcactcggtactcttgcttggtaactctagtaTgaatgaaacgtacagtgagtttttaaaacggagttttgaccactcgacttaaaaacgt
pSC101 ori

gttaaagcatcgtgtagtgtttttcttagtccgttaTgtaggtaggaatctgatgtaatggttgttggtattttgtcaccattcatttttatctggttgttctcaag
caatttcgtagcacatcacaaaaagaatcaggcaatAcatccatccttagactacattaccaacaaccataaaacagtggtaagtaaaaatagaccaacaagagttc
pSC101 ori

ttcggttacgagatccatttgtctatctagttcaacttggaaaatcaacgtatcagtcgggcggcctcgcttatcaaccaccaatttcatattgctgtaagtgttta
aagccaatgctctaggtaaacagatagatcaagttgaaccttttagttgcatagtcagcccgccggagcgaatagttggtggttaaagtataacgacattcacaaat
pSC101 ori

aatctttacttattggtttcaaaacccattggttaagccttttaaactcatggtagttattttcaagcattaacatgaacttaaattcatcaaggctaatctctata
ttagaaatgaataaccaaagttttgggtaaccaattcggaaaatttgagtaccatcaataaaagttcgtaattgtacttgaatttaagtagttccgattagagatat
pSC101 ori

tttgccttgtgagttttcttttgtgttagttcttttaataaccactcataaatcctcatagagtatttgttttcaaaagacttaacatgttccagattatattttat
aaacggaacactcaaaagaaaacacaatcaagaaaattattggtgagtatttaggagtatctcataaacaaaagttttctgaattgtacaaggtctaatataaaata
pSC101 ori

gaatttttttaactggaaaagataaggcaatatctcttcactaaaaactaattctaatttttcgcttgagaacttggcatagtttgtccactggaaaatctcaaagc
cttaaaaaaattgaccttttctattccgttatagagaagtgatttttgattaagattaaaaagcgaactcttgaaccgtatcaaacaggtgaccttttagagtttcg
pSC101 ori

ctttaaccaaaggattcctgatttccacagttctcgtcatcagctctctggttgctttagctaatacaccataagcattttccctactgatgttcatcatctgagcg
gaaattggtttcctaaggactaaaggtgtcaagagcagtagtcgagagaccaacgaaatcgattatgtggtattcgtaaaagggatgactacaagtagtagactcgc
pSC101 ori

tattggttataagtgaacgataccgtccgttctttccttgtagggttttcaatcgtggggttgagtagtgccacacagcataaaattagcttggtttcatgctccgt
ataaccaatattcacttgctatggcaggcaagaaaggaacatcccaaaagttagcaccccaactcatcacggtgtgtcgtattttaatcgaaccaaagtacgaggca
pSC101 ori

taagtcatagcgactaatcgctagttcatttgctttgaaaacaactaattcagacatacatctcaattggtctaggtgattttaatcactataccaattgagatggg
attcagtatcgctgattagcgatcaagtaaacgaaacttttgttgattaagtctgtatgtagagttaaccagatccactaaaattagtgatatggttaactctaccc
pSC101 ori

                SpeI
ctagtcaatgataattactagtccttttcctttgagttgtgggtatctgtaaattctgctagacctttgctggaaaacttgtaaattctgctagaccctctgtaaat
gatcagttactattaatgatcaggaaaaggaaactcaacacccatagacatttaagacgatctggaaacgaccttttgaacatttaagacgatctgggagacattta
pSC101 ori

tccgctagacctttgtgtgttttttttgtttatattcaagtggttataatttatagaataaagaaagaataaaaaaagataaaaagaatagatcccagccctgtgta
aggcgatctggaaacacacaaaaaaaacaaatataagttcaccaatattaaatatcttatttctttcttatttttttctatttttcttatctagggtcgggacacat
pSC101 ori

taactcactactttagtcagttccgcagtattacaaaaggatgtcgcaaacgctgtttgctcctctacaaaacagaccttaaaaccctaaaggcttaagtagcaccc
attgagtgatgaaatcagtcaaggcgtcataatgttttcctacagcgtttgcgacaaacgaggagatgttttgtctggaattttgggatttccgaattcatcgtggg
pSC101 ori
```

pJ2077.2 (6061 bp) (from 3532-4815 bp)

tcgcaagctcgggcaaatcgctgaatattccttttgtctccgaccatcaggcacctgagtcgctgtctttttcgtgacattcagttcgctgcgctcacggctctggc
agcgttcgagcccgtttagcgacttataaggaaaacagaggctggtagtccgtggactcagcgacagaaaaagcactgtaagtcaagcgacgcgagtgccgagaccg
pSC101 ori

agtgaatgggggtaaatggcactacaggcTccttttatggattcatgcaaggaaactacccataatacaagaaaagcccgtcacgggcttctcagggcgttttatgg
tcacttacccccatttaccgtgatgtccgAggaaaatacctaagtacgttcctttgatgggtattatgttcttttcgggcagtgcccgaagagtcccgcaaaatacc
pSC101 ori

cgggtctgctatgtggtgctatctgactttttgctgttcagcagttcctgccctctgattttccagtctgaccacttcggattatcccgtgacaggtcattcagact
gcccagacgatacaccacgatagactgaaaaacgacaagtcgtcaaggacgggagactaaaaggtcagactggtgaagcctaatagggcactgtccagtaagtctga
pSC101 ori

ggctaatgcacccagtaaggcagcggtatcatcaacaggcttacccgtcttactgtccctagtgcttggattctcaccaataaaaaacgcccggcggcaaccgagcg
ccgattacgtgggtcattccgtcgccatagtagttgtccgaatgggcagaatgacagggatcacgaacctaagagtggttattttttgcgggccgccgttggctcgc
pSC101 ori

ttctgaacaaatccagatggagttctgaggtcattactggatctatcaacaggagtccaagcgcgtaaacttggtctgacagttaccaatgcttaatcagtgaggca
aagacttgtttaggtctacctcaagactccagtaatgacctagatagttgtcctcaggttcgcgcatttgaaccagactgtcaatggttacgaattagtcactccgt
AmpR

cctatctcagcgatctgtctatttcgttcatccatagttgcctgactccccgtcgtgtagataactacgatacgggagggcttaccatctggccccagtgctgcaat
ggatagagtcgctagacagataaagcaagtaggtatcaacggactgaggggcagcacatctattgatgctatgccctcccgaatggtagaccggggtcacgacgtta
AmpR

gataccgcgagacccacgctcaccggctccagatttatcagcaataaaccagccagccggaagggccgagcgcagaagtggtcctgcaactttatccgcctccatcc
ctatggcgctctgggtgcgagtggccgaggtctaaatagtcgttatttggtcggtcggccttcccggctcgcgtcttcaccaggacgttgaaataggcggaggtagg
AmpR

agtctattaattgttgccgggaagctagagtaagtagttcgccagttaatagtttgcgcaacgttgttgccattgctacaggcatcgtggtgtcacgctcgtcgttt
tcagataattaacaacggcccttcgatctcattcatcaagcggtcaattatcaaacgcgttgcaacaacggtaacgatgtccgtagcaccacagtgcgagcagcaaa
AmpR

ggtatggcttcattcagctccggttcccaacgatcaaggcgagttacatgatcccccatgttgtgcaaaaaagcggttagctccttcggtcctccgatcgttgtcag
ccataccgaagtaagtcgaggccaagggttgctagttccgctcaatgtactagggggtacaacacgttttttcgccaatcgaggaagccaggaggctagcaacagtc
AmpR

aagtaagttggccgcagtgttatcactcatggttatggcagcactgcataattctcttactgtcatgccatccgtaagatgcttttctgtgactggtgagtactcaa
ttcattcaaccggcgtcacaatagtgagtaccaataccgtcgtgacgtattaagagaatgacagtacggtaggcattctacgaaaagacactgaccactcatgagtt
AmpR

ccaagtcattctgagaatagtgtatgcggcgaccgagttgctcttgcccggcgtcaatacgggataataccgcgccacatagcagaactttaaaagtgctcatcatt
ggttcagtaagactcttatcacatacgccgctggctcaacgagaacgggccgcagttatgccctattatggcgcggtgtatcgtcttgaaattttcacgagtagtaa
AmpR

ggaaaacgttcttcggggcgaaaactctcaaggatcttaccgctgttgagatccagttcgatgtaacccactcgtgcacccaactgatcttcagcatcttttacttt
ccttttgcaagaagccccgcttttgagagttcctagaatggcgacaactctaggtcaagctacattgggtgagcacgtgggttgactagaagtcgtagaaaatgaaa
AmpR

pJ2077.2 (6061 bp) (from 4816-6061 bp)

```
caccagcgtttctgggtgagcaaaaacaggaaggcaaaatgccgcaaaaaagggaataagggcgacacggaaatgttgaatactcatactcttcctttttcaatatt
gtggtcgcaaagacccactcgtttttgtccttccgttttacggcgttttttcccttattcccgctgtgcctttacaacttatgagtatgagaaggaaaaagttataa
AmpR

attgaagcatttatcagggttattgtctcatgagcggatacatatttgaatgtatttagaaaaataaacaaataggggttccgcgcacatttccccgaaaagtgcca
taacttcgtaaatagtcccaataacagagtactcgcctatgtataaacttacataaatctttttatttgtttatccccaaggcgcgtgtaaaggggcttttcacggt

ccttcactctagatttcagtgcaatttatctcttcaaatgtagcacctgaagtcagccccatacgatataagttgtaattctcatgttagtcatgccccgcgcccac
ggaagtgagatctaaagtcacgttaaatagagaagtttacatcgtggacttcagtcggggtatgctatattcaacattaagagtacaatcagtacggggcgcgggtg

cggaaggagctgactgggttgCTCCTAgGGTCTGATTCGTTACCAATTATGACAACTTGACGGCTACATCATTCACTTTTTCTTCACAACCGGCACGGAACTCGCTC
gccttcctcgactgacccaacGAGGATcCCAGACTAAGCAATGGTTAATACTGTTGAACTGCCGATGTAGTAAGTGAAAAAGAAGTGTTGGCCGTGCCTTGAGCGAG
araC

GGGCTGGCCCCGGTGCATTTTTTAAATACCCGCGAGAAATAGAGTTGATCGTCAAAACCAACATTGCGACCGACGGTGGCGATAGGCATCCGGGTGGTGCTCAAAAG
CCCGACCGGGGCCACGTAAAAAATTTATGGGCGCTCTTTATCTCAACTAGCAGTTTTGGTTGTAACGCTGGCTGCCACCGCTATCCGTAGGCCCACCACGAGTTTTC
araC

CAGCTTCGCCTGGCTGATACGTTGGTCCTCGCGCCAGCTTAAGACGCTAATCCCTAACTGCTGGCGGAAAAGATGTGACAGACGCGACGGCGACAAGCAAACATGCT
GTCGAAGCGGACCGACTATGCAACCAGGAGCGCGGTCGAATTCTGCGATTAGGGATTGACGACCGCCTTTTCTACACTGTCTGCGCTGCCGCTGTTCGTTTGTACGA
araC

GTGCGACGCTGGCGATATCAAAATTGCTGTCTGCCAGGTGATCGCTGATGTACTGACAAGCCTCGCGTACCCGATTATCCATCGGTGGATGGAGCGACTCGTTAATC
CACGCTGCGACCGCTATAGTTTTAACGACAGACGGTCCACTAGCGACTACATGACTGTTCGGAGCGCATGGGCTAATAGGTAGCCACCTACCTCGCTGAGCAATTAG
araC

GCTTCCATGCGCCGCAGTAACAATTGCTCAAGCAGATTTATCGCCAGCAGCTCCGAATAGCGCCCTTCCCCTTGCCCGGCGTTAATGATTTGCCCAAACAGGTCGCT
CGAAGGTACGCGGCGTCATTGTTAACGAGTTCGTCTAAATAGCGGTCGTCGAGGCTTATCGCGGGAAGGGGAACGGGCCGCAATTACTAAACGGGTTTGTCCAGCGA
araC

GAAATGCGGCTGGTGCGCTTCATCCGGGCGAAAGAACCCCGTATTGGCAAATATTGACGGCCAGTTAAGCCATTCATGCCAGTAGGCGCGCGGACGAAAGTAAACCC
CTTTACGCCGACCACGCGAAGTAGGCCCGCTTTCTTGGGGCATAACCGTTTATAACTGCCGGTCAATTCGGTAAGTACGGTCATCCGCGCGCCTGCTTTCATTTGGG
araC

ACTGGTGATACCATTCGCGAGCCTCCGGATGACGACCGTAGTGATGAATCTCTCCTGGCGGGAACAGCAAAATATCACCCGGTCGGCAAACAAATTCTCGTCCCTGA
TGACCACTATGGTAAGCGCTCGGAGGCCTACTGCTGGCATCACTACTTAGAGAGGACCGCCCTTGTCGTTTTATAGTGGGCCAGCCGTTTGTTTAAGAGCAGGGACT
araC

TTTTTCACCACCCCCTGACCGCGAATGGTGAGATTGAGAATATAACCTTTCATTCCCAGCGGTCGGTCGATAAAAAAATCGAGATAACCGTTGGCCTCAATCGGCGT
AAAAAGTGGTGGGGGACTGGCGCTTACCACTCTAACTCTTATATTGGAAAGTAAGGGTCGCCAGCCAGCTATTTTTTTAGCTCTATTGGCAACCGGAGTTAGCCGCA
araC

TAAACCCGCCACCAGATGGGCATTAAACGAGTATCCCGGCAGCAGGGGATCATTTTGCGCTTCAGCCAT
ATTTGGGCGGTGGTCTACCCGTAATTTGCTCATAGGGCCGTCGTCCCCTAGTAAAACGCGAAGTCGGTA
araC
```
