# Supplementary material for: Multistable and dynamic CRISPRi-based synthetic circuits
Source: Nat Commun. 2020 Jun 2;11:2746. doi: 10.1038/s41467-020-16574-1 (PMC7265303; doi:10.1038/s41467-020-16574-1)
Supplement: Supplementary file 5 — Reporting Summary [file 41467_2020_16574_MOESM5_ESM.pdf]

nature research

Corresponding author(s): Yolanda Schaerli

Last updated by author(s): May 4, 2020

Reporting Summary

Nature Research wishes to improve the reproducibility of the work that we publish. This form provides structure for consistency and transparency in reporting. For further information on Nature Research policies, see Authors & Referees and the Editorial Policy Checklist .

Statistics

For all statistical analyses, confirm that the following items are present in the figure legend, table legend, main text, or Methods section.

n/a	Confirmed	
☐	☒	The exact sample size (*n*) for each experimental group/condition, given as a discrete number and unit of measurement
☐	☒	A statement on whether measurements were taken from distinct samples or whether the same sample was measured repeatedly
☒	☐	The statistical test(s) used AND whether they are one- or two-sided<br>*Only common tests should be described solely by name; describe more complex techniques in the Methods section.*
☒	☐	A description of all covariates tested
☒	☐	A description of any assumptions or corrections, such as tests of normality and adjustment for multiple comparisons
☐	☒	A full description of the statistical parameters including central tendency (e.g. means) or other basic estimates (e.g. regression coefficient) AND variation (e.g. standard deviation) or associated estimates of uncertainty (e.g. confidence intervals)
☒	☐	For null hypothesis testing, the test statistic (e.g. *F*, *t*, *r*) with confidence intervals, effect sizes, degrees of freedom and *P* value noted<br>*Give P values as exact values whenever suitable.*
☒	☐	For Bayesian analysis, information on the choice of priors and Markov chain Monte Carlo settings
☒	☐	For hierarchical and complex designs, identification of the appropriate level for tests and full reporting of outcomes
☒	☐	Estimates of effect sizes (e.g. Cohen's *d*, Pearson's *r*), indicating how they were calculated

Our web collection on statistics for biologists contains articles on many of the points above.

Software and code

Policy information about availability of computer code

Data collection	Gen5 3.04, Leica Application Suite X 3.4.2.18368, Amersham Typhoon 1.1.0.7, BD FACSDiva 8.0.
Data analysis	RStudio 1.0.143 (running R 3.4.0), Fiji - ImageJ 2.0.0, FlowJo 10.5.2, Matlab 2018b (including BioSwitch toolbox 1.0.0).

For manuscripts utilizing custom algorithms or software that are central to the research but not yet described in published literature, software must be made available to editors/reviewers. We strongly encourage code deposition in a community repository (e.g. GitHub). See the Nature Research guidelines for submitting code & software for further information.

Data

Policy information about availability of data

All manuscripts must include a data availability statement. This statement should provide the following information, where applicable:

- Accession codes, unique identifiers, or web links for publicly available datasets
- A list of figures that have associated raw data
- A description of any restrictions on data availability

The source data underlying Fig. 1, 2, 4 and 5 and Supplementary Fig. 1-4 and 6 are provided as a Source Data file.
The plasmids used in this study (Supplementary Table 2) and their annotated sequences (Supplementary Data 1) are available through Addgene 124421, 124422, and 140664-140689.

Field-specific reporting

Please select the one below that is the best fit for your research. If you are not sure, read the appropriate sections before making your selection.

Life sciences study design

All studies must disclose on these points even when the disclosure is negative.

Sample size	No statistical sample size calculation was performed. Sample sizes were similar to or larger than previous publications in the field and sufficient for our claims:<br><br>- For NOT gates: Nielsen, A.A. & Voigt, C.A. Mol. Syst. Biol. 10, 763 (2014).<br>- For the toggle switch: Gardner, T.S., Cantor, C.R. & Collins, J.J. Nature 403, 339 (2000).<br>- For stripe networks: Schaerli, Y. et al. Nat. Commun. 5, 4905 (2014).<br>- For the oscillator: Niederholtmeyer, H. et al. eLife 4, e09771 (2015).
Data exclusions	No data were excluded.
Replication	Experiments were performed using at least three biological replicates. All data were reliably replicated.
Randomization	Biological replicates were obtained from cultures inoculated from single colonies chosen randomly from agar plates.
Blinding	For Supplementary Fig. 1, one person performed the experiment and acquired data, and another one analyzed data and guessed the L-arabinose concentration based on fluorescence output levels. Blinding was not relevant for the rest of the experiments since they did not involve any strain allocation to particular treatment groups and subsequent group comparison.

Reporting for specific materials, systems and methods

We require information from authors about some types of materials, experimental systems and methods used in many studies. Here, indicate whether each material, system or method listed is relevant to your study. If you are not sure if a list item applies to your research, read the appropriate section before selecting a response.

Materials & experimental systems

n/a	Involved in the study
☒	☐ Antibodies
☒	☐ Eukaryotic cell lines
☒	☐ Palaeontology
☒	☐ Animals and other organisms
☒	☐ Human research participants
☒	☐ Clinical data

Methods

n/a	Involved in the study
☒	☐ ChIP-seq
☐	☒ Flow cytometry
☒	☐ MRI-based neuroimaging

Flow Cytometry

Plots

Confirm that:

☒ The axis labels state the marker and fluorochrome used (e.g. CD4-FITC).

☒ The axis scales are clearly visible. Include numbers along axes only for bottom left plot of group (a 'group' is an analysis of identical markers).

☒ All plots are contour plots with outliers or pseudocolor plots.

☒ A numerical value for number of cells or percentage (with statistics) is provided.

Methodology

Sample preparation	Single colonies were used to inoculate 2 ml of selective EZ medium (Teknova) containing 0.2% Ara or 10 µM AHL and incubated at 37° C for 10 h in a tilted 200 rpm shaker. Next, cells were pelleted at 5000 rcf for 10 min and resuspended in selective EZ medium free of inducers; 1 µl of the resuspension was used to inoculate 120 µl of inducer-free selective EZ in a 96-well plate, which was incubated at 37° C for 12 h with double-orbital shaking in a Synergy H1 microplate reader (Biotek). Cells were then diluted 1:120 in 120 µl of selective EZ containing the opposed inducer (10 µM AHL or 0.2% Ara) and grown for 10 h more in the microplate reader under identical conditions. Cells were then diluted into an inducer-free medium as before (resuspension in inducer-free selective EZ followed by 1:120 dilution in the same medium) and incubated for 12 h more in the microplate reader under identical conditions. At the end of each induction period (inducer 1, no inducer, inducer 2, no inducer) a sample was taken, diluted 1:100 in phosphate-buffered saline (PBS) and analyzed with a BD LSRFortessa flow cytometer using a 488 nm laser in combination with FITC filter for sfGFP fluorescence determination.
Instrument	BD LSRFortessa Cell Analyzer, Model No.: 649225B2.

Software	BD FACSDiva 6.2 software was used for data acquisition, FlowJo 10.5.2 was used for data export, and RStudio 1.0.143 (running R 3.4.0) was used for gating and plotting.
Cell population abundance	20,000 events were analyzed per sample.
Gating strategy	Green cells were gated (green > 330 FITC-H a.u.) in the FITC-H histogram to differentiate the two states (green and non-green) using non-fluorescent cells as control. The gating was performed in RStudio 1.0.143 (running R 3.4.0).

☒ Tick this box to confirm that a figure exemplifying the gating strategy is provided in the Supplementary Information.
