# Supplementary material for: Multistable and dynamic CRISPRi-based synthetic circuits
Source: Nat Commun. 2020 Jun 2;11:2746. doi: 10.1038/s41467-020-16574-1 (PMC7265303; doi:10.1038/s41467-020-16574-1)
Supplement: Supplementary file 6 — Description of Additional Supplementary Files [file 41467_2020_16574_MOESM6_ESM.pdf]

Title: Supplementary Movie 1
Description: CRISPRlator-driven oscillations of a bacterial population in a microfluidic device. The movie is sped up 7200 times.

Title: Supplementary Data 1
Description: Fully annotated plasmid sequences.
